
JOURNAL INFORMATION
==============================
NLM Title Abbreviation: JBMR Plus
Journal ID: JBMR Plus
NLM Journal ID: 101707013
Journal ID: jbmrplus

JBMR Plus

EISSN: 2473-4039
Publisher: Oxford University Press

ARTICLE INFORMATION
==============================
PMCID: PMC11325577
DOI: 10.1093/jbmrpl/ziae105
Article ID: ziae105
Article version: 1
Subjects: Abstracts, AcademicSubjects/MED00010, AcademicSubjects/MED00160, AcademicSubjects/MED00250

Abstracts of the 11th International Conference on Children's Bone Health

Publication date: 2024 Aug
Electronic publication date: 2024 Aug 15
Volume: 8
Issue: Suppl 2
First page: ii1
Last page: ii399
Copyright: © The Author(s) 2024. Published by Oxford University Press on behalf of the American Society for Bone and Mineral Research.
Copyright year: 2024
License: This is an Open Access article distributed under the terms of the Creative Commons Attribution License (https://creativecommons.org/licenses/by/4.0/), which permits unrestricted reuse, distribution, and reproduction in any medium, provided the original work is properly cited.
License URL: https://creativecommons.org/licenses/by/4.0/

Page count: 1

==============================

ICCBH

11th INTERNATIONAL CONFERENCE ON CHILDREN'S BONE HEALTH

22-25 JUNE 2024

SALZBURG | AUSTRIA

### Book of Abstracts

### Salzburg, Austria

### Saturday 22 to Tuesday 25 June 2024

### Invited Speaker Talk Summaries

### Oral Communication Presentation Abstracts

### Poster Abstracts

### Overview of the ICCBH 2024 Conference

From Saturday 22 to Tuesday 25 June 2024 a total of 470 delegates from over 45 countries attended the 11th International Conference on Children's Bone Health (ICCBH) in the beautiful city of Salzburg, Austria, arranged by International Society for Children's Bone Health (ISCBH).

The meeting was held at Salzburg Congress, our selected conference venue in the heart of the city. The meeting was preceded by three pre-conference sessions, including the well- attended ISCBH ERN BOND Achondroplasia Session, a stimulating New Investigators Workshop and collaborative Allied Health Professionals Networking Session.

Sponsored workshops throughout the meeting covered "A Leap Forward: Improving Diagnosis and Management of Pediatric Patients With Hypophosphatasia (HPP)", "From infancy to adolescence: reshaping achondroplasia management with vosoritide▼" and "Recognizing ENPP1 Deficiency An Overlooked Cause of Hypophosphatemic Rickets".

The meeting featured the ever-popular Challenge the Expert clinical case review, 8 featured Symposia covering hot topics in paediatric bone diseases, 12 Meet the Expert sessions, 29 Oral Communication presentations, 222 Posters, and 15 sponsors and exhibitors. We were also joined by 4 Patient Advisory groups.

The posters and exhibition hall were all located in the same space which enabled a great networking opportunity and exchange of ideas for all. In addition, dedicated "networking hives" were held towards the end of the meeting to facilitate collaborations around a variety of challenging topics.

Thomas Carpenter (New Haven, USA) was the recipient of the prestigious Charles Slemenda Award for Research Excellence in the field of Pediatric Bone Disorders. Dr Carpenter provided an overview of his successful career and his award was presented by the ICCBH Programme Chair, Outi Mäkitie (Helsinki, Finland)

Sare Betul Kaygusuz (Istanbul, Turkey) was the recipient of the ICCBH Maria Luisa Bianchi Award, introduced to recognise the work of female researchers and healthcare professionals towards the beginning of their careers, who have already made a significant contribution to the field of paediatric bone health, and who are based in low and middle income countries.

Feedback from delegates on the meeting was resoundingly positive, and the ISCBH is now actively planning its next meetings including the 3rd ISCBH Bone School (28-30 May 2025, Annecy, France) and the 12th ICCBH which will be held in the Summer of 2026 in Montreal, Canada.

### ISCBH Executive Committee

Frank Rauch – President (Montreal, Canada)
Adalbert Raimann – Treasurer (Vienna, Austria)
Ciara McDonnell – Secretary (Dublin, Ireland)

### ISCBH Steering Committee

Antonella Forlino (Pavia, Italy)
Wolfgang Högler (Linz, Austria)
Alex Ireland (Manchester, UK)
Outi Mäkitie (Helsinki, Finland)
Craig Munns (Brisbane, Australia)
Leanne Ward (Ottowa, Canada)
Alison Boyce (Bethesda, USA)
Marie-Eve Robinson (Ottowa, Canada)

### ICCBH 2024 Programme Organising Committee

Outi Mäkitie, Chair (Helsinki, Finland)
Erik Imel, Co-Chair (Indianapolis, USA)
Alex Ireland (Manchester, UK)
Peter Simm (Melbourne, Australia)
Antonella Forlino (Pavia, Italy)
Cathleen Raggio (New York, USA)

### ICCBH 2024 Abstracts

Abstracts are published as submitted by the authors. All parties will make reasonable efforts to present educational subject matter in a scientific, balanced and unbiased way. However, participants must always use their own judgment and professional opinion when considering future application of this information, particularly as it may relate to patient diagnostic or treatment decisions.

Statements and descriptions are informational only and are not made or given as a warranty. The views, opinions and statements made at the conference are solely those of the presenters and may not reflect the views of the Organisers. Furthermore, participants should bear in mind that presenters may have vested interests in the concepts and products they discuss.

### Invited Speaker Talk Summaries

### S1.1

Details to follow

### S1.2

Details to follow

### Keynote Lecture 1 – Sunday

### Bone as an endocrine organ: From animal models to the clinic

Roberto Civitelli (Washington University, St Louis (MO), USA)

In addition to its mechanical, protective, and metabolic functions, the skeleton is also the source of secretory products with endocrine action. One such hormone is FGF-23, which is primarily produced by osteocytes and acts on kidney proximal tubule cells where it inhibits phosphorus reabsorption resulting in hypophosphatemia. Early studies in mouse models showed that the bone matrix protein, osteocalcin stimulates insulin production and increases insulin sensitivity in peripheral organs, thus linking bone turnover with energy metabolism. However, more recent data using different genetically engineered murine models have not been able to replicate these findings, and epidemiological data in humans found weak correlations between circulating osteocalcin and fasting glucose or diabetes prevalence. A potential role of the Wnt inhibitor, sclerostin, in energy storage and utilization has emerged from preclinical and clinical studies. Mice genetically deficient in sclerostin have shown improved glucose tolerance, insulin sensitivity, lower adiposity, and partial resistance to obesogenic diets. Similarly, the sclerostin-resistant Lrp5A214V mutation improved glucose tolerance and delayed the onset of diabetes in a genetic mouse model of type I diabetes. It also decreased insulin content in pancreatic beta-cells, suggesting that Wnt agonism (or low circulating sclerostin) activates insulin-independent glucose regulatory mechanisms. In humans, circulating sclerostin and glycated hemoglobin were inversely correlated in subjects with type 2 diabetes. However, serum sclerostin correlated poorly with parameters of glucose homeostasis in cross-sectional analyses of unselected populations. Canonical Wnt signaling may modulate glucose utilization and adiposity in special conditions requiring high energy expenditure, such as very active bone remodeling.

### S2.1

### Osteocytes as mechanosensory cells

Alex Robling (Indiana University, Indianapolis, USA)

Mechanical loading is essential to maintain normal bone metabolism and the balance between bone formation and resorption. The cellular mechanisms that control bone cell mechanotransduction are not fully understood , but several key pathways have been identified in osteocytes. One such mechanism involves Wnt signaling—a pathway involved in many physiologic processes including body axis specification/patterning, and morphogenic signaling. The first clues of WNT's role in bone biology began with several genetic studies that sought to identify the cause of Osteoporosis Pseudoglioma (OPPG), an autosomal recessive disease characterized by early-onset osteoporosis and blindness. Mutant mouse studies revealed that loss-of-function mutation in the WNT co-receptor LDL-Receptor-related Protein 5 (Lrp5) caused OPPG, but perhaps more importantly, that osteocytic Wnt signaling through Lrp5 is crucial for load-induced bone formation. Moreover, mechanical loading strongly regulates Sost/sclerostin expression—a potent inhibitor of Wnt/Lrp5 signaling. Sost, and its protein product sclerostin, are nearly exclusive to the osteocytes—the cell postulated to be the tissue's sensor apparatus in transducing mechanical loading events. The suppression of sclerostin expression by mechanical loading may provide a mechanism by which Wnt signaling is enhanced and bone formation is enacted. Furthermore, Wnt signaling modulation via Sost/sclerostin also appears to modulate the process of disuse osteoporosis. As the mechanisms of mechanical signal transduction in osteocytes become revealed, a greater range of targetable molecules will be manifest, that might ultimately be exploited to improve bone health among patients with a wide range of skeletal disease.

### S2.2

### What's new in bone cells, the story of osteomorphs

Duncan Bassett (Imperial College, London, UK)

Osteoclasts are large multinucleated bone-resorbing cells, formed by the fusion of monocyte/macrophage precursors, that were previously thought to undergo apoptosis following completion of bone resorption. However, detailed in vivo studies, using intravital imaging, revealed that osteoclasts have an alternative fate and can also undergo fission into daughter cells called osteomorphs. Single-cell RNA-seq analysis demonstrated that these osteomorphs were transcriptionally distinct from osteoclasts and macrophages. Moreover, these "osteomorph genes" resulted in skeletal phenotypes when deleted in mice, caused human monogenic skeletal disorders when mutated, and were associates with BMD in GWAS. Thus, abnormalities of osteoclasts/osteomorph recycling represent a novel mechanism of skeletal disease and also a novel targeted for its treatment.

### Charles Slemenda Lecture

### Searching for XLH: Observations, hypotheses, mechanisms, and the challenges ahead

Thomas Carpenter (Yale University School of Medicine, New Haven, USA)

Mineral metabolism and skeletal health have often focused on calcium availability, however significant developments have occurred over the past decade with respect to various disorders of phosphorus metabolism in children. Albeit relatively rare, the study of these disorders has revealed an enormous amount of new information regarding the elaborate systems that have evolved to maintain normal phosphorus homeostasis, essentially changing the approach to care. This talk will review the speaker's introduction to a variety of patients with disorders of phosphorus metabolism, and the lessons learned from those encounters.

X-linked Hypophosphatemia (XLH), due to loss of function variants in PHEX, serves as the prototype disorder of renal phosphate wasting, and is the most frequently encountered of these conditions. The study of XLH has brought to light the role of the endocrine Fibroblast Growth Factor, FGF23, as a physiologic mediator of total body phosphorus homeostasis. The discovery that osteocytes have the capacity to recognize states of phosphorus excess and/or demand, and in response, regulate the secretion of FGF23, which in turn, regulates renal tubular handling of phosphate, has been one of the major advances of the past few decades. This discovery revealed a specific molecular mediator of disease amenable to direct pharmaceutical targeting, and led to the subsequent development of burosumab, an inhibitory antibody to FGF23, now used to treat XLH.

Presentation and management of selected heritable FGF23-mediated and FGF23-independent disorders of phosphorus homeostasis will be discussed, as well as lessons learned from acquired forms of hypopophosphatemia.

### S3.1

Details to follow

### S3.2

Details to follow

### S3.3

### How has HRpQCT advanced bone assessment in children and adolescents?

Kate Ward (University of Southampton, Southampton, UK)

Clinically, dual energy X-ray absorptiometry is the gold standard for bone health assessment in children and adolescents. Guidelines exist to overcome the limitations of DXA in assessment of the growing child and adolescent. However, two-dimensional technique to assess in detail bone growth remain, for example, it is still not possible to assess cortical and trabecular compartments separately, nor in detail to obtain assessments of bone microarchitecture, all important components of bone strength.  Over the last two decades, the evolution of peripheral quantitative computed tomography has led to a step-change in the detail in which we can assess developing bone. High resolution peripheral quantitative computed tomography (HR-pQCT) has been applied clinically and in research studies of children and adolescents.  Never has the in-vivo assessment of the growing skeleton in such high resolution been possible. The International Society for Clinical Densitometry 2019 guidelines, state the method may be used clinically but only where appropriate reference data and expertise are available (ISCD Pediatric positions 2019). Recent expert recommendations have been published for the use of HRpQCT in pediatric research (Gabel et al, 2023). This talk will review technical considerations for the application of HRpQCT in pediatric populations, discuss how the method has advanced skeletal assessment in healthy children and adolescents and those with chronic diseases and look to the future for the application of the technique.

### S4.1

### Multidisciplinary approach to giant cell tumour of bone

Professor Craig Munns (The University of Queensland, Brisbane, Australia)

The most common giant cell tumours of bone in children are central giant cell granulomas (CGCG) (1). These are rare lesions that are osteolytic and benign, but often locally aggressive and their growth is mediated by nuclear factor-kappa B ligand (RANKL). Optimal management requires a coordinated interdisciplinary team often consisting of maxillofacial surgeon, dentist, radiologist, nuclear medicine physician, endocrinology, oncologist and nursing. Other important specialists include pathologists.

Surgical curettage is the only curative therapy, but this may not be possible in extensive lesions and where resection carries high morbidity, especially in growing children (1). There has been growing interest in the use of Denosumab in the medical management of lesions unsuitable for resection. Denosumab is a fully humanised monoclonal antibody to RANKL and a powerful inhibitor of bone resorption. In CGCG it has been demonstrated to halt metabolic activity and result in ossification of the lesions. Denosumab use is also associated with disturbance in mineral homeostasis and rapid accumulation of bone throughout the skeleton, most notably in the metaphysis.  Cessation of therapy is associated with a rapid return in metabolic activity in the GCGC and systemic bone reportion with hypercalcaemia that can be difficult to manage. As such close monitoring of children during and post-Denosumab therapy is required.

A collaborative of international experts recently published guidelines on the management of RANKL-mediated bone lesions with Denosumab that highlighted that its optimal use as an adjuvant therapy and timing of definitive surgery remains to be determined (2).

1. Vanderniet JA, Wall CL, Mullins A, London K, Lim L, Hibbert S, Briody J, Padhye B, Poon M, Biggin A, Dalla-Pozza L, Munns CF. Denosumab for giant cell granuloma in an Australian tertiary paediatric centre. Bone. 2022 Jun;159.

2. Vanderniet JA, Szymczuk V, Högler W, Beck-Nielsen SS, Uday S, Merchant N, Crane JL, Ward LM, Boyce AM, Munns CF. Management of RANKL-mediated disorders with denosumab in children and adolescents: a global expert guidance document. J Clin Endocrinol Metab. 2023 Dec 2

### S4.2

Details to follow

### Keynote Lecture 2 – Monday

Details to follow

### S5.1

### Impact of nutrition on bone health in children from low- and middle-income countries

Dr Anuradha Khadilkar (Hirabai Cowasji Jehangir Medical Research Institute, Pune, India)

A large amount of bone is accrued during childhood and adolescence. Among the modifiable factors that impact bone health, nutrition is one of the key factors. Dietary intakes of calcium, protein and vitamin D are often suboptimal in children from low and middle income (LMIC) countries. Further, intake of dairy products is limited and the phytates and oxalates in food reduce the bioavailability of calcium. Vitamin D deficiency is also very common in most countries due to social customs of limiting exposure of skin to sunlight. Since dietary calcium intake also influences the relationship between vitamin D and parathyroid hormone (PTH) and with habitually low calcium diets, PTH resistance is observed, bone health is likely to be further compromised in these populations.

Many countries from LMIC's are in nutrition transition and a sedentary lifestyle likely results in lower muscle function and overweight/ obesity; the double burden of malnutrition is thus likely to further impact bone health. With improving medical care, number of children with chronic disease states in LMIC are on the rise. Optimizing and assessing bone health in chronic disease states is critical, even more so in resource limited settings.

Given the hurdles of resource limitations, poor access to bone densitometers and personnel with expertise to interpret children's bone density scans, strategies for optimising bone health in children and adolescents from LMIC's are the need of the hour. Improving calcium intake through non-dairy foods, targeted interventions during periods such as adolescence and supplementation/ fortification with vitamin D are critical.

### S5.2

Details to follow

### S6.1

### Dental aspects of rare bone disorders

Catherine Chaussain (Bretonneau Hospital, Paris, France)

Bones and teeth are the most important mineralized tissues in the body. They share several mechanisms related to their development and mineralization, including the signaling pathways that regulate the differentiation of their cell types 1, 2. Therefore, dental complications are a common morbidity in patients with a rare bone disorders. Depending on the disorder, either the tooth structure, its morphology, or the number of teeth may be affected. In addition, some malocclusions and craniofacial abnormalities such as craniosynostosis may also be present. Rare bone disorders related to the mineral homeostasis or affecting genes encoding a component of the extracellular matrix can dramatically affect tooth mineralization. As a consequence, patients may endure early tooth loss, or high susceptibility to caries, periodontitis or pulp infections1, 2,3. The aim of this presentation will be to highlight the dental aspects of rare bone diseases and the impact of their complications on the patient's quality of life. In addition, we will show how a thorough dental examination can support the diagnosis of a rare bone disorder, and how ultrastructural examination of the tooth can provide new insights into the pathophysiological mechanisms associated with the disorder. In addition, we will discuss how the ultrastructural examination of the tooth can reveal how a systemic treatment improves the mineralization process. Overall, this presentation will urge the need to include a dentist as part of the multidisciplinary team caring for the patient.

1.Kovacs CS, Chaussain C, Osdoby P, Brandi ML, Clarke B, Thakker RV. The role of biomineralization in disorders of skeletal development and tooth formation. Nat Rev Endocrinol. 2021 Jun;17(6):336-349.

2.Opsahl Vital S, Gaucher C, Bardet C, Rowe PS, George A, Linglart A, Chaussain C. Tooth dentin defects reflect genetic disorders affecting bone mineralization. Bone. 2012 Apr;50(4):989-97.

3.McKee MD, Hoac B, Addison WN, Barros NM, Millán JL, Chaussain C. Extracellular matrix mineralization in periodontal tissues: Noncollagenous matrix proteins, enzymes, and relationship to hypophosphatasia and X-linked hypophosphatemia. Periodontol 2000. 2013 Oct;63(1):102-22.

### S6.2

### Unmet needs in the management of hypophosphatasia

Keiichi Ozono (Iseikai International General Hospital, Osaka, Japan)

Hypophosphatasia (HPP) is a genetic bone disease associated with skeletal and extra-skeletal manifestations. The causal gene is ALPL, which encodes tissue-non-specific alkaline phosphatase (TNSALP). Genetic diagnosis is important, and the ALPL gene variant database, which contains more than 400 pathogenic variants, is available. Several clinical guidelines are reported based on the data collected globally. The Japanese Society of Pediatric Endocrinology also established the clinical guidelines for 21 clinical questions. One of the features of HPP in Japan is a relatively large number of patients with severe HPP. It is mainly due to the c.1559delT variant, which is almost exclusive to Japanese people, and the allele frequency is 0.0014. The therapy with asfotase alfa is life-saving, but surviving patients still suffer from complications such as short stature, muscle weakness and slow achievement of developmental milestones. The limited awareness and non-specific signs and symptoms delay the diagnosis and treatment of HPP. Premature loss of baby teeth is rather HPP-specific, but the prompt diagnosis requires collaboration between pediatricians and dentists. The accumulation of pyrophosphate due to low ALP activity is responsible for the hypomineralization. However, pyrophosphate sometimes binds to calcium and causes calcium pyrophosphate deposition disease. The pathogenesis of premature loss of teeth remains unclear. We are working to elucidate the pathophysiology of abnormal dentin using the differentiation system from iPS cells derived from HPP patients into odontoblasts. In this talk, I will explain the unmet needs in the management of HPP by presenting our patients and experiments.

### S6.3

### The changing landscape and ethical challenges in new treatments

Nick Bishop (University of Sheffield and Sheffield Children's Hospital, Sheffield, UK)

In recent years, several new treatments for rare bone diseases have been brought forward, primarily by pharma companies and the biotechnology industry. Targeted therapies bring the promise of significant improvement in outcome in diseases which previously had significant morbidity and mortality. In assessing such interventions, despite the rarity of some conditions, regulators continue largely to require traditional randomised, placebo-controlled, or head to head studies, despite recommendations to adopt adaptive trial designs in such settings. This means that studies take longer and the delivery of effective treatments to patients is slowed.

The very high cost of some treatments means that they are not available in all parts of the world, and even in rich nations access may be restricted because of concerns about cost in relation to efficacy. The long-term outcomes for these new interventions remains the subject of continued scrutiny, given both the high cost, and the long-term nature of the conditions being treated. One improvement that has been increasingly evident is the stronger input of patients to study designs and the reported core outcome sets used to asses efficacy.

Repurposing of existing drugs is seen by many as an important alternative and a major opportunity, although the mechanisms to secure long-term funding support for legal entities to take on the responsibility of developing and maintaining a new licence indication for an “old” drug remain elusive at present.

### S7.1

Details to follow

### S7.2

### Orthopaedic correction of skeletal deformities in Osteogenesis Imperfecta

Nicolas Nicolaou (Sheffield Children's Hospital, Sheffield, UK)

Management of significant skeletal deformity in Osteogenesis Imperfecta remains a technical challenge for both the child and multi-disciplinary team. We aim to reduce fracture rates, promote independence and improve quality of life. Some aspects of surgical treatment have improved, but a number of clinical problems still remain. We will discuss the contemporary management of deformity, technical challenges and strategies to improve outcomes for these cases in children. We will use clinical cases to illustrate some of the difficulties in surgical care and the solutions available.

### S7.3

Details to follow

### S8.1

### This is how I do It: Pharmacological management of pediatric osteoporosis

Leanne Ward (Children's Hospital of Eastern Ontario, Ottawa, Canada)

The management of children at risk for bone fragility and with established osteoporosis are among the most frequent reasons for referral to pediatric bone health clinics. In the last two decades, advances in our understanding of the pathogenic basis of congenital bone fragility has refined the diagnosis of monogenic bone fragility and led to the study of novel therapeutic targets in the prototypical primary osteoporosis condition of childhood, osteogenesis imperfecta. Similarly, elucidation of the natural history of bone fragility in those with underlying chronic illnesses has chiseled knowledge about fracture risk factors and enabled the identification of best candidates and optimal timing for bone-targeted treatment. Overall, the diagnosis and management of pediatric osteoporosis is predicated upon early, rather than late, identification of those at risk for bone fragility, and intervention with bone-targeted therapy in those with limited potential for spontaneous recovery. While there has been considerable progress in this area, numerous unmet needs remain, the most urgent of which is to move beyond the monotherapeutic anti-resorptive landscape to the study and implementation of agents that will foster the pediatric skeleton's ability to optimally adapt to the mechanical loads that normally catalyze bone strength. This presentation focuses on the evidence and practices that shape the current approach to the management of bone fragility in childhood, with emphasis on the key biological principles unique to the growing patient that provide a blueprint for the diagnosis and treatment of childhood osteoporosis in any clinical context.

### S8.2

Details to follow

### Meet The Expert 5

### Defects in vitamin D metabolism – from deficiency to resistance

Wolfgang Högler (Johannes Kepler University, Linz, Austria)

Bone mineralization is a complex process that requires substrate (calcium, phosphorus), substrate supplier (calcitriol) and functioning bone cells. The hormone calcitriol is made from the inactive vitamin D from dermal production or some food sources, and its main function is to increase intestinal bone mineral absorption. Calcitriol can not work when there is no oral calcium supply, hence rickets develops in conditions of dietary calcium deficiency or malabsorption as much as it develops in vitamin deficient states. These deficiencies a very common, easy to diagnose due to a typical diagnostic biochemical signature (high ALP, high PTH), and a major public health concern. Much in contrast, some rare heritable defects in calcitriol synthesis or action can present with the same biochemical signature but unresponsiveness to vitamin D. These vitamin D dependent forms of rickets (VDDR) are enzyme defects in the calcitriol synthesis pathway (25-Hydroxylase [CYP2R1], 1-Hydroxylase [CYP27B1]), defects in the calcitriol receptor gene (VDR), abnormal expression of the Vitamin D response element (gene unknown) and enhanced enzymatic inactivation of all vitamin D metabolites (CYP3A4). All metabolic defects need specific diagnostic and therapeutic considerations. Recent advances in the understanding of these disease mechanisms have brought about therapeutic progress for hypercalcaemic conditions using enzyme inducers and inhibitors.

### Oral Communication Presentation Abstracts

### OC 1.1

### Complex defects in osteoblast lineage cells underlie the pathogenesis of X-Linked hypophosphatemia: Analysis using PHEX-Knockout human iPS cells

Toshimi Michigami[1], Tatsuro Nakanishi[1,2], Miwa Yamazaki[1], Kanako Tachikawa[1], Ayu Ueta[1,3], Masanobu Kawai[1], Keiichi Ozono[4]

[1]Department of Bone and Mineral Research, Research Institute, Osaka Women's and Children's Hospital, Izumi, Japan. [2]Department of Pediatrics, Osaka University Graduate School of Medicine, Suita, Japan. [3]The 1st Department of Oral and Maxillofacial Surgery, Osaka University Graduate School of Dentistry, Suita, Japan. [4]Professor Emeritus, Osaka University Graduate School of Medicine, Suita, Japan

### Abstract

**OBJECTIVES:**

X-linked hypophosphatemia (XLH) is caused by inactivating variants of *PHEX*, the gene highly expressed in mature osteoblasts and osteocytes. Overproduction of FGF23 leads to hypophosphatemia and impaired vitamin D metabolism in XLH; however, the pathogenesis of the disease still remains unclear. To investigate the intrinsic defects in the osteoblast lineage cells in XLH, we herein analyzed the osteoblast lineage cells differentiated from the *PHEX*-knockout (KO) human induced pluripotent stem (iPS) cells.

**METHODS:**

We generated *PHEX*-KO human iPS cells by applying CRISPR/Cas9-mediated gene ablation to an iPS clone derived from a healthy male. The differentiation of the *PHEX*-KO and isogenic control iPS cells into osteoblast lineage was induced by culture in osteogenic medium containing b-glycerophosphate, dexamethasone, ascorbic acid and all-trans retinoic acid.

**RESULTS:**

The expression of *PHEX* increased by time in the isogenic control iPS cells, while *PHEX*-KO cells completely lacked the functional *PHEX* transcripts. We confirmed the increased production of FGF23 in osteoblast lineage cells differentiated from *PHEX*-KO iPS cells. Interestingly, *in vitro* mineralization was enhanced in osteoblast lineage cells from *PHEX*-KO iPS cells compared to those from isogenic control iPS cells, which reminded us of high bone mineral density and enthesopathy in XLH patients. Extracellular pyrophosphate, an inhibitor of mineralization, was increased in osteoblast lineage cells from *PHEX*-KO iPS cells, which seemed to be partly due to the reduced activity of tissue non-specific alkaline phosphatase. Osteoblast lineage cells from *PHEX*-KO iPS cells also showed the increased expression of multiple molecules including DMP1, Osteopontin, RUNX2, FGF receptor 1 and early growth response 1, and this was similar to the gene dysregulation observed in the osteoblasts/osteocytes of *Phex*-deficient *Hyp* mice. Of note, the phosphorylation of CREB was markedly enhanced in osteoblast lineage cells from *PHEX*-KO iPS cells, which appeared to be associated with up-regulation of the *PTHRP* gene.

**CONCLUSIONS:**

In addition to the overproduction of FGF23, complex intrinsic defects may underlie the pathogenesis of XLH. Enhanced CREB pathway in *PHEX* deficiency might increase the expression of *RUNX2*, and this might affect the expression of various genes such as *DMP1* and *Osteopontin* and capacity to be mineralized.

### Disclosure

TM received consultation fees from Kyowa-Kirin Co. Ltd. and lecture fees from Kyowa-Kirin Co. Ltd., and Alexion Pharma. KO received consultation fees from Kyowa-Kirin Co. Ltd. And lecture fees from Kyowa-Kirin Co. Ltd., Alexion Pharma and Pfizer.

### OC 1.2

### An RNA-First approach identifies deep intronic PHEX variants in children with X-Linked hypophosphatemic rickets

Karissa Ludwig[1,2,3,4], Zenghui Wu[1], Ghalib Bardai[1], Patrizia Mason[1], Juliana Marulanda[1], Nathalie Alos[5], Leanne M Ward[6,7], Frank Rauch[1,8]

[1]Shriners Hospitals for Children, Canada, Montreal, Canada. [2]Queensland Children's Hospital, Brisbane, Australia. [3]West Moreton Health, Ipswich, Australia. [4]University of Queensland, Brisbane, Australia. [5]CHU Ste-Justine, Montreal, Canada. [6]Division of Endocrinology, Children's Hospital of Eastern Ontario, Ottawa, Canada. [7]Department of Pediatrics, University of Ottawa, Ottawa, Canada. [8]McGill University, Montreal, Canada

### Abstract

### Objectives:

To identify intronic variants causing mis-splicing of *PHEX* in patients with clinically-diagnosed X-linked hypophosphatemic rickets (XLH) in whom no variant was identified on clinical molecular diagnostic testing.

### Methods:

Urine samples were obtained from 5 patients (5-12 years, 3 females) with clinically-diagnosed XLH where no candidate variant was identified on rickets gene panel sequencing of genomic DNA. Urine-derived cells (UDC) were cultured as previously described (1). *PHEX* was expressed at low levels in UDC, though expression was superior to fibroblasts and blood (1). PCR amplification of mRNA was performed, followed by next-generation sequencing, as described (2). Abnormalities were confirmed using Sanger sequencing. In addition, DNA sequencing of *PHEX* intronic regions was performed to identify candidate variants potentially causing mis-splicing seen on RNA analysis.

### Results:

Abnormal mRNA splicing was identified in 3 of 5 patients, and a novel candidate variant was identified on sequencing of *PHEX* introns in each of the 3 patients with splicing defects. Pseudoexons were seen in 2 patients. An 84 bp pseudoexon in *PHEX* intron 21 caused by a deep intronic variant (c.2147+1197A>G) was identified in an 11-year-old girl. This variant was *de novo* (not present in either of the unaffected parents) and corresponded to a variant that we had previously published (2). We subsequently found the same variant in two more families with XLH, suggesting that this variant is a common but hitherto overlooked cause of XLH. In addition, a pseudoexon in intron 17 of *PHEX* secondary to a deep intronic variant (c.1768+173A>G) was detected in another 11-year-old female with XLH. Finally, RNA analysis identified skipping of *PHEX* exon 13 in a 12-year-old boy. An 18 bp deletion in intron 13 was detected on whole gene sequencing, which was not seen in the boy's unaffected mother, suggesting that the deep intronic deletion in this boy was causing the exon skipping and thereby giving rise to XLH.

### Conclusions:

Our RNA-based analysis combined with sequencing of the entire *PHEX* gene is a useful approach for identification of novel candidate variants in patients with XLH who do not have a molecular diagnosis through standard clinical testing.

**References:**

1. J Bone Miner Res. 2023;38(8):1125-34. DOI:10.1002/jbmr.4865

2. Bone. 2023;176:116839. DOI:10.1016/j.bone.2023.116839

### Disclosure

LMW Declares consultancy to Ultragenyx and Kyowa Kirin, and participation in clinical trials with Ultragenyx (with funds to Dr. Ward's institution) No other disclosures

### OC 2.1

### Post-natal evolution of bone histomorphometry and matrix mineralization: A prospective post-mortem study in infants and young children

Suma Uday[1,2], Markus A Hartmann[3], Stephane Blouin[3], Marta Cohen[4], Tamas Marton[1,5], Nadja Fratzl-Zelman[3], Wolfgang Högler[6,2]

[1]Birmingham Women's and Children's Hospital, Birmingham, UK [2]Institute of Metabolism and Systems Research, University of Birmingham, Birmingham, UK [3]Ludwig Boltzmann Institute of Osteology at the Hanusch Hospital of OEGK and AUVA Trauma Center Meidling, 1st Medical Department Hanusch Hospital, Vienna, Austria. [4]Sheffield Children's Hospital, Sheffield, UK [5]Semmelweis University, Budapest, Hungary. [6]Department of Paediatrics and Adolescent Medicine, Johannes Kepler University, Linz, Austria

### Abstract

### Objectives:

Data on postnatal changes in bone structure and mineralization in infants are scarce given the invasive nature of biopsies. Therefore, we assessed histomorphometry (HM) and bone mineralization density distribution (BMDD) in iliac bone samples obtained during a prospective post-mortem study.

### Methods:

Children under 2 years with sudden unexpected death, undergoing routine autopsy, were recruited (between 2017-2022) with parental consent following regulatory approvals. Individuals with conditions or medication affecting bone health were excluded. Bone tissue from 39 subjects (age: 0-660 days, median:75) were prepared for HM and quantitative backscattered electron imaging. Data are presented as median [IQR]. Spearman's correlations were used to assess the variation of HM indices with age. BMDD was compared with published pediatric reference data (n=50, age: 2.0-20.9 years, median 12.95).

### Results:

The median trabecular bone volume (BV/TV) was 18.5% [14.6, 24.2] and decreased with age ($r=-0.57$, $p<0.0001$) due to rapidly decreasing trabecular numbers (Tb.N: 3.06/mm [2.3; 4.2], $r=-0.77$, $P<0.0001$), in the first 3 months of life, rather than a drop in trabecular thickness (Tb.Th: 61.1µm [53.5; 78.4], $p=0.37$). Osteoid volume correlated weakly with age (OV/BV: 3.36% [1.1; 4.8], $r=0.35$, $p=0.034$) but osteoid thickness and surface did not (O.Th: 4.3µm [3.1; 5.3], $p=0.54$; OS/BS: 22.7% [13.2; 34.9], $p=0.29$). The mean calcium content of the matrix (CaMean) was significantly lower compared to older children **(see figure)** (trabecular bone: -5.14%, cortical bone: -11.7%, both $p<0.0001$), caused by twice as much lowly mineralized trabecular bone (CaLow) and thrice as much in cortical bone (both $p<0.0001$). Trabecular CaLow was positively correlated with OS/BS ($r=0.66$) and OV/BV ($r=0.61$), both $p<0.0001$. Elevated CaHigh was noted in trabecular bone, particularly of infants < 45 days, due to substantial amount of residual mineralized cartilage. Cortical CaHigh did not vary with age.

### Conclusion:

The trends in HM parameters and observed differences in BMDD of younger vs older children mirror adaptive metabolic changes to extra-uterine life, notably reduction of dense trabecular network through remodelling activity and bone shaping through modelling processes in the cortex. The results presented here fill a gap in reference data required for the investigation of suspected bone disease in infants and young children.

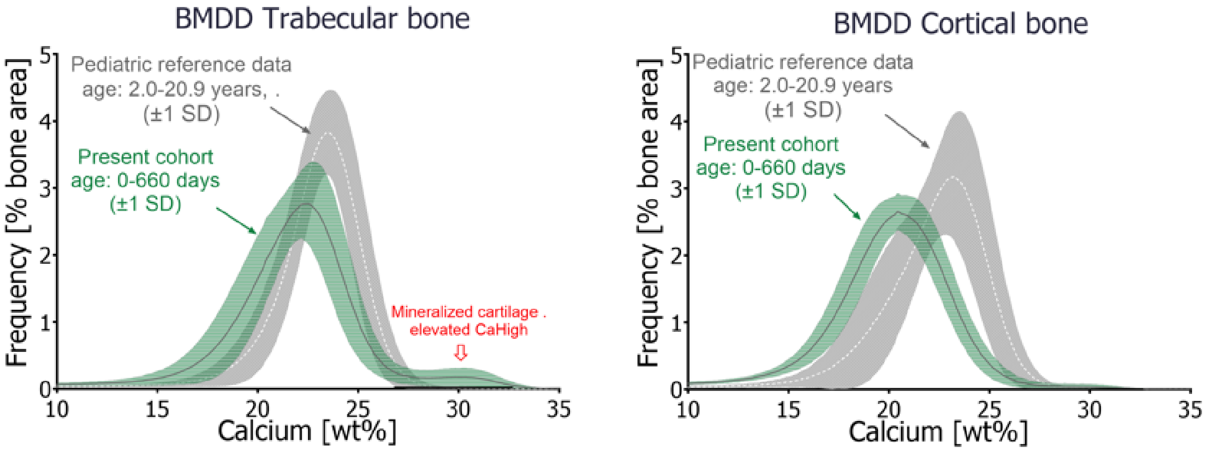

### OC 2.2

### Efficacy of moderate-dose denosumab in fibrous dysplasia: Secondary results from a phase 2 clinical trial

Vivian Szymczuk[1], Ibrahim Elbashir[1], Ramzy Ahmed[2,1], Alison Boyce[1]

[1]National Institute of Dental and Craniofacial Research, National Institutes of Health, Bethesda, MD, USA
[2]Eastern Virginia Medical School, Norfolk, VA, USA

### Abstract

OBJECTIVES: Fibrous dysplasia (FD) is a rare disease in which normal bone and marrow are replaced with expansile fibro-osseous tissue, leading to fractures and disability. FD tissue demonstrates prominent osteoclastogenesis and increased expression of the pro-osteoclastic factor RANKL. A phase 2 clinical trial of the RANKL inhibitor denosumab demonstrated profound reduction in FD lesion activity and increased lesional mineralization after 6 months of high-dose treatment (120mg/month with loading doses on weeks 2 and 3). Denosumab was well-tolerated, although discontinuation was associated with severe hypercalcemia in 1 subject. These results demonstrated that denosumab can provide substantial clinical benefit in FD; however, dose-related side effects remain a concern. We performed a secondary analysis to evaluate the efficacy of a moderate-dose regimen (120 mg/3 months) in comparison to the standard high-dose regimen.

METHODS: Data was analyzed from a phase 2 clinical trial at the NIH (NCT03571191). 8 adult subjects (mean 31y, range 20-55) received high-dose denosumab for 6-months (120mg/month with loading doses on weeks 2 and 3) followed by an 8-month post-treatment observation period. Given the observed substantial clinical benefits, the protocol was amended to restart a moderate-dose regimen (120 mg/3 months) after the post-treatment observation period if clinically indicated. Serial serum bone turnover markers and radiographic $^{18}$F-NaF PET/CT scans were evaluated at the start of moderate-dose therapy and after 6-months in 6 subjects.

RESULTS: Changes in bone turnover markers at initial and final dose were comparable on high vs moderate-dose denosumab (P1NP -82% and -92%, and CTX -86% and -82% for moderate and high-dose, respectively). There was no difference in $^{18}$F-NaF PET/CT lesional activity or absolute change in lesion volume between the moderate and high dose regimens. Bone turnover markers on moderate-dose treatment showed sustained decline in 4 subjects, however 2 subjects with extensive FD experienced bone turnover rebound between the 3-month doses.

CONCLUSION: These findings demonstrate that moderate-dose denosumab may provide clinical benefits comparable to the high-dose regimen in adults with FD, while potentially lowering associated risks. However, patients with extensive FD and high baseline bone turnover may require more frequent dosing, indicating a need to individualize treatment regimens.

### Disclosure

This investigator sponsored study was supported by Amgen, Inc; and received approval from the NIH Investigational Review Board (NCT03571191). Study design, conduct and analyses were performed completely and independently by investigators.

### OC 2.3

### Effects of RANKL inhibition on osteotomy healing in a murine model of fibrous dysplasia

Aleena Arif[1], Kristen Pan[1,2], Luis F de Castro[3], Vardit Kram[1], Alison Boyce[1]

[1]Metabolic Bone Disorders Unit, National Institute of Dental and Craniofacial Research, National Institutes of Health, Bethesda, USA [2]Department of Plastic and Reconstructive Surgery, The Johns Hopkins Medical Institutions, Baltimore, USA [3]Skeletal Disorders and Mineral Homeostasis Section, National Institute of Dental and Craniofacial Research, National Institutes of Health, Bethesda, USA

### Abstract

Fibrous dysplasia (FD) is a rare mosaic disorder resulting in expansile fibro-osseous lesions leading to deformities, fractures, and functional impairment. Surgery to prevent and repair deformities and fractures is a mainstay of treatment. RANKL inhibition with denosumab is a promising therapy to reduce lesional activity and increase mineralization; however, the effects of denosumab on FD tissue healing are unknown.

To investigate RANKL inhibition effects on osteotomy healing, we compared cortical and trabecular bone repair in FD and WT mice with and without anti-RANKL treatment. Our injury model consisted of a 0.9mm diameter unicortical drill hole between the proximal epiphysis and mid-diaphysis of the tibia, including the periosteal and endosteal surfaces of one cortex and extended 0.3 to 0.5mm into the marrow. Bone microarchitecture analysis was performed using Analyze 14.0 software on micro-CT scans. Volumes of interest (VOI) for the cortical and medullary defects were manually traced.

The results demonstrate a significant increase in Bone Volume/Total Volume (BV/TV) and Bone Mineral Density (BMD) in both FD and WT mice treated with anti-RANKL. In the cortical defect, the mean BV/TV increased from 34.3% to 58.7% in WT mice and 34% to 43% in FD mice post treatment. In the medullary defect, the mean BV/TV increased from 15.6% to 27.4% in WT mice and 22.9% to 39.5% in FD mice, and mean BMD increased from 622.7 to 701.7 in WT mice and 583.7 to 694.4 in FD mice post treatment.

These findings indicate that anti-RANKL treatment does not impair bone healing but increases density of healing tissue. These pre-clinical data suggest that denosumab treatment in patients with fibrous dysplasia likely does not impair healing from osteotomy procedures and may serve as a useful adjuvant to surgical management.

### Disclosure

NIDCR receives funding from Amgen, Kyowa Kirin, and Ultragenyx for studies related to fibrous dysplasia.

### OC 3.1

### Increasing effect of pregnancy vitamin D supplementation on offspring bone mineral density during early childhood: Findings from the MAVIDOS randomised controlled trial

Rebecca J Moon[1,2], Stefania D'Angelo[1], Elizabeth M Curtis[1], Kate A Ward[1], Sarah R Crozier[1,3], Keith M Godfrey[1,4], Cyrus Cooper[1,4], Nicholas C Harvey[1,4]

[1]MRC Lifecourse Epidemiology Centre, University of Southampton, Southampton, UK [2]Paediatric Endocrinology, Southampton Children's Hospital, University Hospital Southampton NHS Foundation Trust, Southampton, UK [3]NIHR Applied Research Collaboration Wessex, Southampton, UK [4]NIHR Southampton Biomedical Research Centre, University of Southampton and University Hospital Southampton NHS Foundation Trust, Southampton, UK

### Abstract

**OBJECTIVES:** In the MAVIDOS trial, a positive effect of pregnancy cholecalciferol supplementation on offspring bone mineral density (BMD) at age 4 years was demonstrated. We sought to establish persistence of this at 6-7 years.

**METHODS:** MAVIDOS was a randomized double-blind placebo-controlled trial of 1000 IU/day vitamin D supplementation in pregnancy from 14-17 weeks' gestation until delivery. Dual-energy X-ray absorptiometry (DXA, Hologic Discovery) was performed on the offspring at birth (whole body), 4 years (whole-body-less-head [WBLH]) and 6-7 years (WBLH) to assess bone area (BA), bone mineral content (BMC), BMD and bone mineral apparent density (BMAD). DXA outcomes were transformed to a standard deviation scale. The effect of cholecalciferol supplementation was assessed using linear regression, including age and sex in the models.

**RESULTS:** 447 (216 placebo; 231 cholecalciferol) children had a usable DXA scan at 6-7 years. WBLH BMD was greater in the cholecalciferol group (β=0.16 SD, 95%CI -0.01,0.34) with a similar finding for BMAD (β=0.16 SD, 95%CI -0.01,0.34). WBLH BA and BMC did not differ between the groups.

378 children had WBLH DXA at both 4 and 6-7 years. Between ages 4 and 6-7 years, pregnancy cholecalciferol effect magnitudes tended to increase for each of WBLH BMC (4years: β=0·14 SD, 95%CI -0·06,0·33; 6-7years: β=0·21 SD, 95%CI 0·03,0·40), BMD (4years: β=0·21 SD, 95%CI 0·01,0·40; 6-7years: β=0·25 SD, 95%CI 0·06,0·43) and BMAD (4years: β=0·20 SD, 95%CI 0·01,0·39; 6-7years β=0·24 SD, 95%CI 0·05,0·42). Among the 318 children who had DXA at birth, 4 and 6-7 years, no effect of cholecalciferol on DXA outcomes was evident at birth, but a positive effect on BMC, BMD and BMAD that increased in magnitude from age 4 to 6-7 years was similarly observed.

**CONCLUSIONS:** Supplementation with 1000 IU/day cholecalciferol from 14-17 weeks' pregnancy until delivery increases offspring WBLH BMD at age 6-7 years, with a somewhat greater effect size than observed at 4 years of age. Further work to understand the mechanisms underpinning this evolving effect and to demonstrate persistence into adulthood will be important to support recommendations regarding higher dose vitamin D supplementation in pregnancy.

### OC 3.2

### Gastrointestinal phenotype in a mouse model of moderate-severe osteogenesis imperfecta

Yuan Zhang[1,2], Michèle Iskandar[1], Stan Kubow[1], Svetlana V Komarova[2,3,4], Josephine T Tauer[2,3,5]

[1]School of Human Nutrition, McGill University, Montreal, Canada. [2]Shriners Hospital for Children, Montreal, Canada. [3]Faculty of Dental Medicine and Oral Health Sciences, McGill University, Montreal, Canada. [4]Biomedical Engineering, University of Alberta, Edmonton, Canada. [5]Department of Pediatrics and Adolescent Medicine, Johannes Kepler University Linz, Linz, Austria

### Abstract

**OBJECTIVE:** Osteogenesis imperfecta (OI), an inherited disorder due to collagen-I genetic mutations, notably impacts skeletal structure. Although collagen-I is a crucial component of the gastrointestinal tract's extracellular matrix, the potential consequences on the 'OI intestine' remain unclear. With OI involving metabolic alterations and gastrointestinal factors contributing to OI mortality, this study aimed to evaluate the intestinal morphology, function, and gut microbiota in OI mice.

**METHODS:** Intestinal anatomy was assessed in male and female regular-chow-fed *Col1a1*$^{Jrt/+}$ mice and compared to their sex-matched wild-type (WT) counterparts at ages 4-, 8-, and 12 weeks. The cecal microbial profile (16s rRNA) and activity (short-chain and branched-chain fatty acid profile; SCFA, BCFA) were examined at four weeks of age.

**RESULTS:** Overall, OI mice exhibited consistently more fragile GITs at all ages and greater relative intestinal length, particularly evident in females. Histological examination revealed structural adaptations in OI mice, characterized by a higher villus-to-crypt ratio throughout the small intestine. Quantifying stained total intestinal non- and collagenous proteins indicated lower overall collagen levels in OI, primarily in the duodenum of male OI mice. Additionally, decreased duodenal *CYP24A1* expression in 4-week-old OI mice was found, suggesting altered calcitriol-mediated calcium uptake. At 12 weeks, intestinal permeability testing showed a trend of diminished penetrability in OI, implying a possible altered gut epithelial integrity. Despite similarities in dominant genera in microbial profiles between genotypes, discriminant analysis identified three significantly less abundant genera in OI: Lactobacillus, Lachnospiraceae_FCS020, and Desulfovibrio. Notably, the initial two are primary saccharide fermenters, and microbial activity analysis verified reduced saccharolytic fermentation activities, especially in female OI mice. Specifically, decreased SCFA production, linked to saccharolytic fermentation, was found in female OI mice. This decrease coincided with a reduction in BCFA production.

**CONCLUSIONS:** We demonstrate that OI's underlying collagen mutation significantly affects intestinal structure, function, and microbial fermentation, potentially influencing OI's metabolism. Striking was the altered microbial saccharolytic activity, a crucial nutrient fermentation process for energy production and known to influence the absorptive capacity. Further research is essential for understanding age-specific nutritional needs in OI patients, considering observed intestinal alterations and age-dependent metabolic changes.

### OC 3.3

### Exploring the molecular mechanism of type XV osteogenesis imperfecta caused by WNT1 mutation

Kai Tsun To[1,2], Zhijia Tan[2], Hiutung Shek[2], Yukun Zhang[1]

[1]The University of Hong Kong, Hong Kong. [2]The University of Hong Kong–Shenzhen Hospital, ShenZhen, China

### Abstract

OBJECTIVES:

The primary objective of this study is to investigate the pathogenic mechanisms of type XV osteogenesis imperfecta (OI) caused by WNT1 mutations in patients from southern China and to explore the potential treatment strategies for common low bone mass diseases, such as early onset osteoporosis.

METHODS:

A pilot study was conducted on 243 OI patients from southern China to determine the proportion of type XV patients and the phenotypic spectrum. Functional assays were performed to assess the impact of WNT1 mutations on its secretion and effective activity. Proteomic data analysis was carried out to examine the expression of SOST, while single-cell transcriptome data was used to evaluate the maturation of osteoblasts and osteocytes. Loss-of-function (Wnt1flox) and gain-of-function (Rosa26Wnt1) mouse models were employed to further explore the functions of Wnt1 during skeletal development.

RESULTS:

Approximately 10% of the OI patients in our pilot study were diagnosed with type XV, presenting a diverse phenotypic spectrum. Functional assays demonstrated that WNT1 mutations significantly impaired its secretion and effective activity. Proteomic data analysis revealed a dramatic reduction in SOST expression, and single-cell transcriptome data indicated impaired maturation of osteoblasts and osteocytes.

CONCLUSIONS:

Our findings suggest that WNT1 mutations in type XV OI patients lead to impaired secretion and effective activity, reduced SOST expression, and impaired maturation of osteoblasts and osteocytes. The use of genetic mouse models combined with multi-omics data will help to elucidate the downstream signaling pathways activated by WNT1 and its role in the differentiation and maturation of skeletal stem cells. This research will provide insights into the pathogenic mechanisms of type XV OI and contribute to the development of new treatment strategies for common low bone mass diseases, such as early-onset osteoporosis

### OC 3.4

### Retrospective cohort analysis of childhood autoimmune osteoporosis diagnosed by raised anti-osteoprotegerin antibody

Reena Perchard[1], David B N Lim[2], Toby Candler[3], Christine Burren[3], Justin H Davies[2], M Zulf Mughal[1], Raja Padidela[1], Amish Chinoy[1]

[1]Manchester University NHS Foundation Trust, Manchester, UK [2]University Hospital Southampton NHS Foundation Trust, Southampton, UK [3]University Hospitals Bristol NHS Foundation Trust, Bristol, UK

### Abstract

### Introduction

Osteoprotegerin (OPG) protects the skeleton from excessive bone resorption by binding to RANKL and preventing interaction with RANK. Therefore, regulation of the ratio between RANKL and OPG is crucial in maintaining balanced bone resorption and formation. In contrast to a 1% prevalence amongst healthy individuals, neutralising antibodies to the OPG protein have been detected in 10% of patients with coeliac disease, rheumatoid arthritis and spondyloarthritis, all autoimmune conditions where there is increased risk of osteoporosis.

### Objective

To retrospectively analyse a cohort of children with osteoporosis and elevated anti-OPG antibodies, to review features that may raise clinical suspicion of autoimmune osteoporosis.

### Methods

Clinical and biochemical data were collected on all patients with osteoporosis and elevated anti-OPG antibodies, under tertiary Paediatric Metabolic Bone Disease services across three sites.

### Results

Eight patients were included (all male, age range 10-14 years at presentation). Comorbidities included type 1 diabetes, autoimmune hepatitis, inflammatory bowel disease, coeliac disease as well as autoimmune hypothyroidism and uveitis in a patient with lipodystrophy. Genetic panel tests were negative. Median OPG antibody titre was 329 ng/ml (range 65 to 525; normal < 33) and all exhibited raised bone turnover markers (P1NP + CTX / beta-CTX). Up to 15 vertebral fractures were demonstrated, representing the most severe disease phenotype. In all cases, intravenous bisphosphonate treatment demonstrated improvement in bone mineral density z-scores and successfully reversed debilitating back pain. Further data relating to bone density are provided in Table 1.

### Conclusion

Anti-OPG antibody testing should be considered in children with osteoporosis, particularly those with additional autoimmune/inflammatory conditions, as it may identify a unique form of autoimmune osteoporosis responsive to bisphosphonate therapy. Further research is needed to confirm these findings and explore the role of genetic factors involved in this form of osteoporosis.

**Table 1. Characteristics of eight patients with elevated anti-osteoprotegerin antibodies**

| | Case 1 | Case 2 | Case 3 | Case 4 | Case 5 | Case 6 | Case 7 | Case 8 |
|---|---|---|---|---|---|---|---|---|
| **Age at presentation (years)** | 14 | 12 | 10 | 12 | 14 | 13 | 14 | 11 |
| **Sex** | M | M | M | M | M | M | M | M |
| **OPG Ab titre ng/ml (Normal 33 ng/ml, Positive>96 ng/ml)** | 119 | 354 | 303 | 262 | 402 | 362 | 525 | 65 |
| **No. of vertebral fractures** | 14 | 9 | 15 | 9 | 10 | 7 | 9 | 13 |
| **Baseline DEXA Lumbar spine (L1-4) BMAD SDS** | -4.1 | -5.0 | -3.9 | -3.1 | - 3.1 | -2.0 | -2.1 | -4.2 |
| **Duration of treatment before follow up DEXA (years)** | 1 | 1 | 1.5 | 1 | 1 | 1 | 1 | 1 |
| **Follow up DEXA Lumbar spine (L1-4) BMAD SDS** | -2.3 | -2.3 | -0.9 | Report awaited | -1.5 | 0.5 | -0.4 | -0.5 |

### OC 3.5

### Expanding the spectrum of radiographic features in children with Ectonucleotide Pyrophosphatase/Phosphodiesterase 1 (ENPP1) deficiency: Novel skeletal findings from an international collaboration

Leanne Ward[1,2], Deborah Wenkert[3], Carlos R Ferreira[4], Tom D Thacher[5], Kevin Smit[1,2], Mei Wang[6], Jacob L Jaremko[7], Nagwa Wilson[1,2], Khaldoun Koujok[1,2], Alix Besancon[8], Klaus Dieterich[9], Frank Rutsch[10], M Zulf Mughal[11], Suma Uday[12,13], David R Weber[14,15], Amish Chinoy[16], Joel Steelman[17], Christina M Jacobsen[18], Nathalie Alos[19], Stefan Jackowski[6], Maya Scharke[6], Nasrin Khan[6], Alex Lai[20], David Thompson[20], Ramy El-Jalbout[19]

[1]University of Ottawa, Ottawa, Canada. [2]Children's Hospital of Eastern Ontario, Ottawa, Canada. [3]Wenkert & Young, LLC, Thousand Oaks, USA [4]National Human Genome Research Institute, Bethesda, USA [5]Mayo Clinic, Rochester, USA [6]Children's Hospital of Eastern Ontario Research Institute, Ottawa, Canada. [7]University of Alberta, Edmonton, Canada. [8]Necker-Enfants Malades University Hospital, Assistance Publique-Hôpitaux de Paris, Paris, France. [9]University Grenoble Alpes, Grenoble, France. [10]Muenster University Children's Hospital, Muenster, Germany. [11]Al Jalila Children's Speciality Hospital, Dubai, UAE. [12]University of Birmingham, Birmingham, UK [13]Women's and Children's Hospital, Birmingham, UK [14]The Children's Hospital of Philadelphia, Philadelphia, USA [15]The University of Pennsylvania, Philadelphia, USA [16]Royal Manchester Children's Hospital, Manchester, UK [17]Cook Children's Medical Center, Fort Worth, USA [18]Boston Children's Hospital, Boston, USA [19]Centre Hospitalier Universitaire Sainte-Justine Centre de Recherche, Montréal, Canada. [20]Inozyme Pharma, Boston, USA

### Abstract

**Background:** ENPP1 Deficiency is a rare, genetic disorder characterized by extra-skeletal calcification, neointimal proliferation, and impaired growth plate and skeletal mineralization. The phenotype has a broad range of features including generalized arterial calcification of infancy (GACI) and autosomal recessive hypophosphatemic rickets type 2 (ARHR2). Adult manifestations include osteomalacia, enthesopathy and joint pain.

**Objectives:** To describe the radiographic features of pediatric ENPP1 Deficiency using data from international observational studies.

**Methods:** Radiographs from children with bi-allelic, loss of function variants in ENPP1 were collated through a central imaging portal. Patients were included if they were ≤ 13 years of age at the time of the first x-ray. A team of three clinicians with expertise in rare pediatric bone disorders (including one pediatric radiologist) evaluated the radiographs. Data were collated and sorted by frequency, thematic categorization and skeletal site.

**Results:** Twenty-two patients with 366 radiographs were assessed (9 males, 40.9%); average age at baseline 4.77 ± 3.95 years. 13/22 patients (59.0%) presented with GACI and later ARHR2, 9/22 patients (40.9%) presented for the first time with AHRH2. Radiographic features were categorized into seven different themes: 1. Rickets, 2. Fractures, 3. Abnormal bone density (osteopenia + osteosclerosis), 4. Inflammatory signs, 5.

Osteoarthritis-type changes, 6. Skeletal dysplasia features, and 7. Calcifications (peri-articular + vascular). A total of 52 discrete radiological features were observed; of these, rickets was most frequent (present in all children). Almost 2/3 of children had lower limb deformity and/or long bone metadiaphyseal expansion; in addition, reduced long bone cross-sectional diameter was present in almost 60% and half of the children had negative ulnar variance. Interestingly, inflammatory-type changes, including joint space narrowing, carpal crowding, asymmetric wrist bone age, epiphyseomegaly, erosive signs, peri-articular osteopenia and femoral-acetabular impingement, were also observed.

**Conclusions:** This radiographic review provides an unprecedented description of skeletal features in pediatric ENPP1 Deficiency and in so doing, highlights the substantial disease burden and complex pathobiology of the condition. To our knowledge, inflammatory-type changes have not previously been described in children with ENPP1 deficiency and may have implications for diagnosis, treatment and the development of meaningful endpoints in future clinical trials.

### Disclosure

LMW has been a consultant to, and participated in clinical trials with, Inozyme Pharma, Amgen, Ascendis, Novartis, PTC Therapeutics, ReveraGen, Catabasis, Ipsen and Ultragenyx, with funds to Dr. Ward's institution; DW was previously employed by (and has previously consulted for) Inozyme Pharma and reports having stock options; CRF has collaborated with Inozyme Pharma as part of a Cooperative Research and Development Agreement (CRADA); TDT has consulted for CHEO, Ultragenyx, and Kyowa Kirin; JLJ was funded by CIFAR, MIC, AMII, and Alberta Innovates; MZM has been the principal investigator for three Inozyme studies & trials, consults for and being speaker for Inozyme Pharma; DRW received funding from Inozyme Pharma, consults for Inozyme and Merck, has been the speaker for Inozyme and PTC Therapeutics; NA has been the principal investigator for the ENERGY 3 Study (Inozyme Pharma); AL is the vice president of Inozyme Pharma; DT is an employee and stockholder of Inozyme Pharma. MW, NW, KK, AB, KD, FR, SU, AC, JS, CMJ, SJ, MS, and REIJ report no conflicts of interest.

### OC 3.6

### First results of the Global ALPL gene variant classification project

Mariam Farman[1], Catherine Rehder[2], Theodora Malli[3], Cheryl Rockman-Greenberg[4], Kathryn Dahir[5], Gabriel Ángel Martos-Moreno[6,7], Agnès Linglart[8], Keiichi Ozono[9], Lothar Seefried[10], Guillermo Del Angel[11], Florian Högler[1], Francesca Barbazza[1], Jessica Ebner-Jahn[1], Erica Burner Nading[2], Erin Huggins[2], Eric T. Rush[12,13,14], Priya S Kishnani[2], Gerald Webersinke[3], Wolfgang Högler[1]

[1]Department of Paediatrics and Adolescent Medicine, Johannes Kepler University Linz, Linz, Austria. [2]Duke University Medical Center, Division of Medical Genetics, Dept of Pediatrics, Durham, USA [3]Laboratory for Molecular Genetic Diagnostics, Ordensklinikum Linz, Linz, Austria. [4]Department of Pediatrics and Child Health, Rady Faculty of Health Sciences, University of Manitoba, Winnipeg, Canada. [5]Vanderbilt University Medical Center, Program for Metabolic Bone Disorders, Nashville, Nashville, TN, USA [6]6 Departments of Pediatrics & Pediatric Endocrinology, Hospital Infantil Universitario Niño Jesús, IIS La Princesa, Madrid, Spain. [7]CIBER Fisiopatología de la Obesidad y Nutrición. ISCIII, Madrid, Spain. [8]AP-HP, Paris Saclay University, INSERM, Bicêtre Paris Saclay hospital, Le Kremlin-Bicêtre, France. [9]Department of Pediatrics, Osaka University Graduate School of Medicine, Osaka, Japan. [10]10Julius-Maximilian University, Wurzburg, Germany. [11]Alexion, AstraZeneca Rare Disease, Boston, Boston, MA, USA [12]Division of Clinical Genetics, Children's Mercy Hospital Kansas City, Kansas city, MO, USA [13]Department of Internal Medicine, University of Kansas School of Medicine, Kansas City, KS, USA, Kansas city, KS, USA [14]; Department of Pediatrics, University of Missouri – Kansas City School of Medicine, Kansas City, MO, USA

### Abstract

### Objectives

Hypophosphatasia (HPP) is a multisystemic genetic disorder that primarily affects bone and teeth development and deficiency of serum and bone alkaline phosphatase activity, caused by pathogenic variants in *ALPL* affecting the tissue non-specific alkaline phosphatase. Diagnosing HPP is challenging due to *ALPL* variants of uncertain significance (VUS), which create ambiguity in patients and treating physicians and result in diagnostic delays. The *ALPL* variant classification project was established to reclassify VUS and to continuously assess and update genetic, phenotypic, and functional variant information in the *ALPL* variant database (https://alplmutationdatabase.jku.at/). Here we report the first results of the *ALPL* variant classification project.

### Methods

An international multidisciplinary consortium of HPP experts has been established to reclassify VUS using a multi-step process adhering to the ACMG/AMP variant classification guidelines. The process includes clinical evaluation, literature review aided by AI technology, genetic assessment, and in-vitro testing (a co-transfection model using episomal pcDNA3 vectors to express VUS in renal MDCK-II cells and measure ALP residual activity(Farman et al, 2024)).

### Results

The ALPL database currently contains 440 variants. Since Feb 2021, the ALPL gene consortium has examined 79 VUS with reclassifications; 8 pathogenic, 50 likely pathogenic, 2 likely benign, 1 benign and 18 remained VUS. Also, 92 new genotypes and 76 phenotypes were added to the database, resulting from deep literature search. Through studying the reported phenotypes, we discovered - asymptomatic heterozygote individuals featuring the typical biochemical signature of HPP with no overt phenotype (c.1225C>T, c.466C>T, c818C>T, c.244G>A, c.244G>C, c.906C>A, c.83A>G and c.299C>T) and heterozygote individuals with massive ectopic calcifications (c.1559del and c.1250A>G).

**Conclusion**

This classification project and the *ALPL* variant database serve the global medical community. It will broaden the genotypic and phenotypic HPP spectrum by classifying *ALPL* variants based on ACMG/AMP criteria, thus facilitating improved genetic counselling and medical decision-making for affected patients and families. It serves as a repository for clinicians and scientists to query a variant and review evidence of its pathogenicity.

### Disclosure

This project was funded through an externally sponsored research agreement with Alexion, AstraZeneca Rare Disease. Guillermo del Angel is an employee of and may own stock/options in, Alexion, AstraZeneca Rare Disease. Priya S Kishnani, Gabriel Ángel Martos-Moreno, Kathryn M Dahir, Agnès Linglart, Cheryl Rockman-Greenberg, Keiichi Ozono, Eric Rush, Lothar Seefried, and Wolfgang Högler are consultants for and have received research funding and honoraria from, Alexion, AstraZeneca Rare Disease. Catherine Rehder, Erica Burner Nading and Erin Huggins have received honoraria from Alexion.

### OC 4.1

### Assessing the efficacy and safety of Setrusumab for osteogenesis imperfecta: Updated phase 2 data from the phase 2/3 Orbit study

Erik Imel[1], Gary Gottesman[2], Thomas Carpenter[3], Maegen Wallace[4], Peter Smith[5], Hui Wang[6], Heather Byers[6], Stanley Krolczyk[6], E Michael Lewiecki[7]

[1]Indiana University School of Medicine, Indianapolis, USA [2]Washington University School of Medicine, Divisions of Endocrinology and Diabetes, Genetics and Genomics Medicine, Bone and Mineral Diseases, St. Louis, USA [3]Yale University School of Medicine, New Haven, USA [4]Phoenix Children's Hospital, Phoenix, USA [5]Shriner's Hospitals for Children, Chicago, USA [6]Ultragenyx Pharmaceutical Inc., Novato, USA [7]University of New Mexico Health Sciences Center, Albuquerque, USA

### Abstract

**OBJECTIVES:**

Osteogenesis imperfecta (OI) is a rare genetic disorder characterized by low bone mass and bone fragility with no universally accepted treatment. In a prior phase 2 study, setrusumab, a fully human anti-sclerostin monoclonal antibody, improved bone mineral density (BMD), bone strength, and bone turnover markers in adults with OI.

**METHODS:**

In the Phase 2/3 Orbit study (NCT05125809), Phase 2 evaluated setrusumab in children and young-adults with OI based on PK/PD, safety, and BMD data, to determine the dosing strategy for Phase 3. Subjects with OI Types I, III, or IV, ages 5-<26 years, and having fractures within the prior 2 years, were randomized 1:1 to receive 20 or 40 mg/kg setrusumab intravenously monthly. Six-month data as of August 2023 are presented.

**RESULTS:**

Twenty-four subjects (50% female, 75% <18 years of age) with OI Type I (n=17/24, 71%) or III/IV (n=7/24, 29%) were enrolled and randomized to receive 20 mg/kg (n=14/24) or 40 mg/kg (n=10/24) setrusumab.

Mean (SE) spine BMD change from baseline to M3 and M6 was 9.1% (1.8%) and 12.8% (2.5%), respectively (all p<0.05 vs baseline) after setrusumab 20 mg/kg monthly and 9.3% (2.4%) and 16.1% (3.9%) after 40 mg/kg monthly (M6 p<0.05 vs baseline). Spine BMD Z-score [baseline -2.1 (0.8)] increased to -1.5 (0.8) at M3 and -1.3 (0.8) at M6 after 20 mg/kg. After 40 mg/kg, spine BMD Z-score [baseline -1.1 (0.4)] increased to -0.6 (0.4) at M3 and -0.2 (0.4) at M6 (all p<0.05 vs baseline).

Early outcomes at M6 show the median annualized fracture rate reduced significantly from 0.7 to 0 (p=0.042) after setrusumab initiation with a calculated reduction of 67%. Fingers, toes, and craniofacial fractures were excluded. After starting setrusumab, 4/24 (17%) had new radiographically-confirmed fractures reported.

No unexpected adverse events were reported. Treatment-related adverse events included infusion-related reaction, infusion site pain, headache, bone pain, and upper respiratory tract infection.

**CONCLUSIONS:**

In Orbit Phase 2, we observed significant increases from baseline in lumbar spine BMD at M3 and M6 at both 20 and 40 mg/kg doses, with no marked differences between dose groups. Preliminary data suggests decreased fracture rates after setrusumab initiation.

### Disclosure

EAI: Grant/Research Support/Consultant: Ultragenyx Pharmaceutical, Inc.; GSG: Grant/Research Support: Shriners Hospitals for Children, Ultragenyx Pharmaceutical Inc.; Consultant: Ultragenyx Pharmaceutical Inc.; TOC: Grant/Research Support/Consultant: Ultragenyx Pharmaceutical Inc.; MW: Consultant: Novosteo; Advisory Board: Ultragenyx Pharmaceutical Inc.; PS: Grant/Research Support: Ultragenyx Pharmaceutical Inc.; HW, HMB, and SK: Employees of Ultragenyx Pharmaceutical Inc. EML: Grant/Research Support: Amgen, Radius, Ultragenyx Pharmaceutical Inc.; Consultant: Amgen, Radius; Speakers' Bureau: Amgen, Kyowa Kirin

### OC 4.2

### TransCon CNP (Navepegritide) improves physical functioning and well-being in children with achondroplasia (ACH) in the ACcomplisH Trial

Ravi Savarirayan[1,2,3], Daniel G Hoernschemeyer[4], Merete Ljungberg[5], Carlos A Bacino[6], Janet M Legare[7], Teresa Quattrin[8], Yuri A Zarate[9,10], M Jennifer Abuzzahab[11], Paul L Hofman[12], Wolfgang Högler[13], Dirk Schnabel[14], Ricki Carroll[15], Klane K White[16], Sérgio B. Sousa[17], Meng Mao[18], Alden Smith[18], Mads Kjelgaard-Hansen[19], Aimee D Shu[18], Ciara McDonnell[20,21]

[1]Murdoch Children's Research Institute, Parkville, Australia. [2]Royal Children's Hospital, Parkville, Australia. [3]University of Melbourne, Parkville, Australia. [4]University of Missouri Children's Hospital, Columbia, USA [5]Copenhagen University Hospital Rigshospitalet, Copenhagen, Denmark. [6]Baylor College of Medicine, Houston, USA [7]University of Wisconsin School of Medicine and Public Health, Madison, USA [8]University at Buffalo, Buffalo, USA [9]University of Arkansas for Medical Sciences, Little Rock, USA [10]University of Kentucky, Lexington, USA [11]Children's Minnesota, Minneapolis, USA [12]The Liggins Institute, University of Auckland, Auckland, New Zealand. [13]Johannes Kepler University Linz, Linz, Austria. [14]Center for Chronically Sick Children, Charité – University Medicine Berlin, Berlin, Germany. [15]Nemours Children's Hospital, Wilmington, USA [16]Children's Hospital Colorado, Aurora, USA [17]Hospital Pediátrico de Coimbra, Coimbra, Portugal. [18]Ascendis Pharma Inc., Palo Alto, USA [19]Ascendis Pharma A/S, Hellerup, Denmark. [20]Children's Health Ireland at Temple Street, Dublin, Ireland. [21]University of Dublin, Trinity College, Dublin, Ireland

### Abstract

**Objectives:** Clinical manifestations of ACH are associated with significant, potentially life-threatening complications and reduced health-related quality of life (HRQoL). While ACH has historically been considered a growth disorder, manifestations beyond linear growth include reduced muscle strength and stamina and suggest that ACH also impacts muscular function. Further, there is significant unmet need for interventions that ameliorate complications and improve HRQoL in ACH. TransCon CNP is a prodrug of C-type natriuretic peptide (CNP) under investigation for the treatment of ACH. A 1-year analysis from ACcomplisH, a phase 2, multicenter, randomized, double-blind, placebo-controlled, dose-escalation trial of once-weekly TransCon CNP versus placebo in children with ACH, is presented here.

**Methods:** Prepubertal participants (aged 2-10y) were randomized 3:1 to receive TransCon CNP across 4 dose-escalation cohorts, or placebo for 52 weeks, after which participants could receive TransCon CNP in an open-label extension. HRQoL assessments compared 52-week results from participants whose initial TransCon CNP dose was 100µg/kg/week with results from the pooled placebo group in the randomized period. P-values are nominal. Safety was reported for all participants over the entire study duration.

**Results:** 57 participants (mean age [SD], 5.9y [2.8]; 42% ≥5y; 42% female) were enrolled; 100% continued into the open-label extension. Significant improvements were observed for Daily Living Functioning ($p=0.047$; n=16;13 [treated;placebo]) and Emotional Well-Being ($p=0.045$; n=13;9) domains of the Achondroplasia Child Experience Measure-Impact assessment, and the SF-10 Physical Summary among participants ≥5y ($p=0.002$; n=9;5). Growth across the full trial population (n=57) on TransCon CNP 100µg/kg/week for 52 weeks was consistent with results from this dose cohort during the randomized period. Overall, there were no safety signals; no treatment-related serious adverse events (AEs) were reported. Most treatment-emergent (TE)AEs were Grade 1-2; no TEAEs led to treatment modifications or death. No trends were observed in ACH-related AEs.

**Conclusion:** Participants who received TransCon CNP 100μg/kg/week demonstrated significant improvements in well-being and physical functioning at 52 weeks. Once-weekly TransCon CNP continues to offer clinical benefits with a favorable safety profile. These results support the continued development of TransCon CNP for ACH and further elucidation of the mechanisms by which TransCon CNP can potentially improve outcomes beyond linear growth, including radiological endpoints, HRQoL, physical functioning, and body composition.

### Disclosure

RS: advisory board role for Ascendis and BioMarin; and consulting role for BridgeBio. DGH: stock or other ownership in, consulting role for, patents, royalties, or other intellectual property from, and travel support from Orthopediatrics; honoraria from BioMarin; research funding from BioMarin and Zimvie; speakers' bureau participation for and grants from Zimvie; and other relationship with Orthopediatrics and Zimvie. ML: consulting role for and travel support from Ascendis. CAB: consulting role for Best Doctors, Inc; patents, royalties, or other intellectual property from UpToDate; and institutional grants from Ascendis, BioMarin, Ionis, and Roche. JML: honoraria from, research funding from, speakers' bureau participation for, and grants from BioMarin. TQ: consulting role for Ascendis, Janssen, Merck, Sanofi; and research funding from Ascendis, Janssen, Merck, Pfizer, and Provention Bio. YAZ: no relevant financial relationships to disclose. MJA: advisory board role for Ascendis and Pfizer; consulting role for Endo and Rhythm; institutional research funding from Ascendis, Lumos, Mannkind, Medtronic, Novo Nordisk, Rhythm, and Soleno; and other relationship as DSMB for LG. PH: honoraria from Eli Lilly and Novo Nordisk; and research funding from Ascendis and Novo Nordisk. WH: honoraria from Alexion; advisory board role for Alexion and BioMarin; consulting role for Alexion and Pfizer; research funding from Alexion, Amgen, and Kyowa Kirin; travel support from Alexion and Kyowa Kirin; and grants from Novo Nordisk. DS: honoraria from, consulting role for, and travel support from Kyowa Kirin; and advisory board role for BioMarin, Kyowa Kirin, Novo Nordisk, and Sandoz. RC: honoraria from Actio; advisory board role for Biomarin; consulting role for Guidepoint; and research funding from Biomarin, Ascendis, Ultragenyx, and Medlife.. KKW: honoraria from BioMarin; research funding from Ascendis, BioMarin, Pfizer, and Ultragenyx; and patents, royalties, or other intellectual property from UpToDate. SBS: honoraria from Ascendis, BioMarin, and Kyowa Kirin; advisory board role for Ascendis and BioMarin; and travel support from BioMarin. MM, AS, MK-H, ADS: employment with and stock or other ownership in Ascendis. CM: honoraria from BioMarin and Pfizer; advisory board role for Pfizer; research funding from Ascendis and Kyowa Kirin; and travel support from Pfizer.

### OC 4.3

### Persistent growth-promoting effects of vosoritide in children with achondroplasia is accompanied by improvement in physical aspects of quality of life

Ravi Savarirayan[1], Louise Tofts[2], Melita Irving[3], William R Wilcox[4], Carlos A Bacino[5], Julie E Hoover-Fong[6], Paul Harmatz[7], Frank Rutsch[8], Ricki S Carroll[9], Lynda E Polgreen[10], Klaus Mohnike[11], Joel Charrow[12], Carlos Prada[12], Daniel Hoernschemeyer[13], Keiichi Ozono[14], Takuo Kubota[14], Yasemin Alanay[15], Paul Arundel[16], Yumiko Kotani[17], Natsuo Yasui[17], Klane K White[18], Shelley Brandstetter[18], Howard M Saal[19], Antonio Leiva-Gea[20], Hiroshi Mochizuki[21], Asako Tajima[21], Donald Basel[22], Elena Fisheleva[23], Richard Rowell[23], Alice Huntsman-Labed[23], Jonathan Day[23]

[1]Murdoch Children's Research Institute, Royal Children's Hospital, and University of Melbourne, Parkville, Australia. [2]Kids Rehab, The Children's Hospital at Westmead, Westmead, Australia. [3]Guy's and St. Thomas' NHS Foundation Trust, London, UK [4]Emory University, Atlanta, USA [5]Baylor College of Medicine, Houston, USA [6]Johns Hopkins University School Of Medicine, Baltimore, USA [7]UCSF Benioff Children's Hospital, Oakland, USA [8]Department of General Pediatrics, Muenster University Children's Hospital, Münster, Germany. [9]Nemours/Alfred I. du Pont Hospital for Children, Wilmington, USA [10]Lundquist Institute for Biomedical Innovation at Harbor-UCLA Medical Center, David Geffen School of Medicine, Torrance, USA [11]Otto-von-Guericke Universität, Universitätskinderklinik, Magdeburg, Germany. [12]Ann and Robert H. Lurie Children's Hospital of Chicago, Chicago, USA [13]University of Missouri-Columbia, Columbia, USA [14]Osaka University Hospital, Osaka, Japan. [15]Acibadem Mehmet Ali Aydinlar University, School of Medicine, Istanbul, Turkey. [16]Sheffield Children's NHS Foundation Trust, Sheffield Children's Hospital, Sheffield, UK [17]Tokushima University Hospital, Tokushima, Japan. [18]Colorado Children's Hospital, Colorado, USA [19]Cincinnati Children's Hospital Medical Center, Cincinnati, USA [20]Hospital Universitario Virgen de la Victoria, Málaga, Spain. [21]Saitama Children's Hospital, Saitama, Japan. [22]Medical College of Wisconsin, Milwaukee, USA [23]BioMarin (U.K.) Limited, London, UK

### Abstract

### Objectives:

Vosoritide is a potent stimulator of endochondral bone growth and is approved for use in children with achondroplasia (ACH) until closure of epiphyses. We report data from Quality of Life in Short Stature Youth (QoLISSY) questionnaires collected for treated children in a phase 3 study (111-301) and its ongoing open-label extension (111-302).

### Methods:

Children and/or their caregivers completed the QoLISSY questionnaire at baseline and at 6-month intervals. As of February 2023, data collection was complete up to Year 3. Descriptive summaries assessed mean annual changes from baseline for each domain and total score (based on physical, social, and emotional domains) for caregiver and self-reported questionnaires for all children and for children with ≥1 SD ACH height z-score improvement at Year 3. Untreated data from vosoritide clinical trials were modelled to estimate expected yearly changes in each domain score in the untreated setting to facilitate interpretation of changes observed in the treated population.

**Results:**

Data were available from 119 (100%) children (47% female; mean (SD) age 9.7 (2.6) years, range 5.6-16.4 years). Mean baseline QoLISSY total scores were 57.5 (19.0) as reported by caregivers (n=119) and 64.5 (18.4) as self-reported by children (n=73) (range: 0-100, higher score indicates better QoL). After 3 years, mean (SD) change in physical domain score was 6.0 (19.4) for caregiver-reported (n=99) and 6.3 (20.2) for self-reported (n=60); changes in other domains were less pronounced after 3 years (change in total scores were 3.3 (15.5) for caregivers and 5.4 (17.7) for self-reported). In children with ≥ 1 SD increase in ACH height z-score, mean changes in physical domain scores were 11.4 (19.5) for caregiver-reported score (n= 38) and 8.5 (21.8) for self-reported score (n=28). Mixed models with fixed effects for age and sex and random subject effect for the untreated population provided an estimated annual slope (SE) of 0.16 (0.55) and 1.45 (0.77) for caregiver-reported and self-reported physical domain scores, respectively.

**Conclusions:**

Vosoritide improves health related quality of life among children with ACH by improving physical domains, particularly in children with a pronounced change in height z-score over time.

**Disclosure**

All authors are investigators in this clinical trial except for EF, RR, AHL and JD, who are employees of the funder (BioMarin). RS, LT, FR, and KM have received consulting fees and grants from BioMarin. MI and WW have received consulting fees from BioMarin. JC and DB have received grants from BioMarin. LP, PA and RC have received honoraria from BioMarin. CB and PH have received consulting fees, honoraria and grants from BioMarin. JHF has received consulting fees from BioMarin, Therachon AG and Ascendis, and grants from BioMarin. KW has received consulting fees from BioMarin and Sanofi/Genzyme, and grants from BioMarin, Ultragenyx, and Ascendis. The other authors declare no competing interests.

### OC 4.4

### TYRA-300 demonstrates significant increases in bone length and foramen magnum area in a mouse model of FGFR3-related skeletal dysplasia

Clara Lemoine[1], Matthias Guillo[1], Nabil Kaci[1], Jacqueline Starrett[2], Ronald Swanson[2], Laurence Legeai-Mallet[3]

[1]Institut Imagine-InsermU1163, Paris, France. [2]Tyra Biosciences, Carlsbad, USA [3]Institut Imagine-InsermU1163, Paris, USA

### Abstract

OBJECTIVES: Achondroplasia (ACH) is the most common human skeletal dysplasia affecting ~1 in 25,000 births. Infants with ACH have an increased risk for death related to critical foramen magnum stenosis leading to cervicomedullary compression. A specific mutation in *FGFR3*, G380R, causes over 99% of achondroplasia. FGFR3 is expressed in growth plate chondrocytes where it functions to disturb endochondral bone formation. The G380R mutation, as well as other activating mutations, results in increased FGFR3 activity, which disrupts chondrogenesis in the growth plate, disturbing long bone elongation. Vosoritide, a C-natriuretic peptide analogue, acting exclusively on the MAP kinase pathway, was recently approved as a daily injection to increase annual growth velocity in children with open growth plates. While an important breakthrough, long-term effects on ACH-associated comorbidities are not yet known. TYRA-300 is an oral, highly selective FGFR3 tyrosine kinase inhibitor currently undergoing a Phase 1/2 clinical trial, SURF301 (Study in Untreated and Resistant FGFR3+ Advanced Solid Tumors). To explore the effectiveness of TYRA-300 in FGFR3-related chondrodysplasias, TYRA-300 was evaluated in $Fgfr3^{Y367C/+}$ mice mimicking ACH.

METHODS: TYRA-300 was administered daily at a 1.2 mg/kg dose for 15 days in the $Fgfr3^{Y367C/+}$ mouse model.

RESULTS: TYRA-300 treatment significantly increased body length in $Fgfr3^{Y367C/+}$ mice by 17.9% compared to the vehicle (p<0.0001) and increased the length of the femur (+22.6%), tibia (+33.0%) and L4-L6 (+23.5%) (p<0.0001). mCT analyses showed a significant increase of the foramen magnum area (+15.08%) in TYRA-300 treated $Fgfr3^{Y367C/+}$ mice compared to vehicle treated mice (p<0.0001). Histological staining of the femurs revealed restoration of the hypertrophic zone and secondary ossification center within the epiphysis after TYRA-300 treatment. Collagen X staining also indicated that TYRA-300 improved the structure of the trabecular bone and increased chondrocyte differentiation.

CONCLUSIONS: These data indicate that inhibiting FGFR3 directly leads to highly increased bone length, as well as foramen magnum area, in this preclinical model.

### Disclosure

Jacqueline H Starrett and Ronald V Swanson are employed by Tyra Biosciences

### OC 4.5

### Effect of Burosumab on growth trends in patients with X-linked hypophosphatemia

Leanne Ward[1], Thomas Carpenter[2], Suzanne Jan de Beur[3], Hamilton Cassinelli[4], Erik Imel[5], Jill Simmons[6], Anna Ryabets-Lienhard[7], Zhiyi (Jerry) Li[8], Zunqiu (Eric) Chen[9], Antonio Ramirez[9], Osman Ciğeroğlu[9]

[1]University of Ottawa, Children's Hospital of Eastern Ontario, Ontario, USA [2]Yale University School of Medicine, New Haven, USA [3]University of Virginia, Charlottesville, USA [4]CEDIE, Centros de Investigaciones Endocrinologicas Dr Cesar Bergada, Division de Endocrinologia, Hospital de Niños Ricardo Gutierrez, Buenos Aires, Argentina. [5]Indiana University School of Medicine, Indianapolis, USA [6]Department of Pediatrics, Division of Endocrinology and Diabetes, Vanderbilt University School of Medicine, Vanderbilt University, Nashville, USA [7]Center for Endocrinology, Diabetes and Metabolism, Saban Research Institute, Children's Hospital Los Angeles, Los Angeles, USA [8]Kyowa Kirin, Inc., Princeton, USA [9]Ultragenyx Pharmaceutical Inc., Novato, USA

### Abstract

#### Objectives:

X-linked hypophosphatemia (XLH) is a rare, musculoskeletal disorder characterized by rickets/osteomalacia leading to bone pain, skeletal deformities, and short stature. This analysis explores growth patterns in patients receiving burosumab from the XLH Disease Monitoring Program (DMP; NCT03651505) supplemented with data from prior XLH studies via DataLink, a compilation of clinical trial data.

#### Methods:

The impact of XLH on age-dependent growth velocity was assessed from patient records (regardless of burosumab treatment) aged <25 years at DMP or other XLH study entry with Center for Disease Control (CDC) reference values. We examined height Z-score by age in the study population regardless of burosumab status. To assess the impact of burosumab, changes in height Z-score from first burosumab dose or observation (burosumab-naïve) were examined separately by treatment/observation duration.

Records represent study visits by treatment status; patients may have multiple records by age. Data are presented based on availability of endpoints for each record.

#### Results:

From June 2023, data were available from 592 patients (burosumab-naïve and burosumab-treated combined) aged <25 years at study entry. Median CDC reference growth velocity peaked at age 13 years in males and 11 in females. In patients with XLH (burosumab-naïve and treated combined), median growth velocity peaked at age 15 years in males and 12 in females, remaining above CDC values through age 18 when growth stopped in both cohorts. Mean height Z-scores were descriptively greater in patients receiving burosumab treatment compared with burosumab-naïve patients across most ages assessed. Height Z-score changes from baseline in treated and burosumab-naïve patients age <8 were compared by treatment/observation duration at each record date **(Figure 1)**. Over the first 3 years, height Z-score decreased in burosumab-naïve patients and remained constant in treated patients ($p<0.001$).

**Figure 1: Mean Change in Height Z-Score From First Observation or Treatment in Patients <8 Years of Age**

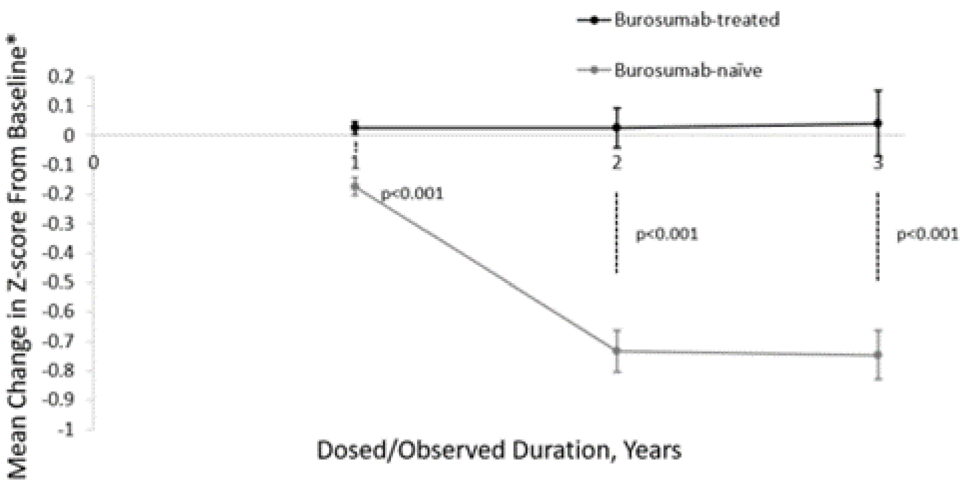

n, records =

| | | | |
|---|---|---|---|
| Burosumab Treated | 474 | 100 | 58 |
| Burosumab-naïve | 876 | 369 | 297 |

*Baseline defined as time of first observation or burosumab treatment

**Conclusions:**

XLH-related growth deceleration appears evident in the first 2 years of life with a delay in peak growth velocity. Burosumab-treated patients had descriptively higher mean height Z-scores versus burosumab-naïve. Over 3 years, mean height Z-scores decreased significantly in burosumab-naïve patients compared with burosumab-treated, which were maintained over the same period. Available data suggest burosumab treatment may mitigate the negative impacts of XLH on growth velocity.

### Disclosure

LW: Consultant to and participant in clinical trials with Ultragenyx Pharmaceutical Inc. (with funds to Dr. Ward's institution, the Children's Hospital of Eastern Ontario Research Institute) TC: Research Investigator (funds to institution): Ultragenyx; Advisory Board and consulting: Ultragenyx Pharmaceutical Inc, Inozyme,Kyowa Kirin; Consulting: Viridian SJdB: Ultragenyx research investigator, scientific advisory board, consultant, Kyowa Kirin scientific advisory board, consultant, Inozyme scientific advisory board, Mereo research investigator HC: Research Support: Ultragenyx Pharmaceutical Inc. EI: Research funding to institution from Ultragenyx and Kyowa Kirin, and consulting for Ultragenyx and Kyowa Kirin. JS: Research funding to institution from Ultragenyx and consulting to Ultragenyx AR-L: Research funding to institution from Ultragenyx and consulting to Ultragenyx ZL: Employee: Kyowa Kirin Inc. ANR, EC, OC: Employee, Stockholder: Ultragenyx Pharmaceutical Inc.

### OC 4.6

### Incidence and outcomes of premature physeal closure in paediatric patients with fibrodysplasia ossificans progressiva in the palovarotene clinical development programme

Geneviève Baujat[1], Robert J Pignolo[2], Mona Al Mukaddam[3], Carmen De Cunto[4], Edward C Hsiao[5], Richard Keen[6], Jean-Luc Delhay[7], Fei Shih[8], Andrew Strahs[8], Frederick S Kaplan[3]

[1]Département de Génétique, Hôpital Universitaire Necker-Enfants Malades, Institut Imagine, Université Paris Cité, Paris, France. [2]Department of Medicine, Mayo Clinic, Rochester, MN, USA [3]Departments of Orthopaedic Surgery and Medicine, Center for Research in FOP and Related Disorders, Perelman School of Medicine, University of Pennsylvania, Philadelphia, PA, USA [4]Pediatric Rheumatology Section, Department of Pediatrics, Hospital Italiano de Buenos Aires, Buenos Aires, Argentina. [5]Division of Endocrinology and Metabolism, the UCSF Metabolic Bone Clinic, the Eli and Edyth Broad Institute for Regeneration Medicine, and the Institute of Human Genetics, Department of Medicine, and the UCSF Program in Craniofacial Biology, University of California San Francisco, San Francisco, CA, USA [6]Centre for Metabolic Bone Disease, Royal National Orthopaedic Hospital, Stanmore, UK [7]Ipsen, Les Ulis, France. [8]Ipsen, Cambridge, MA, USA

### Abstract

### OBJECTIVES

Fibrodysplasia ossificans progressiva (FOP) is an ultra-rare genetic disorder that manifests in childhood, characterised by heterotopic ossification (HO). Palovarotene, a selective retinoic acid receptor-γ agonist, is the first approved treatment for the reduction of new HO in patients aged ≥8 (females) and ≥10 years (males) with FOP, supported by interim results from the pivotal phase III MOVE trial (NCT03312634).[1] In MOVE, palovarotene was generally well tolerated, but there was risk of premature physeal closure (PPC) in paediatric patients, consistent with known effects of systemic retinoids.[1] Here, pooled long-term analyses of PPC events in paediatric patients in MOVE and a phase II open-label extension (OLE; NCT02279095) are reported.

### METHODS

Patients received palovarotene for ≤48 months in MOVE and ≤60 months in the phase II OLE. Physeal status was assessed by hand/wrist and knee radiographs every 3–6 months. PPC events were recorded based on MedDRA (version 22.0) and included partial or complete growth plate closure; all were categorised as serious adverse events. Pooled analyses encompassed data from screening through January 2022.

### RESULTS

Of 102 (MOVE: 80; phase II OLE: 22) patients aged <18 years at enrolment, 27 (26.5%) had clinical events consistent with PPC, all of which occurred in those aged <14 years (**Table**). PPC occurred 6–30 months after initiating palovarotene, and 2 events were detected after treatment discontinuation. PPC was most commonly detected at the femur (20/27 [74.1%]; **Table**). Most PPC events were partial closure at all locations.

In 13 patients aged ≥8/10 years with PPC, 6 reached a normal adult height by last visit (average height z-score at last visit: 0.7). The remaining 7 patients had a height below the fifth percentile at end of study (average height z-score at last visit: –1.7); 3 of these patients had shown growth deceleration before initiating palovarotene.

**CONCLUSION**

PPC is a major risk associated with palovarotene in paediatric patients with open growth plates. For growing patients, the potentially irreversible risks of PPC must be closely monitored and weighed against the benefits of palovarotene in reducing new HO volume.

**References: 1.** Pignolo RJ. J Bone Miner Res. 2023;38(3):381–94.

**Table.** Incidence of PPC events in paediatric patients by age group, sex and location in the phase III MOVE trial and a phase II OLE

| PPC events | n/N | % |
|---|---|---|
| **Total (patients aged <18 years)** | 27/102 | 26.5 |
| **Age**[a] | | |
| <8/10 years | 14/25 | 56.0 |
| ≥8/10 to 14 years | 13/42 | 31.0 |
| ≥14 to <18 years | 0/35 | 0.0 |
| **Sex**[b] | | |
| Female | 14/47 | 29.8 |
| Male | 13/55 | 23.6 |
| **Location**[c] | | |
| Femur | 20/27 | 74.1 |
| Tibia | 16/27 | 59.3 |
| Radius | 4/27 | 14.8 |
| Ulna | 2/27 | 7.4 |

[a]Ages are reported as female/male and patients were grouped by age at enrolment; n/N is the number of patients with PPC/total number of patients in each age group. [b]n/N is the number of patients with PPC/total number of patients by sex. [c]Includes patients with a PPC event status of closed or partially closed; n/N is the number of patients with PPC at each location/total number of patients with PPC. OLE: open-label extension; PPC: premature physeal closure.

### Disclosure

Funding: This study was sponsored by Ipsen. Disclosures: MAM: Research Investigator: Clementia/Ipsen, Incyte, Regeneron; Non-paid consultant: Biocryst, Blueprint, Daiichi Sankyo, Incyte, Keros; Advisory Board: IFOPA Registry Medical Advisory Board, International Clinical Council on FOP (all voluntary); GB: Research Investigator: Alexion, BioMarin, Clementia/Ipsen, Incyte; Advisory Board: Clementia/Ipsen; Speaker: Clementia/Ipsen; FOP European Consortium, International Clinical Council on FOP, IFOPA

Registry Medical Advisory Board (all voluntary); CDC: Research Investigator: Clementia/Ipsen; Member of the International Clinical Council on FOP; ECH: Research support: Ascendis, Clementia/Ipsen, Ultragenyx; Research Investigator: Ascendis, Clementia/Ipsen; Member of the International Clinical Council on FOP, Fibrous Dysplasia Foundation, IFOPA Registry Advisory Board (all voluntary); RK: Research Investigator: Clementia/Ipsen, Kyowa Kirin, Regeneron; Advisory Board: IFOPA Registry Medical Advisory Board, International Clinical Council on FOP; RJP: Research Investigator: Clementia/Ipsen, Incyte, Regeneron; Advisory Board: Immediate Past President of the International Clinical Council on FOP; JLD, FS, AS: Employees of Ipsen; FSK: Research Investigator: Clementia/Ipsen, Incyte, Regeneron; Advisory Board: IFOPA Medical Advisory Board; Founder and Past President of the International Clinical Council on FOP.

### OC 5.1

### Adaptive response to loading is impaired in a mouse model of osteogenesis imperfecta coincident with altered periosteal lacunar properties

David T Bertrand[1,2], Josephine T Tauer[1,3,4], Joan C Marini[5], Frank Rauch[1,6], Bettina M Willie[1,2]

[1]Shriners Hospital for Children, Research Center, Montreal, Canada. [2]Faculty of Dental Medicine and Oral Health Sciences, McGill University, Montreal, Canada. [3]Faculty of Dental Medicine and Oral Health Sciences, McGill University, Montreal, Canada. [4]Department of Pediatrics and Adolescent Medicine, Johannes Kepler University Linz, Linz, Austria. [5]Section on Heritable Disorders of Bone and Extracellular Matrix, NICHD, Bethesda, USA [6]Department of Pediatrics, McGill University, Montreal, Canada

### Abstract

### Introduction

Osteogenesis imperfecta (OI) is characterized by increased bone fragility, frequent fractures, and decreased diaphyseal cross-sectional area in long bones. We hypothesize this reduced bone circumference is the result of reduced periosteal apposition caused by an altered response to mechanical loading.

### Methods

Bone adaptive response after two weeks (5 days/week) of in vivo tibial loading was assessed at 10N (1500με) in ten-week-old female Brtl+/- and wild type (WT) littermates (n=10/genotype; left tibia loaded, right tibia non-loaded control). Assessment of baseline and endpoint bone mass and microstructure was carried out via in vivo microCT. MicroCT-based timelapse morphometry was performed to assess endocortical (Ec) and periosteal (Ps) newly mineralized and eroded volumes normalized to bone volumes over the two-week experimental period (Figure 1B). Lacunar properties were analyzed using synchrotron tomography at the European Synchrotron Research Facility (ESRF, France). ANOVA, Tukeys post-hoc, and paired t-tests were applied ($p<0.05$).

### Results

At baseline, as expected, Brtl+/- mice showed significantly reduced cortical area (CtAr), total area (TtAr) marrow area (MaAr) and cortical thickness (CtTh) compared to WT littermates (Figure 1B). Following two weeks of loading, ΔCtAr, ΔCtTh, and, Ec. Mineralizing volume over bone volume (MV/BV) were significantly increased in loaded compared to non-loaded limbs for both genotypes. However, strikingly, ΔTtAr and Ps. MV/BV were significantly increased in the loaded vs non-loaded limb of WT mice, but not in Brtl+/- mice, suggesting an altered formation response on the periosteal surface (Figure 1C). In terms of resorption, both Ec and Ps EV/BV were reduced in loaded compared to control limb of both genotypes. Synchrotron tomography revealed that lacunar density was increased in Brtl+/- compared to WT mice, while mean lacunar volume was decreased when analyzed across the entire cross-section (total VOI) (Figure 1D). However, within only the periosteal VOI, lacunar density was unchanged, while lacunar mean volume was lower in non-loaded limbs of Brtl+/- compared to WT mice (Figure 1D).

### Conclusion

Our findings indicate an impaired bone formation response to loading on the periosteal surface in Brtl+/- mice, coincident with altered lacunar properties, likely contributing to decreased diaphyseal cross-sectional area in long bones in these mice.

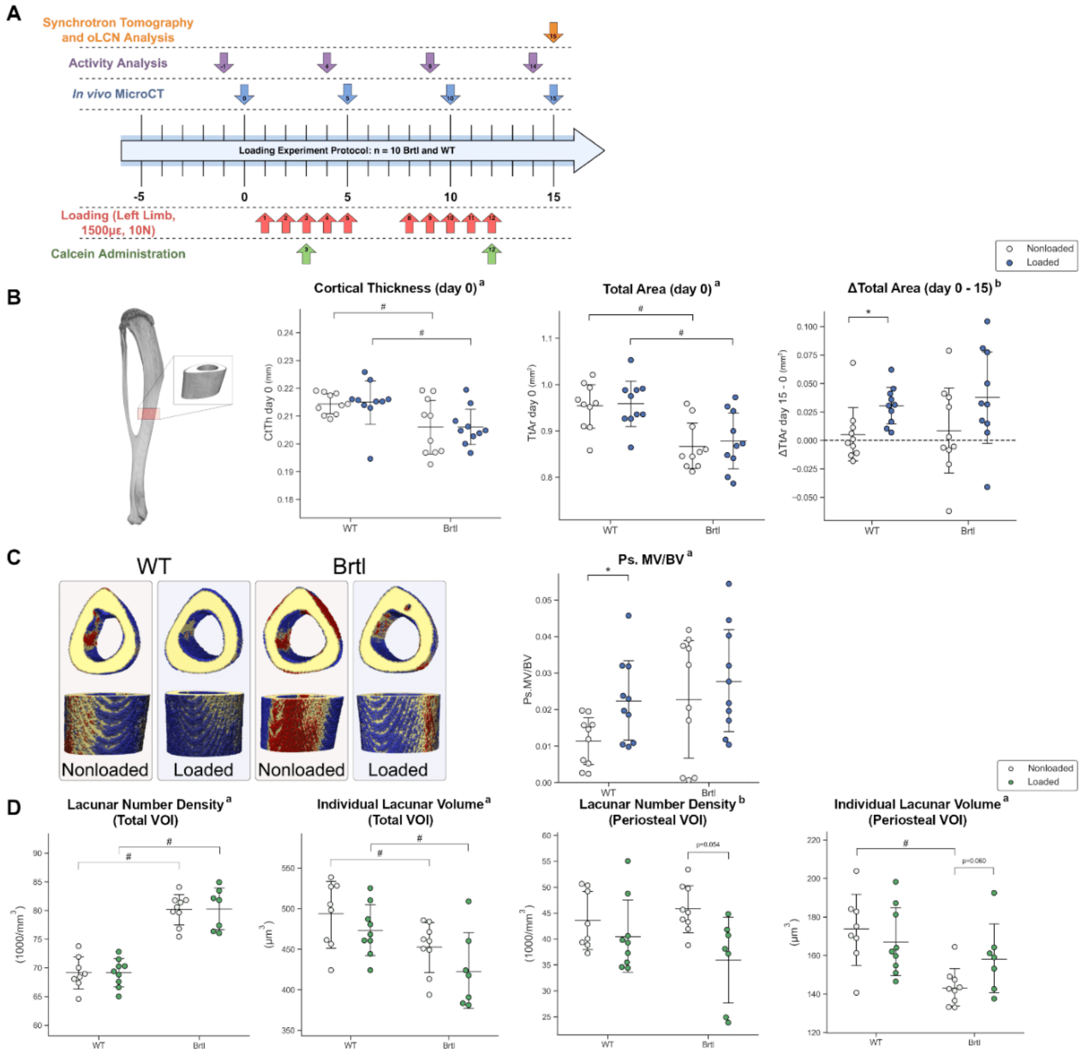

**Figure 1**: Two-week in-vivo loading tibial experiment in *Brtl*$^{+/-}$ mice and WT littermates. A) Experimental design and timeline. B) µCT analysis revealed significantly reduced total area and cortical thickness at baseline. After 2 weeks of loading, the change in total area increased for WT mice in loaded compared to nonloaded limbs, but this increase was not significant in Brtl mice. C) 3D images derived from µCT data and timelapse analysis showing formed, quiescent, and resorbed bone between baseline and the experimental endpoint at day 15. Time-lapse analysis revealed that the amount of newly formed bone normalized to the total bone volume in the periosteal region (Ps. MV/BV) was significantly increased by loading in WT, but not in Brtl mice. D) Synchrotron tomography analysis performed on the entire VOI revealed that the Brtl mice had a higher lacunae density than WT. In the periosteum, however, lacunar density remained unchanged between genotypes, while individual lacunar volume was lower in Brtl compared to WT mice. Statistical analysis carried out with ANOVA: a) Genotype, b) Loading, c) Interaction, as well as Tukey post-hoc tests (#) or paired t-tests (*), $p < 0.05$.

### OC 5.2

### Exogenous HSP47 reduces collagen overmodifications favouring its secretion and extracellular matrix deposition in osteogenesis imperfecta

Roberta Besio[1], Nadia Garibaldi[1], Alessandra Sala[1], Francesca Tonelli[1], Carla Aresi[1], Elisa Maffioli[2], Antonio Rossi[1], Gabriella Tedeschi[2], Antonella Forlino[1]

[1]Department of Molecular Medicine, University of Pavia, Pavia, Italy. [2]Department of Veterinary Medicine and Animal Sciences, University of Milan, Lodi, Italy

### Abstract

OBJECTIVES: Several recessive and most of the dominant mutations causing osteogenesis imperfecta (OI) delay collagen I chains folding, increasing the exposure to post translational modifications that impair collagen secretion and fibrils assembly into the extracellular matrix.

Physiologically heat shock protein 47 (HSP47) is the collagen specific chaperone facilitating its folding, limiting its intracellular aggregation and allowing its secretion. Here we tested in OI patients' cells and in an OI zebrafish model the ability of recombinant HSP47 (rHSP47) to target collagen I synthesis, modulating its post translational modification levels and favoring its secretion.

METHODS: Using primary fibroblasts from individuals with defect in the prolyl-3-hydroxylation complex (OI type VII and VII), we evaluated the exogenous rHSP47 uptake by confocal imaging, and the effect on collagen post translational modifications, secretion, and deposition into the matrix by tandem mass spectrometry, collagen chase and collagen quantification assay. Also, the general ER proteostasis, cellular homeostasis and vitality were analysed by thioflavin T staining, transmission electron microscopy and FACS analyses. The effect of rHSP47 on bone mineralization was tested *in vivo* in the zebrafish *p3h1*$^{-/-}$ OI model.

RESULTS: We proved that the exogenous rHSP47 is able to be uptaken by the cell and localize at the endoplasmic reticulum exit sites and endoplasmic reticulum Golgi intermediate compartment. rHSP47 increased collagen secretion, reduced collagen post translational modifications and intracellular collagen retention and ameliorated the general ER proteostasis, leading to a substantial improvement in cellular homeostasis and vitality. These positive changes were also mirrored by an increased content of collagen I into the OI probands matrix. *In vivo* the administration of rHSP47 improved skeletal development by increasing bone mineralization in the zebrafish *p3h1*$^{-/-}$ OI model, supporting HSP47 beneficial effect on osteoblast anabolic activity.

CONCLUSIONS: The reported data provide the proof-of-principle for exogenous rHSP47 administration as a promising treatment for diseases characterized by poor collagen I secretion and matrix incorporation.

Funding: This work was supported by Fondazione Telethon grant no. GMR22T1024 to RB; PRIN2022 ERWJKZ to RB; PRIN2022 3C8C5B to AF.

### OC 5.3

### A promising adjuvant treatment for growing mice with moderate-to-severe osteogenesis imperfecta: A pilot study of a CNP analog

Jack Mulcrone[1], Ketsia Seide[1], Erin Carter[1], Nancy Pleshko[2], Cathleen Raggio[1]

[1]Hospital for Special Surgery, New York, USA [2]Temple University, Philadelphia, USA

### Abstract

OBJECTIVES:

Osteogenesis imperfecta (OI) is a heterogenous type 1 collagenopathy characterized by fragile bones. Bisphosphonates reduce fracture incidence and are used to manage moderate-to-severe OI in children. C-type natriuretic peptide (CNP) is produced in the growth plate and positively regulates linear bone growth; an unpublished study by Bober et al. found that people with OI have a reduced level of CNP. This study evaluates if the addition of a CNP analog to standard bisphosphonate treatment (alendronate (ALN)) will reduce fracture incidence and increase bone strength, growth, and bone mineral density (BMD) in growing *oim/oim* (oim) mice.

METHODS:

At two weeks of age, growing oim mice (N=27) were divided into four groups: control mice (N=8) receiving saline treatment (saline) and treated mice receiving weekly ALN with one of three CNP dosages: 10 ug/kg three days/week (10x3)(N=6), 20 ug/kg three days/week (20x3)(N=6), or 20 ug/kg five days/week (20x5)(N=7). Faxitron images were taken at two, eight, and 14 weeks (sacrifice) to assess fracture incidence and measure femoral length and vertebral height. Microcomputed tomography was used to assess bone microstructural parameters.

RESULTS:

The 20x5 group had no fractures post-sacrifice; there were two mice with one fracture each in the 10x3 and 20x3 groups. Femoral length was increased in all treated groups in comparison to saline mice; the 20x5 group had the largest increase and the 10x3 and 20x3 groups had similar increases. Vertebral height was increased in all treated groups; the 10x3 and 20x5 groups had greater, comparable increases than the 20x3 group. All treated groups had an increase in trabecular BMD. All three treated groups had increased cortical TMD, BMD, and thickness.

CONCLUSIONS:

Adjuvant treatment with the CNP analog increased femoral length and vertebral height without compromising fracture reduction, as well as showed added benefits for trabecular and cortical bone (not observed with bisphosphonates alone). These results will be used to identify the optimal dose of the CNP analog and a full murine study will be conducted to investigate the potential for use in humans.

Figure 1: Trabecular Bone Representative 3D Images

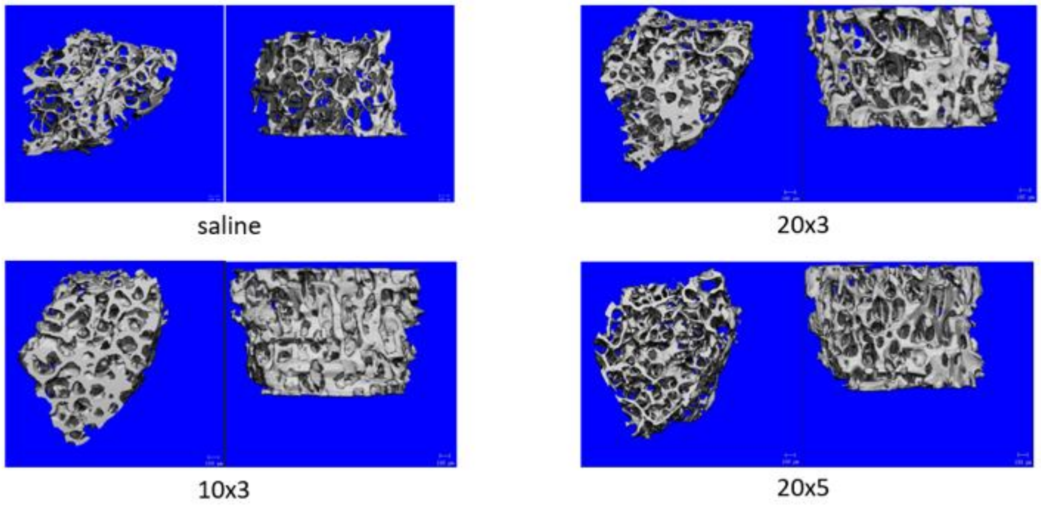

**Disclosure**

Dr. Cathleen Raggio; BioMarin - Grant, Speaker, Consultant Dr. Nancy Pleshko; BioMarin - Grant

### OC 6.1

### Fibrous dysplasia progression in patients with craniofacial involvement of the frontal and zygomatic bones

Jonathan Freeman[1,2], Kristen Pan[3], Ibrahim Elbashir[1], Alison Boyce[1]

[1]National Institutes of Health, Bethesda, USA [2]University of Nevada, Reno School of Medicine, Reno, USA [3]Department of Plastic and Reconstructive Surgery, The Johns Hopkins Medical Institutions, Baltimore, USA

### Abstract

Objectives: Fibrous dysplasia (FD) is characterized by expansile fibro-osseous lesions that may occur in association with endocrinopathies as part of McCune-Albright syndrome (MAS). The purpose of this study was to evaluate expansion rates in craniofacial lesions associated with significant morbidity, including the frontal bone and zygomas.

Methods: Patients with craniofacial FD and serial CT imaging from a natural history study (NCT00001727) at the NIH were evaluated. Volumetric analyses of CT scans were performed using MIM Encore software version 7.3.3 (MIM Software Inc., Cleveland, Ohio). Generalized mixed model analysis was used to account for intra-subject correlation, with FD lesion volume as the dependent variable. In addition to age, effects of MAS-associated endocrinopathies, sex, Skeletal Disease Burden Score (a quantitative measure of total skeletal FD involvement), and history of bisphosphonate treatment were evaluated.

Results: 64 total lesions (frontal N =28, zygomatic N=36) in 33 patients were evaluated longitudinally in serial CT scans. Frontal and zygomatic lesion volume increased with age ($p<0.001$), and expansion rate decreased over time ($p<0.001$). There were no associations between expansion rates and MAS-associated endocrinopathies, sex, Skeletal Disease Burden Score, or bisphosphonate treatment. Compared to recently reported expansion rates in gnathic bones in this cohort (Pan et al J Bone Miner Res 2023, mandibular=42, maxillary=65), frontal FD lesions expanded at a greater rate than all other lesions (5.19 $cm^3$/year, 95% CI 4.33–6.05, $p<0.001$), followed by mandibular FD lesions (0.99 $cm^3$/year, 95% CI 0.15–1.85 $p<0.022$). There was no difference in expansion rates between maxillary and zygomatic lesions.

Conclusions: Craniofacial lesion expansion rates are most rapid in younger children and decline as patients approach adulthood. FD lesions involving the frontal bone and mandible tend to expand at greater rates than other facial bones. These differences in growth rates indicate that location-specific analyses are required to assess FD progression. The availability of quantitative natural history data will guide clinicians in identifying patients who are candidates for medical and surgical interventions and clinical trials for preventative therapies.

### Disclosure

Dr. Alison Boyce: Amgen, Ultragenyx, Kyowa Kirin, BridgeBio

### OC 6.2

### An open-label, multicentre, non-randomised study investigating the safety, tolerability and efficacy of burosumab in infants with X-linked hypophosphataemia: Results from the BUR-CL207 study

Agnes Linglart[1], Francesco Emma[2], Justine Bacchetta[3], Francisco de la Cerda-Ojeda[4], Wesley Hayes[5], Moira Cheung[5], Alessandra Cocca[6], Wolfgang Högler[7], Jo Bowler[8], Lucy Henderson[8], Wei Sun[9], Alina Tudor[10], Emilia Quattrocchi[11], Raja Padidela[12,13]

[1]Paris Saclay university, AP-HP, Paris, France. [2]Bambino Gesu Children's Hospital IRCCS, Rome, Italy. [3]Hospices Civils de Lyon, Lyon, France. [4]Virgen del Rocio Universitary Hospital, Seville, Spain. [5]Great Ormond Street Hospital for Children NHS Foundation Trust, London, UK [6]Evelina London Children's Hospital, London, UK [7]Johannes Kepler University Linz, Linz, Austria. [8]Kyowa Kirin Pharmaceutical Development Ltd, Galashiels, UK [9]Kyowa Kirin Pharmaceutical Development Inc, New Jersey, USA [10]Kyowa Kirin Services Ltd, Marlow, UK [11]Kyowa Kirin Pharmaceutical Development Ltd, Basiglio, Italy. [12]Royal Manchester Children's Hospital, Manchester, UK [13]The University of Manchester, Manchester, UK

### Abstract

**Objectives:** Report safety and efficacy of burosumab from a Phase 1/2 open-label study in infants (<1 year) with XLH (NCT04188964).

**Methods:** Infants with XLH and hypophosphataemia received burosumab every 2 weeks for up to 48 weeks. There were 3 cohorts: Cohorts 1 and 2 enrolled infants aged ≥6–<12 months (starting doses: 0.4 and 0.8 mg/kg respectively); Cohort 3 enrolled infants <6 months (starting dose: 0.4 mg/kg). Primary endpoint was safety, including treatment emergent adverse event (TEAE) incidence and severity. Secondary endpoints included efficacy, measured by changes in serum phosphate, 1,25(OH)$_2$D, alkaline phosphatase (ALP), radiographic global impression of change (RGI-C) and rickets severity score (RSS).

**Results:** Cohorts 1, 2, and 3 were assigned 3, 9, and 4 participants, respectively (N=16). Mean (SD) age at baseline was 8.5 (1.5), 9.2 (1.5), and 4.8 (0.6) months, respectively. Overall, 7/16 participants (43.8%) were female and 15/16 (93.8%) completed the study; mean (SD) exposure to burosumab was 11.2 (1.2) months.

All participants reported TEAEs (Table) with no difference in overall incidence across Cohorts. Four participants experienced ≥1 treatment-related TEAE (high parathyroid hormone [n=3], craniosynostosis [n=1], dermatitis acneiform [n=1]); craniosynostosis was Grade 3 but the participant neither discontinued from treatment nor study. One participant developed anti-drug antibodies, with no impact on safety or efficacy. No TEAEs led to treatment discontinuation or death.

Mean (SD) serum phosphate was 3.1 (0.4) mg/dL at baseline (n=16) and 3.7 (0.5) mg/dL at end of treatment/early termination (EOT/ET; n=15), which was within age-adjusted normal ranges; all mean post-baseline measures were 3.6–3.7 mg/dL. Mean (SD) serum 1,25(OH)$_2$D increased from 65.2 (37.5) pg/mL at baseline (n=16) to 73.3 (16.9) pg/mL at EOT/ET (n=14). Mean (SD) serum ALP decreased by 29.5% at EOT/ET (n=15) from 713.7 (119.3) U/L at baseline (n=16). Mean (SD) global RGI-C score (wrists and knees) was 1.9 (0.4) at EOT/ET (n=15); 13 participants (86.7%) were responders. Other mean (SD) secondary endpoints (baseline, EOT/ET): RSS (4.6 [1.4, n=16], 1.9 [1.0, n=13]); head circumference (45.3 cm [2.5, n=16], 48.7 cm [2.2, n=16]).

**Conclusions:** This study confirms the safety and efficacy of burosumab in infants <1 year of age with XLH.

**Table. Summary of TEAEs**

| Category | Participants (N = 16) |
|---|---|
| **TEAEs, n (%)** | **16 (100)** |
| Mild (Grade 1) | 5 (31.3) |
| Moderate (Grade 2) | 10 (62.5) |
| Severe (Grade 3) | 1 (6.3) |
| Life threatening (Grade 4) | 0 |
| Death (Grade 5) | 0 |
| **TEAEs (>30% incidence), n (%)** | |
| Pyrexia | 12 (75.0) |
| Nasopharyngitis | 7 (43.8) |
| Rhinitis | 7 (43.8) |
| Upper respiratory tract infection | 6 (37.5) |
| Coronavirus disease 2019 (COVID-19) | 5 (31.3) |
| **TEAEs of special interest, n (%)** | **9 (56.3)** |
| Hypersensitivity | 5 (31.3) |
| Hyperparathyroidism | 4 (25.0) |
| Ectopic mineralisation | 1 (6.3) |
| Anti-drug antibodies | 1 (6.3) |
| Injection site reaction | 1 (6.3) |
| Unclassified* | 2 (12.5) |
| **Treatment-related TEAEs, n (%)** | **4 (25.0)** |
| Mild (Grade 1) | 3 (18.8) |
| Moderate (Grade 2) | 0 |
| Severe (Grade 3) | 1 (6.3) |
| Life threatening (Grade 4) | 0 |
| Death (Grade 5) | 0 |
| **Treatment-emergent SAEs, n (%)** | 2 (12.5) |
| Leading to death | 0 |
| **Treatment-related treatment emergent SAEs, n (%)** | 1 (6.3) |
| Leading to death | 0 |

*TEAEs assessed by investigators as of special interest but did not meet the criteria as defined in the statistical analysis plan, were classed as unclassified.
SAE, serious adverse event; TEAE, treatment emergent adverse event.

### Disclosure

Agnes Linglart has received consultant fees and honoraria for conferences from Kyowa Kirin, Alexion and Novo Nordisk. Francesco Emma has received honoraria for conferences from Kyowa Kirin Int. Justine Bacchetta has received speaker, consulting and speaker fees from Kyowa Kirin. Francisco de la Cerda has received honoraria or consultation fees from Kyowa Kirin and has participated in Kyowa Kirin sponsored speaker bureau. Wesley Hayes has nothing to disclose. Moira Cheung received honoraria from Kyowa Kirin for taking part in advisory boards, delivering presentations and for funding of clinical staff for the rare bone service at ELCH and GOSH. She is an investigator on trials for burosumab and the International XLH Registry. Alessandra Cocca has no COIs to disclose. Wolfgang Högler received lecture honoraria and travel support from Kyowa Kirin. Jo Bowler is an employee of Kyowa Kirin Pharmaceutical Development Ltd. Lucy Henderson is an employee of Kyowa Kirin Pharmaceutical Development Ltd. Wei Sun is an employee of Kyowa Kirin Pharmaceutical Development Inc. Alina Tudor is an employee of Kyowa Kirin Services Ltd. Emilia Quattrocchi is an employee of Kyowa Kirin Pharmaceutical Development Ltd. Raja Padidela has received research, travel grants and honorariums from Kyowa Kirin International.

### OC 6.3

### Bone metabolism and bone mass accretion in preterm infants born below 33 weeks of gestation - a case-control study

Justyna Czech-Kowalska[1], Edyta Czekuć-Kryskiewicz[1], Katarzyna Zaniuk[2], Paweł Pludowski[1], Maciej Jaworski[1]

[1]The Children's Memorial Health Institute, Warsaw, Poland. [2]The Holy Family Hospital, Warsaw, Poland

### Abstract

**Table 1.** Comparison of bone formation and resorption markers and bone mass between preterm and full-term infants at baseline (40 weeks postmenstrual age) and after 3 months observation (52 weeks postmenstrual age). (Data are presented as median and range. The statistically significant [illegible] value are ≤ 0.05.)

| Parameter | Preterm infants | Full-term infants | p-value |
|---|---|---|---|
| ***At birth*** | n=66 | n=54 | |
| Gestational age (weeks) | 30 (23.5-32) | 39 (37-41) | <0.001 |
| Birth weight (g) | 1220 (490-2240) | 3514 (2980-4280) | <0.001 |
| ***Baseline (40 weeks PMA)*** | n=66 | n=54 | |
| 25OHD (ng/ml) | 39.9 (9.1-92.1) | 15.05 (5.2-39.5) | <0.001 |
| PTH (pg/ml) | 26.5 (2.74-156) | 4.76 (1.61-26.14) | <0.001 |
| Osteocalcin (ng/ml) | 124.9 (63.02-259.7) | 67.91 (18.95-121.4) | <0.001 |
| PINP (ng/ml) | 4897 (2268-11301) | 2340 (1096-5202) | <0.001 |
| CTx (ng/ml) | 0.847 (0.547-1.44) | 0.659 (0.491-1.57) | <0.001 |
| NT-proCNP (pmol/l) | 43.294 (9.075-100.7) | 42.346 (0.047-79.48) | 0.396 |
| TB BMD | 0.252 (0.159-0.38) | 0.352 (0.274-0.51) | <0.001 |
| TB BMC | 49.75 (28.2-73.9) | 94 (70-132) | <0.001 |
| Area total | 197.85 (138.24-293.15) | 257 (216-319) | <0.001 |
| BMD Index total | 0.006 (0.003-0.009) | 0.007 (0.005-0.010) | <0.001 |
| ***Follow-up (52weeks PMA)*** | n=48 | n=54 | |
| 25OHD (ng/ml) | 33.5 (11.7-138) | 34.75 (20.1-61.7) | 0.565 |
| PTH (pg/ml) | 18.64 (5.07-205) | 17.98 (6.75-33.57) | 0.263 |
| Osteocalcin (ng/ml) | 108.95 (61.09-239.6) | 146.5 (72.75-288) | <0.001 |
| PINP (ng/ml) | 2570 (1494-5967) | 2299 (1141-3805) | 0.001 |
| CTx (ng/ml) | 0.758 (0.45-1.37) | 0.956 (0.71-1.51) | <0.001 |
| NT-proCNP (pmol/l) | 31.103 (9.666-65.59) | 20.069 (4.328-93.5) | <0.001 |
| TB BMD | 0.418 (0,292-0,54) | 0,478 (0,395-0,55) | <0,001 |
| TB BMC | 88,3 (38,3-156,8) | 109 (83-166) | <0,001 |
| Area total | 217,65 (121,59-302,23) | 230,5 (198-300) | 0,002 |
| BMD Index total | 0,009 (0,005-0,012) | 0,010 (0,007-0,013) | <0,001 |
| ***Relative change (%)*** | | | |
| 25OHD | -11.81 (-66.88-454.2) | 141.4 (-18.95-569.2) | <0.001 |
| PTH | -30.68 (-89.04-2343) | 270.3 (-13.89-1097) | <0.001 |
| Osteocalcin(ng/ml) | -7.699 (-58.825-151.19) | 103.296 (-18.069-849.87) | <0.001 |
| PINP (ng/ml) | -47.87 (-71.79-23.44) | 2.462 (-59.409-159.45) | <0.001 |
| CTx (ng/ml) | -18.288 (-62.5-68.72) | 52.615 (-41.019-137.25) | <0.001 |
| NT-proCNP (pmol/l) | -32.278 (-85.14-332.34) | -51.017 (-90.158-52873.32) | 0.009 |
| TB BMD | 58,759 (12,158-140,84) | 30.089 (1.98-80.66) | <0.001 |
| TB BMC | 81.109 (3.218-219.5) | 17.196 (-2.913-42.86) | <0.001 |
| Bone Area total | 12.10 (-27.43-67.55) | -9.69 (-34.64-19.28) | <0.001 |
| BMD Index total | 47.06 (-8.83-182.19) | 38.42 (-7.33-143.88) | 0.809 |

OC, osteocalcin; PINP, procollagen type 1 N-terminal propeptide; CTx, carboxyterminal cross-linked telopeptide of type 1 collagen; NT-proCNP, amino-terminal CNP; PMA, postmenstrual age; TB BMD, total body bone mineral density; TB BMC, total body bone mineral content; BMD Index, bone mineral density index;

OBJECTIVE:

Biochemical markers of bone resorption and formation can provide an idea of the mechanical effects of tactile and kinesthetic stimulation on bone development. In contrast, DXA measurements provide a static picture of bone mass. The assessment of bone turnover markers might be a vital methodological advance in clinical practice. We aimed to asses differences in the dynamics of bone mass development and bone metabolism in preterm infants and full-term infants."

METHODS:

We prospectively assessed 66 Caucasian preterm infants (PT) born <33 weeks of gestation and 54 full-term (FT) healthy singleton infants (control group). Total body bone mineral content (BMC), bone area (BA), bone mineral density (BMD) were measured by DXA (Lunar Prodigy Advance**)** at 40 and 52 weeks postmenstrual age (PMA). Bone formation (osteocalcin; OC, procollagen type 1 N-terminal propeptide; PINP), bone resorption (carboxyterminal cross-linked telopeptide of type 1 collagen; CTx), and growth plate activity (amino-terminal CNP; NT-proCNP) serum markers and 25OHD level were also measured twice.

RESULTS:

All DXA parameters were significantly lower in PT infants than FT infants at 40 and 52 weeks PMA. Analysis of variance ANOVA with repeated measures showed that all DXA parameters beside BMD Index Total had more pronounced increase in PT than FT infants during 3 months observation. Nevertheless, PT infants had still lower TB BMD, TB BMC, Area Total i BMD Index Total than FT infants. All bone formation (OC, PINP) and resorption (CTx) markers were significantly higher in PT infants than FT infants at 40 weeks PMA, while NT-proCNP was comparable between groups. After 3 months of observation PT infants had higher PINP and NT-proCNP concentrations but lower OC and CTx concentrations than FT infants (table 1).

CONCLUSIONS:

Preterm infants exhibit increased bone mass accretion accompanied by higher bone turnover compared to full-term infants. However, deficits in bone mass persist at least up to 52 weeks postmenstrual age despite observed accelerated growth plate activity and bone formation.

### AHP Oral Communication Presentation Abstracts

### P4

### Highly specialised service for severe, complex and atypical osteogenesis imperfecta in England: Real world data in a national cohort

Vrinda Saraff[1], Christine Burren[2], Meena Balasubramanian[3], Stephanie A Borg[3], Jeremy Allgrove[4], Alistair Calder[4], Belinda Crowe[4], Toby Candler[2], Nicola Crabtree[1], Amaka C Offiah[3,5], Suma Uday[1], Catherine Devile[4], Paul Arundel[3] On behalf of the NHS England Highly Specialised Childhood Complex Osteogenesis Imperfecta Service[6]

[1]Birmingham Women's and Children's Hospital, Birmingham, UK [2]University Hospitals Bristol and Weston NHS Foundation Trust, Bristol, UK [3]Sheffield Children's Hospital NHS Foundation Trust, Sheffield, UK [4]Great Ormond Street Hospital for Children NHS Foundation Trust, London, UK [5]Division of Clinical Medicine, University of Sheffield, Sheffield, UK [6]NHS England Highly Specialised Services, London, UK

### Abstract

**Objectives:** Osteogenesis imperfecta (OI) is a rare genetic disorder associated with bone fragility with an incidence of 1:10,000-20,000 live births. The nationally commissioned highly specialised OI service is delivered through 4 OI centres in England (Birmingham, Bristol, London, and Sheffield) providing a multidisciplinary approach to the diagnosis and management of those aged 0–16 years with Severe, Complex, and Atypical (SCA) OI. Given the structure of the service, all children with SCA OI in England sit under the care of the 4 centres, and our objective is to describe this cohort of children.

**Methods:** Data were prospectively collected on children with SCA OI managed by the 4 specialist centres for a 12-month period in the financial year 2022-23. Severe OI included neonates with multiple long bone fractures and children with six or more vertebral fractures. Complex OI encompassed children with cervical spine abnormalities, basilar invagination, and progressive scoliosis. Atypical OI included children with autosomal recessive forms of OI. Data were collected for demographics, OI type, cervical spine abnormalities, scoliosis, long bone and vertebral fractures, and physio and occupational therapy (POT) sessions.

**Results:** Data from 314 children with SCA OI in England were analysed. Of these, 102 (32%) had type 1, 68 (22%) type IV, 59 (19%) type III and 85 (27%) “Atypical” OI. Cervical spine malformations with risk to cerebrospinal fluid flow was noted in 7 (2%), 12 (4%) had basilar invagination. Scoliosis with Cobb angle >45 degrees reported in 25 (8%) and 19 (6%) had undergone spinal fusion.  Confirmed non-vertebral fractures reported in 105 (33%) with a mean of 0.67 fractures per patient. Prevalent vertebral fractures noted in 207 (66%). Mean numbers of vertebral fractures per patient in the service with 10-25% and >25% wedging were 1.97 and 0.95, respectively. Of the 963 follow up POT sessions delivered to these children in the 12-month period, 121 of them were more prolonged “intensive” sessions.

**Conclusion:** We describe a complete national cohort of children with Severe, Complex, and Atypical OI, highlighting both the disease burden and our experience of delivering an integrated multidisciplinary service for this large and complex group.

### P111

### Robotic walking for bone and muscle health in youth with cerebral palsy

Erin Hodgson[1,2,3,4], Elizabeth G Condliffe[1,2,4,5,6], Leigh Gabel[1,2,3,4]

[1]Biomedical Engineering Graduate Program, University of Calgary, Calgary, Canada. [2]Faculty of Kinesiology, University of Calgary, Calgary, Canada. [3]McCaig Institute for Bone and Joint Health, University of Calgary, Calgary, Canada. [4]Alberta Children's Hospital Research Institute, University of Calgary, Calgary, Canada. [5]Department of Clinical Neurosciences and Pediatrics, University of Calgary, Calgary, Canada. [6]Hotchkiss Brain Institute, University of Calgary, Calgary, Canada

### Abstract

**Objectives**: Cerebral Palsy (CP) is the most prevalent childhood motor disability affecting ~2-3/1000 births. Weight-bearing physical activity is essential for developing a strong skeleton; however, ~1/3 of children with CP cannot walk independently, compromising bone health. As such, research into novel robotic trainers provides a promising alternative for children with mobility impairments. Robotic gait trainers offer partial weight-bearing and independence while providing support and stability. Therefore, the objective of this study is to assess the effect of a 3 month walking intervention with a robotic walker on bone and muscle strength in non-ambulatory youth with CP.

**Methods**: This is an ongoing study in 19 participants with CP who are unable to ambulate independently. Participants aim to obtain ≥30 mins/day for ≥5 days/week of robotic walking in a Trexo trainer for 3 months. Peripheral quantitative computed tomography (pQCT) imaging of the tibiae was acquired at the 3%, 38%, and 66% sites prior to and following the intervention on 11 participants. Seven participants were excluded due to motion artifact and one is currently training. Data were converted to age-, sex-, and ethnicity matched z-scores based on a typically developing population to differentiate between typical growth and interventional effects.

**Results**: Currently, three participants have completed baseline and follow-up scans (4.9M, 6.5M, and 9.5F years, respectively). At baseline, trabecular BMD (Tb.BMD) at the 3% site was significantly lower than typically developing children for all participants (-5.94 to -0.31 z-score). Tb.BMD z-scores increased by 9-28% over the trial, such that follow-up z-scores ranged from -5.37 to -0.28. One participant had valid baseline and follow-up scans at the 38% site indicating a 28% increase in Ct.BMD z-score from 1.34 to 1.72 at follow-up and their Ct.BMC z-score increased by 5% (-3.67 to -3.49) over the trial. The MCSA z-score from one participant at the 66% tibia site improved by 1.0% (-4.21 to -4.16) and muscle density increased by 2.7% (1.99 mg/mm$^3$) over the trial.

**Conclusions**: Preliminary results showing improvements in bone and muscle measures are encouraging and suggest the robotic walker may improve bone and muscle health in this population. Continuation of the study with additional participants will solidify conclusions.

### P131

### The End of Waddling? Surgical knee varus correction decreases lateral trunk lean in children and adults with XLH

Celine Akta[1,2], Andreas Kranzl[1,2], Catharina Chiari[1,2], Alexandra Stauffer[1,2], Adalbert Raimann[3,2], Roland Kocijan[4,2], Gabriel Mindler[1,2]

[1]Orthopedic Hospital Speising, Vienna, Austria. [2]Vienna Bone and Growth Center, Vienna, Austria. [3]Medical University Vienna, Vienna, Austria. [4]Hanusch Hospital, Vienna, Austria

### Abstract

**Objectives:** X-linked Hypophosphatemia (XLH, OMIM 307800) is a rare genetic bone disorder characterized by bone deformities, early osteoarthritis, enthesopathies, pain and reduced quality of life. Disease specific gait deviations with increased lateral trunk lean ("Waddling gait") have been associated with knee varus deformity and add significantly to the burden of disease. This study analysis changes of trunk movement after genu varum surgery in children and adults with XLH.

**Methods:** This retrospective study examined patients with XLH who underwent surgical lower limb varus deformity correction at our institute. Radiographic and gait analysis data was analyzed before and after the surgery. Radiographic analysis of anteroposterior long leg standing x-rays included measurement of mechanical axis deviation and standardized deformity angles.

**Results:** Eight female and four male patients (21 limbs) with a mean age of 20.1 years (5-50, SD: 12.9 years) were eligible for the study. The mean follow up was 20 months; included correction procedures: hemiepiphysiodesis (5 limbs), nails (2 limbs), circular external fixators (10 limbs), four limbs were treated with a combination of procedures (hemiepiphysiodesis, osteotomies). Patients with XLH undergoing lower limb varus correction showed significantly reduced mechanical axis deviation (MAD) postoperatively ($p < 0.001$). Lateral thorax range of motion decreased after surgical lower limb intervention ($p < 0.001$), however, still exceeding the normal range. Further analysis indicates that acute correction (nails) had less improvement of thorax movement than gradual correction techniques (hemiepiphysiodesis, external fixator).

**Conclusions:** This is the first study to assess therapeutic strategies for increased lateral trunk lean. Patients with XLH undergoing varus deformity correction of the lower limbs showed significantly reduced lateral trunk lean. However, normal thorax movement was not achieved in all cases. Further analysis of surgical techniques and co-factors with bigger cohorts are necessary to evaluate this disease specific gait.

### Disclosure

AR, RC, GM received non-related honoraria from Kyowa Kirin and Alexion for consultancy and scientific presentations. GM received non-related honoraria from Biomarin for consultancy and scientific presentations.

### P144

### Preparing adults with osteogenesis imperfecta to engage in research on access and quality of care for their rare disorder

Laura Tosi[1], Erika Carter[2], Stacie Connors[2], Michael Stewart[2], Andrew Pennington[1], Kendall Reid[1], Tracy Hart[3]

[1]Children's National Hospital, Washington, USA [2]Osteogenesis Imperfecta Foundation, Gaithersburg, USA [3]Osteogenesis Imperfecta Foundation, Washington, USA

### Abstract

### Background:

While most children with osteogenesis imperfecta (OI) in the US are cared for at one of 57 pediatric OI clinics, there are only 5 adult clinics. Thus, adult OI patients seeking specialized OI care face major challenges 1) transitioning to adult care and 2) gaining access to sites with the capacity to provide appropriate care. Excellent research has been done on the broad issues of transitioning to adult care, but virtually all publications assume that clinicians and systems are prepared to take on care responsibilities for all disorders. The problem for patients with OI is that, in general, there is a dirth of healthcare resources available to meet their unique needs.

### Methods:

The three aims of this project included 1)preparation of OI community members/stakeholders for participation in patient engagement, 2) utilization of patient-centered outcomes research to identify gaps and opportunities in adult OI care, and 3) creation of a strategic plan for leaders of the OI Foundation (OIF) to build on these findings. The project included five phases: 1) establishment of Patient and Scientific Advisory Boards, 2) development of scripts for and conducting listening sessions with volunteers from the OI adult and parent/caregiver community, health care providers and health policy leaders, 3) summarizing findings, 4) presenting findings to key audiences, and 5) developing resources dedicated to bridging identified gaps.

### Results:

The immediate impact of this project was creation of the OI Adult Health Toolkit: Information for Adults Living with OI, Their Families, and Medical Professionals, to help meet identified gaps. Second, the project increased the commitment of the OIF to develop evidence-based data resources on adult OI healthcare.

### Conclusion:

The leadership team recognized that progress in improving care for adults with OI cannot be achieved unless progress is also made to support the greater rare disease community .

### P156

### The collaborative production of "The Hospital of Unhappy Surprises"

Yi Wen Wang[1], Frank Rauch[1,2] Kelly Thorstad[2] Franco Carnevale[1,2] Stephanie Smith[2] Argerie Tsimicalis[1,2]

[1]McGill University, Montreal, Canada. [2]Shriners Hospitals for Children-Canada, Montreal, Canada

### Abstract

**OBJECTIVES:**

Osteogenesis Imperfecta (OI) is a rare genetic bone disorder causing connective tissue weakening, bone deformities, and pain. OI remains largely unknown to healthcare providers, causing significant ethical concerns. Children from Shriners Hospitals for Children-Canada created a play called “The Hospital of Unhappy Surprises” detailing “bad” hospital environments and their vision for an ideal practice. However, the play captured a small subset of OI-affected children’s experiences. The objective was to understand the hospital experiences of OI-affected children *globally* to create a professionally animated, open-access play.

**METHODS:**

Ethno-dramatic storytelling was used to elicit children’s voices and transform oppressive scenarios into liberating ones. OI-affected children participated in semi-structured, online interviews. Children were shown a preliminary staging of the play, invited to discuss their experiences and act out scenes with print-out puppets, permitting the fictional portrayal of children’s lived experiences.

**RESULTS:**

Seventeen children (ages 4-18) participated from the United States, Canada, and Europe. The play reminded them of their own experiences, facilitating discussions with researchers. Children reported experiencing fear, frustration, and difficulties engaging in their OI care due to complex medical terminology, lack of information and resources about OI, and clinicians’ lack of OI knowledge. Children experienced elevated anxiety during x-rays and unexpected hospitalizations when pain control and communication were lacking.

**CONCLUSIONS:**

This study represents the push needed to: (a) understand the experiences of OI-affected children via alternative means of engagement (e.g., play, art); (b) produce educational resources that align with children’s needs; and (c) support clinician’s abilities to meet children’s needs.

### P168

### Pain and its biopsychosocial impact in children with skeletal dysplasias

Matthew Boston[1], Saunya Dover[1], Joanna Lazier[1], Sasha Carsen[1], Kevin Smit[1], Leanne M Ward[1], Eve Blanchard[1], Mary-Ann Matzinger[1], Nazih Shenouda[1], Khaldoun Koujok[1], Adam Khalif[1], Frank Rauch[2], Marie-Eve Robinson[1]

[1]Children's Hospital of Eastern Ontario, Ottawa, Canada. [2]Shriners Hospital for Children, Montreal, Canada

### Abstract

**Objectives:** To describe the pain experienced by children with skeletal dysplasias and to determine which biopsychosocial outcomes are associated with pain.

**Methods:** Participants aged 0-18 years with a confirmed or suspected skeletal dysplasia were recruited from the multidisciplinary clinic for children with skeletal dysplasias at the Children's Hospital of Eastern Ontario in Ottawa, Canada. Parents/caregivers completed a series of validated questionnaires during a routine clinic visit to evaluate their child's pain (current pain and worst pain in the previous week as assessed by a 10 cm visual analog scale on the Pediatric Pain Questionnaire), health-related quality of life (HRQoL), fatigue, pain interference, symptoms of depression and anxiety, sleep, peer relationships, and physical function. As part of the physical function assessment, participants aged ≥6 years also completed single a two-legged jump (to assess lower body muscle power). We summarized our cohort and outcomes using descriptive statistics (mean/standard deviation [SD] or median/rage as appropriate) and tested for associations using Pearson's correlations.

**Results:** Thirty-two participants (41% female) with a mean age of 11.8 (SD 4.1) years were included. Diagnoses included achondroplasia (9/32; 28%), osteogenesis imperfecta (type I, III or IV, 5/32; 16%), juvenile osteoporosis (3/32; 9%), and other skeletal dysplasias (15/32, 47%). Twenty (63%) parents/caregivers reported that their child experiences pain. Of those, median current pain and worst pain in the previous week levels were 2.1 (range 0.2-9.8) and 5.0 (range 0.2-100), respectively. The top 5 biopsychosocial outcomes associated with current pain were pain interference (r=0.73, p<0.0001), HRQoL (r=-0.56, p<0.0001), peer relationships (r=-0.51, p=0.004), fatigue (r=0.45, p=0.01), and depressive symptoms (r=0.38, p=0.03). The top 5 biopsychosocial outcomes associated with worst pain the previous week were pain interference (r=0.72, p<0.0001), HRQoL (r=-0.65, p<0.0001), fatigue (r=0.53, p=0.002), peer relationships (r=-0.52, p=0.002), and weight-adjusted single 2-legged jump score (r=-0.49, p=0.05).

**Conclusion:** Pain is prevalent in children with skeletal dysplasias and is associated with increased pain interference, fatigue and depressive symptoms, as well as decreased HRQoL, peer relationships and muscle power. Those biopsychosocial outcomes should be further explored to determine appropriate targets for future interventions to help prevent and treat pain in children with skeletal dysplasias.

### Disclosure

Leanne Ward reports study grants to institution from Ascendis, Catabasis, PTC Therapeutics, ReveraGen, Ultragenyx and Amgen, and consultancy fees to institution from Ipsen, Santhera, Amgen, Alexion and Ultragenyx. Frank Rauch reports study grants to institution from PreciThera Inc, Ultragenyx Inc and

Catabasis. Marie-Eve Robinson declares study grants to institution from Ascendis Biopharma, QED therapeutics, and Ipsen Biopharmaceuticals and consultancy fees to institution from Ipsen Biopharmaceuticals and Ultragenyx.

### Poster Abstracts

### P1

### Association between parental and offspring DXA and pQCT derived bone health parameters

Sharvani Patil[1], Anuradha Khadilkar[1,2], Neha Kajale[1,2], Vivek Patwardhan[1], Chirantap Oza[1], Shruti Mondkar[1], Chidvilas More[1]

[1]Hirabai Cowasji Jehangir Medical Research Institute, Jehangir Hospital, Pune, India. [2]School of Health Sciences Savitribai Phule Pune University, Pune, India

### Abstract

Objectives:

It's crucial to understand influence of parental bone health on early bone development of children to identify children at risk of poor bone health. Our objectives were 1] To study association between bone health parameters as measured by Dual Energy X ray Absorptiometry (DXA ) and peripheral Quantitative Computed Tomography (pQCT) in prepubertal rural Indian children and their parents.2] To identify inherited SNPs located in genes that code for proteins associated with bone health(subset).

Methods:

In this cross-sectional observational study, 768 children (403 boys,8-10yrs,2304 parents) were studied. Bone density and geometry measured using GE-Lunar iDXA (encore software – version 16) and pQCT (XCT-2000 scanner, Stratec, Inc; Pforzheim, Germany) respectively. Genome Wide Association Study(GWAS) was performed on a subset of 60 children and 120 parents to identify inherited SNPs that influence bone health parameters using the Precision Medicine Research Array. R package was used for computation and checking for the association of identified SNPs with the phenotype. General Linear Model(GLM), Mixed Linear Model(MLM) and Fixed and random model Circulating Probability Unification (FarmCPU) models were used.

Result:

Children's bone densitometry and geometry were significantly associated with parental bone health(Table). For boys, $L_1$-$L_4$ BMD/BMC showed strong and moderate association with both parents (R=0.53, $p$<0.001) (R=0.41, $p$<0.001) respectively. Trabecular density in boys showed moderate correlation with the father (R=0.34, $p$<0.001) as compared to weak association with the mother (0.229, $p$<0.001). In girls, $L_1$-$L_4$ BMD/BMC showed strong and moderate association with both parents (R>0.56, $p$<0.001) (R>0.49, $p$<0.001) respectively. Also, girls' cortical density and thickness showed moderate association with their mother (R=0.49, $p$<0.001) (R=0.313, $p$<0.001) and weak association with their father (R=0.25, $p$<0.001) (R=0.200, $p$<0.001). GWAS found 48 significant ($p$<10−5) intergenerational SNPs . SNP rs56180979 (β= 3.04 $\times 10^{12}$ SE=3.22$\times 0^{11}$ $p$=1.64 $\times 10^{-17}$) in gene *HS1BP3,* SNP rs143051802 ( β= 2.7$\times 10^{12}$ SE= 2.78$\times 10^{11}$ $p$= 2.08$\times 10^{-18}$) in gene *SLC16A2* on X chromosome coding for proteins responsible for transporting calcium and other minerals respectively; these have been reported to be associated with increased osteoporosis risk.

Conclusion: Association between parental and children's bone parameters was observed in rural Indian population. Genomic insight revealed inherited SNPs associated with high risk of osteoporosis.

| Parents | Bone Health Parameters | Father | | Mother | |
|---|---|---|---|---|---|
| | | r value | *p* value | r value | *p* value |
| Boys | TBLH BMD | 0.276** | 0.001 | 0.241** | <0.001 |
| | TBLH BMC | 0.171** | 0.001 | 0.216** | <0.001 |
| | $L_1$-$L_4$ BMD | 0.531** | <0.001 | 0.559** | <0.001 |
| | $L_1$-$L_4$ BMC | 0.418** | <0.001 | 0.470** | <0.001 |
| | Cortical density | 0.201** | 0.001 | 0.184** | 0.003 |
| | Cortical thickness | 0.249** | <0.001 | 0.229** | <0.001 |
| | Trabecular density | 0.340** | <0.001 | 0.261** | <0.001 |
| | Endosteal circumference | 0.292** | <0.001 | 0.279** | <0.001 |
| | Periosteal circumference | 0.277** | <0.001 | 0.292** | <0.001 |
| | Stress-Strain Index | 0.166** | 0.008 | 0.293** | <0.001 |
| Girls | TBLH BMD | 0.373** | <0.001 | 0.398** | <0.001 |
| | TBLH BMC | 0.336** | <0.001 | 0.328** | <0.001 |
| | $L_1$-$L_4$ BMD | 0.569** | <0.001 | 0.582** | <0.001 |
| | $L_1$-$L_4$ BMC | 0.490** | <0.001 | 0.496** | <0.001 |
| | Cortical density | 0.259** | <0.001 | 0.402** | <0.001 |
| | Cortical thickness | 0.200** | 0.007 | 0.313** | <0.001 |
| | Trabecular density | 0.286** | <0.001 | 0.277** | <0.001 |
| | Endosteal circumference | 0.276** | <0.001 | 0.311** | <0.001 |
| | Periosteal circumference | 0.291** | <0.001 | 0.226** | <0.001 |

**. Correlation is significant at the 0.01 level.

### P2

### The impact of birth weight on bone mineral density in a longitudinal Irish birth cohort

Laura Lennon[1], Ciara Mc Donnell[2]

[1]Children's Health Ireland, Crumlin, Dublin, Ireland. [2]Children's Health Ireland, Dublin, Ireland

### Abstract

**Objective:**

Bone health is of great importance during both childhood and adolescence and is predictive of bone density into adulthood. Bone mineral density (BMD) and bone mineral content (BMC) are two measurements of bone health assessed using dual-energy x-ray absorptiometry (DXA). We want to know more about factors, both genetic and environmental, which influence bone health. We are particularly interested in the effect of birth weight on bone health. The aim of this study is to examine the influence of birth weight on BMD and BMC in a longitudinal Irish birth cohort at 5 years of age and again at 9 to 11 years of age.

**Methods:**

Secondary analysis of data of the mother-child pairs from the ROLO Study cohort group where the child has completed a DXA scan at 5 years (timepoint one) and/or 9 to 11 years of age (timepoint two). We will examine these relationships firstly among the whole study group, and secondly among the subgroup of those children who had DXA at both timepoints.

**Results:**

There are significant positive correlations, between birth weight and BMD, and birth weight and BMC at timepoint one. All correlations decrease by timepoint two in both the total population and the subgroup. After adjusting for confounding variables, the predictive value of birth weight for both BMD and BMC is of statistical significance at timepoint two only.

**Conclusions:**

Birth weight is not predictive of BMD or BMC in early childhood, however birth weight is predictive of BMD and BMC in the preteen years. This study supports evidence that the intra-uterine environment, as reflected by birth weight, is an important period for programming of bone health particularly in the pre and early adolescent years. The relationship between birth weight and bone health in early childhood may be mediated by other factors such as growth.

### P3

### Investigating the association between maternal smoking and offspring bone mass in the Southampton Women's Survey (SWS)

Snigdha Harish[1,2], Rebecca Moon[1,3], Hazel Inskip[1], Keith Godfrey[1,4], Nicholas Harvey[1,4], Cyrus Cooper[1,4,5], Sarah Crozier[1,4,6], Janis Baird[1,4,6]

[1]MRC Lifecourse Epidemiology Centre, Southampton, UK [2]University of Southampton, Southampton, UK [3]University Hospital Southampton NHS Foundation Trust, Southampton, UK [4]NIHR Southampton Biomedical Research Centre, Southampton, UK [5]NIHR Biomedical Research Centre, Oxford, UK [6]NIHR Applied Research Collaboration Wessex, Southampton, UK

### Abstract

### Objectives

Smoking in pregnancy has been associated with increased offspring fracture risk, yet the reported relationships with bone mineral density (BMD) are inconsistent. We investigated the associations between smoking before and during pregnancy with offspring dual-energy X-ray absorptiometry (DXA) measured bone outcomes (bone area [BA], bone mineral content [BMC], bone mineral density [BMD] and bone mineral apparent density [BMAD]) during early childhood.

### Methods

In the Southampton Women's Survey, pre-pregnancy, early (11-14 weeks') and late pregnancy (34 weeks') smoking were determined prospectively by interviewer-led questionnaires. Offspring DXA was undertaken at birth (whole body; Lunar DPX-L) and 4,6-7 and 8-9 years (whole-body-less-head; Hologic Discovery). Associations between maternal smoking and standardised bone outcomes were assessed using linear regression. Models were adjusted for maternal education, diet, ethnicity, age at delivery and parity (confounders identified by directed acyclic graph), and offspring age and sex (competing exposures). In further models, offspring weight was included to assess whether it mediated the relationships.

### Results

1,966 mother-child pairs were included. 25.7% of the women smoked before pregnancy, 15.9% in early pregnancy and 15.1% in late pregnancy. Pre-pregnancy smoking was positively associated with offspring BMD at 4, 6-7 and 8-9 years (Figure 1) but not at birth. Associations were similar for BMAD, but not BA or BMC. Smoking in early or late pregnancy were similarly associated with BMD at 4, 6-7 and 8-9 years, but not birth. Offspring of mothers who smoked during pregnancy were lighter at birth (β= -0.17kg, SD 0.05, 95% CI -0.26, -0.08) but heavier at 4, 6-7 and 8-9 years (8-9 years: β= 2.68kg, SD 0.64, 95% CI 1.42, 3.94). The associations between maternal smoking and offspring bone outcomes weakened when including offspring weight in the models (example shown in Figure 1).

### Conclusions

Maternal smoking before or during pregnancy was positively associated with offspring BMD. This relationship appeared to be partly mediated by higher weight in childhood. The relative undermineralisation of bone for weight may account for the reported increased fracture risk in this group of children.

Figure 1: Effect of maternal smoking before pregnancy on offspring BMD

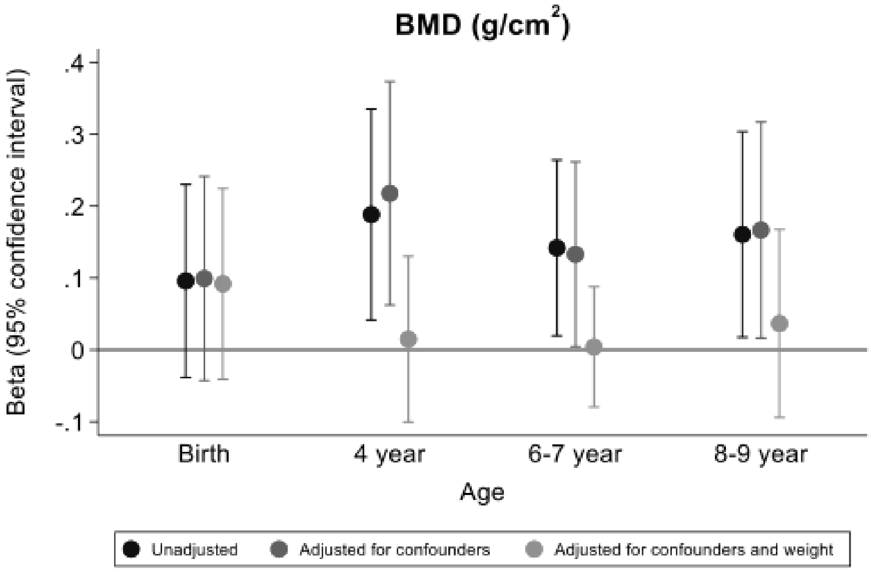

### P4

### Highly specialised service for severe, complex and atypical osteogenesis imperfecta in England: Real world data in a national cohort

Vrinda Saraff[1], Christine Burren[2], Meena Balasubramanian[3], Stephanie A Borg[3], Jeremy Allgrove[4], Alistair Calder[4], Belinda Crowe[4], Toby Candler[2], Nicola Crabtree[1], Amaka C Offiah[3,5], Suma Uday[1], Catherine Devile[4], Paul Arundel[3] On behalf of the NHS England Highly Specialised Childhood Complex Osteogenesis Imperfecta Service[6]

[1]Birmingham Women's and Children's Hospital, Birmingham, UK [2]University Hospitals Bristol and Weston NHS Foundation Trust, Bristol, UK [3]Sheffield Children's Hospital NHS Foundation Trust, Sheffield, UK [4]Great Ormond Street Hospital for Children NHS Foundation Trust, London, UK [5]Division of Clinical Medicine, University of Sheffield, Sheffield, UK [6]NHS England Highly Specialised Services, London, UK

### Abstract

**Objectives:** Osteogenesis imperfecta (OI) is a rare genetic disorder associated with bone fragility with an incidence of 1:10,000-20,000 live births. The nationally commissioned highly specialised OI service is delivered through 4 OI centres in England (Birmingham, Bristol, London, and Sheffield) providing a multidisciplinary approach to the diagnosis and management of those aged 0–16 years with Severe, Complex, and Atypical (SCA) OI. Given the structure of the service, all children with SCA OI in England sit under the care of the 4 centres, and our objective is to describe this cohort of children.

**Methods:** Data were prospectively collected on children with SCA OI managed by the 4 specialist centres for a 12-month period in the financial year 2022-23. Severe OI included neonates with multiple long bone fractures and children with six or more vertebral fractures. Complex OI encompassed children with cervical spine abnormalities, basilar invagination, and progressive scoliosis. Atypical OI included children with autosomal recessive forms of OI. Data were collected for demographics, OI type, cervical spine abnormalities, scoliosis, long bone and vertebral fractures, and physio and occupational therapy (POT) sessions.

**Results:** Data from 314 children with SCA OI in England were analysed. Of these, 102 (32%) had type 1, 68 (22%) type IV, 59 (19%) type III and 85 (27%) "Atypical" OI. Cervical spine malformations with risk to cerebrospinal fluid flow was noted in 7 (2%), 12 (4%) had basilar invagination. Scoliosis with Cobb angle >45 degrees reported in 25 (8%) and 19 (6%) had undergone spinal fusion. Confirmed non-vertebral fractures reported in 105 (33%) with a mean of 0.67 fractures per patient. Prevalent vertebral fractures noted in 207 (66%). Mean numbers of vertebral fractures per patient in the service with 10-25% and >25% wedging were 1.97 and 0.95, respectively. Of the 963 follow up POT sessions delivered to these children in the 12-month period, 121 of them were more prolonged "intensive" sessions.

**Conclusion:** We describe a complete national cohort of children with Severe, Complex, and Atypical OI, highlighting both the disease burden and our experience of delivering an integrated multidisciplinary service for this large and complex group.

**P5**

Withdrawn

### P6

### Fracture risk among children and adolescents with diabetes mellitus: A nationwide cohort study

Galia Zacay[1,2], Hagit Gabay[1], Liana Tripto-Shkolnik[1,2,3], Noah Gruber[2,4], Dalit Modan-Moses[1,2,4], Yael Levy-Shraga[1,2,4]

[1]Meuhedet Health Services, Tel Aviv, Israel. [2]Sackler School of Medicine, University of Tel Aviv, Tel Aviv, Israel. [3]Division of Endocrinology, Diabetes and Metabolism, Sheba Medical Center, Ramat Gan, Israel. [4]Pediatric Endocrinology and Diabetes Unit, The Edmond and Lily Safra Children's Hospital, Sheba Medical Center, Ramat Gan, Israel

### Abstract

**Background:** Impaired bone health is a known complication of diabetes mellitus (DM). Fracture risk is 2-5 fold higher among adults with DM. Data regarding fracture risk among paediatric patients are still sparse and inconclusive. We aimed to assess fracture risk among paediatric patients with DM compared with a matched comparison group.

**Methods:** This registry-based cohort study included individuals who were diagnosed with DM between 2001-2020, at the age of 1-17 years. DM was identified according to the International Classification of Diseases, Ninth Revision (ICD-9) code 250. A comparison group was matched in a 5:1 ratio by age, sex, population sector and socioeconomic status. The date of DM diagnosis was used as the index-date for both groups. Clinical, laboratory and demographic data were obtained from the electronic database of Meuhedet Health Services. All fracture events since the index-date until the age of 18 or the study census date (August 31, 2023) were identified by coded diagnoses.

**Results:** The DM group included 1049 individuals (52% males). The median age at DM diagnosis was 10.9 years (interquartile range (IQR) 7.5-13.4). Median HbA1c at diagnosis was 9.7 mg% (IQR 8.1-11.7). The comparison group included 5245 matched individuals. The median follow-up period of both groups was 5.5 years (IQR 3.6-8.2). We did not find a significant risk for a fracture among children with DM (adjusted hazard ratio (aHR) 1.10 for the DM group, 95% CI 0.93-1.31, p=0.25). There was increased aHR for children with celiac disease 1.46 (95% CI 1.10-1.93, p=0.009). Subgroup analysis of boys aged >11 years at DM diagnosis showed aHR of 1.47 (95% CI 1.06-2.04, p=0.02) for children with DM compared with the matched group. A multivariate analysis demonstrated that male gender (HR 2.40, 95%CI 1.36-4.25, p=0.002), and recurrent hospitalizations (HR 1.76 95%CI 1.03-3.01, p=0.04) were associated with increased risks for fractures among children with DM.

**Conclusions**: We found increased fracture risk among boys aged >11 years with DM compared to a matched comparison group. Among the DM group, male gender and recurrent hospitalization were associated with increased risks for fractures.

### P7

### Chronic Glucocorticoid exposure dephosphorylates NF-κB Inhibitor Alpha (IκBα) in the canonical NF-κB Pathway, reducing PDGF-BB transcription and skeletal angiogenesis

Sisir Kumar Barik, Weixin Zhang, Arryn Otte, Janet Crane

Johns Hopkins University, Baltimore, USA

### Abstract

Prolonged glucocorticoid (GC) use can lead to osteoporosis and osteonecrosis with the common pathophysiology of decreased bone vasculature. Using a young GC-induced osteotoxic (GIO) mouse (C57BL/6J) model, we previously reported that GCs decrease skeletal angiogenesis by decreasing platelet-derived growth factor–BB (PDGF-BB) via the nuclear factor kappa B (NF-κB) signaling pathway. PDGF-BB secretion from osteoclasts supports skeletal angiogenesis by inducing migration of endothelial precursor cells.

Objectives: We further delineated the NF-κB signaling pathway to identify potential therapeutic molecular targets while analyzing the changes in Type-H&L blood vessel volume, length, diameter and sphericity in young GIO wild type (WT) mice upon chronic GC treatment.

Method: Young WT mice were injected with methylprednisolone (MPS) (20 mg/m2/day) or vehicle (Veh) intraperitoneally daily for 2, 4, or 6 weeks (n=5-8/group/time point) beginning at postnatal day 14. Bone and vessel phenotype were analyzed by micro-CT followed by 3D vessel mapping of co-immunostained CD31hi Emcnhi using Imaris. Microarrays were used for angiogenesis-related proteome profiling and detection of NF-kB-phospho antibodies.

Results: Bone and vessel phenotype showed significantly reduced BV/TV and decreased type-H vessels at all age groups. NF-kB phospho-antibody array profiling identified a time-dependent decrease in phospho-NF-κB inhibitor alpha (phospho-IκBα) and phospho-P65ser529. Western blot analysis confirmed decreased phosphorylation of IκBαser32/36, a key regulator of the canonical pathway, at 6 weeks, suggesting its degradation may be decreasing heterodimer nuclear translocation. This finding correlated with reduced PDGF-BB expression, which was analyzed by parallelly detecting relative levels of 53 angiogenesis proteomes from bones of GIO WT mice at stated timepoints. Gene expression analysis revealed a potentially compensatory increase of IκBα, at 6 weeks. While no significant changes were observed in IκBα heterodimer p65 (RelA), p50 showed increased gene expression at 2- and 6-week treatments.

Conclusion: These findings suggest that GCs inhibit IκBα phosphorylation, leading to repression of canonical NF-κB nuclear translocation as the mechanism for reduced Pdgfb gene expression in preosteoclasts. Further experiments including interaction assays and IκBα knockout in osteoclast lineage will provide insights into its role in regulating the NF-κB pathway and PDGF-BB secretion, impacting Type-H vessels and overall bone health in young mice exposed to chronic GCs.

### P8

### Vertebral fracture severity and glucocorticoid dosing regimes predict vertebral fracture progression in boys with Duchenne muscular dystrophy (DMD)

Nicola Crabtree[1,2], Sarah McCarrison[3,4], Sze Choong Wong[3,4], Suma Uday[1,2], Vrinda Saraff[1,2]

[1]Birmingham Women's and Children's NHS Trust, Birmingham, UK [2]Institute of Metabolism and Systems Research, Univeristy of Birmingham, Birmingham, UK [3]Royal Hospital for Children, Glasgow, UK [4]Bone, Endocrine & Nutrition Research Group, University of Glasgow, Glasgow, UK

### Abstract

**Objectives:** Vertebral fractures (VF) are a recognised sequelae in boys with DMD receiving glucocorticoids (GC). Once identified, current treatment and prevention of further VFs is by bisphosphonate therapy. The objective of this review was to observe the changes in VF and back pain twelve months pre and post bisphosphonate initiation, in two different GC regimes.

**Methods:** A retrospective longitudinal review of boys with DMD and VF, initiated on intravenous bisphosphonates, between April 2014 and January 2023, due to concerns regarding VF. Inclusion criteria was spine imaging immediately prior to bisphosphonates and at least one lateral spine image in the preceding and following 12 months. Annual spine monitoring was performed with lateral dual-energy X-ray absorptiometry. Number and grade of VF was documented using the spine deformity index(SDI). Clinical details including GC regime, mobility, pubertal status, back pain, and bone density were collected. Boys were categorised according to GC treatment regime; daily (D) or intermittent (INTER). Boys on the daily regime were further sub-divided according to VF severity at treatment initiation using the following threshold: SDI< 6 = daily mild (D-M) or SDI >= 6 = daily severe (D-S).

**Results:** 34 boys were included; 13 were on intermittent (10 days on/10 days off) and 21 on daily CG regime of whom 7 had SDI≥6 at treatment initiation. At the time of treatment, INTER boys were older [INTER/D-M/D-S: 12.8(1.7)/10.7(2.2)/11.6(1.5) years, p=0.03] with longer time on GC therapy [INTER/D-M/D-S: 7.0(2.4)/4.2(2.5)/4.6(2.1) years, p=0.012]. INTER boys were also more likely to be pubertal but be less ambulant than both daily groups. Progression was rapid for boys in the D-S group compared to both INTER or D-M boys(see figure). Frequency of back pain was highest at treatment initiation but appeared to stabilise post initial bisphosphonate infusion [Back pain at treatment initiation: 54%, 64%, 83% versus 12m post: 18%, 55%, 29%, for INTER, D-M and D-S, respectively].

**Conclusion:** VF progression is most severe in boys on daily GC therapy with more severe VFs prior to treatment. Bisphosphonate treatment stems the development of VFs and reduces back pain. Early intervention, particularly for boys on daily GC with only mild VF appears to mitigate the most severe and painful VFs.

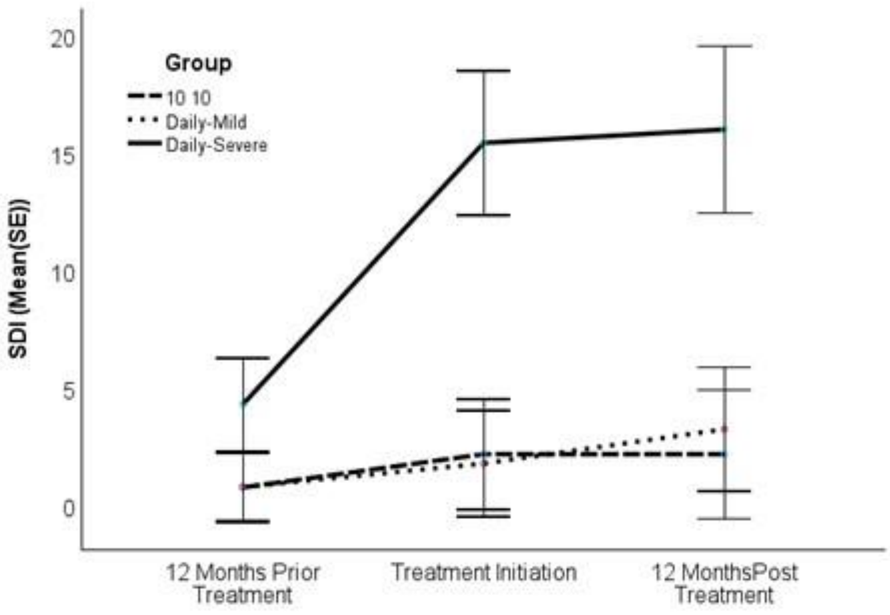

Group
10 10
Daily-Mild
Daily-Severe
SDI (Mean(SE))
20
15
10
5
0
12 Months Prior Treatment
Treatment Initiation
12 MonthsPost Treatment

### P9

### Elevated HbA1c is associated with impaired trabecular microarchitecture in boys with type 1 diabetes

Rylee Saunders[1], Kathleen Kilroe[1], Mary Bouxsein[2,3], Madhusmita Misra[1,4], Ruban Dhaliwal[1,5], Deborah Mitchell[1,3]

[1]Massachusetts General Hospital, Boston, USA [2]Beth Israel Deaconness Medical Center, Boston, USA [3]Harvard Medical School, Boston, USA [4]University of Virginia School of Medicine, Charlottesville, USA [5]University of Texas Southwestern Medical Center, Dallas, USA

### Abstract

#### Objective

Type 1 diabetes (T1D) is associated with a 5-7-fold increased risk of fracture, and altered bone acquisition in childhood may contribute to the increased lifetime fracture risk. We have previously demonstrated altered microarchitecture, particularly lower trabecular bone mineral density (BMD), in a cohort of girls with T1D (mean HbA1c 8.6 ± 1.3%). The goals of this study were to cross-sectionally evaluate differences in skeletal microarchitecture between boys with T1D and healthy controls and to assess factors associated with these differences.

#### Methods

We enrolled boys ages 6-20 years, 36 with T1D and 38 controls. Areal BMD (aBMD) was measured by dual energy x-ray absorptiometry (DXA), and volumetric BMD (vBMD), microarchitecture, and strength estimates were evaluated with high-resolution peripheral quantitative computed tomography (HR-pQCT).

#### Results

Groups were well matched for age, race, pubertal status, and height. Weight and BMI z-scores were non-significantly higher in T1D participants. Mean HbA1c in T1D participants was 8.0 ± 1.6%. After adjusting for age, height, and weight, no between-group differences were noted in aBMD, vBMD, microarchitectural parameters, and estimated failure load at any site.

To evaluate the role of glycemic control, we stratified T1D participants with HbA1c measures (n=32) per median HbA1c into T1D-low (n=17, HbA1c 5.6-7.8%) and T1D-high (n=15, HbA1c 7.9-11.5%) groups. These groups had similar demographic and anthropometric characteristics. At the distal tibia, we observed 11.8% lower trabecular bone volume (BV/TV) (p=0.067), 11.0% lower failure load (p=0.052), and 23.7% lower plate BV/TV (p=0.046) in T1D-high vs. control participants. We did not observe any between-group differences at the distal radius nor differences between T1D-low and control participants at any site.

#### Conclusion

As previously observed in girls, T1D is associated with altered microarchitecture and lower strength estimates in boys and young men, but, unlike in girls, only in those with higher average glycemia. This may reflect sex-specific effects of T1D on the growing skeleton, and/or effects of better glycemic control from improvements in and increased use of diabetes technology. Longitudinal studies investigating the role of sex steroids and the potential protective effect of optimizing glycemic control during growth on the long-term skeletal phenotype will be critical.

### P10

### Developing national consensus on management of osteoporosis in Duchenne muscular dystrophy in the transition to adult care within the UK adult NorthStar network

Shima Abdelrahman[1], Sarah McCarrison[2,1], Ros Quinlivan[3], Richard Keen[4], Sze Choong Wong[1,2]

[1]Bone, Endocrine & Nutrition Research Group in Glasgow, Human Nutrition, University of Glasgow, Glasgow, UK [2]Department of Paediatric Endocrinology, Royal Hospital for Children, Glasgow, UK [3]Centre for Neuromuscular Diseases, National Hospital, UCL Queen Square Institute of Neurology, London, UK [4]Metabolic Bone Disease Unit, Royal National Orthopaedic Hospital, Stanmore, UK

### Abstract

**Objectives:** Osteoporosis commonly occurs as a result of long-term glucocorticoid use and muscle weakness in Duchenne muscular dystrophy (DMD), rendering them susceptible to fragility fractures. Existing clinical guidelines for osteoporosis management in DMD primarily focus on paediatric care. Management during transition from paediatric to adult care is not clarified in current international guidance. A UK working group aims to establish a national clinical consensus for the management of osteoporosis in adult patients with DMD with focus at the time of transition from paediatric to adult care.

**Method:** An expert panel comprising of 13 adult and 5 paediatric bone specialists, 3 adult neuromuscular clinicians (2 of whom also manage paediatrics), 1 clinician scientist/densitometrist and 3 patient representatives was assembled to develop the clinical consensus through a series of online meetings. An online focus groups involving adults with DMD was organised to gather feedback on their experiences and the management of bone health. Systematic and scoping reviews on osteoporosis therapies for DMD, international consensus guidelines for glucocorticoid-induced osteoporosis in adults, and DXA predictions of fractures in DMD were conducted to inform the expert panel. In the initial round, the expert panel proposed areas of clinical care for which consensus statements will be formulated via an online survey. The final consensus statements were developed using the e-Delphi technique.

**Results:** Of the 22 clinicians invited to participate, 19 (86%) provided their suggestions in the first survey round. The suggested areas were categorised into eight main domains, covering various aspects of osteoporosis monitoring and treatment. The findings from the three literature reviews confirmed the paucity of evidence regarding bone health management in adults with DMD. A total of thirteen statements for clinical guidance have been developed and sent out for e-Delphi panel voting in December 2023. Twelve statements reached at least 75% agreement, and one statement is being updated before going into a third voting round. The final clinical consensus is expected to be published in the spring of 2024.

**Conclusion:** Employing an e-Delphi-based systematic approach, this study endeavours to develop national guidance for managing osteoporosis in adults with DMD in the UK and to guide management during transition.

### P11

### Exploring a novel osteogenesis imperfecta phenotype: Insights from BMP1 mutations and clinical management challenges

Sare Betul Kaygusuz[1,2], Ahmet Arman[1], Saygin Abali[3], Tarik Kirkgoz[1], Mehmet Eltan[1], Pinar Ata[1], Tulay Guran[1], Abdullah Bereket[1], Serap Turan[1]

[1]Marmara University School of Medicine, Istanbul, Turkey. [2]Istanbul University, Institute for Health Sciences, Istanbul, Turkey. [3]Acibadem Atakent Hospital, Istanbul, Turkey

### Abstract

### Background:

Osteogenesis imperfecta (OI) is an inherited metabolic bone disorder characterized by increased bone fragility and variable extraskeletal features, often stemming from mutations in COL1A1/COL1A2 genes. The bone morphogenetic protein (BMP), encoded by BMP1, is pivotal in collagen formation. Presently, 28 mutations in BMP1 have been identified in 38 OI patients from 34 families.

### Presenting Problem:

Our study features four BMP1 mutation cases from three families, introducing a phenotype reminiscent of skeletal dysplasia. We explore treatment responses and long-term outcomes in three cases, highlighting the challenges posed by clinical variability and bone density variations in managing BMP1 mutations.

### Clinical Management:

Patient 1: A 4.8-year-old boy, referred to us with a preliminary diagnosis of skeletal dysplasia due to kyphoscoliosis, presented with a height of 95 cm (-3.26 SDS) and weight of 11.2 kg (-3.78 SDS). No history of fractures was reported, and long bone radiographs revealed osteopenia without significant deformity or fractures. Patient 2: second patient with similar findings from same family, presented at 12.5 years old with a history of kyphoscoliosis surgery at the age of 4, resulting in paraplegia and three pathological fractures in long bones. Patient 3: A 10.6-year-old girl with a history of recurrent fractures. Clinical examination revealed rhizomelic, severe kyphoscoliosis, dentinogenesis imperfecta, and grey sclera. Patient 4: A 2.6-year-old boy with a history of recurrent fractures. Clinical examination revealed bowing and deformities in bilateral extremities, scoliosis, and blue sclera. Clinical findings, treatment, and follow-up information for the patients are summarized in Table 1.

### Discussion:

Our study unveils a novel OI phenotype in two patients characterized by severe spondylar involvement, brachydactyly, and contractures, with relatively unaffected long bones and a homozygous mutation in the CUB1 protein region. Remarkably, both patients showcased delayed motor development, relative macrocephaly, and severe kyphoscoliosis causing early spinal stenosis. Despite bisphosphonate treatment, none of the four patients exhibited fractures associated with increased BMD. This research enriches our understanding of the OI-Tip13 spectrum, shedding light on a distinct phenotype and associated mutations.

| | | Patient 1 | Patient 2 | Patient 3 | Patient 4 |
|---|---|---|---|---|---|
| *Variant (NM_001199.3)* | | c.1112G>A (p.Arg371His) | c.1112G>A (p.Arg371His) | c.851A>T (p.Asp284Val) | c.851A>T (p.Asp284Val) |
| Admission | Age (yrs) | 4.8 | 3.18 | 10.6 | 1.1 |
| | Height (SDS) | -3.26 | -5.12 | -6.9 | -3.7 |
| | BMD (SDS) | -4.51 | -4.66 | -4.11 | NA |
| Age at first fracture (yrs) | | - | 4 | 0.03 | 0.125 |
| Total number of fractures | | 0 | 3 | 23 | 11 |
| Deformity | Extremity | Radial bowing | Bowing, rhizomelic deformity | Bowing, rhizomelic deformity | Bowing |
| | Vertebrae | Severe platyspondylia, kyphoscoliosis, spinal stenosis | | Severe platyspondylia, kyphoscoliosis | Severe platyspondylia, scoliosis |
| | Pectus | + | + | + | + |
| Blue sclera / Dentinogenesis Imperfecta | | - / - | - / Discoloration and decay | Grey/ + | +/ Enamel hypoplasia |
| Additional features | | Coxa vara, Brachydactyly, Radius subluxation, Contracture in knees and elbows | Clubfoot, Brachydactyly, Radius subluxation, Contracture in knees and elbows | Femur subluxation | Arachnodactyly |
| Clinical Sillence | | 4 | 3 | 3 | 3 |
| Treatment | Age at initiation (yrs) | 5.19 | 12.5 | 0.5 | 1.1 |
| | Bisfosfanate | Pamidronate | Pamidronate | Pamidronate, Zolendronate | Pamidronate |
| Last admission | Age (yrs) | 12.08 | 18.7 | 17.8 | 2.6 |
| | Mobilization | Independent walking | Wheelchair bound | Walk with support | Wheelchair bound |
| | BMD ( SDS) | -4.46 | -4.43 | -1.3 | -0.76 |

### P12

### Evidence for bone mineral impairment in children with congenital adrenal hyperplasia

Marco Pitea, Marianna Stancampiano, Katia Maruca, Gianni Russo, Stefano Mora

IRCCS Ospedale San Raffaele, Milano, Italy

### Abstract

OBJECTIVES

Congenital adrenal hyperplasia (CAH) is a rare genetic disorder caused by deficient activity of the enzymes involved in the cortisol synthesis. Patients with CAH must receive glucocorticoids as replacement therapy from birth, and the dose is adjusted to avoid excessive or insufficient adrenal suppression. Data on bone mineralization in children with CAH are very scarce. The aim of the current study was to measure bone mineral density in a large cohort of prepubertal children with CAH. We also retested a subgroup of patients after completion of the pubertal development.

METHODS

We enrolled 57 prepubertal children with the classic form of CAH due to pathogenic variants in the CYP21 gene. All patients have been treated with glucocorticoids from birth. The mean age of the patients was 7.8 (1.5) years. They all underwent measurement of the bone mineral density (BMD) at the lumbar spine and whole body by dual-energy x-ray absorptiometry. BMD was also measured in a cohort of 60 healthy children aged 7.9 (1.6) years. Moreover, we measured BMD in a subset of 37 CAH patients who completed their pubertal development; they were aged 18.6 (1.4) years. Bone density measurements were compared to those of 51 healthy controls aged 19.1 (1.8) years.

RESULTS

Multivariate models have been used to compare BMD measurements of patients and controls. The model included sex and weight as confounding variables. Lumbar spine measurements of prepubertal subjects did not differ significantly in the two groups. Conversely, we found a significant ($P<0.0001$) difference in whole body measurements (Figure). BMD measurements of postpubertal patients did not differ from those of controls at the lumbar spine, but they were significantly lower ($P<0.05$) in the whole skeleton.

CONCLUSIONS

Our data indicate that children with CAH have low bone mineral measurements early in life compared to healthy children. Low bone mineral measurements are particularly evident in the whole skeleton, while lumbar spine does not seem to be affected. Bone mineral status should be monitored in patients to CAH to prevent further bone loss.

Prepubertal
1.0
0.8
0.6
0.4
0.2
0.0
g/cm2
Lumbar spine
1.0
0.8
0.6
0.4
0.2
g/cm2
*
Total Body
*P<0.001
Postpubertal
1.4
1.2
1.0
0.8
0.6
0.4
0.2
0.0
g/cm2
Lumbar spine
**
Total Body
**P<0.05

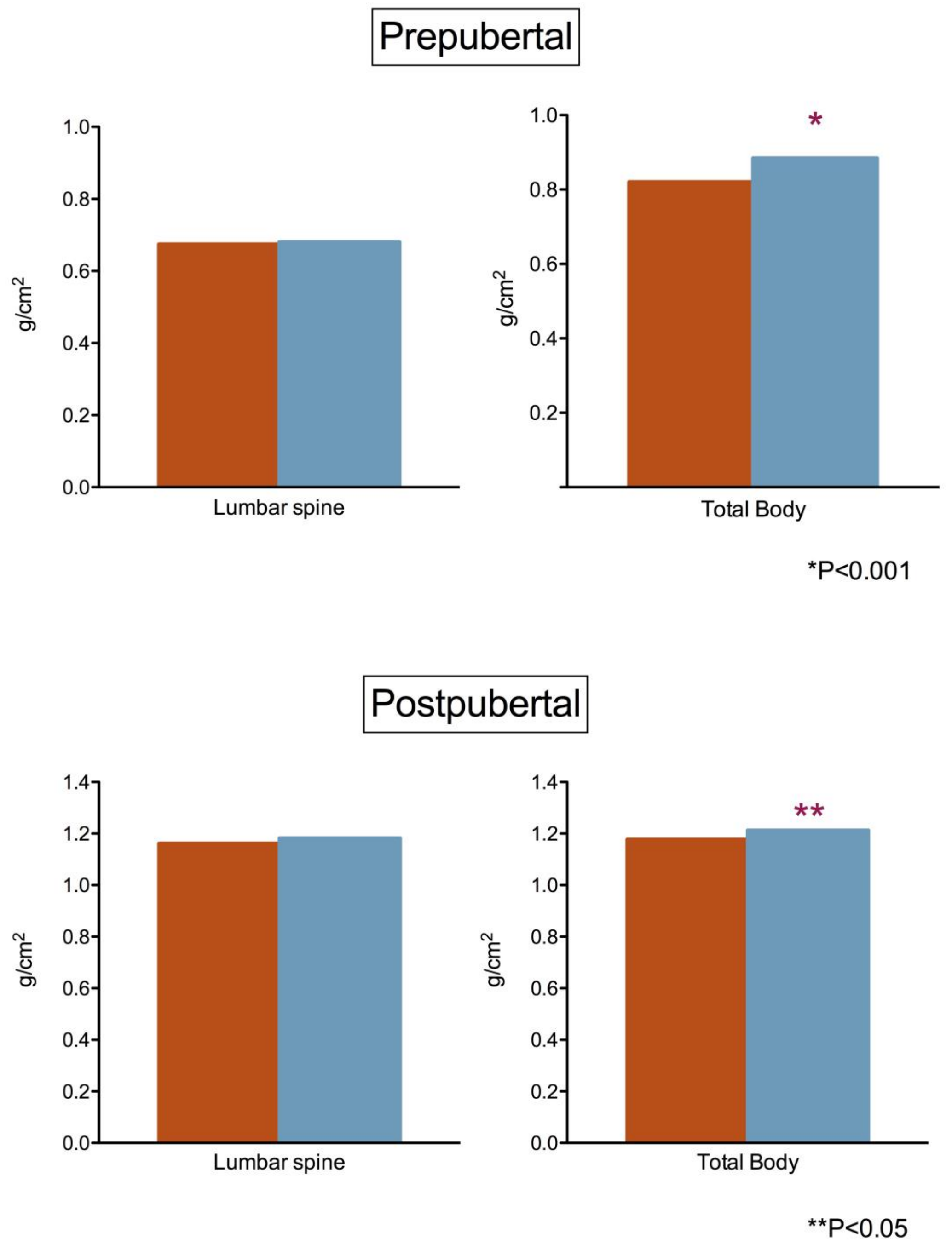

### P13

### Potential biomarkers predicting fracture risk in Duchenne Muscular Dystrophy (DMD)

Angela Sadlowski, Julia See, Weixin Zhang, Arryn Otte, Emely Loscalzo, Sisir Barik, Sonum Bharil, Janet Crane

Johns Hopkins University, Baltimore, USA

### Abstract

**Objectives:** By adolescence, 80% of Duchenne muscular dystrophy (DMD) patients on chronic glucocorticoids (GCs) sustain fractures. Our goal is to identify a biomarker to determine who on high doses of GCs is at highest risk of fracturing. We have shown in a young GC-induced osteoporosis (GIO) mouse model that GCs decrease preosteoclast (POC) platelet-derived growth factor type BB (PDGF-BB), reducing migration of endothelial precursor cells (EPCs) and mesenchymal stem cells (MSCs), ultimately impairing skeletal angiogenesis and osteogenesis.

**Methods:** We conducted a case-control study to determine if there was an association between fracture risk in DMD with PDGF-BB concentrations, as measured by ELISA, or peripheral blood mononuclear cell (PBMC) subpopulations of EPCs (CD34+, CD14-, Stro-1-, CD105-), MSCs (Stro-1+, CD105+, CD14-, CD45-), and POCs (CD14+, Stro-1-, CD105-), quantified by flow cytometry. Cases included 8–20-year-old DMD patients with GIO as defined by significant fracture history (D, n=14); two control groups included disease controls, defined as DMD patients on chronic GCs without fractures (DC, n=10) and age-and sex-matched healthy controls (HC, n=9). Differences between groups were assessed with one-way ANOVA with post-hoc Tukey's test in GraphPad Prism v9.4.1 whereas correlation between factors was analyzed with linear regression models in R v4.2.0.

**Results:** Baseline characteristics between groups were similar, except D and DC were shorter and had a higher BMI relative to HCs. Fractures in cases included vertebral (93%) and long bone (71%) and since biospecimen collection, five DCs and nine Ds have fractured; the median age of first fracture was 13.9 years. Mean PDGF-BB concentration positively correlated to EPC percent (p=0.03); POC percent negatively correlated with total-body-less-head Z-score (p=0.009). The percent of MSCs was lowest in D, intermediate in DC, and highest in HC (p=0.05). Interestingly, DC had significantly decreased PDGF-BB concentration (p=0.02), POC percent (p=0.002), and EPC percent (p=0.03) relative to HC. POC percentage was also decreased in DC relative to D (p=0.02).

**Conclusions:** With the unique pattern of PDGF-BB concentration and PBMC subpopulations observed in people with DMD and the high incidence of fractures since biospecimen collection, additional analyses are underway evaluating if any biomarker correlates with time to next fracture.

### Disclosure

JC receives research funding from Ultragenyx and Calcilytix. All other authors have no conflicts to declare.

### P14

### Response to bisphosphonates in patients with primary osteoporosis due to LRP5-related disorders: Including a novel mutation

Chamila Balagamage[1], Proteek Sen[1], Nicola Crabtree[1,2], Denise Williams[3], Suma Uday[1,2]

[1]Department of Endocrinology and Diabetes, Birmingham Women's and Children's NHS Foundation Trust, Birmingham, UK [2]Institute of Metabolism and Systems Research, College of Medical and Dental Sciences, University of Birmingham, Birmingham, UK [3]Department of Clinical Genetics, Birmingham Women's and Children's NHS Foundation Trust, Birmingham, UK

### Abstract

**Background:** LRP5-related disorders are a heterogeneous group of conditions characterized by variable reduced vision and juvenile osteoporosis. They are due to loss-of-function mutations in low density lipoprotein receptor-related protein-5 gene. The literature on response to bisphosphonate treatment is limited. We report the long-term response to zoledronate treatment in children with LRP5 mutation, including a patient with a novel mutation.

**Methods:** Retrospective review of patients with recessive LRP5-related disorder at a single tertiary centre. Data was gathered on clinical presentation, genetic mutation, treatment and bone densitometry parameters [lumbar spine (L1-4) (LS) BMAD Z-scores and total body less head (TBLH) BMD Z-scores)] including vertebral fractures on dual-energy X-ray absorptiometry (DXA) scan.

**Results:** Four patients (P1-P4) referred for recurrent low impact fractures, visual loss and mutations in LRP5 gene were included. The mean age of referral was 7 years (range 2-13). P1-P3 are siblings with a clinical diagnosis of LRP5-related disorder, but who are homozygous for c.1259 G>C p. (Gly420Ala) biparentally inherited variant of uncertain significance (VUS) in LRP5. P4 is compound heterozygous for -c.362 A>G p.(Lys121Arg) and c.2449 C>T p.(Arg817Cys) pathogenic variants in the gene.

P2, P3 and P4 (all males) had 4, 1 and 2 low trauma fractures respectively in addition to vertebral fractures requiring bisphosphonates, and familial exudative vitreoretinopathy requiring laser treatment. Whereas P1 (female) had no confirmed fractures and non-specific ocular involvement. P1, P3 and P4 had moderate learning disabilities.

P2, P3 and P4 received 6 monthly zoledronate infusion for a mean duration of 3.9 years (range 1.8- 8). For P1-P3, baseline LS BMAD Z-scores were -1.5, -1.8 and -1.9 and TBLH BMD Z-scores were -0.1, -0.7 and -1 respectively. Post-treatment change in LS BMAD Z-score (Δ) were, +1.7 and +0.7 for P2 and P3 respectively and both remain fracture free. P4 received bisphosphonates for 3 years pre DXA and on 5 years of treatment demonstrated Δ LS BMAD of +0.3 and vertebral remodelling.

**Conclusion:** We report clinical and molecular profile of four patients with LRP5-related disorders and osteoporosis including a novel mutation. Zoledronate treatment is effective in improving bone density, reducing fracture frequency and vertebral fracture remodelling.

**P15**

### Broader than bone fragility – the phenotypic spectrum of osteogenesis imperfecta type 15

Belinda Crowe[1], Christine Burren[2], Raja Padidela[3,4], Meena Balasubramanian[5,6], Christian De Goede[7], Toby Candler[2], Vrinda Saraff[8], Alistair Calder[1], Amaka Offiah[5,6], Jeremy Allgrove[1], Paul Arundel[5], Catherine DeVile[1], on behalf of the NHS England Highly Specialised Childhood Complex Osteogenesis Imperfecta Service[9]

[1]Great Ormond Street Hospital for Children NHS Foundation Trust, London, UK [2]University Hospitals Bristol and Weston NHS Foundation Trust, Bristol, UK [3]Manchester University NHS Foundation Trust, Manchester, UK [4]Faculty of Biology Medicine and Health, The University of Manchester, Manchester, UK [5]Sheffield Children's Hospital NHS Foundation Trust, Sheffield, UK [6]Division of Clinical Medicine, University of Sheffield, Sheffield, UK [7]Lancashire Teaching Hospitals NHS Foundation Trust, Preston, UK [8]Birmingham Women's and Children's NHS Foundation Trust, Birmingham, UK [9]National Health Service England Highly Specialised Services, Leeds, UK

### Abstract

**BACKGROUND**

Osteogenesis imperfecta (OI) type 15 is a rare autosomal recessive form caused by *WNT1* gene variants. In addition to bone fragility, it may have extra-skeletal features including neurodevelopmental impairment. Whilst pathogenic mechanisms are not fully understood, the Wnt signalling pathway is a key regulator of bone homeostasis and osteoblast activity, and *WNT1* variants may affect other signalling pathways critical for central nervous system development. We report three patients with OI type 15 across the NHS England Highly Specialised Childhood Complex Osteogenesis Imperfecta Service.

**PRESENTING PROBLEM**

All patients presented with peri- or neonatal fractures and were diagnosed with OI in infancy. Initial skeletal surveys revealed multiple clavicular, rib, and long bone fractures, without Wormian bones. Further long bone fractures and progressive vertebral compression fractures developed; the latter between 3–14 months old. Additional features are tabulated below.

| Clinical/Imaging/<br><br>Genetic Features | Patient 1 | Patient 2 | Patient 3 |
|---|---|---|---|
| Sex | Female | Male | Male |
| Year of birth | 2017 | 2017 | 2020 |
| Parental ethnicity | White British / White British | Southeast Asian Pakistani / Southeast Asian Pakistani | White British / White Newfoundland |
| Parental consanguinity | No (uniparental isodisomy for chromosome 12) | Yes | No |

| | | | |
|---|---|---|---|
| Family history (FH) of OI | Father heterozygous for *WNT1* variant<br><br>No previous FH of OI | Negative<br><br>Subsequent termination of affected pregnancy | Maternal forearm fractures, osteoporosis in maternal grandmother and great aunt |
| Antenatal scans | Normal | Normal | Mild femoral shortening at 32/40 (10th centile) |
| Scleral colour | White | Normal for ethnicity | Pale blue |
| Congenital ptosis | Yes – left | Yes – bilateral | No |
| Long bone deformities | Yes | Yes | No |
| Scoliosis | Yes – from 3 months old left convexity thoracic scoliosis, Cobb angle 22°<br><br>Progressive – aged 6 years Cobb angle 47° | No | No |
| Dentition | Normal | Normal | Normal |
| Movement disorder | Yes – dystonic motor disorder with extensor posturing in infancy; orolingual dyskinesia leading to recurrent tongue and lip biting (improved over time); currently stereotypies and mild dystonic posturing of limbs | Yes – stereotypies | No |
| Neurodevelopment | Autism spectrum disorder – scores well above age on cognitive assessment | Severe learning difficulties, non-verbal | No concerns reported |
| *WNT1* variant(s) | *WNT1*:<br><br>c.[216dupA];[216dupA]<br><br>p.[(Arg73Thrfs*62)];[(Arg73Thrfs*62)] | *WNT1*:<br><br>c.[400G>A];[400G>A]<br><br>p.[(Gly134Arg)];[(Gly134Arg)] | *WNT1*:<br><br>c.287_300del,<br><br>p.(Gln96Profs*58)<br><br>& c.688C>T, p.(Arg230Cys) |
| Brain magnetic resonance imaging | Normal<br><br>(Incidental bilateral incomplete hippocampal rotation) | Normal | Normal |
| Most recent bone mineral density (BMD) lumbar spine age-matched Z-score | -4.7<br><br>(Bone mineral apparent density Z-score: -4.87) | Not available since unable to tolerate scan | Not available since under 5 years old |

### CLINICAL MANAGEMENT

All patients commenced intravenous bisphosphonates between 1–12 months old, with uncertain response. Patient 1 showed improved vertebral modelling after 10 months of treatment despite ongoing long bone fractures, but now aged 7 years has little improvement in BMD, unchanged widespread thoracic vertebral height loss and recurrent upper limb fractures. Patient 2 continues to have severe vertebral fractures with no remodelling; multiple lower limb fractures have reduced after intramedullary rodding. Patient 3 developed vertebral compression fractures aged 12 months despite starting bisphosphonates aged 2 months. All patients have undergone lower limb rodding – 1 and 2 bilaterally. Patient 2's ptosis transiently improved with oral salbutamol.

### DISCUSSION

In addition to bone fragility OI type 15 can be associated with neurodevelopmental impairment, ptosis, movement disorders and autism spectrum disorder. The extra-skeletal features may be most limiting in terms of participation and quality of life. Education of professionals caring for children with this condition is essential, to improve awareness of the phenotypic spectrum and identify associated co-morbidities. This also enables early intervention and multidisciplinary team input to achieve best possible developmental outcomes. All patients have had uncertain benefit from intravenous bisphosphonates. Anti-sclerostin antibodies which activate Wnt signalling may be a more appropriate treatment option for children with OI type 15 and this requires further study.

### P16

### Avulsion injuries in paediatric osteogenesis imperfecta

Emilie Hupin, Mark Heathfield, Jeremy Allgrove, Moira Cheung, Alistair Calder, Andreas Roposch, Belinda Crowe, Catherine DeVile

Great Ormond Street Hospital, London, UK

### Abstract

**Objectives:** Avulsions of the olecranon have been described in Osteogenesis Imperfecta (OI). Although tibial tuberosity avulsions have also been reported, the literature on avulsion fractures at non-olecranon sites is sparse.

We report the incidence and characteristics of all avulsion injuries sustained by patients from a large specialist centre for paediatric OI.

**Methods:** Records of 522 patients were reviewed retrospectively. Avulsion injuries were identified and confirmed by an expert radiologist. Data including age, sex, severity, site of injury, mechanism, treatment, bone mineral density (BMD) Z-scores and bisphosphonate therapy were collected.

**Results:** Sixty-one avulsion injuries occurred (8.6% incidence), in 45 patients. Most patients were male (71.1%) and most (93.3%) had Type 1 OI. All patients were ambulant. The mean age at time of injury was 11.7 years. 73.8% of injuries occurred when patients were bisphosphonate naïve with a mean BMD Z-score of -1.5 (nearest to time of fracture).

The majority of avulsion injuries occurred at the olecranon (73.8%, n=45/61). Of these, 37.5% of patients had bilateral occurrences. The most common mechanisms of injury were falls (64.4%) followed by a direct blow to the elbow (20%). 84.4 % required surgical treatment.

26.2% (n=16/61) avulsion injuries occurred at non-olecranon sites. 50% involved tibial tuberosity avulsions; two patients experienced simultaneous bilateral avulsions. Other sites included the lateral malleolus (n=2), inferior iliac spine (n=1), tibial spine (n=1) and metatarsal (n=1). All injuries were sustained following trauma. Seven patients had also experienced an olecranon avulsion fracture.

**Conclusion:** Avulsion injuries, specifically affecting the olecranon, are common and well recognised in ambulant patients with Type 1 OI. Avulsion fractures of the tibial tuberosity are the most prevalent lower extremity avulsion. Bilateral occurrences were seen affecting the olecranon and tibial tuberosity, and 15.6% of patients had sustained both olecranon and lower extremity avulsions. This suggests patients are at increased risk of recurrence and should be counselled on minimising trauma.

Since our data were retrospective, with all injuries treated at other healthcare centres, it is likely not all avulsions in our population were identified and the overall incidence may therefore be an underestimation.

### P17

### Bone fragility and shape of the femur diaphysis: Quantitative investigation in the osteogenesis imperfecta mouse (OIM)

Grégoire André, Antoine Chretien, Pierre-Louis Docquier, Catherine Behets

IREC, Brussels, Belgium

### Abstract

**Objectives:**

Osteogenesis imperfecta (OI) is a rare congenital bone dysplasia generally caused by a mutation of one of the type I collagen genes and characterized by low bone mass, numerous fractures, and bone deformities. In the osteogenesis imperfecta mouse (oim), a validated model of severe OI, we wanted to identify an alteration of the femur shape, as well as its possible association with bone fragility.

**Methods:**

Wildtype (wt) and oim mice were scanned in vivo at week 5, 11 and 17 to assess the fractures. After euthanasia at 17 weeks of age, left femurs, without fracture and/or deformity visible with x-rays (n=6 for both groups), were dissected and analyzed with pQCT to quantify bone mineral density (BMD), BV/TV, cortical thickness and stress strain index (SSI). To assess bone geometry, 300 10-µm-thick slices were acquired from distal diaphysis with Nano-CT (voxel size of $10 \mu m^3$). ImageJ software allowed to study cortical minor axis/cortical major axis and circularity of each slide, giving a ratio of elliptic-shape and circular-shape of the diaphyseal cortex, respectively, as well as solidity which corresponds to a ratio of compactness of the cortical bone.

**Results:**

cortical BMD, BV/TV, cortical thickness and SSI were significantly lower in oim than wt group. As illustrated with Nano-CT, the diaphyseal cortex was thinner , less circular and more ellipse-shaped in oim than wt mice ($p<0.05$ for circularity and minor axis/major axis; $p<0.01$ for solidity).

**Conclusion:**

In addition to thinner cortex, lower BV/TV and lower cortical BMD, oim femurs are less circular and more elliptical than wt bones. Since spontaneous fractures are characteristics of oim phenotype, this altered cortical shape could also have an impact on bone fragility and biomechanical properties.

### P18

### Vertebral fracture assessment and progression of vertebral fractures prior to bisphosphonate treatment in boys with Duchenne muscular dystrophy (DMD)

Nicola Crabtree[1,2], Sarah McCarrison[3,4], Vrinda Saraff[1,2], Sheila Shepherd[3,4], Suma Uday[1,2], Sze Choong Wong[3,4]

[1]Birmingham Women's and Children's NHS Trust, Birmingham, UK [2]Institute of Metabolism and Sytems Research, University of Birmingham, Birmingham, UK [3]Royal Hospital for Children, Glasgow, UK [4]Bone, Endocrine & Nutrition Research Group, University of Glasgow, Glasgow, UK

### Abstract

**Objectives:** Current (2018) International Care Considerations for DMD recommend regular spine monitoring for vertebral fracture (VF) and initiation of bisphosphonates following identification of moderate or severe VF even without back-pain or VF of any grade with back-pain. Our objective was to investigate the progression in vertebral fractures and pain in the twelve months preceding treatment with intravenous bisphosphonate therapy.

**Methods:** A dual-centre retrospective study of boys with DMD initiated on IV bisphosphonates due to concerns about VF were identified from spine monitoring between 2015 and 2023. Inclusion criteria was spine imaging immediately prior to bisphosphonate treatment and at least one lateral spine image in the preceding 12 months. Annual spine monitoring was performed with lateral dual-energy X-ray absorptiometry or lateral spine radiographs. For purposes of this study, anonymized spine images were centrally reported by a single observer. Boys were categorised according to severity of VFs at time of treatment; spine deformity index (SDI) < 6 = MILD or SDI >= 6 = SEVERE.

**Results:** 39 boys with DMD on daily glucocorticoids were included. Mean age of first bisphosphonate treatment was 11.4 [7.6-17.6] years. Mean duration of glucocorticoid therapy was 5.4 [0.9-10.6] years. At initiation of bisphosphonates 22 (56%) had moderate or severe VFs of whom 16 (73%) had back pain, 9 (23%) had mild VFs of whom 4 (44%) had back pain and a further 8 (21%) had mild height loss (10-20%) of whom 6 (75%) had back pain. Categorising by SDI, 26 and 13 boys were allocated to the MILD and SEVERE groups, respectively. Boys in the SEVERE group had significantly higher SDI compared with those in the MILD group twelve months prior to treatment [5.6 (3.9) vs. 1.8 (2.3); p<0.001] and progressed to more severe, painful fractures at the time of their first infusion [SDI 14.0 (6.5) vs 3.7 (2.3); p<0.001].

**Conclusion:** VFs in boys with DMD, on daily glucocorticoids, deteriorate faster with more back pain before treatment is initiated, when 12-month preceding SDI scores are higher. Early treatment, for mild VFs or even mild height loss is likely to stem further VFs and more importantly significantly reduce future back pain. These results could be used as a basis for a review of the 2018 Care Considerations.

### P19

### Prevalence of fractures among children with type 1 and type 2 diabetes

Nourah Almutlaq[1,2], Sarah Fitzpatrick[3], Linda Dimeglio[1]

[1]Riley hospital for children, Indianapolis, USA [2]Medical cities by Ministry of Interior, Riyadh, Saudi Arabia.
[3]Riley hospital for children, Indianpolis, USA

### Abstract

### Introduction:

Bone fractures are common in children, with incidence rates ranging from 1.2% to 5% across different studies. However, little is known regarding the prevalence and characteristics of fractures in children with diabetes. We investigated the prevalence and types of fractures in children living with type 1 and type 2 diabetes(T1D, T2D).

### Methods:

A fracture survey was conducted at Riley Hospital for Children, in the pediatric diabetes clinic including children ≤ 18 years with T1D or T2D, gathering data on fracture occurrences and locations. Demographic and diabetes-related information were extracted from medical records.

### Results:

Data were obtained from 416 children (age range: 5-18 years, 50% male, 83% T1D/17% T2D), among whom 3.4% were diagnosed with celiac disease and 18.2% with thyroid disease. The lifetime incidence of fracture was 25.5% (106/416), with T1D accounting for 83% and T2D accounting for 17% of cases. There was no significant difference in fracture incidence observed between children with T1D and T2D (25.3% vs. 26.1%, p=0.47). Notably, 20% (21/106) of the fractures occurred after the diabetes diagnosis. T1D children with fractures had a younger age at diagnosis (7.6 years vs. 8.9 years, p = 0.047), longer diabetes duration (6 years vs. 5 years, p = 0.03), and higher male prevalence (62.5% vs. 48.8%, p = 0.018). Conversely, T2D children with fractures were older (15.4 years vs. 12.5 years, p = 0.001) and had lower HbA1C levels (8.3% vs. 10.1%, p = 0.02) compared to the non-fracture T2D group. The most frequently reported fracture locations were the small bones of the wrist/hands (23.9%), upper arm (17.9%), and small bones of the ankle/feet (14.9%). Fracture rates were not higher in diabetic children who had concomitant celiac or thyroid disease.

### Conclusions:

Our study reveals a notable occurrence of fractures in children with T1D and T2D, with about one-fifth occurring after diabetes diagnosis. Furthermore, unique fracture patterns in each diabetes subtype emphasize the need for tailored prevention strategies

### P20

### Bone health status in young patients with Klinefelter syndrome assessed by DEXA scan and correlation with hypogonadism

Jessica Borg Olivier, Tet Yap, Christina Wei, Rui Santos, Alessandra Cocca

Guys and St Thomas', London, UK

### Abstract

#### Objective

Osteoporosis is reported in 6-15% of adults with Klinefelter syndrome (KS). There is limited information in bone health status in younger patients with KS. This project aims to describe bone mineral density (BMD) and its associations with gonadal status of patients under a dedicated KS multidisciplinary (MDT) clinic.

#### Methods

Patients from the young adult KS MDT clinic who underwent their first Dual-energy X-ray absorptiometry (DEXA) scans between September 2020 and July 2023 were included. Data including age at time of DEXA, serum testosterone levels, DEXA outcomes [total-body-less-head (TBLH)-BMD, lumbar spine (LS)-BMAD, vertebral fracture assessment (VFA)], and testosterone treatment, were collected.

#### Results

19 KS patients (all male) aged 16-21 years with karyotype 47XXY (n=16), 48XXYY (n=2) and 48XXXY (n=1) were recruited. Results were expressed as mean (SD). Age of first DEXA was 16.9 (1.48) years. DEXA outcomes reported TBLH-BMD -1.16 (0.73) and LS-BMAD -0.37 (0.9).

One sample T test showed no statistical difference of both TBLH BMD(p=0.8) and LS BMAD(0.2) with the general population.

5 patients were on testosterone treatment when DEXA were performed.  Testosterone [13.9(6.6) nmol/L] was >10nmol/L in 13 patients, 5 of whom were on testosterone replacement. One patient with a vertebral fracture, had testosterone level >10nmol/L was not on replacement.

There was no statistically significant difference in TBLH-BMD ($P$=0.5864) or LS-BMAD ($P$=0.7462) in patients with testosterone level <10 vs >10nmol/L. No statistical difference was found in LS-BMAD ($P$= 0.6132) or TBLH-BMD ($P$= 0.562) in patients treated with or without testosterone. No correlation was found between testosterone levels and TBLH-BMD (p= 0.9, $R^2$<0.1) or LS-BMAD (p=0.9, $R^2$ 0<0.1).

#### Conclusions

Currently, there is a lack of guidance on the management of bone health in younger KS patients. Our study showed BMD within normal limits in young KS patients. The lack of correlation between testosterone levels and BMD may be due to the timing of DEXA scan in our protocol. Testicular failure, which is progressive in adulthood, may not have been present at the time of our patients' first scans. These results also raise questions if bone loss in KS may be independent of hypogonadism. Further longitudinal studies with larger sample sizes are necessary to inform consensus on the appropriate bone health surveillance of patients with KS.

### P21

### Technology for inclusion: Using AV1 robots to support young people with osteogenesis imperfecta to participate in school

Zoe Bowman Bayles[1], Moira Cheung[1], Belinda Crowe[1], Jeremy Allgrove[1], William Lau[2], Catherine DeVile[1]

[1]Great Ormond Street Hospital for Children NHS Foundation Trust, London, UK [2]The Children's Hospital School at Great Ormond Street Hospital, London, UK

### Abstract

**BACKGROUND:**

Attendance at school can be significantly impaired in children with severe osteogenesis imperfecta (OI), due to fractures and medical appointments/procedures. This impacts on learning and social interaction which leads to feeling isolated. Strategies were trialled by the specialist OI Occupational Therapy (OT) team of a large children's hospital.

One strategy was the use of charity funded 'AV1 robots' to encourage greater participation in school. These avatar robots are portable, sit on the child's desk and are equipped with a camera, speaker and microphone It is linked via a remote app through which the child can see, hear and speak through it to have a virtual presence in school.

**Cases:**

First patient: 13-year-old girl with type 3 OI, who uses a power wheelchair. Although she is able to attend school, she was unable to access any of her classes due to faulty lifts (>6months). She identified her performance problem was not being able to interact in lessons and socialise with friends.

Second patient: 14-year-old girl with OI type 3 similarly needing a power wheelchair. She had not been able to access school for nearly a year after spinal surgery. Performance problems included anxiety around forming friendships, a busy environment, fatigue, pain and her physical appearance.

The AV1 robots were identified as a potential aid to overcome some of these performance problems and provide students with a physical presence in the classroom.

Our OT partnered with school to plan and implement access to the robots. Outcomes were evaluated using the Canadian Occupational Performance Measure (COPM) capturing performance and satisfaction to their self-identified goals.

**DISCUSSION:**

Important aspects of OI management include promoting independence, maintaining participation, improving quality of life and managing pain and fatigue. Different innovative strategies using technology should be considered, such as the use of AV1 robots to overcome social isolation and facilitate access to learning. These robots might also enable interaction whilst offering privacy when a child is unwell, has had a procedure and wishes not to be visible.

### P22

### Application of a polygenic risk score for predicting osteoporosis in children with medical complexity - POP-CMC pilot cohort

Despoina Manousaki, Maria Marano, Marie-Joelle Doré-Bergeron, Nathalie Alos, Patricia Olivier, Valérie Bonneau, Melissa Fiscaletti

CHU Sainte Justine, Montreal, Canada

### Abstract

Children with medical complexity (CMC) have an increased risk of fragility fractures. Our objective was to assess if a polygenic risk score (PRS) called g(SOS) which has been associated with severe fracture risk in healthy children and acquired osteoporotic fracture in adults would predict bone fragility in a CMC cohort. There are few research efforts to describe and predict optimal bone health outcomes for CMC. Our aim at this phase was to test the feasibility of recruiting and retaining this patient population over 1 year without increasing hospital visits.

**Methods**:

Eligible children 1-18 years of age followed by the Complex Care Clinic at CHU Sainte Justine were identified using chart reviews. All participants underwent a spine X-ray, and DXA scan to measure spine-, total body less head-, and hip BMD at baseline with repeat imaging after 1 year. Nutritional status and calcium and vitamin D intakes were determined by a registered dietician. Blood was drawn to measure bone metabolism markers and extract DNA. Genome-wide genotyping, quality control, imputation and subsequent bioinformatic analyses to compute the PRS was undertaken. Research visits were coupled with scheduled hospital visits.

**Results**:

31 participants were recruited in a pilot study over 12-month period. 71% were male (N=22), and their mean age was 7 years (range 1-17yrs). In a sample of 13 participants with currently available data, the median lumbar DXA BMD Z-score at baseline was 0.6 (range 0-0.909). We have available genotype data from 23 out of the 31 participants, which were imputed using the TOPMed reference panel. The 23 participants were of European ancestry and principal component analysis did not reveal ethnic outliers. PRS (gSOS) measures were calculated for the 23 individuals and showed a normal distribution. No participants have been lost to follow-up and data ascertainment is ongoing.

**Conclusion**:

It is feasible to recruit CMC in a prospective bone health research study with multiple assessments including genetic extraction and PRS computation. While recruitment rates may be delayed by coupling research visits with clinical visits, accommodating this medically fragile population in remains imperative.

### P23

### Serial B-CTX and P1NP trends during bisphosphonate treatment in the paediatric OI population

Chris Worth, Jennifer Whitehead, Lauren Rayner, Raja Padidela, Amish Chinoy

Royal Manchester Children's Hospital, Manchester, UK

### Abstract

### Objectives

Intravenous bisphosphonate treatment is the standard of care for many children with osteogenesis imperfecta (OI) to reduce the risk of fractures. While bone turnover markers are widely used in adults, there is a paucity of data on the utility of these in children. We aimed to measure serial bone turnover markers (B-CTX and P1NP) in this population to investigate for change with ongoing treatment and potential clinical utility.

### Methods

We studied all children with OI receiving bisphosphonates between September 2022 to December 2023 in a tertiary centre. Pre-infusion blood tests for B-CTX, P1NP, 25-hydroxyvitamin D, calcium, phosphate and alkaline phosphatase were analysed alongside bisphosphonate dose, frequency, cumulative dose, auxology and age in both stepwise and multiple logistic regression to identify features most impacting on B-CTX and P1NP levels.

### Results

During the study period, 68 patients (aged 5w-16y) underwent 175 bisphosphonate infusions. Of these, 57 individuals had more than one B-CTX value available for trend analysis. Change in B-CTX was highly variable between patients (Figure 1) but demonstrated a small mean relative increase of 6% over time. Sequential P1NP measurements were available
for 56 individuals and demonstrated similar interpatient variability with a small overall relative increase of 6% over time.

Logistic regression showed a small, but statistically significant, decrease in B-CTX with cumulative bisphosphonate dose ($R2 = 0.12$, $p<0.001$) but this effect disappeared when adjusting for confounders ($p=0.3$). P1NP showed a small decrease with cumulative bisphosphonate doses ($R2 = 0.27$, $P<0.001$) and persisted when adjusting for confounding factors ($p=0.002$).

### Conclusions

Our data suggests limited clinical utility for B-CTX and P1NP as monitoring tools during bisphosphonate therapy in children with OI. The majority of patients were already established on bisphosphonates at the study start and thus it is not possible to comment on initial change in B-CTX and P1NP following first treatment. Unlike in adult practice, this study indicates that B-CTX and P1NP may not be reliable indicators of treatment response in paediatric OI and should not guide dose adjustment. A longer-duration study, in combination with serial bone density measurement, will be required to confirm these findings.

Change in B-CTX with ongoing bisphosphonate infusions
B-CTX ng/L (line of best fit)
2000
1750
1500
1250
1000
750
500
250
0
0
10
20
30
40
50
Dose of bisphosphonate

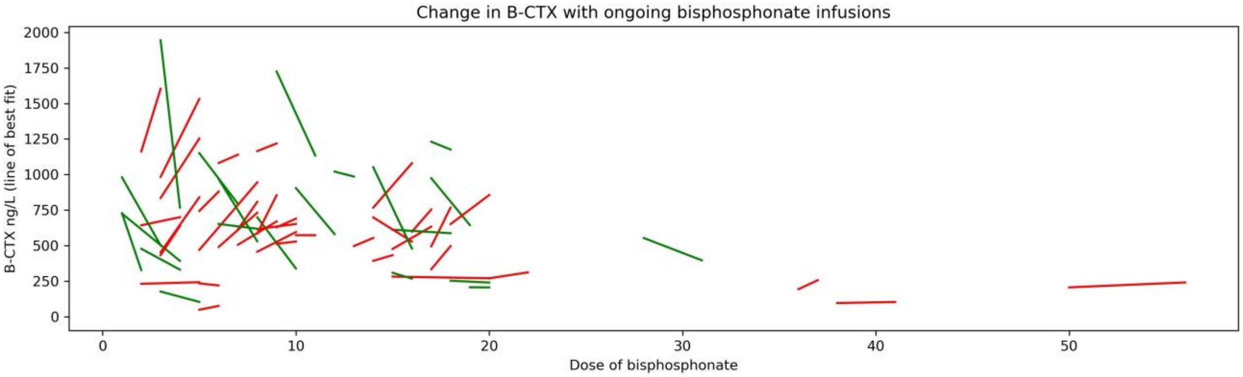

Change in P1NP with ongoing bisphosphonate infusions
P1NP ug/L (line of best fit)
250
200
150
100
50
0
0
10
20
30
40
50
Dose of bisphosphonate

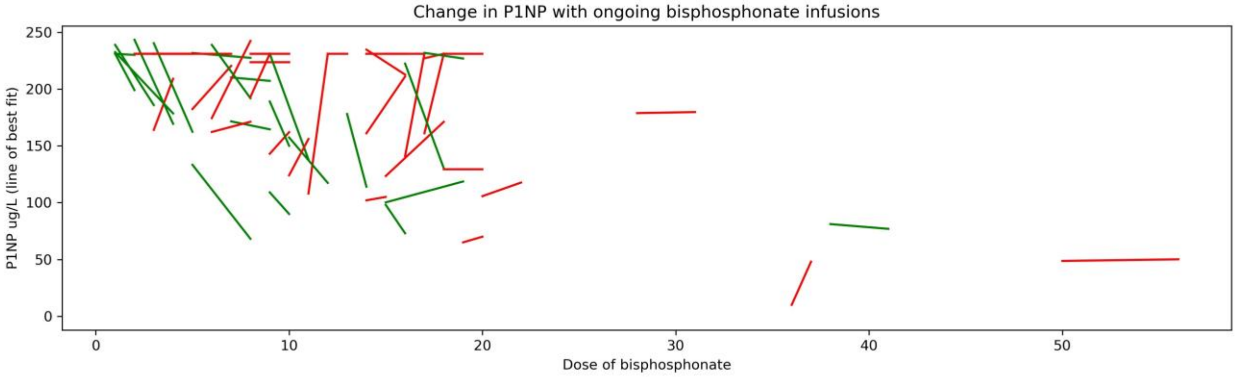

### P24

### Systematic review of clinical practice guidelines and recommendations on the management and treatment of glucocorticoid-induced osteoporosis in adults: Implication for adolescents and young adults on long-term glucocorticoid treatment

Shima Abdelrahman[1], Sarah McCarrison[1,2], Ros Quinlivan[3], Richard Keen[4], Sze Choong Wong[1,2]

[1]Bone, Endocrine & Nutrition Research Group in Glasgow, Human Nutrition, University of Glasgow, Glasgow, UK [2]Department of Paediatric Endocrinology, Royal Hospital for Children, Glasgow, UK [3]Centre for Neuromuscular Diseases, National Hospital, UCL Queen Square Institute of Neurology, London, UK [4]Metabolic Bone Disease Unit, Royal National Orthopaedic Hospital, Stanmore, UK

### Abstract

**Objectives:** Evidence to support guidance on the monitoring and treatment of glucocorticoid-induced osteoporosis (GIOP) in adults on long-term glucocorticoid treatment since childhood is limited.

This systematic review aimed to identify clinical practice guidelines (CPGs) and recommendations on the monitoring and treatment of GIOP in adults and consider the relevance to young adults on long term glucocorticoid treatment since childhood, such as Duchenne Muscular Dystrophy (DMD) especially in relation to ongoing use of osteoporosis therapy.

**Methods:** CPGs and recommendations published until December 2023 were identified from Ovid MEDLINE, Ovid Embase and the Cochrane Library. Grey literature was also searched using CADTH's Gray Matters Light. Search included any CPGs, recommendations, position statements and clinical consensus addressing GIOP in adults.

**Results**: Forty CPGs and recommendations met the inclusion criteria. Nineteen were osteoporosis CPGs where GIOP was addressed as part of the document; thirteen were GIOP specific CPGs and eight were CPGs in other medical conditions where glucocorticoid therapy is used. In nineteen CPGs (48%), age of the target population was not addressed. Only twelve (30%) of the CPGs addressed treatment of GIOP in younger patient groups (<50 years) (published between 2010-2023). However, it remains unclear whether these recommendations are applicable to younger adults (18-30 years). Oral bisphosphonates were recommended as first line treatment in high-risk patients even without fractures in six CPGs, while three CPGs recommend treatment based on clinical judgment and three other CPGs recommend treatment only following fracture. Only three CPGs recommended regular bone health reviews with fracture risk assessment and bone density testing. None of the CPGs addressed management of long-term osteoporosis therapy in those on ongoing glucocorticoid therapy. There was lack of clarity on patient involvement in the development of the CPGs.

**Conclusion:** Current clinical practice guidance do not provide recommendations for management of osteoporosis therapy in young adults on long-term glucocorticoid treatment in particular those who have initiated osteoporosis therapy in childhood. Expert recommendations are needed in high risk groups like young adults with DMD to provide guidance on monitoring and management of osteoporosis therapy during transition and in adult care.

### P25

### Improvements on bone density is not reflected in quality of life outcome measures in children with Idiopathic Juvenile Osteoporosis

Lauren Rayner, Nicola Panchbhaya, Rosie Ferreira, Helen Collins, Rebecca Doodson, Raja Padidela, Amish Chinoy

Royal Manchester Children's Hospital, Manchester, UK

### Abstract

### Introduction:

Idiopathic Juvenile Osteoporosis (IJO) is a form of primary osteoporosis in children, usually presenting with vertebral fractures causing severe back pain. Treatment is with bisphosphonates as well as a multidisciplinary team approach to manage pain and function. This study assessed the impact of bisphosphonates on bone mineral density and quality of life in children with IJO.

### Methods:

The PedsQL Pediatric Quality of Life Inventory questionnaire was completed by both patients and parents to measure quality of life in children with IJO, both at the start of bisphosphonate treatment and on follow-up. Along with a total score, physical and psychosocial domain subsets can also be measured. Bone mineral density was monitored by reviewing lumbar spine bone mineral apparent density (BMAD).

### Results:

7 children (6 male, mean age 12.2 years) with IJO on bisphosphonate treatment completed the questionnaire, along with their parents/carers.

Lumbar spine BMAD standard deviation scores (SDS) were significantly low at baseline:mean -3.2, standard deviation [SD] 1.4, P<0.001). This improved significantly with treatment (mean interval 1.7 years): mean change in lumbar spine BMAD SDS 2.8 (SD 1.3, P=0.008).

There was a significant deficit in mean total score SDS for the PedsQL questionnaire completed by the patient at baseline: -2.5 (SD 1.1), P=0.001. The physical domain SDS (mean -3.2, SD 2.0, P=0.006) was affected more than the psychosocial domain SDS (mean -1.9, SD 1.1, P=0.004). There were similar deficits in parent-completed questionnaires with low total (mean -2.3, SD 0.9, P<0.001), physical (mean -2.2, SD 1.3, P=0.004) and psychosocial (mean -2.0, SD 0.9, P=0.001) score SDS. At follow-up (mean interval 1.4 years), there was no statistically significant change in total (mean change 0.5, SD 1.2, P=0.28), physical (mean change 0.6, SD 3.1, P=0.63) and psychosocial (mean change 0.5, SD 0.8, P=0.12) score SDS in patient-completed questionnaires.

### Conclusions:

Children with IJO continue to experience significant quality of life impairments in both physical and psychosocial domains, highlighting the need for comprehensive multidisciplinary management strategies beyond bisphosphonates to improve well-being alongside bone mineral density.

### P26

### Evaluation of virtual school education sessions for children with metabolic bone disorders with Occupational Therapy (OT) and Physiotherapy (PT) needs

Helen Collins, Rebecca Doodson, Nicola Panchnhaya, Rose Ferreira, Lauren Rayner, Amish Chinoy, Raja Padidela

Royal Manchester Children's Hospital, Manchester, UK

### Abstract

OBJECTIVES: OT and PT are required for a range of Metabolic Bone Disorders including osteogenesis imperfecta, hypophosphatasia, secondary osteoporosis and geroderma osteodysplastica. One of the roles of the therapy team is to educate the school educators to create a safe environment for children and empower them to manage the child's needs so they can participate in school. These education sessions are effective if individualised for each patient's needs and are best offered at the transition into primary and secondary school. The qualitative study aims to assess if these sessions are effective in educating schools and gain feedback on improving these sessions.

METHODS:

Virtual Education sessions were provided to schools. Two questionnaires were created to establish the school's level of knowledge; one before the education session and one after. The questionnaires contained questions regarding:

- Level of knowledge of bone condition
- Where to find useful resources
- Confidence in supporting students with bone conditions
- Knowledge of activities students can engage with
- Feedback on session

RESULTS:

Eleven educational sessions were carried out between July and September 2023, four in high school and seven in primary school. Nine pre-education questionnaires and six post-education questionnaires were returned by the school. The level of knowledge was scored on a scale of 1-10. The median pre-education level of knowledge was 2 and this increased to a median of 8 post-education. The median pre-education level of confidence in implementing adjustments for pupils was moderate, rising to very confident following education.

CONCLUSIONS:

Virtual education sessions provided schools with new knowledge about the condition and gave them confidence to support the students within the school environment. Delivering the sessions virtually is cost effective allowing more members of the school to join and enabling us to deliver more sessions during the transition period. From the feedback, we will reorganise sessions to reinforce individual needs especially around the changes in school staffing or following a major event such as a fracture. We will continue to ask for feedback on our education sessions to continually develop our training.

### P27

### Rapid decline in bone health following acquired brain injury

Belinda Crowe[1], Marianna Shiafkou[2], Kshitij Mankad[3], Alistair Calder[4], Margaret Kaminska[5], Lucinda Carr[1], Jeremy Allgrove[2]

[1]Paediatric Neurosciences, Great Ormond Street Hospital for Children NHS Foundation Trust, London, UK
[2]Paediatric Endocrinology, Great Ormond Street Hospital for Children NHS Foundation Trust, London, UK
[3]Paediatric Neuroradiology, Great Ormond Street Hospital for Children NHS Foundation Trust, London, UK
[4]Paediatric Radiology, Great Ormond Street Hospital for Children NHS Foundation Trust, London, UK
[5]Paediatric Neurosciences, Evelina London Children's Hospital, Guy's and St Thomas' NHS Foundation Trust, London, UK

### Abstract

**BACKGROUND:** Secondary osteoporosis is a known complication of severe physical disability and can be associated with pathological fractures. Optimisation of bone health and monitoring for fractures is therefore essential in children with established motor disorders such as cerebral palsy. However, there is no current consensus on how soon to consider this following acquired brain injury.

**PRESENTING PROBLEM:** A 5-year-old girl suffered a 20-minute cardiac arrest following a choking episode on food. She was previously typically developing apart from mild gross motor delay – she walked aged 2 years, with no history of fractures. She developed hypoxic ischaemic encephalopathy with a bilateral dystonic motor disorder, Gross Motor Function Classification System (GMFCS) level V equivalent. Initial chest x-rays post resuscitation showed a normal spine; calcium levels were also normal. She was transferred to a specialist rehabilitation unit but was unfit for rehabilitation due to severe dystonia. 9 months post-injury she was transferred to our tertiary neurosciences unit for further management.

**CLINICAL MANAGEMENT:** Despite multiple anti-dystonia medications and a 24-hour postural management programme, tonal management remained extremely challenging, and she was often distressed and in pain with extreme extensor dystonic posturing, including her spine. We therefore sought an opinion on intrathecal baclofen (ITB) pump insertion. As part of the work up for ITB she had magnetic resonance imaging of spine which identified multilevel vertebral compression fractures, likely an ongoing source of pain contributing to dystonic posturing. Bone mineral density scan of lumbar spine revealed an age-matched Z-score: -3.22; bone mineral apparent density Z-score: -3.88. She was treated with intravenous zoledronate prior to transfer for ITB pump insertion.

**DISCUSSION:** As this child's history illustrates, rapid decline in bone health can occur following acquired brain injury, with multilevel vertebral compression fractures present 9 months post-injury in a previously fully ambulant child. This is a learning point for professionals involved in rehabilitation and management of children with severe movement disorders. We advocate early screening and consideration of vertebral compression fractures as a source of pain driving dystonia in this setting, since management with bisphosphonates allows opportunity to optimise rehabilitation potential and achieve best possible quality of life.

### P28

### A multipronged approach to pain management in osteogenesis imperfecta

Belinda Crowe[1], Emilie Hupin[1], Mark Heathfield[1], Penny Jones[2], Anita Freeman[1], Tracy Connolly[1], Zoe Bowman Bayles[1], Charlotte Barran[1], Alice Walker[1], Alistair Calder[3], Shobhit Verma[4], Jeremy Allgrove[1], Catherine DeVile[1]

[1]Osteogenesis Imperfecta Service, Great Ormond Street Hospital for Children NHS Foundation Trust, London, UK [2]Paediatric Physiotherapy, Luton and Dunstable University Hospitals NHS Foundation Trust, Luton, UK [3]Paediatric Radiology, Great Ormond Street Hospital for Children NHS Foundation Trust, London, UK [4]Paediatric Orthopaedics, Bedfordshire Hospitals NHS Foundation Trust, Luton, UK

### Abstract

**BACKGROUND:** Pain is a recognised feature of osteogenesis imperfecta (OI) which may be multifactorial and debilitating, having a significant negative impact on children's participation and quality of life. Since complex in nature, it often requires a multipronged, multidisciplinary team (MDT) approach for successful management.

**PRESENTING PROBLEM:** A 9-year-old boy with OI type 4 and a known *COL1A2* gene variant developed sudden worsening of pain affecting his left leg and groin. He had a history of two previous left femur fractures treated with plate and screw insertion and a resultant leg length discrepancy. He was also reporting intermittent back and shoulder pain, with normal vertebrae on spine X-ray. Pain was limiting his mobility and participation in activities, necessitating regular analgesics and increased wheelchair use.

**CLINICAL MANAGEMENT:** Since the precise cause of increased pain was unclear a multipronged management approach was utilised, including MDT assessment and completion of an F-words Goal Sheet to identify personalised goals. He identified he would like to participate in an upcoming residential school trip to an outdoor activity centre alongside his friends. He commenced zoledronate, completing the Adolescent Pediatric Pain Tool (APPT) and Wong-Baker FACES Pain Rating Scale prior to his first and second infusions. Results improved, with reduction in pain sites, pain intensity, and pain quality descriptor words selected, from 42% to 15%. We recommended ongoing strengthening activities, physiotherapy, and shoe raise use whilst awaiting femoral metalwork removal and intramedullary rod insertion locally. He was referred to our team psychologist for pain management input and he participated in our MDT HerOIc Pain Workshop. He subsequently attended his school trip, participating in archery, fencing, kayaking, orienteering, and enjoying a wonderful time.

**DISCUSSION:** Pain in osteogenesis imperfecta is complex and requires a holistic approach for effective management. Tools such as the APTT which is validated in OI and the F-words can be useful to assess pain location, intensity, and quality, monitor response to interventions and identify meaningful personalised goals. Multidisciplinary therapy team and psychosocial support are essential alongside medical and surgical options to ameliorate pain. A multipronged management approach is optimum to enable children to achieve their goals.

### P29

### Trabecular bone score and bone mineral density in children with asthma treated with inhaled corticosteroids

Siriyaporn Keeratisoontorn[1], Pemiga Sukhupanyarak[1], Yiwa Suksawat[2], Voraluck Phatarakijnirund[1]

[1]Division of Endocrinology, Department of Pediatrics, Phramongkutklao Hospital and College of Medicine, Bangkok, Thailand. [2]Division of Allergy, Department of Pediatrics, Phramongkutklao Hospital and College of Medicine, Bangkok, Thailand

### Abstract

**Objectives:**

To determine the effects of inhaled corticosteroids (ICS) on trabecular bone score (TBS) and bone mineral density (BMD) in prepubertal asthmatic children

**Methods:**

A cross-sectional was conducted in children with asthma, age 6-10 years, who received low to medium-dose ICS for at least for 3 months. TBS, lumbar spine (L2-L4) BMD (LS BMD) and body composition were measured by dual energy X-ray absorptiometry (DXA). TBS Z-score and LS BMD Z-score were calculated using reference values for children and adolescents

**Results:**

Twenty-five asthmatic children (13 male and 12 female) age 8.3 ± 1.3 years were included. Of them, 16% were overweight and 24% were obese but none of them were short. Most of the children (92%) were treated with fluticasone propionate or salmeterol/fluticasone propionate, duration of ICS treatment 2.9 ± 1.5 years, 80% received low dose and 20% received medium dose ICS and average dosage of ICS was 145.5 ± 58.1 mcg/m$^2$/day. No vertebral fracture was document from vertebral fracture assessment by DXA. The TBS Z-score was 1.4 ± 1.5 but 1 (4%) demonstrated partial degraded (TBS Z-score -1.4 SDS). Interestingly, 7(28%) have TBS Z-score >+2 SDS (3 had obesity). The LS BMD Z-score were normal (0.2 ± 1.0), however, 1(4%) had LS BMD Z-score < -2 SDS. Total bone mineral content (BMC) and total lean mass Z-score were normal in all subjects. There was no different in TBS Z-score, LS BMD Z-score, total BMC Z-score and body composition parameters between children received low and medium-dose of ICS. TBS Z-score was positively correlated with BMI Z-score (p 0.041, r 0.411). Total lean mass Z-score (p <0.001, r 0.647), total BMC Z-score (p 0.001, r 0.64), android/gynoid percent fat ratio (p 0.015, r 0.482), fat mass index (p 0.007, r 0.525) and lean mass index (p 0.022, r 0.456) were all positively correlated with LS BMD Z-score.

**Conclusion:**

Low to medium-dose ICS treatment in prepubertal asthmatic children may have adverse effects on bone health. Further research with a larger sample size is required and monitoring of TBS and BMD should persist throughout the critical period of bone mass accumulation in adolescents.

### P30

### Pilot project of bone health surveillance among children and adolescents at a local tertiary centre, Malaysia

Hooi Peng Cheng

Sarawak General Hospital, Kuching, Malaysia

### Abstract

**Objective:** To assess the bone health status among children and adolescents followed up by multidisciplinary team in Paediatrics Department, Sarawak General Hospital.

**Methods:** This is a prospective study conducted as the pilot project for the bone health surveillance among the at-risks children and adolescents in Pediatric Department, Sarawak General Hospital, Malaysia in January 2024. All the subjects will answer a bone health screening questionnaire, followed by physical examination and radiological investigation. Blood investigations will be traced from the electronic medical record.

**Results:** Fifteen subjects (6 males, 9 females) with the mean age of 10.6 ± 2.82 years were recruited in this study. Four subjects had primary bone disorders, while another 8 subjects (53.3%) had exposure to glucocorticoids for the management of various conditions including systemic lupus erythematous (2 subjects, 13.3%), juvenile dermatomyositis (2 subjects, 13.3%), juvenile idiopathic arthritis (2 subjects, 13.3%), ANCA vasculitis (1 subject, 6.7%) and acute lymphoblastic leukaemia (1 subject, 6.7%). Slightly more than half of the subjects had adequate dairy consumption and took vitamin D supplements in the form of cholecalciferol or alfacalcidol. 60% of subjects had sedentary lifestyle. Four subjects had physical stigmata of bone fragility disorders and had genetic confirmation of osteogenesis imperfecta. Five subjects (33.3%) had history of long bones fracture and also met the diagnosis of osteoporosis. Three of them had started on bone-active therapy with bisphosphonate. Serum 25-hydroxy vitamin D and parathyroid level were examined in 5 subjects and 2 subjects were detected to have vitamin D deficiency. Vertebral fractures were detected in 4 subjects (26.7%). The mean areal bone mineral density Z-scores were -2.91 ± 1.74 for hip, -2.05 ± 1.58 for lumbar spines and -3.23 ± 2.13 for total body less head.

**Conclusions:** Recognition of the importance of bone health management among the children and adolescents vulnerable to osteoporosis need to be emphasized.

| **Clinical parameters** | | **n (%)** |
|---|---|---|
| Age (years)* | | 10.6 ± 2.82 |
| Male | | 6 (40%) |
| Height Z-score* | | -2.01 ± 1.51 |
| BMI Z-score* | | -0.44 ± 1.81 |
| Discipline | Endocrine | 5 (33.3%) |

| | | |
|---|---|---|
| | Nephrology | 2 (13.3%) |
| | Rheumatology | 4 (26.7%) |
| | Haemato-oncology | 4 (26.7%) |
| Glucocorticoid usage | Yes | 8 (53.3%) |
| | No | 7 (46.7%) |
| Consumption of dairy products | Deficient | 5 (33.3%) |
| | Insufficient | 2 (13.3%) |
| | Adequate | 8 (53.3%) |
| Bone protection therapy | Calcium supplementation | 1 (6.7%) |
| | Vitamin D supplementation | 7 (46.7%) |
| | Bisphosphonate therapy | 3 (20%) |
| Sun exposure (minutes per week)* | | 248.67 ± 248.10 |
| Fitzpatrick skin phototype | Type II | 4 (26.7%) |
| | Type III | 5 (33.3%) |
| | Type IV | 2 (13.3%) |
| | Type V | 4 (26.7%) |
| Physical activity | Inadequate | 9 (60%) |
| | Adequate | 6 (40%) |
| History of fracture | Yes | 5 (33.3%) |
| | No | 10 (66.7%) |
| Physical stigmata of bone fragility condition | Yes | 4 (26.7%) |
| | No | 11 (73.3%) |
| Limb or spine deformity | Yes | 2 (13.3%) |
| | No | 13 (86.7%) |

| | | |
|---|---|---|
| GMFCS classification | Level I | 13 (86.7%) |
| | Level II | 2 (13.3%) |
| Pubertal staging | Prepubertal | 9 (60%) |
| | In puberty | 6 (40%) |
| Bone health surveillance | Bone profile | 15 (100%) |
| | 25-hydroxy vitamin D level | 5 (33.3%) |
| | PTH level | 5 (33.3%) |
| Vertebral fracture | Yes | 4 (26.7%) |
| | No | 11 (73.3%) |
| Bone densitometry* | Hip aBMD Z-score | -2.91 ± 1.74 |
| | Lumbar spine aBMD Z-score | -2.05 ± 1.58 |
| | TBLH aBMD Z-score | -3.23 ± 2.13 |

*Results were expressed as mean ± SD.

### P31

### Debilitating skeletal pain in an adolescent after renal transplantation. Differential diagnosis and treatment challenges. Report of a case

Artemis Doulgeraki[1], Georgia Malakasioti[2], Nick Sekouris[3], Smaragdi Marinaki[4], Georgios Polyzois[1], Varvara Askiti[2]

[1]Department of Bone and Mineral Metabolism, Institute of Child Health, Athens, Greece. [2]Department of Paediatric Nephrology, "P&A Kyriakou" Children's Hospital, Athens, Greece. [3]First Orthopaedic Department, "P&A Kyriakou" Children's Hospital, Athens, Greece. [4]Department of Nephrology and Renal Transplantation, National and Kapodistrian University of Athens, Laiko General Hospital, Athens, Greece

### Abstract

**Background:**

Paediatric patients with end-stage renal failure are candidates for renal transplantation. Their bone metabolism is already severely disturbed and they go through a stormy clinical period before and after the procedure. We report a case of a male adolescent who presented with osteoporosis and severe, debilitating ankle pain.

**Presenting problem:**

A 13.5 year-old patient, suffering from corticosteroid-resistant nephrotic syndrome since the age of 4 years, went on to develop focal segmental glomerulosclerosis and severe kidney failure, requiring renal transplantation, with his donor being his mother. Two months post operatively he was bound to a wheelchair, complaining of severe ankle pain, fatigue and occasional lower back pain.

**Clinical management:**

He had a full skeletal work up, which revealed osteoporosis, i.e. low bone mineral density (BMD, measured by DXA) and multiple vertebral fractures (Z-score BMD L1-L4= -3.5, Z-score BMD total body less head= -3.9). He also had MRI of the ankles; osteonecrosis was excluded. Additionally, his tacrolimus trough level was measured and was normal. His bone profile revealed low vitamin D and mild hypercalciuria. As there was relapse of proteinuria post-transplantation and, given that IV zolendronate is nephrotoxic, a "watch and wait" approach was followed. He is now on vitamin D supplements, regular analgesics and physiotherapy. As a result, he is now pain-free and walking. Depending on the condition of the renal transplant, oral bisphosphonates are being discussed as a safer option for his osteoporosis treatment.

**Discussion:**

Paediatric patients undergoing renal transplantation present with multiple predisposing factors for osteoporosis, e.g. pre-existing metabolic bone disorder, prolonged hospitalizations, drugs affecting the skeleton (e.g. steroids), hypogonadism etc. Skeletal pain in this scenario could be attributed to fractures, osteonecrosis or a painful syndrome due to calcineurin inhibitors (e.g. tacrolimus). Moreover, osteoporosis management with IV bisphosphonates may be challenging or even contraindicated. Paediatric guidelines for solid organ transplant recipients and bone health surveillance and treatment are urgently needed.

### P32

### Clinical manifestation of osteogenesis imperfecta type VI (mutation in SERPINF1 gene). A case report

Anna Maćkowska, Jakub Nowicki, Elżbieta Jakubowska-Pietkiewicz

Department of Paediatrics, Neonatal Pathology and Metabolic Bone Diseases, Medical University of Lodz, Lodz, Poland

### Abstract

**Background:**

Osteogenesis imperfecta (OI) is a group of diseases characterized by low bone mass and frequent fractures. The division into 4 types is based on clinical and radiological classification. In most cases, abnormalities in type I collagen synthesis caused by mutations in the COL1A1 or COL1A2 genes are responsible for OI. Additional types of OI caused by mutations in other genes are currently being distinguished. Type VI OI has been shown to be related with loss-of-function mutations in the SERPINF1 gene. Patients with type VI OI do not present symptoms of OI at birth, fractures appear after 6 months of age, and the number of fractures leads to delayed motor development. They usually do not present dentinogenesis imperfecta, blue sclera and hearing loss. Patients with type VI OI present a poor response to treatment with intravenous bisphosphonates.

**Presenting problem:**

The patient is a 7-year-old boy born at term, without features of OI at birth. At the age of 7 months, the patient developed his first fracture - a fracture of left femur. At the age of 18 months, due to further fractures and suspected OI, treatment with pamidronate sodium was administered. Genetic testing showed c.808G>T and c925_926delCA in a heterozygous pattern.

**Clinical management:**

Despite ongoing treatment, the patient still had fractures (3-10 yearly). To date, the child has suffered approximately 38 fractures. Due to the fractures, there has been a delay in the child's motor development. With the treatment administered, the number of fractures has decreased, but they still occur. Analyzing the DXA results in the Spine projection, the bone density is increasing, which correlates with fewer fractures in recent years. However, due to further fractures, it was decided to change the treatment to zoledronate.

**Discussion:**

Treatment of children with OI type VI is challenging. The literature describes that the efficacy of pamidronate treatment in these patients is poorer than in patients with mutations within the COL1A1/COL1A2 genes. There are also reports on the use of denosumab, zolendronate, including denosumab/zoledronate combination therapy. More research regarding efficacy and treatment options in OI type VI is needed.

**P33**

Withdrawn

### P34

### Osteogenesis imperfecta with bilateral congenital cataract: A rare presentation, first reported case in Sri Lanka

Melani Wijesuriya, Jananie Suntharesan

Sirimavo Bandaranayake Specialized Children's Hospital, Peradeniya, Sri Lanka

### Abstract

**Background:**

Osteogenesis Imperfecta (OI) is a connective tissue disorder primarily affecting bones, characterized by abnormal collagen due to mutations in *COL1A1* and *COL1A2*. While typically an autosomal dominant disorder, de novo mutations occur in 60% of cases. OI often presents with ocular manifestations, including blue sclera, hypertropia, myotropia, retinal detachment, reduced corneal rigidity, and glaucoma. This case report highlights a unique occurrence of type 3 OI in a neonate with bilateral congenital cataracts, marking the first reported case of its kind in Sri Lanka.

**Presenting problem and clinical management:**

A 20-day-old baby girl was referred due to multiple upper limb, lower limb, and rib fractures. Born at term via normal delivery to non-consanguineous parents, she weighed 2.6kg. The neonate required intubation and ventilation for neonatal sepsis-related pneumonia. Also treated for pulmonary hypertension with Sildenafil. Antenatal scans did not reveal intrauterine fractures. Clinical suspicion of type 3 OI led to treatment with IV Zoledronic acid and vitamin D. During the initial evaluation, bilateral cataracts were discovered and surgically removed. Subsequent follow-ups revealed recurrent fractures, poor weight gain, irritability, and poor muscle mass. Brain ultrasound indicated normal cortical sulci and gyri.

**Discussion:**

OI, often referred to as brittle bone disease, presents with multi-system manifestations, including short stature, abnormal development, hearing loss, and ocular complications. Blue sclera, a common ocular manifestation, results from scleral thinning and can lead to increased ocular pressure and retinal hemorrhage following minor trauma. While ocular complications, including cataracts, are documented in literature, this case represents the first reported instance in Sri Lanka of bilateral congenital cataracts in a patient with severe OI.

**Conclusion:**

Emphasizing the importance of ophthalmological assessments at diagnosis and annually, this case underscores the need for early detection and timely management to prevent visual impairment in children with OI. The rarity of this presentation in Sri Lanka highlights the significance of sharing such cases to broaden understanding and improve patient care.

### P35

### The complexity of osteoporosis among children and vertebral fractures as a shared sign of bone mineralisation disorders – 10 case reports

Anna Łupińska[1,2], Izabela Michałus[1], Sara Aszkiełowicz[1], Andrzej Lewiński[1,2], Arkadiusz Zygmunt[1,3], Renata Stawerska[1,2]

[1]Polish Mother's Memorial Hospital – Research Institute, Department of Endocrinology and Metabolic Diseases, Lodz, Poland. [2]Medical University of Lodz, Department of Pediatric Endocrinology, Lodz, Poland. [3]Medical University of Lodz, Department of Endocrinology and Metabolic Diseases, Lodz, Poland

### Abstract

**Background:** Osteoporosis continues to be an exceptionally rare diagnosis in pediatric patients. Bone mineralization disorders in children can either be primary (such as congenital bone fragility) or secondary to other diseases and their respective treatments. Among the most serious complications associated with low bone mass density are compression fractures of the vertebrae, which can result in significant pain, impaired mobility, and even disability.

**Presenting Problem:** The cases we present include 10 patients (5 girls and 5 boys) aged 6 to 16, who were referred to the Endocrinology and Metabolic Diseases Department of the Polish Mother's Memorial Hospital – Research Institute in Lodz, Poland. These patients were diagnosed with bone mineralization disorders complicated by compression fractures of the vertebrae. The etiology and symptomatology of these disorders exhibited considerable diversity.

Among the cases, 2 patients were diagnosed with Duchenne Muscular Dystrophy, 1 patient with Crohn's disease, in whom osteoporosis likely occurred secondary to steroid therapy. Six patients were identified with genetically predisposed disorders leading to osteoporosis: 1 with congenital bone fragility (COL1A1 gene mutation), 2 with LRP gene mutation, and 2 with SGMS2 gene mutation. Additionally, Cushing's disease was identified as the cause of multi-level vertebral fractures in one patient.

**Clinical Management:** Vertebral compression fractures were diagnosed via thoracolumbar spine X-rays in two projections. All patients underwent DXA examinations, indicating decreased bone mineral density. No significant disturbances in calcium-phosphate metabolism were observed in any on the patients, while vitamin D deficiency was identified in two patients. Eight patients agreed to "off-label" bisphosphonate therapy, significantly improving clinical status, including pain relief and enhanced bone mineral density.

**Discussion:** It is advisable, particularly in the pediatric population, to evaluate bone mineralization in every patient presenting with spinal pain complaints. In the case of identified decreased bone mineral density, radiological examination of the spine is essential to assess the presence of compression fractures of the vertebrae. Special attention should be given to patients requiring chronic steroid therapy for their underlying conditions. A comprehensive diagnostic approach is essential to confirm the exclusion of all possible causes of osteoporosis, including the most uncommon ones.

### P36

### Case study on the management of plagiocephaly in osteogenesis imperfecta (OI) type III

Nicola Panchbhaya[1], Lauren Rayner[2], Helen Collins[2], Rose Ferreira[1], Rebecca Doodson[2], Amish Chinoy[1], Raja Padidela[2]

[1]Royal Manchester Children's Hospital, Manchester, UK [2]Royal Manchester Children's Hospital, Manchester, UK

### Abstract

**BACKGROUND:**

Osteogenesis imperfecta (OI) is a lifelong genetic condition that affects type 1 collagen production impacting on both bone quality and strength. Plagiocephaly is recognised in infants with OI. Early intervention for cranial deformity is important because infants with OI spend extended periods in the supine position.

**PRESENTING PROBLEM:**

12 month old boy with Osteogenesis Imperfecta type III (heterozygous for COL1A1 variant), was commenced on pamidronate at 2 days of age, but was noted to have worsening plagiocephaly and brachiocephaly from 8 days of age. CT at 15 days old showed large acute subgaleal haemotoma and multiple skull fractures.

Patient head circumference was found to be in line with age but not weight (Table).

| Age (months) | Head Circumference (cm) | Z score head circumference | Weight (kg) | Z score weight |
|---|---|---|---|---|
| 0 | 33.4 | -1.3 | 2.66 | -2.0 |
| 1-2 | 34.5 | -3.7 | 2.80 | -4.9 |
| 13 | 45 | -1.0 | 3.76 | -8.5 |

Cranial technologies 2002 Severity scale for assessment of plagiocephaly was used and the patient was found to have a severity score of 12/15.

**CLINICAL MANAGEMENT:**

Multiple different modalities were explored by the metabolic bone disease therapists and medical team. There have been intensive contacts with multiple agencies including positioning advice from birth, use of Tortle hats and Leckey positioning system. The Tortle hat has a soft band down the affected side to prevent head turn to that side. The Leckey positioning system is widely used to aid positioning. On 12 month assessment the patient was noted to be delayed in fine and gross motor abilities, social behaviour and play. Multi-specialist centre discussion around appropriateness of helmet suggested this was not appropriate due to failure to thrive and difficulties this may cause for airway.

**DISCUSSION:**

This case highlights the importance of monitoring for plagiocephaly in infants with severe OI, and steps necessary with regards handling and positioning to minimise this. The literature on management of this in OI is scant, with some suggestion of helmets and cranial orthosis, which can prove difficult practically in such scenarios. More research is needed on head positioning in OI to support practice and prevent plagiocephaly/brachiocephaly. It is important to use objective measures for plagiocephaly and pictures to track any progression or decline.

### P37

### Radiofrequency Echographic Multi spectrometry (REMS) for the non-ionizing assessment of femoral neck bone health in a pediatric population

Giovanni Adami[1], Franco Antoniazzi[2], Carla Caffarelli[3], Sergio Casciaro[4], Antonio Colucci[5], Francesco Conversano[4], Angelo Fassio[1], Alessandro Ferruzzi[6], Davide Gatti[1], Stefano Gonnelli[3], Fiorella Anna Lombardi[4], Giorgia Luceri[5], Paola Pisani[4], Maurizio Rossini[1], Thomas Zoller[6]

[1]University of Verona, Policlinico GBRossi, Piazzale A. Scuro, 37134, Rheumatology Unit, Verona, Italy. [2]Pediatric Clinic C, Azienda Ospedaliera Universitaria Integrata, 37126, Verona, Italy. [3]Department of Medicine, Surgery and Neuroscience, University of Siena, Policlinico Le Scotte, Siena, Italy. [4]Institute of Clinical Physiology, National Research Council, Lecce, Italy. [5]R&D Department, Echolight SPA, Lecce, Italy. [6]Department of Surgical Sciences, Dentistry, Gynecology and Pediatrics, Pediatric Clinic, Verona University Medical School, Verona, Italy

### Abstract

*Note: all authors are equal contributors listed in alphabetic order*

### Objectives

Several biological and genetic factors affect bone growth in pediatric age; therefore, being able to perform bone screenings would be very important in the prevention of major diseases and also to assess bone growth compared to the global average. Actually, DXA is the technique able to get information about bone healthy of pediatric subjects. However, the use of ionizing radiation is an important risk factor in pediatric population, so clinical guidelines severely limit the use of DXA. Alternatively, ultrasound-based technologies can be used to investigate bone health status as Radiofrequency Echographic Multi Spectrometry (REMS) technology. REMS, as already reported in adult subjects, is able to assess bone microarchitecture and estimate fracture risk at proximal femur and lumbar spine. Exploiting the full capabilities of this technology, the aim of this work is the evaluation of bone health status on femoral neck in a pediatric population.

### Methods

In this preliminary study, a group of 82 pediatric subjects without sex distinction was unrolled (age 5-15 years) and underwent both REMS and DXA scans on femoral neck. Extending adult REMS models to the pediatric population, ultrasonic signal from these scans has been processed in order to test a new REMS-based algorithm to measure BMD and Z-Score of these subjects. REMS-based BMD was compared to DXA one by Pearson correlation coefficient and by statistical analysis to assess DXA-REMS concordance.

### Results

A significant correlation was found between REMS- BMD and DXA- BMD ($r=0.89$; $p<0.0001$) (fig. 1a), and between REMS-Z-score and DXA-Z-score ($r=0.84$; $p<0.0001$) (fig. 1b):

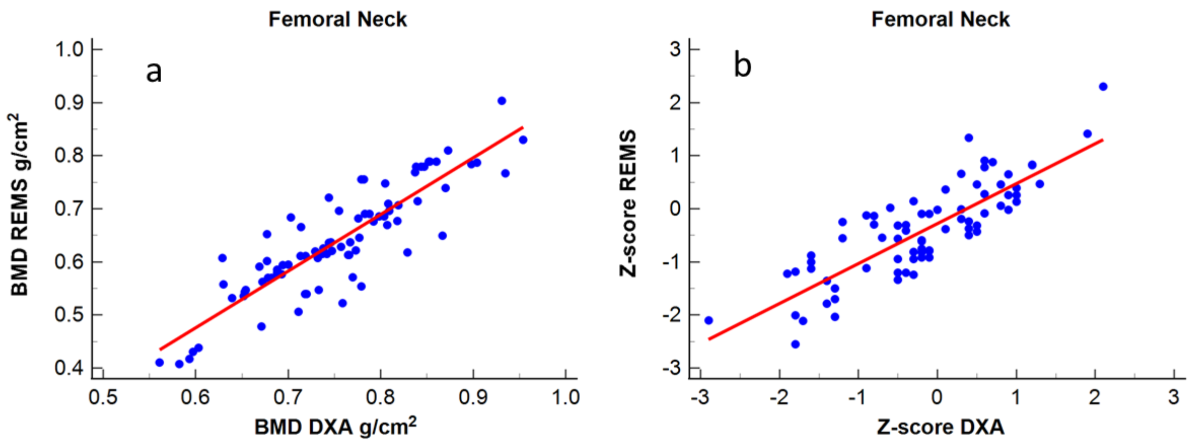

Similar results were obtained for all the age groups included in this study.

**Conclusions**

The results obtained from this study highlight REMS capability to properly investigate bone status also in pediatric subjects, with the major advantage of avoiding the use of ionizing radiation on this class of subjects.

### P38

### Does iron deficiency impair bone structure in early life?

Stephanie A Borg[1], Diane V Lefley[2], Jiabao Zhou[2], Nick Bishop[2]

[1]Sheffield Children's Hospital, Sheffield, UK [2]University of Sheffield, Sheffield, UK

### Abstract

### Hypothesis

Iron deficiency impairs bone architecture in early life

### Methods:

C57/BL6 dams received an iron replete control diet (Group 1) with their offspring remaining on this diet throughout. Group 2 dams received an iron replete diet and their offspring were weaned to an iron deficient diet. Group 3 received an iron deficient diet periconceptually supplemented with 2mg ferrous fumerate weekly and their offspring were weaned to an iron deficient diet for the duration of the study. Group 4 dams were placed on an iron deficient diet from day 14 post conception with their offspring weaned to a replete diet. All offspring were weaned aged 25-28 days.

Whole blood collected in EDTA tubes was analysed using a Scil Vet ABC+. Plasma PTH was measured by ELISA.

MicroCT analysis was performed on vertebrae (age 4 weeks mice only) and tibia trabecular and mid cortical regions.

### Results:

For offspring aged 4 weeks the Hb was lower in all groups compared to those exposed to iron replete diet only (Group 1, replete: 15.6 ±2.3 g/dL; Group 2: 12.37±3.1 g/dL; Group 3: 8.2±4.6 g/dL; Group 4: 11.2±3.3 g/dL all $p<0.05$). At end of study (aged 11-12 weeks) the Hb remained low in Group 2 and 4 only (14.37±1.3; 9.03±2.1*; 14.0±2.0; 9.24±2.7*; *$p<0.001$).

Serum PTH did not differ between offspring of groups 1 and 2 aged 4 weeks (292.1±177.8ng/ml vs 267.1±135.1 ng/ml).

Analysis of the vertebrae showed no significant results between the two groups.

Table 1 microCT parameters measured in tibia cortex and trabecular regions of offspring at age 4 weeks from groups 1 and 3 and at 11/12 weeks from each group. All comparisons are within own age group and compare to control (Group 1) *$p<0.05$; **$p<0.01$;***$p<0.001$

| Group/Parameter | Group 1 | Group 3 | | Group 1 | Group 2 | Group 3 | Group 4 |
|---|---|---|---|---|---|---|---|
| Age (weeks) | 4 | 4 | | 11 | 11 | 11 | 11 |
| Cortical BV $\mu m^3$ | 0.51±0.1 | 0.55±0.0 | | 0.9±0.1 | 0.9±0.1 | 0.69±0.1** | 0.68±0.04 |
| Cortical BV/TV % | 26.4±3.1 | 29.7±2.5* | | 35.1±6.1 | 35.1±4.6 | 31.7±2.5 | 29.3±4.9 |
| Trabecular BV $\mu m^3$ | 0.05±0.0 | 0.06±0.0* | | 0.2±0.0 | 0.16±0.01* | 0.23±0.04 | 0.16±0.03* |

| | | | | | | | |
|---|---|---|---|---|---|---|---|
| Trabecular BV/TV % | 6.1±0.7 | 8.6±1.3*** | | 18.8±2.3 | 12.8±4.9** | 15.8±3.1 | 13.4±1.9* |
| Trabecular number /µm | 2.3±0.2 | 3.1±0.4* | | 5.4±0.4 | 3.3±1.3* | 4.6±0.9* | 3.9±0.6*** |
| Porosity | 93.9±0.7 | 91.4±1.3*** | | 81.2±3.2 | 87.3±4.9* | 84.2±3.1 | 86.7±1.6*** |

**Conclusions:**

Adult mice exposed to early life iron deficiency demonstrated reduced cortical and trabecular bone architecture parameters. In contrast young mice exposed to early life iron deficiency demonstrated unexpected increases in cortical and trabecular microarchitecture parameters. These data do not support the hypothesis that early life iron deficiency contributes to bone fragility in infancy.

### P39

### Tracking bone density and growth dynamics in children with Gaucher disease: A longitudinal study of treatment impacts

David Strich[1,2], Hanan Adler Adler[2], Mayan Tomkin[3], Shoshana Revel-Vilk[3,2], Ari Zimran[3,2], Aya Abramov[3], Udi Lebel[4,2]

[1]Pediatric Endocrinology Unit, Shaare Zedek Medical Center, Jerusalem, Israel. [2]School of Medicine, Hebrew University, Jerusalem, Israel. [3]Gaucher Unit, Shaare Zedek Medical Center, Jerusalem, Israel. [4]Department of Orthopedic Surgery, Shaare Zedek Medical Center, Jerusalem, Israel

### Abstract

**OBJECTIVES**:

Gaucher disease (GD), a rare autosomal recessive disease, is caused by variants in the glucosylceramidase beta 1 (*GBA1*) gene. The disease is manifested by the accumulation of glucosylceramide within lysosomes in macrophages in visceral organs and bone marrow, which leads to hepatosplenomegaly, anemia, thrombocytopenia, growth delay, and variable skeletal features that include events of pain (bone crises), osteoporosis/osteopenia, and osteonecrosis. There is variability in the extent and severity of bone involvement in GD.

In children with GD, the need to start enzyme replacement therapy (ERT) is tailored to the individual because of the variability in the manifestations, severity, progression of the disease, and treatment response. The aim of this study was to study longitudinal changes in the Whole body less head, adjusted to height-for-age Z-score (WBLH) and Height-for-age Z score (HAZ) in association with treatment status.

**METHODS**:

Children and adolescents (6-20 years) with GD who had a minimum of two dual-energy x-ray absorptiometry (DXA) scans were included in the study. These scans, done every 2-3 years with a Hologic Discovery densitometer, were part of regular clinic visits.

The study cohort included 76 children and adolescents (40 males) with a median (range) follow-up of 3.8 (1.4-11.2) years; 36 were never treated, 29 received enzyme replacement therapy (ERT) all along, and 11 started ERT during follow-up.

**RESULTS**:

At baseline, the lowest WBLH and higher rate of short stature were noticed in those untreated at baseline but started ERT during follow-up. At the end of the follow-up, all groups had similar WBLH and prevalence of short stature (Table 1). The change in WBLH was not associated with sex, *GBA1* variants, follow-up time, or baseline parameters, such as age, WBLH, or HAZ. The change in HAZ was associated with baseline HAZ but not with sex, *GBA1* variants, baseline age, and follow-up time.

**CONCLUSIONS**:

Early initiation of ERT in those who necessitate it can effectively normalize WBLH Z-score and enhance height. Not all children require treatment for maintaining normal bone density and height. However, regular follow-up is needed. This underscores the importance of tailored treatment approaches in children with GD.

Table 1: Characteristics of subjects who were untreated, received enzyme replacement therapy (ERT) already at baseline, or started ERT during follow-up.

| **Baseline/<br>End of Study** | **Untreated /<br>Untreated** | **Treated /<br>Treated** | **Untreated /<br>Treated** | **p-value** |
|---|---|---|---|---|
| *Number* | 36 | 29 | 11 | |
| *Male* | 20 (55.6%) | 15 (51.7%) | 5 (45.5%) | 0.8 |
| *Mild genotype* | 32 (89%) | 1 (3.4%) | 4 (36%) | <0.001 |
| *Age at baseline** | 10.5 (6-14.5) | 11 (6-17) | 9.5 (8.5-18) | 0.5 |
| *Follow-up time, years** | 5 (1.6-11) | 4.4 (1.4-11) | 2.2 (1.8-10) | 0.03 |
| ***Whole body less head, adjusted to height-for-age Z-score*** | | | | |
| *Baseline** | -1 (-2.3 to 0.7) | -1.5 (-2.8 to -0.2) | -1.3 (-2.2 to 0.3) | 0.081 |
| *End of study** | -0.7 (-3.4 to 1) | -0.7 (-2.6 to 0.2) | -0.8 (-3.2 to 1.6) | 0.872 |
| ***Short stature, Height-for-age Z score < -2*** | | | | |
| *Baseline* | 1 (2.8%) | 6 (20.7%) | 4 (36.4%) | 0.01 |
| *End of study* | 1 (2.8%) | 2 (6.9%) | 2 (18.2%) | 0.2 |

*median (range)

### Disclosure

S.R.-V. reports receiving grant/research support, honoraria, and advisory fees from Takeda, Pfizer, and Sanofi/Genzyme; A.Z. reports receiving honoraria from Takeda, Pfizer, and Bio-events, being a consultant for Takeda and having ownership at Agyany Pharma Ltd.

### P40

### Longitudinal assessment of DXA and pQCT derived bone health parameters in children and youth with type 1 diabetes

Shruti Mondkar[1], Neha Kajale[1,2], Keyur Desai[3], Vivek Patwardhan[1], Anuradha Khadilkar[1,2], Chirantap Oza[1], Sharvani Patil[1], Chidvilas More[1], Alex Ireland[4], Vaman Khadilkar[1,2]

[1]Hirabai Cowasji Jehangir Medical Research Institute, Jehangir Hospital, Pune, India. [2]School of Health Sciences Savitribai Phule Pune University, Pune, India. [3]Adhikari Lifeline Hospital, Boisar, India. [4]Research centre for Musculoskeletal science and sports medicine, Department of life science, Manchester Metropolitan University, Manchester, UK

### Abstract

**Objectives:** 1) Describing longitudinal changes in bone health over a 4-year period in children and youth with type-1 Diabetes (T1D); 2) Characterizing bone health based on duration of diabetes.

**Methods:** Longitudinal, observational study (4years). Clinico-demographic, anthropometric, biochemical, Dual Energy Xray Absorptiometry (DXA) at lumbar spine and total-body less-head(TBLH) sites, peripheral quantitative computed tomography at 4% and 66% radius (pQCT) both height-adjusted parameters were assessed at baseline and at 4years.

**Results:** 200 participants [diabetes duration <5years (group-A) n=160, >5years (group-B) n=40], mean age at baseline and diabetes duration were 10.6±3.9; 3.2±3.2years respectively. Significant differences between baseline and endline were observed in HbA1c (9.6±2.1vs10.1±2.2%), body fat percentage Z-scores (-1.0±1.3vs-0.5±1.3), lean mass Z-scores (1.0±1.1vs0.4±1.2) and insulin sensitivity (10.2±3.1vs8.6±2.7 mg/kg/min).

DXA-derived TBLH BMD Z-scores (-1.4±1.1vs-1.7±1.2) and pQCT-derived trabecular vBMD (168±36vs150±40 g/cm3) and Z-scores (-0.9±1.5; -1.7±1.2) at 4% radius were significantly lower after 4 years. There was a significant increase in cortical vBMD (1044±64; 1098±59 $g/cm^3$) at 66% radius and significant reduction in cortical thickness (3.1±0.6; 1.8±0.3mm). Increase in height, weight and BMIZ-scores correlated positively with increase in TBLHBMD Z-score and trabecular vBMD Z-score. Change in total vBMD Z-score correlated negatively with HbA1c change(r=-0.3).

None of the participants had fractures at baseline. 1 subject from group-A and 4 from group-B had history of low impact fractures over 4 years. Insulin sensitivity, lean body mass(LBM), TBLHBMD and L1-L4 BMD Z-scores were significantly lower at both, baseline and endline in group-B. Significant increase in fat Z-scores and reduction in LBM, TBLHBMD, total vBMD Z-scores, muscle density, cortical thickness were noted in both groups over 4 years. A significant reduction in trabecular vBMD (4% radius) and strength strain index Z-scores was noted in group-A. Significantly more reduction in total vBMD, muscle area for height and fat area for height Z-scores was noted in group-B. Diabetes duration was significantly associated with LSBMAD Z-score and pubertal status with cortical vBMD Z-score change.

**Conclusions:** A progressive decline in DXA-derived TBLH BMD, pQCT-derived trabecular vBMD, with cortical bone thinning (in respect of typical growth) was observed in children and youth with T1D which were chiefly influenced by diabetes duration and pubertal status.

| Age | Group A (Baseline) 10.0 (3.7) | Group B (Baseline) 13.3 (3.1) | Group A (4 years) 14.1 (3.7) | Group B (4 years) 17.5 (3.2) |
|---|---|---|---|---|
| Diabetes duration (years) [A,B,C,D] | 1.8 (1.5) | 8.4 (2.8) | 4.8 (1.9) | 12.5 (2.9) |
| Height Z score [A,B] | -0.6 (1.0) | -1.3 (1.4) | -0.6 (1.0) | -1.3 (1.1) |
| Weight Z score | -0.7 (0.9) | -0.8 (0.9) | -0.4 (0.9) | -0.8 (1.0) |
| BMI Z score [A] | -0.5 (0.8) | -0.2 (0.8) | -0.2 (0.9) | -0.3 (0.8) |
| Insulin dose (U/kg/day) [A] | 0.9 (0.3) | 1.1 (0.2) | 1.0 (0.3) | 1.1 (0.3) |
| Insulin sensitivity (mg/kg/min) [A,B,C,D] | 10.4 (3.2) | 8.9 (2.3) | 8.8 (2.7) | 7.4 (2.4) |
| HbA1c (%) [C] | 9.6 (2.0) | 9.9 (2.2) | 10.1 (2.1) | 10.2 (2.4) |
| Creatinine (mg/dL) | 0.7 (0.1) | 0.7 (0.2) | 0.7 (0.2) | 0.8 (0.2) |
| eGFR (mL/kg/1.73m2) [C] | 76.3 (21.1) | 84.9 (19.1) | 90.6 (21.9) | 89.0 (22.3) |
| Calcium (mg/dL) | 9.4 (2.4) | 8.9 (0.6) | 9.3 (0.4) | 9.3 (0.4) |
| Phosphorus (mg/dL) [B] | 4.5 (0.8) | 4.6 (0.7) | 3.9 (0.5) | 3.6 (0.5) |
| Alkaline phosphatase (IU/L) | 257.5 (141.0) | 309 (213.9) | 208.0 (96.2) | 212.5 (102.9) |
| 25-Hydroxy Vit D (ng/mL) | 15.7 (10.5) | 13.4 (5.9) | 18.8 (9.2) | 18.8 (9.1) |
| Parathormone (pg/mL) | 57.7 (37.2) | 63.3 (28.2) | 47.6 (25.6) | 40.5 (16.2) |
| Fat % Z [C,D] | -1.0 (1.3) | -0.6 (1.2) | -0.6 (1.3) | -0.3 (1.0) |
| LBM % Z [A,B,C,D] | 1.2 (1.1) | 0.6 (0.9) | 0.6 (1.3) | 0.2 (0.6) |
| L1-L4 BMD (g/m2) [A] | 0.677 (0.165) | 0.749 (0.169) | 0.847 (0.200) | 0.896 (0.167) |
| L1-L4 BMD Z score [A,B] | -1.2 (0.8) | -1.7 (1.2) | -1.2 (0.9) | -1.6 (1.0) |
| LSBMAD (g/cm3) [C,D] | 0.246 (0.038) | 0.259 (0.039) | 0.276 (0.047) | 0.282 (0.041) |
| LSBMAD Z score | -1.1 (1.2) | -1.0 (1.2) | -1.0 (1.7) | -1.0 (1.5) |
| TBLH BMD (g/cm2) | 0.619 (0.148) | 0.691 (0.142) | 0.739 (0.152) | 0.763 (0.119) |
| TBLH BMD Z score [A,B,C,D] | -1.4 (1.1) | -1.6 (1.3) | -1.6 (1.1) | -2.1 (1.5) |
| Trabecular vBMD 4% (g/cm3) [C] | 166.62 (35.22) | 171.59 (45.21) | 146.08 (37.45) | 162.66 (46.67) |
| Cortical vBMD (g/cm3) 66% | 1042.12 (60.62) | 1062.90 (79.31) | 1078.25 (63.98) | 1085.79 (83.31) |
| Total vBMD (g/cm3) [A,B] | 268.56 (46.55) | 298.23 (45.28) | 287.30 (52.92) | 328.13 (80.01) |
| Cortical thickness (mm) [A,C,D] | 2.98 (0.73) | 3.23 (0.58) | 1.67 (0.37) | 1.76 (0.52) |
| Periosteal circumference (mm) | 34.39 (3.89) | 34.63 (3.22) | 35.77 (3.9) | 36.01 (4.01) |
| Endosteal circumference (mm) | 24.82 (3.89) | 24.21 (3.66) | 25.25 (3.77) | 24.93 (4.12) |
| Polar SSI 66% | 149.7 (54.5) | 155.111 (54.2) | 170.964 (62.1) | 173.911 (65.307) |
| Trabecular density for age Z score [C] | -0.9 (1.5) | -0.9 (1.4) | -1.6 (1.2) | -1.4 (1.4) |
| Cortical density for age Z score | -0.0 (1.3) | -0.9 (2.1) | -0.0 (1.6) | -0.5 (1.7) |
| Total density for age Z score [C,D] | -0.6 (1.4) | -0.0 (1.5) | -1.0 (1.0) | -0.9 (1.4) |
| SSI Pol for age Z score [C] | -1.3 (0.8) | -1.7 (0.7) | -1.5 (0.9) | -1.7 (1.1) |
| Muscle area for height Z score [A] | -2.3 (1.1) | -1.9 (0.9) | -2.1 (1.1) | -1.9 (0.9) |
| Bone area for height Z score [C,D] | -1.6 (0.8) | -1.4 (1.0) | -1.3 (0.9) | -0.9 (0.8) |
| Bone for muscle Z score [D] | 0.2 (1.1) | -0.1 (1.5) | 0.4 (1.1) | 0.6 (1.4) |

A- Significant difference between groups A & B at baseline
B- Significant difference between groups A & B at endline
C- Significant difference between baseline & endline in group A
D- Significant difference between baseline & endline in group B

### P41

### Tertiary hyperparathyroidism in two children with X-linked hypophosphatemia during Burosumab treatment

Raja Padidela[1,2], Lauren Rayner[1], Annemieke Boot[3]

[1]Royal Manchester Children's Hospital, Manchester, UK [2]Manchester Academic Health Science Centre, Faculty of Biology, Medicine and Health, The University of Manchester, Manchester, UK [3]Beatrix Children's Hospital - University Medical Center Groningen, Groningen, Netherlands

### Abstract

**Introduction**: Although secondary hyperparathyroidism is known in X-linked hypophosphatemia (XLH), tertiary hyperparathyroidism with hypercalcemia is rare, especially in children. We present two paediatric XLH patients treated with Burosumab who developed this rare complication.

### Case Descriptions

**1:** A female patient with XLH on conventional therapy (phosphate and active vitamin D) since one year of age was switched to Burosumab at 10 years (1.2-1.4 mg/kg/dose, initially 4-weekly then 2-weekly). At that time PTH was 7.9 pmol/l, serum calcium 2.60 mmol/l. She developed tertiary hyperparathyroidism with hypercalcaemia 4.3 years after commencing Burosumab, PTH 22.6 pmol/l, calcium 2.92 mmol/l. Post-parathyroidectomy her hypercalcaemia resolved (PTH 6.3 pmol/, calcium 2.52 mmol/l) and 4 years post-surgery her calcium levels continue to remain normal.

**2:** This female patient with XLH on conventional therapy since 4 months of age was switched to 2-weekly Burosumab (0.55 mg/kg/dose) at 4 years of age. At that time PTH was 4.0 pmol/l, calcium 2.34 mmol/l. At 7 years of age, she developed secondary hyperparathyroidism (PTH 9.8 pmol/l, calcium 2.65 mmol/l, Burosumab dose of 1.85mg/kg/dose). Within 6 months she developed tertiary hyperparathyroidism with hypercalcaemia (PTH 11.8 pmol/l, calcium 2.96 mmol/l). Burosumab was discontinued at 7.5 years of age, and she was commenced on Cinacalcet but, hypercalcaemia failed to resolve. Post-parathyroidectomy her tertiary hyperparathyroidism resolved (PTH 1.9 pmol/l, calcium 2.25 mmol/l). However, within 2 weeks, PTH increased to 14.7 pmol/l (calcium 2.36 mmol/l) which normalised with Cinacalcet. Burosumab has been recommenced and she continues cinacalcet. 25OHD levels had been in the normal range in both patients.

**Discussion:** The cause of tertiary hyperparathyroidism is not clear in these patients. Patient 1 had marginally elevated PTH for a few years before the development of hypercalcemia, notably, this was more acute in patient 2. Higher post-dose phosphate levels (not measured) or the direct effect of *PHEX* mutation on the gland could have triggered PTH secretion.

**Conclusion:** XLH patients treated with Burosumab can develop hyperparathyroidism and should be regularly monitored for the development of secondary and tertiary hyperparathyroidism.

**Disclosure**

Prof Raja Padidela and Dr Annemieke Boot have research support and honorarium from Kyowa Kirin International. Ms Lauren Rayner has received an honorarium from Kyowa Kirin International

### P42

### Characterization of rickets at diagnosis: Preliminary data of an Italian multicenter cohort

Alessia Angelelli[1,2], Natascia Di Iorgi[1,2], Nadia Maiorano[1], Domenico Corica[3], Malgorzata Wasniewska[3], Roberto Franceschi[4], Matilde Ferrario[5], Danilo Fintini[6], Giulia Mirra[6], Mila Ann Kalapurackal[7], Marco Pitea[8], Ruggero Lanzafame[8], Daniele Tessaris[9], Elisa Bonino[10], Andrea Secco[11], Enrica Bertelli[12], Anastasia Ibba[13], Anna Grandone[14], Francesco Vierucci[15], Maria Felicia Faienza[16], Mario Giordano[16]

[1]D.I.N.O.G.M.I., University of Genoa, Genoa, Italy. [2]Pediatric Endocrinology Unit, Genoa, Italy. [3]Department of Human Pathology of Adulthood and childhood "G.Barresi", University of Messina, Messina, Italy. [4]Pediatric Unit, S. Chiara Hospital, Trento, Italy. [5]Pediatric Endocrinology Unit , Ospedale dei Bambini "Vittore Buzzi", Milano, Italy. [6]Endocrinology and Diabetology Unit "Bambino Gesù" IRCCS, Roma, Italy. [7]Pediatric division, Ospedale Regina di Bolzano, Bolzano, Italy. [8]Pediatric Endocrinology, IRCCS S. Raffaele, Milano, Italy. [9]Pediatric Endocrinology Unit, Regina Margherita Children's Hospital, University of Turin,, Torino, Italy. [10]University of Turin, Torino, Italy. [11]Pediatric Unit, Azienda ospedaliero Nazionale SS Antonio e Biagio e Cesare Arrigo, Alessandra, Italy. [12]Pediatric Unit, Azienda ospedaliero Nazionale SS Antonio e Biagio e Cesare Arrigo,, Alessandra, Italy. [13]Pediatric endocrinology, Ospedale Pediatrico Microcitemico "A. Cao", Cagliari, Italy. [14]Department of Woman, Child and of General and Specialized Surgery, Università degli Studi della Campania "Luigi Vanvitelli", Napoli, Italy. [15]Pediatria di Lucca, Lucca, Italy. [16]Unit of Endocrinology and Rare Endocrine Diseases, University of Bari "Aldo Moro", Bari, Italy

### Abstract

**Objectives**: To characterize a large cohort of patients with Rickets(R).

**Methods**:

Retrospective multicenter study including 164 patients (85 Females, 79 Males) diagnosed with rickets between 0-19 years in 13 centres throughout Italy. According to clinical, biochemical and genetic data, they were classified as a) hypocalcemic-R(n=81), furthermore stratified into nutritional-R, secondary-R, and genetic-R, or b) hypophosphatemic-R (n=83). According to age at diagnosis, patients were further divided into <2yrs (n=78) and 2-19yrs (n=85).

**Results:**

Nutritional-R was prevalent in hypocalcemic-R (90,1%) while PHEX-mutation in hypophosphatemic-R (91,5%). Mean age at diagnosis of hypocalcemic-R and hypophosphatemic-R was 3.7±0.3yrs and 4.2±0.5yrs, respectively ($p=0.09$). In the hypocalcemic-R, genetic-R patients(n=4) were the youngest (mean age 1.1-0.2yrs). An early diagnosis (<2 yrs) was performed in 44/79 (55.7%) of hypocalcemic-R and in 35/79 (44.3%) of hypophosphatemic-R.

The phototype was fair in 88(53.7%, red), intermediate in 48(29.3%, green) and dark in 28 cases(17%, blue): differences in the distribution between hypocalcemic-R and hypophosphatemic-R were evident (P<0.05)(Figure1).

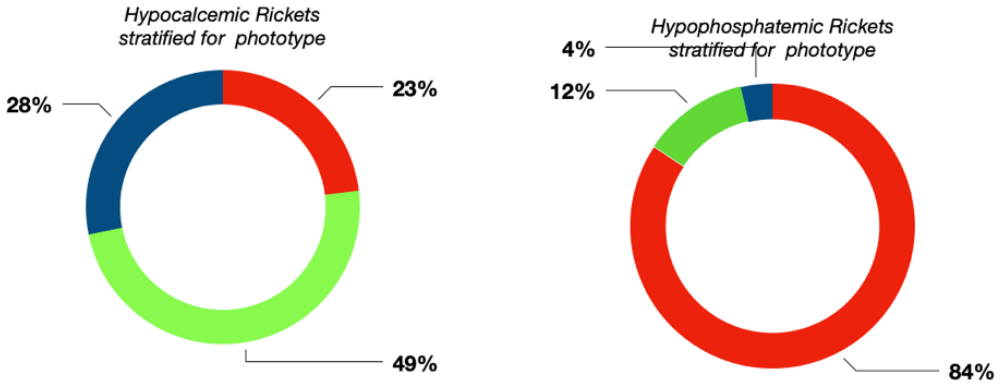

**Figure 1**

Short length (CDC-2009, <5°percentile) was found in 32% of patients<2years (n=15 hypocalcemic, n=10 hypophosphatemic) and 28.2% between 2-19 years (n=8 hypocalcemic, n=16 hypophosphatemic). Mean stature was -1.0±0.2SDS and -1.7±0.1SDS in subjects with hypocalcemic-R and hypophosphatemic-R (p=0.05).

Symptoms/signs were available for 153/164 subjects (93.3%): globally, 138/153(90.2%) had at least one at presentation. The prevalence in hypocalcemic/hypophosphatemic-R were, respectively: skeletal deformities 66.6/72.3%, functional limitations 28.4/22.9%, pain 19.8/12%, craniosynostosis 2.5/7.3% and fractures 4.9/1.2%. In the hypocalcemic-R cohort tetany and convulsions were present in 9.9% and in 11.1%, respectively. Among the 37 subjects who underwent ECG(33 with symptoms), 12 had abnormal results(32.4%), including 1 asymptomatic.

**Conclusions**:

Preliminary data show a ratio of 1:1 between hypocalcemic/hypophosphatemic-R. Dark/intermediate phototype was prevalent in nutritional-R, while fair in the PHEX-mutation group. Age at diagnosis was similar in the cohorts; however, only 44% of PHEX-mutations were diagnosed before 2 years of age. Deformities were frequent and comparable in the two groups, short stature was present in ¼ of patients with rickets regardless of age, in particular in the hypophosphatemic-R cohort. In hypocalcemic-R, tetany and craniosynostosis were observed in about 1:10 while ECG abnormalities in 1/3 of the patients evaluated.

### P43

### Assessment of gut microbiome profile and bone status in Indian children with type 1 diabetes - A pilot study

Sharvani Patil[1], Vaishali Tamahane[1,2], Nikhil Shah[1], Shruti Mondkar[1], Neha Kajale[1,2], Vivek Patwardhan[1], Anuradha Khadilkar[1], Chirantap Oza[1], Chidvilas More[1], Yogesh Shouche[3], Alex Ireland[4], Vaman Khadilkar[1,2]

[1] Hirabai Cowasji Jehangir Medical Research Institute, Jehangir Hospital, Pune, India. [2] School of Health Sciences Savitribai Phule Pune University, Pune, India. [3]National Centre for Microbial Resource - National Centre for Cell Science, Pune, India. [4]Research centre for Musculoskeletal science and sports medicine, Department of life science, Manchester Metropolitan University, Manchester, UK

### Abstract

**Objectives:** The gut microbiome influences bone metabolism and bone remodelling through the immune system. Dysbiosis results in intestinal inflammation and is associated with bone destruction in disorders such as type 1 diabetes mellitus (T1DM), especially when poorly controlled. Our aim was to study gender-specific associations of the gut microbiome profile with bone-muscle parameters in children with T1DM.

**Methods:** 68 (boys=28) children with T1DM were recruited for a cross-sectional, observational, pilot study. Clinical and anthropometric measurements were performed. The GE-Lunar i-Dual Energy X-ray absorptiometer (DXA) fan-beam DXA scanner (software version encore18) was used to measure total body less head bone mineral content [TBLHBMC(g)], total lean mass [TLM(g)], total fat mass[TFM(kg)] and appendicular lean mass[ALM(kg)]; the appendicular skeletal mass index[ASMI(kg/$m^2$)] was computed. Stool samples were collected, the gut-microbiome was analysed using 16s-rRNA sequencing technique. Statistical analysis was performed using IBM-SPSS-26 software.

**Results:** The mean age of boys(girls) was 12.5±2.5yrs(12.9±2.9yrs), hbA1c was 9.4±1.5%(10.0±2.0%), weight was 38.8±10.4kg (39.6±11.7kg), disease duration was 5.1±2.6yrs(5.4±2.9yrs) respectively. The relative abundance of *Olsenella* (mean-rank=38), *Paraprevotella* (mean rank=37) and *Slackia* (mean rank=37) were significantly higher ($p$<0.05) in boys and lower in girls while the relative abundance of *Monoglobaceae* and *Monoglobus* was higher in girls(mean-rank=37) and lower in boys(mean-rank=25). TBLHBMC adjusted for height was significantly higher in boys (980±329g) than girls(947±313g).Total fat mass was significantly higher in girls(12.9±5.8kg) than boys(9.6±4.9kg) ($p$<0.05 for all). ALM and ASMI were similar in the two groups.

*Fusobacteriia*(r=0.27) showed positive while *Butyricimonas*(r=-0.3), *Lachnospiraceae_UCG006*(r=-0.3) and *Paludicola*(r=-0.28) showed significant negative correlation with height adjusted TBBMCLH ($p$<0.05). *Sutterellaceae*(r=0.25) showed positive while *Fenollaria*(r=-0.26), *Lachnospiraceae_UCG006*(r=-0.28) and *Paludicola*(r=-0.25) showed negative association with TLM. *Gammaproteobacteria*(r=0.3), *Gemella*(r=0.3), *Neisseria*(r=0.3), *Solobacterium*(r=0.29), *Proteobacteria*(r=0.3) showed significant (p<0.05) positive association while *Euryarchaeota*(r=-0.25) showed negative association with TFM. *Sutterellaceae*(r=0.25) and *Neisseria*(r=0.25) showed positive while *Butyricimonas*(r=-0.27), *Lachnospiraceae_UCG006*(r=-0.27), Paludicola(r=-0.25) showed negative correlation with ALM. *Butyricimonas*(r=-0.25), *Fenollaria*(r=-0.25), *Lachnospiraceae_UCG006*(r=-0.27), *Paludicola*(r=-0.25) showed significant negative while *Neisseria*(r=0.25) showed positive association with ASMI.

**Conclusion:** Significant and moderate association exists between the gut microbiome profile and bone-muscle parameters in children with T1DM. Future studies are needed to explore this association and the possibility of optimising the gut microbiome for improvement of bone-muscle health.

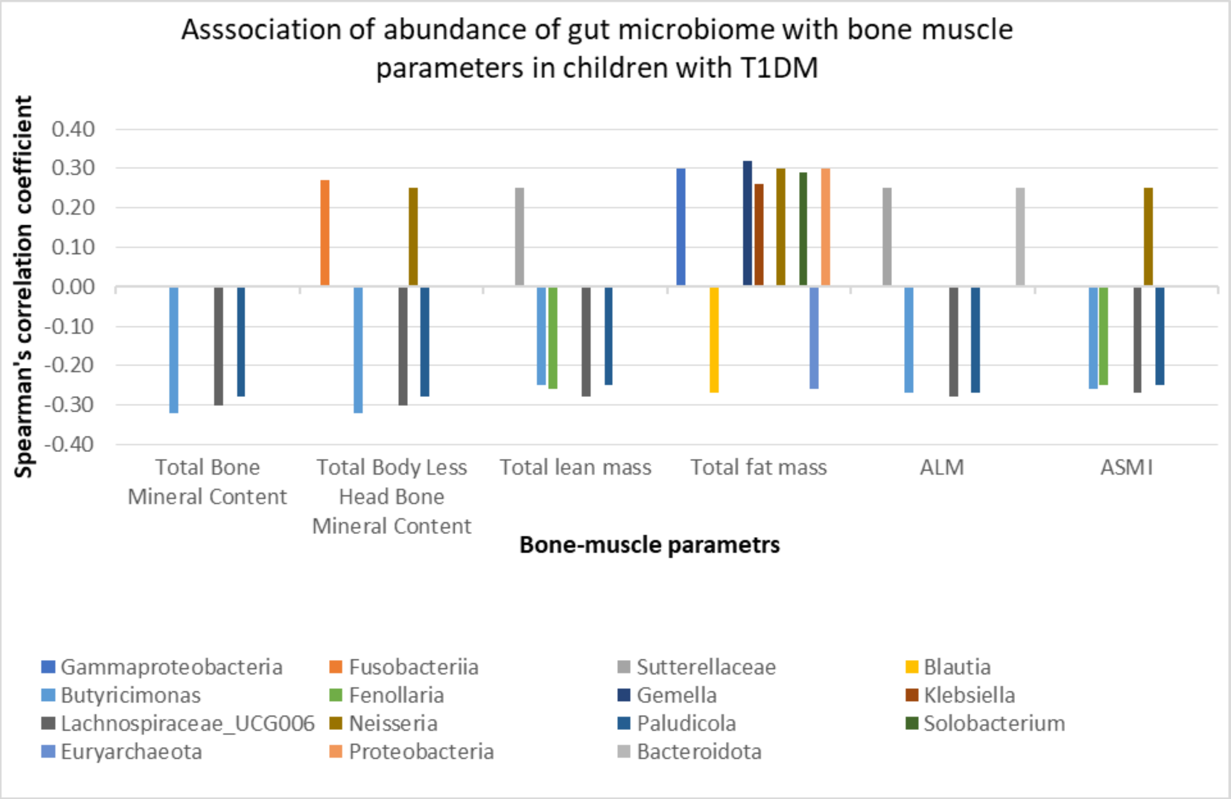

### P44

### Characterisation of hypocalcemic seizures secondary to vitamin D deficiency in a tertiary centre

Nalini M Selveindran

Hospital Putrajaya, Putrajaya, Malaysia

### Abstract

**OBJECTIVES:**

The epidemiology of symptomatic Vitamin D deficiency (VDD) in children in Malaysia is largely undetermined, limited to local case reports. This study aimed to describe the clinical characteristics and risk factors of inpatient admissions due to severe hypocalcemia and vitamin D 25-OH deficiency at a tertiary referral hospital.

**METHODS:**

Medical records were reviewed on all inpatient admissions from January 2022 to January 2024 for children aged 0 – 18 years of age with hypocalcemic seizures with a corrected calcium < 2.0mmol/l and a Vitamin D level < 20ng/ml.

**RESULTS:**

Nine patients satisfied the inclusion criteria. All patients presented before the age of 2 years with majority of the patients presenting before the age of 2 months (56%). Seven patients (77%) presented with episodes of multiple seizures. Seizure duration was generally brief, however in two patients (22%) seizures lasted more than 10 minutes. Four patients (44%) also had signs of rickets. Two patients (22%) had restrictive diets and five patients (55.5%) were exclusively breastfed. Investigation results revealed a serum Vitamin 25-OHD level of < 10nmol/l in 78% of patients. Where available, 25-OH-D levels were less than 25nmol/L in 75% of mothers. All patients were admitted to hospital with a median duration of 3 days [ 2–5 days]. None of the patients had any underlying medical condition. All patients were given acute treatment to terminate the seizure or prevent recurrence with two (22%) requiring anticonvulsant therapy. Three patients (33%) received alphacalcidiol. All patients were started on cholecalciferol therapy and oral calcium supplementation.

**DISCUSSION**

Hypocalcemic seizures is a more severe manifestation of Vitamin D deficiency. At present there is a lack of public health policy on prevention of Vitamin D deficiency in children. Further studies are required to evaluate the epidemiology of symptomatic VDD more broadly in order to guide future public health decisions.

### P45

### Type 1 diabetes – a low bone turnover disease: A study of Swedish adolescents with well-controlled long-duration (10 years) type 1 diabetes

Diana Swolin Eide[1,2], Auste Pundziute Lyckå[2], Daniel Novak[2], Björn Andersson[3], Gun Forsander[2], Per Magnusson[4]

[1]University of Gothenburg, Gothenburg, Sweden. [2]Region Västra Götaland, Sahlgrenska University Hospital, Gothenburg, Sweden. [3]Bioinformatics and Data Center, Sahlgrenska Academy, Gothenburg, Sweden. [4]Department of Clinical Chemistry, and Department of Biomedical and Clinical Sciences, Linköping University, Linköping, Sweden

### Abstract

**OBJECTIVES:** Type 1 diabetes (T1D) increases worldwide and is associated with many severe complications, including increased fracture risk. The aim of this case-control study was to investigate if young individuals with long-duration T1D have decreased bone mass and how bone biomarkers reflect bone remodeling in relation to healthy controls.

**METHODS:** Fifty individuals, 15.0-17.9 years old, with a T1D duration of mean 10.6±2.1 years were included. Thus, these individuals had diabetes through the entire puberty and growth spurt. Mean HbA1c since diabetes diagnosis was 57±6 mmol/mol (7.37±0.55%). Bone mass and body composition were assessed by DXA and pQCT.

**RESULTS:** Clinical follow-up data were retrieved from the National Diabetes Registry (NDR). The groups were well-matched with no differences between the groups regarding age, weight, height, body mass index (BMI) and amount of physical activity. Fourteen individuals with T1D had 16 fractures, and 18 control subjects had sustained 19 fractures before study start. Total body less head (TBLH) aBMD and TBLH BMC were significantly lower in T1D individuals. Lumbar spine L1-L4 BMD and BMC showed no differences between the groups. According to pQCT, total tibia density and trabecular density were reduced in the T1D group, $p=0.01$. Although BMI-matched groups, T1D individuals had higher total body fat mass and higher trunk fat mass ($p<0.05$). Parathyroid hormone, 25(OH)D, bone-specific alkaline phosphatase (BALP), type I procollagen intact N-terminal propeptide (PINP), sclerostin, bioactive sclerostin and osteoprotegerin did not differ between the groups. However, individuals with T1D had reduced C-terminal telopeptide cross-links of collagen type I (CTX) and nuclear factor κB ligand levels indicating altered bone resorption. Positive correlations were observed between diabetes duration and the bone biomarkers PINP, BALP, osteocalcin and CTX.

**CONCLUSIONS:** In summary, young individuals with well-controlled long-duration T1D have already impaired bone microstructural changes at several sites and decreased bone resorption, which indicate a low bone turnover disease already in young age. It is important to monitor bone health early on in pediatric diabetes care is for fracture prevention.

### P46

### Management of low vitamin D levels prior to the bisphoshonate infusion in patients with osteoporosis

Diana Suhbat[2], Philippa Prentice[1], Robyn Gilbey-Cross[1], Emma Snow[1], Sophie Emery[1], Alessandra Cocca[1]

[1]Evelina London Children's Hospital, London, UK [2]University of Jordan, Amman, Jordan

### Abstract

### Objectives

In our hospital, patients with osteoporosis on treatment with bisphosphonates, have blood tests prior to the infusion, to check vitamin D and calcium (a maximum of 3 months earlier for vitamin D and 1 month for calcium).

Vitamin D should be >50 nmol/L to minimise the risk of post-infusion hypocalcaemia.

If these levels were lower than the desired cut-off, our practice was that patients would receive either oral or IM Vitamin D supplementation but we would wait at least one month before repeating bloods and, if normal, we would proceed with the infusion.

This caused delays in patients' treatments and higher workload for the team who needed to organize additional patient's appointments.

Aim of this study was to evaluate whether a single IM stoss Vitamin D dose and blood tests repeated shortly after, on the morning of the bisphosphonate infusion, would prevent us from delaying patients' treatments.

### Methods

We included 15 osteoporosis patients booked to receive either a first (11/15, 73%) or following (4/15, 27%) bisphosphonate infusion.

5/15 (33.33%) had Duchenne Muscular Dystrophy, 5/15 (33.33%) Osteogenesis Imperfecta type 1, 5/15 (33.33%) other causes. Mean age was 13.3 +/- 2.8 SDs.

Mean vitamin D prior to the infusion was 43.4 (nmol/L) +/- 8.5. Corrected calcium was 2.3 (mmol/L) +/-0.09.

2/15 patients (13%) had vitamin D <39; 3/15(20%) between 40-44 and 10/15 (67%) between 45-49.

### Results

Only 4 patients had vitamin D repeated post stoss dose, on the morning of the bisphosphonate infusion. Mean vitamin D was 106.25 +/- 6.8.

Interestingly, the patients with the lowest vitamin D of our cohort (16 and 34), had a vitamin D of respectively 97 and 105 after the stoss therapy.

None of the patients reported hypocalcaemia symptoms the week after bisphosphonate infusion. Only 3/15 (20%) reported flu-like symptoms which we would expect after the infusion.

**Conclusions**

Results are reassuring as, prior to this change, infusions were being delayed due to the need to start oral vitamin D supplementation and repetition of bloods after 1 month.

Further studies with larger number of patients (which will be available by June) are necessary to confirm that this practice is safe and effective.

### P47

### Positive association between Serum Alkaline Phosphatase and Hba1c in Saudi children

Nasser Al-Daghri, Danish Hussain, Shaun Sabico

King Saud University, Riyadh, Saudi Arabia

### Abstract

**Objective:**

Previous studies have linked serum alkaline phosphatase levels and the risk of diabetes, but reported controversial results in adult population. It has also been reported that serum alkaline phosphatase has increased activity in growing children. Therefore, this study aims to observe association between serum alkaline phosphatase and hba1c.

**Method:**

This cross-sectional study was performed between September 2019 and March 2021. Adolescents aged 12–17 years from 60 different secondary and preparatory year schools in Riyadh, Saudi Arabia were included.

**Results:**

A total of 2,666 adolescents participated in this study with mean age of 14.9 ± 1.7. There were 1601 girls and 1065 boys. The overall prevalence of diabetes was 4.6% (n=122/2666) and was higher in boys than girls but no significance was observed (5.3% vs. 4.1%, $p = 0.169$). Preliminary analysis showed that there is a significant positive association between alkaline phosphatase (ALP) and Hba1c in adolescent with correlation coefficient of 0.10 (P<0.001). Furthermore, similar positive association was also observed with bone specific alkaline phosphatase (BLP) and hba1c with correlation coefficient of 0.12 (P=0.020). Furthermore, median ALP concentration i.e. 78.2 (55.0 – 108.5) was higher in in Diabetic children as compared to non-diabetic children with median ALP concentration of 63.0 (42.6 – 93.0) (p<0.001).

**Conclusion:**

There is a positive association between alkaline phosphatase and hba1c in children aged 12-17 years. Poor blood glucose control can cause a negative impact on bone mass acquisition at an early age, as these children and adolescents are developing height, along with the progression of diabetes. Diabetes during pubertal growth will foster a deficiency in bone mass gain, thus achieving a bone mass peak smaller than that of healthy individuals.

### P48

### Vertebral Fracture Assessment (VFA) is a useful diagnostic technique for children at risk of low trauma vertebral compression fractures

Jenny Anscombe[1], Tahera Elwell[1], Ruchi Nadar[1], Suma Uday[1,2], Vrinda Saraff[1,2], Nicola Crabtree[1,2]

[1]Birmingham Women's and Children's NHS Trust, Birmingham, UK [2]Institute of Metabolism and Systems Research, University of Birmingham, Birmingham, UK

### Abstract

### Objectives:

Identifying one or more vertebral compression fractures (VF) in the absence of local disease or high energy trauma is pivotal in the diagnosis of osteoporosis in children. In 2019 the International Society of Clinical Densitometry ratified the use of VFA by dual-energy X-ray absorptiometry (DXA) in children. The objective of this study was to audit the use of VFA in a tertiary paediatric hospital.

### Methods:

A clinical audit of VFA was performed on new patient referrals to the bone density service, between January and December 2023. All children had DXA, pQCT and VFA performed as standard. Additionally, a bone health questionnaire was completed collecting data on back pain, self-reported long bone fractures, current medication, and pubertal status. Clinical follow-up was investigated in children reported to have VF.

### Results:

165 new patient referrals had bone density assessments over the 12-month period. Mean age was 12.0 (4.0-17.9) years. VFA was performed in 160 (97%) children and 5 were not scanned due to spine rodding. Ten VFAs were excluded from analysis (6= non-diagnostic due to marked scoliosis, 4 = VFs were considered pathological due to known Chronic non-bacterial osteomyelitis). A total of 103 VFs [52 Mild, 35 Moderate, 16 Severe] were identified in 25 out of 150 children. Treatment with bisphosphonates was initiated in 12 [48%] children, in 10 [40%] children a “watch and wait” approach was recommended and 3 [12%] are undergoing further investigations. Reported daily back pain was highest in those with the most severe fractures [60%]. However, no difference in reported back pain was noted in children with mild or moderate VFs versus those with no VFs (13% vs 11%). Average bone density was reduced in children with VFs, compared to no VF [BMAD Z-score: (0.0 (1.3) vs -1.2 (2.0), p<0.001| pQCT Radius Z-Score: (-0.2 (1.7) vs -1.6(1.3)), p<0.001].

### Conclusion:

VFA by DXA can be successfully integrated into bone health assessment as it provides actionable information for consideration of treatment. Whilst bone density can be considered a significant risk factor for vertebral fractures, back pain for all except those with the most significant VFs, is a relatively ineffective marker.

### P49

### Association of radiogrammetry-derived bone health indices with DXA-measured bone density in children - A pilot study

Sharvani Patil[1], Pranay Goel[2], Chirantap Oza[1], Shruti Mondkar[1], Neha Kajale[1,3], Vivek Patwardhan[1], Anuradha Khadilkar[1,3], Chidvilas More[1], Raja Padidela[4], Alex Ireland[5], Vaman Khadilkar[1,3]

[1]Hirabai Cowasji Jehangir Medical Research Institute, Jehangir Hospital, Pune, India. [2]Indian Institute of Science Education and Research (IISER), Pune, India. [3]School of Health Sciences Savitribai Phule Pune University, Pune, India. [4]Department of Paediatric Endocrinology & Metabolic Bone Diseases, Royal Manchester Children's Hospital, Oxford Road, Manchester M13 9WL, UK [5]Research centre for Musculoskeletal science and sports medicine, Department of life science, Manchester Metropolitan University, Manchester, UK

### Abstract

### Objectives:

Assessment of bone density is essential in children especially in disorders where the bones are known to be affected. Dual-energy x-ray absorptiometry (DXA) is the gold standard for accurately measuring bone density in children and adolescents. However, using DXA for assessment of bone health in low- to middle-income countries (LMIC) like India, especially by people from the lower economic strata presents challenges in terms of affordability, accessibility, and methodological complexity. Radiogrammetry (a method for measuring bone mineral density using hand X rays), on the other hand, may prove to be a cheaper and more easily accessible alternative for screening for poor bone health. Thus, the objectives of our pilot study were 1) To study the association of different radiogrammetry-derived bone health indices with DXA-measured height adjusted TBLHBMC in children. 2) To identify the radiogrammetry index that is most closely associated with DXA derived height adjusted TBLHBMC.

### Methods:

This was an observational, cross-sectional pilot study, n=30 (mean age10.5±4.1years). The GE-Lunar iDXA (encore software – version 16) was used to measure TBLHBMC; X-rays of the non-dominant hand were performed on Skanray Technologies (microSKAN-HF diagnostic X-ray system, Telangana, India). An experienced radiologist and a paediatrician manually measured cortical thickness[T], cortical width[W], and cortical length[L] using a Radiopaque X-ray Lead Ruler. These values were used to calculate various bone health indices using following methods-including 1) Cylindrically symmetrical bone method [$A=\pi TW(1-T/W)$ and $A=\pi TW$, 2) Cortical thickness method [$DXR=cT(1-T/W)$], 3) Exton-Smith Index [$ESI=A/(WL)$], and the 4) Metacarpal index method [$MCI= A/W^2$]. Correlation (Spearman) analysis was performed between bone health indices and height adjusted TBLHBMC.

### Results:

There was a moderately strong significant positive correlation reported between DXA measured height adjusted TBLHBMC and $A=\pi TW(1-T/W)$ (rs=0.545, $p$=0.002), MCI (rs=0.619, $p$<0.001) and ESI (rs=0.454, $p$=0.012) respectively. Cortical thickness method derived indices did not show a positive relation with DXA parameters.

**Conclusion:**

Preliminary results show a significant relationship between bone health indices (highest with MCI and the cylindrically symmetrical bone method) and DXA measured height-adjusted TBLHBMC in children. Further studies into the use of radiogrammetry for screening for poor bone health are required for resource-limited settings.

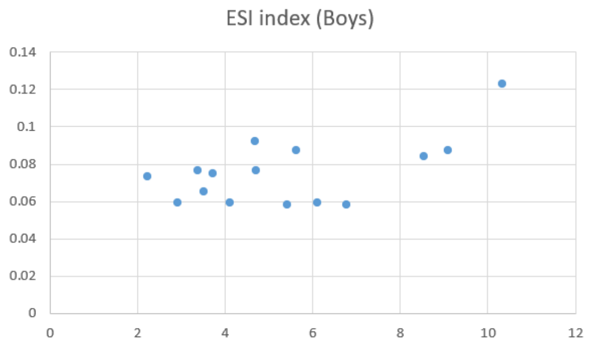

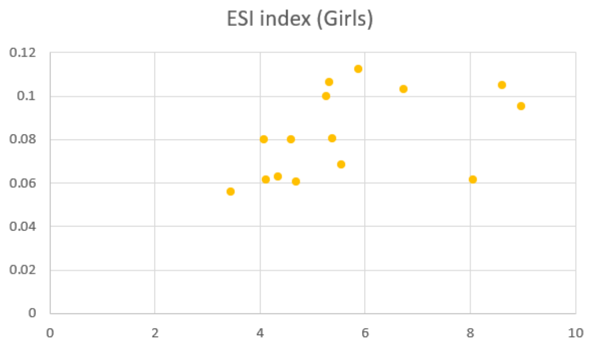

Fig1: Height Adjusted TBLHBMC and ESI Index correlation

### P50

### Reference data for bone density and geometry data on Indian children and adolescents (7-19 years) using peripheral quantitative computed tomography

Neha Kajale[1,2], Vivek Patwardhan[1], Anuradha Khadilkar[1,2], Chirantap Oza[1], Shruti Mondkar[1], Sharvani Patil[1], Chidvilas More[1], Raja Padidela[3], Alex Ireland[4], Vaman Khadilkar[1,2]

[1]Hirabai Cowasji Jehangir Medical Research Institute, Jehangir Hospital, Pune, India. [2]Interdisciplinary School of Health Sciences Savitribai Phule Pune University, Pune, India. [3]Department of Paediatric Endocrinology & Metabolic Bone Diseases, Royal Manchester Children's Hospital, Oxford Road, Manchester, UK [4]Research centre for Musculoskeletal science and sports medicine, Department of life science, Manchester Metropolitan University, Manchester, UK

### Abstract

**Objectives:** Peripheral quantitative computed tomography (pQCT) measures bone density (volumetric bone density- trabecular and cortical), bone geometry, and bone strength non-invasively. There is a lack of ethnic-specific reference data for the pQCT for children from the Indian subcontinent; we thus aimed to provide age- and gender-specific reference pQCT data on healthy Indian children and adolescents at the distal and mid-shaft radius.

**Method:** pQCT (XCT-2000 scanner, Stratec, Inc., Pforzheim, Germany) was used to measure the 4% and 66% sites of the non-dominant radius cross-sectionally among 961 children (560 boys) healthy Indian children and adolescents (aged 7 – 19 years). Gender-specific reference centile curves were constructed using the lambda, mu, sigma (LMS) technique. Also, LMS tables were produced for calculating age and gender-specific standard deviation scores.

**Results:** The mean age of participants was 13.6± 4.0 years. Overall, total bone density at 4% radius and cortical thickness at 66% radius were similar among boys and girls ($p>0.05$). However, means of trabecular density (186 ± 35 vs. 168 ± 41 mg/cm3), cortical density (1033 ± 65 vs. 1060 ± 77 mg/cm3), polar strength strain index (pSSI- 199 ± 88 vs. 156 ± 51 mm3), periosteal (38 ± 4.8 mm vs. 35 ± 3.1 mm), and endosteal circumference (28 ± 4.1 vs. 25 ± 3.5 mm) were significantly different ($p<0.05$) among boys and girls. All these parameters exhibited age-wise increments in both genders. Trabecular vBMD at 18 years was higher in boys, and the increase from 7-8 years was also higher (7.5% vs. 3.5%). At 18 years pSSI almost doubled in boys (186%) compared with (87%) after 18 years, indicating higher bone strength among boys than girls. Conversely, mean cortical density was significantly higher in girls than boys ($p<0.05$). Further, age- and gender-specific reference centiles for all parameters were generated.

**Conclusion:** This study presents ethnic, gender and age-specific reference centiles for parameters of bone density and geometry at the radius. These data may be used as references for clinical interpretations among younger populations with or without pediatric bone diseases.

Polar Strenght Strain Index
SSIPOL (mm3)
320.00
270.00
220.00
170.00
120.00
70.00
8
10
12
14
16
18
20
Age ( years)
Boys
Girls

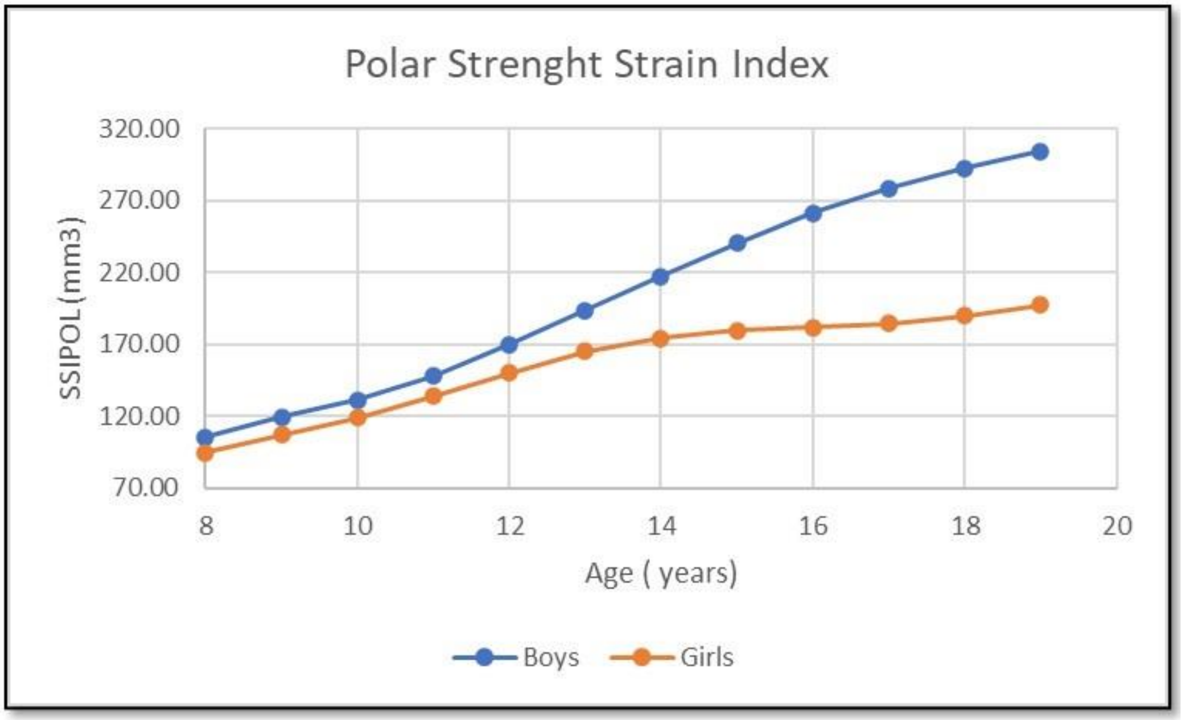

### P51

### Muscle and Bone assessment using peripheral quantitative computed tomography in childhood Acute Lymphoblastic Leukaemia

Madhura Joshi[1], Komal Doal[1], Liana Yusof[2], Jayashree Motwani[2], Wolfgang Högler[3,4], Nicola Crabtree[1], Vrinda Saraff[1]

[1]Department of Endocrinology, Birmingham Women's and Children's Hospital, Birmingham, UK [2]Department of Haematology, Birmingham Women's and Children's Hospital, Birmingham, UK [3]Institute of Metabolism and Systems Research, University of Birmingham, Birmingham, UK [4]Department of Paediatrics and Adolescent Medicine, Johannes Kepler University Linz, Linz, Austria

### Abstract

**Objectives:** Acute Lymphoblastic Leukaemia (ALL) is the most common childhood cancer. Both the disease process and the use of glucocorticoid therapy reportedly affect bone health resulting in osteoporosis. Peripheral quantitative computed tomography (pQCT) is an alternative technique to dual-energy X-ray absorptiometry (DXA) that provides detailed information on limb bone geometry as well as muscle size. Our objective was to evaluate bone health in children with ALL early in the disease process, using pQCT, DXA and vertebral fracture assessment (VFA).

**Methods:** Retrospective cross-sectional analyses of children with ALL aged > 5 years over an eight year period, using pQCT, DXA and VFA performed within 12 weeks of diagnosis. pQCT was performed at the distal and diaphyseal sites of radius. Data included age at diagnosis, type of ALL (B and T cell) and bone structural parameters from pQCT and DXA. Vertebral fractures (VF) were assessed by DXA VFA.

**Results:** Forty-eight children, 24 (50%) female, with ALL were identified, with a mean age 10.6 (range 6.1-16.4) years of which 41 (85 %) had B-cell ALL. VF (>20% height loss) were noted in 7 (14.5%) patients. Z-scores for total body muscle area for height (-2.80±1.50), lumbar spine bone mineral apparent density (LS BMAD) (-0.47±1.39) and total body less head bone density (TBLH BMD) (-0.41±1.01) were all less than zero ($p<0.05$). PQCT-derived Z-scores for total (0.89±1.57) and trabecular density for age (1.17±1.80), fat area for height (1.26±1.72), bone area for height (0.60±1.53) and bone area for muscle area (1.98±1.52) were all greater than zero ($p<0.05$). Cortical density Z-scores (-0.21±1.62) was not different from zero. Significantly lower LS BMAD Z-scores were identified in children with confirmed VF vs those without [-1.8 (1.9) vs -0.2 (1.1), p = 0.001].

**Conclusion:** We report significant reduction in muscle mass (sarcopenia) and smaller reduction in LS BMAD and TBLH BMD measured values but higher than expected total and trabecular density, and bone area within 12 weeks of ALL presentation. The rate of VFs was as expected. Overall, the results imply that the deterioration in muscle mass due to the disease and therapy-related immobility occurs faster than in bone mass.

### P52

### Reference curves for Bone Health Index derived from hand radiographs, in twelve populations, representing five ethnicities across four continents

Hans Henrik Thodberg[1], Peter Thrane[1], David Martin[2], Miranda-Lora América Liliana[3]

[1]Visiana, Hoersholm, Denmark. [2]Universität Witten/Herdecke, Witten, Germany. [3]Hospital Infantil de México Federico Gómez, Mexico City, Mexico

### Abstract

**Objective**: To present reference curves for the Bone Health Index (BHI) versus bone age for twelve different populations representing five ethnicities across four continents.

**Methods**: BHI was determined as part of the BoneXpert method for autonomous bone age determination from digital hand X-rays. BHI is derived from the cortical thickness T in the three middle metacarpals using the bone width W and length L to adjust for the highly variable size of the pediatric population: $BHI = A/(LW)^{0.33}$, where $A = \pi TW (1–T/W)$ is the transverse cortical area. BHI was designed to obtain the lowest relative variation (7.5%) at a given bone age and gender.

**Results**: Data were collected from published study data, e.g. a study from Pune by Oza et al 2023, and from a new study in Mexico. All data were prospective studies of children representative of the normal populations. Caucasian data were obtained from the Netherlands, Switzerland, Johannesburg and Los Angeles (LA); east-Asians from Japan, China and LA; Hispanics from Mexico and LA; blacks from Johannesburg and LA; and south-Asians from Pune in India.

The reference curves displayed approximately the same shape, and the variation between populations could be described well by a scaling factor for each population, common for the two genders. The scaling factor was defined to be 100% on average for the four LA populations. The observed scaling factors are shown in the figure, ranging from 95 to 103%, with the exception of 87% for Pune.

**Conclusions**: (1) The average BHI was remarkably similar across the populations with the dramatic exception that Indian children had 13% lower BHI than LA children. Fracture risk (as surveyed by Kanis et al 2012) is however not elevated in India, so the lower BH level is normal and “healthy” and should be taken into account when computing BHI Z-scores (aka BHI SDS)

(2) Radiogrammetry is effective to study variations of (size-corrected) bone mass across many sites, due to the simplicity of recording X-rays, and the objective reading by BoneXpert.

(3) The BHI reference curves have clinical value for computation of population-adjusted Z-scores for children aged 0-19.

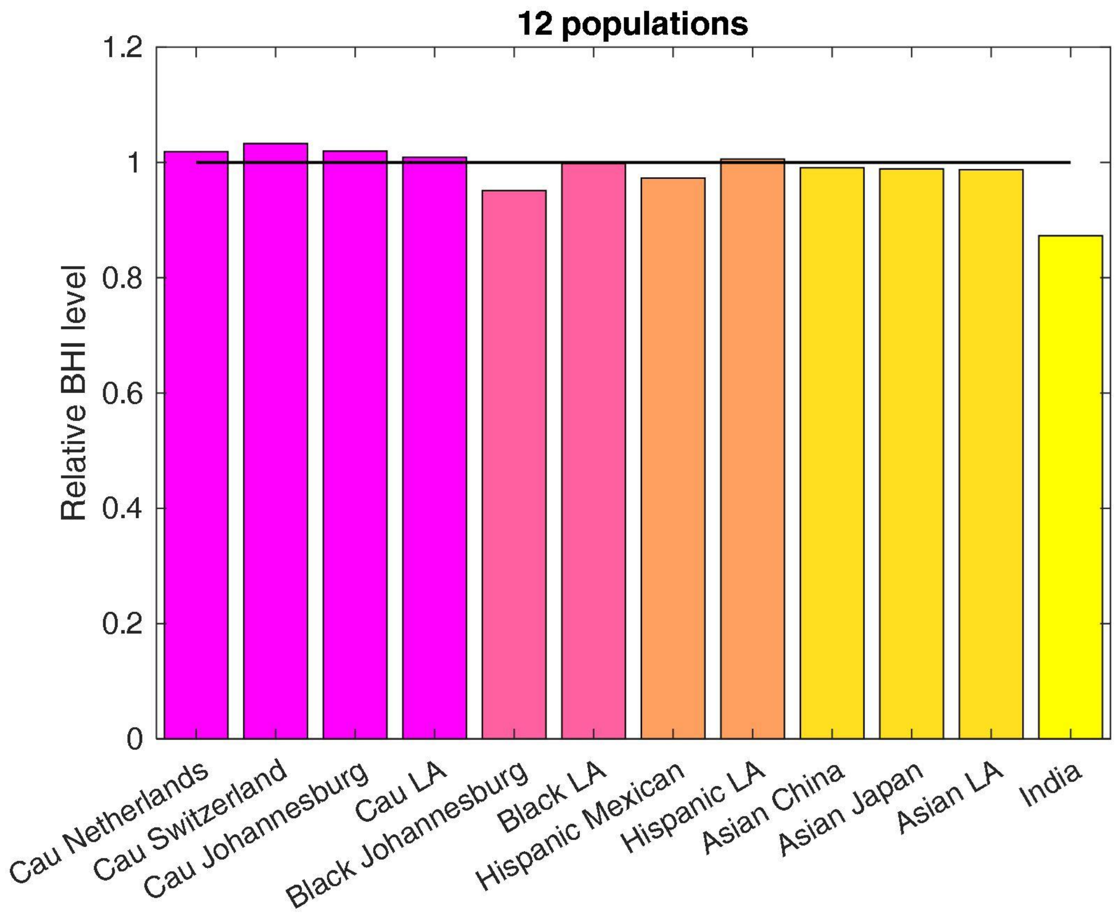

**Disclosure**

HHT is the director and owner of Visiana, which develops and markets the BoneXpert medical device. PT is employed in Visiana. The two other authors have none declared

### P53

### Low bone mineral density and osteoporosis in Cleidocranial dysplasia

Philippa Prentice, Rui Santos, Rhoda Akilapa, Alessandra Cocca

Evelina London Children's Hospital, London, UK

### Abstract

**Objectives:**

Cleidocranial dysplasia (CCD) has an incidence of around 1 in 1,000,000. It is a skeletal dysplasia, typically presenting with hypoplastic or aplastic clavicles, wide pubic symphysis, short middle phalanx of the fifth fingers, delayed closure of the cranial sutures, dental abnormalities and vertebral malformations. It is increasing recognised that CCD can be associated with low bone mineral density (BMD) and osteoporosis. Screening with a dual energy X-ray absorptiometry (DEXA) scan is recommended, particularly in adolescence. However, there are no consensus guidelines. We aimed to review our current practice and contribute to recommendations.

**Methods:**

All current CCD patients, at a tertiary referral centre, were identified and a case note review conducted. Information collected included DEXA scan measurements, evidence of vertebral fractures, by vertebral fracture assessment (VFA) or spine x-ray, and treatment with the bisphosphonate, zoledronic acid. The definition of osteoporosis was low BMD (<-2 SD) and 2 long bone fractures, or vertebral fractures alone.

**Results:**

27 patients were identified, 18 of whom had at least one DEXA scan performed. Average height was -1.0 +/- 1.1 SD. A first DEXA scan was carried out at 9.2 +/- 2.8 (5.2 – 13.2) years. Results were: lumbar spine (LS) -1.8 +/- 1.6 SD; whole body less head (WBLH) -1.7 +/- 1.0 SD.

4 children had osteoporosis (22%), all due to vertebral fractures, with an average age of 10 (6-13) years, and are being treated with zoledronic acid (with a current duration of 1-5 years). In addition, 5 children had osteopenia (BMD <-2 SD); therefore, 50% of children had osteoporosis or osteopenia.

**Conclusions:**

In our cohort of children 5.2-13.2 years, 50% had osteoporosis or osteopenia, and the youngest child treated with zoledronic acid was 6 years old. We would therefore advocate early DEXA screening with VFA, in CCD, well before adolescence, with yearly follow up thereafter.

### P54

### Age and gender-specific reference percentile curves for bone parameters measured using fan-beam dual-energy X-ray absorptiometry in healthy Indian boys and girls aged 1 to 20 years.

Chirantap Oza[1], Shruti Mondkar[1], Neha Sanwalka[1], Neha Kajale[1], Vivek Patwardhan[1], Anuradha Khadilkar[1], Sharvani Patil[1], Chidvilas More[1], Raja Padidela[2], Alex Ireland[3], Vaman Khadilkar[1]

[1]Hirabai Cowasji Jehangir Medical Research Institute, Pune, India. [2]Royal Manchester Children's Hospital, Manchester, UK [3]Manchester Metropolitan University, Manchester, UK

### Abstract

### Objectives:

To develop age and gender-specific reference percentile curves for assessment of bone density measured by fan-bean dual energy X-ray absorptiometry (DXA) for boys and girls from the Indian Subcontinent.

### Methods:

A cross-sectional study was conducted on 1247 (640 boys) healthy participants from Pune, India. The GE-Lunar iDXA (encore software – version 16) was used to measure bone mineral content [BMC], bone area [BA] and bone mineral density [BMD] at total body [TB] [in participants aged 1-20 years] and measured at L1-L4 lumbar spine [LS] and left femur [in participants aged 5-20 years]. Bone mineral apparent density [BMAD] was calculated for LS and femoral neck [FN]. Gender-specific reference percentile curves for age were plotted for BMC, BA and BMD at TB, FN, and LS; Femoral Neck BMAD (FNBMAD) and Lumbar Spine BMAD (LSBMAD) using the GAMLSS model. Total Body BA for height, and lean body mass (LBM) for height and Total Body BMC (TBBMC) for Total Body BA(TBBA) and Total Body BMC (TBBMC) for LBM curves were also generated.

### Results:

The average increase with age in TBBMC was 16%, TBBA was 9% and TBBMD was 6%, LSBMD was 7.2% and FNBMD was 4.5%. TBBMC and TBBA were significantly higher in boys than girls from 14 years of age ($p$<0.05). FNBMAD and LSBMAD were significantly higher in girls than boys ($p$<0.05). An increase in TBBMC, TBBA and TBBMD was observed from 1 to 20 years in boys, whereas, flattening was observed in girls around 14-15 years of age. At 20 years, the median TBBA for age was 20.8% higher in boys than in girls (1941 $cm^2$ *vs* 1537 $cm^2$); the median TBBMC for age was 32.9% higher in boys than in girls (2047g *vs* 1374 g). A strong correlation of TBBA with height, TBBMC with TBBA, height with LBM and TBBMC with LBM was observed in both genders (I<0.001).

### Conclusion:

Reference percentile curves generated in the current study will be a helpful tool for clinical assessment of bone status as measured using a Fan-Beam DXA scanner in Indian children and youth.

### P55

### Bone age audit: Automated software (BoneXpert) vs manual estimation, in a tertiary paediatric endocrinology unit in UK

Supriyo Basu[1], Jennifer Gilbert[1], Ella-Mae Labbett[1], Kaye Platt[1], Sijith Radhakrishnan[2], Fiona Ryan[1]

[1]Oxford Children's Hospital, Oxford, UK [2]Stoke Mandeville Hospital, Aylesbury, UK

### Abstract

OBJECTIVES: Evaluation of skeletal maturity, bone age (BA) estimation, by x-ray (left wrist and hand) in a young person is commonly used in clinical practice. It allows professionals to correlate chronological age with biological maturation and thereby guides diagnoses in a range of clinical scenarios. Our aim was to compare and assess any possible significant differences in Tanner & Whitehouse (TW3) and Grulich Pyle (GP) results between manual assessment by a single expert and automated BoneXpert (BX) software scoring in a tertiary paediatric endocrinology clinic in UK

METHODS: BA was assessed manually by a single independent expert and BX software. We retrospectively audited 330 episodes of BA measurements between June 2016 and March 2021 from electronic patient records and paper notes (age range 2.22 – 20.115 years). After exclusions, 308 TW3 and 315 GP records were included in the study. Paired t-test was used for statistical analysis.

RESULTS: A statistically significant difference was found between manual and BoneXpert TW3. The mean was 0.2331 with standard deviation of 0.4722 (p value 0.00). Between BoneXpert GP and manual TW3 assessments, there was no statistically significant difference (Mean –0.0788 with SD 0.5401, p value 0.11).

CONCLUSIONS: TW3 results had statistically significant differences between manual and BoneXpert methods. This posed a valid question of reliability in real life clinical settings. However, in our clinical practice we determined a that difference of 0.2 years to be clinically insignificant. It showed the correct trend (i.e. advanced or delayed BA) and contributed to making the correct diagnosis. Weaknesses of the software were use at a very young age (< 4 years), those with skeletal dysplasia, image processing artefact and wrist/hand positioning.

Paired Samples test

| | | | | 95% CI of the difference | | | | |
|---|---|---|---|---|---|---|---|---|
| | Mean | SD | SEM | Lower | Upper | T | Df | p-value |
| Manual TW3 vs BX TW3 | 0.2331 | 0.4722 | 0.0266 | 0.1808 | 0.2855 | 8.762 | 314 | 0.00 |
| Manual TW3 vs BX GP | -0.0788 | 0.5401 | 0.0308 | - 0.1394 | -0.0183 | -2.562 | 307 | 0.11 |

### P56

### The impact of sport participation on bone mineral density in children and adolescents

Carla Caffarelli, Caterina Mondillo, Antonella Al Refaie, Alessandro Versienti, Stefano Gonnelli

Department Internal Medicine, Surgery and Neuroscience, University of Siena, Siena, Italy

### Abstract

**OBJECTIVE:**

Sport participation has been shown to have a positive impact on bone mineral density (BMD) in children and adolescents. In fact, the type, intensity, and duration of sports activities may influence the magnitude of the effect on BMD. Additionally, nutritional factors, including adequate intake of calcium, vitamin D, and other micronutrients, also play a crucial role in bone health and should be considered alongside physical activity. The aim of this study was to examine the effect of different sport on bone mineral density in children and adolescents.

**METHODS:**

We studied 78 children and adolescent (age 10.21 ± 2.96 years): 38 soccer players, 23 vocational dancers and 14 active controls. In all subjects bone mineral density at lumbar spine (BMD-LS), at femoral neck (BMD-FN) and at total femur (BMD-T) was measured by using a DXA machine (Hologic Discovery W). Moreover, the daily dietary calcium intake was assessed by a validated Food-Frequency and the presence of prior fractures was reported.

**RESULTS:**

The values of BMD at lumbar spine, expressed as Z-scores, did not differed between the three groups controls (BMD-LS Z-score values, were 0.191±1.014; 0.090±1.022 and 0.118±1.245 for soccer players, for vocational dancers and for active controls, respectively). On the contrary BMD at femoral neck and total femur were significantly higher in the soccer players groups respect with vocational dancers and active controls (BMD-FN Z-score values, were 0.265 ±1.266; -0.265±0.797 and -0.592 ±1.187 for soccer players, vocational dancers and for active controls, respectively) [Figure 1]. Soccer players with history of fractures showed no reduced BMD values compared to non-fractured, whereas vocational dancers and active controls with fracture history had reduced BMD values.

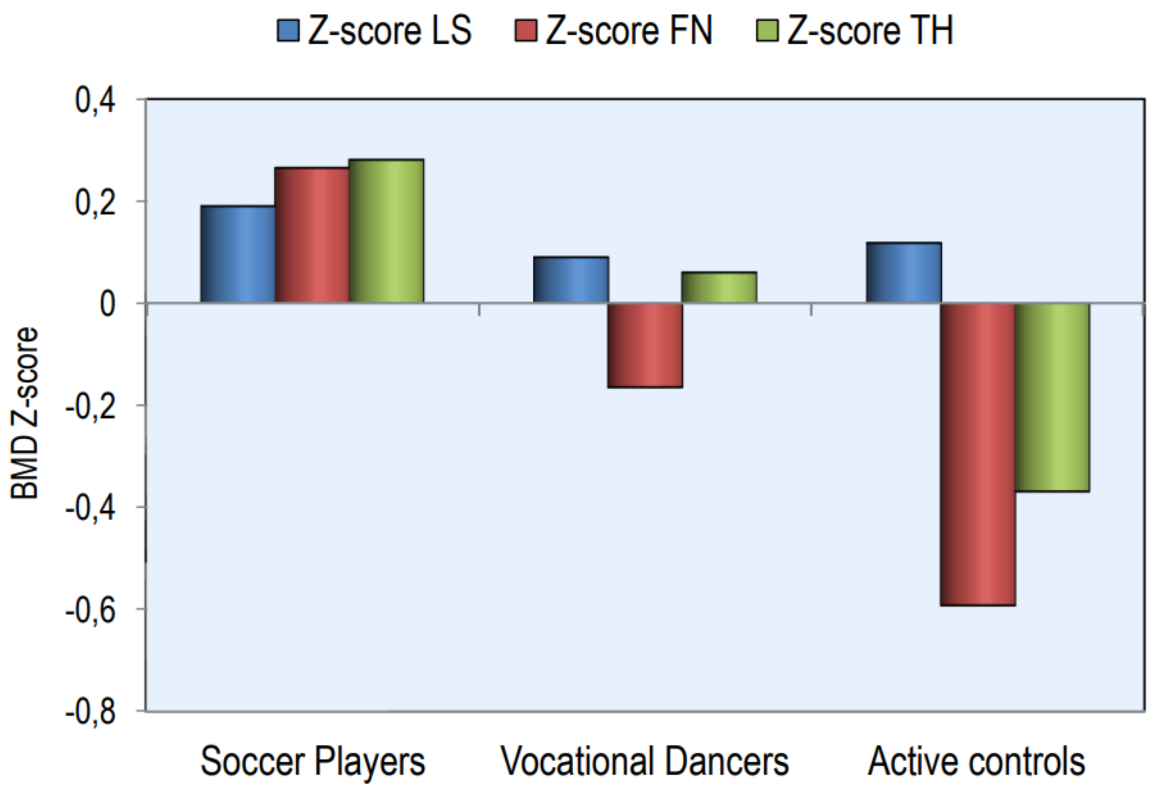

Figure 1: Values of BMD-Z-score at lumbar spine (LS), at femoral neck (FN) and at total hip (TH) in the soccer players groups respect with vocational dancers and active controls

**CONCLUSIONS:**

Sport participation during childhood and adolescence can have significant benefits for bone mineral density, contributing to overall skeletal health and reducing the risk of osteoporosis and fractures later in life. Encouraging children and adolescents to engage in regular physical activity, including sports, is therefore important for promoting optimal bone development and lifelong well-being.

### P57

### Bone densitometry findings and prevalence of osteoporosis at initial scanning in children and young people with anorexia nervosa

Nicola Crabtree[1,2], Bradley King[1], Mekhala Ayya[1], Nick Shaw[1,2], Suma Uday[1,2]

[1]Birmingham Women's and Children's Hospital, Birmingham, UK [2]University of Birmingham, Birmingham, UK

### Abstract

**Objectives:** Optimal timing and indications for dual energy X-ray absorptiometry (DXA) scans in adolescents with Anorexia Nervosa (AN) remains unknown. The UK National Institute for Health and Care Excellence recommends a scan after one year of underweight.

In girls with AN at referral:1) Report the DXA and peripheral quantitative computed tomography (pQCT) findings and stratify risk based on menstrual status. 2) Determine prevalence of osteoporosis [defined as the presence of vertebral fractures (VF) or a clinically significant fracture history (long bone fractures: ≥2 by 10 years or ≥3 at any age up to age 19 years) and low bone mineral density (BMD) Z-score ≤-2.0].

**Methods:** Retrospective review of DXA and pQCT data on girls referred for their first scan between March 2013 and October 2022. A questionnaire was used to gather self-reported fracture history and pubertal status. Pubertal status was classified as (detailed in table) normal, delayed, irregular menstruation, secondary amenorrhoea or unknown (insufficient data). Data on anthropometry, bone density parameters from DXA and pQCT and VF from DXA were gathered. One-way ANOVA was used to ascertain the difference in densitometric parameters between the groups.

**Results:** 264 girls with a median age of 15.6 years were included. The majority (54.5%, n=144) reported secondary amenorrhoea. The mean (SD) height, weight and BMI of the whole cohort were 161.7(7.5) cm, 48.1(8.1) Kg and 18.3(2.3) Kg/m$^2$ respectively. Mean weight SDS [-1.1(1.3)] and BMI SDS [-1.2(1.2)] were significantly lower than zero (p<0.001). All densitometric parameters in the cohort were significantly lower than zero and significantly lower in girls with delayed puberty when compared to other pubertal groups (**table)**. Long bone fractures were reported by 16.7% (n=44/264) of whom 3% (n=8, all >10 years) reported two fractures. Mild (<25%) single VFs were noted in 1% (n=3), who also met the criteria for osteoporosis.

**Conclusions:** Low BMD is frequent but osteoporosis is rare at initial referral in girls with AN. Girls with delayed puberty have the highest skeletal deficits. Developing a risk score may help determine the appropriate timing of first scan and the individuals who would most benefit from it to consider therapeutic interventions.

| Mean Z-scores (SE) - Adjusted for Age (15.5 years) and BMI (18.3 kg/m$^2$)<br>1.Significant difference between pubertal groups ($p<0.05$)<br>2.Significantly different from zero ($p<0.05$) | **All**<br><br>**(n=256)** | **Normal puberty**<br><br>**(n = 39)** | **Delayed puberty** (no breast development by 13 or no menarche by 15 years)<br><br>**(n = 37)** | **Irregular menstruation** (amenorrhoea < 3 months or a missed period)<br><br>**(n = 36)** | **Secondary amenorrhoea** (no menstruation >3 months with previously regular menses)<br><br>**(n = 144)** | Significant covariates in the model |
|---|---|---|---|---|---|---|
| **Lumbar Spine Bone Mineral Apparent Density (LS BMAD)** [1,2] | -0.9 (1.2) | -0.8 (0.2) | -1.3 (0.2) | -1.1 (0.2) | -0.8 (0.1) | Age |
| **Total Body Less Head Bone Mineral Density (TBLH BMD)** [1,2] | -0.8 (0.9) | -1.0 (0.1) | -1.1 (0.1) | -0.8 (0.1) | -0.7 (0.1) | Age, Height, BMI |
| | **All (n=232)** | **Normal (n = 38)** | **Delayed (n = 33)** | **Irregular (n = 35)** | **Secondary (n = 127)** | |
| **Trabecular BMD** [2] **(4% Radius)** | -0.4 (1.2) | -0.8 (0.2) | -0.4 (0.2) | -0.5 (0.2) | -0.3 (0.1) | Age |
| **Total BMD** [1,2] **(4% Radius)** | -0.6 (0.9) | -0.9 (0.1) | -0.9 (0.2) | -0.6 (0.2) | -0.5 (0.1) | Age |
| **Cortical BMD** [1,2] **(66% Radius)** | 0.5 (1.4) | 0.6 (0.2) | -0.7 (0.2) | 0.7 (0.2) | 0.7 (0.1) | Age |
| **Muscle Area for height** [2] **(66% Radius)** | -2.4 (0.9) | -2.4 (0.1) | -2.2 (0.1) | -2.4 (0.1) | -2.4 (0.1) | |
| **Fat Area for height** [2] **(66% Radius)** | -0.9 (1.0) | -0.6 (0.1) | -1.0 (0.2) | -0.6 (0.2) | -1.0 (0.1) | |
| **Bone Area for height** [1,2] **(66% Radius)** | 2.5 (1.4) | 2.1 (0.2) | 1.7 (0.3) | 2.8 (0.2) | 2.8 (0.1) | |
| **Bone Area to Muscle Area** [1,2] **(66% Radius)** | 1.9 (1.2) | 1.7 (0.2) | 1.2 (0.2) | 1.9 (0.2) | 2.2 (0.1) | |

### P58

### A paediatric case of idiopathic tumoral calcinosis in the presence of normal bone biochemistry

Louise Apperley, Charlie Jarvis, Suzanne Armitage, Ghadir Kassab, Renuka Ramakrishnan

Alder Hey Children's Hospital, Liverpool, UK

### Abstract

**Background:**

We report an 18-year-old female with known Jacobsen Syndrome (*11q deletion*) with unusual bilaterally symmetrical hip calcifications. She is a significantly disabled and wheelchair dependent child.

**Presenting Problem:**

This patient had regular spine and hip x-rays for scoliosis surveillance. At 14 years of age, calcification was first noted over the great trochanters. Images showed bilateral symmetrical cotton-like calcification in the periarticular soft tissues. A computerised tomography scan was performed, which suggested idiopathic tumoral calcinosis in the presence of normal phosphate levels and confirmed that this was confined to the gluteus maximus muscle.

**Clinical Management:**

The patient had large visible, palpable and painless lumps to the lateral and posterior areas of the hip joints bilaterally, with full range of movement reserved. She had normal parathyroid hormone, calcium and phosphate levels and renal profile with no evidence of metabolic or collagen disorders. A gene panel was performed which showed no pathogenic variant of *FGF23* and *GALNT3* genes, excluding familial tumoral calcinosis. She had no history of hip surgery or trauma. Serial x-rays have shown a steady increase in the size of these lesions (Figure 1).

**Discussion:**

Guidance from national and international experts was sought to decide on the best management, including surgery or local treatment with sodium thiosulphate. The calcifications are not causing impairment of movement or pain at present. They are currently being monitored to look for extension into the extra-articular surfaces of the hip joint inwardly or into the subcutaneous tissue outwardly. Her overlying skin is regularly assessed for any compromise in viability. Due to her complex background, she is a high-risk patient for surgery, and it is thought that calcified lesions often return after removal.

Idiopathic tumoral calcinosis is a benign condition and occurs due to the presence of calcium phosphate crystals within soft tissue areas. It is often associated with hyperphosphataemia, but this was not seen in our patient. Normophosphataemic familial tumoral calcinosis has been described secondary to a mutation in *SAMD9* in Jewish-Yemenite population. This gene has not been tested in our patient as she does not fit within this ethnic group.

*Figure 1 shows the presence and change of idiopathic tumoral calcinosis in the gluteus maximus muscles bilaterally over the last four years*

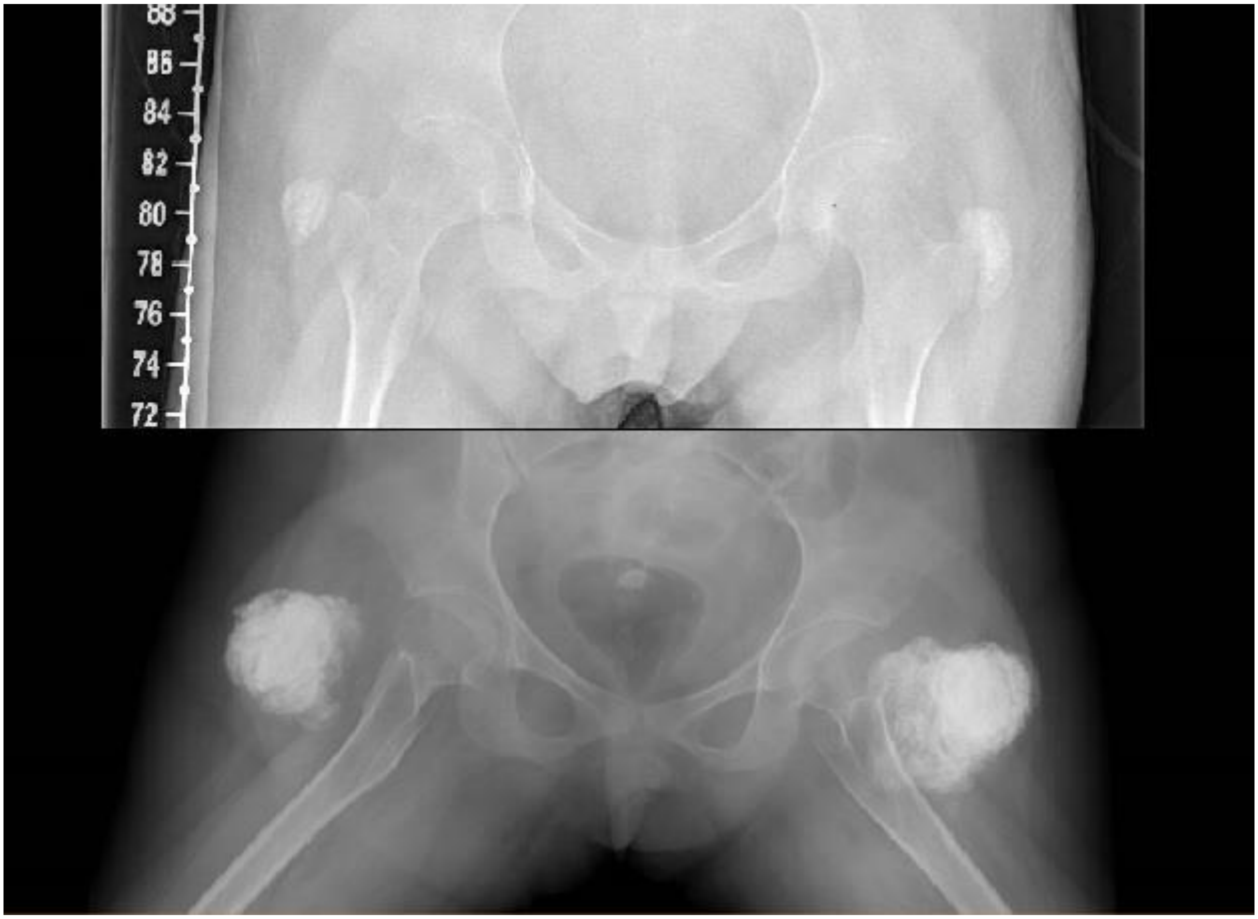

### P59

### Exploring BHI as an alternative to DXA for assessing bone health in children

Huda Elsharkasi, Rui Santos, Alessandra Cocca

Evelina, London children Hospital, London, UK

### Abstract

**Objective**:

This study explores an alternative method for assessing bone health by evaluating the agreement between dual-energy X-ray absorptiometry (DXA)-based areal bone mineral density (BMD) and the Bone Health Index (BHI) derived from digital X-ray radiogrammetry (DXR) in paediatric patients with different medical backgrounds.

**Methods:**

A retrospective analysis was conducted on DXA scans and left-hand radiographs from 24 patients under 18 years old, receiving care at the bone clinic at Evelina Hospital, London, due to a risk for impaired bone health between 2018-2023. Inclusion criteria were patients aged <18 years with a maximum interval period between two scans ≤6 months. Anthropometric data, medication details, and oral contraceptive usage were collected. Areal BMD measurements (Total body less head (TBLH) and lumbar spine (LS)) from DXA and BHI from DXR were obtained. Statistical analyses, including Wilcoxon one-sample tests, sensitivity, specificity and Bland-Altman plots, were performed.

**Results**:

Median Z scores for BHI, BMD TBLH, and BMD LS were significantly lower than the reference population (Z score of 0) ($p < 0.0002$). The Bland-Altman plot indicated a mean difference of 0.03 ± 1.7 between BHI and BMD TBLH Z scores, with limits of agreement from -3.7 to 3.4. For BMD LS, the mean difference was 0.58 ± 2, demonstrating reasonable agreement. Using DXA as the reference method, sensitivity of BHI for abnormal BMD LS was 55%, and for BMD TBLH, it was 42%. For specificity values were 70% and 60% for BMD LS and BMD TBLH, respectively.

**Conclusion:**

Despite variations in sensitivity and specificity, the study suggests acceptable agreement between DXA-based BMD and DXR-derived BHI in paediatric bone health assessment. As DXR holds promise, particularly in resource-limited settings, further research with larger cohorts is imperative to validate these findings and establish the clinical utility of DXR in paediatric bone health assessments.

### P60

### Interpretation of DXA in transgender and gender-diverse youth

Heidi Kecskemethy, Sooji Kim, Evan Graber, H Theodore Harcke

Nemours Children's Health, Wilmington, USA

### Abstract

### Objectives

Evaluate DXA in transgender and gender diverse (TGD) youth and examine interpretation of results using male and female reference values.

### Methods

Retrospective review of patients ≤ 18 years receiving gender affirming medical care at our children's hospital and who had DXA scans. Clinical variables (pharmacologic treatment, fracture history, serum 25(OD)D level) were evaluated. All had at least one DXA scan; all body sites measured were assessed. As available, bone mineral density (BMD) and z-score results for total body less head (TBLH), lumbar spine (LS), and lateral distal femurs (LDF) regions 1 – 3 were examined for both sexes. Height-adjusted z-score (HAZ) was calculated for TBLH and LS. Serial DXA was assessed when available. Z-score patterns comparing birth sex norms to opposite sex norms were assessed.

### Results

Thirty TGD children (16 assigned female at birth (AFAB)) with a mean age at first DXA of 13.3 years (9.9 to 17) were identified. Twenty-seven received pubertal suppression (PS) and 15 received gender-affirming hormone (GAH). Forty percent had insufficient/deficient 25(OH)D status; 30% had fracture history. Eleven patients had serial scans (5 AFAB) with a maximum of 3 scans and 6.1 years of observation.

Mean age of PS initiation differed by sex:  AFAB started at 11.5 years and AMAB started at 13.1 years. Half of the children received GAH; all had received PS.

BMD Z-scores for LS were more influenced by reference value selection than TBLH or LDF. Consistently, LS z-scores were lower in AMAB when using female norms, and higher for AFAB when using male norms. HAZ reduced variation for TBLH. Serially, BMD trend provided clarification of Z-score changes.

### Conclusions

Application of sex-specific normative reference values for assessing BMD by DXA in TGD children yields varying results by body site measured. Treatments including PS and GAH affect expected BMD accretion patterns during childhood, particularly during pubescent years, complicating interpretation of BMD z-scores. Using gender-identity norms, as recommended for transgender adults, is not adequate for TGD children. Ensuring a quality examination, accurately analyzed for BMD and reported with HAZ scores, referenced to both male and female normative values, best serve TGD children.

Table 1. Site BMD Z-score, with and without height adjustment, is affected by application of opposite sex normative reference values in youth naïve to and receiving pubertal suppression. Note, lateral distal femur DXA analysis technique adjusts for bone size, therefore height adjustment is not applicable. (a) Total body less head (b) Lumbar spine (c) Lateral distal femur.

| 2 (a) Total body less head | | | | Effect on z-score by using sex NAAB norms | | | | | |
|---|---|---|---|---|---|---|---|---|---|
| Group<br>(sex AAB) | Reference used | Mean age at DXA (years) | n | Increased | | Decreased | | No change | |
| | | | | z-score | HAZ | z-score | HAZ | z-score | HAZ |
| Male no PS | Female | 13.5 | 6 | 4 | 2 | 2 | 4 | 0 | 0 |
| Male PS | Female | 14.8 | 8 | 5 | 3 | 2 | 2 | 1 | 3 |
| Female no PS | Male | 11.9 | 7 | 7 | 5 | 0 | 0 | 0 | 2 |
| Female PS | Male | 13.0 | 9 | 4 | 7 | 4 | 2 | 1 | 0 |

| 2 (b) Lumbar spine | | | | Effect on z-score by using sex NAAB norms | | | | | |
|---|---|---|---|---|---|---|---|---|---|
| Group<br>(sex AAB) | Reference used | Mean age at DXA (years) | n | Increased | | Decreased | | No change | |
| | | | | z-score | HAZ | z-score | HAZ | z-score | HAZ |
| Male no PS | Female | 13.5 | 6 | 1 | 1 | 5 | 5 | 0 | 0 |
| Male PS | Female | 14.8 | 8 | 0 | 0 | 8 | 8 | 0 | 0 |
| Female no PS | Male | 11.9 | 7 | 7 | 7 | 0 | 0 | 0 | 0 |
| Female PS | Male | 13.0 | 9 | 8 | 9 | 0 | 0 | 1 | 0 |

| 2 (c) Lateral distal femur | | | | Effect on z-score by using sex NAAB norms | | | | | | | | |
|---|---|---|---|---|---|---|---|---|---|---|---|---|
| Group<br>(sex AAB) | Reference used | Mean age at DXA (years) | n | Increased | | | Decreased | | | No change | | |
| | | | | R1 | R2 | R3 | R1 | R2 | R3 | R1 | R2 | R3 |
| Male no PS | Female | 13.5 | 6 | 3 | 2 | 2 | 2 | 3 | 4 | 1 | 1 | 0 |
| Male PS | Female | 14.8 | 8 | 3 | 4 | 3 | 5 | 3 | 5 | 0 | 1 | 0 |
| Female no PS | Male | 11.9 | 7 | 6 | 5 | 3 | 1 | 2 | 3 | 0 | 0 | 1 |
| Female PS | Male | 13.4 | 8 | 4 | 6 | 6 | 1 | 2 | 2 | 3 | 0 | 0 |

Abbreviations: sex NAAB, sex not assigned at birth; sex AAB, sex assigned at birth; DXA, dual-energy x-ray absorptiometry; HAZ, height-for-age z-score; PS, pubertal suppressants.

### P61

### Comparative analysis of bone mineral density in cerebral palsy and spinal muscular atrophy

Brianna Bowden[1], Brett Klamer[2], Garey Noritz[1]

[1]Nationwide Children's Hospital, Columbus, USA [2]The Ohio State University, Columbus, USA

### Abstract

Objectives:

Cerebral palsy (CP) and spinal muscular atrophy (SMA) are conditions characterized by motor disabilities, leading to non-ambulation in severe cases, associated with increased risks of low bone mineral density (BMD) and fragility fractures. Given the distinct pathological mechanisms underlying CP and SMA, this study aims to investigate whether differences exist in BMD between non-ambulatory children with CP (hypertonia) and those with SMA (hypotonia).

Methods:

Utilizing electronic medical records, data were collected from patients diagnosed with CP and SMA, aged five years and older, who were under the care of the metabolic bone clinic at a large children's hospital between January 1, 2012, and July 1, 2023. Dual-energy X-ray absorptiometry (DXA) was used for the assessment of bone mineral density (BMD) at the lumbar spine (LS) and the lateral distal femur, specifically region 3 (R3). Z scores at these sites were calculated using the manufacturer's normative database. The most recent DXA results prior to bisphosphonate treatment were collected. The two groups were compared using the Wilcoxon rank sum test.

Results:

Among the 400 charts reviewed, 274 non-ambulatory children with CP and 16 non-ambulatory children with SMA meeting the criteria were included in the analysis. LS Z scores were low and comparable in both CP and SMA groups (-2.3 vs. -2.6, respectively; $p = 0.7$), but when adjusted for height, the median LS Z scores for both groups were in the normal range (-0.9 vs. -1.6; $p = 0.3$). However, the SMA group had significantly lower BMD Z scores in region 3 of the distal femur compared to the CP group (-3.9 in SMA vs. -2.9 in CP; $p = 0.047$).

Conclusions:

Children with SMA had a more pronounced reduction in BMD, particularly in region 3 of the distal femur, compared to CP patients. This may reflect the differences in the impact of muscle tonicity on bone. This underscores the importance of incorporating distal femur bone density assessment into routine bone health evaluations to identify the increased severity of skeletal status in these patient populations.

### P62

### Vertebral fractures in underprivileged Indian children and youth with poor glycaemic control of type-1 diabetes

Chirantap Oza, Anuradha Khadilkar, Gauri Khot, Shruti Mondkar, Vivek Patwardhan, Sharvani Patil, Chidvilas More, Vaman Khadilkar

Hirabai Cowasji Jehangir Medical Research Institute, Pune, India

### Abstract

**Objective:**

Individuals with type 1 diabetes (T1D) have a 1.2- to 3-fold increase in fracture risk at any site relative to those without diabetes. This higher fracture risk begins in childhood and extends across the lifespan. There is a limited number of studies on the prevalence of vertebral fractures (VF) in T1D, mostly from adult datasets of developed countries. The high prevalence of VFs reported among patients with T1D, raises the question as to whether this population would benefit from earlier screening for VF. We thus conducted this pilot study to estimate prevalence of VF in underprivileged Indian children and youth with poor glycaemic control of T1D, to compare the characteristics between participants with T1D and a VF vs participant with T1D without and to identify factors associated with a VF in participants with T1D.

**Methods:**

This cross-sectional pilot study included 30 (20 girls) Indian children and youth (mean age 13.3 years) with poor glycaemic control (HbA1c >10%) of T1D from a tertiary care centre in Pune, western India. VF assessment by dual-energy x-ray absorptiometry (DXA) was used for prevalent VFs using Genant classification. Demographic data were obtained from patients using questionnaires. Anthropometry, blood pressure, sexual maturity rating and biochemical measurements were performed using standard protocols.

**Results:**

We report 6.7% subjects (n=2) with VF in our study group. None of these patients has history of fracture. Of the subjects with VF, one had compression of L4 and other had severe wedge fracture at L1 (A/P ratio of 0.57) along with moderate concavity at L2 level. The patients with VF were vitamin D insufficient, had high triglyceride level and low insulin-like growth factor-1 (IGF-1) levels as compared to the mean levels of rest of the subjects in study group. Figure 1 shows VF scan of an affected subject.

**Conclusion:**

VF are present in young age in subjects with T1D with poor glycaemic control. Studies with larger sample size are required to identify the predictors, however, vitamin D levels, lipid profile and IGF-1 seem to have a role in the same.

T12
L1 Severe Wedg
L2 Moderate Cor
L3
L4
Image for spine morphometry assessment only

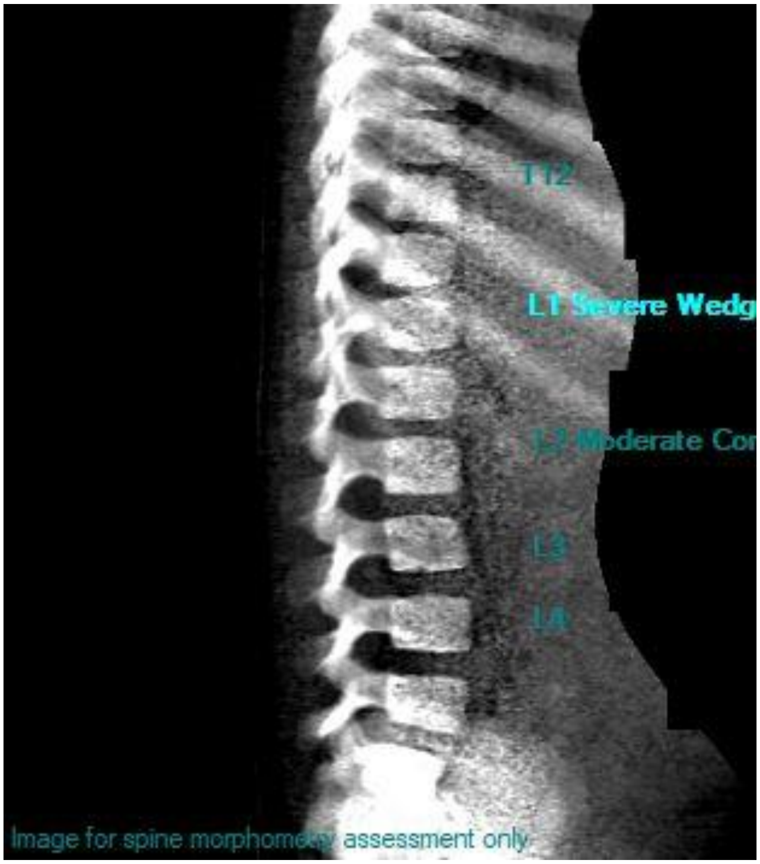

### P63

### Does the bone health index value differ between infants and children under two years of age who do or do not have osteogenesis imperfecta?

Heba Shalof[1], Rachel Shuyi Chong[1], Alan Rigby[2], Amaka Offiah[1]

[1]University of Sheffield, Sheffield, UK [2]University of Hull, Hull, UK

### Abstract

In children with unexplained fractures who are below the age of two years, it may be difficult to distinguish those with low bone mineral density due to conditions such as osteogenesis imperfecta (OI) from those who have been abused, because both groups suffer from multiple fractures and may have otherwise normal-appearing skeletal imaging. Currently, no imaging modality can readily or reliably assess bone mineral density (BMD) or evaluate bone strength in infancy.

#### Aim

The aim of this study is to investigate if infants and children under two years of age with a diagnosis of OI have a significantly lower BHI than those without OI or other conditions associated with low BMD. The correlation between BHI and serum (25-hydroxy) vitamin D levels was assessed in those without OI.

#### Material and methods

A total of 122 radiographs were analysed, of which 33 were of infants diagnosed with OI and 89 radiographs were of healthy infants with known vitamin D levels. BHI values were retrospectively measured from and compared between both groups of radiographs using BoneXpert software and the correlation between BHI and serum vitamin D was assessed.

#### Results

BHI was significantly greater in the group without OI compared to the group with OI, 3.75 and 3.41, respectively (P=0.003). The percentage of children in the OI/non-OI groups with BHI ≤2.49, 2.5–2.99, 3– 3.49 and ≥4 was 0%/0%, 27%/7%, 58%/28%, 18% /29%, and 12%/36%, respectively. While BHI standard deviation scores (SDS) were significantly greater in the group without OI compared to the group with OI, -0.039 and -0.451, respectively (P=0.01), BHI SDS was within the normal range for both groups. There was no correlation (r=-0.09) between BHI and vitamin D in the healthy group. The area under the curve was 0.67 (95% CI=0.56-0.79, p=0.005). For BHI >=3.23 units sensitivity was 0.80, and BHI>=3.99 units specificity was 0.80.

#### Conclusion

We showed a statistically significant difference in BHI and BHI SDS between infants and young children with and without OI. Although our sample is small, our results indicate that in a child <2 years old presenting with unexplained fractures, a BHI value of 4.72 indicates normal bones with a sensitivity of 3% and a specificity of 96%.While BHI may potentially be used to assess predisposition to fracture in an infant or young child with unexplained fractures, further research is required.

### P64

### Does the bone health index correlate with high-resolution peripheral quantitative computed tomography parameters including finite element analysis in children and young adults?

Heba Shalof[1], Alan Rigby[2], Paul Dimitri[1], Amaka Offiah[1]

[1]University of Sheffield, Sheffield, UK [2]University of Hull, Hull, UK

### Abstract

### Aim

The main aim of this study was to assess the degree of correlation between BHI values (measured from hand and wrist radiographs using BoneXpert software) and HR-pQCT parameters [including finite element analysis (FEA) of the distal radius] in healthy children and bisphosphonate naïve paediatric patients with low bone mineral density as measured by DXA.

### Methods

A prospective observational cohort study was carried out over a 2-year period. We recruited 36 participants aged between 8 and 16 years; 20 healthy children and 16 (6 males) bisphosphonate naïve patients with reduced bone mineral density, as confirmed by DXA. Each child underwent a non-dominant hand and wrist radiograph, along with an ipsilateral distal radial HR-pQCT scan on the same day. Participants completed a questionnaire about their experiences of these scans. Pearson's correlation coefficient to assess and calculate the degree of linear association between BHI values and HR-pQCT parameters (including FEA) was performed. A Bland Altman plot was created to graphically demonstrate the degree of correlation between the two methods. The effect of sex and age on the correlation between BHI and HRpQCT parameters was assessed.

### Results

BHI and BHI SDS values were significantly higher in healthy group than patients' group ($p$<0.0001 and $p$<0.0001, respectively). There was a strong correlation between BHI SDS and most of HR-pQCT parameters (including FEA). BHI SDS correlates strongly with total density and trabecular density (r=0.69 and 0.68, respectively), while correlates moderately with cortical density (r=0.50). BHI SDS correlates strongly with most FEA (stiffness, estimated failure load, and Trab average von Mises stress) (r= 0.60, 0.61, and 0.60, respectively). Bland-Altman (BA) plot showed a good agreement between standard deviation of BHI and HR-pQCT parameters.

**To sum up**, a significant correlation between BHI and HR-pQCT parameters included FEA was found. Although the BHI SDS is dependent mainly on cortical structure, a strong correlation was found between the BHI SDS and trabecular parameters. So, BHI SDS may reflect the cortical and trabecular bone density in healthy children. BHI may be used to predict fracture risk in children. A large definitive study will be designed to validate the use of BHI to identify children with low bone mineral density who have a high risk of low trauma fractures.

**P65**

### Coat-hanger ribs: Diagnosis at a glance

Giulia Rosti[1,2], Maria Francesca Di Feo[2], Silvia Buratti[3], Francesca Faravelli[1]

[1]Genomics and Clinical Genetics Unit, IRCCS Giannina Gaslini, Genova, Italy. [2]Department of Neurosciences, Rehabilitation, Ophthalmology, Genetics, Maternal and Child Health (DiNOGMI), Genova, Italy. [3]ICU, IRCCS Giannina Gaslini, Genova, Italy

### Abstract

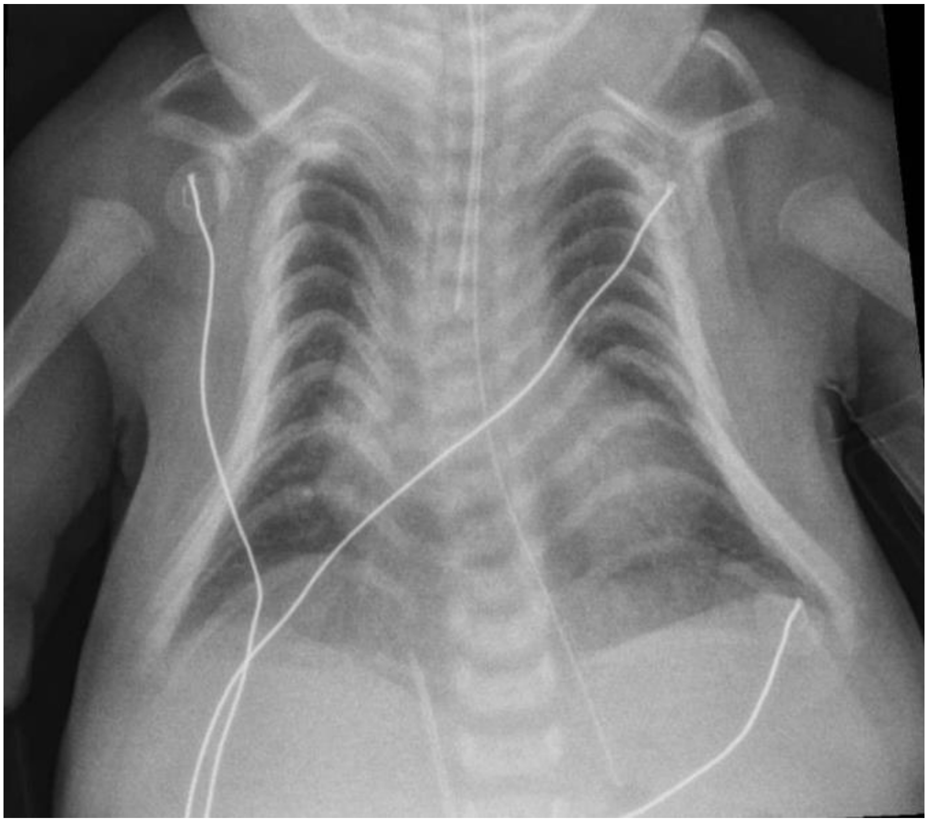

**BACKGROUND:**

A newborn female was admitted to the Neonatal ICU of IRCCS Giannina Gaslini Institute because of respiratory insufficiency. She was born at 35 weeks of gestational age by C-section due to altered CTG. Birth weight was 2.881 kg (94th centile). Apgar score was 3/5/6. Endotracheal intubation was required.

**PRESENTING PROBLEM:**

On examination, macrosomy, short neck, hand contractures and small thorax were observed. Chest X-Ray showed a small, bell-shaped thorax with coat-hanger ribs (Fig.1). Based on this association of findings, she was referred to clinical geneticists with a suspicion of Jeune syndrome.

**CLINICAL MANAGEMENT:**

A review of the X-Rays by the clinical genetics team prompted a suspicion of Kagami-Ogata Syndrome. MS-MLPA analysis confirmed the presence of maternal uniparental disomy (UPD) of chromosome 14.

The baby is now 5-month-old. She was recently discharged but still requires ventilation.

**DISCUSSION:**

This case highlights the value of specialised clinical expertise and X-Ray imaging in the identifications of rare genetic conditions.

The clinical presentation of this patient was consistent with a form of skeletal dysplasia. An initial suspicion of Jeune Syndrome had been considered in view of the significant narrowing of the chest. However, the shape and orientation of the ribs did not resemble the short and horizontal ribs seen in patients with Jeune syndrome. By contrast, the X-Ray image was very peculiar and evocative. A quick review of the literature confirmed that coat-hanger ribs are pathognomonic of Kagami-Ogata Syndrome. This condition is caused by maternal UPD of chromosome 14, a pathogenic mechanism that can only be detected through specific tests. It is important to underline that many of the NGS based diagnostic strategies currently available would have not detected the genetic abnormality in this patient, extending the diagnostic odyssey for this family.

### P66

### Relationship of sunlight exposure, Erythemal dose and Vitamin D concentrations in adolescents from India

Chidvilas More[1], Neha Kajale[1,2], Vivek Patwardhan[1], Chirantap Oza[1], Sharvani Patil[1], Shruti Mondkar[1], Anuradha Khadilkar[1,2]

[1]Hirabai Cowasji Jehangir Medical Research Institute, Jehangir Hospital, Pune, India. [2]School of Health Sciences Savitribai Phule Pune University, Pune, India

### Abstract

**Background:** Despite abundance of sunshine in India, Vitamin D deficiency is common; the most important source of Vitamin D in India is sunlight. Previously, as a part of a multicentre study, we assessed 25(OH)$D_3$ concentrations from dried blood spots in 5–18-year-old Indian children and adolescents using a standardized protocol and identified factors contributing towards Vitamin D deficiency. In the current study, our aim was to assess relationship between Vitamin D concentrations, sunlight exposure and standard erythemal dose(SED) (as assessed by PSU badges) in urban and rural adolescents from various regions of India.

**Objectives:**

1. To assess the relationship of Vitamin D concentrations with sunlight exposure (as erythemal dose assessed by PSU badges),
2. To assess the erythemal dose received as per place of residence, sex and season.

**Methods:** Cross-sectional, observational school-based study was conducted (July 2016-October 2017); multi-stage stratified random sampling was performed. A city and nearby village were selected from 6 Indian states covering wide geographical areas. 2500 children were assessed for vitamin D3 (SR, 2020) out of which, for the current study, 545 children whose polysulfone dosimeter badges (PSU) data was retrieved were analysed. Pearson Chi-Square test, Phi, 2-tailed significance and Cramer's V test were performed for statistical analysis.

**Results:** Vitamin D deficiency was significantly higher in urban girls and boys as compared to their rural counterparts ($p$<0.05). Overall, sunlight exposure (as judged by higher SED values) was more for rural boys (1.07±0.9SED) and girls (0.85±0.5SED) as compared to that of urban boys (0.88±0.6SED) and urban girls (0.63±0.4SED) respectively. Sunlight exposure was higher in winter season (78.3%) whereas it was lower in monsoon season (21.7%). Significant correlation was observed between sunlight exposure and erythemal dose as well as between erythemal dose and vitamin D3 concentrations. Sunlight exposure in rural boys and erythemal dose showed significant correlation.

**Conclusion:** Erythemal dose differed with sunlight exposure, sex, place of stay and season. Higher erythemal dose was found with higher sunlight exposure, in winter season and in rural participants. Increasing sunlight exposure may be an effective strategy to improve vitamin D concentrations especially in monsoon and in urban areas.

Erythemal Dose
0.76, 44%
0.95, 56%
Urban
Rural

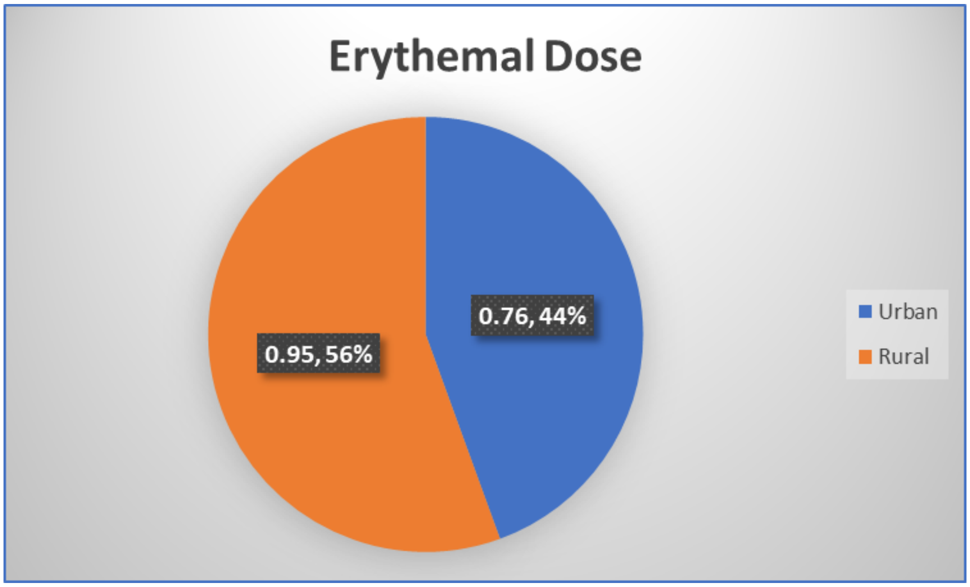

### P67

### Beyond Vitamin D: Elevated Parathyroid Hormone is associated with inferior event-free survival in childhood malignancies

Corinna Grasemann[1], Jakob Höppner[2], Wolfgang Högler[3], Stephan Tippelt[4], Desiree Grabow[5], Gunnar Cario[6], Martin Schrappe[6], Dirk Reinhardt[4], Michael Schündeln[4]

[1]Ruhr-University Bochum, Bochum, Germany. [2]Harvard University, Boston, USA [3]Kepler Universitätsklinikum, Linz, Austria. [4]Universitätsklinikum Essen, Essen, Germany. [5]hannes Gutenberg University Mainz, Mainz, Germany. [6]UKSH, Kiel, Germany

### Abstract

Objective: Vitamin D deficiency is tied to poor outcomes in cancer, but research on its commonly occurring consequence, secondary hyperparathyroidism is limited. In fact, parathyroid hormone (PTH) and its related protein (PTHrP) have been shown to modulate initiation, progression and metastatic potential in different types of cancer *in vitro*. To date, cancer survival in children has not been assessed in relation to PTH status.

Design: This cross-sectional study investigates association of PTH and 25-OH vitamin D (25OHD) in paediatric cancer patients with event free and overall survival (EFS/OS).

Methods: Real world laboratory data of 4349 cancer patients (age 0-18 years) from a tertiary paediatric cancer unit were screened for available PTH and 25OHD readings at diagnosis and the following five years. For each patient, the highest PTH and lowest 25OHD concentrations were recorded.

Data on first relapse, secondary malignancies and mortality were obtained and stratified for **PTH levels above/below the cohort median** (47 pg/ml) and by the presence/absence of **vitamin D deficiency** (25OHD ≤ 30 nmol/L). Event free survival (EFS) and overall survival (OS) was analyzed, and hazard ratios (HR) were calculated for the entire cohort and the cancer subgroups: lymphatic malignancy; malignant primary brain tumor (PBT); benign PBT; embryonal malignancy; sarcoma; myeloid malignancy and other.

Results: PTH and 25OHD values were available in 1286 cases (731 male). Higher PTH was associated with inferior EFS in patients with malignant PBT (HR 1.80 [1.19 – 2.72]), embryonal malignancies (HR 2.20 [1.1 – 4.43]) and lymphatic malignancies (HR 1.98 [1.05 – 3.72]). Vitamin D deficiency was associated with inferior EFS in embryonal malignancies only (HR 2.41 [1.24 – 4.68]). To differentiate the effects of vitamin D deficiency from higher PTH, a multivariate Cox proportional hazard model was conducted in which only higher PTH remained significant for inferior EFS.

Conclusion: In certain paediatric cancers, higher PTH may signal adverse outcomes. The results indicate that adequate vitamin D and calcium supply should be ensured to avoid secondary hyperparathyroidism. Prospective studies are needed.

### P68

### Estimation of prevalence of Limited Joint Mobility (LJM) in Indian children and adolescents with type 1 diabetes

Sharvani Patil[1], Nimisha Dange[1], Chirantap Oza[1], Neha Kajale[1,2], Shruti Mondkar[1], Chidvilas More[1], Anuradha Khadilkar[1,2]

[1]Hirabai Cowasji Jehangir Medical Research Institute, Jehangir Hospital, Pune, India. [2]School of Health Sciences Savitribai Phule Pune University, Pune, India

### Abstract

**Background:** Limited joint mobility (LJM) is common in long-standing type 1 diabetes mellitus (T1D) especially with suboptimal diabetes management. Presence of LJM is believed to be an early marker of developing complications such as albuminuria and retinopathy. Data on LJM in Indian children and adolescents with diabetes are lacking.

**Objectives:**

1. To compare joint mobility in children with T1D and healthy controls.
2. To determine the prevalence of LJM in children and adolescents with type 1 diabetes.

**Methods:** This was an observational cross-sectional study on l 132 children and adolescents from 10 to 21 years of age who presented at the pediatric diabetic clinic at a tertiary centre in Pune (Western Maharashtra), India. Sixty-six age gender matched healthy children, without any chronic illness or conditions affecting joint function were also recruited. Demographic details were noted, and anthropometry was measured (standard protocol). Range of motion on passive extension of small joints of both hands (proximal and distal interphalangeal joints, metacarpophalangeal joints) was carried out using a finger goniometer. Large joint involvement was assessed with a goniometer for wrist/elbow extension and ankle dorsiflexion. Involvement of lateral flexion of cervical/thoracolumbar spine or obvious hand deformity were considered as grade 3 (severe) limited joint mobility. Joint mobility of children with diabetes was then compared with healthy children; LJM was classified using Rosenbloom's staging.

**Results:** Mean age of children with diabetes was 15.7 ± 3.4 and for healthy children was 14.8 ± 3.3 years. Amongst the 132 children with diabetes (54 boys (40.9%)), 37 (28%), 53 (40.2%) and 32 (24.2%) were classified with mild, moderate and severe LJM, respectively. Independent T test showed significant differences in restricted passive extension of distal and proximal interphalangeal joints of hand when compared with healthy children (Table 1). Limited joint mobility was significant in children with long term diabetes (<5 years) (p-value 0.029).

**Conclusion:** Prevalence of LJM is high using goniometer assessment, contrary to the traditional 'prayer sign' or 'tabletop sign' assessment as reported in literature. Early recognition of such individuals may halt the progression towards prospective microvascular complications by appropriate timely intervention.

| Joints tested | Movement | Mean ROM for diabetic children | Mean ROM for healthy children | *p*-value | Effect size |
|---|---|---|---|---|---|
| Right IP | Extension | 38.4 | 46.5 | <0.001* | -3.478 |
| Left IP | Extension | 39.8 | 46.8 | <0.001* | -3.043 |
| Right 2nd DIP | Extension | 23.1 | 31 | <0.001* | -4.388 |
| Left 2nd DIP | Extension | 24.1 | 32.9 | <0.001* | -4.888 |
| Right 3rd DIP | Extension | 23 | 30.9 | <0.001* | -4.388 |
| Left 3rd DIP | Extension | 24.1 | 30 | <0.001* | -3.105 |
| Right 4th DIP | Extension | 23.7 | 32.0 | <0.001* | -4.666 |
| Left 4th DIP | Extension | 24.8 | 32.0 | <0.001* | -4 |
| Right 5th DIP | Extension | 23.9 | 32.7 | <0.001* | -4.579 |
| Left 5th DIP | Extension | 25.1 | 33.7 | <0.001* | -4.526 |
| Right 2nd PIP | Extension | 33.7 | 39.1 | <0.001* | -2.842 |
| Left 2nd PIP | Extension | 35 | 38.2 | 0.1 | -1.833 |
| Right 3rd PIP | Extension | 34 | 39.4 | <0.001* | -2.842 |
| Left 3rd PIP | Extension | 33.8 | 38.3 | <0.001* | -2.444 |
| Right 4th PIP | Extension | 34.1 | 40.2 | <0.001* | -3.389 |
| Left 4th PIP | Extension | 33.6 | 38.6 | <0.001* | -2.777 |
| Right 5th PIP | Extension | 34.3 | 37.8 | 0.1 | -1.842 |
| Left 5th PIP | Extension | 33.9 | 37.7 | <0.001* | -2.111 |
| Right 1st MCP | Extension | 60.7 | 62 | 0.6 | -0.518 |
| Left 1st MCP | Extension | 64.3 | 63.4 | 0.7 | 0.333 |
| Right 2nd MCP | Extension | 68.1 | 64.2 | 0.1 | 1.52 |
| Left 2nd MCP | Extension | 68.8 | 66.8 | 0.4 | 0.875 |
| Right 3rd MCP | Extension | 63.5 | 61.4 | 0.4 | 0.84 |
| Left 3rd MCP | Extension | 64.1 | 62.7 | 0.5 | 0.608 |
| Right 4th MCP | Extension | 61.7 | 60.1 | 0.5 | 0.696 |
| Left 4th MCP | Extension | 62.5 | 60.7 | 0.4 | 0.75 |
| Right 5th MCP | Extension | 62.9 | 60.4 | 0.3 | 1.041 |
| Left 5th MCP | Extension | 64.3 | 61.3 | 0.2 | 1.25 |
| Right wrist | Extension | 84.7 | 86.3 | 0.4 | -0.842 |
| Left wrist | Extension | 84 | 87 | 0.1 | -1.55 |
| Right elbow | Extension | 0 | 0 | 0.1 | -1.518 |
| Left elbow | Extension | 0 | 0 | 0.2 | -1.444 |
| Right ankle | Dorsiflexion | 26.2 | 26.7 | 0.6 | -0.454 |
| Left ankle | Dorsiflexion | 26.2 | 27.2 | 0.4 | -0.833 |
| Cervical spine | Right lateral flexion | 34.5 | 36.3 | 0.1 | -1.5 |
| Cervical spine | Left lateral flexion | 35.1 | 36.4 | 0.3 | -1.083 |
| TL spine | Right lateral flexion | 32.1 | 33.3 | 0.3 | -0.923 |
| TL spine | Left lateral flexion | 31.6 | 32.7 | 0.4 | -0.833 |

Abbreviations: ROM- Range of motion, IP- Interphalangeal, PIP- Proximal interphalangeal, DIP- Distal interphalangeal, MCP-Metacarpophalangeal, TL- Thoracolumbar
*Significant at $p$-value < 0.05

### P69

### Genotype-Phenotype correlations in a study of 294 patients with osteogenesis imperfecta

Maegen Wallace[1], Danita Velasco[2], Jay Byrd[2], Andrew White[2], Makayla Schissel[2], Matthew Van Ormer[2]

[1]Phoenix Children's Hospital, Phoenix, USA [2]University of Nebraska Medical Center, Omaha, USA

### Abstract

**OBJECTIVES**:

Osteogenesis imperfecta (OI) is an inherited disorder characterized by bone fragility with extraskeletal manifestations. Mutations in *COL1A1* and *COL1A2* account for most OI, yet non-*COL1A1/2* mutations are responsible for many cases. There are now 16 widely accepted genes causing OI; however, literature on genotype-phenotype correlation and incidence of non-skeletal clinical features are limited. This study aims to identify genotype-phenotype correlations in patients with OI, allowing clinicians to better inform families of prognosis, optimize patient care, and facilitate evidence-based clinical decision-making.

**METHODS:**

A retrospective review of 294 patients with OI was performed to collect demographic data, clinical characteristics, genotypic information, and phenotypic information. Patients were stratified by *COL1A1/2* vs. non-*COL1A1/2* mutations to evaluate differences in phenotype. Data were summarized with descriptive statistics, followed by Chi-square or Fisher's Exact tests for categorical variables and independent samples t-tests or Wilcoxon Rank sum tests for continuous variables. P <0.05 was considered statistically significant.

**RESULTS:**

Most cases were due to mutations in *COL1A1/2* (91%), with the remaining 9% due to non-*COL1A1/2* mutations. 137 (51.1%) are male and 131 (48.9%) are female. Most patients in the *COL1A1/2* group were white compared to the non-*COL1A1/2* group (78% vs. 50%; p=0.004). Blue sclerae were significantly associated with genotype, being present in 83% and 50% of those with *COL1A1/2* and non-*COL1A1/2* mutations, respectively (p=0.002). Dentinogenesis imperfecta was also more common in the *COL1A1/2* versus the non-*COL1A1/2* group (49% vs. 15%, p<0.001). Of those in the *COL1A1/2* group, 34% have a family history of OI, compared to only 12% of those in the non-*COL1A1/2* group (p=0.03). Those in the non-*COL1A1/2* group have higher rates of scoliosis compared to those in the *COL1A1/2* group (62% vs. 40%, p=0.04). Expressive language disorder/delay was also significant, with a prevalence of 15% and 0.4% in non-*COL1A1/2* and *COL1A1/2* patients, respectively (p<0.001).

**CONCLUSIONS:**

Identifying the underlying molecular etiology early is imperative for optimal clinical care, allowing for appropriate risk counseling, identification of affected relatives, and improved anticipatory care and management. This data supports that rare subtypes of OI occur more frequently in non-White individuals and that there is a correlation with the incidence of blue sclera, dentinogenesis imperfecta, scoliosis, and expressive language disorders.

### P70

### Six new pathogenic variants in the GORAB gene in three patients with osteodysplastic gerodermia

Elena Merkuryeva[1], Tatiana Markova[1], Vladimir Kenis[2]

[1]Research Centre for Medical Genetics, Moscow, Russian Federation. [2]H. Turner National Medical Research Center for Children's Orthopedics and Trauma Surgery, Saint Petersburg, Russian Federation

**Abstract**

Osteodysplastic gerodermia (OMIM #231070) is a rare autosomal recessive disorder characterized by congenital wrinkled skin and pronounced osteoporosis in childhood. The disease is caused by pathogenic variants in the *GORAB* gene. The rarity of the syndrome, as well as the similarity of clinical manifestations to a heterogeneous group of hereditary diseases accompanied by decreased skin elasticity, leads to a delayed diagnosis.

The aim of the present study was to investigate the molecular and clinico-radiological features of three patients aged three to sixteen years with osteodysplastic gerodermia, caused by newly identified compound heterozygous variants in the *GORAB* gene.

The study material consisted of data from a comprehensive examination of three patients. To refine the diagnosis, a complex of examination methods was used: genealogical analysis, clinical examination, standard neurological examination, radiography of the spine, hip joints, and tubular bones of the limbs, complete genome sequencing, and direct automatic Sanger sequencing.

We report detailed clinico-radiological and genetic characteristics in these patients with new nucleotide variants in the *GORAB* gene in a compound heterozygous state: c.170C>G (p.Ser57Ter) and c.790G>C (p.Ala264Pro); c.295C>T (p.Gln99Ter) and a heterozygous deletion on chromosome 1 (chr1:g.170531967-170539494del), affecting the 1st and 2nd exons of the gene; c.15G>C (p.W5C) and a heterozygous deletion on chromosome 1 (chr1:g. 170530069-170541390del), affecting the 1st and 2nd exons *GORAB.*

The main clinical manifestations of the syndrome were congenital wrinkled skin, sagging cheeks, malar hypoplasia, prognathism, muscular hypotonia, and joint hypermobility. Radiological signs in all three patients included congenital bilateral hip dislocation, compressive vertebral fractures. Bisruption of the continuity of the epiphyseal growth plate of the distal femur and proximal tibia in the form of an "insertion" type was diagnosed in male patients aged three years eight months and eight years ten months.

Osteodysplastic gerodermia is an extremely rare disease that requires constant dynamic monitoring and interdisciplinary interaction among specialists of different profiles. Although the clinico-radiological picture of this disease is quite specific, molecular diagnostics within this symptom complex may require full-genome sequencing to search for intragenic deletions. The results of the conducted study expand the spectrum of nucleotide variants in the *GORAB* gene.

### P71

### New insights from six cases of Spondylodysplastic Ehlers Danlos Syndrome

Rhoda Akilapa[1], Fleur Van Djik[2], Deborah Shears[3] , Laura Watts[3], Lucy Loong[3], Alistair Calder[4], Sher-Bano Syed[2], Juliette Harris[2], Neeti Ghali[2]

[1]South East Thames Regional Genetics Service, Guy's & St Thomas' Hospital, London, UK [2]North West Thames Regional Genetics Service, Northwick Park Hospital, London, UK [3]Oxford Centre for Genomic Medicine, Oxford University Hospitals, Oxford, UK [4]Great Ormond Street Hospital for Children, London, United Kingdom

### Abstract

**Background**:

Spondylodyplastic type Ehlers Danlos syndrome (spEDS) is a rare autosomal recessive disorder characterised by short stature, muscular hypotonia, bowing of the long bones, skin hyperextensibility and pes planus. The condition is caused by biallelic pathogenic variants in *B4GALT7*, *B3GALT6* or *SLC39A13*. There is overlap in the characterisation of these disorders, with *B3GALT6* and *B4GALT7*-related spEDS being included in the skeletal dysplasia 2023 nosology as part of group 5- 'Dysplasias with multiple joint dislocations'. *SLC39A13*-related spEDS is included in group 13-'Spodyloepimetaphyseal dysplasias'.

The combination of a connective tissue and skeletal phenotype brings many of these patients to the attention of the Paediatric and Genetics services for investigation and diagnosis. In some cases, the skeletal phenotype predominates. This can range from an antenatal appearance of a severe skeletal dysplasia, leading to second trimester termination to a postnatal progressive kyphoscoliosis.

**Presenting problem:**

We report six patients from four families presenting with spEDS with a focus on clinical presentation and radiological findings. In three of these patients, variants in *B3GALT6* confirmed the diagnosis with clinical features in keeping with previously reported cases. Presenting features included fixed flexion deformities of the limbs, proportionate short stature with normal head circumference and rapidly progressive kyphoscoliosis in two patients. The presence of multiple haemangiomas in patient 1, is a novel finding and may expand the phenotype in this condition. In two siblings, a variant in *SCL39A13* is thought to be causative. Interestingly, the maternal carrier displayed mild features raising the possibility of heterozygous phenotypic expression.

**Clinical Management:**

Rapidly progressive scoliosis in patient 3 was treated in plastic spinal jacket. She was deemed not suitable for surgery in view of her small size and lack of subcutaneous tissue. Convex spinal fusion is being considered but this will limit future spinal growth. Recurrent knee subluxations in our adult patient (patient 6) with B4GALT7-related spEDS was treated with orthopaedic surgery to stabilise the muscles around knee joint. This achieved 8 years free of subluxations before a recurrence. A DEXA scan was done as part of a research study which revealed low bone mineral density (z score -3.9). No history of compression fractures was reported. Mild learning difficulties was prevalent in this cohort. Patients seemed to make steady progress with additional education support. For patient 6, counselling for future off-spring risk proved difficult, as no successful pregnancies in B4GALT7- related spEDS have been reported to our knowledge.

**Discussion:**

These cases highlight the distinctive features that set apart the more prevalent *B3GALT6* and *B4GALT7* spEDS phenotype from the rarer cases of *SLC39A13* in the literature. The small number of *SLC39A13*- related spEDS makes variant interpretation challenging due to a milder phenotype and subsequent uncertainty when applying phenotypic weight to ACMG guidelines. The multisystem involvement of spEDS necessitates a multidisciplinary approach to treatment and ongoing management.

### P72

### Expanding the phenotype of PLS3-related osteoporosis

Tashunka Taylor-Miller[1], Peter Simm[2], Margaret Zacharin[2], Alison Yeung[1]

[1]Victorian Clinical Genetics Service, Melbourne, Australia. [2]Royal Children's Hospital, Melbourne, Australia

### Abstract

**Background:**

The *PLS3* gene encodes for Plastin 3 (PLS3) protein which is involved in the development of collagen and actin-binding throughout the body. It is expressed in all solid tissue and appears to have a 'dosage effect.' PLS3-related X-linked osteoporosis has a skeletal phenotype, but no extra-skeletal manifestations have been described in mice or humans. Here we expand the PLS3-related phenotype by describing two patients; one with a novel stop-gain variant, the second with an Xp23 deletion containing *PLS3*, and vascular and ophthalmologic features.

**Presenting problem:**

**Case 1**: This 12 year-old male presented with back pain and two previous minimal trauma long-bone fractures. He had mild small-joint hypermobility and thoracic spine kyphosis. Imaging revealed multiple thoracic wedge fractures, and low bone density.

**Case 2:** This 6-month-old male presented with epiphora and photophobia, was diagnosed with bilateral congenital glaucoma and mild posterior subcapsular cataracts. At 10 years-old he had additional features of amelogenesis imperfecta, short stature, and grey-blue sclera.

Family history of maternal Madelung deformity (due to a deletion Xp22.33 containing SHOX gene) and a diagnosis of osteogenesis imperfecta and fatal aortic aneurysm dissection in maternal grandmother and uncle in whom an Xq23 deletion containing the PLS3 gene was identified. The patient underwent multiple corrective ophthalmologic interventions during infancy. Imaging in adolescence revealed L4 vertebral wedge fracture and low bone density. Echocardiogram revealed mild dilation of his aortic root and ascending aorta.

**Discussion:**

The *PLS3* gene is located on the X-chromosome. Reported males with variants in this gene are typically more severely affected with childhood-onset osteoporosis and bone fragility compared to heterozygous females who have a varied bone-phenotype ranging from normal bone density to multiple fractures with mild osteopenia. PLS3 displays tissue-specific expression and dosage effects[4]. Pathogenic variants in *PLS3* were first described by van Dijk and colleagues in 2013, and various pathogenic mechanisms have been described including whole-gene deletion, frameshift deletions, insertion, indel, missense, nonsense, and splice-site variants[1–5].

Cardiac (other than a patent ductus arteriosis[1]) and ophthalmological features have not previously been characterised in PLS3-related osteopenia cohorts. However, *PLS3* variants have been reported in Keratoconus[6], a complex corneal disorder, and S-nitrosylation of PLS3-protein plays a role in the development of thoracic aortic dissection[7].

**Table 1. Relevant available investigations**

| | Case 1 | Case 2 | Maternal uncle of case 2 |
|---|---|---|---|
| Current age | 15 years old | 13 years old | Deceased 28 years old |
| Height z-score | - 0.4 SD | | |
| X-ray spine | Osteopenia and multiple wedge fractures involving T5-T12 vertebra | L4 vertebral wedge fracture | - |
| Bone Densitometry | At 12 years old- Height adjusted lumbar spine BMD Z score -4.7 SD | At 12 years old- Vertebral fracture analysis demonstrated a mild (22%) loss of posterior<br><br>vertebral height at the L4 vertebrae. Height adjusted Lumbar spine BMD Z score -1.0 SD. | |
| Molecular genetic diagnosis | Exome capture gene panel: hemizygous pathogenic variant PLS3 c.352C>T (p.Gln118*) | Microarray:<br><br>Deletion Xp22.33 containing SHOX gene (maternally inherited)<br><br>Deletion Xq23 containing PLS3 gene<br><br>(maternally inherited) | Microarray:<br><br>Deletion Xp22.33 containing SHOX gene<br><br>Deletion Xq23 containing PLS3 gene<br><br>Panel of aortopathy genes: negative |

### References

1. S. van DF, Carola ZM, Dimitra M, Markus R, L.M. MC, E. de DSC, et al. PLS3 Mutations in X-Linked Osteoporosis with Fractures. N Engl J Med. 2013;369(16):1529–36.

2. Kämpe AJ, Costantini A, Levy-shraga Y, Zeitlin L, Roschger P, Taylan F, et al. PLS3 Deletions Lead to Severe Spinal Osteoporosis and Disturbed Bone Matrix Mineralization. J Bone Miner Res. 2017;32(12):2394–404.

3. Mäkitie RE, Niinimäki T, Suo-Palosaari M, Kämpe A, Costantini A, Toiviainen-Salo S, et al. PLS3 Mutations Cause Severe Age and Sex-Related Spinal Pathology. Front Endocrinol. 2020;11:393.

4. Wolff L, Strathmann EA, Müller I, Mählich D, Veltman C, Niehoff A, et al. Plastin 3 in health and disease: a matter of balance. Cell Mol Life Sci. 2021;78(13):5275–301.

5. Kannu P, Mahjoub A, Babul-Hirji R, Carter MT, Harrington J. PLS3 Mutations in X-Linked Osteoporosis: Clinical and Bone Characteristics of Two Novel Mutations. Horm Res Paediatr. 2017;88(3–4):298–304.

6. Xu L, Yang K, Zhu M, Yin S, Gu Y, Fan Q, et al. Trio-based exome sequencing broaden the genetic spectrum in keratoconus. Exp Eye Res. 2023;226:109342.

7. Pan L, Lin Z, Tang X, Tian J, Zheng Q, Jing J, et al. S-Nitrosylation of Plastin-3 Exacerbates Thoracic Aortic Dissection Formation via Endothelial Barrier Dysfunction. Arter, Thromb, Vasc Biol. 2019;40(1):175–88.

P73

### Acromicric dysplasia caused by a novel mutation in FBN1

Chan Jong Kim

Department of Pediatrics, Chonnam Univ. Hospital, Gwangju, Republic of Korea

### Abstract

**BACKGROUND:**

Acromicric dysplasia (AD) is a rare heritable severe short stature syndrome with the presence of joint stiffness and shortened hands and feet. Patients are born with a normal height but progressively drop down the percentiles in childhood. Radiological features include delayed bone age, cone-shaped epiphyses, and ovoid vertebral bodies. Other distinct skeletal features are internal and external notches of the femoral head and certain metacarpals. Heterozygous mutations in the Fibrillin-1 (FBN1) gene have been proposed as the predominant cause of this disease. Most reported patients only showed mild phenotype, but severe cases also existed.

**PRESENTING PROBLEM and CLINICAL MANAGEMENT:**

Here, we describe a 13-year-old Korean girl with AD who was born to non-consanguineous parents. She was referred to our hospital because of short stature. She had been born via vaginal delivery at a gestational age of 38 weeks with normal birth weight (2800 gram), but had shown progressive post-natal growth failure. She showed short stature for her age (below $3^{rd}$ percentile), short and stubby hands and feet, normal intelligence, and mild facial dysmorphisms. Growth hormone provocation test, chromosomal study, and FGFR3 gene study revealed normal. Radiographic study presented delayed bone age and mild brachymetacarpia. We conducted whole-exome sequencing (WES) to investigate multiple genes associated with skeletal dysplasia. The WES results revealed a heterozygous novel variant, p.Cys1733Trp, in FBN1, which was validated by Sanger sequencing. The parents did not have this variant.

**DISCUSSION:**

AD is a very rare skeletal dysplasia with a prevalence of <1 of 1,000,000 with only about 60 cases being reported worldwide. Accurate diagnosis of this disorder may be the essential to offering the best care to the patients, especially as patients with short stature due to a skeletal dysplasia. This case broadens the phenotypic spectrum of growth disorders associated with FBN1 mutations.

**P74**

### Transmembrane Protein 53 (TMEM53) gene deactivation underlies craniotubular dysplasia

Michael P Whyte[1,2], Robert S Weinstein[3], Paul H Phillips[4], Rongsheng Cai[4], G Bradley Schaefer[4], Michelle A Hutchison[4], Gary S Gottesman[1,2], William H McAlister[5], Shenghui Duan[2], Steven Mumm[1,2]

[1]Center for Metabolic Bone Disease and Molecular Research, St. Louis, USA [2]Division of Bone and Mineral Diseases, Washington University School of Medicine, St. Louis, USA [3]Division of Endocrinology, Metabolic Bone Diseases, University of Arkansas for Medical Sciences, Little Rock, USA [4]Arkansas Children's Hospital, Little Rock, USA [5]Mallinckrodt Institute of Radiology at St. Louis Children's Hospital, Washington University School of Medicine, St. Louis, USA

### Abstract

**Objectives**: We were referred a 13-year-old American boy in 2018 for a generalized skeletal disorder including progressive vision and hearing loss due to cranial foramina encroachment by bone. Steroid therapy for 6 months was ineffective, whereas optic nerve decompressions by curettage of soft overgrown vascular bone improved his vision. Radiographic skeletal survey showed bone tissue accumulation at his skull base, but generalized osteopenia elsewhere. His calvarium and facial bones were widened whereas his optic, auditory, and spinal canals were narrowed. DXA bone density was normal for the whole body assessment, but low in his spine and hip. Biochemical studies of mineral metabolism were unremarkable and bone turnover markers indicated active bone formation. Tetracycline-labeled iliac crest histomorphometry revealed abundant widely spaced "double labels" consistent with twice the expected rate of bone formation. Then, vision loss did not progress during prednisone and zoledronate treatment. The objective was to identify his genetic defect causing this seemingly unique skeletal disorder, to understand the mechanism of disease and to help guide therapy.

**Methods**: The patient's genomic DNA was analyzed using an Ion Torrent panel (Thermo Fisher Scientific) of 35 genes underlying high bone turnover or high bone mass, and whole genome sequencing (WGS; Illumina).

**Results**: The gene panel was negative. Trio WGS subsequently identified in *TMEM53*: i) a paternally inherited heterozygous 54-base deletion, including the mRNA splice acceptor site for exon 2, and 31 bases of exonic sequence (c. 62-23_92), and ii) a maternally inherited extremely rare, (gnomAD frequency = 0.000036), heterozygous missense variant (c.650C>T, p.Ser217Leu). In silico missense prediction models, SIFT and Mutation Taster, scored this variant as damaging. Ser217 is highly conserved across species. Craniotubular dysplasia (OMIM # 619727) denotes the disorder, reported in 2021 from investigation of four Indian families, caused by a homozygous 4-base exonic insertion or a homozygous small deletion at the exon 2 splice acceptor site within *TMEM53*. *TMEM53* encodes nuclear envelope transmembrane protein 53 involved in BMP-SMAD signaling.

**Conclusion**: Bi-allelic deactivation of *TMEM53* causes what is classified as a sclerosing bone disorder, "craniotubular dysplasia." We find generalized features of poor bone quality and propose "*TMEM53*-associated skeletal disease".

### P75

### To study the association of lifestyle factors with Vitamin D and PTH concentrations in rural prepubertal children: A pilot genome-wide association study

Sharvani Patil[1], Neha Kajale[1,2], Vivek Patwardhan[1], Chirantap Oza[1], Shruti Mondkar[1], Chidvilas More[1], Dushant Baghel[3], Anuradha Khadilkar[1,2]

[1]Hirabai Cowasji Jehangir Medical Research Institute, Jehangir Hospital, Pune, India. [2]School of Health Sciences Savitribai Phule Pune University, Pune, India. [3]Nucleome Informatics Private Limited, Hyderabad, India

### Abstract

**Objectives:**

It is crucial to assess vitamin D and parathormone(PTH) concentrations in prepubertal children for bone health, growth, immunity, and overall well-being. Several Indian studies have investigated factors related to vitamin D and PTH levels in children; however, single nucleotide polymorphisms (SNPs) within genes encoding proteins involved in various stages of their metabolism may also impact these hormones. Therefore, the objective of our pilot study were- 1) To study lifestyle factors associated with vitamin D and PTH concentrations. 2) To identify the Single Nucleotide Variants associated with Vitamin D and PTH.

**Methods:**

Pilot, observational, cross-sectional study, n=60 (mean age=9.5±1.0years). 25 (OH)vitamin D and intact PTH ( Beckman Coulter Access 2 immunoassay system) were measured. SNP genotyping of the blood samples was performed using the Precision Medicine Research Array (process included-Genomic DNA Isolation, quality control(QC), sample preparation, DNA Amplification, Fragmentation & Precipitation, Drying, Resuspension & QC, Denaturation & Hybridization, Ligation, Staining & Stabilization. Bioinformatic analysis was performed( Axiom Analysis Suite software package). R package was used for enhanced computation and checking for the association of identified SNPs with the phenotype. General Linear Model(GLM), Mixed Linear Model(MLM) and Fixed and random model Circulating Probability Unification (FarmCPU) models were used.

**Results:**

Mean vitamin D and PTH concentrations were 26.7±9.4 ng/ml and 33.1±18.6 pg/ml (ref range < 20 ng/ml deficient 20- 30 ng/ml insufficient 30-100 ng/ml sufficient and ref range- 12 to 88 pg/ml) respectively. 21% participants were deficient, 47% insufficient and 32% were sufficient. There was a significant positive correlation between vitamin D concentrations and sunlight exposure (rs=0.270, $p$=0.028) and dietary calcium (rs=0.262, $p$=0.034). PTH showed a positive correlation with dietary protein (rs=0.242, $p$=0.040). In all, 30 significant ($p<10^{-5}$)SNPs for vitamin D and 20 for PTH were reported; 5 were associated with genes coding for associated proteins. (table as below)

| SNP ID | CHROM | β | SE | *P* value | Gene | Influence |
|---|---|---|---|---|---|---|
| rs2304026 | 5 | 25.576 | 3.463 | $7.25 \times 10^{-10}$ | *FAT2 and SLC36A* | Fatty acid and folate transport |
| rs62446990 | 7 | 39.441 | 4.829 | $3.62 \times 10^{-11}$ | *HDAC9* | Encode for histone deacetylase affecting gene expression |
| rs150870306 | 7 | 39.44119065 | 4.828782619 | $3.62 \times 10^{-11}$ | *KCND2* | Encode for potassium channel protein |
| rs55890839 | 12 | 12.41497 | 2.543543 | $9.78 \times 10^{-9}$ | *CRACR2A* | Encodes a calcium release-activated channel protein. |
| rs115637886 | 3 | 63.03044 | 10.00031 | $4.57 \times 10^{-8}$ | *ITPR1* | Encodes inositol 1,4,5-trisphosphate receptor 1, involved in calcium release from intracellular stores |

**Conclusions:**

To the best of our knowledge, no GWAS studies have been conducted for investigating Vitamin D and PTH concentrations in Indian prepubertal children. Preliminary results show 50 significant SNPs associated with vitamin D and PTH concentrations. These SNPs need to be further explored for a better understanding of genetic drivers of vitamin D and PTH.

**P76**

Withdrawn

### P77

### Safety of alternating denosumab and zoledronate for osteonecrosis of the femoral head in children

Joel Vanderniet[1,2], Christie-Lee Wall[1], Suzie Taylor[1], Myra Poon[1,2], Andrew Biggin[1,2], Craig Munns[3,4]

[1]The Children's Hospital at Westmead, Sydney, Australia. [2]The University of Sydney, Sydney, Australia. [3]University of Queensland, Brisbane, Australia. [4]Queensland Children's Hospital, Brisbane, Australia

### Abstract

### Objectives

To report the safety of alternating denosumab and zoledronate therapy in children with osteonecrosis of the femoral head (OFH). Following institutional Drug Committee approval, this protocol was developed because previous protocols of 12-weekly and 8-weekly denosumab followed by 2 doses of zoledronate resulted in several cases of severe rebound hypercalcaemia.

### Methods

Denosumab 1mg/kg (maximum 60mg) and zoledronate 0.025mg/kg (maximum 2mg) were alternately administered at 12-week intervals in Group 1 and alternate dosing interval increasing from 4- to 12-weeks over 16 weeks in group 2, for total 66 weeks treatment in both groups. Calcium 500mg twice daily and cholecalciferol 1000IU daily were administered throughout treatment. Serum calcium, phosphorus, alkaline phosphatase and parathyroid hormone were measured at least 6-weekly from baseline to 78 weeks. Bone mineral density (BMD) was measured using dual-energy x-ray absorptiometry (DXA) at baseline and completion of treatment. Dental review was performed at baseline and regularly throughout treatment. Data are presented as mean or median (range).

### Results

Twenty-five patients (14 in Group 1, 11 in Group 2) completed the 78-week protocol. Median age at start was 10 years (5-15). Eight had OFH secondary to Legg-Calve-Perthes disease, 7 to slipped capital femoral epiphysis, 4 to femoral neck fracture, 1 to hip dislocation, 3 to high-dose corticosteroids (chemotherapy) and 2 to sickle cell disease.

Seven (28%) had asymptomatic hypocalcaemia 4 weeks after the first denosumab, but only 3 had calcium <2.00mmol/L (1.77-1.95mmol/L; reference range 2.20-2.65). All hypocalcaemia resolved with increased oral calcium supplementation. Six (24%) had mild asymptomatic hypercalcaemia while on treatment or after discontinuation, calcium 2.66-2.85mmol/L, and all resolved without intervention. There was no hypophosphataemia.

Mean sex/height-adjusted total body BMD Z-score increased from -0.4 (-2.3-1.4) to 0.7 (-1.5-2.3) and L2-L4 Z-score from -0.3 (-2.8-1.5) to 1.5 (-1.7-3.3) from baseline to end of treatment. Z-scores exceeded 2.0 in 5 patients (20%). There was no osteonecrosis of the jaw.

**Conclusions**

With careful monitoring, alternating denosumab and zoledronate appears to be safe in children with OFH. Biochemical disturbances were mild and mostly self-resolving. There was no significant rebound hypercalcaemia. BMD increased during treatment but is expected to decrease following discontinuation of antiresorptives. No dental abnormalities were reported.

### Disclosure

Craig Munns has participated in clinical trials with Amgen, with funds to his institution.

### P78

### Persistence of growth promoting effects in infants and toddlers with achondroplasia: Results from a phase 2 extension study with Vosoritide

Ravi Savarirayan[1], William R Wilcox[2], Paul Harmatz[3], John Phillips III[4], Melita Irving[5], Lynda E Polgreen[6], Louise Tofts[7], Keiichi Ozono[8], Paul Arundel[9], Carlos Bacino[10], Donald Basel[11], Ricki S Carroll[12], Joel Charrow[13], Hiroshi Mochizuki[14], Yumiko Kotani[15], Howard M Saal[16], Lynn Han[17], Andrea Low[17], Jonathan Day[18]

[1]Murdoch Children's Research Institute, Royal Children's Hospital, and University of Melbourne, Parkville, Australia. [2]Emory University, Atlanta, USA [3]UCSF Benioff Children's Hospital, Oakland, USA [4]Vanderbilt University Medical Center, Nashville, USA [5]Guy's and St. Thomas' NHS Foundation Trust, London, UK [6]Lundquist Institute for Biomedical Innovation at Harbor-UCLA Medical Center, David Geffen School of Medicine, Torrance, USA [7]Kids Rehab, The Children's Hospital at Westmead, Westmead, Australia. [8]Osaka University Hospital, Osaka, Japan. [9]Sheffield Children's NHS Foundation Trust, Sheffield Children's Hospital, Sheffield, UK [10]Baylor College of Medicine, Houston, USA [11]Medical College of Wisconsin, Milwaukee, USA [12]Nemours/Alfred I. du Pont Hospital for Children, Wilmington, USA [13]Ann and Robert H. Lurie Children's Hospital of Chicago, Chicago, USA [14]Saitama Children's Hospital, Saitama, Japan. [15]Tokushima University Hospital, Tokushima, Japan. [16]Cincinnati Children's Hospital Medical Center, Cincinnati, USA [17]BioMarin Pharmaceutical Inc., Novato, USA [18]BioMarin (U.K.) Limited, London, UK

### Abstract

### Introduction

We recently reported results from a phase 2, randomized, double-blind, placebo-controlled study (111-206) of vosoritide in young children with achondroplasia (ACH) aged 3 months – <5 years. Here we report results from the open-label extension study (111-208) spanning up to 4 years of treatment in children aged <5 years at start of vosoritide therapy.

### Methods

Primary objectives are evaluation of long-term safety and tolerability, and change in height z-score (referenced to CDC average stature growth charts). Data were analysed by age at treatment initiation (≥ 2 years and < 2 years cohorts). Comparative cross-sectional and longitudinal analyses were performed for each year of follow-up using two independent external controls: (1) observational/placebo data from vosoritide trials, and (2) the CLARITY natural history dataset.

### Results

Of 73 participants who entered the extension study, 67 received the first dose of vosoritide aged <5 years. Of the 66 who had ≥ 1 year of treatment follow-up, mean (SD) height z-score at baseline was -4.72 (1.04) for the 34 children aged ≥ 2 years at start of vosoritide and -3.56 (0.84) for the 32 children <2 years of age at vosoritide initiation. Vosoritide remained well-tolerated with no change in adverse event profile and no discontinuations due to treatment-related adverse events. Treated children consistently demonstrated greater height gain over time compared to matched observational/placebo controls and to matched untreated children from CLARITY, with mean height z-score increases ranging from 0.55-0.86 SDS after 3 years and 1.10-1.42 SDS after 4 years in the ≥ 2 year age cohort, and 0.79-0.98 SDS after 3 years in the <2 year age cohort. Gains in absolute height, relative to those of matched children of average stature, were 86% at Year 3 and 90% at Year 4 for treated children in the ≥ 2 year age cohort (versus 66% for untreated children in Years 3 and 4) and 80% for treated children in the <2 year age cohort at Year 3 (versus 67% for untreated).

**Conclusions**

Vosoritide was well tolerated and maintained consistent positive effects on linear growth over time in children who started treatment aged <5 years.

### Disclosure

All authors are investigators in this clinical trial with the exception of LH, AL, EF, and JD, who are employees of BioMarin. RS and LT have received consulting fees and grants from BioMarin. MI and WRW have received consulting fees from BioMarin. JC and DB have received grants from BioMarin. LEP, PA and RC have received honoraria from BioMarin. CB and PH have received consulting fees, honoraria, and grants from BioMarin. Other authors declare no competing interests.

### P79

### Persistent growth-promoting effects of vosoritide in children with achondroplasia for up to 4 years: Update from phase 3 extension study

Ravi Savarirayan[1], Louise Tofts[2], Melita Irving[3], William R Wilcox[4], Carlos A Bacino[5], Julie E Hoover-Fong[6], Paul Harmatz[7], Frank Rutsch[8], Ricki S Carroll[9], Lynda E Polgreen[10], Klaus Mohnike[11], Joel Charrow[12], Carlos Prada[12], Daniel Hoernschemeyer[13], Keiichi Ozono[14], Takuo Kubota[14], Yasemin Alanay[15], Paul Arundel[16], Yumiko Kotani[17], Natsuo Yasui[17], Klane K White[18], Shelley Brandstetter[18], Howard M Saal[19], Antonio Leiva-Gea[20], Hiroshi Mochizuki[21], Asako Tajima[22], Donald Basel[23], Elena Fisheleva[24], Andrea Low[25], Sue Lawrinson[24], Jonathan Day[24]

[1]Murdoch Children's Research Institute, Royal Children's Hospital, and University of Melbourne, Parkville, Australia. [2]Kids Rehab, The Children's Hospital at Westmead, Westmead, Australia. [3]Guy's and St. Thomas' NHS Foundation Trust, London, UK [4]Emory University, Atlanta, USA [5]Baylor College of Medicine, Houston, USA [6]Johns Hopkins University School Of Medicine, Baltimore, USA [7]UCSF Benioff Children's Hospital, Oakland, USA [8]Department of General Pediatrics, Muenster University Children's Hospital, Münster, Germany. [9]Nemours/Alfred I. du Pont Hospital for Children, Wilmington, USA [10]Lundquist Institute for Biomedical Innovation at Harbor-UCLA Medical Center, David Geffen School of Medicine, Torrance, USA [11]Otto-von-Guericke Universität, Universitätskinderklinik, Magdeburg, Germany. [12]Ann and Robert H. Lurie Children's Hospital of Chicago, Chicago, USA [13]University of Missouri-Columbia, Columbia, USA [14]Osaka University Hospital, Osaka, Japan. [15]Acibadem Mehmet Ali Aydinlar University, School of Medicine, Istanbul, Turkey. [16]Sheffield Children's NHS Foundation Trust, Sheffield Children's Hospital, Sheffield, UK [17]Tokushima University Hospital, Tokushima, Japan. [18]Colorado Children's Hospital, Colorado, USA [19]Cincinnati Children's Hospital Medical Center, Cincinnati, USA [20]Hospital Universitario Virgen de la Victoria, Málaga, Spain. [21]Saitama Children's Hospitals, Saitama, Japan. [22]Saitama Children's Hospital, Saitama, Japan. [23]Medical College of Wisconsin, Milwaukee, USA [24]BioMarin (UK) Limited, London, UK [25]BioMarin Pharmaceutical Inc., Novato, USA

### Abstract

**Objectives:** Vosoritide, a potent stimulator of endochondral bone growth, is approved for use in children with achondroplasia (ACH).  We report available long-term data from the ongoing phase 3 open-label extension study.

**Methods:** 119 children were enrolled and received open label vosoritide. AGV, height z-score, body proportion ratio and rate of adverse events (AEs) were analyzed. At time of data cut, 3-year data was complete with extensive follow-up at 4 years.

**Results:** Mean age- and sex-specific AGVs were consistently higher in children treated with vosoritide compared to untreated controls from the CLARITY ACH natural history data set.  Mean AGVs of treated children were comparable to that of average stature children prior to puberty but were maintained over a longer duration. There was no apparent pubertal growth spurt. The mean (SD) of the mean differences in AGV between treated and untreated children across integer ages 6 – 17 years was 1.46 (0.61) cm/year in girls and 1.73 (0.52) cm/year in boys. Mean (SD) change from baseline height z-score was +0.23 (0.32) at Week 52

(n=111), +0.59 (0.73) at Week 156 (n=111) and +0.83 (0.69) at Week 208 (n=53) relative to an average stature population (CDC), and +0.32 (0.20) at Week 52 (n=111), +0.89 (0.44) at Week 156 (n=111) and +1.17 (0.42) at Week 208 (n=53) relative to an untreated ACH population (CLARITY). Mean (SD) upper to lower body segment ratio improved with change from baseline of -0.03 (0.10) at Week 52 (n=104), to -0.10 (0.12) at Week 156 (n=105) and -0.15 (0.14) at Week 208 (n=52). Most AEs were mild. Two participants had 3 serious AEs (SAEs) assessed as related to vosoritide: an SAE of genu valgum and kyphoscoliosis in 1 participant, and femur fracture in 1 participant. The SAE of kyphoscoliosis led to discontinuation of vosoritide. There was no evidence of a negative effect of vosoritide on body proportions, bone mineral density, or bone age.

**Conclusions:** The effect of vosoritide on growth continues to be maintained in children with ACH aged 5 to 18 years for at least 3 years. The long-term safety profile remains favourable without any treatment-limiting adverse events.

### Disclosure

All authors are investigators in this clinical trial except for EF, AL, SL and JD, who are employees of the funder (BioMarin). RS, LT, FR, and KM have received consulting fees and grants from BioMarin. MI and WW have received consulting fees from BioMarin. JC and DB have received grants from BioMarin. LP, PA and RC have received honoraria from BioMarin. CB and PH have received consulting fees, honoraria and grants from BioMarin. JHF has received consulting fees from BioMarin, Therachon AG and Ascendis, and grants from BioMarin. KW has received consulting fees from BioMarin and Sanofi/Genzyme, and grants from BioMarin, Ultragenyx, and Ascendis. The other authors declare no competing interests.

### P80

### Follow-up of 13 paediatric patients treated with Denosumab and Zoledronate for Aneurysmal Bone Cyst (ABC)

Benjamin Jacobs, Alessandro Vidoni, M Zulf Mughal

Royal National Orthopaedic Hospital, London, UK

### Abstract

**OBJECTIVES**: Denosumab has been shown to be effective in treating ABC but its use in the UK was restricted due to the risk of rebound hypercalcaemia*. We therefore used Zoledronate to stabilise calcium metabolism during Denosumab treatment.

**METHODS**: The diagnosis and treatment plan for each ABC patient was established in our Multidisciplinary Bone Tumour Team meetings. Patients with tumours in locations that were not amenable to surgery were offered Zoledronate (0.025 – 0.050 mg/kg six-monthly) and Denosumab (60mg monthly) combination treatment. Each child had regular dental assessments. Optimal vitamin D nutrition was ensured with blood tests before each drug dose. Response was monitored by symptoms (pain), signs (mobility) and tumour size and morphology on follow-up imaging (radiographs and MR).

**RESULTS**: We treated 13 patients (6 female, 7 male; age 7-17, median 13 years) with ABC tumours. Ten have now finished Denosumab (treatment duration range 1 year to 3 years) with follow-up after stopping Denosumab ranging from 1.1 to 3.3 years (median 1.65). Three of these children showed tumour growth after stopping Denosumab, but responded well to further treatment, one with surgical curettage, one with radio-ablation and one with sclerotherapy. Most are now asymptomatic and starting long-term follow-up. All had a reduction in tumour size, ossification on radiographs and reduction in fluid levels on MRI.

There were significant side-effects. The first two patients (who did not receive pre-treatment Zoledronate) both developed rebound hypercalcaemia requiring rescue bisphosphonate. Another two children developed rebound hypercalcaemia notwithstanding their Zoledronate pre-treatment, possibly due to long intervals between their follow-up Zoledronate infusions. One child developed a pathological wrist fracture. All patients developed prominent metaphyseal bands on follow-up radiographs and MRI. Many patients notice bone pains, especially round the knees and ankles. One teenage boy developed a transient, exceedingly high Creatine Kinase blood level (76,000 u/L).

**CONCLUSIONS**: Denosumab combined with Zoledronate was effective and tolerated with no discontinuation due to side-effects. It requires meticulous follow-up and monitoring. Further follow-up is required to determine optimal dosage and duration of treatment.

*** UK Medicines and Healthcare products Regulatory Agency (MHRA) Warning regarding Denosumab Published 17 May 2022** https://www.gov.uk/drug-safety-update/denosumab-60mg-prolia-should-not-be-used-in-patients-under-18-years-due-to-the-risk-of-serious-hypercalcaemia

### P81

### Continuous subcutaneous parathyroid hormone therapy guided by point of care calcium testing – first experience in Western Australia

Rachel Romans[1], Fiona McKenzie[2,3], Frances Gehrmann[1,4], Kiranjit Joshi[1,3,5], Matthew Moller[6], Dean Huppatz[6], Karen Winer[7], Aris Siafarikas[1,3,4,8,5]

[1]Department of Endocrinology and Diabetes, Perth Children's Hospital, Perth, Western Australia, Australia. [2]Genetic Services of Western Australia, King Edward Memorial Hospital, Perth, Western Australia, Australia. [3]Medical School, University of Western Australia, Perth, Western Australia, Australia. [4]Telethon Kids Institute for Child Health Research, University of Western Australia, Perth, Western Australia, Australia. [5]Western Australian Bone Research Collaboration, Perth, Western Australia, Australia. [6]Department of Pharmacy, Perth Children's Hospital, Perth, Western Australia, Australia. [7]Eunice Kennedy Shriver National Institutes of Child Health and Human Development, National Institutes of Health, Bethesda, Maryland, USA [8]Institute of Child Health Research, University of Notre Dame, Fremantle, Western Australia, Australia

### Abstract

**BACKGROUND AND PRESENTING PROBLEM:** A 9-year-old pre-pubertal girl with APECED (autoimmune polyendocrinopathy, candidiasis and ectodermal dystrophy) Syndrome due to a mutation in the AIRE (autoimmune regulator) gene was on conventional therapy with oral calcitriol and calcium for isolated hypoparathyroidism. She presented with recurrent symptomatic hypocalcaemic episodes requiring frequent hospital admissions for intravenous calcium therapy. Despite oral calcium supplementation and calcitriol at high doses, stable normocalcaemia could not be achieved and she developed nephrocalcinosis. In these scenarios subcutaneous (s/c) parathyroid hormone (PTH1-34) is a therapeutic alternative. Given the short half-life of PTH (1hr), continuous s/c infusion is regarded the most physiological and efficient regimen.

**CLINICAL MANAGEMENT:** We received local approval and informed consent to start continuous subcutaneous (s/c) PTH-infusion (Teriparatide, PTH 1-34), using an omnipod® pump (Insulet). In addition, we provided a point of care device for capillary testing of ionised calcium (iStat®, Abbot) to the family. They were instructed how to use the system and adjust PTH-infusion rates to ionised calcium levels following a "sliding scale" management plan with a 24/7 endocrine emergency service in place.

**DISCUSSION**: Looking at the first 18 months on subcutaneous pump therapy, the family quickly gained proficiency in the use of the pump system, calcium testing and management plan. Stable ionised calcium levels (0.8-1.0 mmol/L) were achieved on average PTH-doses lower than reported by other groups not using point of care testing (0.1-0.15 vs. 0.3-0.5 mcg/kg/day). There were no hospital admissions for intravenous calcium therapy. Adjustments were mainly needed for exercise and during high temperatures in summer; this was very suitable to the patient's lifestyle and climate conditions in Western Australia. Carers and patient reported improved quality of life and independence. Urinary calcium excretion normalised, there were no signs of progression of nephrocalcinosis, or renal insufficiency. Bone turnover markers P1NP and CTX increased only initially. Bone mineral content and density were stable on both pQCT and DXA.

We conclude that point of care testing of calcium levels optimises therapy with subcutaneous PTH infusion, and improves quality of life for both patient and carers.

### P82

### Evaluating the impact of growth hormone therapy on growth velocity and adult height in individuals with achondroplasia and hypochondroplasia

Hirofumi Nakayama[1,2], Yasuhisa Ohata[1], Makoto Fujiwara[1], Ikumi Ueda[1], Takeshi Ishimi[1], Chieko Yamada[1], Yukako Nakano[1], Tatsuya Nakamichi[3], Kenichi Yamamoto[1,4], Hiroyuki Saito[3], Taichi Kitaoka[3], Natsumi Yamamura[5], Toshimi Michigami[5,6], Katsusuke Yamamoto[5], Yasuji Kitabatake[1], Takuo Kubota[1], Keiichi Ozono[1,3]

[1]Department of Pediatrics, Osaka University Graduate School of Medicine, Suita, Japan. [2]The first Department of Oral and Maxillofacial Surgery, Osaka University Graduate School of Dentistry, Suita, Japan. [3]Iseikai International General Hospital, Osaka, Japan. [4]Division of Health Sciences, Osaka University Graduate School of Medicine, Suita, Japan. [5]Department of Pediatric Nephrology and Metabolism, Osaka Women's and Children's Hospital, Izumi, Japan. [6]Department of Bone and Mineral Research, Research Institute, Osaka Women's and Children's Hospital, Izumi, Japan

### Abstract

OBJECTIVES: Achondroplasia (ACH) and hypochondroplasia (HCH) are skeletal dysplasia characterized by marked short stature. Growth hormone (GH) is utilized for ACH and HCH patients in Japan. While previous reports have demonstrated the effects of GH, comprehensive analyses of its effects throughout the treatment process have been lacking.

METHODS: Participants diagnosed with ACH and HCH, who attended 2 bone dysplasia centers between 2011 and 2022 and received GH treatment for over 1 year, were included. Height standard deviation score (H-SDS) and annual growth velocity (AGV) were assessed before treatment initiation, at the outset, and annually up to 5 years post the initiation of GH treatment. H-SDS was determined based on data of individuals healthy and ACH or HCH, and both H-SDS and AGV were analyzed. For participants who reached their adult height (AH), it was calculated by adjusting for leg lengthening and compared with prior reports and the H-SDS at the initiation of GH.

RESULTS: The effects of GH administration for 1 to 5 years were investigated in 72 cases of ACH and 13 cases of HCH. Among ACH participants, H-SDS significantly increased from the first to the fifth year compared to the baseline. AGV significantly increased after the first year, with a decrease observed in the second year compared to the first. In HCH participants, H-SDS significantly increased at all points compared to baseline, while AGV increased significantly during the first and second years compared to the initiation of GH treatment. AH was assessed in 32 ACH and 4 HCH cases. For ACH participants, AH adjusted for leg-lengthening, was 126.5 and 132.9 cm for women and men, respectively, significantly higher than previously reported average values for women. The H-SDS of AH was also significantly higher than at the start of GH treatment. Among HCH participants, 3 out of 4 cases exceeded the previously reported data.

CONCLUSION: ACH participants experienced increased growth effects for 5 years following initiation of GH therapy, while HCH participants exhibited effects for 2 years. The H-SDS of AH among ACH participants increased compared to baseline, suggesting a GH-induced increase in AH.

**Disclosure:** BioMarin Pharmaceutical Inc. : Lecture fee

**P83**

### Navigating dose adjustments in Burosumab treatment for X-linked hypophosphatemia: The impact of reference range discrepancies

Manju Chandwani[1], Vrinda Saraff[2], James Law[1]

[1]Nottingham Children's Hospital, Nottingham, UK [2]Birmingham Children's Hospital, Birmingham, UK

### Abstract

X-linked hypophosphatemia (X-LH) is caused by a pathogenic variant in the PHEX gene, leading to elevated fibroblast growth factor 23 (FGF23) levels. Burosumab, a monoclonal antibody inhibiting FGF23 activity, was endorsed by National Institute for Health and Care Excellence (NICE) in October 2018. Burosumab doses are typically adjusted to achieve euphosphataemia.

A 9-year-old boy diagnosed with X- LH transitioned from conventional therapy to Burosumab (0.4mg/kg, 10mg) at the age of 4 years. After two months, the Burosumab dose was increased to 1 mg/kg/dose (20 mg) fortnightly following persistent low phosphate levels (1.01 mmol/L, Nottingham Children's Hospital, UK reference range: 1.45–1.78 mmol/L) and, subsequently, to 2 mg/kg/dose (40 mg) fortnightly within four months of starting Burosumab. The other biochemical markers (alkaline phosphatase and parathyroid hormone) stayed within a normal range and there was a radiological improvement in the severity of rickets. Nevertheless, even with dosage modifications, the serum phosphate levels consistently remained below the lower limit of the reference range.

By 8 years of age, the Burosumab dose had increased to 50 mg fortnightly (1.9 mg/kg/dose) with a serum phosphate level of 1.26 mmol/L and then 1.03 mmol/L (1.33–1.92). Therefore, paired samples were analysed at the local endocrine centre (Nottingham Children's Hospital, UK) and regional bone-specialist centre (Birmingham Children's Hospital, UK). Absolute results were similar but with markedly different reference ranges (Birmingham: 1.20 mmol/L [0.9–1.8]; Nottingham: 1.13 mmol/L [1.33–1.92]). Nottingham ranges are based on CALIPER (Canadian Laboratory Initiative on Paediatric Reference Intervals) data using the Abbott Alinity analyser. BCH uses Paediatric Pathology Harmony reference ranges (data from UK population) with Roche Cobas C501 analyser.

Although both labs use age-specific validated reference ranges, the difference in these between centres resulted in a significantly different clinical interpretation. Phosphate levels below the lower limit of normal led to a higher dose of medication being administered, against best practise prescribing principles, and increasing treatment cost. Given the cost of Burosumab (BNFC list price: £2992/10 mg vial), an additional 10mg/dose costs £77,792/year. Discussions with biochemical scientists highlighted the concern and an agreement was reached that the Paediatric Pathology Harmony reference ranges will be adopted as part of a local harmonisation project.

This case highlights the importance of comprehensive investigation and collaboration across specialities and centres in the event of a mismatch between the clinical and biochemical picture to achieve optimal patient outcomes.

**P84**

### The development of interstitial lung disease in a paediatric case whilst receiving burosumab treatment

David Eardley, Suzanne Armitage, Louise Apperley, Charlotte Jarvis, Sarah Mayell, Poonam Dharmaraj

Alder Hey Children's Hospital, Liverpool, UK

### Abstract

**Background:**

A 6 year old child, diagnosed with X-linked hypophosphatemic rickets (XLH) in India, was commenced on conventional therapy with suboptimal clinical and biochemical response. After moving to the UK aged 8 years, burosumab (anti-FGF23 monoclonal antibody) was commenced and increased to a maximum dose of 1.25mg/kg every 2 weeks. Symptoms, radiological rickets and biochemistry all subsequently improved.

There was a background history of atopy with significantly raised IgE, allergic rhinitis and eczema.

**Presenting Problem:**

After 2 years on burosumab therapy he was admitted with influenza B, adenovirus and rhinovirus infection, hypoxia and a 6 week history of dyspnoea and reduced exercise tolerance. Chest imaging showed bilateral ground glass opacification consistent with interstitial lung disease. Lung biopsy confirmed hypersensitivity pneumonitis. Throughout this period his bone chemistry remained within age specific reference ranges apart from insufficient vitamin D levels.

**Clinical Management:**

Despite extensive analysis of environmental exposure, no trigger for pneumonitis was identified. Burosumab was discontinued and conventional therapy recommenced. An adverse drug reaction was reported via the UK Medicines and Healthcare products Regulatory Agency (MHRA).

High dose intravenous steroids, hydroxychloroquine, azathioprine and azithromycin were commenced with good response clinically and radiologically.

Although his respiratory condition improved, bone health deteriorated during the 9 months on conventional therapy, with lower limb pains, poor growth and suboptimal biochemistry (low phosphate, high PTH and ALP levels). A multidisciplinary decision was made to restart burosumab with close monitoring. 6 months on he had improved symptomatically and biochemically without the respiratory symptoms returning, although he remains on immunosuppressant therapy.

**Discussion:**

Hypersensitivity pneumonitis is rare in children, with an incidence of 2 per million and prevalence of 4 per million [1]. No cases of pneumonitis linked to burosumab have been reported since licensing in 2018. Monoclonal antibodies used in rheumatological and cancer treatments have been implicated in hypersensitivity pneumonitis in adults [2, 3, 4]. Although burosumab was not proven to have contributed to the pneumonitis, it was prudent to withdraw the drug temporarily given the severity of the lung disease in a previously well child. Burosumab is a relatively new drug and any potential adverse effects should be reported.

**References**

| | |
|---|---|
| [1] | F. Buchvald, B. Petersen, K. Damgaard, R. Deterding, C. Langston, L. Fan, G. Deutsch, M. Dishop, L. Kristensen and K. Nielsen, " Frequency, treatment, and functional outcome in children with hypersensitivity pneumonitis.," *Pediatr Pulmonol. ,* vol. 46., no. 11, pp. 1098-107, 2011 . |
| [2] | D. Patel, S. Madani, S. Patel and L. Guglani, " Review of pulmonary adverse effects of infliximab therapy in Crohn's disease," *Expert Opin Drug Saf.,* vol. 15, no. 6, pp. 769-75, 2016. |
| [3] | C. Powell, S. Modi, H. Iwata, S. Takahashi, E. Smit, S. Siena, D.-Y. Chang, E. Macpherson, A. Qin, J. Singh, S. C. Taitt and D. Ross Camidge, "Pooled analysis of drug-related interstitial lung disease and/or pneumonitis in nine trastuzumab deruxtecan monotherapy studies," *ESMO Open. 2022,* vol. 7, no. 4, August 2022. |
| [4] | A. Tonelli, R. Lottenberg, R. Allan and P. Sriram, "Rituximab-induced hypersensitivity pneumonitis," *Respiration,* vol. 78, no. 2, pp. 225-9, 2009. |

### P85

### Three years of Burosumab treatment in a child with cutaneous skeletal hypophosphatemia syndrome: A case report and literature review

Manal Mustafa, M Zulf Mughal

Al Jalila Children's hospital, Dubai, UAE

### Abstract

Cutaneous skeletal hypophosphatemia syndrome (CSHS) is a rare mosaic disorder caused by somatic gain-of-function RAS mutations. It is characterized by segmental epidermal nevi and fibroblast growth factor-23 (FGF23) mediated hypophosphatemic rickets. These patients also have dysplastic cortical skeletal lesions.

We describe an Emirati child with CSHS whose hypophosphatemic rickets and dysplastic skeletal lesions failed to heal due to poor adherence to conventional oral phosphate supplements and alfacalcidol treatment. The diagnosis of CSHN with FGF23-mediated hypophosphataemic rickets was made in our patient due to increased urinary phosphate excretion and hypophosphatemia in the face of normal serum PTH levels and inappropriately elevated plasma FGF23 levels. The whole-exome sequence on cells from nevoid skin biopsy revealed a somatic missense variant c.182A>G p.(Gin61Arg) (chr11:533874;hg19) in the HRAS gene (OMIM *190020; chromosome 11p15.5).

Burosumab, a fully human immunoglobulin G1 monoclonal antibody to FGF23, has been approved for treating children with X-linked hypophosphatemia (XLH), a multisystem disorder caused by increased expression of FGF23. Treatment of our patient with Burosumab for 36 months resulted in normalization of her serum inorganic phosphate and alkaline phosphatase levels, healing of rickets, improvement in her linear growth, symptoms of myopathy, and quality of life.

In summary, we report the successful long-term treatment of hypophosphatemic rickets in CSHS with Burosumab over 36 months with a low dose of 0.2mg/kg. Burosumab showed promising efficacy and safety profile in our patient, without any side effects. This success may help in the approval of this targeted therapy for CSHS.

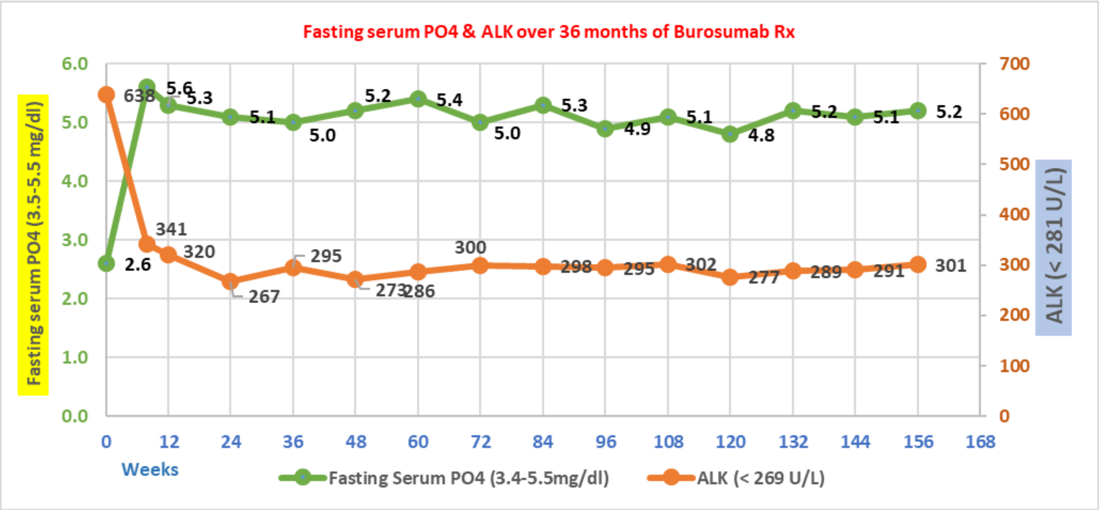

### P86

### Burosumab treatment for McCune Albright Syndrome - A case report

Sophia Sakka[1], Danai Georgakopoulou[1], Artemis Doulgeraki[2], Christina Kanaka-Gantenbein[1]

[1]Division of Endocrinology, Diabetes and Metabolism and Aghia Sophia Children's Hospital ENDO-ERN center for rare pediatric Endocrine disorders, First Department of Pediatrics, Medical School, National and Kapodistrian University of Athens, Aghia Sophia Children's Hospital, Athens, Greece. [2]Department of Bone and Mineral Metabolism, Institute of Child Health, Athens, Greece

### Abstract

### Background

Fibrous dysplasia/McCune-Albright syndrome (FD/MAS) is a rare condition caused by a mutation in the *GNAS* locus, resulting in activation of the G protein alpha subunit. Apart from several endocrinopathies, some cases are characterized by excessive FGF23 production from abnormal fibro-osseous tissue in FD lesions and results in increased renal phosphate excretion and impaired 1α-hydroxylase with subsequent hypophosphatemia and hyperparathyroidism.

### Presenting problem

We present a female paediatric patient who developed polyostotic fibrous dysplasia with various skeletal complications such as bone fractures, craniofacial deformity and scoliosis. She was also treated for hyperthyroidism and peripheral precocious puberty. Serum phosphate concentration was below, and alkaline phosphatase was above the reference range despite optimal conventional management with oral phosphate and alphacalcidol, which could not be tolerated because of gastrointestinal disturbances and hypercalciuria. She was also started on zoledronic acid infusions in preparation of hip surgery and had a normal bone density at the beginning of treatment with Burosumab.

### Clinical management

In an attempt to moderate the deleterious effects of uncontrolled hypophosphatemia on bone health we decided to treat her with burosumab off label at a dose of 0.66 mg/kg (20mg) every 2 weeks after one week off conventional treatment. Serum phosphate levels normalised within 2 weeks of treatment. Laboratory results showed improvement in serum alkaline phosphatase and PTH levels. After the second injection of burosumab, phosphate and PTH raised above the normal range with normal vitamin D levels, so we increased the interval between doses to 3 weeks to decrease the risk for ectopic mineralization and added calcium 500mg daily to lower PTH levels. However, phosphate levels dropped again below normal range, so she had to return to 2weekly injections of 20mg. There were no short-term adverse events associated with burosumab.

### Discussion

Burosumab is a monoclonal antibody against FGF23 which is able to suppress FGF23 expression and reduce the risk of developing FGF23-mediated hypophosphatemia and its associated complications. Burosumab should be considered as an effective and safe alternative strategy for FGF23-mediated hypophosphatemia in FD/MAS for those who either cannot tolerate or do not respond to the conventional therapy. To our knowledge this is the fourth case worldwide to have been successfully treated with burosumab.

| | Week 0 | Week 2 | Week 4 | Week 7 | Week 10 | Week 16 |
|---|---|---|---|---|---|---|
| | 1st injection of burosumab 20mg (0,66mg/kg) | 2nd injection of burosumab 20mg (0,66mg/kg) | 3rd injection of burosumab 20mg (0,66mg/kg) | 4th injection of burosumab 20mg (0,66mg/kg) | 5th injection of burosumab 20mg (0,66mg/kg) | 8th injection of burosubam 20mg (0,66mg/kg) |
| ALP | 736 | 614 | 487 | 496 | 442 | 425 |
| Calcium | 10,2 | 10,1 | 9,9 | 10,1 | 9,9 | 10,2 |
| Posphorus | 2,4 | 5,2 | 5,2 | 4,7 | 3,6 | 4,8 |
| Creatinine | 0,37 | 0,31 | 0,27 | 0,31 | 0,33 | 0,3 |
| Albumin | 4,9 | | | 4,6 | 4,5 | 4,6 |
| Sodium | 138 | | 139 | 140 | 140 | 140 |
| Potassium | 4,6 | | 4,7 | 4,8 | 4,6 | 4,8 |
| PTH | 63,5 | 99,3 | 78,2 | 97,6 | 21 | 75,8 |
| 25(OH)D | 25,6 | | | | 44,2 | 19 |
| 1,25(OH)D | 27,3 | | | | | |
| urine Ca | 11,4 | 10,3 | 7,4 | 4,5 | | 13,2 |
| Urine Ca/Cr | 0,13 | 0,1 | 0,07 | 0,04 | | 0,11 |
| urine P | 152,4 | | 178,3 | 124,8 | | 228 |

| | | | | | | |
|---|---|---|---|---|---|---|
| urine Cr | 85 | | 112 | 119 | | 116 |
| TmP/GFR | 2,39 | | 5,2 | | | 4,79 |
| bTRAP5b | 13,1 | | | | | |
| PICP | 135 | | | | | |
| Osteocalcin | 40 | | | | | |
| DEXA L1-L4<br><br>Z-score | 0,9 | | | | | |
| | Burosumab every 2 weeks | Burosumab every 2 weeks | Burosumab every 3 weeks<br><br>Calcium carbonate 500mg/day | Burosumab every 3 weeks | Burosumab every 2 weeeks<br><br>Vit D<br><br>2000iu/d | Burosumab every 2 weeks |

**Disclosure**

Dr Sakka has received honoraria for delivering presentations from Kyowa Kirin and Genesis. Dr Doulgeraki has received honoraria for delivering presentations from Genesis.

### P87

### Effect of growth hormone therapy on bone mineral density in children and young adults with Turner syndrome

Shruti Mondkar[1], Chirantap Oza[1], Keyur Desai[2], Neha Kajale[1,3], Vivek Patwardhan[1], Sharvani Patil[1], Chidvilas More[1], Vaman Khadilkar[1,3], Anuradha Khadilkar[1,3]

[1]Hirabai Cowasji Jehangir Medical Research Institute, Jehangir Hospital, Pune, India. [2]Adhikari Lifeline Hospital, Boisar, India. [3]School of Health Sciences Savitribai Phule Pune University, Pune, India

### Abstract

**Objectives:**

To study growth, body composition and bone density outcomes in girls with Turner syndrome stratified by therapeutic intervention [Growth hormone (GH), Estrogen (E2)]

**Methods**:

Cross-sectional, observational study. Karyotype-proven girls with Turner syndrome aged 4-25 years were included and those with uncontrolled hypothyroidism, untreated chronic disease were excluded. Anthropometry, sexual maturity rating (SMR), body composition, DXA parameters were assessed. $P<0.05$ was considered significant.

**Results:**

70 participants (Received GH:33, GH naïve:37) aged 13.8±5.1 years were enrolled. 24 (34.3%) had attained menarche prior to DXA assessment; 38 (54.3%) were on E2 replacement; 13 (18.6%) were hypothyroid but well-controlled on medication. None of the patients gave history of fractures. Mean height, weight, BMI Z-scores were -2.7,-1.4,-0.1 respectively. Mean age at GH initiation:9.5±2.6 years; E2 initiation:13.5±1.3 years; menarche:15.1±1.5 years. Mean serum FSH:66.1±56.4 mIU/mL. Those who received GH (group-B) were significantly taller than their GH-naïve counterparts (group-A) (Height Z-scores: -2.0±0.7 vs -3.4±0.8). Height and SMR-adjusted lean body mass (LBM=21.6±6.2%) and Z-scores (-3.5±0.05) were significantly higher among group-B versus group-A (17.8±6.7, -3.9±0.05). Height and SMR-adjusted TBLH aBMD Z-scores (-2.3±0.2;-3.1±0.2), lumbar spine aBMD Z-scores (-1.4±0.2;-2.6±0.2), LSBMAD Z-scores (-0.8±0.3;-1.3±0.2) were significantly higher among those who received GH. Following age and height-adjustment, FSH concentrations showed significant negative correlation with L1-L4 aBMD Z-score (r=-0.4), TBLH aBMD Z-score (r=-0.5). Duration of GH therapy correlated positively with height-adjusted TBLH aBMD Z-scores (r=0.4). LBM Z-score (B=2.70,p<0.05) and duration of E2 therapy (B=0.579,p<0.05) were predictors of lumbar spine aBMD Z-scores, while height (B=0.083,p<0.05), LBM Z-scores (B=2.746,p<0.05) and FSH concentrations (B=-0.07,p<0.05) were predictors of TBLH aBMD Z-scores (Table).

**Conclusions:**

Height, lean body mass and bone mineral density at TBLH and lumbar spine were significantly benefitted by growth hormone therapy. Apart from lean body mass, lumbar spine aBMD was significantly predicted by duration of Estrogen therapy and TBLH aBMD by height and FSH concentration.

### P88

### Vosoritide therapy in children with achondroplasia: Early experience in an Italian cohort

Anna Elsa Maria Allegri[1], Caterina Tedesco[1], Natascia Di Iorgi[1,2], Flavia Napoli[1], Daniela Fava[1], Alessia Angelelli[1], Emilio Casalini[1], Angela Pistorio[3], Mohamad Maghnie[1,2]

[1]Pediatric Clinic - Clinical Service in Pediatric Endocrinology, Diabetes and Metabolism IRCCS Giannina Gaslini, Genoa, Italy. [2]Department of Neuroscience, Rehabilitation, Ophtalmology, Genetics, Maternal and Child Health - DINOGMI, University of Genova, Genoa, Italy. [3]Scientific Direction, Epidemiology and Biostatistics Unit, IRCCS Giannina Gaslini, Genoa, Italy

### Abstract

### Objectives

Vosoritide is a recombinant C-type natriuretic peptide analogue that increases growth velocity in children with achondroplasia (ACH) and is administered at a subcutaneous dose of 15.0 µg/kg once-daily. In Italy vosoritide was approved in September 2022 in ACH patients aged ≥ 5 years; in July 2023 in ACH patients aged ≥ 2 years. We aimed to describe our experience in a cohort treated with vosorotide for at least 6 months.

### Methods

From December 2022 to February 2024, 38 patients started therapy (T0) at the Pediatric Endocrinology Unit, IRCCS Giannina Gaslini, Genoa, Italy. Inclusion criteria were: genetic diagnosis of ACH, open epiphysis (hand x-ray), age (≥ 2 and < 14 years).

A cardiac evaluation, an educational nurse therapeutic training were performed; a quality of life (QoL) evaluation was proposed.

At T0, at 3, 6 and 12 months, height standard deviation score (SDS), sitting height SDS, head circumference SDS, weight SDS, upper and lower segment ratio (SH/LS), pubertal Tanner stage (TS, I-V), adverse effects (AEs) were assessed.

3 patients were not included due difficulty in obtaining accurate anthropometric measurements during simultaneous lower limb lengthening surgery; 5 due to short follow-up (FU) (<6 months).

### Results

30 patients with >6 months of FU (males: 14, 46.7%, females: 16, 53.3 %) were enrolled. At baseline the median age was of 8.9 years (1st-3rd quartiles: 7.1-10.1 years), 26 patients (86.7%) were in TS I-I/II, 4 (13.3%) in TS II-II/III, weight SDS median value was -0.11 (1st-3rd quartiles -1.40; 0.76), and height SDS was -0.43 (-1.17; 0.31). Other parameters are reported in table 1.

**Table 1.** Pubertal stage and anthropometric parameters at baseline and at last Follow-up in the study cohort [N=30].

| | $T_{Last FU}$ | | | |
|---|---|---|---|---|
| | **Pubertal stage: I-I/II** | **Pubertal stage: II-II/III** | **Pubertal stage: III/IV** | **Total** |
| **Pubertal stage: I-I/II** | 23 (88.5 %) | 3 (11.5 %) | - | 26 |
| **II-II/III** | - | 2 (50.0 %) | 2 (50.0 %) | 4 |
| **Total** | **23** | **5** | **2** | **30** |
| P: 0.001 | | | | |

| | $T_0$ | $T_{Last FU}$ | P |
|---|---|---|---|
| Weight-SDS | -0.11 (-1.40; 0.76) | 0.03 (-1.10; 1.29) | 0.013§§ |
| Height-SDS | -0.43 (-1.17; 0.31) | -0.17 (-1.00; 0.50) | 0.0006§§ |
| Head Circumference-SDS | -0.48 (-0.90; 0.20) | -0.48 (-1.10; 0.40) | 0.35§§ |
| Sitting height/Height | 0.67 (0.65; 0.67) | 0.66 (0.65; 0.67) | 0.67§§ |
| Upper/ lower segment ratio | 1.99 (1.88; 2.06) | 1.97 (1.89; 2.06) | 0.45§§ |

§§ P: Wilcoxon test for paired data

AEs were limited to transient local reactions at injection site without any treatment discontinuation. No cardiac abnormalities were identified. Complete data concerning therapeutic training and QoL are ongoing to short FU.

**Conclusion**

We confirm a significant increase in height, while the SH/LS ratio between upper and lower segment remains stable after 6 months of treatment with vosorotide. Weight SDS shows a significant increase and AEs were limited to injection site reactions. Longer follow-up is needed to understand the impact of vosorotide on quality of life and cardiometabolic effects.

### P89

### Use of burosumab in the treatment of hypophosphatemic osteomalacia in neurofibromatosis type 1: A rare case with a novel treatment

Andrew Calabria

The Children's Hospital of Philadelphia, Philadelphia, USA

### Abstract

### Background:

Neurofibromatasis type 1 (NF1) is a multisystem genetic disorder characterized by dermatologic, neurologic, and oncologic manifestations and can be accompanied by skeletal manifestations, including scoliosis, osteoporosis, and less commonly, hypophosphatemic osteomalacia (HO). The cause of hypophosphatemia has been more commonly thought to be related to vitamin D deficiency and secondary hyperparathyroidism but rarely has been associated with increased levels of fibroblast growth factor 23 (FGF23).

### Presenting Problem:

Here presented is a case of 19 year old female with NF1 and history of medulloblastoma, Moyamoya, optic glioma, and osteoporosis with history of multiple fractures from 2019 to 2021, including treatment with zoledronic acid (ZA). Patient was found to have persistent hypophosphatemia (<2.5 mg/dl) unrelated to ZA, with elevated alkaline phosphatase of 147 U/L (42-106 U/L), low tubular reabsorption of phosphate (63%, inappropriately low 1,25(OH)2D (38.3 pg/ml; 24.8-81.5 pg/ml), and elevated FGF23 (225 RU/ml; 44-215 RU/ml). Other serum values were normal, including 25(OH)D (54.6 ng/ml), calcium (9.5 mg/dl), and intact PTH (31 pg/ml; 15-65 pg/ml). Imaging, including Ga68-DOTATATE scan, did not identify causative tumor. Patient underwent a histomorphometric bone biopsy of iliac crest, which confirmed severe osteomalacia (see figure).

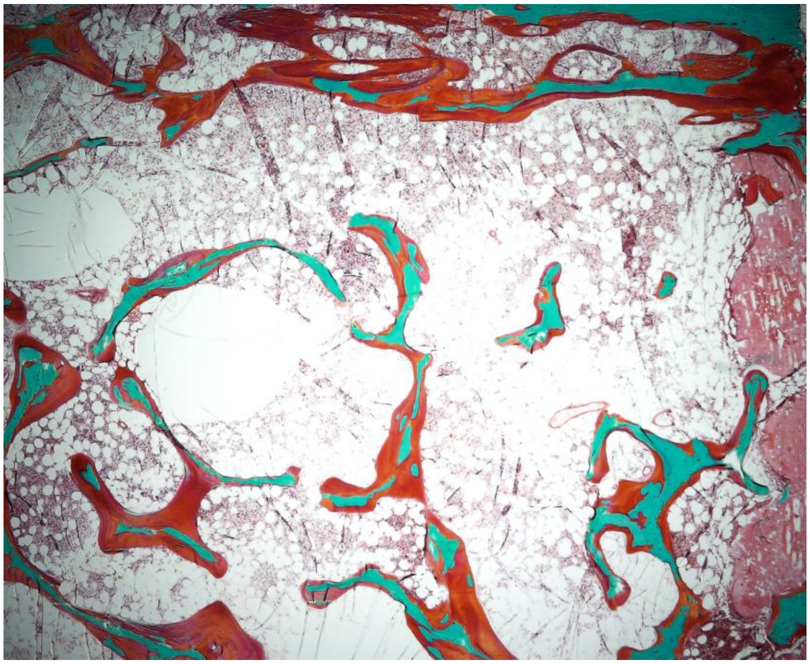

**Clinical Management:**

Patient was started on conventional therapy with calcitriol and phosphorus with persistent hypophosphatemia. Patient was transitioned to burosumab (0.5 mg/kg/dose q4 weeks) under tumor induced osteomalacia indication with normalization of serum phosphorus within 6 weeks.

**Discussion:**

HO is a rare skeletal association with NF1 and can be associated with increased FGF23 levels. While conventional treatment with calcitriol and phosphate has been previously used in such cases, this case shows that burosumab can be a potential treatment option in FGF23 mediated HO.

**P90**

### Natural history of leg deformities after treatment with burosumab

Rui Santos[1], Alessandra Cocca[1], Robyn Gilbey-Cross[1], Sophia Sakka[2,1], Jessica Sandy[3,1], Moira Cheung[4,1]

[1]Evelina London Children's Hospital, London, UK [2]Mitera Children's Hospital, Athens, Greece. [3]The Children's Hospital at Westmead, Westmead, Australia. [4]Great Ormond Street Hospital, London, UK

### Abstract

**Background:**

Leg deformities in children with X-linked hypophosphatemic rickets (XLH) are well described and orthopaedic intervention is at times necessary. With the advent of use of burosumab, a monoclonal antibody against FGF23, resolution of deformities appears to improve more quickly just with medical management.

**Aim and method:**

**Aim:** To characterise the natural history of leg deformities in children with XLH after 2 years of burosumab treatment.

**Method:** All children with XLH and had leg deformities who were about to commence burosumab from a single tertiary centre were identified. The rickets severity score and mechanical axis were evaluated at baseline, 6, 12 and 24 months.

**Results:**

16 of 21 children had leg deformities at baseline. The mean RSS was 1.65 (0 to 5.5) at start of treatment with 13 children (81%) having varus deformity in one or both legs, 2 (12.5%) with valgus deformity in one leg, and 1 (6%) with varus deformity in one leg and valgus in the other.

At 12 months the mean RSS was 0.06 (0 to 0.5), 15 children (94%) had improvement of the mechanical axis, 4 (27%) with normal mechanical axis in both legs, and 5 (33%) in one leg.

At 24 months the mean RSS was 0.03 (0 to 0 0.5), 15 (94%) children had improved mechanical axis from baseline, 11 (69%) normal mechanical axis in both legs, 2 (13%) normal mechanical axis in one leg and almost normal axis in the other, 1 (6%) normal mechanical axis in one leg and varus deformity in the other, and 1 (6%) child with worse varus deformity on both legs.

5 patients had some form of orthopaedic correction, with a variety of procedures (8-plates, derotation osteotomies), 3 had normal mechanical axis at the start of treatment which did not change, 1 had deformity in one limb which corrected after treatment and another normalized after treatment.

**Conclusions**

Most children on treatment with burosumab have progressive improvement of their limb deformity as early as 12 months after treatment. This is sustained at 24 months with 69% having normal mechanical axis in both legs.

| Mechanical axis of the lower limb (Right/Left) - normal from 3 to 17mm | | | | | |
|---|---|---|---|---|---|
| Ortho int (Y/N) | 0m | 6m | 12m | 24m | Deformity |
| N | 25/27 | 23/23 | 20/21 | 12/4 | Varus |
| N | 29/35 | 25/31 | 13/18 | 0/10 | Varus |
| N | 10/16 | 4/5 | 1/4 | 0/0 | Normal |
| Y | 19/11 | 0/0 | 0/0 | 0/0 | Valgus |
| Y | 10/54 | 6/49 | 14/53 | 16/22 | Valgus |
| N | 47/50 | 30/27 | 20/20 | 12/0 | Varus |
| N | 28/27 | 24/32 | 13/23 | 7/13 | Varus |
| N | 11/35 | 7/28 | 9/24 | 0/0 | Valgus/varus |
| N | 35/44 | NA | 28/30 | 41/44 | Varus |
| N | 3/27 | No x-rays | 0/27 (18 months) | 0/21 (over 24m) | Varus |
| N | 26/34 | 10/15 | 0/0 | 0/0 | Varus |
| N | 28/36 | 21/21 | 17/21 | 6/11.5 | Varus |
| Y | 0/35 | 11/30 | 19/8 | 6/0 | Valgus |
| Y | 0/7 | No x-rays | No x-rays | 0/0 | Normal |
| N | 46/39 | 36/29 | 29.6/21.8 | 27/13 | Varus |
| N | 8/17 | 7/16 | 9/17 | 18/22 | normal to valgus |
| N | 8/14 | 6/17 | 13/15 | NA | Normal |
| N | 0/0 | 0/0 | 0/0 | Normal | Normal |
| Y | 62/37 | 40/16 | Not performed | Normal | Varus |
| N | 85/75 | 88/76 | 79/70 | 71/66 | Varus |
| N | 15/22.5 | Not performed | 3/17 | Not performed | Left Varus deformity |

### Disclosure

Funding from Kyowa Kirin have been received from authors for travel grants (all), Research grant (JS), delivering lectures (MC, SS, RS)

### P91

### Design and objectives of the Acorn Study: A non-interventional study evaluating long-term safety in achondroplasia children treated with Vosoritide

Jeanne M Pimenta[1], Fiona Fettes[1], Swati Mukherjee[1], Sheetal Ingole[2], Veronika Horvathova[2], Ekkehart Lausch[3]

[1]BioMarin Pharmaceutical Inc., London, UK [2]BioMarin (UK) Limited, London, UK [3]University Hospital Freiburg, Freiburg, Germany

### Abstract

**OBJECTIVES**: Achondroplasia is a rare skeletal dysplasia leading to impaired endochondral bone growth and multiple medical complications. Vosoritide (C-type natriuretic peptide analog) is indicated for treatment of genetically confirmed achondroplasia (≥4 months until epiphyseal closure). We describe the first treatment-based registry, created to monitor long-term safety of vosoritide in real-world use.

**METHODS:** Acorn is a multicenter, non-interventional, post-authorisation, Category 3 safety study requested by the Europe Medicines Agency. The study aims to recruit approximately 380 participants into 2 cohorts: (1) incident users aged ≥ 4 months to ≤ 8 years old, defined as those who either recently started, or plan to start treatment with vosoritide, and (2) prevalent users who initiated treatment as part of the French expanded access program or vosoritide open-label clinical trials. The primary objective is to evaluate long-term safety including bone-related adverse events (eg. joint and spinal cord disorders, fractures and slipped capital femoral epiphyses) and immunogenicity. Secondary objectives include evaluating the long-term impact of treatment on disease-related outcomes, including achondroplasia-related complications and surgeries and changes in anthropometric measures. The study period is 10 years from the date of first patient enrolled. In addition, participants who complete (reach final adult height) or discontinue treatment during the study, will be followed up 2 years later. Almost all vosoritide treated children aged ≥ 4 months to ≤ 8 years will be eligible for the study, leading to a more representative population of participants than in clinical trials. Data collected will reflect standard clinical practice and real-life management.

**RESULTS:** The EMA-approved protocol is registered on the EU post-authorization study register (EUPAS47514). In total, 8-10 countries (~30 sites) are involved. Recruitment started in April 2023; 25 participants from 9 sites are enrolled (16 in cohort 1 and 9 in cohort 2) and data will be shared on the number, location and demographics of participants.

**CONCLUSIONS:** Vosoritide is the first approved medicinal treatment for children with achondroplasia. Acorn will collect important long-term, real-world data across Europe, and will provide important insights into the impact of long-term treatment on safety, effectiveness and the use of vosoritide in context of other interventions.

### Disclosure

JMP, FF, SW, SI, VH are employees of BioMarin. EL has participated in advisory board meetings for BioMarin.

### P92

### Metacarpal bone health parameters in boys treated with Anastrozole

Reham Ghanim, Nandu Thalange, Sarah Ehtisham, M Zulf Mughal

Al Jalila Children's Hospital, Dubai, UAE

### Abstract

**Introduction:** Aromatase Inhibitors such as Anastrozole are utilized to enhance final adult height in peripubertal boys by impeding the conversion of androgens to estrogens, a critical process in growth plate closure. However, concerns have been raised about the potential adverse effect of aromatase inhibitors on bone health, as estrogen plays a critical role in maintaining bone density and strength. The impact of Anastrozole on bone health in peripubertal boys has not been extensively investigated.

**Objectives:** To study the effect of Anastrozole on metacarpal bone health parameters [metacarpal index (MCI) and the bone health index (BHI] using the standalone BoneXpert v3.2.2, in boys before and 2 years after anastrozole treatment.

**Methodology:** This was a retrospective analysis of 90 hand radiographs from 30 adolescent boys with idiopathic short stature who received Anastrozole over two years. Patients with chronic medical conditions or those treated with growth hormone were excluded. The Bone age (BA), MCI and BHI were analyzed using automated bone age radiograph analysis by BoneXpert software.

**Results:** The mean (SD) age of starting Anastrozole in our cohort was 13.5 (SD 1.69) years. The results of changes in standard deviation (SDS) of the BA, MCI and BHI after 2 years of treatment with Anastrozole are shown in Table 1.

**Conclusion:**

We conclude that Anastrozole treatment did not have adverse effects on metacarpal bone health parameters. In fact, we observed a significant improvement in the metacarpal bone index and the bone health index standard deviation scores following treatment with Anastrazole. However, further studies, using peripheral quantitative tomography would be helping in validating these findings.

**Table 1:**

| Years after starting Anastrozole treatment | 0<br><br>Mean ± SD | 2<br><br>Mean ± SD | *P* value assessed by the paired T test |
|---|---|---|---|
| BA SDS | 0.11 (± 1.6) | 0.73 (±1.4) | <0.001 |
| MCI SDS | -0.97 (± 1.19) | -0.42 (±1.23) | <0.001 |
| BHI SDS | -0.97 (± 1.15) | -0.71 (±1.37) | <0.001 |

### P93

### Time to rebound hypercalcaemia associated with cumulative weight-based denosumab dose for central giant cell granuloma treatment in children despite dose weaning regimen

Alexander Chesover[1,2], Jeremy Allgrove[1], Cathy Campbell[3], Dearbhla McKenna[3], Madeleine Rooney[3], Caroline Mills[1], Nadeem Saeed[1]

[1]Great Ormond Street Hospital for Children, London, UK [2]Royal National Orthopaedic Hospital, London, UK [3]Musgrave Park Hospital, Belfast, UK

### Abstract

**BACKGROUND.** Central giant cell granulomas (CGCG) of the mandible and maxilla are locally destructive, non-neoplastic, intraosseous lesions. CGCG contain multinuclear osteoclast-like giant cells that express receptor activator of nuclear factor-κB (RANK). Denosumab, a monoclonal antibody against RANK ligand, is licensed in skeletally mature patients with giant cell bone tumours. There is less published experience in children who are at increased risk of rebound hypercalcaemia.

**PRESENTING PROBLEM.** We describe the response to denosumab of five skeletally immature children with CGCG, aged 2.1-11.6 years at treatment initiation, for lesions with high surgical risk or unresponsive to surgery. Consent for publication was provided for all patients.

**CLINICAL MANAGEMENT.** Patient 1: 1.7 mg/kg monthly for 17 months. Patient 2: 1.7 mg/kg monthly for 8 months. Patient 3: 120 mg monthly for 21 months. Patient 4: 60 mg every 2 months. Patient 5: 1 to 1.7 mg/kg monthly for 13 months and now on wean protocol. Denosumab wean protocol (dose frequency wean and scheduled Zoledronic acid) aimed to decrease rebound hypercalcaemia rate and completed in patients 1 and 3.

Lesion ossification and no further surgery, n=3; lesion ossification then excision of unilateral lesion, n=1; lesion mostly ossified, n=1 (and denosumab wean ongoing). No recurrence (median follow-up 11.5 months). All had rebound hypercalcaemia, 8.9-47 weeks after last treatment dose, two complicated by transient acute kidney injury, and all treated with bisphosphonates. Cumulative denosumab treatment dose/kg positively correlated with time to rebound after last treatment dose ($r=0.97$; $p=0.03$).

**DISCUSSION.** Denosumab successfully treated all CGCG lesions; however, was universally associated with rebound hypercalcaemia, not allayed by a weaning protocol used in two patients. The cumulative weight-based treatment dose strongly correlated with time to rebound hypercalcaemia (but not with total cumulative dose; data not shown). This suggests that weight-based dosing may affect denosumab's pharmacodynamic properties in children, in contrast to adult data. When denosumab is indicated, and based on this experience, we advocate for weight-based dosing in children to help predict time to rebound hypercalcaemia and direct proactive monitoring and treatment. Shared-cared arrangements with local paediatric teams is essential. The long-term consequences of denosumab in children remain uncertain.

### P94

### Long-term dose-titration experience with vamorolone in subjects with Duchenne muscular dystrophy (DMD) enrolled in Expanded Access Programs

Ana de Vera[1], Wido Tilmann[1], Aki Linden[1], Paula R Clemens[2], Michela Guglieri[3], Eric P Hoffman[4]

[1]Santhera Pharmaceuticals (Switzerland) Ltd, Pratteln, Switzerland. [2]Department of Neurology, University of Pittsburgh School of Medicine, Pittsburgh, USA [3]John Walton Muscular Dystrophy Research Centre, Newcastle University, Newcastle upon Tyne, UK [4]ReveraGen BioPharma, Rockville, USA

### Abstract

**Objectives:** The recommended dose of vamorolone in children with DMD is 6 mg/kg/day. Doses may be titrated down to as low as 2 mg/kg/day, based on tolerability. Here we report experience with vamorolone dose titration in Expanded Access Programs (EAPs).

**Methods:** Data were collated from subjects who had completed studies VBP15-LTE or VBP15-004 and enrolled in one of three EAPs in the USA, Canada and Israel. Available data were pooled to explore the frequency of dose titration during the EAPs. The EAPs allowed for up- or down-titration of vamorolone as medically warranted.

**Results:** At EAP baseline, the median age of subjects (N=69) was 7.1 years, median height was 117.5 cm and median weight was 25.8 kg. At the start of the EAPs, most subjects were dosed at vamorolone 4 mg/kg/day (44.9%; n=31) or 6 mg/kg/day (39.1%; n=27), with fewer dosed at 2 mg/kg/day (15.9%; n=11). The median duration of vamorolone exposure during the EAPs was 3.3 years with a maximum of 4.9 years. The majority of subjects did not change dose, and up- and down-titrations occurred at a similar frequency (Table). Up-titration from 4 to 6 mg/kg/day and down-titration from 6 to 4 mg/kg/day were the most common dose changes, and the least common changes were down-titrations from the higher doses to 2 mg/kg/day (Table). During the EAPs, 16 subjects discontinued treatment; most of these were due to withdrawal by either the subject or parent/guardian (n=14), one was due to physician decision and one was due to an adverse event. By the end of the EAP or treatment cut-off, most subjects were dosed at vamorolone 4 mg/kg/day (43.5%; n=30) or 6 mg/kg/day (37.7%; n=26) and fewer were dosed at 2 mg/kg/day (18.8%; n=13).

**Conclusions:** Long-term experience in the EAPs shows that vamorolone 4 and 6 mg/kg/day are the most frequently used doses and that most patients do not change dose. However, the therapeutic dose range from 2 to 6 mg/kg/day allows physicians to up- or down-titrate dosing based on individual tolerability.

**Table: Dose changes during the EAPs**

| Category | Vamorolone 2–6 mg/kg/day (N=69) n (%); f |
|---|---|
| Any dose changes | 29 (42.0); 41 |

| | |
|---|---|
| Up-titrations | 16 (23.2); 21 |
| Down-titrations | 18 (26.1); 20 |
| 4 mg/kg/day to 6 mg/kg/day | 13 (18.8); 15 |
| 6 mg/kg/day to 4 mg/kg/day | 11 (15.9); 12 |
| 2 mg/kg/day to 4 mg/kg/day | 4 (5.8); 6 |
| 6 mg/kg/day to 2 mg/kg/day | 4 (5.8); 4 |
| 4 mg/kg/day to 2 mg/kg/day | 4 (5.8); 4 |

Dose levels 3–5 mg/kg/day are included in 4 mg/kg/day.

Dose changes as part of tapering to discontinue vamorolone in two subjects were excluded.

EAP, Expanded Access Program; n, subject count; %, percentage of subjects from total subject count; f, number of events.

### Disclosure

Ana de Vera and Aki Linden: Employees of Santhera. Paula R Clemens: Grants: ReveraGen, NS Pharma, Amicus, Sanofi, Spark, NIH, FDA, Foundation to Eradicate Duchenne; Consultancy: Catalyst Medical Education, Med Learning Group. Michela Guglieri: Grants: Edgewise, Pfizer, Italfarmaco, Santhera, Roche, ReveraGen, Dynacure, Dyne, Sarepta, PTC Therapeutics; Consultancy: Pfizer, Dyne, NS Pharma, Antisense; Honoraria: Italfarmaco, Santhera, Roche, Sarepta, Novartis. Eric P Hoffman: Stock ownership: ReveraGen, AGADA BioSciences; Employee: ReveraGen, TRiNDS, AGADA BioSciences.

### P95

### Cardiovascular health in pediatric patients with X-linked hypophosphatemia (XLH) following two years of burosumab therapy

Avivit Brener[1,2], Gil Beer[1,2], Guy Baruch[1,2], Ehud Rothschild[1,2], Michal Yackobovitch-Gavan[2], Roxana Cleper[1,2], Leonid Zeitlin[1,2], Livia Kapusta[1,2,3]

[1]Dana-Dwek Children's Hospital, Tel Aviv, Israel. [2]Tel Aviv University, Tel Aviv, Israel. [3]Amalia Children's Hospital, Nijmegen, Netherlands

### Abstract

**Objectives:** An inactivating mutation in the phosphate-regulating endopeptidase X-linked (*PHEX*) gene leads to X-linked hypophosphatemia (XLH). A defective *PHEX* gene fails to curb the production of phosphatonin fibroblast growth factor 23 (FGF23), which has been identified as a potential contributor to damage to cardiac structure and function. We aimed to assess the prevalence and severity of cardiovascular morbidity in pediatric patients with XLH before, during and after 2 years of treatment with burosumab, the recombinant anti-FGF23 antibody.

**Methods:** This prospective observational study was conducted in a tertiary medical center. It included 13 individuals with XLH (10 girls and 3 boys, age range 0.6–16.2 years at baseline), who received burosumab every 2 weeks. Clinical assessment at treatment initiation and after .5, 1 and 2 years of uninterrupted treatment included anthropometric measurements and comprehensive cardiologic evaluation (blood pressure [BP], electrocardiogram, conventional echocardiography and myocardial strain imaging). Parameters were converted to sex- and age-adjusted z-scores.

**Results:** The linear growth of all patients improved significantly (mean height z-score: from -1.70 ± 0.80 to -0.96 ± 1.08, $p = 0.03$). Other favorable effects were the decline in rates of overweight/obesity (from 46.2% to 23.1%) and a decreased rate of elevated BP (systolic BP: from 38.5% to 15.4%; diastolic BP: from 38.5% to 23.1%). Electrocardiogram did not reveal any significant abnormality throughout the study period. The baseline echocardiography had revealed mild cardiac anomalies in 4 patients: mitral regurgitation (MR) in 2 and a patent foramen ovale (PFO) (n=2, at the age of 6 and 7 months). After 2 years of burosumab therapy, the PFOs resolved with no changes in the MRs. Cardiac dimensions and myocardial strain parameters were within the normative range for age at baseline and did not change significantly during the study period.

**Conclusion:** The comprehensive cardiologic evaluation provided reassurance that there were no notable cardiac health issues before starting burosumab treatment, and that this status remained unchanged after two years of therapy. A beneficial effect of this treatment was a reduction in cardiovascular risk factors, as evidenced by the lower prevalence of both overweight/obesity and elevated blood pressure.

### P96

### Care for infants with osteogenesis imperfecta in a neonatal setting

Cristina McGreal[1], Sarah Little[1], Andrea Schelhaas[1], Jeanne Franzone[1,2], Ricki Carroll[1,2]

[1]Nemours Children's Health, Wilmington, USA [2]Thomas Jefferson University, Philadelphia, USA

### Abstract

**Background:**

Osteogenesis imperfecta (OI) is a rare condition that occurs in 1 in every 16,000 to 20,000 births. The prevalence of each type of OI is unknown. It can be challenging to determine the severity and prognosis of the condition in utero, often limiting care and interventions offered to families.

**Presenting Problem:**

In the past 5 years we have cared for 13 infants with moderate to severe OI. 11 of these infants were diagnosed in-utero with type II or “lethal” OI. Of these 13 infants, 11 are still alive today and thriving. 8 of these infants were born in our advanced delivery unit, and all 13 of these infants were cared for in our neonatal intensive care unit in consultation with our multidisciplinary OI team.

**Clinical Management:**

In our experience, the severity of an infant’s OI cannot be determined until after birth. Therefore, for families who opt for life-sustaining treatments, our center offers a trial of interventions, irrespective of the genotype or perceived phenotype in utero. Our initial step is to meet with the family to offer education around the diagnosis of OI, review the infant’s images with the family, and discuss possible interventions and likely outcomes.

Following birth, our multidisciplinary team works together to provide the infant with immediate needs which can include respiratory support, nutrition optimization and pain management, with the hope of successfully transitioning to a focus on safe handling, fracture care, feeding, and initiating bisphosphonate therapy as soon as practical.

**Discussion:**

The best time to begin caring for the family of an infant diagnosed with OI is prenatally. A multidisciplinary team that offers the family education, elicits their goals for their infant, and creates a family-centered care plan is the first step in supporting a family who faces this new diagnosis. Our team-based approach to care for these infants, which starts before birth and continues through the delivery room, into the NICU, and beyond, is the foundation for the improved outcomes we are seeing for infants diagnosed with “lethal” OI in utero.

### P97

### Benefits of low-dose Denosumab for managing treatment-resistant rebound hypercalcaemia after cessation of Denosumab therapy for giant cell granulomas

Karissa Ludwig[1,2,3], Madeleine Hall[1], Andrew Cotterill[1], Wayne Nicholls[4,3], Geoff Donald[5], Tony Huynh[1,3,6], Craig Munns[1,3]

[1]Department of Endocrinology and Diabetes, Queensland Children's Hospital, Brisbane, Australia. [2]Department of Paediatrics, West Moreton Health, Ipswich, Australia. [3]Faculty of Medicine, University of Queensland, Brisbane, Australia. [4]Department of Oncology, Queensland Children's Hospital, Brisbane, Australia. [5]Department of Orthopaedics, Queensland Children's Hospital, Brisbane, Australia. [6]Department of Chemical Pathology, Mater Pathology, Brisbane, Australia

### Abstract

### Background

Denosumab is a monoclonal antibody against RANK-ligand, which is essential for osteoclast differentiation and activation. Noonan syndrome is a RAS-opathy with a propensity for giant cell granulomas (GCGs) which express RANK and respond well to treatment with denosumab. Adverse effects in children include metaphyseal sclerosis and post-cessation hypercalcaemia (1).

### Presenting problem

A 17-year-old male with Noonan Syndrome was diagnosed with multiple GCGs of the maxilla and mandible at 6 years old. After repeated surgical debulking, denosumab was commenced at 12 years old: 120mg weekly for 3 weeks, monthly for 18 months, 3rd monthly for 6 months, then 4th monthly. Positron emission tomography (PET)-computed tomography (CT) and magnetic resonance imaging (MRI) scans performed at 1 and 3 years demonstrated complete metabolic response, with reduction in size and sclerosis.

At 16 years old, after 4 years of denosumab therapy, he presented with a right distal femoral fracture, at the junction of a thick band of metaphyseal sclerosis and 'normal' bone. The fracture required surgical fixation, and calcium levels monitored weekly. He presented 3 weeks later with severe hypercalcaemia 3.08 mmol/L (2.20-2.65).

### Clinical Management

Initial management with IV hyperhydration followed by 0.025 mg/kg Zoledronate resulted in normalisation of calcium. However, one week later his rebound hypercalcaemia returned (4.20 mmol/L), and did not resolve with hyperhydration, calcitonin, risedronate, and 5 doses of Zoledronate (total 0.15mg/kg) over a 2-week period. Denosumab (30mg; 0.85mg/kg) was subsequently given resulting in rapid normalisation of serum calcium.

He now requires low doses of denosumab (10mg; 0.25mg/kg) every 6-7 weeks when hypercalcaemia recurs. Metaphyseal sclerosis at the proximal tibia has improved on plain x-ray. After 15 months on this low-dose regimen, PET-CT showed no evidence of GCG activation.

**Discussion**

Denosumab therapy significantly reduced morbidity from aggressive facial GCGs, but also resulted in a pathologic fracture and treatment-resistant hypercalcaemia in this patient. 15 months of low-dose denosumab has successfully managed rebound hypercalcaemia, whilst allowing remodelling of the metaphyseal sclerosis and supressing GCT activity on PET-CT. This case highlights the importance of exploring the utility of long-term low-dose denosumab therapy in patients with GCG, especially in the setting of a predisposing RAS-opathy.

**References**

1. Vanderniet JA, Wall CL, Mullins A, London K, Lim L, Hibbert S, et al. Denosumab for central giant cell granuloma in an Australian tertiary paediatric centre. Bone. 2022;159:116395.

### P98

### Rhabdomyolysis following zoledronic acid infusion in a patient with Duchenne muscular dystrophy

Rebecca J Moon[1,2], Victoria Ivison[3], Nabil Boulos[1], Eleanor J Marshall[3], Louise E Milne[3], Justin Davies[1,4]

[1]Paediatric Endocrinology, University Hospital Southampton NHS Foundation Trust, Southampton, UK [2]MRC Lifecourse Epidemiology Centre, University of Southampton, Southampton, UK [3]Paediatrics, Portsmouth University Hospital NHS Foundation Trust, Portsmouth, UK [4]Faculty of Medicine, University of Southampton, Southampton, UK

### Abstract

**BACKGROUND:** Patients commencing bisphosphonate therapy should be counselled on the benefits and risks. We report a case of rhabdomyolysis following zoledronate, highlighting the need to advise patients/parents of this potential complication.

**PRESENTING PROBLEM:** An 11-year-old boy with Duchenne muscular dystrophy (DMD) and steroid-induced osteoporosis was commenced on intravenous zoledronic acid. He was advised to drink 2-3 litres the day before the first infusion to ensure adequate hydration.

Blood sampling on the day of the infusion showed a urea of 3.1 mmol/l (2.5-6.5), creatinine 28 mmol/l (35-95), normal serum electrolytes, including calcium and phosphate, and 25-hydroxyvitamin D (89nmol/l). He was administered 0.0125 mg/kg zoledronic acid intravenously over 45 minutes, which was well tolerated. He was discharged the same day with calcium supplementation and advised to have stress-dose hydrocortisone for 72 hours.

The next day, his parent contacted the paediatric endocrinology team concerned about new onset very dark urine. Fever and myalgia were also reported. Urgent review was advised. On admission he had a markedly elevated serum creatine kinase (CK) of 95111 U/L (normal range 4-320 U/L) with 3+ proteinuria and 4+ blood on urine dipstick. Retrospective CK analysis on the baseline blood sample was 1576 U/L, confirming acute rhabdomyolysis (as opposed to an elevated CK due to DMD). There was no acute kidney injury (AKI) (creatinine 13 mmol/l, urea 1.6 mmol/l).

**CLINICAL MANAGEMENT:** He was managed with intravenous hyperhydration. CK normalised to baseline over nine days with no AKI. Further bisphosphonate has not yet been given.

**DISCUSSION:** Rhabdomyolysis is a rare complication of bisphosphonate therapy. Four cases have previously been reported in the literature, all in boys with DMD. This was the lowest zoledronate dose reported to cause rhabdomyolysis. Prudent observation and appropriate questioning of the cause for dark urine by the parents of this case enabled prompt diagnosis and supportive management without an AKI. Patients given zoledronate should be advised of this complication and to seek urgent review if dark urine is seen. Annual monitoring of CK in patients with DMD on bisphosphonates may be helpful to differentiate DMD-related CK rise from acute rhabdomyolysis.

### P99

### Phalangeal microgeodic syndrome treated with Nifedipine

Binod Pandey, Benjamin Jacobs, Alessandro Vidoni, Paul O'Donnell

Royal National Orthopaedic Hospital, London, UK

### Abstract

### Background:

Phalangeal Microgeodic Syndrome (PMS) is a bone disease primarily affecting children's fingers and toes. It was first described by Maroteaux in 1970 when diagnosis depended on radiographic findings. More recently MRI changes have become recognised. The aetiology of PMS is still not fully understood, but it occurs mainly in winter and exposure to cold is part of the pathogenesis. There have been 3 case reports of PMS treatment with Nifedipine, which is a standard treatment for a related condition Raynaud's phenomenon

### Presenting Problem:

Four patients (1 male, 3 female) presented between 5 and 14 years of age with pain and swelling in the toes and/or fingers. Clinical signs consisted of swelling and skin discoloration.

Radiographs of hands/feet were either normal or showed distinctive features such as phalangeal lucencies. MRI findings included soft tissue and bone oedema, lytic lesions, and periosteal reaction.

### Clinical Management:

Standard treatment of PMS is by avoiding cold exposure. Three of our cases had Vitamin D deficiency, which we treated with long-term supplementation. One child had particularly severe PMS of her toes limiting her ability to walk and not healing despite avoiding cold exposure. Nifedipine treatment was therefore tried. The dose was gradually increased up to two 20mg modified release tablets in the morning and one in the evening. Her symptoms improved and her toes healed.

### Discussion:

We describe the clinical and radiological findings of 4 children with PMS and the response one child showed to treatment with oral Nifedipine.

### P100

### Breast milk-associated hypercalcaemia – a retrospective cohort study to describe clinical and radiological outcomes

Sandhya Govindarajan[1], Raja Padidela[1,2], M Zulf Mughal[1,3], Imran Zamir[4], Amish Chinoy[1,2]

[1]Royal Manchester Children's Hospital, Manchester, UK [2]University of Manchester, Manchester, UK [3]Al Jalila Children's Specialty Hospital, Dubai, UAE. [4]North Manchester General Hospital, Manchester, UK

### Abstract

**Objectives**: Asymptomatic hypercalcaemia in infants who are exclusively breast-fed is recognised in clinical practice but has never been formally evaluated or outcomes described if breast feeding is continued. We conducted a novel retrospective analysis to evaluate the biochemical and radiological outcomes of breast milk-associated hypercalcaemia.

**Methods**: A multi-site retrospective study was conducted across a 4-year period in infants noted to be hypercalcaemic (corrected Calcium (cCa) ≥2.8 mmol/L) who were exclusively breast-fed and did not have other medical conditions causing hypercalcaemia. Clinical, biochemical and radiological parameters were collected and analysed.

**Results**: 18 infants (10 males; 5 preterm) were included in the analysis. Mean peak cCa was 3.1 mmol/L (reference range 2.2-2.8; standard deviation [SD] 0.2). All infants were exclusively breast-fed at detection of hypercalcaemia and all were clinically asymptomatic. None of them had interventions such as locasol, intravenous fluids, diuretics or bisphosphonates.

Mean corresponding PTH was low (0.9 pmol/L; reference range 2.0-9.4; SD 0.8). Mean corresponding 25-hydroxyvitamin D (60.8 nmol/L; reference >50; SD 37.9) and phosphate (1.7 mmol/L; reference range 1.3-2.6; SD 0.4) were normal. There was no significant correlation of peak cCa with corresponding PTH, phosphate and 25-hydroxyvitamin D.

All 18 infants had renal ultrasound imaging - none of them had evidence of nephrocalcinosis. Mean duration of resolution of hypercalcaemia was 123 days (range 10-329). Mean duration of exclusive breast feeding was 107 days (range 8-180). There was no significant correlation between age of resolution of hypercalcaemia and duration of breast feeding.

**Conclusion**: Breast milk-associated hypercalcaemia is characterised by PTH-independent hypercalcaemia. Unlike other causes of PTH-independent neonatal hypercalcaemia, it is an asymptomatic condition with no nephrocalcinosis, and appears to be self-limiting without requiring active intervention. In view of its comparative benign clinical course, such infants with asymptomatic hypercalcaemia and absence of nephrocalcinosis can safely continue to breast feed with monitoring, rather than switching to low calcium formulas which would result in loss of the many benefits of breast milk. The cause for hypercalcaemia in these infants is not yet established and requires further research, but we hypothesise may be due to reduced phosphorus content in maternal breast milk.

### P101

### Management of hypoparathyroidism and pseudohypoparathyroidism in children: A retrospective observation study of 123 patients

Aashima Dabas[1], Lauren Rayner[2], M Zulf Mughal[3], Amish Chinoy[2], Raja Padidela[2,4]

[1]Maulana Azad Medical College, New Delhi, India. [2]Royal Manchester Children's Hospital, Manchester, UK [3]Dubai Health, Dubai, UAE. [4]Manchester Academic Health Science Centre, Faculty of Biology, Medicine and Health, The University of Manchester, Manchester, UK

### Abstract

**Introduction:** Hypoparathyroidism and pseudohypoparathyroidism are clinically heterogeneous disorders characterised by hypocalcaemia. Recommended management is with active vitamin D analogues (aVD) and oral calcium supplementation. This study evaluated long-term management and complications in children with these conditions.

**Methods:** Medical records of 123 children (82 with hypoparathyroidism, 41 with pseudohypoparathyroidism) treated between 2003-2023 were reviewed. Clinical data, serial biochemistry, renal imaging, dosages of aVD and oral calcium requirements were reviewed. All patients were encouraged to ensure adequate dietary calcium intake with minimal dependence on oral calcium supplementation except during intercurrent illnesses. The treatment goal in hypoparathyroidism was to maintain serum calcium between 1.9-2.2 mmol/l and in pseudohypoparathyroidism to normalise serum calcium and reduce PTH concentration.

**Results:** Most children (82%) successfully managed without regular oral calcium after an initial short period (median 1 month) (**Table**). Fifteen (18%) patients with hypoparathyroidism and 5/41 (12%) with pseudohypoparathyroidism required regular oral calcium on follow-up (mean dose 24.7 mmol/dl). They had either concurrent vitamin D deficiency or had discontinued aVD. The median serum calcium was the lowest in those with *GCMB* mutation (n=11) with a relatively higher requirement of aVD. Out of 74 children with hypoparathyroidism, 19 (23.2%) did not require aVD at the last follow-up of which 12 were children with *22q11* deletion (8 discontinued post-puberty, 3 during infancy and 1 had mild disease). Twenty out of 25 patients with pseudohypoparathyroidism who were tested had *GNAS* mutations. Six children (14.6%) with pseudohypoparathyroidism did not require aVD on the last follow-up (two had negative genetics). Fourteen (17.1%) children with hypoparathyroidism developed radiological features of nephrocalcinosis (mean spot urine calcium: creatinine ratio of 0.32) but none with pseudohypoparathyroidism.

**Conclusion**: This study shows effective long-term management of hypoparathyroidism and pseudohypoparathyroidism in children, with most avoiding regular oral calcium dependence. *GCMB* mutations presented as the most severe phenotype with higher calcium and aVD requirements. Children with *22q11* had a milder course of illness. Children with pseudohypoparathyroidism did not develop nephrocalcinosis and had low aVD requirements.

Table: Clinical and Laboratory parameters as per Genotype

| Parameter | Hypoparathyroidism | | | | Pseudohypo-parathyroidism (n=41) |
|---|---|---|---|---|---|
| | *GCMB* mutation (n=11) | *CaSR*mutation (n=5) | *22q11* deletion (n=27) | Others/ Unclassified (n=29) | |
| Age at diagnosis*, y | 0.1 (0.1, 0.2) | 0.2 (0.1, 2.43) | 7.85 (0.13, 10.8) | 8.48 (2, 12.45) | 10.1 (4.5, 14.5) |
| Males, n | 8 | 1 | 16 | 14 | 18 |
| Hypothyroidism, n | 1 | 0 | 3 | 4 | 23 |
| Seizures at onset *, n | 10 | 2 | 6 | 7 | 8 |
| Require AED*, n | 2 | 1 | 0 | 0 | 3 |
| Duration of oral calcium after diagnosis, mo | 11.0 (2.88, 48) | 0.5 (0,2.5) | 0 (0, 3.5) | 2.5 (0, 6.75) | 0 (0, 3.75) |
| Baseline Ca*, mmol/L (2.2-2.7) | 1.72 (1.34, 1.79) | 1.8 (1.6, 1.85) | 1.94 (1.73, 2.24 ) | 1.5 (1.31, 1.79) | 1.95 (1.41, 2.26) |
| Nadir Ca*, mmol/L (2.2-2.7) | 1.59 (1.50, 1.78) | 1.74 (1.5, 2.09) | 1.90 (1.8, 2.15) | 1.87 (1.6, 2.0) | 2.31 (2.21, 2.41) |
| Baseline Pi*, mmol/L | 2.81 (2.43, 2.57) | 2.45 (2.18, 3.01) | 1.76 (1.57, 2.48) | 2.22 (1.85, 3.13) | 2.01 (1.36, 2.36) |
| Baseline ALP, U/L | 241.5 (220.8, 519.8) | 255 (183.5, 322.8) | 173.5 (167, 247.8) | 175.5 (104.0, 265.5) | 236 (136.5, 248) |
| Baseline PTH*, pmol/L (1.6 - 6.9) | 0.6 (0.3, 0.61) | 4.4 (-) | 2.10 (1.25, 2.8) | 1.05 (0.6, 1.62) | 28.2 (13.8, 48.1) |
| Baseline 25OHD, nmol/L (>50) | 115.95 (-) | 56.9 (48.5, 87.9) | (-) | 47 (26, 100.7) | 49.0 (35, 75.1) |
| Follow-up Ca, mmol/L (2.2-2.7) | 2.09 (1.84, 2.14) | 2.13 (1.96, 2.24) | 2.25 (2.14, 2.34) | 2.19 (2.03, 2.35) | 2.31 (2.21, 2.41) |
| aVD dose, ng/kg/d | 42.1 (34.7, 66.2) | (-) | 1.0 (0, 13.0) | 42.3 (18.4, 59.5) | 26.5 (17.7, 41.0) |
| Urine Ca:Cr, mg/mg (< 0.57) | 0.37 (0.21, 0.64) | 1.59 (-) | 0.25 (0.02, 1.65) | 0.43 (0.15, 0.62) | 0.17 (0.04, 0.45) |
| Nephrocalcinosis*, n | 5 | 3 | 1 | 5 | 0 |

Values expressed as median (IQR) or *n*; *p value <0.05 for intergroup comparison between types of hypoparathyroidism; (-) not computed for *n*<5; AED- antiepileptic drugs, PTH- parathyroid hormone, aVD-active vitamin D, Ca:Cr- calcium: creatinine

### P102

### Is there an impact of familial relationship on vitamin D status in children living in Pune, India?

Chirantap Oza, Rubina Mandlik, Swapna Deshpande, Ketan Gondhalekar, Shruti Mondkar, Neha Kajale, Vivek Patwardhan, Sharvani Patil, Chidvilas More, Vaman Khadilkar

Hirabai Cowasji Jehangir Medical Research Institute, Pune, India

### Abstract

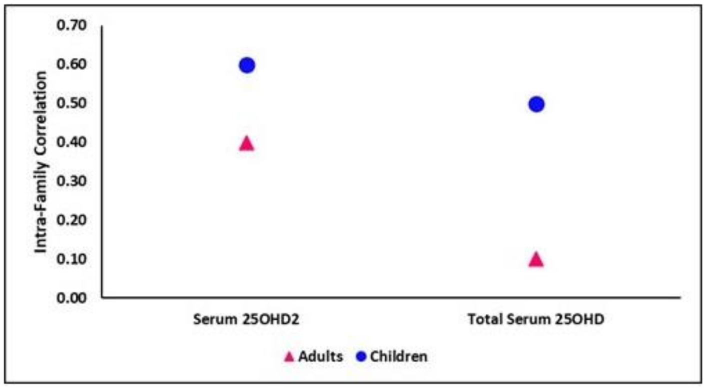

**Objectives:** The knowledge of the influence of familial relationship on vitamin D concentrations could be useful in designing community-based strategies to improve vitamin D status (Madsen 2014). Hence, we planned this study with the aim of exploring if familial relationship has impact on serum $25OHD_2$ and total 25OHD concentrations in children living in Pune, India.

**Methods:** We studied 103 families as a part of a two-year, community-based, longitudinal study designed to explore the effectiveness of consuming vitamin D fortified foods (milk and oil) in improving vitamin D status among families living in Pune, India. Data collected included anthropometry (height and weight), body composition (bioelectric impedance), sunlight exposure (validated questionnaire), and blood samples for estimation of serum $25OHD_2$ and $25OHD_3$ by LC-MS/MS. Total serum 25OHD was computed as the sum of the serum $25OHD_2$ and $25OHD_3$ concentrations. Linear mixed model analysis was employed to assess the impact of family, age, body fat, and sunlight exposure on serum $25OHD_2$ and total 25OHD concentrations. The strength of the family level component was assessed by calculating the intra-family correlation for each model separately for children and adults.

**Results:** The participants included 118 children and 235 adults. The mean age of the children in this study was 11.7 ± 3.7 years and that of adults was 37.6 ± 2.9 years. Intra-familial correlation was found to be much higher in children that in adults for serum $25OHD_2$ (0.6 vs 0.4) and total serum 25OHD (0.5 vs 0.1) concentrations (Figure 1). These results suggest a role played by either genetic factors or possibly shared habits among children in a family.

**Conclusion:** Our findings imply that the likelihood of having similar vitamin D concentrations is higher among children in a family as compared to adults. This can enable implementation of early preventive strategies for treatment or prevention of vitamin D deficiency even if one child among siblings is identified as having inadequate vitamin D concentrations.

### P103

### More than just hypercalcemia: A case of neonatal severe hyperparathyroidism treated with cinacalcet monotherapy

Emma Metivier[1,2], Maria-Elena Lautatzis[3,4], Julia Sorbara[1,2]

[1]Hospital for Sick Children, Toronto, Canada. [2]University of Toronto, Toronto, Canada. [3]Winnipeg Children's Hospital, Winnipeg, Canada. [4]University of Manitoba, Winnipeg, Canada

### Abstract

**BACKGROUND**: Neonatal severe hyperparathyroidism (NSHPT) is a rare disease due to inactivating mutations of the CASR gene. NSHPT presents with severe hypercalcemia, markedly elevated PTH, lethargy, hypotonia, respiratory compromise and fractures.

**PRESENTING PROBLEM**: A term female infant presented with respiratory distress requiring positive pressure support secondary to perinatal depression at birth. Her chest x-ray showed a bell-shaped thorax and multiple bony abnormalities. Subsequent investigations were consistent with primary hyperparathyroidism: total calcium 3.38mmol/L (2.08-2.64mmol/L), PTH 155ng/L (6-89ng/L) and phosphate 1.22mmol/L (1.63-2.83mmol/L). Over the first few days, calcium remained elevated, PTH increased to >2500ng/L. Skeletal survey showed multiple bony abnormalities including suspected pathologic fractures with periosteal reactions.

**CLINICAL MANAGEMENT**: Hypercalcemia was initially managed with hyperhydration, calcitonin and bisphosphonates. The infant was noted to have hypotonia, tachypnea, respiratory distress and bicytopenia (anemia and thrombocytopenia). Whole exome sequencing revealed a homozygous mutation, variant of unknown significance (VUS), in the calcium sensing receptor gene (CASR: c.2333G>T(p.GLy778Val)), suggestive of a diagnosis of NSHPT. Cinacalcet was initiated as definitive therapy. The patient has required high doses, approximately 9 mg/kg/day, to maintain normalization of calcium and PTH. Despite resolution of hypercalcemia, chronic respiratory distress and hypoventilation persisted, requiring home oxygen and nocturnal BiPAP. She is now 2.5 years old and stable on cinacalcet monotherapy. Cytopenias resolved by 4 months and respiratory support weaned off at 15 months.

**DISCUSSION**: This case emphasizes several important clinical features of NSHPT. While the degree of hypercalcemia was mild, the clinical presentation of NSHPT was multisystemic and severe. The VUS was eventually re-classified as a likely pathogenic mutation. Long-term monotherapy with cinacalcet has been safe and effective, although high doses have been required likely due to the location of the genetic mutation. Unlike previously reported cases, the respiratory distress did not improve shortly after normalization of calcium and its persistence was thought to be multifactorial, with hypotonia, neuromuscular weakness and bell-shaped thorax contributing. Finally, this case highlights the impact of PTH on the bone marrow. Unique to this case is the bicytopenia; despite extensive hematologic work-up, a specific etiology was not identified and was attributed to a leukoerythroblastic response secondary to hyperparathyroidism.

### Disclosure

Dr. Emma Metivier: no disclosures Dr. Maria-Elena Lautatzis: no disclosures Dr. Julia Sorbara is a co-investigator on a clinical trial sponsored by Ultragenyx (Sertusumab study)

### P104

### Association of ALP and BALP with linear growth and pQCT-derived parameters in healthy children

Freja Pontán[1], Helena Hauta-Alus[1,2,3,4], Outi Mäkitie[1,2], Diana Atanasova[5], Sture Andersson[1], Per Magnusson[5], Elisa Holmlund-Suila[1,2]

[1]Children's Hospital, Pediatric Research Center, University of Helsinki and Helsinki University Hospital, Helsinki, Finland. [2]Research Program for Clinical and Molecular Metabolism, Faculty of Medicine, University of Helsinki, Helsinki, Finland. [3]3 Population Health Unit, Finnish Institute for Health and Welfare (THL), Helsinki, Finland. [4]Clinical Medicine Research Unit, Medical Research Center Oulu, Oulu University Hospital and University of Oulu, Oulu, Finland. [5]Department of Clinical Chemistry, Linköping University, Linköping, Sweden

### Abstract

**Objectives**: To explore in healthy children the relationship between alkaline phosphatase (ALP) and bone-specific alkaline phosphatase (BALP), and their association with linear growth and peripheral quantitative computed tomography (pQCT)-derived bone parameters. We also examined the effect of vitamin D intervention on ALP and BALP.

**Methods**: In the Vitamin D Intervention in Infants study (VIDI), 987 infants were randomized to receive daily vitamin D3 supplementation of either 10 or 30 µg from the age of two weeks to two years. We conducted a follow-up study in this cohort from 2019 to 2021 when children were 6-8 years old. We determined serum ALP and BALP levels, anthropometry, and bone strength with pQCT in 180 children. We explored the association between ALP and BALP, and differences in ALP and BALP levels among vitamin D intervention groups (Mann-Whitney U-test). The association of ALP and BALP with height and growth rate was assessed through one-way ANOVA with quintiles based on ALP- and BALP-levels. Growth rate was calculated as residuals from a linear model accounting earlier growth.

**Results**: ALP and BALP correlated strongly (Spearman's rho, r=0.93, p<0.001). Vitamin D intervention at 0-2 years had no effect on ALP (p=0.523) or BALP (p=0.394) at 6-8 years. Median (interquartile range) ALP and BALP levels were in the 10 µg vitamin D3 group 289 (226-338) U/L U/L and 88 (72-106) µg/L and in the 30 µg vitamin D3 group, 272 (229-319) U/L and 85 (71-102) µg/L, respectively.
When dividing ALP and BALP into quintiles, height Standard Deviation Score (SDS) and growth rate differed between the quintiles, with the highest quintiles showing the tallest children and highest growth rates (Table 1). No difference was found between ALP- and BALP-quintiles for pQCT-derived parameters (p>0.05 for all parameters).

**Conclusions**: Our study confirms that ALP in healthy children aged 6-8 years consists mainly of BALP, reflecting active bone growth. Our findings also highlight the association between BALP and linear growth, raising the question of whether anthropometric measurements should be considered when interpreting ALP/BALP values. Vitamin D intervention at 0-2 years of age did not affect ALP and BALP levels.

**Table 1:** Differences in height SDS and growth rate in height according to ALP- and BALP-based quintiles, with quintile 1 representing the lowest, and quintile 5 representing the highest level of ALP or BALP.

| | Height SDS | | | | Growth rate in height | | | |
|---|---|---|---|---|---|---|---|---|
| | Quintile | Mean | SD | p-value | Quintile | Mean | SD | p-value |
| ALP | 1 | −0.23 | 0.93 | p=0.033 | 1 | −0.37 | 0.95 | p=0.011 |
| | 2 | 0.096 | 1.02 | | 2 | −0.02 | 1.09 | |
| | 3 | 0.032 | 0.94 | | 3 | 0.03 | 0.95 | |
| | 4 | 0.15 | 1.04 | | 4 | 0.25 | 0.91 | |
| | 5 | 0.52 | 1.02 | | 5 | 0.45 | 1.08 | |
| BALP | 1 | -0.30 | 1.14 | p=0.006 | 1 | −0.16 | 1.13 | p=0.019 |
| | | | | | | | | |

| | | | | | | | | |
|---|---|---|---|---|---|---|---|---|
| | 2 | 0.12 | 0.82 | | 2 | −0.08 | 0.88 | |
| | 3 | −0.096 | 1.04 | | 3 | −0.19 | 0.96 | |
| | 4 | 0.37 | 0.83 | | 4 | 0.36 | 0.91 | |
| | 5 | 0.47 | 1.03 | | 5 | 0.40 | 1.11 | |

Data presented as mean, standard deviation (SD), and p-values.
P-values calculated with one-way ANOVA.
Total n=179, all quintiles n=36, except quintile 1 where n=35.

### P105

### Optimal vitamin D status for Chinese Infants in Hong Kong

Joanna Yuet-ling Tung[1], Hung-Kwan So[2], Ka-Man Yip[2], Sarah Poon[1], Gloria Pang[1], Keith Tsz-Suen Tung[2], Hing-Wai Tsang[2], Wilfred Hing-Sang Wong[2], Patrick Ip[1,2]

[1]Hong Kong Children's Hospital, Hong Kong SAR, Hong Kong. [2]The University of Hong Kong, Hong Kong SAR, Hong Kong

### Abstract

**OBJECTIVES:**

Vitamin D status is commonly assessed by serum 25-hydroxyvitamin D (25OHD) level. However, the cutoff level to define vitamin D deficiency has been controversial. Most studies were performed in Caucasian adults and its applicability to other ethnicity and age groups is uncertain. In this study, we aimed to identify the 25OHD threshold that maximally suppressed parathyroid hormone (PTH) in a group of healthy Chinese Infants in Hong Kong.

**METHODS:**

Healthy infants detected to have low cord blood 25OHD less than 25nmol/L in a population study on vitamin D status were referred to Hong Kong Children's Hospital (HKCH) for further management. Their total 25OHD was repeated with serum calcium, phosphate, alkaline phosphatase and PTH. Three-phase segmented regression was used to identify the optimal breakpoint between 25OHD and PTH.

**RESULTS:**

Two hundred and twelve infants were included (59% male). They were reassessed at a median age of 156 days (IQR: 111-247 days). Upon assessment in HKCH, 19 (9.0%) had persistently low 25OHD <25 nmol/L, while 37 (17.4%) and 156 (73.6%) had 25OHD between 25 to 50 nmol/L and >50 nmol/L respectively. Using unadjusted three-phase segmented regression, the estimated first and second break-point of 25OHD on PTH suppression were 20.0 nmol/L (95% CI: 13.6 to 26.4) and 38.2 nmol/L (95% CI: -16.2 to 90.6) with an r2 of 0.375; whereas the estimated first and second break-point of 25OHD were 20.0 nmol/L (95% CI: 13.1 to 26.9) and 40.4 nmol/L (95% CI: -0.1 to 80.0) with an r2 of 0.402 after adjusting for factors including age, gender, history of vitamin D supplement and mode of feeding. The relationship between 25OHD and PTH was significantly affected by the mode of infant feeding (p = 0.04), but not sex, age, or history of vitamin D supplement.

**CONCLUSIONS:**

The threshold of 25OHD that maximally suppressed PTH estimated in our Chinese infants was lower than that reported in the literature, and hence the optimal vitamin D status might be lower in Chinese infants, though the second breakpoint was not statistically significant. This might be explained by different ethnicity. Further study with larger sample size is needed to validate the observation.

### P106

### Extrapyramidal movements in a child with hypoparathyroidism: An unusual presentation

Louise Apperley, Charlie Jarvis, Suzanne Armitage, Ghadir Kassab, Renuka Ramakrishnan

Alder Hey Children's Hospital, Liverpool, UK

### Abstract

**Background:**

A 9-year-old child with hypoparathyroidism due to 22q11 deletion presented with a one-week history of choreoathetoid movements after recent significant cardiac surgery. He previously had been maintaining low-normal calcium levels on active vitamin D alone. Post-operatively he required calcium supplements, which were subsequently stopped, and he was discharged on active vitamin D.

**Presenting Problem:**

The patient presented two months later with choreoathetosis and bradykinesia movements when awake, along with dysarthria. He had involuntary movements of his arms, legs and face and he had a shuffling gait, causing him to trip over. His speech was slurred and low in volume.

His initial investigations showed a significantly low adjusted calcium (1.68mmol/L) with normal vitamin D and low parathyroid hormone level (Table 1). A computerised tomography scan showed evidence of symmetric periventricular white matter adjacent to the frontal horns of the lateral ventricles, raising the possibility of extrapyramidal movements due to microcalcification in the basal ganglia secondary to hypoparathyroidism. A magnetic resonance imaging of the head has been requested for further evaluation. Rheumatic fever was not considered by the local hospital as a possible diagnosis.

**Clinical Management:**

His hypocalcaemia was managed by optimising his calcium and alfacalcidol doses. Since normalising the calcium and phosphate levels, the choreoathetoid movements have become less frequent and his speech and gait have improved.

**Discussion:**

Abnormal neurology secondary to calcification in the basal ganglia has been shown in the adult population with hypoparathyroidism, but rarely in children. Even in adults, these are often incidental radiological findings, as patients are rarely symptomatic.

These deposits are due to the accumulation of a basophilic substance, consisting of acid mucopolysaccharides. The chronic hyperphosphataemia and hypocalcaemia in hypoparathyroidism is thought to promote calcification due to the presence of osteogenic molecules found within the caudate nucleus.

This is an interesting and unusual case of a paediatric patient presenting with extrapyramidal symptoms likely due to intracranial calcifications secondary to hypoparathyroidism. It is important to recognise this and treat promptly for a good outcome. In adults, extrapyramidal movements are reported to settle down with time once calcium and phosphate levels are maintained within normal range.

*Table 1 shows the results of the investigations completed during his presentation with choreoathetosis movements*

| **Biochemistry Results** | | | |
|---|---|---|---|
| **Test** | **29/12/2023** | **05/01/2024** | **11/01/2024** |
| Adjusted Calcium (mmol/L)<br><br>[2.2-2.6] | 1.68 | 2.32 | 2.09 |
| Phosphate (mmol/L)<br><br>[0.9-1.8] | 2.24 | N/A | 1.49 |
| Alkaline Phosphatase (iu/L)<br><br>[62-425] | 205 | N/A | 132 |
| Parathyroid Hormone (pmol/L)<br><br>[2-9.3] | 0.8 | N/A | 0.9 |
| Vitamin D (nmol/L)<br><br>[>50] | 63 | N/A | 58 |
| **Microbiology Results** | | | |
| **Test** | **Result** | | |
| Throat Swab | Negative for viral infections | | |
| Urine Culture | Negative | | |
| Blood Culture | Negative | | |
| **Imaging** | | | |
| Computerised Tomography scan (head) | Evidence of symmetric periventricular white matter adjacent to the frontal horns of the lateral ventricles. A magnetic resonance imaging of the head has been requested for further evaluation. | | |

*N/A – not available*

### P107

### Vitamin D status and biomarkers of bone health in diabetic adolescents and children in a multi-ethnic cohort

Rashida Farhad Vasanwala[1], Clement K M Ho[2], Nicole K L Lee[1]

[1]KK Women's & Children's Hospital, Singapore. [2]Sengkang General Hospital, Singapore

### Abstract

### Objectives

This prospective study aimed at investigating (1) the prevalence of vitamin D deficiency and insufficiency in diabetic adolescents and children in a multi-ethnic cohort, and (2) any associations between vitamin D status and ethnic background, glycaemic control (HbA1C), metabolic markers of bone health and lipids.

### Methodology

Venous blood was drawn from 199 patients with type 1 diabetes (mean age ± SD, 8.2 ± 3.7 years) and 48 patients with type 2 diabetes (mean age ± SD, 12.7 ± 2.7 years) during their outpatient appointments in a tertiary hospital. Vitamin D status was classified according to serum 25-hydroxyvitamin D (25OHD) concentrations as deficient (less than 12 ng/ml), insufficient (12-20 ng/ml) or sufficient (more than 20 ng/ml)

### Results

Vitamin D insufficiency (25OHD from 12 to 20 ng/ml) was highly prevalent in both type 1 (42%) and type 2 (50%) diabetes patients, whereas vitamin D deficiency (25OHD < 12 ng/ml) was detected in 11% of type 1 and 15% of type 2 diabetes patients respectively. Deficiency or insufficiency of vitamin D was particularly common in the Malay and Indian ethnic groups. Patients of Indian extraction had the lowest mean (± SD) serum 25OHD among all four ethnic groups: 16.1 (± 5.6) ng/ml in type 1 and 14.1 (± 3.8) ng/ml in type 2 diabetes patients respectively. Among type 1 patients, Malays had lower 25OHD than the Chinese ($p<0.01$) and Others ($p<0.05$) ethnic groups.

Vitamin D deficiency or insufficiency was found to be associated with lower calcium ($p<0.05$) and higher PTH ($p<0.001$) concentrations compared with the sufficiency group (25OHD >20 ng/ml) in type 1 diabetes patients but not in type 2 patients. Concentrations of magnesium, phosphate, alkaline phosphatase, HbA1C, total cholesterol, HDL-cholesterol and LDL-cholesterol were not associated with 25OHD in either of the two types of diabetes patients.

### Conclusions

Statistically significant differences in 25OHD concentrations were detected among the four ethnic groups. Indian ethnicity is a risk factor for vitamin D deficiency in both types of diabetes. Vitamin D deficiency or insufficiency was also associated with biomarkers of poorer metabolic bone health (lower calcium and higher PTH) in type 1 diabetes patients.

### P108

### A toddler with severe vitamin D dependent rickets type 1A (VDDR1A), «hungry bones» and a stormy RSV infection: presentation and therapeutic challenges

Artemis Doulgeraki[1], Eleni Statha[2], George Paltoglou[2], Eleni Vakali[3], Elpis Vlachopapadopoulou[3], Kyriaki Karavanaki[2], Elena Fryssira[4]

[1]Department of Bone and Mineral Metabolism, Institute of Child Health, Athens, Greece. [2]2nd Department of Pediatrics, National and Kapodistrian University of Athens (NKUA), “P. & A. Kyriakou” Children’s Hospital, Athens, Greece. [3]Department of Endocrinology-Growth and Development, “P. & A. Kyriakou” Children’s Hospital, Athens, Greece. [4]Medical Genetics Laboratory, National and Kapodistrian University of Athens (NKUA), “Aghia Sophia” Children’s Hospital, Athens, Greece

### Abstract

#### Background:

Vitamin D Dependent Rickets Type 1A (VDDR1A) is an autosomal recessive disorder due to mutations in the CYP27B1 gene, which result in inability to generate 1,25$(OH)_2$D.

#### Case presentation:

A 18-month-old boy with VDDR1A, presented with hypotonia and respiratory distress. He had been diagnosed two months earlier, having been evaluated for stunted growth, hypotonia and delayed developmental milestones. He was stabilized with oxygen and bronchodilators for his bronchiolitis and high doses of alphacalcidol, calcium and phosphate supplements for his “hungry bone” syndrome. Additionally, he sustained upper limb fractures after a fall from his bed during admission. Overall, he had a protracted disease course; however, as his bone profile was improving, he gradually recovered.

#### Conclusion:

VDDR1A causes failure to thrive, hypotonia, increased fracture risk and it may complicate the clinical course of lower respiratory tract infections. Furthermore, management of hungry bone syndrome requires supraphysiologic doses of vitamin D metabolites and calcium

**P109**

Withdrawn

### P110

### Is phosphate supplementation safe in ENPP1-related disease?

Martin Munteanu[1], Yvonne Nitschke[2], Frank Rutsch[2], Uwe Kornak[3], Corinna Grasemann[1]

[1]Department of Pediatrics, Division of Rare Diseases, Katholisches Klinikum Bochum, Ruhr-University Bochum, Bochum, Germany. [2]Department of General Pediatrics, Muenster University Children's Hospital, Münster, Germany. [3]Institute of Human Genetics, University Medical Center Göttingen, Göttingen, Germany

### Abstract

**Background***: ENPP1* encodes for the ectonucleotide pyrophosphatase/phosphodiesterase 1; an enzyme which catalyzes the degradation of adenosine triphosphate (ATP) to adenosine monophosphate (AMP) and pyrophosphate (PPi). Deficiency of ENPP1 due to biallelic loss-of-function-variants in the *ENPP1*-gene causes may cause generalised arterial calcification of infancy (GACI1, MIM# 208000) in neonates, an ultra-rare disease associated with high mortality.

In survivors of GACI and in older children and adolescents *ENPP1*-deficiency is also associated with autosomal-recessive hypophosphatemic rickets 2 (ARHR2, MIM# 613312), characterized by FGF23-dependent phosphate wasting. Therapeutic options are limited to supplementation with phosphate and calcitriol and may present a risk for vascular calcification

**Case**: We present the case of a 17-year-old male with no medical history of GACI who first presented to the tertiary endocrine unit for evaluation for short stature (-2,42 SDS) at 9 years of age.  Hormonal work-up was unremarkable but hypophosphatemia, elevated PTH (84.3 pg/ml [norm 15-65 pg/ml]) and skelettal findings indicated hypophosphatemic rickets. Genetic analysis confirmed ARHR2 due to a pathogenic homozygous *ENPP1*-variant (NM_006208) c.2677G>T, p.(Glu893*).

Given high burden of disease due to pain (lower extremities) a phosphate supplementation by 25 mg/kg/d 3/daily was initiated at 11 years of age after exclusion of arterial (carotid) and heart valve calcification via ultrasound.

Bone Mineral density was unremarkable.

On follow-up visits no effect of the low-dose phosphate supplementation on pain and alkaline phosphatase serum levels was noted.  An echocardiographic re-evaluation 6 months following therapy initiation revealed calcification on the aortic valve (figure 1).  Phosphate supplementation was terminated. On follow-up echocardiographies no further change or calcification was noted until present.

**Discussion**: Phosphate supplementation may be harmful in GACI-survivors / patients with ARHR2, an observation that is backed by case reports in the literature. Evidence for efficacy for this treatment is lacking. We suggest thorough screening for calcifications in these patients.

### P111

### Robotic walking for bone and muscle health in youth with cerebral palsy

Erin Hodgson[1,2,3,4], Elizabeth G Condliffe[1,2,4,5,6], Leigh Gabel[1,2,3,4]

[1]Biomedical Engineering Graduate Program, University of Calgary, Calgary, Canada. [2]Faculty of Kinesiology, University of Calgary, Calgary, Canada. [3]McCaig Institute for Bone and Joint Health, University of Calgary, Calgary, Canada. [4]Alberta Children's Hospital Research Institute, University of Calgary, Calgary, Canada. [5]Department of Clinical Neurosciences and Pediatrics, University of Calgary, Calgary, Canada. [6]Hotchkiss Brain Institute, University of Calgary, Calgary, Canada

### Abstract

**Objectives**: Cerebral Palsy (CP) is the most prevalent childhood motor disability affecting ~2-3/1000 births. Weight-bearing physical activity is essential for developing a strong skeleton; however, ~1/3 of children with CP cannot walk independently, compromising bone health. As such, research into novel robotic trainers provides a promising alternative for children with mobility impairments. Robotic gait trainers offer partial weight-bearing and independence while providing support and stability. Therefore, the objective of this study is to assess the effect of a 3 month walking intervention with a robotic walker on bone and muscle strength in non-ambulatory youth with CP.

**Methods**: This is an ongoing study in 19 participants with CP who are unable to ambulate independently. Participants aim to obtain ≥30 mins/day for ≥5 days/week of robotic walking in a Trexo trainer for 3 months. Peripheral quantitative computed tomography (pQCT) imaging of the tibiae was acquired at the 3%, 38%, and 66% sites prior to and following the intervention on 11 participants. Seven participants were excluded due to motion artifact and one is currently training. Data were converted to age-, sex-, and ethnicity matched z-scores based on a typically developing population to differentiate between typical growth and interventional effects.

**Results**: Currently, three participants have completed baseline and follow-up scans (4.9M, 6.5M, and 9.5F years, respectively). At baseline, trabecular BMD (Tb.BMD) at the 3% site was significantly lower than typically developing children for all participants (-5.94 to -0.31 z-score). Tb.BMD z-scores increased by 9-28% over the trial, such that follow-up z-scores ranged from -5.37 to -0.28. One participant had valid baseline and follow-up scans at the 38% site indicating a 28% increase in Ct.BMD z-score from 1.34 to 1.72 at follow-up and their Ct.BMC z-score increased by 5% (-3.67 to -3.49) over the trial. The MCSA z-score from one participant at the 66% tibia site improved by 1.0% (-4.21 to -4.16) and muscle density increased by 2.7% (1.99 $mg/mm^3$) over the trial.

**Conclusions**: Preliminary results showing improvements in bone and muscle measures are encouraging and suggest the robotic walker may improve bone and muscle health in this population. Continuation of the study with additional participants will solidify conclusions.

A)

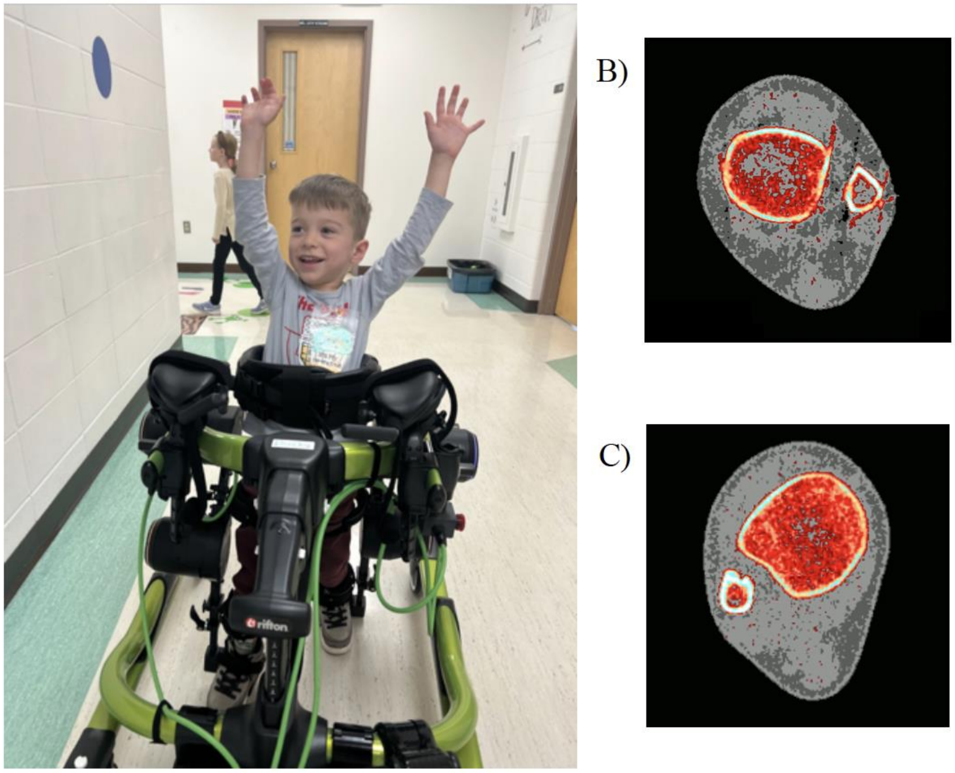

Figure 1. A) Trexo robotic gait trainer. B) 3% slice of Right tibia of 9.3 year old Male with cerebral palsy (CP). C) 3% slice of Left tibia of 8.2 year old typically developing Female.

### Disclosure

Trexo Robotics provided in-kind support for this study.

### P112

### ENMC recommendations for optimizing bone strength in neuromuscular disorders

Anne Dittrich[1], Chiara Panicucci[2,3], Sara Liguori[4], Antimo Moretti[4], Nicol Voermans[5], David Weber[6], Leanne Ward[7,8]

[1]Amalia Children's Hospital, Nijmegen, Netherlands. [2]IRCCS Istituto Giannina Gaslini, Genoa, Italy. [3]Centro di Miologia Traslazionale e Sperimentale, Genoa, Italy. [4]University of Campania "Luigi Vanvitelli", Naples, Italy. [5]Radboudumc, Nijmegen, Netherlands. [6]Children's Hospital of Philadelphia, Philadelphia, USA [7]Children's Hospital of Eastern Ontario, Ottawa, Canada. [8]University of Ottawa, Ottawa, Canada

### Abstract

**Objectives**: To describe the deliberations and action items arising from the 274th European Neuro Muscular Centre (ENMC) workshop held in Hoofddorp, NL Jan 19-22, 2024. The goal of the workshop was to deveolop "Recommendations for Optimizing Bone Strength in Neuromuscular Disorders".

**Methods**: Twenty participants, including three patient representatives plus experts in bone health and neuromuscular medicine, convened to discuss bone strength in NMDs. The workshop aimed to review literature, identify knowledge gaps, and formulate plans for enhancing clinical care and guiding future research. Participants reviewed and summarized existing evidence in presentation format. Post-meeting, each presenter provided brief summaries of their findings. Two attendees were assigned to compile this information for knowledge dissemination.

**Results**: Patient representatives highlighted the lack of routine and standardized bone health care as part of NMD treatment. Clinician surveys revealed varying practices despite moderate to good awareness of bone strength importance. Workshop discussions covered terminology, definitions, and normal bone strength development. The complex interplay between muscles and bones in NMDs, influenced by biomechanical and biochemical factors were explored. Factors such as chronic steroid exposure and nutritional challenges escalate bone fragility, increasing the risk of osteoporosis and fractures, and negatively impacting quality of life. Challenges for the clinical assessment of bone quality include technical issues and data interpretation. While bisphosphonates are commonly used, long-term efficacy and safety data in NMDs are insufficient. Most evidence supporting bisphosphonate use is from pediatric experience, especially in Duchenne muscular dystrophy. Extending knowledge to other NMDs and incorporating bone health management into overall care recommendations is crucial. The workshop concluded with plans for further research and initiatives to disseminate knowledge. Key recommendations included integrating bone strength into NMD patient assessments, considering clinical context over diagnostic tests, avoiding trivialization of peripheral or other fractures, emphasizing longitudinal skeletal phenotyping, conducting multi-site skeletal assessments, evaluating recovery potential without bone-targeted therapy, and promoting multidisciplinary osteoporosis prevention and treatment.

**Conclusions**: It is crucial to improve bone management in all individuals with neuromuscular disorders. Educating clinicians and patients on bone issues and creating awareness are key first steps. Highlights and recommendations from this workshop are in preparation for publication.

### P113

### Comorbid sarcopenia status and bone mineral density in young survivors of cancer. The iBoneFIT Project

Andres Marmol-Perez[1,2], Esther Ubago-Guisado[1], José J Gil-Cosano[3,1], Andrea Rodriguez Solana[1], Juan F Pascual-Gazquez[4], Francisco J Llorente-Cantarero[5,6,7], Vicente Martinez-Vizcaino[8], Manuel Muñoz-Torres[9,10,11,12], Jonatan R Ruiz[1], Luis Gracia-Marco[1,10,6]

[1]Department of Physical Education and Sports, Faculty of Sport Sciences, Sport and Health University Research Institute (iMUDS), University of Granada, Granada, Spain. [2]Department of Epidemiology and Cancer Control, St. Jude Children's Research Hospital, Memphis, USA [3]Department of Communication and Education, Loyola University Andalusia, Sevilla, Spain. [4]Pediatric and adolescent hematology and oncology service, Pediatrics and Pediatric Surgery Clinical Management Unit, Virgen de las Nieves University Hospital, Granada, Spain. [5]Maimonides Biomedical Research Institute of Cordoba (IMIBIC), Cordoba, Spain. [6]CIBEROBN, Biomedical Research Networking Center for Physiopathology of Obesity and Nutrition, Carlos III Health Institute, Madrid, Spain. [7]Department of Specific Didactics, Faculty of Education, University of Cordoba, Cordoba, Spain. [8]Health and Social Research Center, Universidad de Castilla-La Mancha, Cuenca, Spain. [9]Endocrinology and Nutrition Unit, University Hospital Clínico San Cecilio, Granada, Spain. [10]Biosanitary Research Institute, ibs.Granada, Granada, Spain. [11]Department of Medicine, University of Granada, Granada, Spain. [12]Biomedical Research Network in Fragility and Healthy Aging (CIBERFES), Instituto de Salud Carlos III, Madrid, Spain

### Abstract

**OBJECTIVES**: to examine whether bone health differs by sarcopenia status in young paediatric cancer survivors.

**METHODS**: Cross-sectional study including 116 pediatric cancer survivors (12.1±3.3 years old; 42% female) enrolled on a randomized controlled trial designed to improve bone health (iBoneFIT project). Handgrip strength was used to assess muscle strength. DXA estimated areal bone mineral density (aBMD, g/cm$^2$) and appendicular lean mass index (ALMI, kg/m$^2$). Sarcopenia status was determined using age and sex specific international reference data from a healthy population. "No sarcopenia" was defined when muscle strength was > decile 2. "Sarcopenia probable" was defined when muscle strength was ≤ decile 2 and ALMI Z-score was > -1.5 Standard Deviation (SD). "Sarcopenia confirmed" was defined when muscle strength was ≤ decile 2 and ALMI Z-score ≤ -1.5 SD. Analysis of covariance was used to examine differences in age-, sex- and race-specific aBMD Z-score by sarcopenia status.

**RESULTS**: Survivors with sarcopenia confirmed had significantly lower aBMD Z-score than survivors with no sarcopenia at total body (-1.2 [95% CI: -1.5 to -0.8] vs. 0.2 [95% CI: -0.2 to 0.6], P < .001), lumbar spine (-0.7 [95% CI: -1.1 to -0.3] vs. 0.4 [95% CI: 0.0 to 0.8], P < .001), total hip (-0.5 [95% CI: -0.9 to -0.2] vs. 0.4 [95% CI: 0.1 to 0.8], P < .001) and femoral neck (-1.0 [95% CI: -1.4 to -0.6] vs. 0.1 [95% CI: -0.3 to 0.4], P = .001). In comparison to survivors with sarcopenia probable, survivors with sarcopenia confirmed had significantly lower aBMD Z-score at total body (-1.2 [95% CI: -1.5 to -0.8] vs. -0.2 [95% CI: -0.7 to 0.4], P = .009), total hip (-0.5 [95% CI: -0.9 to -0.2] vs. 0.5 [95% CI: -0.1 to 1.0], P = .010) and femoral neck (-1.0 [95% CI: -1.4 to -0.6] vs. 0.1 [95% CI: -0.5 to 0.7], P = .014). No differences were found between survivors with sarcopenia probable and no sarcopenia (P > .607).

**CONCLUSIONS**: These findings suggest that interventions to mitigate osteosarcopenia in this population should be implemented at early stages after treatment completion.

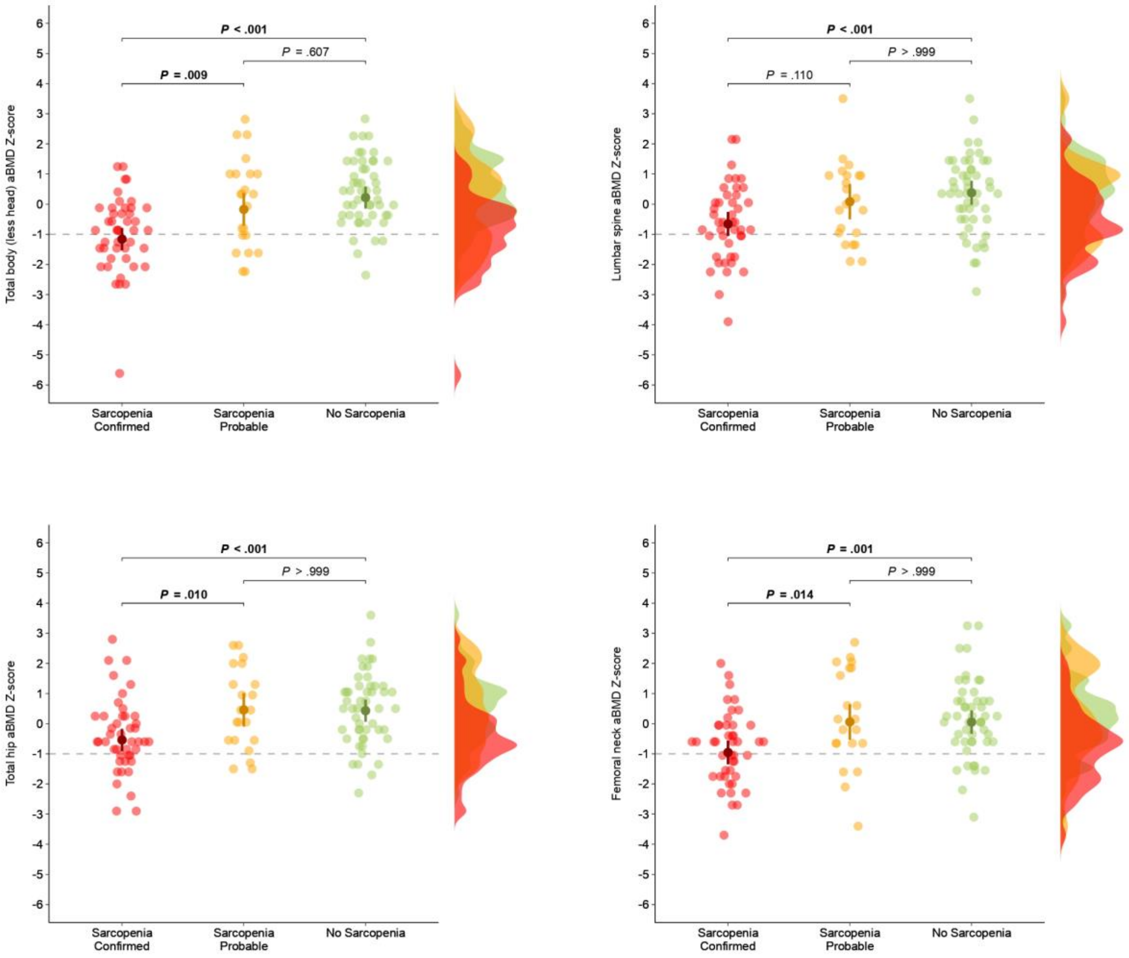

### P114

### Sarcopenia and its influence on bone health in children and adolescents with obesity

Marcela Zembura, Kamila Czepczor-Bernat, Paweł Matusik

Department of Pediatrics, Pediatric Obesity and Metabolic Bone Diseases, Faculty of Medical Sciences in Katowice, Medical University of Silesia, Katowice, Poland

### Abstract

**Objectives:**

According to the European Working Group on Sarcopenia in Older People, sarcopenia could be diagnosed when low muscle strength is present, and diagnosis is confirmed when low muscle mass occurs, additionally, if low physical performance is present, sarcopenia is considered severe.

Sarcopenic obesity (SO) is defined as obesity with low skeletal muscle function and mass.

Regarding the mechanical and biochemical muscle-bone crosstalk, the aim of this study was to evaluate association between bone parameters and elements of sarcopenia (muscle strength and physical performance) in children and adolescents with obesity.

**Methods:**

A total of 95 children and adolescents with obesity (diagnosed with the use of International Obesity Task Force criteria) with a mean age of 12.7( ± 3) years participated in the study. Bone mineral content (BMC) and bone mineral density (BMD) were assessed with dual-energy X-ray absorptiometry—DXA (Hologic). A dynamometer was used in order to measure grip strength. Six-minute walk test (6MWT) was used to assess physical performance.

**Results:**

Handgrip strength was positively and significantly correlated with bone mineral content in both arms (left arm $r=0.575$ $p<0.001$, right arm $r=0.604$ $p<0.001$), trunk ($r=0.553$ $p<0.001$), both legs (left leg $r=0.528$ $p<0.001$, right leg $r=0.525$ $p<0.001$) and bone mineral density in both arms (left arm $r=0.464$ $p<0.001$, right arm $r=0.531$ $p<0.001$), ribs on both sides (left side $r=0.457$ $p<0.001$, right side $r=0.370$ $p<0.001$), pelvis ($r=0.417$ $p<0.001$), thoracic spine ($r=0.397$ $p<0.001$) and both legs (left leg $r=0.483$ $p<0.001$, right leg $r=0.398$ $p<0.001$) with age and gender used as covariates. Moreover, handgrip strength correlated positively and significantly with bone mineral content and bone mineral density in L1-L4 vertebrae ($r=0.479$ $p<0.001$;$r=0.386$ $p<0.001$ respectively) (adjustment for age and gender). Six-minute walk test correlated positively and significantly only with bone mineral content in left arm ($r=0.225$ $p<0.05$) and right arm ($r=0.235$ $p<0.05$) (adjustment for age and gender).

**Conclusions:**

Our results indicated that muscle strength and physical performance are positively correlated with bone parameters in children and adolescents with obesity.

Considering rapid growth of childhood obesity rates, relation between muscle and bone parameters in children and adolescents with obesity is of growing importance.

**P115**

### Systematic review of pharmacological and non-pharmacological therapies for prevention and treatment of osteoporosis in Duchenne Muscular Dystrophy

Sarah McCarrison[1,2], Shima Abdelrahman[2], Rosaline Quinlivan[3], Richard Keen[4], Sze Choong Wong[1,2]

[1]Department of Paediatric Endocrinology, Royal Hospital for Children, Glasgow, UK [2]Bone, Endocrine & Nutrition Research Group in Glasgow, Human Nutrition, Glasgow, UK [3]Department of Neuromuscular Diseases, UCL Queen Square Institute of Neurology, London, UK [4]Metabolic Bone Disease Unit, Royal National Orthopaedic Hospital, Stanmore, UK

### Abstract

**OBJECTIVES:** Glucocorticoid treatment is commonly used in Duchenne Muscular Dystrophy (DMD), but is associated with a high incidence of fragility fractures. This systematic review aims to assess the current evidence for pharmacological and non-pharmacological treatment for osteoporosis in children and adults with DMD, with the goal of guiding future management strategies.

**METHODS:** Three online databases (Embase, Medline, Cochrane Library) were searched for studies that evaluated interventions for treatment or prevention osteoporosis in DMD. The included studies had to report changes in bone mineral density (BMD) Z-scores or fracture incidence.

**RESULTS:** Seventeen studies were identified, including eleven on bisphosphonate treatment, two on testosterone therapy, one on vitamin D/calcium, one on teriparatide treatment, and two on vibration therapy. Only two randomised-controlled trials were found, one for bisphosphonate (zoledronic acid) with 24-month follow-up and one for vibration therapy with 14-month follow-up. Among the bisphosphonate studies, four investigated intravenous administration, five oral administration, one intramuscular administration, and one both intravenous and oral administration. Changes in lumbar spine BMD Z-scores ranged from -0.3 to +1.3 with bisphosphonate treatment, from +0.13 to +0.38 with testosterone therapy, +0.9 with vitamin D/calcium, and from -0.2 to 0.0 with vibration therapy. The zoledronic acid trial showed a significant difference of +1.4 standard deviations in lumbar spine BMD Z-scores between the treatment and no treatment groups. Less information was available regarding the impact of treatment on fracture incidence, although data from the randomised trial of intravenous zoledronic acid showed a lower rate of vertebral fractures in the treatment group (15%) compared to the no treatment group (24%). None of the studies involved a population without prior fractures, and none addressed individuals over 18 years.

**CONCLUSIONS:** This systematic review provides evidence for the effectiveness of bisphosphonate therapy in improving bone density in children and adolescents with DMD. However, there is less information regarding fracture reduction. The review did not find studies exclusively in those over 18 years old with DMD and limited information on non-bisphosphonate pharmacological agents. Developing new clinical guidance for osteoporosis in children and adults with DMD will require expert consensus in conjunction with published evidence.

### P116

### Management of osteoporosis in Duchenne Muscular Dystrophy: Results of a international clinician survey

Sarah McCarrison[1,2], Shima Abdelrahman[2], Justin H Davies[3], Talat Mushtaq[4], Raja Padidela[5], Vrinda Saraff[6], Claire Wood[7], Sze Choong Wong[1,2]

[1]Department of Paediatric Endocrinology, Royal Hospital for Children, Glasgow, UK [2]Bone, Endocrine & Nutrition Research Group in Glasgow, Human Nutrition, University of Glasgow, Glasgow, UK [3]Department of Paediatric Endocrinology, Southampton Children's Hospital, Southampton, UK [4]Department of Paediatric Endocrinology, Leeds Teaching Hospitals NHS Trust, Leeds, UK [5]Paediatric Endocrine, Royal Manchester Children's Hospital and Faculty of Biology, Medicine & Health, University of Manchester, Manchester, UK [6]Birmingham Women's and Children's Hospital NHS Foundation Trust, Birmingham, UK [7]Department of Paediatric Endocrinology, Great North Children's Hospital, Newcastle upon Tyne, UK

### Abstract

**OBJECTIVES:** The current international Care Considerations for Duchenne Muscular Dystrophy (DMD) recommend initiation of bisphosphonate following first fracture. Given the extent and burden of osteoporosis, some have advocated initiating therapy prior to fracture, although this is not standard practice. Our objective was to investigate the current clinical practice of clinicians managing osteoporosis and their opinion on treatment prior to fracture in DMD.

**METHODS:** An online survey was circulated to paediatric clinicians involved in the management of osteoporosis in DMD via patient groups in four countries (UK, USA, Italy, Israel).

**RESULTS:** A total of 51/105 (48%) responses were received. The two commonest indications for starting bisphosphonate (multiple responses allowed) were vertebral fracture (VF) of any grade without back pain (44/51,86%) or long bone fracture (34/51,67%). 18% (9/51) would initiate treatment without any fracture. IV Zoledronate was the most common agent (44/51,86%) used following fracture (multiple responses allowed), with 7/51(14%) responders opting to prescribe oral bisphosphonate. 9/51(18%) currently use other non-bisphosphonate agents (Denosumab, Teriparatide, Romosozumab) following fracture. Of those who prescribe IV bisphosphonate, 26 (52%) prescribe sick-day steroid with first infusion and 18 (36%) with subsequent infusions; 44 (88%) prescribe oral calcium supplements with first infusion and 39 (78%) with subsequent infusions; 32( 64%) prescribe anti-pyretics with first infusion and 19 (38%) with subsequent infusions. 28 (56%) adjust the dose of subsequent infusions and 30 (60%) alter frequency of subsequent infusions based on bone density. Of those who transitioned patient care to adult bone specialists (n=36), 21(58%) continue bisphosphonate during this process, 4 (11%) stop treatment and 7 (19%) develop individualised plans in joint transition review with the adult bone specialist. Of the 51 responders, 20 (39%) are of the opinion that bisphosphonates should be initiated prior to fracture, 24 (47%) feel there may be a role and 7 (14%) do not see a role.

**CONCLUSIONS:** This international clinician survey identified variation in management of osteoporosis in DMD. Expert recommendations on management of side-effects of IV bisphosphonate, the role of non-bisphosphonate therapies, management during transition and longer-term osteoporosis treatment in adulthood is greatly needed. There was no consistent view on initiation of osteoporosis therapy prior to fracture in DMD.

### P117

### Fracture femur in young people with Duchenne muscular dystrophy: Characteristics, management and follow-up

V Wilson[1], S Brown[2], R Hart[2], I Horrocks[2], S Joseph[2], J McCaul[1], S C Wong[3,4]

[1]Department of Paediatric Orthopaedic, Royal Hospital for Children, Glasgow, UK [2]Department of Paediatric Neurology, Royal Hospital for Children, Glasgow, UK [3]Bone, Endocrine, Nutrition Group in Glasgow, Human Nutrition, University of Glasgow, Glasgow, UK [4]Department o Paediatric Endocrinology, Royal Hospital for Children, Glasgow, UK

### Abstract

### Objectives

To review the characteristics, management and follow-up of fracture femur in young people with Duchenne Muscular Dystrophy(DMD) in a retrospective service evaluation(2013-2023).

### Methods

Clinical data were obtained from electronic case records. All fractures were confirmed radiologically and images were re-reviewed jointly by an orthopaedic registrar and a senior paediatric orthopaedic consultant. Results reported as median(Range).

### Results

Twenty-nine low trauma fracture femur in 18/80 boys(23%) with DMD with 580.4 person-years follow-up period were included in this study. The incidence of fracture femur was 310.1/10,000 person-years in boys with DMD compared with 3.3/10,000 person-years in healthy UK boys [Copper C et al J Bone Miner Res 2004]. Median age at fracture was 12 years(2,22). Twenty-seven fractures(93%) occurred whilst the boys were on glucocorticoid; 2/29(7%) occurred in two boys prior to diagnosis of DMD. Twenty-one(72%) were distal femur fracture, four(14%) neck of femur, two(7%) sub-trochanteric and two(7%) femoral shaft fractures. Of the 25 fractured femur(excluding four with fractured neck of femur), 18(72%) had atypical features in accordance with the American Society of Bone Mineral Research classification system. 11/18(61%) were in bisphosphonate naïve boys, 6/18(33%) were in those on bisphosphonate treatment and one with previous exposure to bisphosphonate. Four(17%) fractured neck of femur in four ambulant boys were treated operatively with internal fixation. Two(7%) separate episodes of fractures in one non-ambulant boy were treated by manipulation under anaesthetic followed by immobilization. Twenty-three(79%) fractures in 14 boys(10/23, 43% ambulant at time of fracture) were treated non-operatively with immobilisation. There was no structured rehabilitation programme in place. Hydrotherapy was used in a small number. Repeat imaging was performed in 23/29 at a median of one week (1,6) post-fracture. 10/23(43%) had follow-up until radiological union.

### Conclusion

In this retrospective review, the incidence of fracture femur was 94 times higher in DMD compared with healthy boys in the UK Distal femur was the commonest site of fracture, with a proportion of fractures with atypical features. We report variability in management, follow-up and rehabilitation of fracture femur in DMD. National guidance and care pathways should now be developed alongside prioritization of multi-centre outcome audit.

### P118

### The use of testosterone for delayed puberty in adolescents with Duchenne Muscular Dystrophy: An international clinician survey

Sarah McCarrison[1,2], Shima Abdelrahman[2], Justin H Davies[3], Talat Mushtaq[4], Raja Padidela[5], Vrinda Saraff[6], Claire Wood[7], Sze Choong Wong[1,2]

[1]Department of Paediatric Endocrinology, Royal Hospital for Children, Glasgow, UK [2]Bone, Endocrine & Nutrition Research Group in Glasgow, Human Nutrition, University of Glasgow, Glasgow, UK [3]Department of Paediatric Endocrinology, Southampton Children's Hospital, Southampton, UK [4]Department of Paediatric Endocrinology, Leeds Teaching Hospitals NHS Trust, Leeds, UK [5]Paediatric Endocrine, Royal Manchester Children's Hospital and Faculty of Biology, Medicine & Health, University of Manchester, Manchester, UK [6]Birmingham Women's and Children's Hospital NHS Foundation Trust, Birmingham, UK [7]Department of Paediatric Endocrinology, Great North Children's Hospital, Newcastle upon Tyne, UK

### Abstract

**OBJECTIVES:**

Delayed puberty is very common in glucocorticoid treated boys with Duchenne Muscular Dystrophy (DMD) and is an additional threat to bone health. The current (2018) International Care Considerations for DMD recommend consideration of testosterone for treatment of hypogonadism from the age of 12 years, supported by recent studies that show that testosterone treatment in DMD improves bone density. Our objective was to investigate the current clinical practice amongst clinicians who manage testosterone treatment in DMD.

**METHODS:**

An online survey was circulated to paediatric clinicians involved in the management of puberty in DMD via patient groups in four countries(UK, USA, Italy, Israel).

**RESULTS:**

A total of 49/105 (47%) responses were received. 45% (22) of clinicians reported initiating testosterone therapy in boys with DMD with delayed puberty from 14 years, 35% (17) from 13 years and 35% (17) from 12 years (multiple responses allowed). The majority of responders (47/49,96%) prescribe intramuscular therapy, 31% (15) topical, 2% (1) oral and 22% (11) reported other methods including subcutaneous testosterone. Regarding duration of therapy (multiple responses allowed), 35% (17) of clinicians used a kick-start period of 4-6 months; 43% (21) prescribed continuous gradual build-up of therapy until either adult replacement dose was achieved, adult virilization was achieved (11,22%), testes were greater than 6-8mls (17,35%) or until LH levels were detectable (4,8%). 14% (7) continue testosterone indefinitely into adulthood. In those that discontinue therapy (multiple responses allowed), testicular function was monitored upon discontinuation most commonly by reviewing testicular volume (31/48,65%), monitoring of morning (25/48,52%) or non-timed (12/48,25%) LH, FSH and testosterone levels or by GnRH stimulation testing (6/48,13%). Regarding thresholds for re-initiating testosterone in DMD upon discontinuation, there was a wide variation in cut-off levels reported and reliance on both non-timed and morning testosterone levels.

**CONCLUSIONS:**

This international survey confirms initiation of testosterone in DMD in line with existing international Care Considerations at 12-14 years to address delayed puberty and as adjunctive therapy for bone health. However, there is wide variation in treatment strategies especially in relation to duration of therapy. Further guidance, particularly in regard to optimal regime, monitoring after therapy discontinuation and the need and threshold for re-initiating testosterone therapy is required.

### P119

### Duchenne Muscular Dystrophy and risk of fracture

Kelly Maury[1], Oscar Brunetto[1], Ruben Abdala[2], Sandra Piamonte[1]

[1]Hospital Pedro Elizalde, Buenos Aires, Argentina. [2]IDIM, Buenos Aires, Argentina

**Abstract**

A high risk of fractures was reported in a patient with duchenne muscular dystrophy (DMD ) which could be due to inmobilization, the pathophysiology of the disease , treatment with glucorticoids (GCs) and increased BMI . Knowledge of the risk factors for the disease contributes to creating preventive strategies that reduce co-morbidities and improve the quality of life in these patients.

**Objective:**

To assess the incidence of first fractures and factors contributing to risk of first fracture in children with DMD

**Methods:**

A retrospective analysis of a cohort of patients with Duchenne Muscular was carried out A total of 27 children under 18 years of age with clinical and molecular diagnosis of DMD were enrolled. The study was approved by the ethics committee and all procedures were performed following the Declaration of Helsinki. The study began in 2010, a total of 27 children were referred to our endocrinology service for their evaluation. Anthropometric variables were evaluated at the start of the study and during follow-up. In addition, annual spine radiographs were performed. Data on long bone (LBF) and vertebral fractures (VF) were recorded. Questionnaires about calcium intake and mobility were also administered. The variables are summarized in mean (SD), median (IQR) and percentage according to their nature.

**Results:**

The age of DMD diagnosis was 6.52±2.83y and the follow-up time was 7.77 (±4.57y).Age of starting GCs was 8.12±2.45y and average daily was 0.6±0.12 mg/k/d. 7 children with new fractures (VF or LBF) were observed during this period, median time 5.07 (RIQ 3-6.66y) .Cumulative incidences was 0.26 or 26%. Children with fractures presented a delay in the age of diagnosis (8 vs 5.5 y) and increased BMI ($p=0.01$). No differences were observed in biochemical markers, vitamin D, time and dose of corticosteroids between fractured and non-fractured.

**Conclusion:**

We observed a high incidence of fractures in this population, which highlights the importance of a multidisciplinary approach and the creation of strategies to reduce modifiable risk factors to improve the quality of life of these children. BMI could be a risk factor to take into account when considering treatment strategies in these patients.

### P120

### Changes in lean mass and fat mass in children with osteogenesis imperfecta

Holly Wright[1,2], Sze Choong Wong[3,2], Misha Gilani[2,1], Helen McDevitt[2], Sheila Shepherd[2], Avril Mason[2]

[1]School of Medicine, University of Glasgow, Glasgow, UK [2]Department of Paediatric Endocrinology, Royal Hospital for Children, Glasgow, UK [3]Bone, Endocrine, Nutrition Group in Glasgow, Human Nutrition, University of Glasgow, Glasgow, UK

### Abstract

**Objectives**:

Deficits in skeletal muscle and function is a recognised feature of Osteogenesis Imperfecta (OI). Less is known about longitudinal change in body composition in OI. Our objective was to perform a retrospective analysis of longitudinal change in body composition with focus on lean mass and fat mass in children with OI.

**Methods:**

Data was collected from 29 children with a diagnosis of OI, who had at least two dual energy x-ray absorptiometry (DXA) scans performed between 2015 and 2022. Assessed variables of height, body mass index (BMI), lean mass index (LMI) and fat mass index (FMI), converted to standard deviation scores (SDS), were compared. Results were reported as median (range).

**Results:**

Median age at baseline and follow-up were 10.7 and 14.2 years, respectively.

Median height-SDS at baseline was -1.10, which was significantly lower than the normal population ($p<0.001$).

Median height-SDS at latest follow-up was -0.80, which was not significantly different from baseline ($p=0.870$).

Median BMI-SDS at baseline was 0.15, which was not significantly different than the normal population ($p=0.804$). Median BMI-SDS at latest follow-up was 0.02, which was not significantly different from baseline ($p=0.730$).

Despite normal BMI, abnormalities in body composition were noted.

At baseline, median LMI-SDS was -2.43, which was significantly lower than the normal population ($p<0.001$).

Median LMI-SDS at follow-up was -1.78, which was not significantly different from baseline ($p=0.080$).

At baseline, median FMI-SDS was 0.57 which was significantly higher than the normal population ($p=0.001$). Median FMI-SDS at follow-up was 0.62, which was not significantly different from baseline ($p=0.540$).

**Conclusion:**

Children with OI have abnormal body composition throughout childhood typically with low lean mass and relatively high fat mass. These abnormalities in body composition do not change with follow-up. Strategies to improve lean mass, including physical or medical therapies, should be explored in OI given the close relationship between muscle and bone.

### P121

### Investigating the relationships between physical therapist assessed muscle function, bone density and lean mass in children at risk of skeletal fragility

Mallory Gardner[1], Babette Zemel[1,2], Eva Partridge[1], David Weber[1,2]

[1]The Children's Hospital of Philadelphia, Philadelphia, USA [2]Perelman School of Medicine at the University of Pennsylvania, Philadelphia, USA

### Abstract

OBJECTIVES:

The paradigm for initiating bone protective pharmacotherapy in children at high risk of skeletal fragility is shifting to primary fracture prevention. We hypothesize that assessing muscle function will help identify children with skeletal weakness prior to fracture. The objectives of this cross-sectional study were to establish the feasibility of physical therapist (PT) measured muscle function in children at risk of skeletal fragility and to investigate the associations between muscle function, bone mineral density (BMD), and lean mass (LM).

METHODS:

Leg and handgrip strength were assessed by handheld dynamometry, power by broad jump, and endurance by 6-minute walk test (6MWT). Areal BMD (aBMD) and LM were measured by DXA. Outcomes were converted into and age and sex specific Z-scores using standard references. Data are reported as mean ± standard deviation or median (interquartile range).

RESULTS:

Subjects included 12 children with primary [42% female, median age 11 (8-13) years] and 13 with secondary [39% female, median age 13 (12-15) years] skeletal fragility. Lifetime fracture prevalence was 83% in primary vs 69% in secondary skeletal fragility, p=0.41. Mean height-Z adjusted hip, lumbar spine, and radius aBMD-Z were -1.5±1.4, -0.9±1.4 and -1.4±1.1, respectively. Hip aBMD-Z was lower in primary vs secondary fragility (p=0.01), other sites did not differ. PT outcomes did not differ by skeletal fragility type and were -1.5±0.6 (knee extension-Z), -1.8±1.1 (knee flexion-Z), -1.7±1.7 (6MWT-Z), -1.1±1.2 (broad jump-Z), and -1.1±0.5 (grip strength-Z). Pearson correlations between muscle function-, aBMD- and LM Z-scores are shown in Table 1. Leg strength correlated with leg lean mass [LLM, flexion: R=0.61, p<0.01; extension: R=0.58, p<0.01)], but not hip aBMD; 6MWT and broad jump correlated with hip aBMD (R=0.47, p=0.02 and R=0.72, p<0.01, respectively), but not LLM. Grip strength was associated with aBMD at the radius (R=0.44, p=0.03), but not other sites. DXA LM outcomes [lean mass index (LMI), appendicular LMI, LLM] were not correlated with aBMD.

CONCLUSIONS:

PT performed assessments of muscle function are feasible in children at risk for skeletal fragility. Strength, power, and endurance assessments appear to capture different aspects of the muscle-bone unit which could inform their respective uses in clinical practice.

**Pearson Correlations Between Muscle Function and DXA aBMD and Lean Mass Z-Scores**

| | Hip aBMD | LS aBMD | Radius aBMD | LMI | ALMI | LLM |
|---|---|---|---|---|---|---|
| ***Strength*** | | | | | | |
| **Knee Flexion** | 0.38 | 0.09 | 0.04 | 0.43* | 0.46* | 0.61* |
| **Knee Extension** | 0.09 | -0.04 | 0.12 | 0.37 | 0.45* | 0.58* |
| **Handgrip** | -0.07 | 0.01 | 0.44* | 0.22 | 0.34 | 0.33 |
| ***Endurance*** | | | | | | |
| **6 Minute Walk** | 0.47* | 0.41* | 0.16 | -0.14 | -0.16 | -0.13 |
| ***Power*** | | | | | | |
| **Broad Jump** | 0.72* | 0.48* | 0.43* | 0.01 | 0.04 | 0.16 |

aBMD, areal bone mineral density; ALMI, appendicular lean mass index; LLM, leg lean mass; LMI, total body lean mass index; LS, lumbar spine

* statistically significant, $p<0.05$

### Disclosure

DRW has received grant funding from Inozyme and consulting fees from PTC therapeutics.

### P122

### Acceptability and barriers of exercise in children with osteogenesis imperfecta

Misha Gilani[1], Stacey Todd[1], Sze Choong Wong[1,2], Helen McDevitt[1], Avril Mason[1]

[1]Paediatric Endocrinology, Royal Hospital for Children, Glasgow, UK [2]University of Glasgow, Glasgow, UK

### Abstract

**Objectives**:

To define barriers to participation in exercise in paediatric osteogenesis imperfecta (OI) and the feasibility of an exercise intervention in paediatric OI.

**Methods**:

17 children with OI, at the Royal Hospital for Children Glasgow were surveyed anonymously; to assess exercise habits, perceived barriers to exercise, and acceptability of an exercise intervention as a potential treatment for improvement of muscle-bone outcomes.

**Results**:

Median age of the population was 12.5 years (range 3.6-17.7). 11/17 (65%) were fully ambulant, 1/17 (6%) ambulant with an aid, 3/17 (18%) occasional wheelchair users and 2/17 (12%) full-time wheelchair users. 16/17 (94%) were currently being treated with bisphosphonate therapy (oral or IV) and 5/17(30%) were using pain relieving medication on at-least a weekly frequency. 16/17 (94%) of respondents said having OI made participation in exercise more difficult due to joint pain (69%), muscle weakness (50%), tiredness/fatigue (75%) and concern about fracture (75%). All children surveyed participated in sport, outside of school, at least once a week and 15/17 (88%) stated they would participate in an at least one additional 30 minutes of exercise per week to improve muscle and bone strength, most commonly swimming (71%).

**Conclusions**:

Despite barriers to participation in exercise in OI most children surveyed would wish to participate in an exercise intervention to improve muscle-bone.

### P123

### Long term effects of vitamin D supplementation with dairy vs pharmacological calcium on bone health of underprivileged Indian children and youth with poor glycaemic control of type-1 diabetes

Chirantap Oza, Anuradha Khadilkar, Neha Kajale, Gauri Khot, Shruti Mondkar, Vivek Patwardhan, Sharvani Patil, Chidvilas More, Vaman Khadilkar

Hirabai Cowasji Jehangir Medical Research Institute, Pune, India

### Abstract

**Objective:**

In a previous study, we report that 1-year supplementation of vitamin D with milk (group A) and vitamin D with pharmacological calcium (group B) caused similar improvement in bone mineral density (BMD) and cortical thickness of underprivileged Indian children and youth with poor glycaemic control of type-1 diabetes (T1D) with a more pronounced effect in girls. Since pharmacological calcium is more cost effective, it was suggested as better alternative to optimise bone health in resource limited settings. We hypothesize that due to anabolic effect of milk protein, participants of group A would have prolonged effect on bone health than participants of group B. The objective of this study was to compare the effect of supplementation of vitamin-D with milk vs pharmacological calcium on bone mass accrual one year after completion of supplementation.

**Methods:**

Participants of group A received 200ml milk+ 1000 international unit (IU) vitamin-D3/day and participants of group B received 500 mg of calcium carbonate+1000 IU of vitamin-D3 daily for a period of 12 months. One year after the completion of intervention period, all study participants who continued to receive standard diabetes care, were re-evaluated for anthropometric, biochemistry and bone health parameters (using dual-energy x-ray absorptiometry and peripheral quantitative computed tomography- DXA and pQCT respectively).

**Results:**

We studied 130 study participants of whom 64 belonged to group A and 66 to group B. There was no significant difference in the BMD at lumbar spine level, however, the mean total body less head BMD Z score remained same in group A, the mean reduction of -0.2 was noted in group B. There is significant difference in decrease in cortical thickness (24.7% vs 38.5%) in participants of group B as compared to group A, but no difference is observed in reduction in trabecular density. Figure 1 shows trend in mean DXA and pQCT parameters in the study group post-supplementation.

**Conclusion:**

Supplementation of vitamin D with milk has more prolonged effect on bone accrual of Indian children and youth with T1D as opposed to vitamin D with pharmacological calcium.

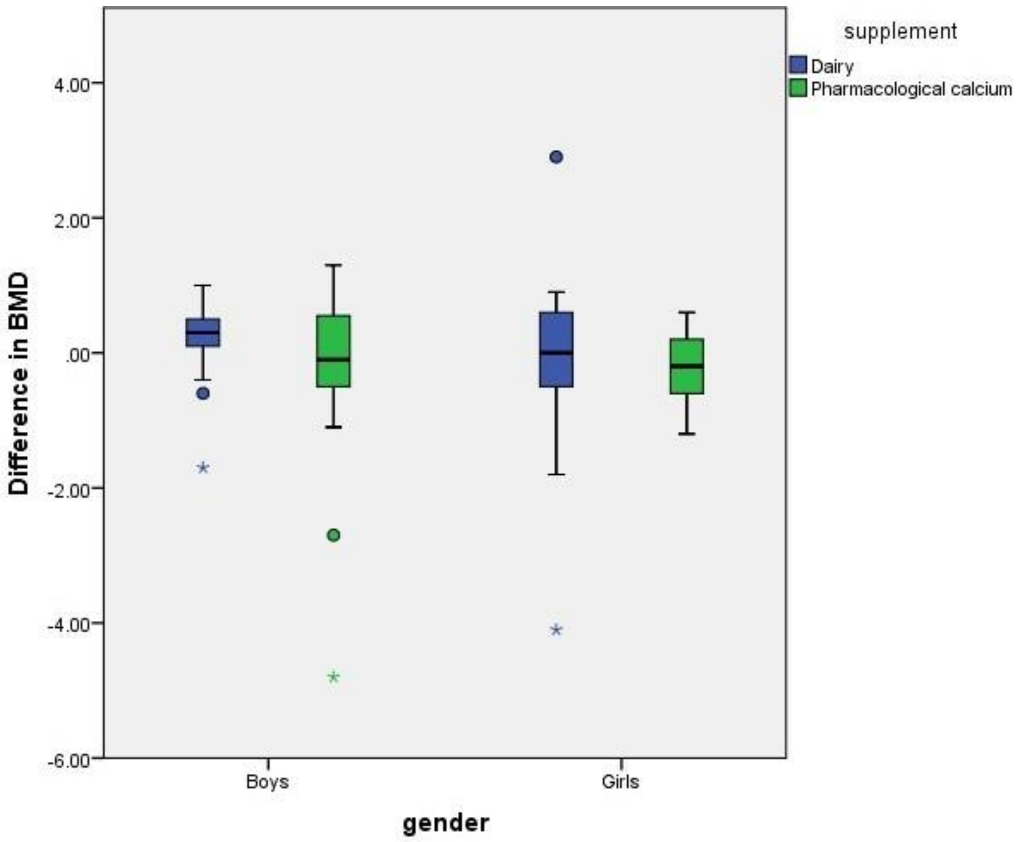

supplement
Dairy
Pharmacological calcium
4.00
2.00
.00
-2.00
-4.00
-6.00
Difference in BMD
Boys
Girls
gender

### P124

### The potential contribution of sulfated vitamin D metabolites to infant nutrition

Carmen Reynolds, Roy Dyer, Sara Oberhelman-Eaton, Brianna Konwinski, Renee Weatherly, Ravinder Singh, Tom Thacher

Mayo Clinic, Rochester, Minnesota, USA

### Abstract

**Objectives:**

Vitamin D is essential for preventing rickets in breastfeeding infants. Maternal supplementation is the preferred method for infant nutrition, yet concentrations of vitamin D and 25-hydroxyvitamin D in breastmilk are low unless the mother consumes substantial doses of vitamin D. We hypothesized that sulfated vitamin D metabolites are contributing to the vitamin D composition of breastmilk for infant nutrition.

**Methods:**

Breastmilk and serum were assessed at baseline and 28 days after 5000 IU/day $VitD_3$ (cholecalciferol) supplementation in 20 lactating women. Vitamin $D_3$-sulfate ($VitD_3$-S) and $25OHD_3$-sulfate ($25OHD_3$-S) were measured by mass spectrometry. A subset of milk samples was used to assess storage effects.

**Results:**

Baseline concentration of $VitD_3$-S in breastmilk was 6.4±3.9 nM and was greater than both serum $VitD_3$ ($p$ = 0.018) and serum $VitD_3$-S ($p$ <0.001). Baseline $25OHD_3$-S in breastmilk was 0.47±0.09 nM. High dose supplementation increased mean±SD serum 25-hydroxyvitamin D concentrations from 68.5±19.2 nM to 95.6±15.7 nM, but neither sulfated metabolite was affected by supplementation ($VitD_3$-S, $p$=0.81; $25OHD_3$-S, $p$=0.62). Metabolites remained intact when stored at 21°C or 4°C for ≤6 hrs, but not at 4°C for 24 hrs. Degradation occurred via structural isomerization rather than sulfate hydrolysis.

**Conclusions:**

$VitD_3$-S and $25OHD_3$-S are important components of breastmilk from vitamin D-sufficient mothers and are not affected by excess maternal intake of $VitD_3$. Sulfated vitamin D metabolites, however, are not stable for overnight refrigeration. We conclude that $VitD_3$-S and $25OHD_3$-S in breastmilk contribute to the infant's breastmilk diet, with $VitD_3$-S being actively secreted into the milk, and that infants who regularly consume stored milk may not be consuming adequate levels of these sulfated vitamin D metabolites.

### P125

### Age and sex-related parameters of total body lean and fat mass in Ukrainian children and adolescents

Anna Musiienko[1], Nataliya Balatska[2], Nataliia Grygorieva[1], Maryna Bystrytska[1]

[1]D.F. Chebotarev Institute of Gerontology of the National Academy of Medical Sciences of Ukraine, Kyiv, Ukraine. [2]Bogomolets National Medical University, Kyiv, Ukraine

### Abstract

**OBJECTIVES**:

To study the age and sex peculiarities of total body lean and fat mass indices of Ukrainian children and adolescents evaluated by dual-energy X-ray absorptiometry (DXA).

**METHODS**:

590 healthy children and adolescents (254 boys and 336 girls) aged 5 to 17 years from various regions of Ukraine were enrolled into the study. The height and weight of the examined were between the 3rd and 97th percentiles. The average age of the group was 12.50±3.74 years (12.44±3.87 years in boys and 13.87±3.64 years in girls). The body mass index (BMI) of males was 17.57±3.28 kg/m$^2$, and in females BMI was 18.08±3.28 kg/m$^2$. Total body lean mass (TBLM) in kg and total body fat mass (TBFM) in kg were measured using DXA (Hologic Discovery WI, USA, 2016).

**RESULTS:**

The results of the study are presented in the table. Parameters of TBLM and TBFM increased with age, parallel to BMI in both sexes. We didn't find any sex difference in the proportion of TBLM and TBFM in children up to 11 years. From 12 to 17 years of age, males' TBLM increased by 86.7%, and females' one – by 44.4%. TBFM, on the contrary, increased more prominently in females compared to males, its increment from 12 to 17 years was 74.5 and 6.8%, respectively.

**Table.** Parameters of the body composition indices of Ukrainian children and adolescents evaluated according to age and sex.

| Age, years | Males | | | | Females | | | |
|---|---|---|---|---|---|---|---|---|
| | n | BMI, kg/m$^2$ | Lean mass, kg | Fat mass, kg | n | BMI, kg/m$^2$ | Lean mass, kg | Fat mass, kg |
| 5 | 11 | 15.6±1.9 | 12.5 [11.6-16.2] | 5.3 [3.1-6.8] | 14 | 14.8±1.7 | 12.3 [10.9-14.6] | 4.7 [4.1-5.8] |
| 6 | 18 | 15.1±1.8 | 15.9 [13.9-18.4] | 4.8 [3.9-5.5] | 11 | 15.4±2.1 | 16.0 [14.0-17.8] | 5.6 [5.3-8.6] |
| 7 | 14 | 15.1±1.9 | 17.3 [15.8-19.2] | 3.9 [3.2-6.6] | 16 | 14.7±1.9 | 15.4 [14.3-17.0] | 5.2 [4.6-7.8] |
| 8 | 10 | 14.8±2.4 | 20.4 [16.6-23.8] | 4.4 [3.8-6.2] | 19 | 15.9±1.8 | 18.2 [16.3-20.9] | 7.7 [6.2-9.2] |
| 9 | 15 | 15.9±2.0 | 23.0 [19.4-25.0] | 7.2 [5.0-8.8] | 22 | 16.1±2.6 | 21.5 [19.9-22.7] | 7.0 [5.8-10.2] |
| 10 | 18 | 16.5±3.9 | 25.4 [21.9-26.9] | 7.6 [5.0-9.8] | 31 | 16.7±2.9 | 23.4 [21.4-26.0] | 7.7 [6.5-9.9] |
| 11 | 19 | 17.1±3.2 | 27.1[24.0-28.2] | 8.8 [6.0-13.2] | 29 | 17.0±2.6 | 26.8 [22.8-28.8] | 9.8 [8.5-15.0] |
| 12 | 22 | 17.7±3.6 | 30.9 [28.0-33.9] | 8.6 [5.1-13.7] | 19 | 17.0±2.3 | 27.3 [23.2-31.3] | 11.4 [8.3- |

| | | | | | | | | |
|---|---|---|---|---|---|---|---|---|
| | | | | | | | | 14.4] |
| 13 | 18 | 17.1±3.3 | 34.6 [29.0-38.6] | 9.2 [7.5-12.3] | 31 | 18.7±2.7 | 33.9 [30.3-36.1] | 13.3 [10.6-15.9] |
| 14 | 33 | 18.7±2.4 | 40.4 [37.8-44.8] | 9.9 [7.3-13.4] | 37 | 19.7±3.1 | 34.2 [31.8-36.7] | 15.4 [11.6-20.5] |
| 15 | 17 | 18.1±1.9 | 42.6 [39.8-47.6] | 8.3 [6.6-12.8] | 39 | 19.7±3.0 | 33.1 [44.1-36.2] | 17.7 [11.8-21.6] |
| 16 | 27 | 19.3±3.1 | 48.3 [43.8-53.7] | 8.1 [6.4-13.8] | 25 | 20.1±2.8 | 36.6 [35.2-39.9] | 16.2 [11.5-18.4] |
| 17 | 32 | 20.3±3.2 | 50.6 [46.4-57-4] | 9.4 [8.0-13.9] | 43 | 20.8±2.9 | 38.7 [36.2-41.5] | 17.1 [13.6-20.5] |

In the table, the BMI is presented as M±SD. Lean mass and fat mass are presented as M [LQ; UQ]

**CONCLUSIONS:**

Our study showed that the body composition of children does not depend on sex for up to 11 years. However, in adolescents, after 11 years, sex maturation changed the proportion of total body lean and fat mass. Comprising of DXA data revealed that per cent of total body fat mass is higher in females while lean mass in males is. Our results are primary reference values of TBLM and TBFM of DXA-derived measurements for the Ukrainian population and would provide clinicians with useful information in assessing patients with different disorders.

### P126

### Scurvy combined with vitamin D deficiency in a young male with intellectual disability

Myrina Vasiliki Mylona, Konstantinos Roussis, Dimitrios Christakos, Charalampos Valsamis, Artemisia Panou, Ioannis Karnezis, Ioannis Platanitis, Ioanna Paspati

Penteli Children's Hospital, Athens, Greece

### Abstract

**BACKGROUND**: Children with abnormal dietary habits, mental illness, physical disabilities are prone to develop several vitamin deficiencies. Vitamin D deficiency is highly prevalent among children worldwide. Vitamin C deficiency (scurvy), is rare and frequently belatedly diagnosed. Vitamin D deficiency and scurvy's musculoskeletal manifestations are often dramatic and probably irreversible. We present a case of a young, mentally retarded patient, suffering scurvy and vitamin D deficiency, presenting multiple spinal and appendicular skeleton's fractures.

**PRESENTING PROBLEM:** A 19year-old non-verbal, mentally retarded male, initially hospitalized in another hospital due to spontaneous femoral and wrist fractures, was admitted to our clinic due to multiple spinal and appendicular skeleton's fractures. At his admission, he appeared irritable and any movement of his upper limbs or legs seemed to cause great pain.

The initial laboratory examinations revealed severe hypocalcemia, low 25(OH)D levels and high PTH level.

X-rays revealed, apart from the recent fractures of the left femur and the left wrist, severe osteopenia and multiple vertebral bodies deformities of the entire spinal column. CT-scan evaluation revealed new bone formation around the right femur's diaphysis and a large hematoma on the left femur, attributed to the recent fracture, with a surprisingly extended calcification on a two-week follow up X-ray. This picture arouse the suspicion of ascorbic acid deficiency that was confirmed by low levels serum ascorbic acid measured.

**CLINICAL MANAGEMENT:** IV Calcium Gluconate and a single IM dose of ergocalciferol were given immediately. The patient was also given daily supplementation of vitamin D and ascorbic acid, as well as IM B12. Immediately after treatment he showed great symptoms' improvement.

**DISCUSSION**: Vitamin D deficiency poses major public health issues among children and adolescents given that vitamin-D deficiency play a key role in the pathophysiology of many chronic diseases beyond rickets. Scurvy, caused by chronic deficiency of vitamin C, necessary for the maintenance of intercellular connective tissues, osteoid, dentine and collagen, has detrimental effects on bones. In children, scurvy has been associated with subperiosteal hemorrhage/hematomas's calcification/epiphyseal separations.

The two diseases combination jeopardizes vital body's functions increasing severe complications' probability. Scurvy should be included in the differential diagnosis of at-risk populations.

### P127

### Pediatric Scurvy: An uncommon cause for knee pain and limping unveiled through the lens of bone radiographs

Brianna Bowden[1], Stephen Druhan[2], Brent Adler[1], Sasigarn Bowden[1]

[1]Nationwide Children's Hospital, Columbus, USA [2]NYU Grossman School of Medicine, New York City, USA

### Abstract

BACKGROUND:

Scurvy, once considered relegated to the tales of ancient maritime explorations, persists in modern times among individuals with dietary restrictions due to psychiatric illness or neurodevelopmental disabilities. Scurvy affects connective tissues by weakening blood vessels and impairing collagen synthesis, resulting in petechiae, ecchymoses, gingival bleeding, and musculoskeletal symptoms. We present a case of scurvy in a healthy child presenting with knee pain and limping.

PRESENTING PROBLEM:

A 6 year-old male with left knee pain, difficulty walking, and refusal to bear weight on the left knee for 2.5 weeks. Examination revealed diffuse tenderness to palpation of the knee and distal femur. There were no skin lesions.

CLINICAL MANAGEMENT:

Evaluation by his primary care physician revealed normal calcium, phosphorus, low alkaline phosphatase (121 U/L, normal 151-342 U/L), elevated sedimentation rate (70 mm/hour, normal <13 mm/hour), and normal C-reactive protein. Concerned about infection or inflammatory conditions, the patient was referred to the emergency department. The left knee x-ray was initially read as subphyseal irregularity and sclerosis, raising suspicions of stress-related changes or possible fracture. Orthopedics advised non-weight bearing for 2 weeks with a knee immobilizer, and the patient was discharged in a wheelchair. Further review of the knee radiograph on the next day revealed subphyseal sclerosis, a dense zone of provisional calcification (Frankel line), a lucent metaphyseal band (Trummerfeld zone), and metaphyseal spurring indicative of Pelken's spur--indicating scurvy. Dietary history revealed a primary beverage consumption of artificial children's juice with minimal intake of fruits and vegetables. Upon confirmation of undetectable vitamin C levels, he received oral vitamin C and dietary modifications. Within a few weeks, pain and gait significantly improved. At the 6-week follow-up, laboratory tests showed normalized vitamin C and alkaline phosphatase levels. Repeat knee radiographs at 3 months showed substantial improvement.

DISCUSSION:

This case underscores the importance of recognizing radiographic signs in diagnosing scurvy. Scurvy's potential presentation in a healthy child, despite consuming seemingly fruit juices, accentuates the diagnostic challenge in a limping child with a broad differential. Identifying these radiographic signs proves crucial to prevent misdiagnosis and to facilitate timely and effective treatment, leading to rapid improvement.

### P128

### Clinical and biomechanical evaluation of the upper extremity in patients with osteogenesis imperfecta

Katharina Oder[1], Fabian Unglaube[1], Sebastian Farr[1], Andreas Kranzl[1,2], Alexandra Stauffer[1,2], Rudolf Ganger[1,2], Adalbert Raimann[3,2], Gabriel Mindler[1,2]

[1]Orthopedic Hospital Speising, Vienna, Austria. [2]Vienna Bone and Growth Center, Vienna, Austria. [3]Medical University Vienna, Vienna, Austria

### Abstract

**Objectives:** Osteogenesis imperfecta (OI) is a hereditary disorder primarily caused by mutations in type I collagen genes, resulting in bone fragility, deformities, and functional limitations. Studies on upper extremity deformities and associated functional impairments in OI are limited. This study aimed to evaluate upper extremity deformities and functional outcomes in OI.

**Methods:** We included patients regardless of their OI subtypes with a minimum age of 7 years. Radiographic analysis of radial head dislocation, ossification of the interosseous membrane and/or radioulnar synostosis of the forearm were performed and deformity was categorized as mild, moderate, or severe. Clinical evaluation was done using the Quick Disabilities of Arm, Shoulder, and Hand (qDASH) questionnaire and shoulder-elbow-wrist range of motion (ROM). Three-dimensional motion analysis of the upper limb was conducted using the Southampton Hand Assessment Procedure (SHAP). The SHAP quantifies execution time through the Linear Index of Function (LIF) and assesses the underlying joint kinematics using Arm Profile Score (APS). Additionally, maximum active Range of Motion (aRoM) was measured.

**Results:** Fourteen patients aged 8 to 73 were included. Radiographic findings revealed diverse deformities, including radial head dislocation, interosseous membrane ossification, and radioulnar synostosis. 6 patients had a mild, 6 a moderate and 2 a severe deformity of the upper extremity. Severe deformities and radial head dislocation correlated with compromised ROM and worse qDASH scores. The qDASH Score ranged from 0 to 37.5 (mean 11.7). APS was increased and LIF reduced in OI-affected persons compared to non-affected peers. APS and LIF also varied depending on the severity of bony deformities. aRoM was remarkable reduced for pro-supination

**Conclusions:** Patients with OI showed variable functional impairment from almost none to severe during daily life activities, mainly depending on the magnitude of deformity. Larger multicenter studies are needed to confirm these results of this heterogeneous cohort.

### Disclosure

AR, GM received non-related honoraria from Kyowa Kirin and Alexion for consultancy and scientific presentations. GM received non-related honoraria from Biomarin for consultancy and scientific presentations.

### P129

### Cerebral palsy and its impact on occupational performances in childhood

Rastislava Krasnik[1,2], Aleksandra Mikov[1,2], Jelena Zvekić-Svorcan[1,3], Dragana Vukliš[1], Milena Kovačević[1], Dejana Dedić Novaković[1,4]

[1]University of Novi Sad, Faculty of Medicine, Novi Sad, Serbia. [2]Institute of Child and Youth Health Care of Vojvodina, Novi Sad, Serbia. [3]Special Hospital for Rheumatic Diseases, Novi Sad, Serbia. [4]Oncology Institute of Vojvodina, Physical medicine, rehabilitation and EMNG diagnostics department, Novi Sad, Serbia

### Abstract

### Objectives:

Cerebral palsy (CP) results from brain damage occurring during the early stages of its growth and development. Due to various associated difficulties, this condition impacts a child's daily functioning. The aim of this research was to determine which daily life activities are most affected due to the underlying condition.

### Methods:

The study was conducted among 41 children with CP, of both genders, aged 5-18 years, treated at the Clinic for Child Habilitation and Rehabilitation in Novi Sad, Serbia, with prior consent from the child and their parents/guardians, as well as approval from the institution's Ethics Committee. The included children had various levels of gross motor function disability, according to the Gross Motor Function Classification System (GMFCS). The Canadian Occupational Performance Measure (COPM) was used to assess occupational performance.

### Results:

In the total sample (n=41; 24 males (59%)), the average age was 9.5 years. All levels of motor disability were represented on the GMFCS scale (Level I:49%; Level II:10%; Level III:5%; Level IV:7%; Level V:29%). In the domain of personal care, 7 occupational activities were challenging (most commonly dressing (61%) and undressing (66%)). Regarding functional mobility, patients reported 6 difficulties (most commonly walking up and down stairs (56%) and getting in and out of a car (56%)). In terms of leisure/socialization, 68.3% of respondents had difficulties with playing with other children and engaging in free activities.

### Conclusions:

By obtaining information on the most affected occupational performances, more appropriate rehabilitation treatments tailored to children with CP can be planned.

### P130

### Bone fractures in upper and lower extremities and occupational performances in childhood

Rastislava Krasnik[1,2], Čongor Senji[1], Milica Stanić[1,2], Dragana Vukliš[1], Milena Kovačević[1], Mirjana Kolundžić[3]

[1]University of Novi Sad, Faculty of Medicine, Novi Sad, Serbia. [2]Institute of Child and Youth Health Care of Vojvodina, Novi Sad, Serbia. [3]Health Center Novi Sad, Novi Sad, Serbia

### Abstract

### Objectives:

Bone fractures in the upper and lower extremities in childhood represent a significant health issue that affects a child's daily activities. The aim of this research was to determine the impact of fractures on a child's everyday occupational performances.

### Methods:

A prospective study conducted over a 4-month period included 36 participants of both genders, up to 18 years old, treated at the Clinic for Child Habilitation and Rehabilitation in Novi Sad, Serbia, with prior consent from the child and their parents/guardians and approval from the Ethics Committee. The Canadian Occupational Performance Measure (COPM) was used to assess occupational performances.

### Results:

In the total sample (n=36; 25 males (69%)), the average age was 9.94 years. Fractures of the upper extremity were most commonly observed (58%), followed by 31% lower extremity fractures and 11% fractures in both upper and lower extremities. In the domain of personal care, 8 occupational activities were challenging (most commonly brushing teeth (26%) and washing the face (22%)). In the domain of functional mobility, patients reported 6 difficulties (most commonly riding a bicycle (34%) and walking up and down stairs (22%)). In the community navigation domain, the greatest difficulty was using elevators (80%) and 34% of respondents reported difficulties in using their free time and socializing with friends.

### Conclusions:

Fractures in the upper and lower extremities during childhood affect independence, self-care, functional mobility, and daily functioning. Recognizing these difficulties and providing timely rehabilitation to address them can contribute to a faster recovery for the child.

### P131

### The End of Waddling? Surgical knee varus correction decreases lateral trunk lean in children and adults with XLH

Celine Akta[1,2], Andreas Kranzl[1,2], Catharina Chiari[1,2], Alexandra Stauffer[1,2], Adalbert Raimann[3,2], Roland Kocijan[4,2], Gabriel Mindler[1,2]

[1]Orthopedic Hospital Speising, Vienna, Austria. [2]Vienna Bone and Growth Center, Vienna, Austria. [3]Medical University Vienna, Vienna, Austria. [4]Hanusch Hospital, Vienna, Austria

### Abstract

**Objectives:** X-linked Hypophosphatemia (XLH, OMIM 307800) is a rare genetic bone disorder characterized by bone deformities, early osteoarthritis, enthesopathies, pain and reduced quality of life. Disease specific gait deviations with increased lateral trunk lean ("Waddling gait") have been associated with knee varus deformity and add significantly to the burden of disease. This study analysis changes of trunk movement after genu varum surgery in children and adults with XLH.

**Methods:** This retrospective study examined patients with XLH who underwent surgical lower limb varus deformity correction at our institute. Radiographic and gait analysis data was analyzed before and after the surgery. Radiographic analysis of anteroposterior long leg standing x-rays included measurement of mechanical axis deviation and standardized deformity angles.

**Results:** Eight female and four male patients (21 limbs) with a mean age of 20.1 years (5-50, SD: 12.9 years) were eligible for the study. The mean follow up was 20 months; included correction procedures: hemiepiphysiodesis (5 limbs), nails (2 limbs), circular external fixators (10 limbs), four limbs were treated with a combination of procedures (hemiepiphysiodesis, osteotomies). Patients with XLH undergoing lower limb varus correction showed significantly reduced mechanical axis deviation (MAD) postoperatively ($p < 0.001$). Lateral thorax range of motion decreased after surgical lower limb intervention ($p < 0.001$), however, still exceeding the normal range. Further analysis indicates that acute correction (nails) had less improvement of thorax movement than gradual correction techniques (hemiepiphysiodesis, external fixator).

**Conclusions:** This is the first study to assess therapeutic strategies for increased lateral trunk lean. Patients with XLH undergoing varus deformity correction of the lower limbs showed significantly reduced lateral trunk lean. However, normal thorax movement was not achieved in all cases. Further analysis of surgical techniques and co-factors with bigger cohorts are necessary to evaluate this disease specific gait.

### Disclosure

AR, RC, GM received non-related honoraria from Kyowa Kirin and Alexion for consultancy and scientific presentations. GM received non-related honoraria from Biomarin for consultancy and scientific presentations.

### P132

### KLIC score for predicting unfavorable outcomes following hip replacement early acute prosthetic joint infections

Dinghua Yu

Jiangbei Traditional Chinese Hospital, Chongqing, China

### Abstract

**Background:**

Previous studies have reported conflicting results with regard to the predictive significance of the Kidney, Liver, Index surgery, Cemented prosthesis, and C-reactive protein value (KLIC) score for treatment responses. In this study, we aimed to explore the significance of KLIC score for predicting unfavorable outcomes in patients with early acute prosthetic joint infections.

**Methods**:

We prospectively collected data from 64 patients with early acute prosthetic joint infections following hip replacement from a single center during March 2020 and December 2023. The primary endpoint is success or failure of the Debridement, antibiotic agents, and implant retention treatment. Treatment failure was defined as all-cause mortality, unscheduled surgery or prescription with long-term suppressive antibiotics at day 60.

**Results:**

Univariate analysis showed that the a higher KLIC score was associated increased risk for treatment failure (OR=1.32 [95% CI 1.02-1.87], P=0.04). The area under the receiver-operaing characteristic curve for predicting treatment failure was 0.59 (95% CI 0.53-0.68). The predictive significance of the KLIC score disappeared (OR=1.03, 95% CI 0.72-1.34, P=0.53) in the fully adjusted regression model.

**Conclusion**:

The KLIC score has limited efficacy for predicting treatment failure for early acute prosthetic joint infections in patients with hip replacement.

### P133

### Rehabilitation after humerus fracture in childhood - A case study

Dajana Dedić Novaković[1,2], Aleksandra Mikov[3,4], Čila Demeši-Drljan[1,5], Rastislava Krasnik[1,5], Dragana Vukliš[1], Milena Kovačević[1]

[1]University of Novi Sad, Faculty of Medicine, Novi Sad, Serbia. [2]Oncology Institute of Vojvodina, Physical medicine, rehabilitation and EMNG diagnostics department, Sremska Kamenica, Serbia. [3]University of Novi Sad, Faculty of Medicine, Novi Sad, Serbia. [4]Institute of Child and Youth Health Care of Vojvodina, Novi Sad, Serbia. [5]Institute of Child and Youth Health Care of Vojvodina, Novi Sad, Serbia

### Abstract

BACKGROUND:

Fractures of the humerus in children are a common reason for participation in rehabilitation treatment. Limited range of motion in the elbow joint and adjacent joints after a fracture can have a negative impact on daily functioning.

PRESENTING PROBLEM:

We present the rehabilitation process of a 6-year-old boy who suffered a fracture of his dominant right arm (humerus) after falling from a bicycle. Treatment was carried out at the Institute for Child and Youth Health Care of Vojvodina in Novi Sad, Serbia.

CLINICAL MANAGEMENT:

Orthopedic reduction was performed, and the fragments were fixed with 2 Kirschner wires, followed by immobilization. The fixation material and immobilization were removed after 4 weeks. The patient underwent a comprehensive rehabilitation treatment in a hospital setting. The boy had limited range of motion in the right elbow: flexion (95°) and extension (-30°), with no limitation in supination and pronation, as well as movement in the wrist and hand. Kinesiotherapy, thermotherapy with cooled paraffin, electrotherapy (potassium iodide electrophoresis), occupational therapy, continuous passive motion therapy (Artromot®), and hydrotherapy were conducted. Improvement was achieved over two therapy cycles, totaling 4 weeks: passive elbow flexion of 120° (Artromot® 130°), and passive extension of -10° (Artromot® +5). On the BBT (Box & Blocks Test), the patient scored 24 with his right hand and 23 with his left hand.

DISCUSSION:

A multidisciplinary approach, individualized rehabilitation treatment, and a combination of physical modalities contribute to a positive outcome in the treatment of humerus fractures in childhood.

**P134**

Withdrawn

### P135

### Achondroplasia multidisciplinary team clinic evaluation

Emilia Zwierzchowska[1], Adam Watt[1], Anthony Amato-Watkins[2], Roddy O'Kane[2], Philip Davies[3], Panayiotis Constantinou[4], Alexandra Smith[5], Stacey Todd[6], Haytham Kubba[7], Helen McDevitt[8], Sze Choong Wong[9,6], Avril Mason[6]

[1]The University of Glasgow School of Medicine, Dentistry & Nursing, Glasgow, UK [2]Department of Paediatric Neurosurgery, Royal Hospital for Children, Glasgow, UK [3]Department of Respiratory Medicine, Royal Hospital for Children, Glasgow, UK [4]Department of Genetics, Royal Hospital for Children, Glasgow, UK [5]Department of Orthopaedics, Royal Hospital for Children, Glasgow, UK [6]Department of Paediatric Endocrinology, Royal Hospital for Children, Glasgow, UK [7]ENT (Ear Nose & Throat) Department; Royal Hospital for Children, Glasgow, UK [8]Department of Neonatology, Royal Hospital for Children, Glasgow, UK [9]Bone, Endocrine, Nutrition Group in Glasgow, Human Nutrition, University of Glasgow, Glasgow, UK

### Abstract

OBJECTIVES:

The achondroplasia multidisciplinary team (MDT) clinic was created in Glasgow to allow patients to receive coordinated care from paediatric specialists in the following fields: endocrinology, neurosurgery, ENT, respiratory, clinical genetics, orthopaedic surgery and occupational therapy. This survey-based study is aiming to analyse the perspectives of clinicians, and patients' families on the achondroplasia MDT clinics and their effectiveness.

METHODS:

To gather insights from the two groups of interest, two surveys were designed. The clinician's questionnaire included eight questions and the questionnaire for patients' families included fifteen questions. Both incorporated a mix of close-ended and open-ended questions. The surveys assessed various aspects such as satisfaction with the clinics and their organisation, effectiveness of communication with the clinicians, and general feedback.

RESULTS:

The survey received 7 responses from patients' families, and 8 from clinicians. 100% of clinicians, and 71% of families preferred the MDT clinics compared to the previous model of separate appointments. The questionnaire suggested general agreement on the effectiveness and clarity of communication and management plans, although one family reported difficulties with understanding these aspects. There were some suggestions of involving more clinicians in the clinics, particularly: clinical psychology, dentistry and dietetics. Some participants also recommended increased frequency of the clinics and one of the clinicians prompted improvement in room organisation. Moreover, one of the families expressed a preference for a clinic closer to their place of residence. Overall, the survey demonstrated satisfaction and appreciation for the MDT achondroplasia clinics while emphasising on possible areas for improvement to provide holistic and accessible care.

CONCLUSIONS:

In conclusion, the study indicates high satisfaction and appreciation of the MDT achondroplasia clinics among both groups. It highlights the strength of the clinics while offering suggestions of enhancement in terms of inclusion of additional specialities and ensuring that communication is always clear and all information is understood. These findings highlight the significance of ongoing efforts towards improvement and the importance of the clinics' existence, prompting consideration for establishing more of them in the future.

### P136

### Associations between gestational age, birth size, timing of puberty and adult height

Carin Skogastierna[1,2], Nazli Kalay[2], Diana Swolin-Eide[1,2], Anton Holmgren[1,3,4]

[1]Department of Pediatrics, Institute of Clinical Sciences, Sahlgrenska Academy, University of Gothenburg, Gothenburg, Sweden. [2]Region Västra Götaland, Sahlgrenska University Hospital, Department of Pediatrics, Gothenburg, Sweden. [3]Department of Pediatrics, Halland Hospital, Halmstad, Sweden. [4]Department of Research and Development, Region Halland, Halmstad, Sweden

### Abstract

**Objectives:**

To investigate the importance of gestational age and birth size for timing of puberty and adult height.

**Methods**:

We used a newly formed subgroup of individuals from two earlier Swedish cohorts with children born in 1974 and in 1990 (n=4700, m=3255, f[DSE1] =2355). The subgroup comprised healthy individuals with Nordic ethnicity, known parental heights and measured adult height. Birth characteristics (gestational age, birth length and birth weight) were collected, and the longitudinal growth data was individually, visually inspected. Pubertal timing[DSE2] [CS3] was assessed as age at peak height velocity.

**Results**:

Gestational age had no association with adult height or pubertal timing. Birth length and weight could explain 12% and 8% ($p \leq 0.001$) respectively of attained adult height. Including mid-parental height in the model, the degree of explanation was 25% and 21%, respectively. Among children born small for gestational age based on birth length, 8% did not reach target height (mid parental height ±1.5SDS). Neither birth length nor birth weight was associated with pubertal timing.

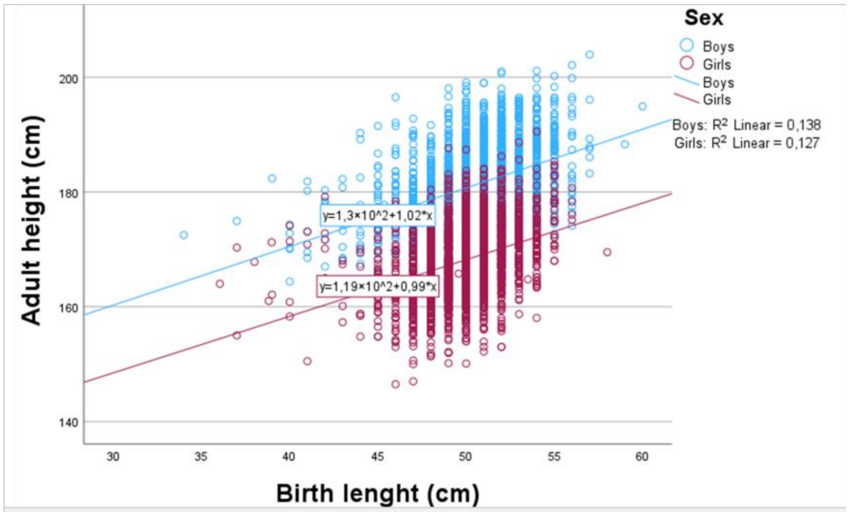

**Figure 1.** Linear relationship between birth length and adult height

**Conclusions**:

This study shows that gestational age is not associated with pubertal timing or adult height. A positive relationship was found between birth size, mainly birth length, and adult height. No association between birth size and peak height velocity was found in this study.

### P137

### Evaluating transition in osteogenesis imperfecta in the West of Scotland

Holly Wright[1,2], Helen McDevitt[2], Sze Choong Wong[3,2], Maria Talla[2], Panayiotis Constantinou[2], Stacey Todd[2], Mandy Fanning[2], Avril Mason[2]

[1]School of Medicine, University of Glasgow, Glasgow, UK [2]Department of Paediatric Endocrinology, Royal Hospital for Children, Glasgow, UK [3]Bone, Endocrine, Nutrition Group in Glasgow, Human Nutrition, University of Glasgow, Glasgow, UK

### Abstract

**Objectives:** Osteogenesis Imperfecta (OI) is a genetic disorder characterised by bone fragility and predisposition to fracture. The increased risk of fracture and skeletal morbidity in this patient cohort is lifelong. Long-term follow up is important in addressing health needs. The aim of this retrospective clinic review was to evaluate the success of transition in OI and to determine factors influencing long-term follow-up in an adult service.

**Methods:** Young people attending the Complex Bone Clinic at Glasgow's Royal Hospital for Children between 2014-2020 were identified. Attendance data was obtained from patient records and an electronic appointment system. Success of transition in OI was determined by the proportion of young people in established follow-up, defined as those young people still attending adult clinic 3 years after transfer. Good late attendance was measured as attending ≥50% of offered appointments at the paediatric Complex Bone Clinic in the 3 years prior to transfer. Good early attendance was measured as those attending ≥50% of offered appointments at any adult bone clinic in the 3 years following transfer.

**Results:** 23 young people (median age 17.5yrs) were identified who had care transferred to an adult service between 2014-2020. 10 (43.5%) of which were in established follow-up at 3 years. 16 (66.7%) young people were offered an initial appointment at the adult service. 18 (78.3%) young people were good late attenders, and 12 (52.2%) young people were good early attenders. Good late attenders were significantly more likely to be offered an initial appointment with an adult service (p=0.017). Those offered an initial appointment for an adult service were significantly more likely to be in established follow-up at 3 years than those not (p=0.007). Good early attendance did not have a significant impact on established follow up (p=0.118).

**Conclusion:** A significant proportion of patients with OI are currently lost to follow-up following transfer to adult clinics. Good late attendance to paediatric clinic has a significant impact on the likelihood of being offered an initial appointment with an adult service, which appears to predict established long-term follow up. Strategies to improve attendance pre-transition, and long-term, are required to ensure lifelong health needs are addressed.

### P138

### Hypercalcemia due to systemic Bacillus Calmette-Guerin infection post vaccination

Ayman Asaly[1,2], Amarilla Mandola[2,3], Michal Stein[2,4], Yael Levy-Shraga[1,2]

[1]Pediatric Endocrinology and Diabetes Unit, The Edmond and Lily Safra Children's Hospital, Sheba Medical Center, Tel-Hashomer, Israel. [2]Faculty of Medicine, University of Tel Aviv, Tel Aviv, Israel. [3]Pediatric Department A and Immunology Service, The Edmond and Lily Safra Children's Hospital; Clinical Immunology, Angioedema and Allergy Unit, Center for Autoimmune Diseases, Sheba Medical Center, Tel-Hashomer, Israel. [4]Pediatric Infectious Diseases Unit, Sheba Medical Center, Edmond and Lily Safra Children's Hospital, Tel-Hashomer, Israel

### Abstract

**Background:**

The Bacillus Calmette–Guérin (BCG) vaccine is a live attenuated vaccine that is included in the routine neonatal vaccination program of the Palestinian authority. Although safe, complications can occur, including local, extra-regional, and disseminated BCG infections, especially in immunocompromised patients. While hypercalcemia has not yet been considered a complication of BCGitis, tuberculosis is a well-known cause of hypercalcemia.

**Presenting problem:**

A 6-month-old male term infant from the Palestinian authority, with normal reported pregnancy and birth, received all his vaccinations. The parents are first-degree cousins with unremarkable family history. He was admitted because of recurrent respiratory infections, chronic diarrhea, recurrent febrile episodes, and failure to thrive. Immunological workup was performed due to suspicion of immunodeficiency, which demonstrated significant lymphopenia, complete T-cell deficiency, and hypogammaglobulinemia. Genetic investigation revealed a homozygous mutation in the IL7R gene, leading to the diagnosis of SCID. Due to fever, respiratory deterioration, lymphadenopathy, and skin lesions, microbiological investigations were expanded. Shoulder abscess aspiration and bronchoalveolar lavage yielded positive results for acid-stable bacilli. Disseminated BCGitis was diagnosed, and combined therapy was initiated. After stabilization, the patient underwent a bone marrow transplantation. One month later, he developed chronic hypercalcemia.

**Management**:

Endocrine evaluation was performed, which revealed a high level of corrected calcium (12.8 mg/dl) and ionized calcium (1.58 mmol/l), phosphor 5.8 mg/dl, hypercalciuria, suppressed PTH (less than 6 pg/ml), high level of 25-hydroxy vitamin D (64 ng/ml) and inappropriate normal 1,25 dihydroxy vitamin D (42 pg/ml). Treatments for hypercalcemia were initiated and included fluids, furosemide, calcitonin and steroids. Calcium levels were normalized.

**Discussion:**

The WHO recommends BCG vaccination of infants at birth, or as soon as possible after birth, as part of routine childhood immunization programs in selected areas. Side effects have been described but hypercalcemia is not one of them yet. Granulomatous diseases, such as tuberculosis, can cause hypercalcemia secondary to the overexpression of 1-alpha hydroxylase enzyme in macrophages. According to the medical literature, two patients diagnosed with bladder cancer who were treated with intravesical BCG therapy developed secondary granulomatous disease and hypercalcemia. Further studies are needed to investigate hypercalcemia as a complication of BCG vaccination.

**Table 1 – Endocrine evaluation**

| | **Blood level** | **Normal range** |
|---|---|---|
| **Calcium** | 12.1 mg/dl | 8.5-10.2 mg/dl |
| **Albumin** | 3.1 g/dl | 3.5-5 g/dl |
| **Corrected calcium** | 12.8 mg/dl | 8.5-10.2 mg/dl |
| **Phosphate** | 5.8 mg/dl | 4-7 mg/dl |
| **25 Hydroxy vitamin D** | 64 ng/ml | 50-70 ng/ml |
| **1,25-Hydroxy vitamin D** | 42 pg/ml | 20-100 pg/ml |
| **Parathyroid hormone** | <6 pg/ml | 15-65 pg/ml |
| **Alkaline phosphatase** | 250 iu/l | 24-147 iu/l |
| **$Calcium_{urine}$/$creatinine_{urine}$** | 4481 mg/g | 12-244 mg/g |

**P139**

Withdrawn

### P140

### Shouldn't the bones of high school girls living normal lives draw more attention?

Hiromi Kawasaki, Kazue Yoshihara, Satoko Yamasaki

Hiroshima University, Hiroshima, Japan

### Abstract

Objectives

Bone density measurement and intervention in Japan focuses on pre- and postmenopausal women. High school students leading normal lives are not measured for bone density. It is important to take an interest in bones starting in adolescence, a critical time for creating peak bone density, reducing the risk of fractures and kyphosis (round back), and maintaining quality of life over a long life span. Is there no problem with bone density in high school girls who look healthy? We clarified the actual bone density of female high school students and examined how health guidance should be provided.

Methods

Sixty-two first-year students of a private high school for girls in Hiroshima City were selected. The girls were surveyed and instructed from August 2016–2017. Bone Stiffness Index, related to bone density, was determined for calcaneal bone using an ultrasonic measuring device (A-1000insight, GE HealthCare Japan). Health guidance on diet, exercise, and sleep was provided.

Results

The Stiffness Index was measured in 60 students. The mean was 99.95, standard deviation 14.68, minimum 63, maximum 127. There was one girl with a Stiffness Index of 63 and two in the 70s. Twenty girls had irregular menstruation. There was a significant association with athletic club affiliation ($p < 0.001$). No significant changes were found in the post-health guidance survey and eating habits, exercise habits, and sleep scores.

Conclusions

Stiffness Index measurements showed that there were girls with a low Stiffness Index. The girls were not previously detected because they did not have the opportunity to have their Stiffness Index measured. However, they needed to be instructed on how to increase peak bone density in adolescence. Group instruction did not show any significant change in their thoughts or behavior. It was suggested that tutoring students whose Stiffness Index was 2 SD below the mean was more efficient than tutoring everyone. Bone density monitoring of female high school students should be added to school health checks to detect those who need individualized instruction. Bone densitometry for all students at school would increase interest in their bones.

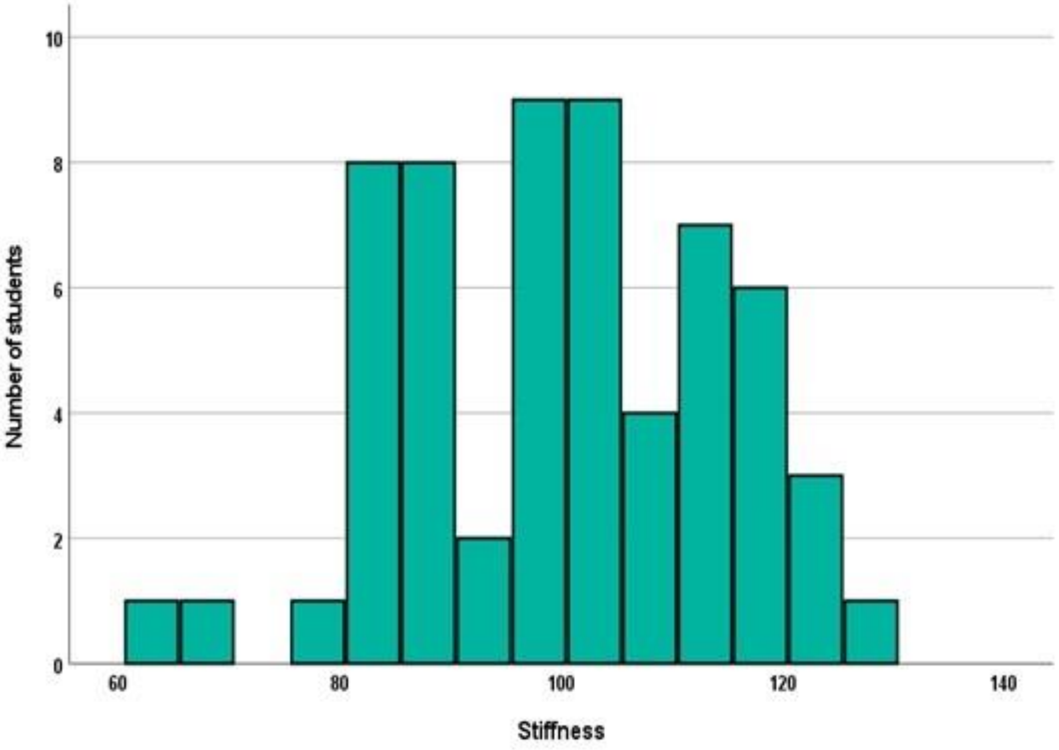

Figure. Distribution of Bone Stiffness Index

### P141

### Striving for change, making a big impact.  How a pilot therapy-led clinic promoted the importance of occupational therapy (OT) intervention for children with skeletal dysplasia's and helped secure dedicated therapy time.

Jill Baker

Sheffield Children's Hospital Metabolic Bone Team, Sheffield, UK

### Abstract

**Background:**

Children with skeletal dysplasia's are often medically well but may have difficulties in achieving independence in activities of daily living. These children have generally been reviewed within a consultant-led multidisciplinary clinic including therapists and nurses. However, time can be relatively limited in this setting due to the need to address multiple aspects of care.

**Presenting Problem:**

It can be difficult to provide a comprehensive and holistic evaluation of the child's functional ability, therapy needs and identify goals.

**Clinical Management:**

In 2022 a pilot therapy-led clinic was trialled for 5 months to undertake therapist activity separately in order to improve the quality of therapy intervention. The clinic evaluated exceptionally well. Data collected from the OT monthly patient contact sheets showed that over30 percent of overall OT caseload was taken up with skeletal dysplasia activity. A successful business case was submitted to the medical directorate to demonstrate that funding was needed to secure dedicated OT hours to enable the role in this group of children to continue.

**Discussion:**

We present an alternative model of multidisciplinary care, the trial of which allowed evaluation which succeeded in securing dedicated hours and enabled service development.

### P142

### Diagnosis, staging and monitoring of paediatric fibrous dysplasia at a Tertiary Paediatric Bone Service in Scotland

Morna Campbell[1], Helen McDevitt[2], Jarod Wong[2], Avril Mason[2]

[1]University of Glasgow, Glasgow, UK [2]NHS Greater Glasgow and Clyde, Glasgow, UK

### Abstract

**Objectives:** A Fibrous Dysplasia (FD) International Consortium has developed guidance for best clinical practice for the definition, diagnosis, staging, and monitoring for Fibrous Dysplasia/McCune Albright Syndrome (FD/MAS). We wished to examine how closely our clinical practice, a tertiary paediatric bone service in Scotland, adhered to the recommendations set out in this guidance for both investigations and frequency of follow-up.

**Methods:** Four patients (aged 9-17), craniofacial FD (n=3) and appendicular FD (n=1), were identified from a clinic database. Two of the four had FD/MAS due to presence of café-au-lait macules. Investigations, at diagnosis and staging, and appropriate monitoring and follow-up were recorded from a clinical electronic record and compared to the recommendations (Table 1).

| **Investigations at Point of Diagnosis and Staging** | | |
|---|---|---|
| **Evaluation of Skeletal System** | **Bone related Blood tests** | **Extra-skeletal Assessment** |
| | Renal Profile | Gonadal |
| | ALP | Thyroid |
| Skeletal Imaging | Bicarbonate | Growth hormone |
| Physical Examination | Calcium | Adrenal |
| Assessment of Bone Pain | Phosphate | Dermatological |
| | Vitamin D | Ophthalmology |
| | PTH | Audiology |

**Results:** Investigations at point of diagnosis and staging for evaluation of the skeletal system: 4 had skeletal imaging, 2 had a physical examination and 1 had assessment of bone pain. All 4 children had investigations at point of diagnosis for the bone related blood tests, however these were not complete. Investigations at point of diagnosis and staging for the extra-skeletal assessment: 4 had dermatological, 3 ophthalmological and 3 audiological assessments. The endocrine assessments performed were variable with thyroid evaluation the most common (n=3). All 4 children met the minimum frequency of follow-up with endocrinology, of once/year.

**Conclusion:** Adherence to recommended investigations for diagnosis and staging in FD/MAS is poor in a Tertiary paediatric bone service. Increasing awareness of published guidance in this rare disease setting and possible development of local pathways could improve clinical care and minimise complications.

### P143

### The complexity of play

Caroline Marr, Rebecca Jones

Sheffield Children's NHS Foundation Trust, Sheffield, UK

### Abstract

**Background:**

Play is a word the evokes many thoughts and ideas. Play can be functional, social, expressive, and creative. It is both a physical and mental activity, that facilitates skill acquisition, and for children underpins the foundations of their development.

**Presenting problem:**

At Sheffield Children's Hospital we wanted to explore play in osteogenesis imperfecta (OI). We felt it important to gain both parent, guardian, and professionals' perspectives investigate what play means to them & how, if any influences the condition has on this activity.

**Clinical management:**

We held two workshops: one for professionals and one for parents/ guardians. Both workshops included questions that focused on how the definition of play, what type of play children with OI engaged in, what toys they chose and their play environment.

**Discussion:**

In both workshops play was seen as problematic. Both parents, guardians and professionals talked about the need to be creative when devising play activities and finding suitable toys for children with OI. Discussion was had about how limb length, facture status, position of the child, their strength, stamina, and fatigue levels effect play. An association with loss was seen throughout the conversations, with participants speaking about loss of fun, loss of experiences and loss of opportunities, not just for the child but for their carer. Parents and guardians spoken about the isolation they felt their child experienced when playing.

From this initial work we feel there is justification to explore this area further. We intend to build upon the insights gained, ensuring that this important childhood activity continues to be support in a way that addresses the needs of parents, guardians, and children.

### P144

### Preparing adults with osteogenesis imperfecta to engage in research on access and quality of care for their rare disorder

Laura Tosi[1], Erika Carter[2], Stacie Connors[2], Michael Stewart[2], Andrew Pennington[1], Kendall Reid[1], Tracy Hart[3]

[1]Children's National Hospital, Washington, USA [2]Osteogenesis Imperfecta Foundation, Gaithersburg, USA [3]Osteogenesis Imperfecta Foundation, Washington, USA

### Abstract

### Background:

While most children with osteogenesis imperfecta (OI) in the US are cared for at one of 57 pediatric OI clinics, there are only 5 adult clinics. Thus, adult OI patients seeking specialized OI care face major challenges 1) transitioning to adult care and 2) gaining access to sites with the capacity to provide appropriate care. Excellent research has been done on the broad issues of transitioning to adult care, but virtually all publications assume that clinicians and systems are prepared to take on care responsibilities for all disorders. The problem for patients with OI is that, in general, there is a dirth of healthcare resources available to meet their unique needs.

### Methods:

The three aims of this project included 1)preparation of OI community members/stakeholders for participation in patient engagement, 2) utilization of patient-centered outcomes research to identify gaps and opportunities in adult OI care, and 3) creation of a strategic plan for leaders of the OI Foundation (OIF) to build on these findings. The project included five phases: 1) establishment of Patient and Scientific Advisory Boards, 2) development of scripts for and conducting listening sessions with volunteers from the OI adult and parent/caregiver community, health care providers and health policy leaders, 3) summarizing findings, 4) presenting findings to key audiences, and 5) developing resources dedicated to bridging identified gaps.

### Results:

The immediate impact of this project was creation of the OI Adult Health Toolkit: Information for Adults Living with OI, Their Families, and Medical Professionals, to help meet identified gaps. Second, the project increased the commitment of the OIF to develop evidence-based data resources on adult OI healthcare.

### Conclusion:

The leadership team recognized that progress in improving care for adults with OI cannot be achieved unless progress is also made to support the greater rare disease community .

### P145

### Assessment of serum alkaline phosphatase, serum phosphate, rickets severity score, spinal stenosis, nephrocalcinosis and renal function with Burosumab or conventional therapy among patients with X-linked hypophosphatemia (XLH): Results from a multinational, long-term, prospective outcomes Disease Monitoring Program (DMP)

Leanne Ward[1], Kathryn Dahir[2], Hamilton Cassinelli[3], Pablo Florenzano[4], Erik Imel[5], Aliya Khan[6], Jill Simmons[7], Suzanne Jan de Beur[8], Carolina Moreira[9], Anna Ryabets-Lienhard[10], Zhiyi Li[11], Zunqiu Chen[12], Antonio Ramirez[12], Osman Ciğeroğlu[12], Thomas Carpenter[13]

[1]Children's Hospital of Eastern Ontario, Ottawa, Canada. [2]Vanderbilt University Medical Center, Nashville, USA [3]CEDIE, Centro de Investigaciones Endocrinologicas Dr Cesar Bergada, Division de Endocrinologia, Hospital de Niños Ricardo Gutierrez, Buenos Aires, Argentina. [4]Endocrinology Department, School of Medicine, Pontificia Universidad Católica de Chile, Santiago, Chile. [5]Indiana University School of Medicine, Indianapolis, USA [6]McMaster University, Ontario, Canada. [7]Vanderbilt University School of Medicine, Nashville, USA [8]University of Virginia, Charlottesville, USA [9]Endocrine Division (SEMPR), Department of Internal Medicine, Federal University of Parana, Curititba, Brazil. [10]Children's Hospital Los Angeles, Los Angeles, USA [11]Kyowa Kirin, Inc., Princeton, USA [12]Ultragenyx Pharmaceutical Inc., Novato, USA [13]Yale University School of Medicine, New Haven, USA

### Abstract

### Objectives:

To provide an update from the XLH DMP, an ongoing, non-interventional, prospective study with a 10-year planned duration, that characterizes disease progression and long-term effectiveness and safety of burosumab and other treatments (NCT03651505).

### Methods:

Children (<18 years) and adults (≥18 years) at DMP enrollment (Month 0; M0) were grouped by burosumab treatment: Group 1 (Gr1) began burosumab in a prior clinical trial and have the longest exposure among the study populations; Group 2 (Gr2) began commercial burosumab prior to/during the DMP; Group 3 (Gr3) had no burosumab exposure, but most received conventional therapy (CT; e.g., oral phosphate and active vitamin D). Results from patients who completed Year 3 (Y3) at data cutoff are reported.

### Results:

As of February 2023, 347 children and 461 adults were enrolled; mean (SD) age at M0 was 9.0 (4.8) and 39.0 (14.7) years, respectively. Mean duration of burosumab exposure was similar between children (133.1 [65.5] weeks) and adults (135.0 [72.0] weeks). At Y3, mean serum alkaline phosphatase was lower in children receiving burosumab. Mean serum phosphate was higher in children (Fig 1A) and adults (Fig 1B) receiving burosumab. Low baseline rickets severity scores (RSS) in Gr1 children persisted at Y3 and improved in Gr2 children; Gr3 patients had not yet completed RSS at Y3. At M0, 75 (16%) adults across groups reported an existing diagnosis of spinal stenosis (SS) or spinal cord compression (SCC), including 36 patients (37%) in Gr1, 28 patients (14%) in Gr2, and 11 patients (7%) in Gr3. Few patients with a previous diagnosis of SS or SCC reported worsening (20%–43%) or underwent a related surgery (0%–14%) by Y3. Comparing M0 to Y3, 4 patients (2 each from Gr2 and Gr3) had renal ultrasound score increases of ≥2, and 1 from Gr2 improved with score decreases of ≥2; no Gr1 patients had increases ≥2. Burosumab was not associated with declines in renal function, urinary protein/creatinine ratio, or new safety concerns.

**Conclusions:**

Long-term exposure to burosumab for up to 3 years in the DMP was consistent with its previously reported benefits vs CT and positively affected markers of disease activity.

### Disclosure

KD: Research funding to institution from Ultragenyx, Regeneron, Astra Zeneca Alexion and Kyowa Kirin, and consulting for Ultragenyx, Astra Zeneca Alexion and Kyowa Kirin. HC: Research Support: Ultragenyx Pharmaceutical Inc. PF: Research funding to institution from Ultragenyx. Consulting fees Kyowa Kirin EI: Research funding to institution from Ultragenyx and Kyowa Kirin, and consulting for Ultragenyx and Kyowa Kirin. AK: Grants and honoraria from Alexion, Amgen, Ascendis, Chugai, Radius, Takeda, and Ultragenyx JS: Research funding to institution from Ultragenyx and consulting to Ultragenyx LW: Consultant to and participant in clinical trials with Ultragenyx Pharmaceutical Inc. (with funds to Dr. Ward's institution, the Children's Hospital of Eastern Ontario Research Institute) SJdB: Ultragenyx research investigator, scientific advisory board, consultant, Kyowa Kirin scientific advisory board, consultant Inozyme scientific advisory board, Mereo research investigato CM: Research Support: Ultragenyx Pharmaceutical Inc. AR-L: Research support/site PI: Ultragenyx Pharmaceutical Inc., Amgen, Ascendis, Takeda. Consultant/Advisory board: BioMarin, Ascendis, BridgeBio. ZL: Employee: Kyowa Kirin, Inc. ZC: Employee, Shareholder: Ultragenyx Pharmaceutical, Inc. AR: Employee, Shareholder: Ultragenyx Pharmaceutical, Inc. TC: Research Investigator (funds to institution): Ultragenyx; Advisory Board and consulting: Ultragenyx Pharmaceutical Inc, Inozyme,Kyowa Kirin; Consulting: Viridian

### P146

### Disease burden in hypophosphatasia: Quality of life, pain, disability and mobility in patients by number of *ALPL* variants in the global HPP registry

Lothar Seefried[1], Kathryn Dahir[2], Gabriel Martos-Moreno[3], Keiichi Ozono[4], Wolfgang Högler[5], Cheryl Rockman-Greenberg[6], Shona Fang[7], Anna Petryk[7], William Mowrey[7], Priya Kishnani[8], Agnès Linglart[9]

[1]University of Würzburg, Würzburg, Germany. [2]Vanderbilt University Medical Center, Nashville, USA [3]Hospital Infantil Universitario Niño Jesús, IIS La Princesa, Universidad Autónoma de Madrid, CIBERobn, ISCIII, Madrid, Spain. [4]ISEIKAI International General Hospital , Suita, Osaka, Japan. [5]Johannes Kepler University Linz, Linz, Austria. [6]University of Manitoba, Winnipeg, Manitoba, Canada. [7]Alexion, AstraZeneca Rare Disease, Boston, USA [8]Duke University Medical Center, Durham, USA [9]Paris-Saclay University, AP-HP and INSERM, Paris, France

### Abstract

**Objective:** Hypophosphatasia (HPP) is a rare, inherited metabolic disease caused by usually detectable *ALPL* gene variants and is associated with compromised bone mineralization, fractures/pseudofractures, muscle weakness, pain, impaired mobility, and reduced health-related quality of life. This analysis compared measures of disease burden between patients with HPP (excluding perinatal/infantile HPP) with 1 versus ≥2 *ALPL* variants.

**Methods:** Patients in the Global HPP Registry (NCT02306720) with HPP manifesting after age 6 months and ≥1 *ALPL* variant classified as pathogenic, likely pathogenic, or of uncertain significance were included. The Pediatric Quality of Life Inventory (PedsQL; age 2-<18 years), 36-item Short-Form Health Survey, version 2 (SF-36v2; age ≥18 years), Brief Pain Inventory-Short Form (BPI-SF; ≥18 years), Health Assessment Questionnaire-Disability Index (HAQ-DI; ≥18 years), and 6-Minute Walk Test (6MWT; 5-<18 years and ≥18 years) were compared between patients with 1 versus ≥2 *ALPL* variants. The most recently collected assessments were analyzed (before starting asfotase alfa in treated patients). Data cutoff: 30 June 2023.

**Results:** Of 685 eligible patients, 568 (83%) had 1 *ALPL* variant and 117 (17%) had ≥2 variants. Among patients with 1/≥2 variants, 70%/48% were age ≥18 years, 67%/56% were female, 77%/62% were White, and 6%/20% were Asian. Outcomes are summarized by variant number (**Table**). In children, median PedsQL scores were comparable between groups with no apparent effect of variant number. In adults, median SF-36v2 scores were not statistically different between groups, although the physical component, physical functioning, and bodily pain scores trended lower in adults with ≥2 versus 1 variant. BPI-SF and HAQ-DI scores were comparable between groups. Median 6MWT percent predicted distances walked did not differ by variant number in children, but were greater in adults with 1 (475 m, 87%) versus ≥2 (288 m, 54%) variants.

**Conclusions:** Patients with HPP had predominately similar disease burden regardless of *ALPL* variant count. Adults with ≥2 variants appeared to have greater mobility impairment than those with 1 variant, although interpretability of the 6MWT data is limited by the small number of adults with ≥2 variants.

**Table**

| **Outcome** | **1 *ALPL* variant** | **≥2 *ALPL* variants** | **Difference in means (95% CI)** |
|---|---|---|---|
| PedsQL,[a] median (Q1, Q3) | (n=116) | (n=31) | |
| Total | 84.7 (69.0, 93.0) | 84.8 (70.5, 94.6) | 0.6 (−6.4, 7.7) |
| Physical functioning | 89.1 (70.1, 100) | 87.5 (65.6, 96.9) | −1.4 (−10.0, 7.3) |
| Psychosocial functioning | 83.3 (61.7, 92.3) | 83.3 (71.7, 95.0) | 1.4 (−5.5, 8.2) |
| Emotional functioning | 80.0 (60.0, 95.0) | 80.0 (60.0, 95.0) | 0.4 (−7.5, 8.3) |
| Social functioning | 90.0 (70.0, 100) | 95.0 (75.0, 100) | 1.9 (−6.0, 9.7) |
| School functioning | 85.0 (65.0, 100) | 85.0 (65.0, 95.0) | 1.5 (−6.8, 9.9) |
| SF-36v2,[b] median (Q1, Q3) | (n=305) | (n=25) | |
| Physical component | 43.9 (34.8, 53.2) | 35.4 (30.5, 49.7) | −4.5 (−9.0, 0.1) |
| Mental component | 45.5 (36.1, 53.5) | 43.8 (34.4, 53.6) | −0.3 (−5.0, 4.3) |
| Vitality | 43.7 (34.8, 52.6) | 40.7 (34.8, 52.6) | −1.4 (−5.9, 3.2) |
| Physical functioning | 46.1 (36.5, 53.7) | 38.4 (30.8, 51.8) | −3.7 (−8.0, 0.7) |
| Bodily pain | 42.2 (34.2, 51.5) | 38.2 (30.6, 46.3) | −4.1 (−8.6, 0.5) |
| General health perceptions | 43.7 (34.2, 50.8) | 40.4 (26.1, 50.8) | −3.8 (−8.4, 0.8) |
| Physical role functioning | 41.4 (32.5, 54.9) | 37.0 (30.2, 50.4) | −3.9 (−8.6, 0.8) |
| Emotional role functioning | 45.7 (35.3, 56.2) | 38.8 (31.8, 52.7) | −2.6 (−7.6, 2.5) |
| Social role functioning | 42.3 (32.3, 52.3) | 42.3 (32.3, 52.3) | −0.6 (−5.4, 4.2) |
| Mental health | 45.6 (37.8, 53.5) | 48.3 (32.6, 53.5) | −1.0 (−5.5, 3.4) |
| BPI-SF[c] | (n=319) | (n=27) | |
| Pain severity, median (Q1, Q3) | 3.3 (1.0, 5.5) | 4.3 (1.3, 6.5) | 1.0 (−0.3, 2.0) |
| Age-adjusted LS mean | 3.4 | 4.4 | |
| Pain interference, median (Q1, Q3) | 2.7 (0.3, 5.7) | 3.3 (1.1, 6.3) | 0.6 (−0.3, 1.7) |
| Age-adjusted LS mean | 3.3 | 4.0 | |
| HAQ-DI[d] | (n=307) | (n=24) | |
| Median (Q1, Q3) | 0.38 (0.00, 1.00) | 0.28 (0.00, 1.31) | 0.00 (−0.13, 0.13) |
| Age-adjusted LS mean | 0.57 | 0.70 | |
| 6MWT in children | (n=36) | (n=13) | |
| Meters walked, median (min, max) [Q1, Q3] | 490.0 (312.0, 747.0) [428.0, 589.0] | 513.0 (320.0, 745.0) [431.0, 600.0] | 11.1 (−59.2, 81.3) |
| Age-adjusted LS mean | 508.4 | 502.4 | |
| Percent predicted,[e] median (min, max) [Q1, Q3] | (n=31)<br><br>74.5 (56.5, 111.5) [66.5, 93.3] | (n=12)<br><br>79.5 (56.4, 108.0) [70.3, 87.7] | 1.2 (−9.5, 11.8) |
| 6MWT in adults | (n=103) | (n=8) | |
| Meters walked, median (min, max) [Q1, Q3] | 475.0 (145.0, 740.0) [402.0, 540.0] | 288.0 (24.0, 540.0) [240.5, 362.5] | −172.3 (−259.5, −85.2)* |
| Age-adjusted LS mean | 463.1 | 304.2 | |
| Percent predicted,[e] median (min, max) [Q1, Q3] | (n=87) | (n=7) | −29.4 (−47.8, −10.9)* |

| | | | |
|---|---|---|---|
| | 87.0 (27.3, 131.6) [67.0, 101.1] | 54.1 (4.2, 101.3) [42.4, 67.1] | |

CI, confidence interval; HRQOL, health-related quality of life; LS, least squares.

*Difference between groups was statistically significant based on the 95% CI for the mean difference not including 0.

[a]PedsQL is scored on a 0–100 scale; higher scores indicate better HRQOL.

[b]SF36v2 is scored on a 0–100 scale; higher scores indicate more favorable health state.

[c]BPI-SF is scored on a 0–10 scale; lower scores indicate less pain or less interference of pain.

[d]HAQ-DI is scored on a 0–3 scale; lower scores indicate less disability.

[e]Normal range for percent predicted distance walked on the 6MWT: 80% to 120%.

### Disclosure

LS, KMD, GÁMM, KO, WH, CRG, PSK, and AL have consulted for and/or received research funding/honoraria from Alexion, AstraZeneca Rare Disease. SF, WRM, and AP are employees of and may own stock/options in Alexion, AstraZeneca Rare Disease.

### P147

### Novel biallelic PISD missense variants cause spondyloepimetaphyseal dysplasia with disproportionate short stature and fragmented mitochondrial morphology

Line Nolting[1], Tess Holling[2], Gen Nishimura[3], Jakob Ek[4], Mads Bak[4], Merete Ljungberg[1], Kerstin Kutsche[5], Hanne Hove[6]

[1]Department of Pediatrics and Adolescents, Copenhagen University Hospital, Rigshospitalet, Copenhagen, Denmark. [2]Institute of Human Genetics, University Medical Center Hamburg-Eppendorf, Hamborg, Germany. [3]Center for Intractable Diseases, Saitama Medical University Hospital, Saitama, Japan. [4]Department of Clinical Genetics, Copenhagen University Hospital, Rigshospitalet, Copenhagen, Denmark. [5]Institute of Human Genetics, University Medical Center Hamburg-Eppendorf, Hamburg, Germany. [6]Department of Pediatrics and Adolescents, Copenhagen University Hospital, Copenhagen, Denmark

### Abstract

**Background/Objectives:** Biallelic variants in the *PISD* gene are associated with a phenotypic spectrum ranging from short stature with spondyloepimetaphyseal dysplasia to a complex multisystem disorder impacting the eye, ear, bone, and brain. *PISD* encodes the mitochondrial-localized enzyme phosphatidylserine decarboxylase. PISD is produced as a proenzyme that is self-cleaved to generate a heteromeric mature enzyme, which converts phosphatidylserine to the phospholipid phosphatidylethanolamine. Herein, we present a patient with novel biallelic *PISD* missense variants.

**Methods:** Comprehensive clinical, genomic, and functional investigations, including immunoblotting and immunofluorescence analyses, were conducted using fibroblasts derived from the patient, his parents, and control subjects.

**Results:** The 17-year-old male patient, born to unrelated healthy parents, presented with distinctive skeletal features, including disproportionate short stature and spondyloepimetaphyseal dysplasia characterized by platyspondyly, prominent epiphyses, and metaphyseal dysplasia, but no extraskeletal features Trio genome sequencing revealed compound heterozygous *PISD* variants: NM_001326411.1:c.569C>T; p.(Ser190Leu) and c.799C>T; p.(His267Tyr). Analysis of fibroblasts revealed comparable levels of the PISD precursor in patient and control cell lines, however, patient cells exhibited a significantly higher proportion of fragmented mitochondria compared to control cells, cultured under basal conditions and following treatment with 2-deoxyglucose, a glycolysis-repressing agent that stimulates respiration. Insights derived from the published structure of the *E. coli* PISD orthologue indicated that Ser190Leu and His267Tyr likely impair PISD's autoprocessing activity and/or phosphatidylethanolamine biosynthesis.

**Conclusion:** Our findings demonstrate that the novel *PISD* p.(Ser190Leu) and p.(His267Tyr) variants function as hypomorphs and underlie the pure skeletal phenotype observed in the patient. Recent data highlighting the subcellular targeting of PISD to the endoplasmic reticulum and lipid droplets, coupled with its involvement in lipid droplet biogenesis and neutral lipid storage, suggest diverse functional consequences underlying the pleiotropic phenotypes associated with distinct biallelic *PISD* pathogenic variants.

### P148

### Assessments of bone mineralisation, strength and fracture of the lumbar spine in patients with fibrodysplasia ossificans progressiva treated with palovarotene: Data from the phase III MOVE trial and a phase II open-label extension

Richard Keen[1], Robert J Pignolo[2], Mona Al Mukaddam[3], Geneviève Baujat[4], Angela M Cheung[5], Carmen De Cunto[6], Edward C Hsiao[7], Jean-Luc Delhay[8], Fei Shih[9], Andrew Strahs[9], Frederick S Kaplan[3]

[1]Centre for Metabolic Bone Disease, Royal National Orthopaedic Hospital, Stanmore, UK [2]Department of Medicine, Mayo Clinic, Rochester, MN, USA [3]Departments of Orthopaedic Surgery and Medicine, Center for Research in FOP and Related Disorders, Perelman School of Medicine, University of Pennsylvania, Philadelphia, PA, USA [4]Département de Génétique, Hôpital Universitaire Necker-Enfants Malades, Institut Imagine, Université Paris Cité, Paris, France. [5]Department of Medicine and Joint Department of Medical Imaging, University Health Network, University of Toronto, Toronto, ON, Canada. [6]Pediatric Rheumatology Section, Department of Pediatrics, Hospital Italiano de Buenos Aires, Buenos Aires, Argentina. [7]Division of Endocrinology and Metabolism, the UCSF Metabolic Bone Clinic, the Eli and Edyth Broad Institute for Regeneration Medicine, and the Institute of Human Genetics, Department of Medicine, and the UCSF Program in Craniofacial Biology, University of California San Francisco, San Francisco, CA, USA [8]Ipsen, Les Ulis, France. [9]Ipsen, Cambridge, MA, USA

### Abstract

**OBJECTIVES:** Fibrodysplasia ossificans progressiva (FOP) is an ultra-rare genetic disorder characterised by heterotopic ossification (HO) that manifests in childhood. Palovarotene, a selective retinoic acid receptor-γ agonist, is approved for reduction of new HO in patients with FOP aged ≥8 (females) and ≥10 (males) years, although decreased bone mineral density (BMD) and vertebral fracture risks have been identified.[1] Here, post hoc computational analyses on computed tomography (CT) scans were used to evaluate bone mineral content (BMC), BMD and strength of normotopic bone in patients receiving palovarotene in the pivotal phase III MOVE trial (NCT03312634) and phase II open-label extension (OLE; NCT02279095).

**METHODS:** Patients received palovarotene for ≤48 months in MOVE and ≤60 months in the phase II OLE. Using finite element analysis, biomechanical CT (VirtuOst® software) was performed on low-dose whole-body CT scans to assess spinal (L1, L2 or adjacent vertebrae) BMC, BMD and bone strength annually since baseline. Results were adjusted for baseline characteristics and compared with patients untreated beyond standard of care from an FOP natural history study (NHS; NCT02322255).

**RESULTS:** 107 patients received palovarotene in MOVE (mean [SD] age: 15.5 [10.1] years; follow-up: 32.7 months) and 54 in the phase II OLE (mean [SD] age: 21.0 [9.2] years; follow-up 42.7 months); 114 patients participated in the NHS (mean [SD] age: 17.6 [9.7] years; follow-up: 25.9 months).

Changes in mid-vertebral density, entire vertebral BMC and vertebral body strength for patients receiving palovarotene in MOVE and the phase II OLE compared with untreated patients are presented in the **Table**. In MOVE, patients receiving palovarotene had a 2.29 times higher risk of radiographically observed annualised new-onset vertebral fractures versus untreated patients (p=0.009); a significantly higher risk was not observed in the phase II OLE.

**CONCLUSION:** Palovarotene generally did not impact computed spinal BMC, BMD or bone strength in patients across both trials, except for a decrease in BMC in the phase II OLE. Radiographically observed vertebral fracture risk was higher with palovarotene in MOVE. The long-term clinical significance of these findings requires further investigation.

**References: 1.** Pignolo RJ. J Bone Miner Res. 2023;38(3):381–94.

**Table.** Change in mid-vertebral density, entire vertebral BMC and vertebral body strength for patients receiving palovarotene in MOVE and the phase II OLE compared with untreated patients from the NHS

| | Mid-vertebral (trabecular) density, mg/cm³ | Entire vertebral body BMC, g | Vertebral body strength, newtons |
|---|---|---|---|
| MOVE[a] | −0.55 | −0.01 | 1.23 |
| p-value | p=0.538 | p=0.812 | p=0.978 |
| Phase II OLE[b] | −0.04 | −0.15 | −180.18 |
| p-value | p=0.964 | p=0.001[c] | p=0.056 |

[a]BCT measurements were taken from 85 patients receiving palovarotene in MOVE and 85 untreated patients in the NHS; [b]BCT measurements were taken from 45 patients receiving palovarotene in the phase II OLE and 85 untreated patients in the NHS; [c]$p<0.05$. Characteristics adjusted for included: baseline CAJIS score, age-adjusted HO at baseline, sex, age at FOP onset and steroid use. BCT: biomechanical computed tomography; BMC: bone mineral content; CAJIS: cumulative analogue joint involvement scale; FOP: fibrodysplasia ossificans progressiva; HO: heterotopic ossification; NHS: natural history study; OLE: open-label extension.

### Disclosure

Funding: This study was sponsored by Ipsen. Disclosures: MAM: Research Investigator: Clementia/Ipsen, Incyte, Regeneron; Non-paid consultant: Biocryst, Blueprint, Daiichi Sankyo, Incyte, Keros; Advisory Board: IFOPA Registry Medical Advisory Board, International Clinical Council on FOP (all voluntary); GB: Research Investigator: Alexion, BioMarin, Clementia/Ipsen, Incyte; Advisory Board: Clementia/Ipsen; Speaker: Clementia/Ipsen; FOP European Consortium, International Clinical Council on FOP, IFOPA Registry Medical Advisory Board (all voluntary); AMC: Research Investigator: Clementia/Ipsen, Incyte, Regeneron; Consultant: Ipsen; CDC: Research Investigator: Clementia/Ipsen; Member of the International Clinical Council on FOP; ECH: Research support: Ascendis, Clementia/Ipsen, Ultragenyx; Research Investigator: Ascendis, Clementia/Ipsen; Member of the International Clinical Council on FOP, Fibrous Dysplasia Foundation, IFOPA Registry Advisory Board (all voluntary); RK: Research Investigator: Clementia/Ipsen, Kyowa Kirin, Regeneron; Advisory Board: IFOPA Registry Medical Advisory Board, International Clinical Council on FOP; RJP: Research Investigator: Clementia/Ipsen, Incyte, Regeneron; Advisory Board: Immediate Past President of the International Clinical Council on FOP; JLD, FS, AS: Employees of Ipsen; FSK: Research Investigator: Clementia/Ipsen, Incyte, Regeneron; Advisory Board: IFOPA Medical Advisory Board; Founder and Past President of the International Clinical Council on FOP.

### P149

### Children with X-linked hypophosphataemia with higher baseline serum phosphate or TmP/GFR require lower doses of burosumab after two years

Jessica Sandy[1,2], Alessandra Cocca[3], Robyn Gilbey-Cross[3], Sophia Sakka[4], Rui Santos[3], Moira Cheung[5]

[1]University of Sydney, Sydney, Australia. [2]The Children's Hospital at Westmead, Westmead, Australia. [3]The Evelina London Children's Hospital, London, UK [4]Athens University Medical School, "Aghia Sophia" Children's Hospital, Athens, Greece. [5]Great Ormand Street Hospital, London, UK

### Abstract

**Background:** The phenotype of X-linked hypophosphaetemia (XLH) can be mild to severe. Burosumab, a monoclonal antibody against FGF23, is now widely used in XLH but guidelines on starting dose are mixed, with two main recommendations: 0.8mg/kg[1] or 0.4mg/kg[2]. Dose titration is based on trough fasting serum phosphate. The long-term effect of over-treatment is unknown. Currently, there no recognised markers that help predict who will require higher or lower doses.

**Aims:**

1. Describe the dose of burosumab needed at baseline (BLD) and after 2 years of titration (titrated dose, TD).
2. Describe characteristics of those needing doses <0.8mg/kg at TD.
3. Suggest possible predictors of TD to guide management.

**Methods:**

Retrospective review of clinical data in all children with XLH treated at a specialist children's hospital who had completed 2 years (+/- 3 months) of burosumab therapy. Washout period of all medications was ≥2 weeks prior to baseline biochemistry. Information on TmP/GFR, phosphate and alkaline phosphatase (ALP) z scores, height z scores were collected.

Baseline characteristics of children whose TD ≥0.8mg/kg versus <0.8mg/kg were compared (t-test). Correlation between dose at TD and biochemical markers of the whole population was evaluated (Pearson).

**Results:**

A summary of the results is shown in the table. Mean±SD BLD was 0.63±0.21 and TD 1.03±0.51.

Patients who required TD <0.8mg/kg (versus TD≥0.8mg/kg) had significantly higher baseline serum phosphate and TmP/GFR. There was negative linear correlation seen between TD and baseline serum phosphate ($r$ =-0.60,p=0.040); TmP/GFR ($r$ = -0.61,p= 0.004); phosphate z scores ($r$ = -0.55,p=0.024) and TmP/GFR % LLN ($r$ = -0.59,p=0.009). There was no correlation seen between TD and baseline ALP z scores or radiological rickets scores between the two groups.

TD was lower in individuals with baseline serum phosphate ≥ 0.8mmol/L (0.66±0.29 versus 1.27±0.46mg/kg ), phosphate z scores ≥ -2.0 (0.54±0.03 versus 1.13±0.49mg/kg), TmP/GFR ≥ 0.70mmol/L (0.57±0.17 versus 1.17±0.48mg/kg) and TmP/GFR ≥ 70% LLN (0.57±0.17 versus 1.17±0.48mg/kg), p values<0.05.

**Conclusion:**

Children with XLH who have higher baseline phosphates ( ≥ 0.8mmol/L, z scores ≥ -2.0) and/or TmP/GFR (≥ 0.70mmol/L, ≥ 70% LLN), require lower burosumab doses after 2 years. Clinicians may consider this when deciding on starting dose of burosumab.

*Table: Baseline characteristics of cohort of children with XLH and comparison of those requiring higher versus lower burosumab doses*

| | Overall<br><br>N =19 | Burosumab dose[a] < 0.8mg/kg<br><br>N= 8 | Burosumab dose[a] ³ >/= 0.8mg/kg<br><br>N= 11 | P value |
|---|---|---|---|---|
| % Female | 44% | 50% | 40% | - |
| Age when commencing burosumab (Mean±SD) | 7.7±4.8 | 8.4±4.6 | 7.1±5.2 | 0.279 |
| Burosumab dose at baseline (Mean±SD) | 0.63±0.21 | 0.60±0.14 | 0.66±0.25 | 0.290 |
| Titrated 24m burosumab dose[a] (Mean±SD) | 1.03±0.51 | 0.60±0.15 | 1.38±0.42 | 0.000* |
| ALP z score baseline[c] (Mean±SD) | +4.39±2.84 | +5.33±3.99 | +3.64±1.22 | 0.111 |
| ALP z score 24m (Mean±SD) | +0.99±1.78 | +1.15±2.00 | +0.84±1.66 | 0.371 |
| **Serum phosphate baseline[c] (Mean±SD)** | **0.72±0.13** | **0.80±0.13** | **0.66±0.10** | **0.009*** |
| **Serum phosphate z score baseline[c] (Mean±SD)** | **-2.86±0.74** | **-2.40±0.44** | **-3.22±0.74** | **0.016*** |
| Serum phosphate 24m (Mean±SD) | 1.12±0.14 | 1.12±0.17 | 1.12±0.12 | 0.496 |
| Serum phosphate z score 24m (Mean±SD) | -1.18±0.76 | -1.19±0.98 | -1.18±0.51 | 0.495 |
| Height z score baseline<br><br>(Mean±SD) | -1.97±1.06 | -1.52±1.09 | -2.32±0.94 | 0.056 |
| **TmP/GFR baseline[c]**<br><br>**(Mean±SD)** | **0.62±0.14** | **0.70±0.14** | **0.56±0.12** | **0.016*** |
| **TmP/GFR baseline[c] (% of lower limit of normal for age and sex)[f]**<br><br>**(Mean±SD)** | **63±16%** | **71±17%** | **56±12%** | **0.025*** |
| Thacher Rickets Severity Score baseline (Mean±SD) | 1.25±1.73 | 1.00±1.31 | 1.45±2.05 | 0.299 |
| Thacher Rickets Severity Score 24m (Mean±SD) | 0.00±0.00 | 0.05±0.16 | 0.05±0.16 | 0.194 |

[a]Burosumab dose at 24 months titrated based on serum fasting phosphate levels

[c]Biochemistry performed while fasting prior to commencement of burosumab after at least 2 weeks off all treatments (including oral phosphate and calcitriol).

[f]Reference range and TmP/GFR calculator available at: https://gpn.de/service/tmp-gfr-calculator

*Statistically significant p value

References:

1. J. L. Sandy, P. J. Simm, A. Biggin, C. P. Rodda, C. L. Wall, A. Siafarikas, et al. Clinical practice guidelines for paediatric X-linked hypophosphataemia in the era of burosumab. J Paediatr Child Health. 2022;58(5):762-8.

2. R. Padidela, M. S. Cheung, V. Saraff, P. Dharmaraj. Clinical guidelines for burosumab in the treatment of XLH in children and adolescents: British paediatric and adolescent bone group recommendations. Endocr Connect. 2020;9(10):1051-6.

### Disclosure

Dr Cheung has received honoraria for advisory boards and delivering presentations from Kyowa Kirin Dr Sandy has been involved in investigator led research funded by Kyowa Kirin.

### P150

### Natural history of spinal cord compression stage AFMS3 in infants with achondroplasia: Retrospective cohort study

Moira Cheung[1], Alessandra Cocca[2], Amaka Offiah[3], Stephanie Borg[4], Charlotte Harvey[5], Connor Brett[5], Nathan Jenko[6], Felice D'Arco[1], Dominic Thompson[1]

[1]Great Ormond Street Hospital, London, UK [2]Evelina London Children's Hospital, London, UK [3]Sheffield Children's Hospital NHS Foundation Trust, Sheffield, UK [4]Sheffield Children's NHS Foundation Trust, Sheffield, UK [5]Evelina London Children's Hospital, London, UK [6]Sheffield Children's NHS Trust, Sheffield, UK

### Abstract

Foramen magnum stenosis (FMS) is a common, serious complication of achondroplasia in infancy and associated with sudden infant death. The Achondroplasia Foramen Magnum Score (AFMS; 0-4) is used to classify the severity of stenosis to inform appropriate neurosurgical management. Infants with AFMS4 are referred for neurosurgery, whilst well children with AFMS3 undergo repeat MRI routinely after 12 months.

As the natural history of children with AFMS3 is currently unclear, the objective was to review follow-up MRI scans of infants initially classified as AFMS3 to define more clearly the evolution of this degree of stenosis.

This retrospective cohort study, from two tertiary centres, included infants with a confirmed diagnosis of achondroplasia and AFMS3 on initial MRI who subsequently underwent repeat MRI or proceeded straight to neurosurgery.

22 cases satisfied the inclusion criteria. Mean age in months was 6.23 (SD±3.82) and 17.95 (SD±7.68) at baseline and follow-up scans, respectively. Follow-up MRI showed no change in 22.7% (N=5), improvement in 36.2% (N=8) to either AFMS1 (N=5) or AFMS2 (N=3). There was progression in 40.9% to AFMS4 (N=8) or had neurosurgey without a repeat (N=1).

These results support MRI screening for FMS in infants with achondroplasia. Furthermore, infants with AFMS3 should undergo follow-up MRI as over 40% progress prompting neurosurgical intervention. There is currently no consensus on frequency or timing of screening for FMS in achondroplasia; however, we suggest that guidance for follow-up imaging for AFMS3 is modified to six months to detect progression earlier in this at-risk cohort.

### P151

### Does Vosoritide treatment affect bone strength in children with achondroplasia?

Chloe Derocher[1], Erin Carter[1], Karl Jepsen[2], Cathleen Raggio[1]

[1]Hospital for Special Surgery, New York, USA [2]University of Michigan, Ann Arbor, USA

### Abstract

**OBJECTIVES:**

Achondroplasia is an autosomal dominant disorder affecting endochondral bone formation, commonly characterized by disproportionate short stature. The objective of this study was to determine whether vosoritide, an FDA-approved drug meant to increase endochondral bone linear growth, affects both length and development of bone strength in children with achondroplasia using measurements of the second metacarpal.

**METHODS:**

This study included 103 deidentified AP hand/wrist radiographs from 30 children with achondroplasia (13M, 17F; ages 7.8-16 years). Proprietary data included hand films collected at four time points: week 104 (at rollover into the phase II extension study), week 156, week 208 and week 260 on treatment. Second metacarpal length and midshaft width, cortical thickness, robustness (total area/length), and cortical area (correlated with strength) were measured. Measurements were compared to 378 radiographs from 114 average-stature controls (61M, 53F; ages 6-16 years) via non-parametric Kruskal-Wallis tests ($p<0.05$).

**RESULTS:**

Children with achondroplasia at week 208 and week 260 of treatment demonstrated longer metacarpals with increased cortical area and cortical thickness compared to week 104 (all $p<0.05$). There was no significant change in RCA and robustness across the treatment time points (Table 1). Furthermore, no differences were seen between males and females with achondroplasia at any of the timepoints. Compared to the controls, children with achondroplasia had higher robustness and cortical area throughout treatment ($p<0.001$).

**CONCLUSIONS:**

We observed that an additional 2-3 years of vosoritide treatment was associated with increases in bone length compared to the initial timepoint (week 104), as well as increases in metacarpal cortical area, which is correlated with strength. This study suggested this bone lengthening treatment did not adversely affect bone strength in children with achondroplasia. The lack of a significant difference in robustness after treatment indicated that periosteal expansion continued outward at a pace which maintains robustness, allowing the bone to remain strong as it lengthened. Future work comparing treated and untreated children with achondroplasia is necessary to more rigorously confirm whether the treatment did not adversely affect the development of bone strength. Overall, this work may have important clinical implications in terms of treatment choices for children with achondroplasia.

**Table 1.** Measurements of the second metacarpal (mean±SD) at each time point in treatment.

| | Baseline (n=29) | Week 52 (n=29) | Week 104 (n=28) | Week 156 (n=17) | Controls (n=378) |
|---|---|---|---|---|---|
| **Le (mm)** | 39.75±4.49‡^° | 42.13 ± 4.36° | 44.12±4.56*° | 45.00±4.98*° | 56.83±9.40*#‡^ |
| **T.Ar ($mm^2$)** | 42.84±11.82^ | 45.79±12.49° | 49.80±14.63° | 54.29±17.64*° | 39.01±11.79#‡^ |
| **Ct.Ar ($mm^2$)** | 25.60±6.59‡^° | 28.12±6.72^ | 32.06±7.93* | 35.60±9.59*#° | 29.64±9.57*^ |
| **Marrow Area ($mm^2$)** | 17.24±9.38° | 17.67±10.54° | 17.73±12.15° | 18.68±12.06° | 9.37±4.81*#‡^ |
| **Cortical Thickness (mm)** | 1.39±0.35‡^° | 1.49±0.37° | 1.67±0.43*° | 1.77±0.41*° | 1.10±0.35*#‡^ |
| **RCA** | 0.61±0.12° | 0.63±0.12° | 0.66±0.12° | 0.67±0.12° | 0.76±0.10*#‡^ |
| **T.Ar/Le (mm)** | 1.08±0.30° | 1.09±0.29° | 1.13±0.32° | 1.20±0.37° | 0.68±0.13*#‡^ |

*significantly different (p<0.05) than baseline, #significantly different (p<0.05) than w52, ‡significantly different (p<0.05) than w104, ^significantly different (p<0.05) than w156, °significantly different (p<0.05) than controls°

### Disclosure

Dr. Cathleen Raggio; BioMarin - speaker bureau, consultant, grants.

### P152

### Chiari 1 malformations and craniosynostosis in X-Linked hypophosphatemia

Chelsey Grimbly[1,2], Jacob L Jaremko[1], Serdar Kilicarslan[3], Rose Girgis[1], Elizabeth Rosolowsky[1], Manpreet Doulla[1], Nasrin Khan[4], Leanne M Ward[5], R Todd Alexander[1,2]

[1]University of Alberta, Edmonton, Canada. [2]Women and Children's Health Research Institute, Edmonton, Canada. [3]Children's Hospital of Eastern Ontario Research Institute, Ottawa, Canada. [4]Children's Hospital of Eastern Ontario Research Institute, Ottawa, Canada. [5]Children's Hospital of Eastern Ontario, Ottawa, Canada

### Abstract

**Background**: X-linked hypophosphatemia (XLH) is a genetic form of rickets that has neurodevelopmental complications including craniosynostosis, Chiari 1 malformations (CM1) and syringomyelia. CM1 can present with acute and potentially permanent neurologic deficits including increased intracranial pressure, papilledema, or nerve radiculopathy. The risk factors for CM1 are unclear, including whether there is a relationship with craniosynostosis.

**Objectives:** To prospectively evaluate the rates of CM1, craniosynostosis and syringomyelia in children and adults with XLH, and to assess if craniosynostosis is a risk factor for CM1.

**Methods**: We performed a cross-sectional study evaluating imaging of individuals with XLH followed at the University of Alberta and Children's Hospital of Eastern Ontario. Patients aged 6 years and above were eligible for brain MRI, skull CT and/or spine MRI. Chiari 1 malformation was defined as cerebellar protrusion ≥5 mm below the foramen magnum measured on MRI. Craniosynostosis was defined as loss of visible sutures on CT. Syringomyelia was visualized on spine MR. Descriptive statistical analysis was performed.

**Results**: 27 individuals were enrolled (median 16.5 years of age, range 6-69, 70% female). Five participants were on Burosumab, 17 on conventional therapy, and five had a history of conventional therapy but were not on treatment at the time of imaging. Participants underwent brain MRI (N=26), skull CT (N=26), and/or spine MRI (N=22). CM1 was observed in 5/26 individuals (19%), of whom 1/5 (20%) required cranial vault remodeling. Craniosynostosis was observed in 11/26 (42%) but was absent in 2/4 individuals with CM1 (50%). Headaches were present in only 1/5 individuals with CM1 (20%), and in 6/11 (55%) with craniosynostosis. 2/22 individuals (89%) had syringomyelia in the absence of CM1 or craniosynostosis. One participant with CM1 had evidence of papilledema, requiring urgent neurosurgery referral.

**Conclusion:** One fifth of our cohort with XLH had CM1 and almost 50% demonstrated craniosynostosis. Both diagnoses were frequently asymptomatic, suggesting the need for routine surveillance for their detection. These data also provide proof of principle that the CM1 can occur in the absence of craniosynostosis, suggesting factors other than skull fusion in the development of this potentially serious complication.

### Disclosure

Dr. Ward declares consultancy to Ultragenyx and Kyowa Kirin, and participation in clinical trials with Kyowa Kirin, with funds to Dr. Ward's institution. Dr. Grimbly and Dr. Alexander declare consultancy with Ultragenyx and Kyowa Kirin.

### P153

### The importance of early bone marrow transplantation in protecting eyesight in osteopetrosis

Jeremy Allgrove

Great Ormond Street Hospital, London, UK

### Abstract

### BACKGROUND

The osteopetroses are a group of rare conditions characterised by increased bone densisty but, in most cases, increased bone fragility. Most severe forms are autosomal recessive and are associated with bone overgrowth. This includes narrowing of the optic, and to a lesser extent, auditory canals leading to nerve compression and optic nerve damage which is usually irreversible. The only effective cure is Bone Marrow Transplantation (BMT) which can only be used in certain cases.

### OBJECTIVE

To review the patients with osteopetrosis and compare the age at BMT with current eyesight, particularly with regard to their ability to function in normal school.

### METHODS

The records of the patiets attending the Osteopetrosis clinic at Great Ormond Street were reviewed. Only those who had diagnoses that are deemed eligible for BMT were included. These diagnoses were: OPTB1 (TCIRG1), OPTB7 (TNFSF11) and OLEDAID (IKBKG).

### RESULTS

These are summarised in the Table.

| Age at Transplant | Vision R | Vision L |
|---|---|---|
| **TCIRG1** | ↓ | ↓ |
| 1½ | N | N |
| 2 | ↓ | ↓ |
| 3 | ↓ | ↓ |
| 3½ | N | N |
| 5 | ↓ | ↓ |
| 5 | ↓ | ↓ |
| 6 | ↓ | ↓ |
| 6 | ↓ | ↓ |
| 11 | ↓ | ↓ |
| 11 | ↓ | ↓ |

| | | |
|---|---|---|
| N/D | ↓ | ↓ |
| N/D | ↓ | ↓ |
| **TNFSF11** | ↓ | ↓ |
| 10 | ↓ | ↓ |
| 54 | N | N |
| 100 | | |
| **IKBKG** | | |
| 3 | | |

Table comparing age at transplant (months) with current eyesight. ‘N’ indicates that eyesight is sufficiently good for them to be in normal school. All other patients are registered blind although they may retain some light perception.

There were twelve patients with OPTB1, two of whom had not been transplanted. There were three patients with OPTB7 all of whom had had late transplants and one with OLEDAID who had received transplantation at the age of three months.

Of the ten patients with OPTB1 who had received transpalnts, only two had retained sufficiently normal vision to be able to attend normal school. All others were registered blind and were attending specialist schools for the visually impaired. Two of the patients who had been transplanted at the age of 2 and 3½ months respectively were sisters. The sister transplanted at 2 months retains good vision whilst her younger sister, whose transplant had to be delayed, is registered blind.

The one patient with OLEDAID who was transplanted at 3 months retains good vision.

**CONCLUSIONS**

1. Transplantation done after the age of six months is uniformly associated with severe visual loss.
2. Even if transplant is done before the age of six months, vision may still not be preserved.
3. The earlier transplant is done, the greater the chance of avoiding significant visual loss.

### P154

### Hyperparathyroidism after 3 years of Burosumab in children affected with X-linked hypophosphatemia

Volha V Zhukouskaya[1,2,3], Diana-Alexandra Ertl[1], Jugurtha Berkenou[1], Christelle Audrain[1], Claire Bardet[2], Catherine Chaussain[1,2], Anya Rothenbuhler[1], Agnes Linglart[1]

[1]AP-HP, Endocrinology and diabetes for children, Reference center for rare diseases of the Calcium and Phosphate Metabolism, DMU SEA, OSCAR filière, EndoRare and BOND ERN, Bicêtre Paris Saclay Hospital, Le Kremlin Bicêtre, France. [2]Université Paris Cité, Laboratory Orofacial Pathologies, Imaging and Biotherapies URP2496 and FHU-DDS-Net, Dental School, and Plateforme d'Imagerie du Vivant (PIV), Montrouge, France. [3]AP-HP, Endocrinology department, Hôpital Cochin, Paris, France

### Abstract

**Background/Aim:** Hyperparathyroidism (HPHT) is a common feature in patients with X-linked hypophosphatemia (XLH) especially when treated with vitamin D analogues and phosphate supplements. Although the exact mechanism is not clear, it is assumed that phosphate supplements taken chronically stimulate parathyroid hormone (PTH) secretion. We prospectively assessed the effect of a novel pathogenetic treatment anti-FGF23 (burosumab) on PTH levels in children with XLH.

**Patients and methods:** 37 XLH-children (23 girls / 14 boys; 8.8±3.2 years) were switched from conventional therapy to burosumab and completed at least three years of burosumab therapy. Subjects with secondary HPTH at baseline were excluded from analysis. The biochemical parameters of calcium-phosphate metabolism were measured at baseline (M0) and then every 6 months under burosumab (M6 up to M36). According to our national guidelines, the target serum phosphate upon burosumab was >1.2 mmol/l (>3.7 mg/dl).

**Results:** After three years of treatment with burosumab, there was a global increase of PTH levels from 39.1±18.2 (M0) to 54.8±27.6 ng/l (M36) (18.5-88 ng/l) (p=0.001). Four subjects of 37 (10.8 %) developed secondary HPTH with mean±SD PTH levels equal to 110±17.9 ng/l. None of the children developed tertiary hyperparathyroidism. The subjects who developed HPTH had significantly higher PTH levels at baseline (62.8±22.5 *vs* 36.3±15.7 ng/l, p=0.004, respectively), and tended to have higher levels of serum phosphate at M36 (1.33 vs 1.19 mmol/l, p=0.11, respectively) and higher delta of phosphate increase from M0 to M36 (94% vs 63%, p=0.051, respectively), in comparison to those without HPTH. In ANOVA analyses, PTH levels increases with increase in serum phosphate> 1.3 mmol/l (p=0.012). Finally, serum phosphate is positively and independently associated with high PTH levels (OR=2.1, CI95%=1.1-3.8, p=0.021).

**Conclusion:** This is the first study describing the effect of anti-FGF23 treatment on PTH secretion in children with XLH. We suggest that normal high phosphate levels may stimulate PTH secretion thus leading to development of hyperparathyroidism after three years of treatment. Nonetheless, longer follow-up study is needed to better understand and describe this phenomenon.

### P155

### Response to bisphosphonate treatment in children with Chronic Recurrent Multifocal Osteomyelitis

Fahim Patel, Suma Uday, Penny Davis, Nicola Crabtree

Birmingham Children's Hospital, Birmingham, UK

### Abstract

### Background

Chronic Recurrent Multifocal Osteomyelitis (CRMO) is a rare autoinflammatory bone disease. Benefit from bisphosphonates (BP), mostly pamidronate, in CRMO has been reported in a handful of studies. We report our experience of using pamidronate and zoledronate in a large cohort of CRMO patients.

### Methods

A retrospective review of all CRMO patients diagnosed between 2008 and 2023 at a single tertiary centre. Data, from electronic patient records, was gathered on treatment, response in pain and dual energy X-ray absorptiometry (DXA) parameters [lumbar spine (L1-L4) bone mineral apparent density (LS BMAD) Z-score, total body less head (TBLH) BMD Z-score] and vertebral fracture assessment (VFA).

### Results

A total of 64 patients (72% female) were diagnosed with CRMO at a median age of 12 years, of whom 31% (n=20) received bisphosphonates. The majority received BPs for pain (45%, n=9) or pain and spinal involvement (45%, n=9) and rest for spine involvement only (10%, n=2). Prior to BP treatment, 80% (n=16) used NSAIDs regularly, 30% (n=6) had received steroids and 10% (n=2) were prescribed methotrexate.

The majority (70%, n=14) received zoledronate, 10% (n=2) received pamidronate followed by zoledronate and 10% (n=2) received pamidronate. The median treatment duration was 2 years (6 months–5 years). The median age at start of BP therapy was 12 years (6-18 years). Improvement in pain was reported by 82% (n=14 /17,3=missing data) after the first dose. De-escalation of adjunctive therapy was seen in 35% (n=7) within 12 months. After one year of treatment, the median increase in LS BMAD Z-score was + 1.4 SD (+0.1 to +2.1) and TBLH Z-score was +1.1 (+0.3 to +1.6). On surveillance spinal imaging (MRI or VFA), 27% (n= 3) had evidence of mild spinal remodelling. No remodelling was seen in patients with vertebra plana (n=4). First-phase reactions (pyrexia) were reported in all patients who received BP but none were significant.

### Conclusions

Similar to pamidronate, zoledronate is well tolerated in CRMO patients. BPs improved pain and enabled de-escalation of adjunctive therapy however spinal remodelling was minimal with no response in patients with vertebra plana. Bone density monitoring to avoid overtreatment is recommended.

### P156

*(this abstract was presented in the AHP Communications session, but did not have a printed poster)*

### The collaborative production of "The Hospital of Unhappy Surprises"

Yi Wen Wang[1], Frank Rauch[1,2] Kelly Thorstad[2] Franco Carnevale[1,2] Stephanie Smith[2] Argerie Tsimicalis[1,2]

[1]McGill University, Montreal, Canada. [2]Shriners Hospitals for Children-Canada, Montreal, Canada

### Abstract

**OBJECTIVES:**

Osteogenesis Imperfecta (OI) is a rare genetic bone disorder causing connective tissue weakening, bone deformities, and pain. OI remains largely unknown to healthcare providers, causing significant ethical concerns. Children from Shriners Hospitals for Children-Canada created a play called “The Hospital of Unhappy Surprises” detailing “bad” hospital environments and their vision for an ideal practice. However, the play captured a small subset of OI-affected children’s experiences. The objective was to understand the hospital experiences of OI-affected children *globally* to create a professionally animated, open-access play.

**METHODS:**

Ethno-dramatic storytelling was used to elicit children’s voices and transform oppressive scenarios into liberating ones. OI-affected children participated in semi-structured, online interviews. Children were shown a preliminary staging of the play, invited to discuss their experiences and act out scenes with print-out puppets, permitting the fictional portrayal of children’s lived experiences.

**RESULTS:**

Seventeen children (ages 4-18) participated from the United States, Canada, and Europe. The play reminded them of their own experiences, facilitating discussions with researchers. Children reported experiencing fear, frustration, and difficulties engaging in their OI care due to complex medical terminology, lack of information and resources about OI, and clinicians’ lack of OI knowledge. Children experienced elevated anxiety during x-rays and unexpected hospitalizations when pain control and communication were lacking.

**CONCLUSIONS:**

This study represents the push needed to: (a) understand the experiences of OI-affected children via alternative means of engagement (e.g., play, art); (b) produce educational resources that align with children’s needs; and (c) support clinician’s abilities to meet children’s needs.

### P157

### Skeletal and dental co-morbidities in children with hereditary hypophosphatemia compared to controls – a retrospective Danish register study

Signe Sparre Beck-Nielsen[1], Rikke Faergemann Hansen[2], Ulla Ege Johansen[2], Jennifer Gleisner[3], Angela Williams[4]

[1]Centre for Rare Diseases, Arhus University Hospital, Aarhus, Denmark. [2]Signum, Copenhagen, Denmark. [3]Kyowa Kirin AB, Copenhagen, Sweden. [4]Kyowa Kirin International, Marlow, UK

### Abstract

### Objectives:

Hereditary hypophosphatemia (HH) are rare diseases, characterized by excessive renal phosphate wasting and inappropriately low 1,25-dihydroxy-vitamin D causing hypophosphatemia. In children, the disease manifests as rickets and osteomalacia, in adults osteomalacia. The aim of this study was to describe the skeletal and dental co-morbidities in children (<18y) with HH compared to controls.

**Methods:** Through a register search based on five diagnosis codes referring to rickets or hypophosphatemia, a case population was identified in the Danish National Patient Register. A journal audit was performed based on all cases identified in Region of Midt Jutland and on available medical files from the remaining four regions in Denmark to verify the diagnosis of HH based on predefined diagnostic criteria. The background population (control) matched by gender, birth year and month, was randomly selected using the Danish Civil Registration System matching fifty controls to each child with HH. Data on skeletal co-morbidities were retrieved from the Danish National Patient Register, dental data from the Danish Health Authority's Central Odontology Register.

**Results:** The dataset comprised 94 children with HH matched with 4,700 controls. Compared to controls, children with HH were less prone to experience a fracture of the spine, pelvis, elbow and forearm, wrist and hand or metatarsal bone ($p<0.05$ for all mentioned sites). Overall, the relative risk of fractures among children with HH compared to controls was 0.7. Children with HH had a higher prevalence of skeletal manifestations of genu varum or valgum, dwarfism or craniosynostosis compared to controls (all $p<0.001$). Osteotomy and guided growth procedures had been undertaken in 19.1% and 19.2% of children with HH respectively. Children with HH had significantly higher prevalence of disease in dental pulp and periapical tissues compared to controls ($p<0.001$) and earlier age of presentation. There were no differences between HH and controls regarding number of decayed, missing, filled or extracted teeth.

**Conclusions:** This retrospective analysis of Danish register data shows a significantly lower prevalence of fractures in children with HH compared to controls in addition to a higher prevalence of already well-known skeletal manifestations and dental disease.

### Disclosure

Dr Signe Sparre Beck-Nielsen receives research funding and consultancy for Kyowa Kirin, safety board for Inozymes, invited lecture for Novo Nordisk Dr Jennifer Gleisner is an employee of Kyowa Kirin AB Dr Angela Williams is an employee of Kyowa Kirin International

### P158

### Natural history of the peripheral bones in children with osteogenesis imperfecta and age - and sex-matched healthy controls using longitudinal HR-pQCT analysis

Seyedmahdi Hosseinitabatabaei[1,2,3], Samantha McCluskey[1,2,3], Carolyn Denton[1,2,3], Elizabeth Zimmermann[1,3], Francis Glorieux[1], Fredrick Charbonneau[1], Frank Rauch[1], Bettina Willie[1,2,3]

[1]Research Centre, Shriners Hospital for Children-Canada, Montreal, Canada. [2]Department of Biomedical Engineering, McGill University, Montreal, Canada. [3]Faculty of Dental Medicine and Oral Health Sciences, McGill University, Montreal, Canada

### Abstract

Objectives: To compare bone density, microstructure, and strength at the radius and tibia between children with osteogenesis imperfecta (OI) and matched controls using longitudinal high-resolution peripheral quantitative computed tomography (HR-pQCT).

Methods: At baseline and 1-year, we scanned the radial and tibial metaphysis and diaphysis of 20 children with OI and 20 age- and sex-matched controls. We report trabecular (Tb.) and cortical (Ct.) volumetric bone mineral density (vBMD), trabecular bone volume fraction (Tb.BV/TV), separation (Tb.Sp), inhomogeneity (Tb.1/N.SD), and area (Tb.Ar), and cortical bone thickness (Ct.Th),area (Ct.Ar), and marrow (Mr.Ar). Bone strength from micro-finite-element analysis and 1-year changes were compared between the OI and control groups.

Results: At the tibial metaphysis, Tb.vBMD and Tb.BV/TV were lower and Tb.Sp and Tb.1/N.SD were greater in children with OI. Tb.Sp and Tb.1/N.SD increased for the OI group, but not the controls over 1-year, while Tb.vBMD only increased for controls. Ct.Th was higher for the control group at baseline compared to OI group. Ct.Th increased significantly, but similarly for both groups over 1-year. Ct.vBMD was similar at baseline, but changes were larger for the OI group. Strength was lower for the OI group but increased similarly for both groups. At the radial metaphysis, Tb.BV/TV, Tb.Sp, and Tb.1/N.SD were lower for the OI group. Both groups had similar cortical outcomes that increased over time, with larger Ct.vBMD increases and smaller Ct.Ar increases for the OI group. Strength was lower for the OI group, but increased similarly for both groups over 1-year. At the tibial and radial diaphysis, Mr.Ar and strength (only tibia) was significantly lower in the OI group, while tibial Ct.vBMD was higher for the OI group. Ct.vBMD increased for the OI group only, while other parameters showed similar changes over 1-year between the groups.

Conclusions: Deteriorated bone properties in the OI group at baseline was more prominent at the tibial metaphysis compared to the radius. The diaphyseal differences were mainly driven by lower area in children with OI. This is the first study to examine longitudinal changes in children with OI using HR-pQCT. Our results showed comparable 1-year changes in density, microstructure and strength.

### Disclosure

This research was supported by Mereo Biopharma and Shriners Hospitals for Children. FR and BMW. BMW and FR have received institutional research support and materials and have served as consultants for Mereo BioPharma.

### P159

### Combined administration of anabolic and antiresorptive drugs in dominant and recessive OI zebrafish models

Cecilia Masiero[1], Francesca Tonelli[1], Carla Aresi[1], Silvia Cotti[1], Marta Filibian[2], Filippo Doria[3], Paul Eckhard Witten[4], Antonella Forlino[1]

[1]Department of Molecular Medicine, Biochemistry Unit, University of Pavia, Pavia, Italy. [2]Laboratorio di Imaging a Risonanza Magnetica e Micro Tomografia a raggi X, Centro Grandi Strumenti, Pavia, Italy. [3]Department of Chemistry, University of Pavia, Pavia, Italy. [4]Biology Department, Evolutionary Developmental Biology, Ghent University, Ghent, Belgium

### Abstract

**Objectives:** Osteogenesis imperfecta (OI) is a group of collagen I related diseases characterized by skeletal fragility, frequent fractures and growth delay. The abnormal collagen I is both secreted and intracellularly retained, causing cellular stress. No resolutive treatment is so far available for OI. Bisphosphonates are currently administered to limit bone resorption and recently, the chemical chaperone 4-phenylbutyrate (4PBA) was demonstrated to increase collagen secretion in vitro and in vivo models of dominant and recessive OI. In this study, the *Chihuahua* (*Chi/+*) zebrafish, model for dominant OI, and the *p3h1-/-* zebrafish, resembling recessive OI, were exploited to evaluate the anabolic activity of 4PBA combined with the antiresorptive property of the bisphosphonate Alendronate (ALN).

**Methods:** WT, *Chi/+* and *p3h1-/-* adult fish were treated daily with 50 µM 4PBA dissolved in water and injected with 30 µM ALN once a week for 8 weeks. At the end of the treatment the expression of *col1a1a* and *bglap* was evaluated by qPCR. Bone volume (BV), vertebral thickness (V.Th) and bone mineral density (BMD) were evaluated by µCT. Toluidine blue staining and tartrate-resistant acid phosphatase (TRAP) were performed on histological sections to evaluate the bone microstructure and osteoclast activity, respectively.

**Results:** col1a1a and bglap expression was downregulated in both *Chi/+* and *p3h1-/-*respect to WT. The combined treatment significantly increased bglap expression in both models, whereas col1a1a was only increased in *p3h1-/-*.
µCT data showed impaired bone geometrical parameters in both the dominant and recessive OI zebrafish models. BV, V.Th and BMD significantly increased after the only-ALN and combined treatments only in *Chi/+*, while no effect was reported in *p3h1-/-*.
TRAP staining revealed significantly higher osteoclast activity in *Chi/+* vertebrae respect to WT, while only a slight augment was reported in *p3h1-/-*. Interestingly, the only-4PBA, only-ALN and combined treatments decreased osteoclast activity in *Chi/+*, while no effect was reported in *p3h1-/-*.

**Conclusions:** Our data showed that the combination of the anabolic activity of 4PBA with the antiresorptive activity of ALN in the zebrafish models has a different effect in dominant and recessive OI forms pointing to the relevance of identifying the specific causative molecular defect for a better OI treatment.

### P160

### Skeletal dysplasia - recommendations for genetic testing and diagnostic strategies

Marija Mijovic[1], Goran Cuturilo[1,2], Jelena Ruml Stojanovic[1], Aleksandra Miletic[1], Brankica Bosankic[1], Hristina Petrovic[1]

[1]University Children's Hospital, Department of Clinical Genetics, Belgrade, Serbia. [2]Faculty of Medicine, University of Belgrade, Belgrade, Serbia

### Abstract

OBJECTIVES:

Skeletal dysplasia are very heterogeneous group of genetic hereditary disorders. The most recent revision of the nosology and classification of genetic skeletal disorders from 2023 contains 771 disorders associated with 552 genes reflecting advances in molecular delineation of new disorders thanks to advances in DNA sequencing technology. Therefore, most genetic skeletal dysplasia are monogenic diseases. Very rarely, affected patients have a chromosomal aberration.

METHODS:

We conducted a retrospective analysis of data from UCH skeletal dysplasia database. Database contains next generation sequencing (NGS) and clinical data of 168 consecutive patients with different skeletal dysplasia syndromes. Goal of the present study was to determine the detection rate of the causative gene variants and propose a strategy in genetic testing.

RESULTS:

In our series of patients with skeletal dysplasia, we showed a high detection rate of the causative gene variants of 60.71% (102/168). Additionally, 5.95% (10/168) of a non-diagnostic patient reports had suspicious gene variant(s) of unknown significance (VUS), in which additional family studies that provide segregation data or functional characterization may result in reclassification to likely pathogenic.

CONCLUSIONS:

Our results showed that next generation sequencing achieved a high diagnostic rate for genetic skeletal dysplasia, helping clinical geneticists to confirm patients' molecular diagnoses, formulate treatment directions, and carry out genetic counseling. This study indicate that NGS should be the method of first choice in the genetic diagnostics of patients with skeletal dysplasia. Chromosomal tests, such as chromosomal microarray (*arrayCGH*), should be performed in patients with associated extraskeletal or multiple anomalies, if a firm diagnosis of a specific skeletal disease cannot be made clinically. Consideration of additional genetic testing and periodic reevaluation of NGS results are necessary for all negative patients.

### P161

### Age at diagnosis of X-Linked hypophosphatemia in children with vs. without a family history of the disorder: Results from the Disease Monitoring Program

Erik Imel[1], Thomas Carpenter[2], Hamilton Cassinelli[3], Pablo Florenzano[4], Jill Simmons[5], Shubham Tewari[6], Osman Cigeroglu[7], Angela Williams[8], Kerry Sandilands[8], Mark Vincent[9], Jerry Li[9]

[1]Indiana University School of Medicine, Indianapolis, IN, USA [2]Yale University School of Medicine, New Haven, NJ, USA [3]Hospital de Niños Ricardo Gutierrez, Buenos Aires, Argentina. [4]Pontificia Universidad Católica de Chile, Santiago, Chile. [5]Vanderbilt University School of Medicine, Nashville, TN, USA [6]KMK Consulting Inc., Morristown, NJ, USA [7]Ultragenx Pharmaceutical Inc., Novato, CA, USA [8]Kyowa Kirin International, Marlow, UK [9]Kyowa Kirin, Inc., Princeton, NJ, USA

### Abstract

**Objectives:** X-linked hypophosphatemia (XLH) is caused by inherited or de novo *PHEX* gene variants. The objective of this study was to examine whether a known family history (FH) of XLH influences the timing of diagnosis in children using data from the XLH Disease Monitoring Program (XLH-DMP).

**Methods:** The XLH-DMP is an ongoing, prospective, multicenter study of patients in the Americas designed to characterize the presentation and outcomes of XLH over 10 years (NCT03651505). This analysis included pediatric patients enrolled during 2018–2022. Patients were divided into two groups based on presence or absence of a family member with XLH. Age at XLH diagnosis was compared between groups using the t-test and Kaplan–Meier analysis. Cox proportional hazards modeling was used to analyze the associations between patient characteristics and age at XLH diagnosis.

**Results:** Among 347 children, 231 had a FH and 116 did not (mean age 8.4 and 10.2 years, 55% and 73% female, respectively). Kaplan–Meier analysis demonstrated that age at diagnosis was significantly earlier in children with a FH than in those without (log rank $P<0.0001$). Examining groups by age at enrollment, the finding persisted: mean age at diagnosis was lower in patients with a FH compared with those without at enrollment ages 1–4 years (0.8 vs. 2.6 years; $P<0.0001$), 5–11 years (1.8 vs. 2.6 years; $P<0.0145$), and 12–17 years (2.5 vs. 2.9 years; $P<0.5064$) (**Table 1**). Notably, patients with a FH enrolled at younger ages reported earlier initial diagnosis than those enrolled at older ages, suggesting that age at diagnosis may be decreasing in families with known affected members. Cox modeling adjusting for age at enrollment, country, gender, and race/ethnicity demonstrated a significant association between FH and age at XLH diagnosis.

**Conclusions:** This real-world study demonstrates that children with XLH without a FH were diagnosed significantly later than those in families known to be affected. Children enrolled in the younger age categories with a FH appeared to be diagnosed at earlier time points than those who were older at enrollment, suggesting that testing and diagnostic practices may have improved in recent years.

**Table 1.** Age at Diagnosis in Patients With vs. Without a Family History of XLH.

| Age Category at Enrollment (Years) | Age at XLH Diagnosis (Years) | XLH Family History | | Total | *P* |
|---|---|---|---|---|---|
| | | No | Yes | | |
| <1 | | (n=0) | (n=8) | (n=8) | – |
| | Mean (SD) | – | 0.2 (0.2) | 0.2 (0.2) | |
| | Median (IQR) | – | 0.2 (0.0, 0.3) | 0.2 (0.0, 0.3) | |
| | Range | – | –0.04, 0.46 | –0.04, 0.46 | |
| 1–4 | | (n=17) | (n=63) | (n=80) | <0.0001 |
| | Mean (SD) | 2.6 (1.0) | 0.8 (1.0) | 1.2 (1.2) | |
| | Median (IQR) | 2.6 (1.9, 3.0) | 0.5 (0.1, 1.3) | 0.8 (0.2, 1.9) | |
| | Range | 1.09, 4.65 | –0.09, 4.26 | –0.09, 4.65 | |
| 5–11 | | (n=53) | (n=101) | (n=154) | 0.0145 |
| | Mean (SD) | 2.6 (1.7) | 1.8 (2.3) | 2.1 (2.1) | |
| | Median (IQR) | 2.2 (1.7, 2.9) | 0.9 (0.2, 2.3) | 1.5 (0.5, 2.8) | |
| | Range | 0.45, 9.79 | –0.09, 10.91 | –0.09, 10.91 | |
| 12–17 | | (n=46) | (n=59) | (n=105) | 0.5064 |
| | Mean (SD) | 2.9 (2.7) | 2.5 (3.8) | 2.7 (3.3) | |
| | Median (IQR) | 2.2 (1.6, 3.0) | 0.9 (0.2, 3.5) | 1.9 (0.5, 3.0) | |
| | Range | –0.07, 13.52 | –0.10, 17.29 | –0.10, 17.29 | |

IQR: interquartile range; SD: standard deviation; XLH: X-linked hypophosphatemia

Name of funder: Kyowa Kirin, Inc., Princeton, NJ, USA

### Disclosure

- Tom Carpenter, Yale University School of Medicine – Yale University received funding as a site for carrying out this work (Ultragenyx) and received royalties for chapter contributions. (UpToDate), TC received direct payment for services rendered (Ultragenyx, Kyowa Kirin, Viridian), received honoraria for speaking at meetings sponsored by the entity (Kyowa Kirin, Ultragenyx), was reimbursed for travel to advisory board meeting (Kyowa Kirin, Ultragenyx), and received direct payment for services rendered for data safety monitoring (Kyowa Kirin, Ultragenyx). TC is an Assistant Editor at the Journal of Bone and Mineral Research and President of the Pediatric Endocrine Society (unpaid). Kyowa Kirin and Ultragenyx support their related manuscripts with medical writing - Hamilton Cassinelli, CEDIE – Medical writing support, article processing, grants or contracts from Kyowa Kirin and Ultragenyx; payment or honoraria for lectures, presentations, speakers bureaus, manuscript writing or educational events (Kyowa Kirin, Bio Sidus, Astrazeneca, Ultragenyx, Sandoz, Personal, Pediatric Endocrinology Foundation); payment for expert testimony (Astra Zeneca, Ultragenyx); support for attending meetings and/or travel (Ultragenyx, Novonordisk, Sandoz); member of the XLH DMP Steering Committee (Ultragenyx and Kyowa Kirin) - Pablo Florenzano - Pontificia Universidad Católica de Chile – Research grant (Ultragenyx), consulting fees (Kyowa Kirin, Ultragenyx), XLH DMP Steering Committee (Ultragenyx and Kyowa Kirin), IHN Steering Committee (Kyowa Kirin), Vice President Chilean Society of Bone and Mineral Metabolism - Erik Imel, Indiana University School of Medicine – Grants or contracts (Ultragenyx), consulting fees (Ultragenyx, Kyowa Kirin) - Jill Simmons, Vanderbilt University School of Medicine – Grants or contracts (Ultragenyx), consulting fees (Kyowa Kirin), member of XLH DMP Steering Committee (Ultragenyx and Kyowa Kirin) - Shubham Tewari, KMK Consulting Inc. –Employee of KMK Consulting Inc., which received financial compensation for this study from Kyowa Kirin - Osman Cigeroglu, Ultragenyx Pharmaceutical Inc – Employee of Ultragenyx - Angela Williams, Kyowa Kirin International – Employee of Kyowa Kirin - Kerry Sandilands, Kyowa Kirin International – Employee of Kyowa Kirin - Marc Vincent, Kyowa Kirin, Inc. – Employee of Kyowa Kirin, Inc. - Jerry Li, Kyowa Kirin, Inc. – Employee of Kyowa Kirin, Inc.

### P162

### From genotype to phenotype: The impact of early management in pycnodysostosis

Gustavo Tempone Cardoso Penna[1], Carolina Costa Figueiredo[2], Nara Michelle de Araújo Evangelista[2], Vânia de Fátima Tonetto Fernandes[1,2], Patricia Salmona[2], Guido de Paula Colares Neto[1,2]

[1]Centro Universitário São Camilo, São Paulo, Brazil. [2]Hospital Infantil Darcy Vargas, São Paulo, Brazil

### Abstract

**BACKGROUND:** We present four cases of Pycnodysostosis (PYCD), an osteosclerotic skeletal dysplasia caused by mutations in the *CTSK* gene, observing their clinical progression and therapeutic responses.

**PRESENTING PROBLEM AND CLINICAL MANAGEMENT:**

**Case 1:** A 3-year-old boy with related parents had short stature (Z-score -3.75), low growth rate (2cm/year), wide sutures, exophthalmos, blue sclera, small limbs, and multiple tibial fractures. He had a homozygous *CTSK* variant (c953G>A; p.Cys318Tyr). Without prior treatments and with normal IGF1 and a positive clonidine test, he began growth hormone at age 6, combined with cholecalciferol and calcium, increasing growth (7cm/year) and height (Z-score -2.65), with a fracture in tibia by 8.5 years.

**Case 2:** A 2-year-old girl with unrelated parents had disproportionately short stature (Z-score -3.23), slow growth (3cm/year), wide fontanelles, small hands, no exophthalmos, and no fractures. She used cholecalciferol and calcium previously. Two *CTSK* variants (c.436G>C; p.Gly146Arg and c.721C>T; p.Arg241*) were identified. Besides normal IGF1 and a positive clonidine test, somatropin was started at age 3, increasing growth (8cm/year) and stature (Z -2.21), with no fractures by age 6.

**Case 3:** A 2-year-old boy, sibling of Case 2, had disproportionate stature (Z-score -1.81), slow growth (4.5cm/year), wide fontanelles, small hands, and no fractures. Previously, he had cholecalciferol and prednisolone for wheezing. Two *CTSK* variants (c.436G>C; p.Gly146Arg and c.721C>T; p.Arg241*) were found. With normal IGF1 and a positive clonidine test, somatropin at age 3 improved his growth velocity (3cm/4 months) without fractures.

**Case 4:** An 8-year-old girl with related parents had short stature (Z-score -3) and slow growth (3.5cm/year), wide sutures, exophthalmos, blue sclera, small extremities, and numerous tibial fractures since birth. She had bisphosphonates for a wrongly assumed osteogenesis imperfecta. She had a homozygous *CTSK* variant (c953G>A; p.Cys318Tyr). By age 10, she had stunted growth (Z-score -3.27) with additional femur and tibia fractures.

**DISCUSSION:**

Besides the genotype's foundational blueprint for PYCD, clinical variability suggests the phenotypic expression modulation by early management. With cholecalciferol and calcium supplementation, growth hormone was a positive factor in enhancing growth and potentially improving bone metabolism in homozygous and compound heterozygous cases. Bisphosphonates negatively impacted bone quality in homozygous patients.

### P163

### Skull and spine changes in Autosomal Recessive Hypophosphatemic Rickets type 2 – a previously unreported finding

Pon Ramya Gokul[1], M Zulf Mughal[2], Ghadir Kassab[3], Senthil Senniappan[1], Charlotte Jarvis[1], Suzanne Armitage[1], Renuka Ramakrishnan[4]

[1]Alder Hey Children's Hospital, Liverpool, UK [2]Al Jalila Children's Hospital,, Dubai, UAE. [3]Alder Hey Children's Hospital, Liverpool, UK [4]Alder Hey Children's hospital, Liverpool, UK

### Abstract

**Background:**

Autosomal recessive hypophosphatemic rickets type 2 (ARHR2) is a rare hereditary form of rickets caused by a loss-of-function variants in *ENPP1*. This variant leads to chronic renal phosphate loss and impaired bone mineralisation. Inactivating *ENPP1* variants are associated with both generalised arterial calcification of infancy (GACI) and ARHR2. While pathogenic *ENPP1* variants are known to be associated with various phenotypes including vascular calcification, hearing loss, ossification of the posterior longitudinal ligament and pseudoxanthoma elasticum, skull changes and spinal canal narrowing have not previously been reported to our knowledge.

**Presenting Problem:**

We present a case of a 10-year-old girl who presented with right-sided knee pain and was diagnosed with ARHR2. She was compound heterozygous for pathogenic *ENPP1* mutation. ECHO, ultrasound carotid doppler and computed tomography abdomen showed no vascular calcifications. Her mother's antenatal period was normal, with no significant family history. Routine optician review identified bilateral papilledema a few years after her initial diagnosis. Neuroimaging showed lateral and third ventricular dilatation, narrowing of the foramen magnum, Arnold Chiari malformation (CM) type 1 and a retroverted odontoid peg. Spinal imaging showed fusion abnormalities at C1- T2 levels, with minimal C6-C7 disk bulges, lumbar canal spinal stenosis and crowding of the cauda equina nerve roots. She later developed occipital headaches and pain at the back of neck, which exacerbated on extension of the neck and valsalva manoeuvre with no associated sensory symptoms in limbs.

**Clinical Management:**

Clinical management included treatment with active vitamin D and phosphate supplements, along with corrective orthopaedic procedures. Two endoscopic third ventriculostomy procedures and a foramen magnum decompression were performed to manage chronic hydrocephalus. Her headaches and papilledema improved on further reviews.

**Discussion**:

The impact of rickets on the posterior fossa and craniocervical junction can cause bony abnormalities due to excessive production of incompletely calcified bone matrix. This may reduce the posterior fossa capacity leading to chronic hydrocephalus and also contribute to the development of CM. Our report highlights the need for regular surveillance with neuroimaging even in asymptomatic ARHR2 cases. Timely diagnosis and intervention to address these changes can optimise the clinical outcome.

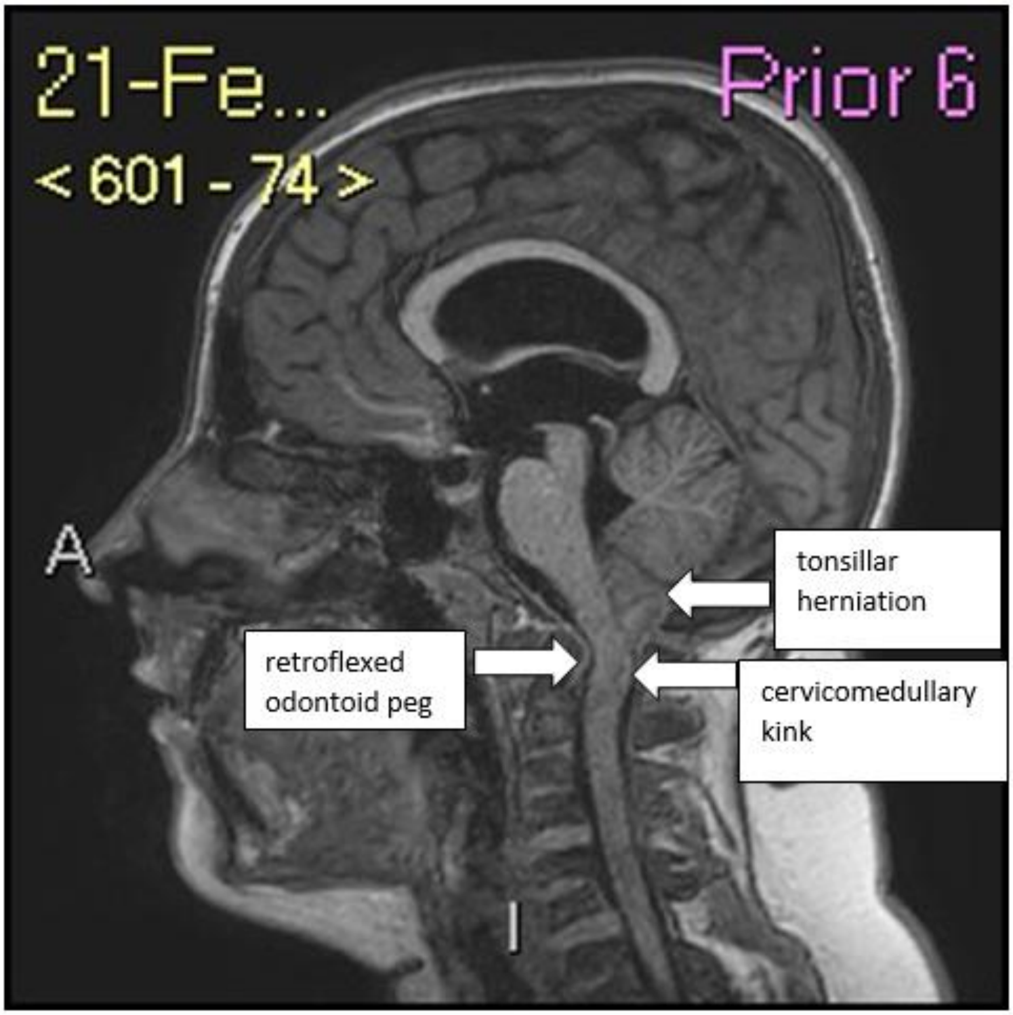

### P164

### Cross-sectional evaluation of quality of life in patients with X-linked hypophosphatemic rickets (XLH)

Florentina Haufler[1,2], Gabriel Mindler[3,2], Gabriele Hartmann[1,2], Adalbert Raimann[1,2]

[1]Department of Pediatrics and Adolescent Medicine, Division of Pediatric Pulmonology, Allergology and Endocrinology, Medical University of Vienna Department of Pediatrics and Adolescent Medicine, Division of Pediatric Pulmonology, Allergology and Endocrinology, Medical University of Vienna, Austria, Vienna, Austria. [2]Vienna Bone and Growth Centre, Vienna, Austria. [3]Department of Pediatric Orthopedics, Speising Orthopedic Hospital, Vienna, Austria

### Abstract

**Objective:** X-linked hypophosphatemia (XLH) is a rare metabolic bone disease caused by inactivating mutations in *Phosphate-regulating neutral endopeptidase, X-linked* (*PHEX*). Patients with XLH suffer from multiple musculoskeletal symptoms such as long bone deformities, short stature, bone pain, muscle weakness, dental abscesses and fatigue.

This study aimed to characterize QoL in a pediatric single center cohort by an age-specific self- and proxy-reported questionnaire. Further, this study targeted to identify relations between QoL and surrogate markers of metabolic affection.

**Methods:** In total, this study included 16 children, adolescents and young adults (f:m = 10:6; mean age 12.2 years) with genetically confirmed XLH. The German version of Pediatric Quality of Life Inventory (PedsQL ™), a standardized questionnaire to self-assess health related QoL, was performed as part of regular clinical visits. Biochemical, anthropometric and therapy-associated parameters were collected at the time of inclusion. Comparison with a healthy and an osteogenesis imperfecta (OI) cohort were assessed descriptively. Correlations were investigated by calculation of Spearman coefficients. The level of statistical significance was set at $p < 0.05$.

**Results:** BMI SDS and the numbers of osteotomies showed a negative correlation with physical QoL in the investigated pediatric XLH cohort (BMI SDS: $p = 0.033$; osteotomies: $p = 0.049$). According to the equ-BMI 41.5 % of patients in our study population were classified as overweight or obese. Disease specific factors such as age, alkaline phosphatase (ALP SDS) or the rickets severity score (RSS) did not predict QoL. Overall, mean QoL of the investigated XLH patients was comparable to a pediatric OI cohort.

**Conclusion:** In this study cohort, BMI and the numbers of osteotomies are associated with physical QoL impairment. Our data are in line with previous observations on increased rates of obesity in XLH. Higher BMI in XLH patients indicates a significant impact on disease burden, despite the incomplete understanding of this association. Further studies are needed to characterize the underlying mechanisms such as reduced mobility, increased rates of lower limb deformities or inflammatory factors. Identification of modifiable factors contributing to the burden of disease will help to optimize management of patients with XLH.

### Disclosure

A. Raimann and G. Mindler consulting Kyowa Kirin, not associated with the presented data.

### P165

### Treatment of Ghosal Haematodiaphyseal Dysplasia (GHDD) with ibuprofen results in normalization of bone mineral density over three years

Brianna Reilly[1], Ciara McDonnell[1,2]

[1]Children's Health Ireland, Dublin, Ireland. [2]Discipline of Paediatrics, Faculty of Medicine, Trinity College, Dublin, Ireland

### Abstract

**Background**: Ghosal Haematodiaphyseal Dysplasia (GHDD) is a rare autosomal recessive disorder characterised by pancytopenia and increased bone density (IBD). It is associated with loss of function mutations in the TBXAS1 gene encoding thromboxane synthase (TXAS), a crucial component of the arachidonic acid pathway responsible for the formation of thromboxane A2 ($TXA_2$) from prostaglandin H2.

**Presenting problem:** The proband was reviewed after identification of a homozygous TBXAS1 mutation on her whole exome sequencing following a presentation of marked anaemia and thrombocytopaenia. She was born to non-consanguineous parents of Filipino origin and prior to presentation at 3 years of age was an otherwise well child. On examination she was pale, with no dysmorphic features, skeletal disproportion, or physical sign of impaired bone health.

The proband had a normal bone mineralisation assessment prior to commencing treatment. Bone turnover biomarkers, skeletal biochemistry and skeletal survey were unremarkable apart from the appearance of increased bone density on plain radiographs. There was no increase in bony trabeculae appreciated on bone marrow biopsy.

**Clinical management**: Treatment was initiated with ibuprofen at regular therapeutic doses of 30 mg/kg/day, with the dose reduced to 10mg/kg/day after one year. The treatment was well tolerated with no gastrointestinal side effects.

DXA scans carried out at baseline and after 3 years of treatment showed a statistical significant decrease in Lumbar spine aBMD of 17.1%. Initial aBMD L1-4 = 0.638g/cm$^2$ (Z-score +2.4) Follow up aBMD L1-4= 0.529 g/cm$^2$ (Z-score -0.1).

**Discussion:** Inhibition of cyclooxygenase upstream of thromboxane synthase in the arachidonic acid pathway, by utilising NSAID's results in the normalisation of bone mineral density in tandem with resolution of haematological parameters. Treatment of GHDD with ibuprofen is a novel approach that demonstrates clear potential benefits with a superior safety profile in comparison to long term corticosteroids.[1]

### Reference:

1. Timothy J. Brown, Neil Barrett, Hu Meng, Emanuela Ricciotti, Ciara McDonnell, Andrew Dancis, Julianne Qualtieri, Garret A. FitzGerald, Melanie Cotter, Daria V. Babushok; Nonsteroidal anti-inflammatory drugs as a targeted therapy for bone marrow failure in Ghosal hematodiaphyseal dysplasia. *Blood* 2023; 141 (13): 1553–1559. doi: https://doi.org/10.1182/blood.2022018667

### P166

### Systematic review and meta-analysis on collagen pathophysiology in osteogenesis imperfecta

Priyesh Patel[1,2], Siron Aksornthong[1,2], Gurveer Palia[1], Bettina Willie[1,2], Elisa Sachaeffer[1], Svetlana Komarova[1,3]

[1]McGill University, Montreal, Canada. [2]Shriners Hospital, Montreal, Canada. [3]University of Alberta, Edmonton, Canada

### Abstract

**Introduction**:

Osteogenesis imperfecta (OI) is a rare connective tissue disorder characterized by bone brittleness caused by a mutation in type 1 collagen encoding genes or collagen processing genes. The severity of OI can vary drastically. The structure of type 1 collagen differs between healthy and OI collagen beyond base change mutation, due to the increased levels of post-translational modifications. As a result, structural changes in collagen alter the entire collagen pathway in OI. Our objective was to identify the existing literature and quantify OI-associated alterations in the entire collagen pathway, which encompasses collagen post-translational modifications, folding, secretion, crosslink, and mineralization.

**Methods:**

A systematic review and meta-analysis were performed to quantify all studies measuring outcomes related to collagen pathophysiology. Adaptations were made to the meta-analytic protocol to encompass case study publication with only one patient.

**Results**:

164 studies contained quantitative data for the selected outcomes, with 546 patients being described. The percentage increase for lysine hydroxylation was 43% [confidence interval of 32% to 51%] and 4% [1% to 6%] for proline hydroxylation. The OI fibril diameter was decreased by -25% [ -15% to -34%]. The melting temperature decreased by 2.2°C [2°C to 2.5°C] OI. The bone mineral density distribution had a CA mean increase of 0.9 [0.71 to 1.1.], CA peak increase of 1.45 [1.32 to 1.55] and CA width increase of 0.45[0.36 to 0.56] in OI. Due to limited data, the collagen folding, secretion, and crosslink outcomes are described qualitatively.

**Conclusion**:

This meta-analysis shows that OI's entire collagen pathway is modified. These findings provide valuable insights into the complex pathophysiology of OI and highlight the structural changes of collagen to the broader collagen pathway.

### P167

### Cosmic: An open-label, randomized, active-controlled, phase 3 study of setrusumab compared with bisphosphonates in pediatric subjects with osteogenesis imperfecta

Elżbieta Jakubowska-Pietkiewicz[1], Thomas Carpenter[2], Michael Bober[3], Gary Gottesman[4], Oliver Semler[5], Têmis Félix[6], Leanne Ward[7], Hui Wang[8], Heather Byers[8], Stanley Krolczyk[8]

[1]Department of Pediatrics, Newborn Pathology, and Bone Metabolic Diseases, Medical University of Lodz, Lodz, Poland. [2]Yale University School of Medicine, New Haven, USA [3]Nemours Children's Hospital, Wilmington, USA [4]Washington University School of Medicine, St. Louis, USA [5]University Hospital, University of Cologne, Cologne, Germany. [6]Medical Genetics Service of Hospital de Clínicas de Porto Alegre, Porto Alegre, Brazil. [7]Children's Hospital of Eastern Ontario, Ottawa, Ontario, Canada. [8]Ultragenyx Pharmaceutical Inc., Novato, USA

### Abstract

### Objectives:

Setrusumab is a recombinant, fully human monoclonal antibody that selectively binds and inhibits sclerostin, thereby increasing anabolic bone activity and decreasing bone resorption; setrusumab is in development for the treatment of osteogenesis imperfecta (OI). A Phase 2b study in adults with OI showed increases in bone formation, bone density, and bone strength with setrusumab across OI Types I, III, and IV (Asteroid; NCT03118570) and a pivotal Phase 2/3 study is ongoing to determine the effects of setrusumab on dose, efficacy and safety in children and young adults with OI (Orbit; NCT05125809).

### Methods:

This abstract describes the study design of *Cosmic*, an open-label, randomized, active-controlled, Phase 3 study of setrusumab compared with bisphosphonates in pediatric subjects 2 to <7 years of age with OI Types I, III or IV (NCT05768854). Key inclusion criteria are a clinical diagnosis of OI Types I, III, or IV; a history of ≥1 fracture in the past 12 months, ≥2 fractures in the past 24 months, or ≥1 femur, tibia, or humerus fracture in the past 24 months; and prior or current IV-BP. Exclusion criteria are a history of skeletal malignancies, bone metastases or neural foraminal stenosis; clinical manifestations of Chiari malformation or basilar invagination; current uncontrolled concomitant diseases potentially impacting bone metabolism; any skeletal condition (other than OI) causing bone deformity and/or increased risk of fractures; and cardiovascular disease.

### Results:

The primary objective is to evaluate the effect of setrusumab vs intravenous bisphosphonate (IV-BP) therapy on fracture rate reduction. Participants will receive either setrusumab or IV-BP for up to 24 months during an active-control period. All participants will enter an extension period for at least 12 months, with those initially assigned to IV-BP transitioning to setrusumab, if desired.

The primary endpoint is the annualized rate of all radiographically-confirmed fractures, including morphometric vertebral fractures. Secondary endpoints include the annualized rate of radiographically-confirmed fractures, excluding morphometric vertebral fractures; change from baseline in POSNA-PODCI

Sports/Physical Functioning and Pain/Comfort Subscale Scores; serum setrusumab concentration; and the subject incidence of TEAEs, SAEs, and AEs of special interest.

**Conclusions:**

The *Cosmic* study initiated in June 2023 and is currently recruiting.

### Disclosure

TC: Research Investigator (funds to institution): Ultragenyx; Advisory Board and consulting: Ultragenyx Pharmaceutical Inc, Inozyme, Kyowa Kirin MB: Research investigator (funds to institution): Ultragenyx. Consultant to Ultragenyx and Mereo. GG: Research Investigator: Ultragenyx; Advisory Board: Ultragenyx Pharmaceutical Inc: Consulting fees TF: Research investigator (funds to institution): Ultragenyx Pharmaceutical Inc. LW: Consultant to and participant in clinical trials with Ultragenyx Pharmaceutical Inc. (with funds to Dr. Ward's institution, the Children's Hospital of Eastern Ontario Research Institute) HW, HB, SK: Ultragenyx: Employee and stock owner

### P168

### Pain and its biopsychosocial impact in children with skeletal dysplasias

Matthew Boston[1], Saunya Dover[1], Joanna Lazier[1], Sasha Carsen[1], Kevin Smit[1], Leanne M Ward[1], Eve Blanchard[1], Mary-Ann Matzinger[1], Nazih Shenouda[1], Khaldoun Koujok[1], Adam Khalif[1], Frank Rauch[2], Marie-Eve Robinson[1]

[1]Children's Hospital of Eastern Ontario, Ottawa, Canada. [2]Shriners Hospital for Children, Montreal, Canada

### Abstract

**Objectives:** To describe the pain experienced by children with skeletal dysplasias and to determine which biopsychosocial outcomes are associated with pain.

**Methods:** Participants aged 0-18 years with a confirmed or suspected skeletal dysplasia were recruited from the multidisciplinary clinic for children with skeletal dysplasias at the Children's Hospital of Eastern Ontario in Ottawa, Canada. Parents/caregivers completed a series of validated questionnaires during a routine clinic visit to evaluate their child's pain (current pain and worst pain in the previous week as assessed by a 10 cm visual analog scale on the Pediatric Pain Questionnaire), health-related quality of life (HRQoL), fatigue, pain interference, symptoms of depression and anxiety, sleep, peer relationships, and physical function. As part of the physical function assessment, participants aged ≥6 years also completed single a two-legged jump (to assess lower body muscle power). We summarized our cohort and outcomes using descriptive statistics (mean/standard deviation [SD] or median/rage as appropriate) and tested for associations using Pearson's correlations.

**Results:** Thirty-two participants (41% female) with a mean age of 11.8 (SD 4.1) years were included. Diagnoses included achondroplasia (9/32; 28%), osteogenesis imperfecta (type I, III or IV, 5/32; 16%), juvenile osteoporosis (3/32; 9%), and other skeletal dysplasias (15/32, 47%). Twenty (63%) parents/caregivers reported that their child experiences pain. Of those, median current pain and worst pain in the previous week levels were 2.1 (range 0.2-9.8) and 5.0 (range 0.2-100), respectively. The top 5 biopsychosocial outcomes associated with current pain were pain interference ($r=0.73$, $p<0.0001$), HRQoL ($r=-0.56$, $p<0.0001$), peer relationships ($r=-0.51$, $p=0.004$), fatigue ($r=0.45$, $p=0.01$), and depressive symptoms ($r=0.38$, $p=0.03$). The top 5 biopsychosocial outcomes associated with worst pain the previous week were pain interference ($r=0.72$, $p<0.0001$), HRQoL ($r=-0.65$, $p<0.0001$), fatigue ($r=0.53$, $p=0.002$), peer relationships ($r=-0.52$, $p=0.002$), and weight-adjusted single 2-legged jump score ($r=-0.49$, $p=0.05$).

**Conclusion:** Pain is prevalent in children with skeletal dysplasias and is associated with increased pain interference, fatigue and depressive symptoms, as well as decreased HRQoL, peer relationships and muscle power. Those biopsychosocial outcomes should be further explored to determine appropriate targets for future interventions to help prevent and treat pain in children with skeletal dysplasias.

### Disclosure

Leanne Ward reports study grants to institution from Ascendis, Catabasis, PTC Therapeutics, ReveraGen, Ultragenyx and Amgen, and consultancy fees to institution from Ipsen, Santhera, Amgen, Alexion and Ultragenyx. Frank Rauch reports study grants to institution from PreciThera Inc, Ultragenyx Inc and

Catabasis. Marie-Eve Robinson declares study grants to institution from Ascendis Biopharma, QED therapeutics, and Ipsen Biopharmaceuticals and consultancy fees to institution from Ipsen Biopharmaceuticals and Ultragenyx.

### P169

### A mixed methods analysis of the lived experience of adolescents living with X-Linked hypophosphataemia at the End of Skeletal Growth (EoSG)

Vrinda Saraff[1], Pedro Arango Sancho[2], Justine Bacchetta[3], Annemieke Boot[4], Christine Burren[5], Amish Chinoy[6], Poonam Dharmaraj[7], Maria Amelia Gómez Llorente[8], Juan David González Rodríguez[9], Iva Gueorguieva[10], Elin Haf Davies[11], Pol Harvengt[12], Wesley Hayes[13], Sandra Komarzynski[11], Héctor Ríos Duro[14], Angela Jane Rylands[15], Kerry Sandilands[15], Dirk Schnabel[16], San Selveindran[17], Agnès Linglart[18]

[1]Birmingham Women's and Children's Hospital, Birmingham, UK [2]Sant Joan de Déu Barcelona Hospital, Barcelona, Spain. [3]Hospices Civils de Lyon, Lyon, France. [4]University Medical Center Groningen, Groningen, Netherlands. [5]University Hospitals Bristol and Weston NHS Foundation Trust, Bristol, UK [6]Royal Manchester Children's Hospital, Manchester, UK [7]Alder Hey Children's Hospital, Liverpool, UK [8]Hospital Virgen de Las Nieves, Granada, Spain. [9]Department of Pediatric Nephrology, Santa Lucia General University Hospital, Cartagena, Spain. [10]Centre Hospitalier Universitaire de Lille, Lille, France. [11]Aparito Ltd, Wrexham, UK [12]Belgium XLH Patient Association, Waterloo, Belgium. [13]Great Ormond Street Hospital, London, UK [14]Pediatric Nephrology, Vall d´Hebron Universitary Hospital, Barcelona, Spain. [15]Kyowa Kirin International, Marlow, UK [16]Center for Chronic Sick Children, Pediatric Endocrinology, Charité – University Medicine Berlin, Berlin, Germany. [17]Open Health Ltd, Marlow, UK [18]AP-HP, Paris Saclay University, Paris, France

### Abstract

**Objectives:** X-linked hypophosphatemia (XLH) is a rare, genetic disease due to PHEX mutations resulting in elevated FGF23 levels  manifesting as chronic hypophosphatemia. Limited data describes the experiences of adolescents with XLH. This observational, prospective, European, multicentre, mixed methods study describes the lived experience of XLH at end of skeletal growth (EoSG).

**Methods:** Data were collected from adolescents treated with burosumab for 4 weeks within a 26 week-period prior to EoSG  via   smartphone app,  wearable device and 1:1 interviews. Here we combine quantitative smartphone app daily reported data on symptom (pain, stiffness and fatigue) intensity (measured on a scale of 0 [none] to 10 [worst imaginable]) with associated qualitative 1:1 interview data that was thematically analysed.

**Results:** 24 adolescents participated (62.5% female) with the mean age at EoSG of 16.9  ± 1.1 years for males and 15.0 ±1.1 years for females. Participants were treated with burosumab for a mean 4.5 ±2.1 years prior to EoSG. Fatigue intensity median (IQR) score 1.85 (1.97) was the highest symptom score. Fatigue was described as a daily (n=4, 17%) or periodical (n=10, 42%) occurrence, usually following physical activity which limited subsequent activity choices, and in four (17%), impacted education.  Pain intensity was low with a median score 1.02 (2.00) being described as minimal and manageable, achy and occurring in the lower limbs. There was limited emotional impact because of "being so accustomed to it" with only three (13%) participants reporting negative feelings due to pain and eight (33%) reporting interference with daily physical activities. Stiffness intensity median score was 0.65 (1.12) described as brief (minutes to an hour) and in relation to moving; 'unable to move' or feeling like 'bones 'cracking' in the lower limbs. Three (13%) reported emotional impacts, mainly due to mobility limitations from stiffness.

**Conclusions:** To our knowledge, this is the first detailed account of the lived experience of adolescents treated with burosumab with XLH, using a mixed methods analysis. Quantitative scores of XLH symptom severity were minimal during the period prior to EoSG. Qualitative data indicated some emotional impact and limitations to participation in social and physical activities.

### Disclosure

Vrinda Saraff received an honorarium for lectures and advisory board participation from Kyowa Kirin International and served as Lead Study Advisor for this study. Annemieke M. Boot has received research grants from Ultragenyx and Kyowa Kirin, and payment for lectures and consulting fees from Kyowa Kirin. Juan David Gonzalez received speaker fees (in educational conferences) and participated in clinical reports sponsored by Kyowa Kirin; Elin Haf Davies is the Chief Executive Officer of Aparito Ltd funded in relation to this study by Kyowa Kirin International; Sandra Komarzynski is employed by Aparito Ltd and funded in relation to this study by Kyowa Kirin International; Angela J Rylands is an employee of Kyowa Kirin (study sponsor); Kerry Sandilands is an employee of Kyowa Kirin (study sponsor); Dirk Schnabel received honoraria for consulting & advisory board roles as well as travel support from Kyowa Kirin, Ascendis, BioMarin, Kyowa Kirin, Novo Nordisk, and Sandoz. San Seveindran is employed by OPEN Health and funded by Kyowa Kirin International for this study. Agnès Linglart received honoraria from Kyowa Kirin International for being a presenter at symposia, support for attending meetings.

### P170

### Scoliosis in osteogenesis imperfecta - quality of life and surgical impact

Janus Wong, Michael To

The University of Hong Kong, Hong Kong

### Abstract

OBJECTIVES:

We aim to investigate whether scoliosis among osteogenesis imperfecta (OI) patients is associated with lower quality of life, and whether orthopaedic surgery is associated with higher treatment satisfaction.

METHODS:

OI patients were recruited through the University of Hong Kong Shenzhen Hospital in this cross-sectional epidemiological study. Patients with a confirmed clinical or genetic diagnosis of OI were invited to complete questionnaires where demographics, health seeking behaviour, treatment received, radiographs, and quality of life measures (SRS-22 and EQ-5D-5L) were assessed. OI patients were divided into the following groups: no scoliosis with Cobb angle <10, scoliosis of Cobb angle between 10 and 50 without receiving treatment, scoliosis of Cobb angle over 50 without receiving treatment, scoliosis treated with bracing only, and scoliosis treated with surgery. Patients without confirmed diagnoses of OI, or without radiographs for assessment of spinal deformities were excluded.

RESULTS:

148 OI patients - 101 with scoliosis (M=60, F=41, mean age 16.5 years) and 47 without scoliosis were analysed. The scoliosis group had lower self-image (3.31 vs 3.70, p=0.003) and mobility (3.13 vs 3.66, p=0.015). Within the scoliosis groups, the median Cobb angle for the primary curve was 34.5 (interquartile range 16.8-58.4), with elder patients having greater curves (σ=0.544, p<0.001). Curve magnitude was associated with pain (σ=-0.355, p<0.001) and anxiety (σ=-0.352 p<0.001), but inversely correlated with function (σ=-0.225, p=0.024), self-image (σ=-0.379, p<0.001), total SRS score (σ=-0.283, p=0.004). 11.9% underwent scoliosis surgery, 13.9% underwent bracing, and 41.6% underwent physiotherapy. 14.9% reported that they did not receive any treatment for scoliosis because they did not have the means, while 23.8% were not aware or uncertain whether they had scoliosis. Patients treated surgically reported higher treatment satisfaction scores (4.41 vs 3.43, p=0.002) than patients with scoliosis of Cobb angle over 50 without receiving treatment, despite similar curve magnitudes (p=0.354).

CONCLUSIONS:

OI patients with scoliosis reported lower self-image and mobility. Curve magnitude was associated with older age, pain and anxiety, and was inversely correlated with function, self-image, and total SRS score. Patients who received surgery reported higher treatment satisfaction than those managed nonoperatively.

### P171

### Delayed bone healing observed among type XV osteogenesis imperfecta with WNT1 mutations

Janus Wong, Michael To

The University of Hong Kong, Hong Kong

### Abstract

OBJECTIVES:

Bone healing rates in WNT1-mutated type XV OI patients versus classic COL1A1-mutated type I OI patients is unknown. We aim to quantitatively investigate differences in bone healing between OI caused by WNT1 and COL1A1 mutations, to prognosticate and guide clinical management.

METHODS:

OI patients in 3 regional tertiary referral centres with genetically confirmed WNT1 and COL1A1 mutations with femoral or tibial fractures were screened for inclusion. Pathological or incomplete fractures, patients who received recent bisphosphonate therapy, or had inadequate follow up were excluded. Age-matched normal subjects with femoral and tibial fractures were recruited as controls. Fracture union was defined radiologically as a modified RUST (Radiographic Union Score for Tibia) score of 13 or above. Age, gender, location of fracture, presence of operative fixation, and previous bisphosphonate use were screened as possible confounders. Cox multivariable regression model incorporating identified covariates was used to detect difference in time to bony union among the 3 groups.

RESULTS:

82 fractures (19 fractures from WNT1 patients, 22 fractures from COL1A1 patients, and 41 fractures from normal controls) were included in analysis. All fractures were followed up till union, with a mean of 66.8 days. There was substantial agreement (Cohen's Kappa: 0.717) in radiographic assessment of bone healing through modified RUST.

Mean time to bony union in the WNT1 and COL1A1 groups were 57±9 days and 51±9 days respectively, which were significantly slower than the normal control group of 35±9 days ($p<0.001$). Univariable analysis identified age and operative intervention as potential covariates. Multivariable Cox regression model adjusted for age and operative intervention demonstrated a difference in time to bone healing between WNT1 and COL1A1 patients (adjusted HR 0.013, 95%CI 0186-0.819, p=0.013). On subgroup analysis this difference was observed more in the femur (adjusted HR 0.831, 95%CI 0.747-0.926, p=0.015) than the tibia (adjusted HR 0.408, 95%CI 0.074-2.244, p=0.303).

CONCLUSIONS:

Bone healing is slower in OI patients with WNT1 mutations compared to COL1A1 and normal subjects. Orthopaedic surgeons, patients, and carers should be aware of the longer time for bone healing WNT1 mutation entails, and its implications on postoperative immobilisation, weight bearing restriction, and rehabilitation.

### P172

### Incidental detection of joint calcification on chest radiograph of neonate - a case of generalised arterial calcification of infancy

Lydia Forestier-Zhang[1], Paul Arundel[1], Alistair Calder[2], Stephen Giles[1], Gemma Greenacre[1], M Zulf Mughal[3], Amaka Offiah[4], Ashok Raghavan[1], Frank Rutsch[5], Fiona Shackley[1], Benjamin Shillitoe[1], Alison Stewart[1], Christopher Vas[6], Stephanie Borg[1]

[1]Sheffield Children's NHS Foundation Trust, Sheffield, UK [2]Great Ormond Street Hospital for Children NHS Foundation Trust, London, UK [3]Al Jalila Children's Hospital, Dubai, UAE. [4]The University of Sheffield, Sheffield, UK [5]Münster University Children's Hospital, Münster, Germany. [6]Sheffield Teaching Hospitals NHS Foundation Trust, Sheffield, UK

### Abstract

Generalised arterial calcification of infancy (GACI) is a rare disorder caused by mutations in *ENPP1*. GACI is characterised by infantile onset of widespread arterial calcification and neointimal proliferation, leading to arterial stenosis. Calcification also occurs in joints and parenchymal organs. Mortality in the first six months of life is high due to cardiovascular complications. Patients who survive develop renal phosphate wasting and hypophosphataemic rickets beyond infancy.

A term female neonate presented on day one of life with respiratory distress, oxygen requirement and raised inflammatory markers (C-reactive protein 111mg/L). Calcification of the left glenohumeral joint was incidentally found on chest radiograph. Examination identified reduced left arm movement. Initially treated for osteomyelitis with intravenous antibiotics; no organisms were identified, and inflammatory markers remained elevated. Whole body magnetic resonance imaging (MRI) and abdominal ultrasound were normal. Her respiratory distress resolved, and initial echocardiogram was normal. Further radiographs and subsequent whole-body computed tomography (CT) revealed extensive arterial and joint calcification. Plasma inorganic pyrophosphate concentration was low at 0.11µm (healthy control 2.22 µm). Whole genome sequencing identified compound heterozygote pathogenic mutations of the *ENPP1* gene.

There are no approved treatments for GACI. She was commenced on monthly pamidronate; the preferred first-generation bisphosphonate etidronate could not be sourced. Echocardiogram identified mild pulmonary artery stenosis with no myocardial dysfunction. She has mildly raised proteinuria and stable blood pressure. She has bilateral hearing aids for conductive hearing loss. Biochemical monitoring has shown modestly reduced TmP/GFR (1.13). Radiographs aged eight months showed resolution of joint calcification.

GACI is a rare, potentially lethal condition to consider when joint calcification is identified in infancy. Whole-body CT imaging is suggestive of the diagnosis if intravascular calcification is identified. Ultrasound, echocardiogram, and MRI were falsely reassuring with intravascular calcification not seen, due to inadequate resolution. Persistent raised inflammatory markers can add to diagnostic challenge and may relate to the calcification process, although the mechanism remains poorly understood. There is no cure for GACI. Whilst bisphosphonates are used there is little evidence of a positive effect. An ENPP1 enzyme replacement therapy is currently in clinical trial and may be a promising future treatment.

### P173

### Differential diagnosis of low serum ALP: A retrospective laboratory survey

Julian Schirl, Thomas Hörtenhuber, Elisabeth Laurer, Katharina Tischlinger, Rodrigo Montero-Lopez, Alexandra Blaschitz, Wolfgang Högler

Department of Paediatrics and Adolescent Medicine, Johannes Kepler University Linz, Linz, Austria

### Abstract

**Objectives:**

Hypophosphatasia (HPP) is a rare multisystemic genetic disorder caused by pathogenic variants in *ALPL*. Disease severity varies substantially leading to diagnostic delay in less symptomatic forms of HPP. A persistently low alkaline phosphatase (ALP) is the hallmark laboratory finding of HPP. However, low ALP values are often overlooked in everyday clinical practice, which led us to conduct a laboratory survey to assess the frequency and possible causes of low ALP.

**Methods:**

In this retrospective data analysis we included all patients at Kepler University Hospital with at least two low serum ALP measurements between January 2021 and January 2022. Medical records were used as a data source. Patients with no identifiable cause for persistent low ALP were classified as possibly having HPP. The lower limit of serum ALP was defined using sex- and age-specific reference values.

**Results:**

Out of 42.304 patients with ALP measurements, 1.161 had at least one low ALP value detected. Out of these, 537 patients (365 adults, 172 children) fulfilled the inclusion criteria. The most common causes were transiently low ALP values following cardiac surgery (n=185, 34.5%) and during acute severe illnesses (n=159, 29.6%). Additionally, medications such as fibrates (n=10, 1.9%) or denosumab (n=10, 1.9%) and underlying oncological diseases were associated with low ALP. No cause for low ALP could be identified in 91 patients (16.9%), 39 of whom had persistently low ALP (7.3%) and were therefore classified as possibly having HPP.

**Conclusions:**

In the vast majority of patients with at least two low ALP measurements secondary causes could be identified. Nevertheless in 7.3% of patients HPP has to be considered a likely cause. Knowledge of differential diagnosis of low ALP is important to identify those patients that deserve further investigation for HPP.

### Disclosure

WH has worked as consultant and received research funding and travel support from Alexion Pharmaceuticals.

### P174

### Osteoclast and bone resorption indices in patients and animal models with osteogenesis imperfecta: Systematic review and meta-analysis

Sirion Aksornthong[1,2], Priyesh Patel[2,3], Svetlana V. Komarova[1,2,3]

[1]Department of Experimental Surgery, McGill University, Montreal, Canada. [2]Shriners Hospital for Children – Canada, Montreal, Canada. [3]Faculty of Dental Medicine and Oral Health Sciences, McGill University, Montreal, Canada

### Abstract

**Background:** Osteogenesis imperfecta (OI) is a rare bone fragility disorder caused by mutations in genes that encode collagen type I or affect its processing. Alterations in bone resorbing osteoclasts were suggested to contribute to OI pathophysiology.

**Objective:** We aimed to systematically identify studies reporting measures of osteoclast formation and function in patients and mouse models of OI, to quantify OI-induced changes.

**Method:** The systematic search of Medline, OVID and Web of Science identified 798 unique studies. After screening, we included for meta-analysis 23 studies reporting osteoclast parameters in 310 OI patients of 9 different types and 16 studies reporting osteoclast parameters in 406 animals of 11 different OI mouse models. The standardized mean difference was used as the effect size, random effects meta-analysis was performed using R-studio.

**Result:** In OI patients, collagen degradation markers were significantly higher in patients with OI compared to age-matched control with the effect size of 1.23 [Confidence interval (CI): 0.36, 2.10]. Collagen degradation markers were the most elevated in the 3 to 7-year-old age group, and in patients with more severe forms of OI. Bone histomorphometry demonstrated the trends for higher osteoclast numbers, 1.16 [CI: -0.22, 2.55], and osteoclast surface, 0.43 [CI: -0.63; 1.49], and significantly higher eroded surface, 3.24 [CI: 0.51, 5.96] compared to the aged-match control. In OI mice, meta-analysis demonstrated significant increases in collagen degradation markers, 1.59 [CI: 1.07, 2.11]; in osteoclast numbers, 0.94 [CI: 0.50, 1.39], osteoclast surface, 0.73 [CI:0.22, 1.23], and eroded surface 1.31[CI: 0.54, 2.08]. The largest differences were in OI mice with the mutations in Col1a1 and Col1a2 genes. There were no differences between males and females in clinical or animal studies.

**Conclusions:** Quantitative estimates of changes in osteoclast indices and their variance for patients with OI are important for planning future studies. We confirmed that similar changes are observed in mice with OI, supporting their translational utility.

### P175

### Chondrodysplasia phenocopy and short stature in children after early haematopoietic stem cell transplant for non-oncologic and oncologic disorders

Cheng Guang Gan, Alistair D Calder, Maaike A Kusters, Austen J J Worth, Alexander D Chesover

Great Ormond Street Hospital for Children NHS Foundation Trust, London, UK

### Abstract

**BACKGROUND:**

Skeletal dysplasias (chrondrodysplasias) are individually rare genetic disorders of skeletal development that usually result in disproportionate short stature and more frequently now have an identified genetic cause. Paediatric haematopoietic stem cell transplant (HSCT) is associated with growth failure and some children with non-oncologic disorders (mainly hemophagocytic lymphohistiocytosis) have been described with a genetic-negative chondrodysplasia phenocopy after early HSCT. We present a relatively large paediatric cohort with both non-oncologic and oncologic disorders after early HSCT with short stature and radiological findings of a chondrodysplasia.

**PRESENTING PROBLEM:**

Total 14 children, 5/14 with an oncological diagnosis. Current age 8-15 years and all >5 years after their first HSCT, which was completed within the first 5 years of life and all had only chemotherapy conditioning except one required additional myeloablative total body irradiation. There was no evidence of chondrodysplasia prior to their primary disease diagnosis. The current height Z-score is either <-2.5 or <-2 SD below the mid-parental height.

**CLINICAL MANAGEMENT:**

Imaging findings include: abnormalities of the iliac crests (irregularity and/or laciness), vertebrae (mostly platyspondyly), metaphyses (mainly irregularity and striations), and epiphyses (dysplasia). In 13/14, at least two areas have a significant radiographic abnormality. Two patients have been established on growth hormone for growth hormone deficiency but shown an unusually poor response to treatment.

**DISCUSSION:**

We present the largest case series of children with chondrodysplasia phenocopy and growth failure after early HSCT. This case series uniquely describes the chondrodysplasia in children with both oncologic and non-oncologic disorders. Although some primary disorders are associated with a skeletal phenotype, this chondrodysplasia phenocopy is not characteristic of these conditions, is acquired, and shows a similar pattern across all cases. Although the aetiology of a HSCT-associated chondrodysplasia phenocopy is unknown, better characterising this entity could help contribute to patient information prior to HSCT, understand risk factors for it’s development, and predict response to other therapies, such as growth hormone. Our next steps include investigating the prevalence in our patient cohorts and measuring patient-reported outcome measures.

### P176

### Comorbidity and mortality burden among patients with hypochondroplasia in England between 1998-2019

Sheila R Reddy[1], Jeanne M Pimenta[2], Hilary Shepherd[3], Jaim Sutton[2], Moira Cheung[4], Melita Irving[4], Swati Mukherjee[2], Rachael Williams[3]

[1]BioMarin Pharmaceutical Inc., Novato, USA [2]BioMarin (UK) Limited, London, UK [3]Clinical Practice Research Database (CPRD), Medicines and Healthcare products Regulatory Agency (MHRA), London, UK [4]Guy's and St Thomas' National Health Service (NHS) Foundation Trust, London, UK

### Abstract

**OBJECTIVES:** Hypochondroplasia (HCH) is a rare genetic skeletal condition resulting in disproportionate short stature. This study aimed to assess and contextualise the natural history of HCH compared to the general population in England.

**METHODS:** This was a retrospective, real-world matched cohort study using 1998-2019 electronic medical records (EMR) from the Clinical Practice Research Datalink (CPRD-Aurum) linked to hospitalisation and vital statistics data. HCH patients were defined by a specific HCH code (SNOMED:315057016) or general code (ICD-10-CM:Q77.4) without evidence of other growth conditions. HCH patients were indexed by first HCH record and matched 1:4 to controls by sex, nearest birth year, and general practice (GP) region. Event rates (number of events/100 person-years) and rate ratios (RR) (cases versus controls) were calculated for selected comorbidities, healthcare use, and mortality.

**RESULTS:** 610 HCH patients and 2,440 matched controls were included. Mean (standard deviation) follow-up time among cases and controls was 7.4 (7.1) and 12.0 (7.3) years. Most patients were aged 16-65 years at start of follow-up (63.6% and 58.4%); mean age at first HCH code was 28 years although 20% had a record before 11 years. Comorbidity event rates were higher among HCH patients vs. controls across most body systems. Respiratory, cardiovascular, orthopedic, and mental health-related events were most frequent among HCH patients (16.5-24.3 episodes/100 person-years) with RRs>1 (3.9, 3.1, 4.9, 2.0, respectively) indicating significantly increased burden compared to controls. GP visits (RR=1.8; median annual visits 11.2 vs. 5.4), inpatient stays (RR=5.5), and average length of hospital stay (5.6 vs. 3.8 days) were all greater among HCH patients compared to controls. Mortality was higher among HCH patients 16-65 years (RR=5.5) and >65 years (RR=2.0) vs. controls; respiratory and circulatory diseases were the most common causes of death.

**CONCLUSIONS:** This large observational study of paediatric and adult patients found HCH patients experienced substantially higher rates of both skeletal and non-skeletal comorbidity events than controls across the age-spectrum. Use of EMR data for patients of all ages allowed for broad assessment of medical impact and determination of mortality. These results highlight the medical impact of HCH and underscore the need for coordinated and multidisciplinary management.

### Disclosure

JMP, SM: Employees of BioMarin and hold shares in BioMarin. JS: Paid consultant for BioMarin. HS, RW: Full or part-time employees of the CPRD. MI, MC: Have received consulting fees for providing clinical input to this study from BioMarin

### P177

### Beyond body mass index: Unfavorable body composition of children and adolescents with osteogenesis imperfecta

Ravit Regev[1,2], Erez Azoulay[1], Asaf Ben Simon[1,2], Yael Lebenthal[1,2], Leonid Zeitlin[2,3], Avivit Brener[1,2]

[1]The Institute of Pediatric Endocrinology and Diabetes, Dana-Dwek Children's Hospital, Sourasky Medical Center, Tel Aviv, Israel. [2]Faculty of Medicine, Tel Aviv University, Tel Aviv, Israel. [3]The Metabolic Bone Disease Unit, Dana-Dwek Children's Hospital, Sourasky Medical Center, Tel Aviv, Israel

### Abstract

**Objectives**:

Osteogenesis Imperfecta (OI) refers to the spectrum of connective tissue disorders characterized by increased bone fragility. Low bone mass, immobilization after a fracture and fear of injury can adversely affect body composition. Bisphosphonate therapy aimed to increase bone mineral density, reducing the risk for recurrent fractures, reduces pain and hasten their recovery. We aimed to investigate the body composition parameters of individuals with OI undergoing bisphosphonate therapy.

**Methods**:

We carried out a retrospective observational study in a tertiary medical center from August 2018 to December 2023. Sociodemographic and clinical information was extracted from medical records, including age at bisphosphonates initiation. Body composition had been assessed during routine clinic visits using bioelectrical impedance analysis (BIA) and z-scores for appendicular skeletal muscle mass (ASMM) and muscle-to-fat ratio (MFR) were computed.

**Results**:

The study included 169 BIA assessments of 68 individuals (42 boys, 61.8%) diagnosed with various types of OI. Individuals with OI were 12.7±5.4 years (range 5.1-19 years) at first BIA. Bisphosphonates therapy had already been initiated at a mean age of 8.2±4.7 years (range 0.04-18.4 years) in 138 (81.2%) of BIA assessments. Although the mean BMI z-score of subjects with OI was within the normal range (0.16±1.11), their body composition assessment revealed relatively low muscle mass and muscle-to-fat ratio z-scores (-1.00±0.86 and -0.81±0.94, respectively). Age was positively correlated with both ASMM z-scores (r=0.356, $p<0.001$) and MFR z-scores (r=0.189, $p=0.013$).

**Conclusions**:

Young people with OI exhibit an unfavorable body composition marked by lower muscle mass, placeing them at risk for sarcopenic obesity. The relationship between OI and adverse body composition underscores the importance of its assessment within this at-risk population. Our findings highlight the need for targeted interventions to regulate the balance between muscle and adipose tissue.

### P178

### Somewhere to go for adults with childhood-onset rare diseases: A conversation about how we can fill gaps in care

Laura Tosi[1], Eleanor Perfetto[2], Melissa Bonardi[2], Erika Carter[2], Stacie Connors[2], Tracy Hart[2]

[1]Children's National Hospital, Washington, USA [2]Osteogenesis Imperfecta Foundation, Gaithersburg, USA

### Abstract

### Background:

For most of the more than 30 million Americans living with a rare disease (RD), the illness began in childhood. Many receive specialized pediatric care, but then encounter a void with with "nowhere to go" in adulthood. With growing numbers of identifiable RDs and emerging therapies extending lives, the dearth of appropriate, adult-focused RD care is a growing national problem. In response, the Osteogenesis Imperfecta (OI) Foundation organized a patient-community-driven, solution-oriented conference entitled, 'Somewhere to Go for Adults with Childhood-Onset Rare Diseases: A Conversation About How We Can Fill Gaps in Care.' The objectives were to: 1) Convene a multistakeholder, multidisciplinary working meeting. 2) Enumerate care gaps, barriers, and challenges RD patients face transitioning from pediatric to adult care; 3) Think strategically about solutions; and 4) Prioritize issues/solutions the assembled group could address collaboratively over 2-3 years.

### Methods:

The planning committee developed a 2-day meeting (attended by 47 participants/speakers) which included presentation sessions: 1) Envisioning 21st century centers of excellence; 2) Leveraging patient data and experiences; 3) Care-coordination innovations; and 4) Leveraging coalitions/collaboratives. Following each session, discussion facilitated by guiding questions explored issues and identified actions. Following the final session, the group reviewed, refined, and prioritized the issues/actions discussed.

### Results:

Guided discussions followed by a final review, produced 21 refined issue/action combinations; the top 3 were: 1) Perform a "landscape analysis" that identifies who is doing what and explore where that can be joined, built on, leveraged, disseminated, scaled); 2) Develop transition success measures; and 3) Leverage use of telemedicine in transition.

### Conclusion:

The convened group plans to conduct a landscape analysis in 2024 that serves as the next step in a collaborative effort to develop an RD Transition-Resource Information Center. The OI Foundation will continue to convene the group to advance this objective.

### P179

### Lower limb deformity in different types of rickets - a systematic literature review

Anahita Mayr[1], Gabriel T Mindler[2,3], Adalbert Raimann[1,3]

[1]Comprehensive Center for Pediatrics, Department of Pediatrics and Adolescent Medicine, Division of Pediatric Pulmonology, Allergology and Endocrinology, Medical University of Vienna, Vienna, Austria. [2]Department of Pediatric Orthopaedics, Orthopaedic Hospital Speising, Vienna, Austria. [3]Vienna Bone and Growth Center, Vienna, Austria

### Abstract

OBJECTIVES:

Lower limb deformities are a major cause of burden of disease in patients with rickets and associated disorders. Nevertheless, structured data on this substantial symptom in hypophosphatemic and hypocalcemic conditions is sparse. This review aims to depict the available data and identify gaps in the current literature on lower limb deformities in patients with hypophosphatemic rickets (HPR) such as XLH and hypocalcemic types of rickets (HCR).

METHODS:

A comprehensive literature search on PubMed was conducted using the terms “rickets” and “deformity” in combination with the Boolean operator “AND” until 08/2023. The inclusion criteria comprised studies, featuring pediatric case series with specification of the type of rickets and a description of lower limb deformities. Exclusion criteria included review articles, book chapters, redundant patient data, and secondary rickets.

RESULTS:

126 studies with 2924 patients presenting with leg deformity associated with rickets qualified for study inclusion. Nearly equal numbers of varus deformities were reported among patients with HPR and HCR. However, the prevalence of valgus leg deformity and windswept deformities was notably higher in HCR patients. Pathologic femoral torsion was exclusively documented in HPR (n=70), alongside 85 cases with procurvatum and 6 cases of recurvatum deformities of the femur. Seven studies assessing sagittal tibial deformities revealed a higher prevalence in the HPR cohort 54,8% of the included studies reported on basic laboratory assessments (ALP and Phosphorus or Calcium) in all patients. Only 17,5% assessed and defined ≥ 1 radiographic or functional parameter (e.g. gait laboratory data) to quantify deformities. Merely 5,6% (7/126) of the included studies covered both minimal radiological and basic laboratory assessments.

CONCLUSIONS:

*The current data on X-linked hypophosphatemia (XLH) and other rickets forms presents limitations, hindering precise conclusions on deformity variations. Only 5,6% of studies document lower limb deformities using standardized, basic radiological and lab parameters. Available data is heterogeneous, often lacking in minimal standards for sufficient interpretation. Establishing minimal quality criteria for deformity assessment is crucial for addressing this vulnerable patient cohort's needs. Deformity associated with rickets is a multidisciplinary challenge - and in need of multidisciplinary research approaches.*

| Type of deformity | HCR | HPR | Total |
|---|---|---|---|
| Varus | 1065 | 1001 | 2066 |
| Valgus | 834 | 365 | 1199 |
| Maltorsion femur | 0 | 70 | 70 |
| Maltorsion tibia | 8 | 39 | 47 |
| Procurvatum femur | 0 | 85 | 85 |
| Recurvatum femur | 0 | 6 | 6 |
| Sagittal deformity tibia | 7 | 95 | 102 |
| Windswept | 86* | 18* | 104* |
| **patients* | | | |

**Disclosure**

Gabriel T. Mindler, M.D. and Adalbert Raimann, M.D. : Consulting/lecturing Kyowa Kirin, not associated with presented data.

### P180

### Primary hyperparathyroidism: A misleading family history

Ruggero Lanzafame, Elisa Sala, Ludovica Crocè, Giorgia Piselli, Gabriele Drago, Stefano Mora, Marco Pitea

IRCCS Ospedale San Raffaele, Milano, Italy

### Abstract

BACKGROUND:

Primary hyperparathyroidism is a common endocrine disorder in adults in whom the typical presentation is incidentally discovered as asymptomatic hypercalcemia. It is much less common in children and adolescents. Typical biochemical features are hypercalcemia and hypophosphatemia, but the clinical features can be heterogeneous, and in some cases symptoms are vague and nonspecific, leading to misdiagnosis or late diagnosis.

PRESENTING PROBLEM:

A 17-year-old girl came to our attention for a second opinion due to reduced bone mineralization. She had a family history of osteogenesis imperfecta: her mother and younger sister carry a *COL1A1* mutation. The girl had no history of fractures, but considering her family history, bone density measurement was performed, showing a Z-score of -1.9 SDS at the femoral level and -2 SDS at the lumbar level; no fractures were detected in the spine X-ray. Genetic testing for *COL1A1* was negative.

CLINICAL MANAGEMENT:

The patient brought her blood test results to our first evaluation: PTH exceeding the normal values (9.4 pmol/L with normal values 1.6-6.9) and calcium at the upper limits (11 mg/dL), 25OH-vitamin D 29.7 ng/mL. An ultrasound of the neck showed a 6 x 11 mm nodule with poor intrinsic vascular signal at the lower pole of the left thyroid lobe; Tc99/sestaMIBI parathyroid scan showed a focal radiopharmaceutical uptake indicating hyperactivity of the left inferior parathyroid gland (*image attached*). Tests to rule out MEN1, MEN2A, and MEN4 were all negative: blood pressure monitoring, urinary catecholamine measurement, calcitonin, chromogranin, prolactin, fasting glucose, insulin, abdomen ultrasound and *MEN1*, *RET* and *CDKN1B* gene analysis.

The patient underwent left inferior parathyroidectomy and histological examination confirmed the presence of a parathyroid adenoma. PTH and calcium concentrations normalized after the procedure and the patient is currently not requiring calcium supplementation.

DISCUSSION:

Primary hyperparathyroidism should be considered in pediatric patients as a potential cause of reduced bone mineralization: an excess of PTH leads to an increased bone resorption. In these cases it is essential to perform an ultrasound of the thyroid and parathyroid glands and a Tc99/sestaMIBI parathyroid scan for early identification and treatment of parathyroid adenomas.

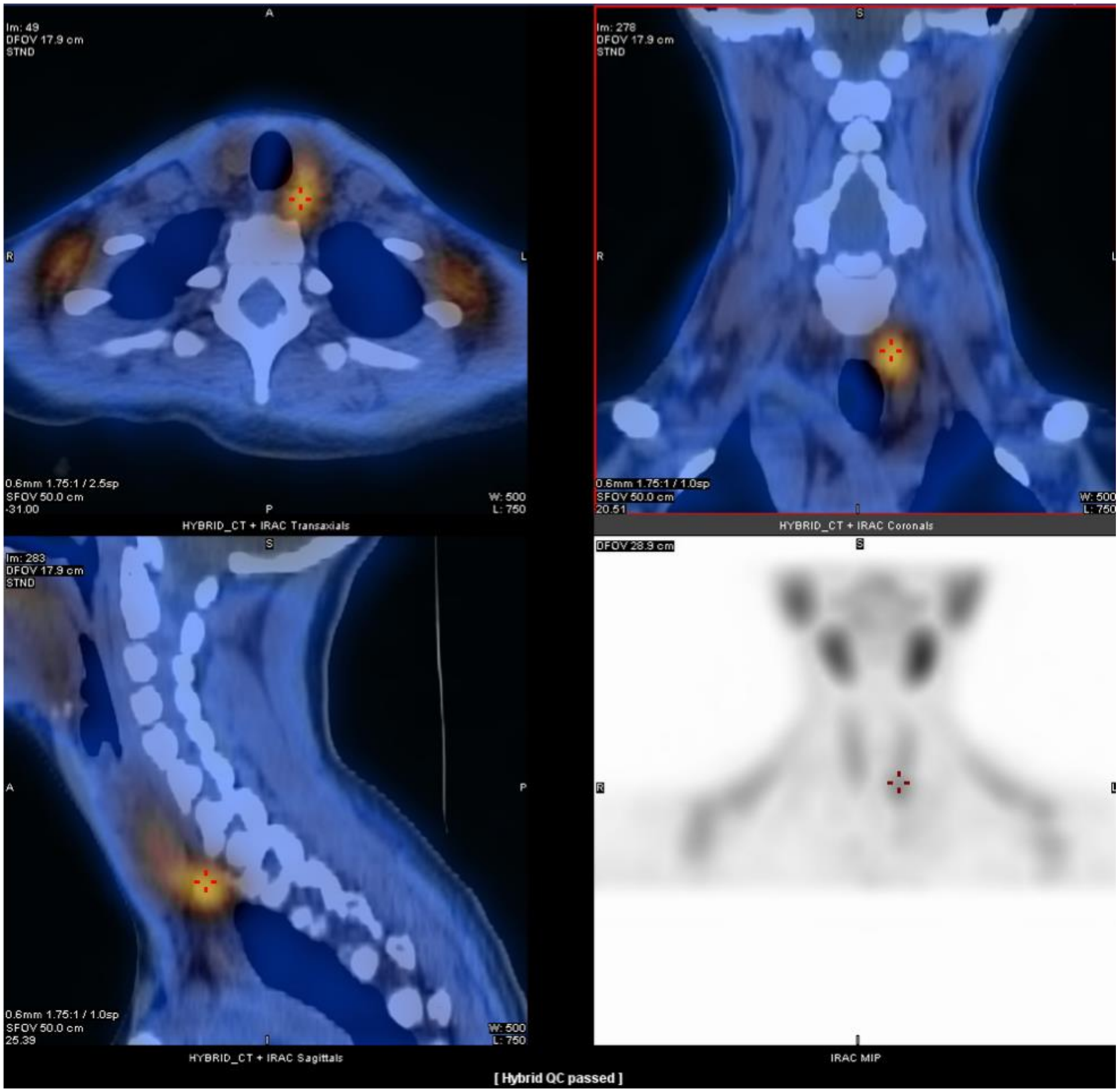

Im: 49
DFOV 17.9 cm
STND
0.6mm 1.75:1 / 2.5sp
SFOV 50.0 cm
-31.00
W: 500
L: 750
HYBRID_CT + IRAC Transaxials
Im: 278
DFOV 17.9 cm
STND
0.6mm 1.75:1 / 1.0sp
SFOV 50.0 cm
20.51
W: 500
L: 750
HYBRID_CT + IRAC Coronals
Im: 283
DFOV 17.9 cm
STND
0.6mm 1.75:1 / 1.0sp
SFOV 50.0 cm
25.39
W: 500
L: 750
HYBRID_CT + IRAC Sagittals
DFOV 28.9 cm
IRAC MIP
[ Hybrid QC passed ]

### P181

### Unveiling a rare paediatric phenomenon: Juvenile Paget disease with secondary hyperparathyroidism

Aravinda Karunarathna, Jananie Suntharesan

Sirimavo Bandaranayake Specialized Children's Hospital, Peradeniya, Sri Lanka

### Abstract

**Background:**

Juvenile Paget Disease (JPD) is a rare genetic disorder resulting from mutations in the *TNFSF11B*, impacting Osteoprotegerin (OPG) synthesis and causing accelerated bone turnover. Commonly associated with elevated alkaline phosphatase (ALP) levels, secondary hyperparathyroidism is a frequent manifestation due to increased calcium demands by pagetic bone. This case report details a unique presentation of Juvenile Paget Disease with hyperparathyroidism in a 10-year-old boy.

**Presenting problem and clinical management:**

The patient first sought medical attention at the age of 5, reporting low-trauma fractures since the age of 3. Initial assessments revealed high ALP, vitamin D insufficiency, and normal Parathyroid hormone (PTH). DXA scan confirmed low bone mineral density. He was treatment with vitamin D and IV Pamindronic acid which was discontinued after 2 years. Bilateral sensory neuronal hearing loss with normal vision was also diagnosed. At 8 ½ years, he experienced another low-trauma fracture, prompting the resumption of treatment with IV Zoledronic acid. Subsequent joint, bone, and back pain persisted, accompanied by elevated PTH and insufficient vitamin D. Lytic lesions in the left femur were revealed in X-rays. Despite appropriate vitamin D treatment, symptoms persisted with elevated PTH. Treatment with adequate calcium and vitamin D supplementation along with physiotherapy resulted marked clinical improvement.

**Discussion:**

Increased PTH levels in Juvenile Paget Disease can be attributed to heightened osteoclast activity, leading to increased bone resorption. Secondary hyperparathyroidism is also seen in Paget disease due to factors such as anti-resorptive therapy and inadequate vitamin D and calcium intake. This case emphasizes the importance of thorough management, including adequate vitamin D and calcium replacement, during anti-resorptive therapy for Juvenile Paget Disease, ultimately contributing to marked clinical improvement.

**Conclusion**:

This rare presentation of Juvenile Paget Disease with secondary hyperparathyroidism underscores the need for comprehensive understanding and management of this complex pediatric condition.

### P182

### Two long-term surviving patients with atelosteogenesis type 2 associated with compound heterozygous variants in SLC26A2 gene

Daria Gorodilova[1], Tatiana Markova[1], Vladimir Kenis[2], Alexandra Filatova[1], Vyacheslav Tabakov[1], Evgeniy Melchenko[2], Mikhail Skoblov[1], Elena Dadali[1], Sergey Kutsev[1]

[1]Research Centre for Medical Genetics, Moscow, Russian Federation. [2]H.Turner National Medical Research Center for Children's Orthopedics and Trauma Surgery, Saint Petersburg, Russian Federation

### Abstract

**OBJECTIVES:** Atelosteogenesis type II (AO2; OMIM #256050) is a rare predominantly lethal at birth or shortly thereafter skeletal dysplasia caused by biallelic variants in *SLC26A2* gene

**METHODS:** We present clinical radiological and genetic characteristics of 2 surviving patients with AO2. To confirm diagnosis Sanger sequencing of the *SLC26A2* gene was used. To study the pathogenicity of novel missense variant a sulfate uptake assay was performed on fibroblast cultures from one patient and his father.

**RESULTS:** Two probands (boy and girl) from two unrelated families firstly presented at the age of 3-year 5 months and 17 months, respectively. Fetal ultrasound in the first trimester revealed pronounced shortening of the tubular bones and bilateral talipes. After birth, immediate respiratory support was provided to both probands due to tracheobronchomalacia and pulmonary hypoplasia. The boy's birth height was 42 cm (-3,18 SD), and the girl's height was 36 cm (-6,52 SD). Preliminary diagnosis of diastrophic dysplasia/atelosteogenesis was made after radiological and clinical investigation by multidisciplinary team of clinical geneticists and orthopedic surgeons specialized in skeletal dysplasias. Musculoskeletal abnormalities included severe shortening of the tubular bones, bilateral hip, knee and elbow dislocations, hand abnormalities and severe bilateral clubfeet. Cervical kiphosys on the radiographs was accompanied by spinal stenosis confirmed with MRI. At the time of presentation boy's height was 65 cm (-8,09 SD). No pinnae deformation was detected. Proband adapted to walking on his knees due to pronounced knee and hip contractures. The girl's height was 47 cm (-9,9 SD), cleft palate diagnosed after birth, pinnae swelling occurred at 2 months. Due to preliminary clinical diagnosis, molecular analysis of the SLC26A2 gene revealed two known variants: p.Cys653Ser and p.Ser551ValfsTer34 in compound heterozygosity in the boy and known p.Arg279Trp and novel p.Met418Arg variants in compound heterozygosity in the girl. It was demonstrated that c.1253T>G (p.Met418Arg) leads to the dramatically reduction in sulfate transport.

**CONCLUSIONS:** Here, we report two long-term surviving patients with AO2, one of them associated with novel missense variant in *SLC26A2* gene. Clinical and radiological manifestation included those typical for SLC26A2 spectrum disorders with maximal severity. The pathogenicity of the novel variant was proven by measuring SLC26A2 protein transport activity.

### P183

### Treatment responses in an adolescent male with combined Camurati-Engelmann Disease and Crohn's disease

Elise Nauwynck, Nathalie Smeets, Koen Huysentruyt, Annelies Scholliers, Boyan Dimitrov, Aurélie Andrzejewski, Eva De Wachter, Jean De Schepper

UZ Brussel, Jette, Belgium

### Abstract

### Background

Camurati-Engelmann disease (CED) is a rare, autosomal dominant sclerosing bone disease, characterized by a progressive symmetric diaphyseal sclerosis of the long bones due to increased transforming growth factor beta 1 (TGFb1) activity. We report our experience with corticosteroids, Losartan and anti-TNF alfa treatments in a male adolescent with both CED and Crohn's disease.

### Presenting problem

A 15-year-old boy with autism was referred for gait disturbance, muscle weakness and progressive pain in his lower limbs. Growth analysis showed a progressive decline in body mass index (BMI) and height. He had also poor appetite, regular abdominal cramps and diarrhea. On physical examination, his height was 161 cm (Z score -1.9) and BMI 14.49kg/m$^2$ (Z score -3.5). He was at Tanner stage I. Neurological examination showed an antalgic gait with signs of muscle weakness. EMG was normal. Diaphyseal sclerosis and hyperostosis was seen at the skull and long bone X -rays. Biochemical investigation showed an increased sedimentation rate, normal creatine kinase, decreased gonadotropins and testosterone concentrations and an increased faecal calprotectin. Pelvic MRI and total colonoscopy revealed distal ileum inflammation. Intestinal biopsy confirmed inflammatory bowel disease (IBD) type Crohn. Genetic analysis identified a heterozygous pathogenic variant c.241T>C p.(Tyr81His) in exon 1 of the TGFB1 gene, confirming the diagnosis of CED.

### Clinical Management

Temporary bone pain relief was seen after oral corticosteroid treatment and intravenous Infliximab, given as inflammatory bowel disease treatment. Subsequent trials with Losartan and high dose intravenous prednisolone treatment resulted in only temporary improvement of its bone and abdominal pain. The same *TGFb1* mutation was present in his father, known with a thoracic aortic aneurysm and bone pain as an adolescent, and his one year younger brother, presenting with excessive weight gain but no bone pain or muscular complaints.

### Discussion

This case report illustrates the wide clinical variation of *TGFb1* gene mutations within families and confirms previous reports on associations with obesity as well as Crohn's disease. The need for the development of new bone targeted medications in CED is of utmost importance.

### P184

### A novel *CLCN7* variant in siblings with autosomal recessive intermediate osteopetrosis

Alexander Chesover[1,2], Chrysanthi Tsika[3], Benjamin Jacobs[1], Sagnik Sen[3,4], Alessandro Vidoni[1], Genevieve A Wright[3], Michel Michaelides[3,4]

[1]Royal National Orthopaedic Hospital, London, UK [2]Great Ormond Street Hospital for Children, London, UK [3]Moorfields Eye Hospital, London, UK [4]UCL Institute of Ophthalmology, London, UK

### Abstract

**BACKGROUND**. Osteopetrosis is a rare disease characterised by high bone mass. *CLCN7* encodes an $H^+/Cl^-$ exchanger and sequence variants cause defective acidification of the osteoclast resorption lacunae. *CLCN7* variants cause a spectrum of osteopetrosis from severe infantile to mild adult-onset types. Intermediate osteopetrosis is characterised by radiographic changes, sometimes anaemia, rarely visual impairment, and absent central nervous system involvement. We present a novel homozygous variant in *CLCN7* (c.480G>C, p.[Lys160Asn]) in siblings with features of intermediate osteopetrosis. Consent for publication was provided.

**PRESENTING PROBLEM**. 14-year-old female (P1), 13-year-old female (P2), and 8-year-old male (P3). All siblings are homozygous for the *CLCN7* variant. Their parents are consanguineous and heterozygous for the *CLCN7* variant. All have no significant fracture history. DXA lumbar spine shows bone mineral density Z-scores: +5.5 in P1, +1.8 in P2, +8.4 in P3. Plain radiographs: P1 and P3 have diffusely increased bone density consistent with osteopetrosis; P2 has minimal metaphyseal sclerosis. MRI head: P1 has diffuse bone thickening with narrowing of the optic foramina; P3 has narrowing of the internal auditory meati; P2 has no abnormalities. Ophthalmology: all have normal visual acuity and are asymptomatic; P1 shows mild rod dystrophy (by electrophysiology) and peripheral retinal pigmentary changes; P2 and P3 show mild peripheral retinal pigmentary changes and electrophysiology pending. P1 is heterozygous for a pathogenic variant in *IQCB1;* P2 is negative for the *IQCB1* variant; and P3 has pending genetic testing. All have normal full blood count, normal hearing, normal EEG, and normal brain MRI.

**DISCUSSION.** All three have features of intermediate osteopetrosis; however, milder in P2. The novel *CLCN7* variant is absent from population databases: dbSNP, Exome Variant Server, ExAC exome database. The lysine at position 160 is highly conserved across species and has a moderate physicochemical difference with the substituted asparagine. Two variants within the same protein domain have also been associated with osteopetrosis. We present three siblings with a phenotype consistent with a novel autosomal recessive intermediate osteopetrosis. Retinal degeneration without optic nerve pathology is rarely reported in osteopetrosis and, without evidence for another aetiology, these siblings' retinal pathology may be attributed to the *CLCN7* variant.

### P185

### 10 Years with hypophosphatasia: Insights from the Endocrinology and Metabolic Diseases Department of the Polish Mother’s Memorial Hospital Research Institute in Lodz, Poland

Izabela Michałus[1], Ewa Starostecka[1], Agnieszka Konopka[1], Łukasz Kępczyński[2], Agnieszka Gach[2], Arkadiusz Zygmunt[1], Andrzej Lewiński[1]

[1]Polish Mother’s Memorial Hospital – Research Institute, Department of Endocrinology and Metabolic Diseases, Lodz, Poland, Lodz, Poland. [2]Polish Mother’s Memorial Hospital – Research Institute, Department of Genetics, Lodz, Poland, Lodz, Poland

### Abstract

**Objectives:** Hypophosphatasia (HPP) is a life-threatening, progressive, systemic, and genetically inherited metabolic disease with severe implications for patients across all age groups. It stems from a mutation in the ALPL gene, responsible for encoding tissue-nonspecific alkaline phosphatase (TNSALP). The disease's presentation and severity vary based on the form and the age of symptom onset. However, the intricate diversity of forms and symptoms has left the natural history of HPP poorly comprehended.

The aim of study has been to present the clinical picture of hypophosphatasia in patients with symptoms suggesting calcium-phosphate metabolism disorders, all diagnosed with HPP.

**Methods:** Data from 20 patients (11 females, 9 males), aged 1 month to 52 years, diagnosed with hypophosphatasia between 2013 and 2023 at the Endocrinology and Metabolic Diseases Department or Inborn Metabolic Diseases Outpatient Clinic at the Polish Mother’s Memorial Hospital—Research Institute in Lodz, Poland, were studied.

**Results:** Diagnoses included perinatal (2, 1 fatality), infantile (3), childhood (10), odontohypophosphatasia (3), and adult forms (2). Genetic testing predominantly confirmed diagnoses in children aged 2-6 years (10 patients).

Common skeletal symptoms involved premature tooth loss (12/20 patients), bone deformities (10/20), fractures (10/20), and chronic bone pain (9/20). All patients exhibited reduced alkaline phosphatase activity, hypercalcemia (11/20), hyperphosphatemia (11/20), and decreased parathyroid hormone levels (7/20) in calcium-phosphate metabolism disorders.

Abdominal ultrasound detected urolithiasis in 3/20 patients. X-rays commonly showed bone mineralization disturbances and metaphyseal abnormalities in long bones, such as widening, uneven contours, and defects.

DXA examination in 16 patients revealed decreased bone mineral density in 13. Genetic testing conclusively identified ALPL gene mutations, confirming the diagnosis in all patients.

**Conclusions:** The diagnosis of hypophosphatasia is frequently delayed due to the rare occurrence of this disease and the diversity of clinical manifestation. The symptoms of hypophosphatasia depend on the patient's age and the form of the disease. It is advisable to include the assessment of alkaline phosphatase activity as a routine test for patients exhibiting complaints and symptoms associated with the musculoskeletal system.

### P186

### Access to physical therapy for children with osteogenesis imperfecta in Austria

Magdalena Jablonska[1,2], Gabriel T Mindler[3,2], Adalbert Raimann[1]

[1]Medical University of Vienna, Vienna, Austria. [2]Vienna Bone and Growth Center, Vienna, Austria. [3]Orthopedic Hospital Speising, Vienna, Austria

### Abstract

OBJECTIVES:

Physical therapy is a crucial component in the multidisciplinary care for pediatric patients with chronic bone disorders. Similar to other European regions, most patients with rare bone disorders in Austria rely on the availability of extramural therapists. However, access to physical treatments can be limited due to factors such as long waiting times and lack of public insurance coverage. Structured data on accessibility and burdens to receive physical treatment is lacking in many European regions. This study aims to depict the actual situation for pediatric patients with OI in Austria as a sample region.

METHODS:

Physical therapists of the official Austrian registry (Physio Austria) have been contacted with a standardized call for therapeutic consultation with a 5 year old patient with Osteogenesis imperfecta type I. Waiting time to the first appointment and the cost of treatment will be documented and analyzed regarding regional differences.

RESULTS:

483 functional therapists across Austria with declared focus on pediatrics have replied to an initial request. Nationwide, 21 physiotherapists (4%) offered an appointment on public health insurance plans with an average waiting time of 50 days. 263 physiotherapists (55%) offered a private appointment with an average waiting time of 36 days. 62 (13%) rejected any offer due to the diagnosis of the sample patient, the remaining 137 (28%) declined for other or unspecified reasons.

CONCLUSIONS:

The results of this confirm reported difficulties faced by families of children with chronic bone disorders in accessing functional treatments. Despite the obvious differences in national health care systems, similar findings can be expected in other European regions. Long waiting times and high costs could prevent children from receiving necessary treatments with negative effects on their physical and mental health. By identifying regional differences, this aims to raise awareness and to improve access to physical treatments for children with chronic bone disorders.

### Disclosure

AR: Consultancy and lecturing Kyowa Kirin, not associated to presented data

### P187

### Serum total osteocalcin concentration mainly depends on the spine DXA Z-score in children with osteogenesis imperfecta type I and III treated with sodium pamidronate

Jakub Nowicki, Anna Maćkowska, Elżbieta Jakubowska-Pietkiewicz

Department of Paediatrics, Neonatal Pathology and Metabolic Bone Diseases, Medical University of Lodz, Lodz, Poland

### Abstract

OBJECTIVES:

Osteocalcin (OC) is routinely determined as a clinical marker in the diagnosis of diseases of bone metabolism. One of the diseases in which OC levels are routinely determined is osteogenesis imperfecta (OI). The aim of the study was to identify the metabolic and skeletal parameters on which OC levels are most influenced in children with OI types I and III treated with sodium pamidronate.

METHODS:

The results of 53 patients hospitalised in the Department of Paediatrics, Neonatal Pathology and Metabolic Bone Diseases at the Medical University of Lodz with a diagnosis of OI were included in the analysis. All patients had COL1A1 and COL1A2 genes mutations, and all were treated with sodium pamidronate. Age, sex, clinical type of OI, number of pamidronate sodium cycles received, number of long bone fractures, serum concentrations of total OC, leptin, glucose, alkaline phosphatase, vitamin D3, centiles of weight, height and BMI, Z-score DXA scores in the Total Body and Spine protocols, and body fat percentage and fat mass/height ratio were analysed.

RESULTS:

OC concentration differed statistically significantly by OI type I vs. III (95 vs. 39 [ng/ml], $p<0,05$). Positive, statistically significant correlation coefficients were obtained between OC levels and age ($r=0.29$), leptin concentration ($r=0.36$), body weight centile ($r=0.46$), height centile ($r=0.49$) and spine DXA Z-score ($r=0.41$). The other parameters included in the analysis showed no statistically significant relationship.

In the linear regression model analysis, after excluding the type of OI due to correlation with the other parameters, it was shown that OC concentration depends only on the spine DXA Z-score. An increase in this score by 1 results in an increase in osteocalcin concentration by an average of 15 ng/ml (SE=5, $p<0.05$).

CONCLUSIONS:

Serum total OC concentration is an important marker of bone turnover that directly correlates with the spine DXA Z-score in a group of children with OI. The other parameters assessed in this study have little or no effect on the concentration of the assessed marker in the selected group. Analysis of OC concentrations can make an important contribution to analysing the effectiveness of OI treatment.

### P188

### Lasting effect of Burosumab on Quality of life and Pain improvement on children with X-linked hypophosphataemia (XLH)

Robyn Gilbey-Cross[1], Moira Cheung[2], Jessica Sandy[3,4], Emma Snow[1], Alessandra Cocca[1], Sophia D Sakka[5]

[1]Evelina London Children's Hospital, London, UK [2]Great Ormond Street Children's Hospital, London, UK [3]Chidlrens Hospital Westmead Clinical School Faculy of Medicine and Healht, Sydney, Australia. [4]Institute of endocrinology and Diabetes The children's Hospital Westmeadd, westmead, Australia. [5]First Department of Paediatrics, Athens University Medical school, 'Aghia Sophia' Children's hospital, Athens, Greece

### Abstract

**Objectives:** Burosumab, a monoclonal antibody that therapeutically targets the underlying elevated levels of fibroblast growth factor 23 (FGF23) in X-linked hypophosphateamia (XLH), has been available to children out of trial conditions for a number of years. We previously described the effect of Burosumab on pain, quality of life and functionality of children on treatment for 12 months. We have now extended our observations for a duration of treatment of 4 years.

**Methods**: Questionnaire tools were completed at baseline and 48 months for 10 children with XLH receiving Burosumab at the Evelina London Children's Hospital. Questionnaire tools used included: Pain Severity Score (PSS) and PIS (Pain Intensity Score) from the Brief Pain Index (BPI), Core Paediatric Quality of Life Inventory (PedsQL Core), and Paediatric Quality of Life multidimensional fatigue scale (PedsQL Fatigue). Paired t-test was used to compare scores between baseline and 48 months.

**Results:** The mean age at the beginning of treatment was 6 years. There was no statistical significance between baseline and 48months for PIS (1.36 vs 1.41, p=0.951), but there was significant improvement of PSS between baseline and 48 months (2.03 vs 0.575, p=0.009). PedsQL Core score showed significant improvement at 48 months (80,15 at 48months vs 65 at baseline, p=0,042). Also, PedsQL Fatigue score showed significant improvement between baseline and 48months (69,4 vs 78,1, p=0.03, only 4 patients).

**Conclusion:** XLH is a condition that affects not only the biochemistry and radiology of children, but most importantly the quality of life causing pain and fatigue. Quality of life and pain scores are improved after four years of Burosumab therapy in children with XLH, suggesting a lasting impact on overall health and wellbeing for children with XLH and a beneficial effect of long term Burosumab treatment. These results reinforce the favourable effect of Burosumab on children with XLH and the need for continuous treatment.

### Disclosure

Sophia Sakka has received honoraria for delivering presentations from Kyowa Kirin and Genesis. Dr Cheung has received honoraria for advisory boards and delivering presentations from Kyowa Kirin.

### P189

### Tumor-induced osteomalacia: A rare association with newly diagnosed hepatoblastoma

Jonathan Bowden[1], Nat Nasomyont[2]

[1]University of Cincinnati College of Medicine, Cincinnati, USA [2]Cincinnati Children's Hospital Medical Center Division of Endocrinology, Cincinnati, USA

### Abstract

**Background:**

Tumor-induced osteomalacia (TIO) is a rare paraneoplastic syndrome characterized by hypophosphatemia, bone pain, fractures, and muscle weakness. It is typically caused by an overproduction of fibroblast growth factor 23 (FGF23) in small mesenchymal tumors, which leads to renal phosphate wasting and impaired bone mineralization. The biochemical hallmark of TIO is elevated FGF23 and normal/low 1,25-dihydroxy vitamin D level despite hypophosphatemia. While incidental fractures can be observed, TIO has not been reported in children with newly diagnosed hepatoblastoma.

**Presenting problem:**

An 18-month-old female with trisomy 18, congenital heart disease, and newly diagnosed hepatoblastoma presented with multiple fractures incidentally found on her diagnostic imaging for hepatoblastoma. She had multiple rib fractures of various stages of healing and multiple vertebral compression fractures. Metabolic bone evaluations revealed persistent hypophosphatemia (serum phosphorus <4.0 mg/dL; normal 4.3-7.4 mg/dL), elevated bone alkaline phosphatase (871 U/L; normal 0-189 U/L), normal serum calcium, normal 25-hydroxyvitamin D (52.5 ng/mL), low-normal 1,25-dihydroxyvitamin D (52.7 pg/mL; normal 47.1-151 pg/mL), markedly elevated serum FGF23 at 857 RU/mL (normal ≤230 RU/mL), and low tubular reabsorption of phosphate at 30%, consistent with FGF23-mediated hypophosphatemia/TIO.

**Clinical management:**

The patient was treated with cisplatin, followed by surgical resection of the tumor. She was started on phosphorus supplementations and calcitriol for hypophosphatemia. One month after tumor resection, she sustained new vertebral compression fractures and had persistently elevated FGF23 (838 RU/mL). Two months after resection, serum phosphorus and FGF23 normalized (187 RU/mL). Subsequently, calcitriol and phosphate doses were tapered and discontinued shortly after, as her phosphorus levels remained within normal range. Follow-up radiographs revealed healing rib fractures with no new vertebral compression fractures.

**Discussion:**

Hepatoblastoma is the most likely culprit of elevated FGF23, leading to TIO. Even though TIO eventually resolved after tumor resection, the FGF23 level remained elevated for one month despite no evidence of residual tumor. To our knowledge, this is the first case report of TIO secondary to hepatoblastoma in a very young child. Further study into the association between liver tumors and FGF23 is needed.

### P190

### Digging family history to uncover X-linked hypophosphatemia

Stefano Mora, Katia Maruca, Ruggero Lanzafame, Elisa Sala, Ludovica Crocè, Marco Pitea

IRCCS Ospedale San Raffaele, Milano, Italy

### Abstract

OBJECTIVES: X-linked hypophosphatemia (XLH) is the commonest inherited form of rickets. Patients with XLH exhibit rickets and osteomalacia, severe deformities of the lower limbs, bone and muscular pain, stunted growth, and reduced quality of life. The disease is reported to be familial in the majority of the cases, but not infrequently sporadic cases are reported. In familial cases, mild phenotypes may go unrecognized and therefore it is difficult to understand the frequency of the disease. To address this issue we thoroughly investigated all the families of the patients we currently follow in our unit.

METHODS: The patients with XLH followed in our Center and their family members underwent specific interviews to build wider and accurate pedigrees. The family analysis was extended as far as the recollection could go. We interviewed each family to assess the presence of affected members, asking specific questions regarding growth, height, bowing of the legs, articular and muscular pain, presence of odontostomatologic problems, weakness, fatigue, among others. We employed a specific online tool (Genetic Health Map tool: https://it.gh-map.com), which is able to store information regarding the phenotype, and to indicate the subjects that are likely to be affected based on the inheritance pattern.

RESULTS: We contacted 15 parents of children with XLH for participation. Eleven families agreed to participate and 5 of them revealed familial recurrence of the disease (Figure). Molecular analyses revealed 2 nonsense, 1 splicing, 1 duplication, and 1 deletion variants in the *PHEX* gene in 15 subjects. The interviews revealed the presence of 31 family members showing signs of XLH and 2 likely affected individuals. Moerover, we could assemble data from two large pedigrees, in which the disease is particularly frequent.

CONCLUSIONS: XLH is often underdiagnosed due to its large phenotypic variability. Our study demonstrates that a specific and thorough investigation may lead to reveal the presence of XLH in previously undiagnosed subjects. The use of a specific tool is also useful to find likely affected individuals who are paucisymptomatic.

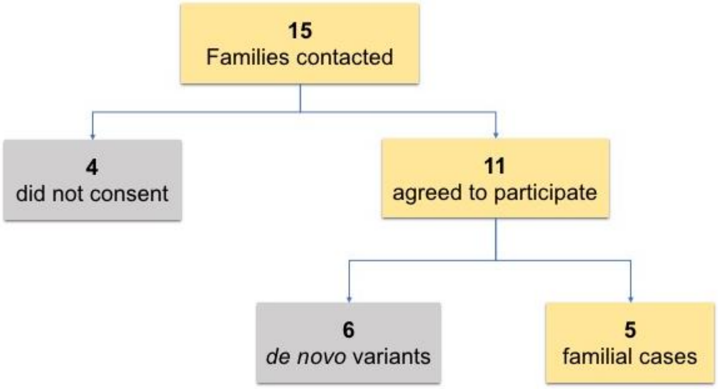

**P191**

### Two sisters, two different causes of hypocalcemia

Ruggero Lanzafame, Elisa Sala, Ludovica Crocè, Giorgia Piselli, Gabriele Drago, Stefano Mora, Marco Pitea

IRCCS Ospedale San Raffaele, Milano, Italy

### Abstract

BACKGROUND: Hypocalcemia in pediatric patients may present as asymptomatic or manifest with a variety of symptoms. We present two cases of hypocalcemia in two sisters leading to two different diagnoses, demonstrating the diverse clinical presentations of this condition.

PRESENTING PROBLEM (I): An 11-year-old Pakistani girl was admitted to the ER for a five-minute seizure episode characterized by unresponsiveness, upper limb clonus and sialorrhea.

CLINICAL MANGEMENT (I): Blood tests showed calcemia 5.48 mg/dL, phosphoremia 5.01 mg/dL, 25-OH vitamin D < 3 ng/ml, magnesium within limits, PTH 601 ng/ml. Acute treatment was started with 0.5 mL/kg of intravenous calcium gluconate, followed by a maintenance infusion. ECG showed QTc prolongation; EEG showed no epileptiform abnormalities; wrist and lower limbs X-ray showed no signs of rickets; negative celiac disease screening. After a few days she was discharged with the indication to continue treatment with calcium and cholecalciferol. In the follow-up she showed a resolution of hypocalcemia and hypovitaminosis D, with persistence of high PTH values (>100 ng/mL): a methylation analysis of the *GNAS* gene was therefore carried out, leading to the diagnosis of *pseudohypoparathyroidism*. Calcitriol and calcium therapy was started, with subsequent normalization of PTH values.

PRESENTING PROBLEM (II): One month later, her 13-year-old sister was brought to our attention for perioral and upper limbs paresthesias.

CLINICAL MANAGEMENT (II): Blood tests showed hypocalcemia, hypovitaminosis D, hyperparathyroidism and an iron deficiency microcytic anemia. Calcium, cholecalciferol and iron therapy was administered during hospitalization; *celiac disease* screening came out positive. X-ray of wrist and lower limbs showed no frank signs of rickets. She was discharged with instructions to start a gluten-free diet and to continue calcium, vitamin D and iron therapy. In the subsequent follow-up there was a progressive resolution of the anemia and hyperparathyroidism as the blood values of vitamin D and calcium normalized.

DISCUSSION: The main symptom of acute hypocalcemia is neuromuscular irritability, which can lead to paresthesias, muscle cramps, and even seizures and laryngospasm. Diagnostic investigations are essential to undertake a specific therapy in order to prevent the recurrence of subsequent episodes of acute hypocalcemia in the future.

### P192

### Col1A-2 mutation alters tendon properties in the osteogenesis imperfecta mouse (oim)

Antoine Chretien[1], Grégoire André[1], Julia Vettese[2], Pierre-Louis Docquier[2], Catherine Behets[1]

[1]Pole of Morphology, Institute of Experimental and Clinical Research, UCLouvain, Brussels, Belgium.
[2]Neuromusculoskeletal lab, Institute of Experimental and Clinical Research, UCLouvain, Brussels, Belgium

### Abstract

Introduction and objective: Osteogenesis imperfecta (OI) is a genetic disorder of connective tissue mainly due to a mutation of *COL1A1* or *COL1A2* gene. The patients present bone fragility, fractures, as well as extra-skeletal manifestations. Tendon ruptures have been reported in OI patients; indeed, these dense, regular connective tissue structures are mainly composed of type I collagen. The objective of the present work was to investigate the tendon properties in the osteogenesis imperfecta mouse (oim), a validated model of human type III OI, characterized by a mutation in the *COL1A2* gene.

Methods: The tendons of 17-week-old oim and control (WT) mice (n=15 for both groups) were compared using tensile test, contrast-enhanced computed tomography (CECT), polarized light microscopy, immunohistochemistry, and Raman spectroscopy.

Results: The mechanical tests highlighted a significant impairment of viscoelastic properties in oim flexor digitorum longus (FDL) tendons, as compared to WT ones. Moreover, the measurement of tendon density was significantly lower in oim than WT tail tendons. In CECT analysis, the collagen fibre thickness was higher and more variable in oim FDL tendons than WT ones, while the difference was not statistically significant. The triceps brachii tendons were significantly less birefringent under polarized light microscopy in oim than WT mice. The cell density was significantly higher, with a high proportion of chondrocyte-like cells. This observation was confirmed by the significantly higher proportion of Sox-9 positive cells in oim than WT tendons. Raman spectroscopy highlighted a significant difference for the three biomarkers 3243/2940, 3333/2940 and 3457/2940, indicating a lower proportion of bound water in oim FDL tendons than in WT ones. However, there was a weak correlation between these ratios and the mechanical properties.

Conclusion: This work highlighted several alterations of tendon organization in the oim mouse. They could be associated with pain and musculoskeletal disorders in OI patients and deserve clinical investigations.

*Keywords: osteogenesis imperfecta, oim, tendon, collagen.*

This research was supported by the French-speaking Belgian Association of the Osteogenesis Imperfecta (www.fetealavie.be/, www.afboi.be/).

### P193

### Acrodysostosis: A Rare condition

Ludovica Crocè, Elisa Sala, Ruggero Lanzafame, Giorgia Piselli, Gabriele Drago, Stefano Mora, Marco Pitea

IRCCS Ospedale San Raffaele, Milano, Italy

### Abstract

BACKGROUND:

Acrodysostosis is characterized by adult short stature, brachydactyly, facial dysostosis and multiple hormonal resistances, always to PTH and TSH. These patients are often born SGA. Diagnosis is based on clinical presentation, blood tests and genetic analysis (mutations of the *PRKAR1A* and *PDE4D* genes). Since this is a rare and heterogenous condition, the management of these patients must be based on a multidisciplinary approach.

PRESENTING PROBLEM:

A 2.6-year-old girl of Egyptian origins comes to our attention due to a deflection of the growth rate. The girl was born small for gestational age (SGA), and is affected by congenital hypothyroidism treated with Levothyroxine, with normal sized thyroid.

At the age of 1.8y an x-ray of pelvis and lower limbs was performed, showing absence of the distal tibial ossification nucleus in the right leg and entanglement of the trabecular design of the distal metaphyseal region; she underwent magnetic resonance imaging of the ankles, showing distal meta-epiphyseal dysmorphism in the right tibia, with dysmorphic and irregularly ossified epiphyseal nucleus. These findings were seen to a lesser extent contralaterally.

CLINICAL MANAGEMENT:

Her clinical examination revealed brachydactyly with short phalanges, saddle nose, round face, asymmetry in the lower limbs, attitude in flexion of the right elbow.

The x-ray of the left hand-wrist confirmed the presence of brachydactyly with short and dysmorphic phalanges, while the x-ray of pelvis and lower limbs showed absence of the distal tibial ossification nucleus in the right leg and entanglement of the trabecular design of the distal metaphyseal region (figure).

Blood tests showed PTH 121 pg/ml (normal range: 15-65), calcium, phosphate, and alkaline phosphatase within normal ranges, 25-hydroxy-vitaminD 33.9 ng/ml (lower limit of normal >20), TSH 23.1 microUI/ml (nr: 0.36-3.74) with Ft4 1.44 ng/dl (nr: 0.76-1.46), and negative antibodies. Therefore, we adjusted the L-thyroxine posology, and started the treatment with alfacalcidiol and calcium.

CONCLUSION:

The clinical presentation of the girl was found compatible with congenital dysostosis or pseudohypoparathyroidism. Thus, genetic analysis (NGS) for genes related to pseudohypoparathyroidism and bone dysplasia was performed, revealing a heterozygous variant of the *PRKAR1A* gene, classified as pathogenic and associated with acrodysostosis

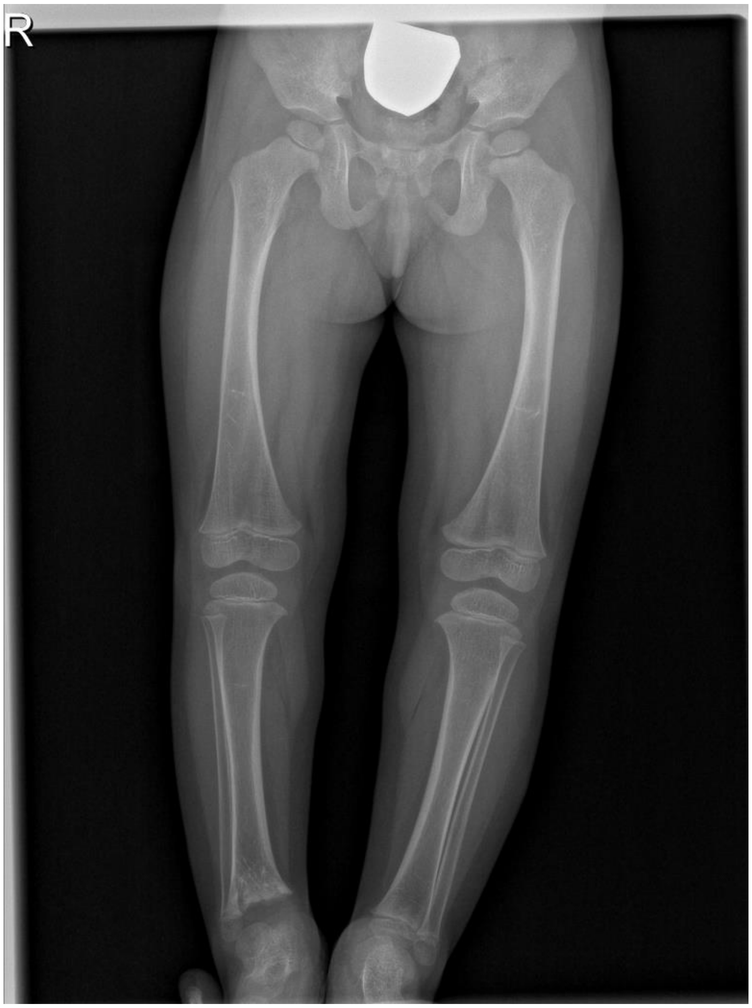

### P194

### Hypophosphatasia: An extremely heterogeneous pathology

Elisa Sala, Ruggero Lanzafame, Ludovica Crocè, Katia Maruca, Marco Pitea, Stefano Mora

IRCCS Ospedale San Raffaele, Milano, Italy

### Abstract

BACKGROUND:

Hypophosphatasia (HHP) is a rare inherited disease caused by loss-of-function variants in *ALPL* gene which encodes for the enzyme tissue non-specific alkaline phosphatase (TNSALP). The disease causes a defect in bone and teeth mineralization and in severe cases to the development of vitamin B6 responsive seizures. The clinical phenotype may range from complete absence of bone mineralization resulting in fetal death, to spontaneous fractures, seizures, premature teeth loss and nephrocalcinosis in adulthood.

PRESENTING PROBLEM:

The presentation and clinical evolution are extremely heterogeneous, and no phenotype-genotype correlation has yet been established. In this report we describe the variability at clinical presentation in our patients undergoing treatment with asfotase alfa.

CLINICAL MANAGEMENT:

In the first family the index case was a three-year-old girl who presented teeth loss and bone deformities. Her blood test revealed low serum alkaline phosphatase and elevated vitamin B6. The X-ray demonstrated metaphyseal irregularities and demineralization. Sequencing analysis of *ALPL* gene revealed the compound heterozygous mutations p. E191K (c. 571G>A) and p.G420S (c.1258G>A), pathognomonic for hypophosphatasia. The older sister have the same genotype without any clinical features.

In the second family the diagnosis was done through genetic analysis performed on a fetus with major bone malformations. The exome sequencing has shown two variants in compound heterozygosity (c.407G>A and c.1489T>C) in the *ALPL* gene associated with hypophosphatasia. The parents were identified as unaffected carriers, both having a single variant in heterozygosity. At the following pregnancy, the fetus did not present any skeletal deformities, but the genetic analysis revealed the same variants. At birth the baby had no clinical characteristics suggestive of hypophosphatasia, but blood test confirmed low alkaline phosphatase activity and high serum vitamin B6. Radiographs showed diffuse hypomineralisation and metaphyseal irregularities.

DISCUSSION:

These two families are an example of the extreme clinical variability which characterizes hypophosphatasia. A database currently exists to assist the clinician in defining the pathogenicity of a mutation, but it would be desirable to collect as much clinical data as possible to better define clinical symptoms, outcomes and a possible correlation with the genetic profile.

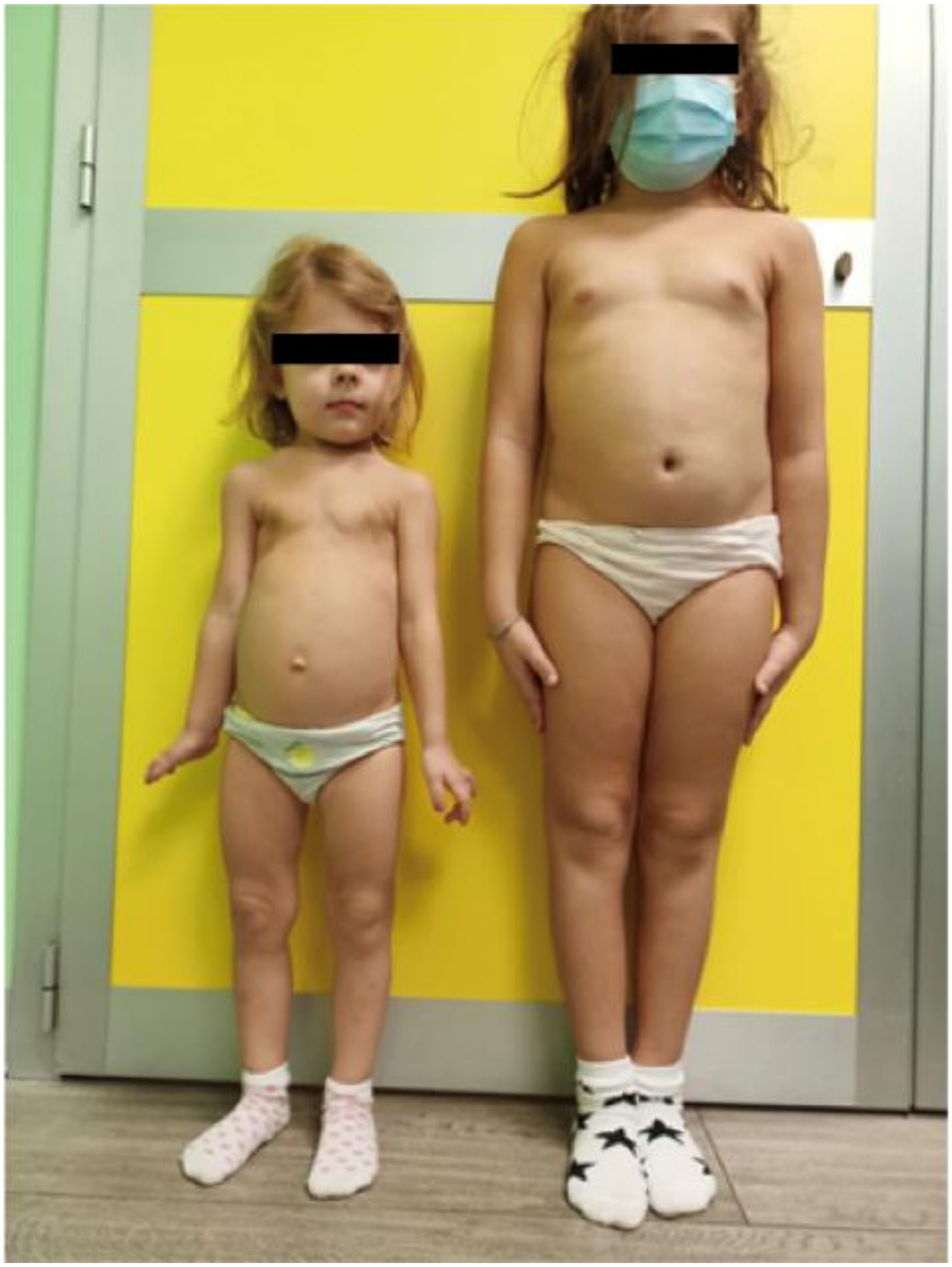

### P195

### Three cases of ENPP1 gene mutations causing Autosomal Recessive Hypophosphataemic Rickets type 2 and generalised arterial calcification of infancy

Jessica Sandy[1,2], Lucy Collins[3], Kate Lomax[4], Sarah Black[4], Fiona McKenzie[5], Craig Jefferies[6], Wendy Hunter[7], Peter Simm[8], Christine Rodda[9], Craig Munns[10], Andrew Biggin[2], Aris Siafarikas[4]

[1]University of Sydney, Sydney, Australia. [2]The Children's Hospital at Westmead, Westmead, Australia. [3]Royal Children's Hospital, Melboure, Australia. [4]Perth Children's Hospital, Perth, Australia. [5]University of Western Australia, Perth, Australia. [6]Starship Children's Hospital, Aukland, New Zealand. [7]Te Whatu Ora, Nelson Marlborough, Nelson, New Zealand. [8]Royal Children's Hospital, Melbourne, Australia. [9]University of Melbourne, Melbourne, Australia. [10]University of Queensland, Brisbane, Australia

### Abstract

Autosomal recessive hypophosphataemic rickets type 2 (ARHRT2) and Generalised Arterial Calcification of Infancy (GACI) are rare disorders caused by mutations in Ectonucleotide Pyrophosphatase/Phosphodiesterase 1 gene (ENPP1).

GACI presents at birth or during early infancy with widespread arterial calcification, resulting in high mortality. ARHRT2 presents in childhood with hypophosphataemia and rickets. Individuals with GACI in infancy may also develop clinical features of ARHRT2 later in childhood. Treatment of ARHRT2 is with activated vitamin D (calcitriol or alfacalcidol) and multiple daily doses of oral phosphate. This treatment causes side effects and can lead to complications including nephrocalcinosis.

We present three cases below and summarised in the table.

Case 1: 5y male presents with 12-month history of worsening valgus deformity and leg pain with radiological signs of rickets. Parents were consanguineous, and older sister had rickets. Biochemistry is summarised in the table. Improvement was seen with oral calcitriol and phosphate. Genetics confirmed homozygous pathogenic *ENPP1* variant (both parents carriers) and heterozygous pathogenic *ALPL* variant consistent with hypophosphatasia (paternally derived). Older sister had both genetic mutations. Further history revealed loss of complete teeth with intact roots. Further investigation revealed aortic/pulmonary arterial calcification and bilateral medullary nephrocalcinosis, currently not requiring treatment.

Case 2: Antenatal diagnosis of *ENPP1* mutation due to older affected sibling (died at 4d from GACI) with maternal etidronate treatment from 29-weeks' gestation. After birth, extensive arterial calcifications were present, managed with bisphosphonates until 8 months. These calcifications gradually improved, but she subsequently developed rickets, nephrocalcinosis (likely due to calcitriol treatment), internal carotid narrowing, and increased renal artery resistive indexes.

Case 3: 7y female presented with knee pain, fixed bilateral flexion knee deformities, limited cervical spine movement, and thickened left clavicle. Investigations are summarised in the table. Homozygous, likely pathogenic *ENPP1* mutation was confirmed. She was managed with calcitriol and phosphate. She developed nephrocalcinosis requiring potassium citrate therapy. Arterial calcification has not been documented, and blood pressure remains normal.

These cases add to the phenotypic spectrum of this rare condition and highlight diagnostic and therapeutic challenges faced by clinicians.

Table: Summary of three cases with ENPP1 mutations

| | Case 1 | Case 2 | Case 3 |
|---|---|---|---|
| Age/ Sex<br><br>Referring complaint | 5-year-old male<br><br>Rickets (at 5y) | 6-year-old female<br><br>Antenatal diagnosis | 11-year-old female<br><br>Lower limb pain and deformity (at 7y) |
| Background:<br><br>Family/social history<br><br>Antenatal/neonatal history | • Ethnicity: Afghani<br>• Parental consanguinity<br>• Older sister: short stature, leg bowing, bone/joint pain<br>• Born in Pakistan<br><br>• Term baby<br>• Macrocephaly, poor weight gain<br><br>Development normal except walked at 2 years | • Ethnicity: Indian<br>• No parental consanguinity<br>• Older child (female) deceased at 4 days from GACI complications which was diagnosed postnatally<br><br>• Antenatal diagnosis<br>• Treated with etidronate from 29 weeks gestation<br>• Born via LSCS at 37 weeks<br>• Birthweight 2.55kg | • Ethnicity: Cambodian<br>• Mother and sister report leg pain<br><br>• Term infant<br>• Normal growth and development<br>• Sore legs and abnormal gait |
| Biochemistry at diagnosis | • ALP 170U/L (120-370)<br>• Phosphate 0.61mmol/L (1.1-2.0)<br>• PTH 11mpol/L (1.6-9.0))<br>• Normal vitamin D<br>• TmP/GFR 0.583 (low) | N/A (Antenatal diagnosis) | • Calcium 2.3mmol/L (2.2-2.6)<br>• Phosphate 0.7mmol/L (1.0-2.0)<br>• ALP 564IU/L (80-360)<br>• PTH 7.8pmol/L (1.6-7.0)<br>• 25-OH Vit D 26 (50-150)<br>• Normal urine Ca:Cr ratio |
| Rickets | Yes – presenting complaint | Yes | Yes |

| | | | |
|---|---|---|---|
| Arterial calcification | Yes<br><br>• Aortic and pulmonary arterial calcification | Yes<br><br>• Extensive calcifications present at birth that improved with age:<br>• Cardiopulmonary, thoracic, and abdominal aortic vessels<br>• Calcification of ligamentum flavum, intervertebral discs, posterior cervical spine bodies<br>• Peri-articular calcification of right shoulder limiting movement and causing radial nerve palsy<br><br>• Subsequent development of:<br>• Internal carotid narrowing<br>• Increased renal artery resistive indexes. | No, not documented to date |
| Nephrocalcinosis | Yes | Yes | Yes |
| Other Clinical features | • Dental: Loss of multiple complete teeth with root intact<br>• Valgus deformity of lower limbs<br>• Knee pain<br>• Abnormal gait<br>• Short stature | • Dental: Generalised dental hypoplasia<br>• Unilateral coronal craniosynostosis<br>• Conductive hearing loss requiring hearing aids<br>• Mild speech delay | • Dental: Dental crowding<br>• Knee pain<br>• Abnormal gait<br>• Limited flexion/extension cervical spine<br>• Thickened left clavicle<br>• C2-C5 fused posteriorly via posterior longitudinal ligament<br>• Metaphyseal end of clavicle and 1st rib splayed and bowed |

| | | | |
|---|---|---|---|
| Genetics | • Homozygous pathogenic ENPP1 variant (AHRH type 2): c.783C>G p.(Tyr261*)<br>o Sister also affected, parents carriers<br>• Heterozygous pathogenic ALPL variant<br>• Sister also affected, father carrier | • Homozygous pathogenic ENPP1 mutation (GACI): c.749C>T p.(Pro250Leu) | • Homozygous, likely pathogenic ENPP1 mutation (AHRH type 2): C.403_404del.p.(Asp135Leu) |
| Management | • Calcitriol and oral phosphate with normalisation of phosphate levels<br>• No management required of arterial calcification or nephrocalcinosis | • Bisphosphonates from birth until 8 months (pamidronate then risedronate, ceased due to social reasons)<br>• Phosphate and calcitriol once diagnosed with rickets at 2y<br>• Aspirin (commenced at 5y due to internal carotid narrowing) | • Calcitriol (60ng/kg/day◊ 20ng/kg/day) and phosphate (40mg/kg/day)<br>• Potassium citrate 300mmol TDS (nephrocalcinosis)<br>• Iron supplementation |

### P196

### Osteosclerotic metaphyseal dysplasia associated with fragility fractures & osteomyelitis of the mandible

Aysha Shattaf[1], M Zulf Mughal[1], Shehzanaa Fatima[2]

[1]Al Jalila Children's Hospital, Dubai, UAE. [2]Rashid Hospital, Dubai, UAE

### Abstract

### Background:

Osteosclerotic metaphyseal dysplasia (OSMD) is an ultrarare sclerosing skeletal dysplasia characterized by osteosclerosis of the metaphysis of long bones and vertebrae. OSMD is caused by biallelic pathogenic variants in the leucine-rich repeat kinase 1 (*LRRK1*: OMIM 610986 ) gene, which is crucial for bone remodelling and maintenance. It is an osteopetrosis-like diseases characterized by short stature and recurrent fragility fractures. While the skeletal manifestations of OSMD have been well-documented, the potential dental complications, specifically the risk of chronic osteomyelitis of the mandible/jaw following dental extraction, have received limited attention in the literature.

### Presenting Problem:

We present the case of a child with OSMD with confirmed homozygous pathogenic variant in the *LRRK1* gene: c.2404_2405+1delCAG p.(His802*). The patient, a 10-year-old boy was referred to the metabolic bone disease clinic with history of fragility fractures. At the age of 7 years he suffered fracture of right Radius (FIG 1) following a trivial injury. There was slow healing of the fracture and he was left with a residual deformity. Following dental exactions by a community dentist, he developed swelling of right side of the maxillary area. He had halitosis and the clinical examination of the oral cavity revealed thickening of gums and bony tissue in right upper part of oral cavity, His height Z score -1.2,weight -0.87 . CT scan of the fascial bone revealed Periosteal thickening along the alveolar process of right maxillary bone, bone erosions seen at the inner wall and roof of alveolar process of right maxilla this finding are inconsistent with chronic osteomyelitis of right maxilla.

### Clinical Management:

There is no medical treatment for OSMD. Like many osteoclast-rich forms of osteopetrosis, patients with OSMD may be amenable to treatment by hematopoietic stem cell transplantation. The management is essentially involves minimizing activities that might lead to fractures and looking out for complications associated with OSMD. The osteomyelitis of the maxilla in our patient was treated with antibiotics, surgical debulking and hyperbaric oxygen. Given the increased susceptibility to infections and osteomyelitis of the maxilla, meticulous dental hygiene is of paramount importance.

**Discussion:**

This case underscores the importance of genetic diagnosis in unravelling rare skeletal disorders such as OSMD and emphasizes the necessity of tailored clinical management. It also highlights the previously underreported osteomyelitis of the mandible following dental extraction. .

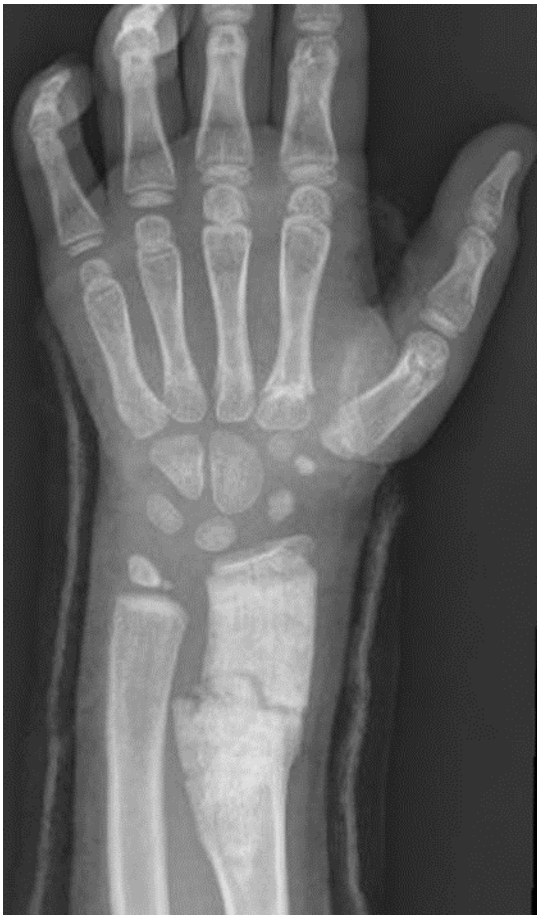

### P197

### Growth response to Vosoritide in achondroplasia – real world experience from a tertiary centre in the UAE

Anju Jacob[1,2], Manal Mustafa[1], Mohamed Elabiary[1], M Zulf Mughal[1], Nandu Thalange[1]

[1]Al Jalila Children's Specialty Hospital, Dubai, UAE. [2]Mohammed Bin Rashid University, Dubai, UAE

### Abstract

#### Introduction

Achondroplasia is an autosomal dominant skeletal dysplasia caused by activating mutations in the Fibroblast Growth Factor Receptor 3 (FGFR3) gene. This results in severe short stature with a final height of 124 to 131 cm. The prevalence of achondroplasia ranges from 0.36 to 0.60 per 10,000 live births. All cases result from de novo mutations in the FGFR3 gene.

Vosoritide, a recombinant C-type natriuretic peptide analogue, a once-daily subcutaneous injection is approved by the FDA and EMA from infancy for use from birth for children with achondroplasia.

#### Objectives

To assess the growth response in children with achondroplasia treated with Vosoritide (weight dependent dosing).

#### Methods

We assessed the linear growth response expressed as standard deviation score (SDS) of children who received Vosoritide treatment for at least 1 year. Their height was plotted on the Height SDS chart compared to the population. We also looked at linear height SDS before, 6 months and 12 months after Vosoritide treatment.

#### Results

We had 8 patients in our hospital, of which 5 were on Vosoritide treatment for over 6 months and were followed up at our hospital. The treatment outcomes in height in this cohort (see Figure 1). Vosoritide has a clear beneficial effect on growth reflected in increased height SDS which is slow and gradual as seen in our patient cohort except one who had poor response currently undergoing investigation for growth hormone deficiency.

In the first year of Vosoritide treatment in our cohort, SDS range increment was + 0.24 to + 0.87 SDS.

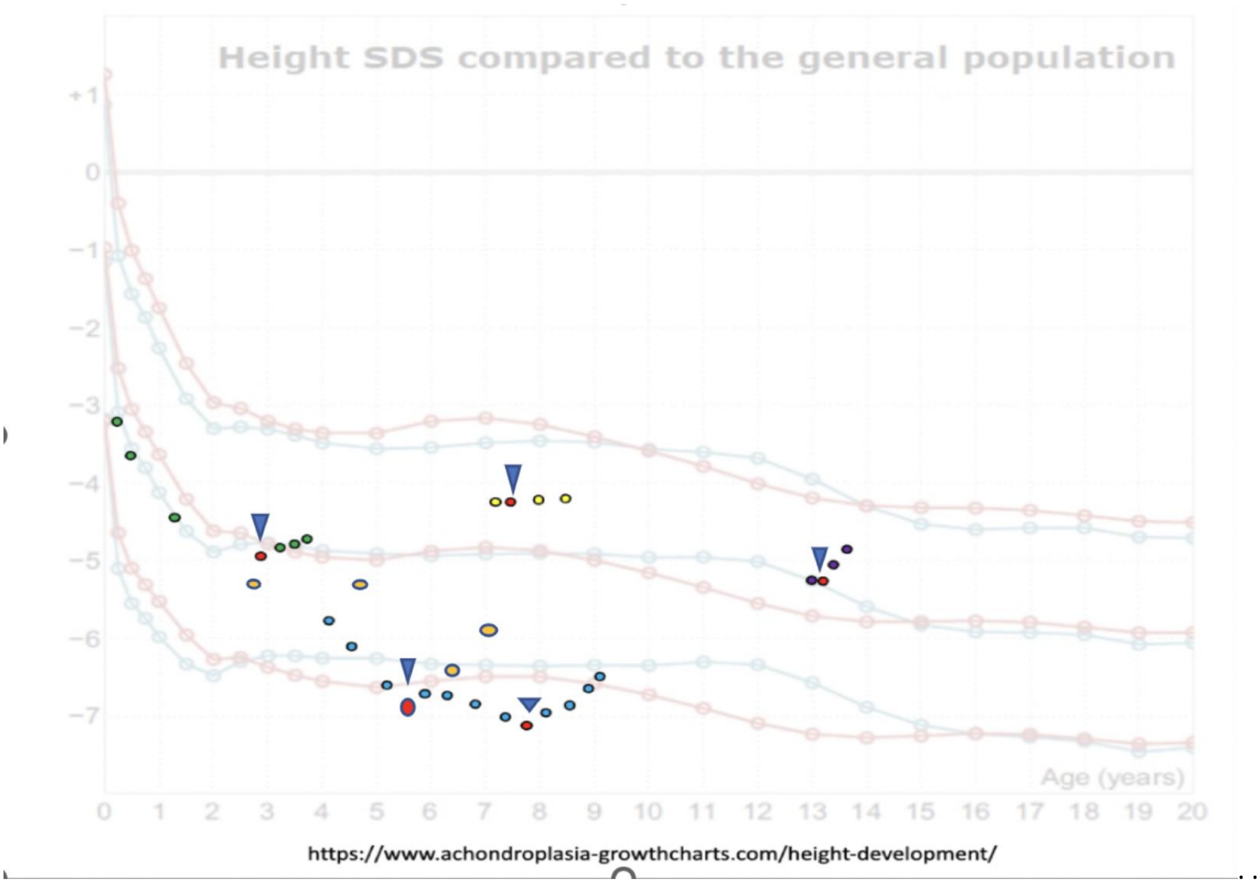

Fig1. Height SDS in achondroplasia children compared to general population (Green – Girl 1, Blue- Girl 2, Yellow- Girl 3, Orange – Girl 4, Purple- Boy 1, Red and blue arrow – Initiation of Vosoritide treatment)

**Conclusions**

Treatment with Vosoritide produced positive growth response in our patients as expected as based on clinical trial data with the exception on case in our cohort with suspected growth hormone deficiency. Whilst the initial results are promising, long term growth outcomes are yet to be determined.

### P198

### Foramina Parietalia Permagna

Stepan Kutilek, Kamila Pecenkova, Martina Vracovska

Department of Paediatrics, Klatovy Hospital, Klatovy, Czech Republic

### Abstract

### Background

Foramina parietalia permagna (FPP; OMIM 168500) are two symmetrical bony defects located at both sides of the saggital suture. Incidence of FPP varies between 1:15 000 and 1:25 000. Foramina parietalia permagna have an autosomal dominant inheritance, loss of function mutations in chromosome 5 and 11 are responsible for this anomaly, in particular ALX4 or SMX2 homeobox gene mutations. With advancing age FPP tend to become smaller. FPP are usually isolated and asymptomatic, but can be rarely associated with various skeletal abnormalities or vascular variations. Clinical manifestations rarely include headaches, vomiting, pain over unprotected cerebral cortex, and seizures may be also experienced by some of the patients.

### Case Report & Clinical Management

We report two cases of foramina parietalia permagna within one family. A boy of unrelated parents, born on 37th gestational week, body weight 2330grams, length 44 cm, both pregnancy and delivery were uneventful. His mother is followed by a neurologist since early childhood because of FPP. FPP were also noticed on boy´s skull shortly after birth. The skull X-ray confirmed the diagnosis of FPP. There were no other skeletal or soft tissues malformations. Basic biochemical parameters of bone metabolism (calcaemia, phosphataemia, serum alkaline phosphatase activity, parathyroid hormone level) were within reference ranges, ultrasonography of the brain was also normal. The boy remains under our surveillance. At the age of 5 months he was doing well (7200 g weight/62 cm length); the FPP were still palpable (5 mm in diameter each), his psychomotor development is normal. Mother still has palpable FPP. There are no skeletal malformations in the rest of the family. The genetic examination is underway.

### Discussion & Conclusion:

FPP are usually diagnosed incidentally. Back in 1997 we described nine cases of FPP in one family, and later, in 2000, ALX4 mutations were identified in this family. There is no causal treatment for FPP, the patients should protect their skull and avoid headblows. FPP can be occassionally misdiagnosed as craniotabes, tempting the physician to administer high doses of vitamin D. Therefore, paediatricians should be aware of this condition in order to avoid vitamin D intoxication.

### P199

### Allied Health Professionals (AHP) supporting children with skeletal dysplasia moving to secondary school

Paul Watson, Jonathan Gibbins, Kathryn Johnson, Alessandra Cocca

Evelina London Children's Hospital, Guys and St Thomas' Hospital, London, UK

### Abstract

**Background:** Children with a skeletal dysplasia (SKD) face psychological and participatory challenges when they move to secondary school. Their short stature can create barriers to participation in larger school settings where they experience comments and looks from peers. Parents commonly raise concerns about their child coping with this change. Occupational Therapy (OT) and Psychology services offer a joint clinic, providing practical and psychological advice to the child and family. A workshop is then organised for all children to then come together to reflect on their challenges and achievements.

**Objectives:** The secondary school clinic/workshop has been running since 2019. The purpose of this study is to give an overview of this service development, its outcomes, effectiveness and significance for this cohort of children.

**Methods:** This single institution retrospective cohort study analyses data from over 3 consecutive years (2021-2023)

Joint 1:1 OT and Psychology virtual consultations, offered post school place allocation, were underpinned by themes commonly reported by parents and children: environment access, managing self-care, PE, stamina, tiredness, pain, bullying and friendships.

A virtual group workshop was offered to all children and parents after the first term at school. Qualitative feedback from parents and children was collated following each intervention.

**Results:** **46** children were identified to be moving to secondary school. **22** children attended the clinics (12 boys and 10 girls), **18** children attended the workshops (11 girls and 7 boys). Those that turned down the clinics informed us that they already felt they had enough community and SENCO support in place.

**Conclusion**

- Positive qualitative feedback from parents and children highlights the key importance of the role of tertiary AHP services within the clinical field of SKD.
- This service improvement project is an example of the important role tertiary AHP services can play within the clinical field of Skeletal Dysplasia.
- The cohort is too small to draw overall condition specific conclusions.
- Further studies will address the paucity in literature and evidence base regarding the occupational performance, participation and the psychological wellbeing of children as they move to secondary school.

### P200

### Clinical case: Craniosynostosis in hypophosphatasia

Elisa Sala, Ruggero Lanzafame, Ludovica Crocè, Katia Maruca, Stefano Mora, Marco Pitea

IRCCS Ospedale San Raffaele, Milano, Italy

### Abstract

BACKGROUND:

Hypophosphatasia (HPP) is a rare genetic disease caused by mutations with loss of function in the *ALPL* gene, which encodes for non-specific tissue alkaline phosphatase (TNSALP). We report a case of craniosynostosis in infantile hypophosphatasia.

PRESENTING PROBLEM:

Due to positive family history for hypophosphatasia, a chorionic villus sampling was made, with evidence of compound heterozigous variants c.407G>A and c.1489T>C in the *ALPL* gene. At birth the patient did not have clinical complications, however the blood measurements of alkaline phosphatase and vitamin B6 confirmed the diagnosis. On the third day of life, treatment with asfotase alfa has been started at a dose of 2 mg/kg three times a week subcutaneously.

CLINICAL MANAGEMENT:

Starting from the third month of age, a progressive worsening of the cranial conformation has been noted with the appearance of marked asymmetry of the frontal drafts (left > right) and apparent hypoplasia of the left eyebrow arch. The anterior fontanel was, however, of normal size and conformation and the neuromotor development progressed regularly. At 6 months of life, head CT with 3D reconstruction was carried out, with evidence of craniosynostosis of the right coronal suture (Figure). The patient underwent a corrective neurosurgery at the age of 7 months.

DISCUSSION:

As reported in literature, about 2/3 of patients with childhood-onset hypophosphatasia present craniosynostosis as a complication; therefore, it is important to monitor the possible occurrence of this condition and eventually direct patients to a correct multidisciplinary diagnostic and therapeutic process. It is also important that the follow up continues for a long time after the surgical correction because recurrences are more frequent than in patients not affected by HPP.

To date, further studies are needed to determine whether the introduction of enzyme replacement therapy can change the incidence and presentation of this complication. However, it is possible to hypothesize that treatment with asfotase alfa, will improve the quality of bones, contributing to the success of surgical correction, while reducing the risks associated with the pathology.

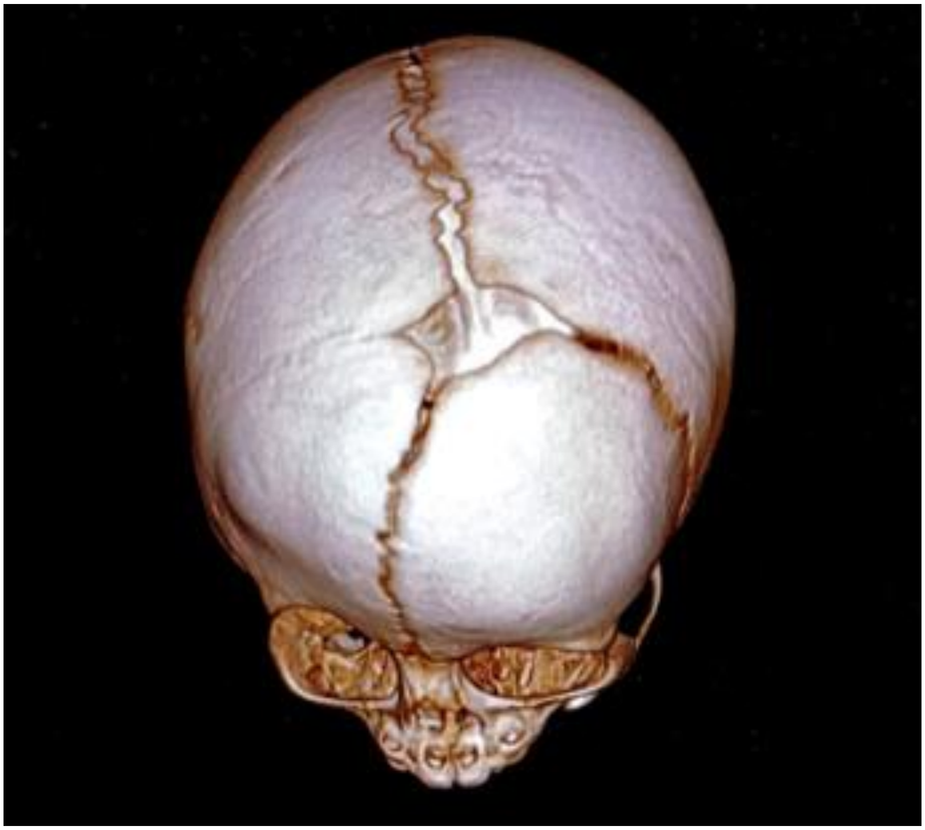

### P201

### Spontaneously improving infantile osteopetrosis

Sarah Ackah, Stuart Warden, Erik Imel, Michael Econs

Indiana University, Indianapolis, USA

### Abstract

**Background:** Osteopetrosis is a rare bone disorder of abnormally dense bone due to impaired osteoclast resorption predisposing to increased fracture risk. The infantile form presents at birth with increased bone mineral density (BMD) and can cause macrocephaly, growth retardation, frontal bossing, exophthalmos, choanal stenosis, and micrognathia, as well as risk of marrow failure.

**Presenting Problem:** A 4-week-old male born at 38+6 weeks with multiple anomalies was transferred for concerns of infantile osteopetrosis. Physical exam was significant for cleft lip and palate, right facial palsy, imperforate anus, and small thumbs, but no frontal bossing or limb deformity. Radiographs on day 2 of life showed diffusely increased density of axial and appendicular skeleton and narrowing of the femur marrow cavity. Laboratory testing showed some mild anemia, leukopenia and thrombocytopenia. Genetic testing indicated a heterozygous variant of uncertain significance of PLEKHM1 (c.864G>A, pMet288Ile) and duplication of chromosome 15q14; both were maternally inherited. The PLEKHM1 variant is predicted by two analysis programs to be benign.

His 31-year-old mother had type 2 diabetes and history of cleft lip repair, but no fractures. During pregnancy she was treated with insulin, labetalol, nifedipine, semaglutide, and prenatal vitamins. Laboratory testing revealed no anemia or cytopenia. DEXA scan performed on the research unit indicated elevated bone density for age lumbar spine Z-score of +4.27 and femoral neck Z-score + 2.22 .

**Clinical Management:** The infant required colostomy and multiple surgeries for his anomalies. was supplemented with cholecalciferol 1000 units daily and ferrous sulfate. Anemia and cytopenia improved and transplant was not pursued. Radiographs at 3 months old showed improvement of sclerosis. Subsequent radiographs at 12 months showed normalization of skeletal appearance at the spine and femur, which was maintained at 24 months.

**Discussion:** This infant demonstrated radiographic findings concerning for infantile osteopetrosis which spontaneously improved without intervention, though the mother has asymptomatic elevations of BMD as an adult. The duplication on chromosome 15q14 is likely the cause of their mild osteopetrosis, though the specific gene involved in this area is uncertain.

### Disclosure

Michael Econs -Research funding and consultations for SiSaf

### P202

### Tibial tubercle fractures in pediatric patients with osteogenesis imperfecta

Anthony Dure[1], Emily Niu[2], Evan Sheppard[2], Benjamin Martin[2], Laura Tosi[2], Syed Ahmed[2]

[1]George Washington University School of Medicine and Health Sciences, Washington, D.C., USA
[2]Children's National Hospital, Washington, D.C., USA

### Abstract

### Objectives:

Osteogenesis imperfecta (OI) is a rare disorder involving abnormal production of type I collagen. Although fragility fractures are common, tibial tubercle apophyseal fracture reporting is limited in the literature. This study evaluates our series of tibial tubercle operative fractures in OI patients.

### Methods:

Retrospective review of all operative tibial tubercle fractures from 2010-2022 at a level I academic pediatric trauma center identified four patients with concomitant OI. Demographic information, OI type, antecedent and subsequent fracture history, surgical treatment, and OI treatment method was collected. Comparison was made with non-OI operative tibial tubercle patients (n=183).

### Results:

The mean OI tibial tubercle surgical patient was 11 years (range 9-14). The mean non-OI tibial tubercle surgical patient was 14.5 years (range 8.5-17.0). Two out of four OI patients were female (50%) while 94.5% were male in the non-OI group. Within the OI group one patient was diagnosed with OI after their tibial tubercle fracture (15 months later). OI types included: type I (n=3) and type IV (n=1). Prior to tibial tubercle fracture, no patients were on bisphosphonate therapy and three were on vitamin D supplementation. All patients underwent open reduction with cannulated screw fixation. Post-operative casting was used in one OI (25%) compared to 27.3% of non-OI patients. Immediate weight bearing was allowed in three OI (75%) compared with 52.5% of non-OI patients. Within the OI group, there was one fracture displacement four days after index surgery with revision surgery fourteen days after index surgery. A postoperative superficial wound infection and a non-displaced inferior patella pole fracture were non-operatively managed in two patients. All OI patients returned to full baseline activities at 5.3 months compared with 4.4 months in the non-OI group. All OI patients went on to suffer subsequent fractures in other anatomical locations.

### Conclusions:

OI tibial tubercle surgical fractures demographics favored younger female patients compared to the non-OI cohort of older male patients. The OI group made up 2.1% of all tibial tubercle surgical fractures. Surgical revision rate was 25% for the OI group vs 1.1% in the non-OI group. All OI patients returned to their baseline function comparably to the non-OI group.

### P203

### Unusual presentation of isolated parathyroid failure in an adolescent boy

Pon Ramya Gokul[1], M Zulf Mughal[2], Charlotte Jarvis[1], Suzanne Armitage[1], Renuka Ramakrishnan[1]

[1]Alder Hey Children's Hospital, Liverpool, UK [2]Al Jalila Children's Hospital, Dubai, UAE

### Abstract

**Background:**

Hypoparathyroidism is an uncommon condition in children characterized by hypocalcaemia and hyperphosphataemia due to defective synthesis/secretion of parathyroid hormone (PTH), end-organ resistance (pseudohypoparathyroidism) or an inappropriately activated calcium-sensing receptor (CaSR). Clinical symptoms include muscle spasms, stridor, seizures, and syncope. Here we present an unusual case of isolated hypoparathyroidism presenting with cardiac failure in an adolescent boy.

**Presenting Problem:**

A 13-year-old boy, previously fit and well presented with only a week's history of hand twitching, spasms, and breathlessness on exertion. He was admitted to intensive care with respiratory distress and poor cardiac function. There was no significant medical, surgical, or family history and examination did not reveal any signs of ectodermal dysplasia or fungal infection. He required ventilation and inotropic support. Initial echocardiogram showed systolic impairment with poor ejection fraction and a rim of pericardial effusion in a structurally normal heart, with significantly elevated cardiac markers. Adjusted calcium level at presentation was significantly low at 1.69 (2.15-2.74 mmol/L) with an inappropriately normal Parathyroid hormone level of 2.1(1.1-6.9 pmol/L), a low urine Calcium/creatinine ratio and a normal vitamin D level. His nasopharyngeal aspirate PCR was positive for adenovirus.

**Clinical management:**

Treatment with intravenous calcium and active Vitamin D was administered, resulting in the gradual improvement of calcium levels. Additional tests looking at an underlying cause were all reported to be normal (highlighted in the table). Currently the patient is well with calcium levels maintained in the normal range on treatment. His cardiac enzymes are showing a steadily improving trend and he has resumed his usual activities.

**Conclusion:**

To our knowledge, there are no reports of isolated hypoparathyroidism presenting in this severe manner at diagnosis in older children. Prompt recognition and treatment of hypocalcaemia is crucial for full recovery of cardiac function. As no underlying cause could be found after extensive workup, it raises the possibility of a pathogenic change in a novel gene, adenovirus associated parathyroid injury or isolated CaSR antibody development in such patients.

| **Blood investigations** | **At presentation** | **Recent** |
|---|---|---|
| **Bone profile:**<br>Corrected calcium | 1.69 | 2.27 |

| | | |
|---|---|---|
| (2.15-2.74) mmol/L | | |
| Parathyroid hormone<br>(1.1-6.9) pmol/L | 2.1 | 0.6 |
| Phosphate<br>(0.81-1.61) mmol/L | 2.35 | 1.63 |
| Magnesium<br>(0.7-1.00) mmol/L | 0.80 | 0.70 |
| Vitamin D<br>(>50) nmol/L | 36 | 92 |
| Creatine kinase<br>(24- 195) iu/L | 4436 | 339 |
| Troponin I<br>(0-14) ng/L | 6203 | 40 |
| NT-pro BNP (<200ng/L) | 11346 | 500 |
| **Microbiology:**<br>Nasopharyngeal aspirate PCR<br>Coronavirus COVID-19 PCR<br>Blood and urine culture | Adenovirus positive<br>Negative<br>No growth | |
| **Imaging:**<br>ECHO | Ejection fraction 35%<br>septal dyskinesia with impaired function | |
| Chest X-ray | Extensive bilateral pulmonary infiltrates lower lobe. Left retrocardiac density. Heart size is at the upper limits of normal | |
| Renal ultrasound | Normal, no nephrocalcinosis | |
| CT head | Normal | |
| **Genetics:**<br>Microarray | Microarray normal (no change in 22q11.21 syndrome region) | Hypoparathyroidism gene panel – negative<br><br>Trio whole genome sequencing to look for TBX1 and other novel gene mutation- results awaited (proband and parents) |

| | | |
|---|---|---|
| **Autoimmune disease and other workup:** | | Normal Ig and complement profile,<br>Negative for TPO Ab, Anti GAD Ab,<br>Islet cell Ab, Zn transporter Ab<br><br>CaSR Ab – test unavailable in the UK |
| Wilson's disease | | Normal Ceruloplasmin and serum copper and urine copper levels |
| Hemochromatosis | | Normal ferritin |
| Mitochondrial disease | | Normal lactate, pyruvate, acylcarnitine, plasma and urine amino acids |

### P204

### Empowering diagnosis and access: The Tin Soldiers Clinician Champions Alliance for Fibrodysplasia Ossificans Progressiva (FOP) and paediatric musculoskeletal conditions

Christiaan Scott[1,2], Odette Schwegler[3], Clive Friedman[4], Patricia Delai[5], Vrisha Madhuri[6], Amanda Cali[7], Vanda Harries[3]

[1]Professor of Paediatric Rheumatology, University of Cape Town, Cape Town, South Africa. [2]Medical Director, Tin Soldiers, Cape Town, South Africa. [3]Tin Soldiers Global, Johannesburg, South Africa. [4]Schulich School of Medicine and Dentistry, London, Canada. [5]Hospital Israelita Albert Einstein research Institute, Sao Paulo, Brazil. [6]Senior consultant Orthopaedics Amara hospital Tirupati, Vellore, India. [7]Tin Soldiers Global, New Jersey, USA

### Abstract

**Objectives:** Fibrodysplasia Ossificans Progressiva (FOP) is a rare inherited condition characterized by progressive heterotopic ossification in ligaments and muscles, serving as a focal point for the Tin Soldiers Clinician Champions Alliance. With an incidence rate of one in a million births, FOP inflicts severe disability, compounded by limited awareness, delayed diagnosis, and challenges in accessing care pathways.

Other rare Musculoskeletal (MSK) conditions contribute significantly to the global non-communicable disease (NCD) burden, resulting in pain, disability, mental health challenges, and heightened risks of other chronic health conditions and all-cause mortality.

Here we describe the role of the Tin Soldiers Clinician Champions Alliance (CCA) in driving the diagnosis and enhancing access to care pathways for Fibrodysplasia Ossificans Progressiva (FOP) and other paediatric musculoskeletal conditions.

**Methods:** The Tin Soldiers CCA adopts a comprehensive strategy, encompassing targeted education campaigns, capacity building for healthcare professionals, and the identification of care and referral centres. Through these initiatives, the alliance strives to create a supportive environment for clinicians, fostering the exchange of insights and the development of effective care pathways. To ensure sustained efforts and address the broader spectrum, the Tin Soldiers Clinician Champions Alliance has been established.

**Results:** Over the past four years, approximately 3800 professionals have been educated through initiatives in urban and rural South Africa, Kenya, India, Brazil, and an online CME series. The effort has led to 52 FOP diagnoses and the referral of scores of children with other rare musculoskeletal conditions from South Africa to Kenya, Ghana, Chad, Ethiopia, Nigeria, India, Bangladesh, and Australia.

**Conclusion:** The Tin Soldiers Clinician Champions Alliance emerges as a dynamic force in the global effort to empower diagnosis and access to care for FOP and paediatric musculoskeletal conditions. By uniting clinicians, raising awareness, and fostering collaboration, the alliance strives to make a profound impact on the lives of individuals affected by these conditions, promoting early diagnosis and improving access to appropriate care pathways.

### P205

### Allogeneic hematopoietic stem cell transplantation in Morquio syndrome type A: A case report

Ruggero Lanzafame, Maria Ester Bernardo, Giulia Consiglieri, Francesca Tucci, Matilde Cossutta, Alessandro Aiuti

IRCCS San Raffaele Hospital, Milan, Italy

### Abstract

**BACKGROUND**: Morquio syndrome type A is an autosomal recessive lysosomal storage disorder caused by the deficiency of the enzyme N-acetylglucosamine-6-sulfate sulfatase (GALNS). This deficiency causes the accumulation of glycosaminoglycans (GAG) primarily in bone, cartilage and its extracellular matrix, leading to a skeletal spondyloepiphyseal dysplasia.

**PRESENTING PROBLEM**: A 14-month-old boy, born to non-consanguineous parents, presented with short stature (-2.07 SDS), kyphosis, protrusion of the chest, genu valgum and craniocervical instability. After initial assessments, urinary GAG levels were found to be above reference values, with undetectable GALNS enzyme activity in leukocytes. GALNS gene analysis was performed, confirming the diagnosis of Morquio syndrome type A.

**CLINICAL MANAGEMENT**: Enzyme replacement therapy (ERT) was initiated at the age of 16 months, despite its inability to adequately penetrate non-vascularized cartilage tissues is known. Allogeneic hematopoietic stem cell transplantation (HSCT), performed in a small number of patients, has been shown to effectively restore a sustained production of GALNS enzyme activity to normal levels in leukocytes, and in some patients this has been associated with improvements in joint mobility, height gain and reductions in thoracic deformities. Therefore, HSCT from a matched unrelated donor was performed at the age of 4 years and 2 months in our patient, following a myeloablative conditioning.

One year after treatment, enzyme activity levels normalized and urinary GAG levels showed a slight reduction, despite the patient developed a mixed chimerism (12% host, >85% donor). In terms of skeletal manifestations, craniocervical instability and kyphosis remained stable, joint mobility slightly worsened and the patient is currently a candidate for orthopedic surgery to correct genu valgum. The stature is now -3.48 SDS with normal GH secretion.

**DISCUSSION**: While ERT and HSCT are available treatment options for patients with Morquio syndrome, their effectiveness in addressing bone and cartilage lesions remains limited due to the difficulty in delivering the deficient enzyme to avascular tissues, particularly in patients with advanced stages of the disease when skeletal damage is already established. Autologous hematopoietic stem cell-gene therapy, currently under development in preclinical studies, could provide supraphysiological persistent enzyme levels, potentially delivering more enzyme to bone and cartilage lesions.

### P206

### Bisphosphonate treatment in a case of generalized arterial calcification of infancy with hyperinsulinism and an uncertain ENPP1 mutation

Sasigarn Bowden, Kandamurugu Manickam, Brent Adler, Rajesh Krishnamurthy, May Ling Mah

Nationwide Children's Hospital, Columbus, USA

### Abstract

**Background:**

Generalized arterial calcification of infancy (GACI) is a rare disorder characterized by infantile-onset diffuse calcification and/or stenosis of large and medium-sized arteries. Overall mortality is high (55%) within the first 6 months of life, with 24.4% dying in utero or being stillborn. We present a case of GACI co-existing with hyperinsulinism and the clinical outcome following bisphosphonate therapy.

**Presenting Problem:**

A male infant, born large for gestational age at 30 weeks, developed persistent hypoglycemia with elevated insulin (100 uIU/mL), consistent with hyperinsulinism. Echocardiogram obtained on day 12 prior to diazoxide treatment demonstrated pulmonary hypertension, mild biventricular hypertrophy, and increased echogenicity of the main and branch pulmonary arteries with mild stenosis, raising concerns for GACI.

**Clinical Management:**

Computed tomography (CT) confirmed extensive arterial calcification in the pulmonary arteries. Weekly intravenous pamidronate was initiated on day 17 at 0.1 mg/kg/dose. A CT performed 1 month later showed disease progression with new calcifications in the ascending aorta and right atrium. Pamidronate dose was increased to 0.2-0.3 mg/kg/dose for total 7 doses and transitioned to oral etidronate. Hypoglycemia was managed by intravenous dextrose, later transitioning to enteral feeding. Oral etidronate was maintained (15-18 mg/kg/day) until age 1.5 years. CT at 3.6 years revealed decreased calcifications. At age 5, he demonstrated normal growth and normal pulmonary artery dimensions by echocardiogram. Genetic testing identified a heterozygous *ENPP1* gene variant of uncertain significance (c.484A>G p.Ser162Gly), previously unreported. Despite extensive testing including genome sequencing, a second variant was not identified. His younger sister, carrying the same variant, had hypoglycemia and abdominal aortic wall calcifications noted at one week of life. Both siblings were initial twin gestations with in-utero demise.

**Discussion:**

We report a case of GACI and hyperinsulinism linked to an *ENPP1* mutation of uncertain significance. Approximately 30% of GACI cases lack identification of a second variant. The proband was diagnosed early as incidental finding on echocardiogram and pulmonary artery involvement. The impact of bisphosphonate treatment on disease progression improvement remains uncertain. Previous retrospective analysis suggested benefits in reducing mortality, but recent findings challenge this, emphasizing the need for further investigation into the efficacy of bisphosphonate therapy for GACI.

### P207

### Kenny Caffey syndrome 2; a rare cause of hypoparathyroidism, and short stature

Kyriaki Hatziagapiou[1], Amalia Sertedaki[1], Dermentzoglou Vasiliki[2], Christina Kanaka-Gantenbein[1], Sophia D. Sakka[1]

[1]Division of Endocrinology, Diabetes and Metabolism, ENDO-ERN Center for Rare Pediatric Endocrine Disorders, First Department of Pediatrics, Medical School, National and Kapodistrian University of Athens, Aghia Sophia Children's Hospital, Athens, Greece. [2]Department of Radiology, Aghia Sofia Children's Hospital, Athens, Greece

### Abstract

**Background:** Kenny–Caffey syndrome 2 (KCS2) is a rare cause of hypoparathyroidism, inherited in an autosomal dominant manner, resulting from pathogenic variants in the FAM111A (Family With Sequence Similarity 111 Member A). FAM111A has a crucial role in intracellular pathways regulating skeletal development, and height gain, parathyroid gland development, and parathormone synthesis, thus calcium, and phosphorus homeostasis.

**Presenting Problem:** We report the case of a 4-year-old boy, who presented with the characteristic, and newly identified clinical, biochemical, radiological and genetic abnormalities of the syndrome. The toddler presented for short stature investigation. He is the second child of the family, born after an otherwise full-term uneventful pregnancy. On revisiting his personal history several episodes of hypocalcemia were reported, requiring repeated hospital admissions. There is no family history of short stature or hypoparathyroidism. On examination his height and weight were -4.11SD, and -3SD, respectively. He had noticeable dysmorphic features, including prominent forehead, small palpebral fissures, low set ears, long philtrum, thin upper lip, pectus excavatum, and genu valgus. The closure of anterior fontanel was delayed until the age of 2-years. Skeletal survey revealed cortical thickening, and medullary stenosis of the long bones.

The biochemical evaluation demonstrated hypocalcemia, high normal phosphorous and inappropriately low normal parathyroid hormone (PTH). To rule out other causes of short stature, routine investigations were performed, revealing on CNS MRI small pituitary gland and empty sella, and low IGF-1. Nocturnal levels of growth hormone were normal. Comprehensive cardiovascular, and ophthalmological evaluation was negative for any pathology. Based on the aforementioned clinical, biochemical and radiological features KCS2 was suspected. Genetic testing revealed heterozygosity for the FAM111A gene with de novo pathogenic variant c.1706G>A, p.Arg569His.

**Clinical management:** Normocalcemia was achieved with oral elemental calcium and cholecalciferol, or alfacalcidol, and occasionally with parenteral calcium gluconate.

**Conclusion:** To our knowledge this is the first time that KCS2 is associated with low levels of IGF-1, and pituitary abnormalities on the MRI of the hypothalamic-pituitary region. The underlying pathogenic variant results in hyperactive protease activity of FAM111A causing abnormal degradation of DNA-binding proteins and aberrant nuclear morphology leading to disease-associated comorbidities.

### P208

### A rare cause of hypoparathyroidism: Kenny Caffey type 2 - Case report

Kelly Maury[1], Oscar Brunetto[2], Fabiana Vacile[3], Romina Flores[4], Gabriela Bidal[5], Gabriel Faraj[5]

[1]Hospital Pedro Elizalde, Buenos Aires, Argentina. [2]Hospital Pedro Elizalde, Buenos aires, Argentina. [3]Hospital Médico Policial Churruca Visca, Buenos Aires, Argentina. [4]Hospital Medico Policial Churruca, Buenos Aires, Argentina. [5]Hospital Medico Policial Churruca, Buenos Aires, Argentina

### Abstract

### Background:

Hypoparathyroidism is a disorder in calcium homeostasis that is caused by inadequate secretion of parathyroid hormone, either due to an alteration in its synthesis, secretion or action. In children there may be different genetic etiologies. It can be classified as isolated or syndromatic hypoparathyroidism, within which we find Kenny Caffey syndrome.

We describe a patient with a diagnosis of Kenny caffey type 2..

### Case report:

A two-month-old child was referred to the Pedro Elizalde hospital with a diagnosis of hypocalcemia secondary to hypoparathyroidism and suspicion of Degeorge syndrome. On physical examination, her phenotype was peculiar, finding elphoid fascies and hypoplasia of the facial mass, with micrognathia and microphthalmia,relative macrocephaly, large anterior fontanelle, severe short stature and a micropenis.

Laboratory tests showed normocytic normochromic anemia, elevated transaminases, inhibited pH with hypocalcemia, hyperphosphatemia and increased vitamin D for the age range. His kidney function and other hormonal axes were normal. The echocardiogram showed an atrial septal defect and mild pulmonary stenosis.

Whole body x-rays revealed tubular long bones and medullary space with stenosis.

Fish was performed for 22q11.2 deletion and was normal. A clinical exome was performed without finding positive results, and ruling out Kenny Coffey type 1 and alterations in the CASR, PTH and GCMB.

Kenny coffey type 2 is suspected, and exome expansion is requested and a variant of uncertain significance in heterozygosity was identified in the .FAM111A, c.913A>G p.(Arg305Gly)

At this time, the genetic results of the parents are awaited.

### Conclusion:

Kenny Caffey syndrome type 2 is a heterogeneous disorder with multiple manifestations

Early diagnosis helps prevent comorbidities and provide better interdisciplinary follow-up.

### P209

### 11-year old boy with autosomal dominant osteopetrosis type II.  A case report

Monika Morawska, Bogumiła Górczewska, Elżbieta Jakubowska-Pietkiewicz

Department of Pediatrics, Newborn Pathology and Bone Metabolic Diseases Medical University of Lodz, Łódź, Poland

### Abstract

BACKGROUND:

Osteopetrosis is a group of severe inherited bone diseases characterized by increased bone mineral density and fragility due to defective function or development of osteoclasts. Patients suffer from frequent fractures, and in some cases from reduction of marrow space or cranial nerve entrapment which may result in visual and/or hearing impairment. Among osteopetrotic conditions autosomal dominant osteopetrosis type II (ADO II), caused by mutation in the chloride channel 7 (*CLCN7*) gene, is the most frequent form. In this case we report an ADO II case, firstly mistakenly treated as myopathy, in a patient without the family history of ADO.

PRESENTING PROBLEM:

11-year old boy was admitted with a history of frequent bone fractures, one sided vision loss due to optic nerve atrophy and fatigue. On examination the patient was found with low body weight – centile BMI <3, poorly developed muscle tissue, bone exostosis in both feet and severe caries. High AST and CK values were observed from 6th year of live, which in combination with clinical presentation caused the diagnosis of unspecified miopathy.

CLINICAL MANAGEMENT:

We extended radiological diagnostic and found greatly increased bone density in densitometry: Z-score total body 7,1, Z-score spine 10,0. Characteristic ,,sandwich vertebrae" were found in spine X-ray.  Genetic tests has shown the CLCN7 gene mutation.

DISCUSSION:

Osteopetrosis is a rare disease, although one should consider in case of frequent fractures, characteristic radiological findings and otherwise unexplained elevation of CK, LDH and AST enzymes. Early diagnosis and implementation of multidisciplinary care can improve quality of life in this group of patients.

### P210

### Radiological features of cleidocranial dysplasia

Ruggero Lanzafame, Elisa Sala, Ludovica Crocè, Giorgia Piselli, Gabriele Drago, Stefano Mora, Marco Pitea

IRCCS Ospedale San Raffaele, Milano, Italy

### Abstract

BACKGROUND: Cleidocranial dysplasia is a rare developmental disease affecting the skeleton and the teeth. The radiographic evaluation of patients is important for the diagnosis, as the radiological findings are pathognomonic.

PRESENTING PROBLEM: A 13-year-old boy was referred to us for a second opinion due to his short stature compared to the midparental target height (height -2.25 SDS, target height +0.57 SDS). He was born at term, AGA, to non-consanguineous parents with no family history of bone diseases.

CLINICAL MANAGEMENT: During our initial evaluation, we noted some peculiar phenotypic characteristics: relative macrocrania, broad forehead with frontal bossing, hypertelorism, saddle nose, prominent philtrum and wide mouth. He still had all his deciduous teeth and exhibited mild hypermobility of the shoulder girdles, a broad thorax, and a squared appearance of thumbs and toes.

He had previously undergone a hand-wrist X-ray, which showed a bone age delay of 2 years compared to his chronological age, with a squared appearance of the metacarpals and phalanges. He had also undergone a genetic analysis of the *ROR2* and *WNT5* genes, ruling out Robinow syndrome.

We requested the following exams:

- Chest X-ray, which revealed clavicle hypoplasia and a bell-shaped ribcage;
- Lateral teleradiography of the skull (*image attached*), which showed open coronal and metopic sutures, multiple wormian bones in the occipital area and some unerupted supernumerary teeth;
- Genetic analysis of the *RUNX* and *CBFA1* genes, which confirmed the suspicion of cleidocranial dysplasia by revealing a de novo pathogenic mutation of the *RUNX* gene.

DISCUSSION: One of the most indicative clinical findings of cleidocranial dysplasia is shoulder hypermobility due to partial or complete absence of the clavicles. Dental findings are characterized by decreased eruptive force of both primary and permanent dentitions, prolonged retention of primary teeth and an increase in odontogenesis leading to an excessive number of supernumerary teeth. Radiographic evaluation of the chest and skull can reveal hypoplasia or complete absence of the clavicles, late closure of cranial fontanels, open skull sutures, wormian bones and multiple unerupted supernumerary teeth.

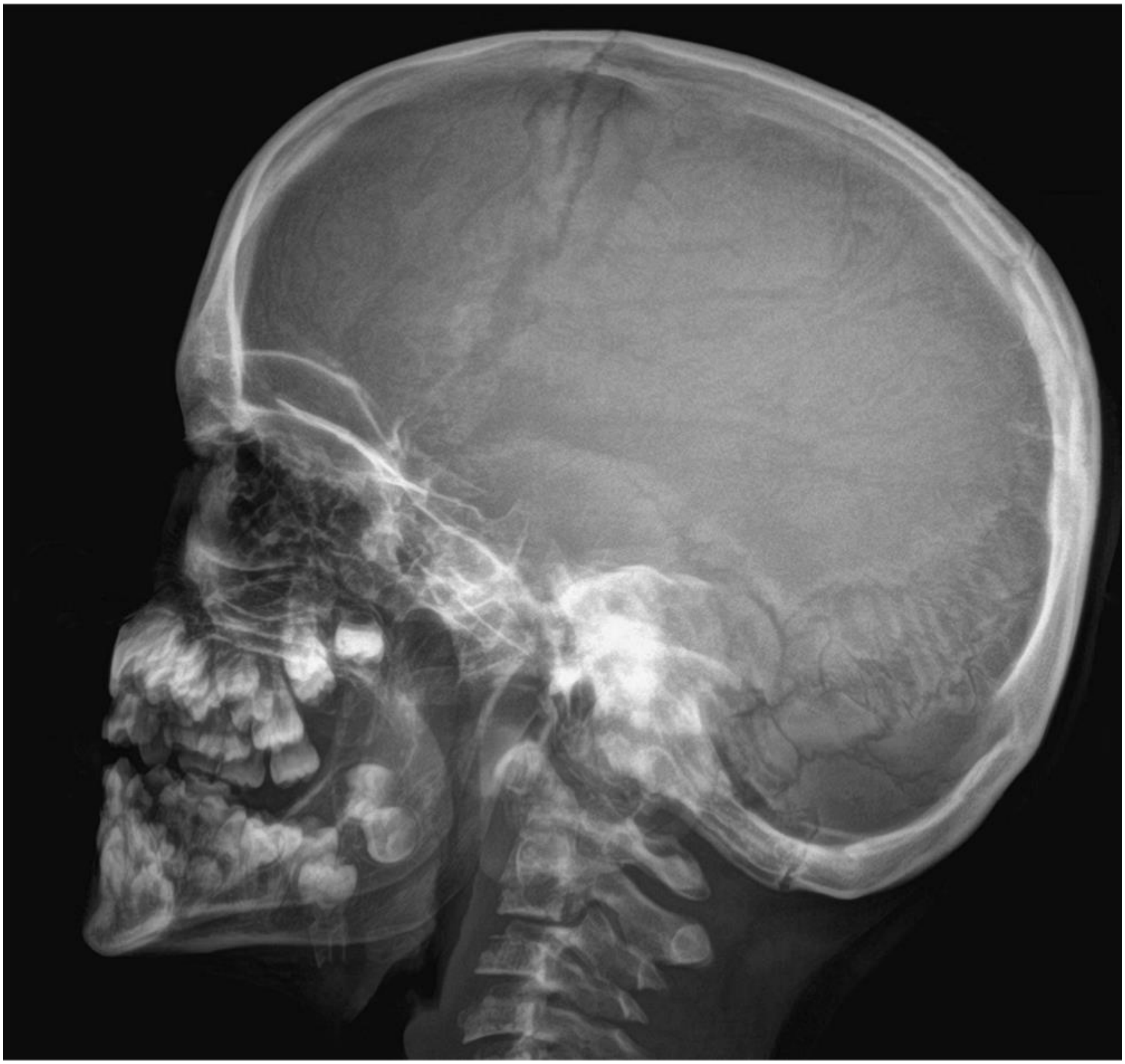

### P211

### Creation and assessment of the reliability of a standardised score for the evaluation of a spinal gibbus in children with achondroplasia

Naomi Pibworth[1], Kathryn Phillips[1], Paul Watson[1], Moira Cheung[2], Melita Irving[1], Rhoda Akilapa[1], Phillipa Prentice[1], Alessandra Cocca[1]

[1]Evelina Children's Hospital, London, UK [2]Great Ormond Street Hospital for Children, London, UK

### Abstract

### Background

75% of infants with Achondroplasia have a Thoracolumbar kyphosis/ spinal gibbus of 20 degrees or more between T10-L2 vertebra1,2. The presence of this reduces with age as truncal tone increases and children progress to independent sitting, standing and walking3,4,6.

Progression to a fixed thoracolumbar gibbus can potentially be linked to future back pain and spinal red flags in adulthood5,6. At present there are no local or national standardised clinical measures to assess a spinal gibbus, other than completing spinal radiographs.

### Objectives

- Reach a consensus within the skeletal dysplasia team as to what would qualify as a mild, moderate or severe gibbus in children with Achondroplasia.
- Create a Spinal Gibbus Score assessment tool that can be used by any clinician in clinic to identify severity of the gibbus.
- Evaluate the reliability of the Spinal Gibbus Score assessment tool by piloting in our MDT Achondroplasia clinic.

### Methods

- Single institution prospective quantitative double blinded cohort study
- Spinal Gibbus Score assessment tool piloted in MDT clinic with children diagnosed with Achondroplasia aged between 0-5 years of age, with the patient assessed in prone to eliminate postural influences.
- Spinal Gibbus Score to be completed and recorded separately by the Skeletal Dysplasia Physiotherapist and the Paediatric Bone consultant
- Results to be statistically analysed comparing inter-tester reliability

Fig.1: Example of gibbus score

| Palpation of Spine T10-L2 (in prone) | score | Observation of spine (in prone) | score | Total score | |
|---|---|---|---|---|---|
| No palpable gibbus | 0 | No vertebral prominence visible  | 0 | **No Gibbus:** | 0 |
| Palpable prominence of 1-2 vertebra | 1 | Visible prominence of 0-1 vertebrae   | 1 | **Mild Gibbus** | 1 -2 |
| Palpable prominence of 3-4 vertebrae | 2 | Visible prominence of 2-3 vertebrae   | 2 | **Moderate Gibbus** | 3-4 |
| Palpable prominence of 5+ vertebrae | 3 | Visible prominence of 4+ vertebrae  | 3 | **Severe Gibbus** | 5-6 |
| | | | | **Pressure marking on skin +** | |

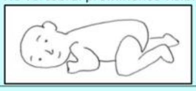

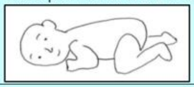

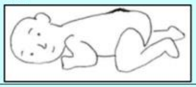

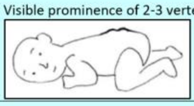

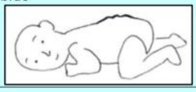

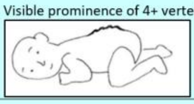

### Results

In order to measure inter-rater reliability, the Cohen's Weighted Kappa method was used for:

- The total gibbus score (k 0.792)
- The observation score (k 0.821)
- The palpation score (k 0.756)

All p.values were < 0.05.

The level of agreement is “almost perfect” for the observation score and substantial; for the palpation score and the total score.

| | Weighted Kappa | Std. Error | p.value | Asymptotic 95% Confidence Interval Lower Bound | Asymptotic 95% Confidence Interval Upper Bound |
|---|---|---|---|---|---|
| **Total score R1 vs R2** | 0.792 | 0.122 | <0.001 | 0.552 | 1.032 |
| **Observation score R1 vs R2** | 0.821 | 0.122 | <0.001 | 0.583 | 1.060 |
| **Palpation score R1 vs R2** | 0.756 | 0.166 | 0.003 | 0.430 | 1.082 |

**Conclusion**

The Spinal Gibbus Score assessment tool proved easy to use in clinic and our data shows it has a very high inter-rater reliability score, which is statistically significant. We are aware that this is a preliminary study using a very small sample group (10 patients) and so future studies would need to test inter-rater reliability on a bigger patient cohort. Future studies could also look at intra-rater reliability and could also include other staff such as our CNS or OT, or compare the Spinal Gibbus Score to spinal X-ray imaging.

The Spinal Gibbus Score could be a potentially very useful assessment tool in our MDT clinics and may allow us to see a correlation between a Spinal Gibbus Score and a child's age, mobility development, clinical symptoms, or their Foramen Magnum Score7- which could help inform their ongoing management.

References
1. Khan, B.I. et al. (2016) 'Prevalence of scoliosis and thoracolumbar kyphosis in patients with achondroplasia', Spine Defor mity, 4(2), pp. 145–148. doi:10.1016/j.jspd.2015.08.003. 2. Ando, K. et al. (2021) 'Sagittal alignment at 3 years old determines future Thoracolumbar kyphosis in achondroplasia: A prospective study with minimum 5-year follow-up from infancy', North American Spine Society Journal (NASSJ), 6, p. 100070. doi:10.1016/j.xnsj.2021.100070. 3. Mok, S. et al. (2022) 'Radiographic factors for progression of thoracolumbar kyphosis in achondroplasia patients after walking age: A generalized estimating equation analysis', Clinics in Orthopedic Surgery, 14(3), p. 410. doi:10.4055/cios22046. 4. Khan, B.I. et al. (2016) 'Prevalence of scoliosis and thoracolumbar kyphosis in patients with achondroplasia', Spine Deformity, 4(2), pp. 145–148. doi:10.1016/j.jspd.2015.08.003. 5. Borkhuu, B. et al. (2009) 'Factors related to progression of thoracolumbar kyphosis in children with achondroplasia', Spine, 34(16), pp. 1699–1705. doi:10.1097/brs.0b013e3181ac8f9d. 6. Liao, J.-C. et al. (2006) 'Surgical treatment of achondroplasia with thoracolumbar kyphosis and spinal stenosis—a case report', Acta Orthopaedica, 77(3), pp. 541–544. doi:10.1080/17453670610012566. 7. Cheung, M.S. et al. (2020) 'Achondroplasia foramen magnum score: Screening infants for stenosis', Archives of Disease in Childhood, 106(2), pp. 180–184. doi:10.1136/archdischild-2020-319625.

### P212

### The many faces of hypophosphatasia

Stepan Kutilek[1], Petr Munzar[2], Tomas Honzik[3]

[1]Department of Paediatrics, Klatovy Hospital, Klatovy, Czech Republic. [2]Department of Paediatrics, Pardubice Hospital, Pardubice, Czech Republic. [3]Department of Paediatrics and Inherited Metabolic Disorders, First Faculty of Medicine, Charles University and General University Hospital in Prague, Prague, Czech Republic

### Abstract

### Background

Hypophosphatasia (HPP) is a rare, inherited disease characterized by disorders of calcium/phosphate metabolism, bone deformities, loss of teeth,recurrent fractures,growth retardation.HPP is caused by mutations of gene encoding tissue-non-specific alkaline phosphatase(TNSALP). The mutation results in TNSALP deficiency, resulting in defective skeletal mineralization. There are six subtypes of HPP: (1)Perinatal,(2)Infantile,both are lethal; (3)Benign prenatal HPP, (4)Childhood-onset HPP, (5)Adult HPP and (6)OdontoHPP with a better prognosis.Clinical manifestations of HPP are very diverse, ranging from severe skeletal deformities to asymptomatic course.

### Case Report & Management

A girl with uneventful family history. At the age of three months muscle hypotonia was apparent,followed by psychomotor retardation. At the age of 12 months she was able to sit and she started to walk at 24 months.The brain ultrasound was repeatedly normal. Joint hypermobility and muscle hypotonia were apparent, together with craniofacial dysmorfic features (incl.turicephaly, saddle nose)and pectus excavatum. She never suffered any fracture, never had any dental problems. At the age of 13 months, low S-ALP (1.5µkat/L; normal 2.36–7.68) was encountered with normal calcaemia, phosphataemia, PTH. Wrist X-ray was normal.Urinary phosphoethanolamine excretion was elevated. Screening for inborn errors of metabolism was negative. Her father has somewhat similar facial appearance with normal psychomotor development,never suffered fractures.His S-ALP was also low (0.79µkat/L;normal 0,83-2,27).The assessment of TNSALP gene revealed heterozygous mutation c.571G>A in both girl´s and father´s DNA.Concerning girl´s mother, the screening for TNSALP mutations was negative.Girl was further followed-up, her S-ALP was within normal age-dependent range (2.3 and 2.66 µkat/L;normal 2.13 – 5.89 µkat/L) at the age of three and four years, respectively. Currently, the girl is 12 years old, mildly retarded, completely free of fractures or dental issues. Her growth curve is normal.

### Discussion & Conclusions

The c.571G>A mutation originates from Scandinavia and is responsible for recessive mild-to-moderate forms of HPP, but not for lethal HPP. Both girl and father are asymptomatic HPP heterozygots.There is no correlation between genotype and phenotype in HPP. In conclusion, HPP should be taken into consideration when low S-ALP is encountered. However, even individuals with proven TNSALP mutation can be completely asymptomatic.

### P213

### Bisphosphonate role in children with osteogenesis imperfecta

Siska Lubis, Melda Deliana

Universitas Sumatera Utara, Medan, Indonesia

### Abstract

**Background**:

Osteogenesis imperfecta (OI) is a genetic disease wherein the body fails to produce collagen, leading to increased susceptibility to bone fractures even with minimal trauma or without any trauma. Bisphosphonates are a primary therapy for OI in children. Currently, bisphosphonate therapy is covered by government health insurance, thereby expecting to improve the quality of life for children with osteogenesis imperfecta in Indonesia.

**Presenting Problem**:

A 3-year-10-month-old male with a history of repeated fractures since 3 months of age. The patient has broken his right leg five times, with the recent fracture occurred three months ago before admission on his left leg. The patient also experienced pain in his bones. Physical examination showed blue sclera. The bone mineral density (BMD) examination revealed severe osteoporosis with a Z-score of -7.3 in the pediatric spine L1-L4 and a Z-score of -4.0 in the pediatric left femur/total hip. A bone survey of this patient depicted features consistent with osteogenesis imperfecta, including pathological fractures and osteoporosis. The patient was diagnosed with OI.

**Clinical Management**:

The patient was treated with intravenous bisphosphonate in zoledronic acid form at a dose of 0.05 mg/kgBW. There were no side effects such as flu-like syndrome during and after administration. The patient was also treated with vitamin D 1000 IU and Calcium supplementation. Six months after the administration of zoledronic acid, the patient received a second dose. There were no new fractures or any bone pain reported after the initial administration.

**Discussion**:

Bisphosphonate therapy reduces bone resorption by osteoclasts, induces osteoclast apoptosis, and prevents osteoclast formation. Intravenous bisphosphonates should be considered for use in children with severe OI, particularly in those experiencing two or more long bone fractures per year. To minimize the risk of bisphosphonate-induced hypocalcemia, serum vitamin D levels should be >50 nmol/L before therapy, and adequate calcium intake should be maintained post-therapy. The patient in this study experienced more than two long bone fractures; therefore, intravenous bisphosphonates were chosen. After administration, the patient showed improvement in their condition.

### LB1

### Stable and functional osteosynthesis of femoral deformity with intramedullary growing rods: The results of surgical correction in three patients with systemic skeletal disease

Andrii Zyma[1], Oleksandr Skuratov[1], Yurii Guk[1], Yurii Demyan[2], Tamara Kinchaya-Polishchuk[1], Andrii Cheverda[1]

[1]SI "The Institute of Traumatology and Orthopedics" by NAMS of Ukraine, Kyiv, Ukraine. [2]Regional Children Hospital, Mukachevo, Ukraine

### Abstract

**Objectives:** The use of intramedullary telescopic constructs for osteosynthesis in surgical correction of bone deformities in children with systemic skeletal disease (osteogenesis imperfecta, fibrous dysplasia) can be complicated by delayed bony union, and the structural and functional pathology of bone in patients with these disorders do not always make it possible to avoid displacement of bone fragments and effectively correct the deformity. This study evaluated the use of a novel telescopic intramedullary growing rod for the treatment of femoral deformity.

**Methods:** Analysis of complex treatment of femoral deformity was performed in 3 patients (1 female and 2 males, including 2 patients with osteogenesis imperfecta type I and 1 patient with localized fibrous dysplasia, deformities 25-30 degrees). Average age was 9.8 years (range 8-11). Patients underwent anti-osteoporotic therapy with pamidronic acid drugs 3 months before surgery, in order to improve the structure of bone tissue. After that, this group of patients was performed underwent corrective osteotomy of the femur with osteosynthesis using an advanced intramedullary locking rod with a T-shaped telescopic part. The intramedullary construct consisted of a rod with proximal holes for locking screws, distal holes in for locking screws in two planes, and a T-shaped telescopic part with holes of the same diameter and distance between them as in the rod. In the first stage, a corrective osteotomy was performed and stabilized by the intramedullary construct with distal locking of the rod and its T-shaped telescopic component. In the second stage, distal locking screws were removed after consolidation of the osteotomy, dynamizing the construct to growth mode.

**Results:** Correction of the deformity and bony union were achieved in all cases with no recurrence of the deformity or implant failure over three year follow-up.

**Conclusion:** The efficacy of the application of the improved intramedullary telescopic construct for the surgical correction of bone deformities in children with systemic skeletal disease was demonstrated on the basis of principles of blocking intramedullary osteosynthesis, sustainable bony union, and growth over a telescoping intramedullary rod.

### LB2

### Multiple fractures as a presenting feature of osteopetrosis – a case report

Maria Sakellariou[1], Artemis Doulgeraki[2], Ioannis Tastsidis[1], Yvonne Papamerkouriou[1]

[1]Second Department of Orthopaedics, "P. & A. Kyriakou" Children's Hospital, Athens, Greece.
[2]Department of Bone and Mineral Metabolism, Institute of Child Health, Athens, Greece

### Abstract

BACKGROUND

Osteopetrosis refers to a group of rare genetic disorders of the bone characterized by increased bone mass, as a result of osteoclastic dysfuction. Depending on severity, age of onset and gene involved, it can be from asymptomatic to fatal and appears with a large range of heterogeneous symptoms, both skeletal and extraskeletal.

PRESENTING PROBLEM

This is a case report of late-diagnosed osteopetrosis, with is sole manifestation being multiple fractures. A 10 year-old female patient presented to the emergency department with a history of hitting her upper limb against the door of a car. A radiograph of the left hand demonstrated a fracture of the middle phalanx of the ring finger. An important incidental finding was the presence of markedly increased bone density.

CLINICAL MANAGEMENT

The fracture was treated conservatively. Diagnostic work up for increased bone density was undertaken. On further questioning, four more long bones fractures and hypothyroidism were reported. Radiographs of the whole spine showed vertebral body endplate sclerosis (“rugger jersey sign”). Vision and hearing were normal. Basic laboratory bone profile was unremarkable, except for high 5bTRAP (13,7 ng/ml) and LDH (320 U/l). Dual-energy X-ray absorptiometry revealed increased bone mineral density Z-scores of the lumbar spine and subcranial (Z-scores +9. 8 and +7.5, respectively). Genetic testing revealed a *de novo* heterozygous, pathogenic *CLCN7* mutation (exon 10, c856C>T).

DISCUSSION

Autosomal dominant osteopetrosis is the most common form of this disorder (1 in 20.000 births). It appears in late childhood or adolescence with mainly skeletal manifestations. Differential diagnosis includes other sclerosing bone dysplasias, such as pycnodysostosis, diaphyseal dysplasia, osteopoikilosis, melorrheostosis. Our patient presented with multiple fractures and no other symptoms. The incidental finding of increased bone density had gone unnoticed during previous fracture episodes, however the careful evaluation of the latest Xray enabled prompt diagnosis of this rare disease.

### LB3

### An exploratory molecular investigation of Cartilage-Hair Hypoplasia syndrome in Brazil

Maria Eduarda Gomes[1], Fernanda Khedy[2], Fernanda Manta[2], Bruna Luisa Fadel[3], Nicole Woldmar[3], Têmis Félix[4], Denise Cavalcanti[5], Dafne Horovitz[6], Juan Llerena Jr[6,7] , Sayonara Gonzalez[1]

[1]Laboratório de Medicina Genômica - IFF/Fiocruz, Rio de Janeiro, Brazil. [2]Laboratório de Hanseníase - IOC/Fiocruz, Rio de Janeiro, Brazil. [3]Laboratóriode Biologia Molecular e Proteômica do Sangue, LADETEC, IQ-UFRJ, Rio de Janeiro, Brazil. [4]Hospital de Clínicas de Porto Alegre - HCPA, Porto Alegre - RS, Brazil. [5]Departamento de Medicina Translacional - UNICAMP, Campinas - SP, Brazil. [6]Centro de Genética Médica - IFF/Fiocruz, Rio de Janeiro, Brazil. [7]Faculdade de Medicina de Petrópolis - UNIFASE, Petrópolis, RJ, Brazil

### Abstract

**Introduction:** Cartilage-Hair Hypoplasia (CHH) syndrome is an autosomal recessive disease described by McKusick and colleagues in 1964 in the Amish community. Clinically, patients present with metaphyseal dysplasia to varying degrees, along with hypotrichosis and immunodeficiency. At the molecular level, the condition is characterized by bi-allelic pathogenic variants in the *RMRP* gene. Despite being delineated almost 60 years ago it's still a not well-understood genotype-phenotype correlation. Previously, our group described a cohort of 23 Brazilian patients with CHH in which several pathogenic variants in the *RMRP* gene were reported. Among these, the variant g.196C>T stood out for its high frequency, unlike what was observed in patients in other nationalities, suggesting a possible founder effect (FE) in the Brazilian population.

**Objectives:** The aim objective of this work was to perform different molecular assays to expand de comprehension of CHH, as well as to investigate the hypothesis of a common ancestral origin of the g.196C>T variant.

**Methodology:** This work was structured in two exploratory axes concerning the mechanisms of the disease and one dedicated to the analysis of the FE. Molecular studies of the possible FE and genetic ancestry of families with CHH; and gene expression analysis comprehending qPCR and proteomics were performed.

**Results:** Nine new patients presented pathogenic variants compatible with CHH and were included in the cohort comprehending 31 patients with CHH. Using a panel of TAG SNPs markers, a common haplotype was observed on the chromosomes carrying the g.196C>T variant, suggesting a common origin for this variant. In addition, the cohort was characterized by autosomal Ancestry Informative Markers (AIMs), and European ancestry was the most prevalent (58%), followed by African American (24%) and Native American (18%) background. Additionally, proteomic analysis showed that the proteins expressed in leukocytes of patients translated distinct molecular pathways once compared to those expressed in control individuals and also show differences in the expression of proteins associated with key disease phenotypes, such as skeletal and immunological functions. RT-qPCR assays showed that both RMRP RNA levels and the two small RNAs derived from RMRP were significantly reduced in patients compared to the control group.

**Conclusion:** Our results contributed to the expansion of knowledge about various aspects of CHH syndrome, especially in Brazilian patients.

**LB4**

### The successful zoledronate treatment for patients with hematogenous osteomyelitis: The case series

Lubov Sorokina[1], Eugenia Isupova[1], Veronika Petukhova[2], Alexei Maletin[2], Alexander Mushkin[2,3], Mikhail Kostik[1]

[1]Saint-Petersburg State Pediatric Medical University, Saint-Petersburg, Russian Federation. [2]Saint-Petersburg Research Institute of Phthisiopulmonology, Saint-Petersburg, Russian Federation. [3]First Saint-Petersburg State Medical University n.a. academician Pavlov, Saint-Petersburg, Russian Federation

### Abstract

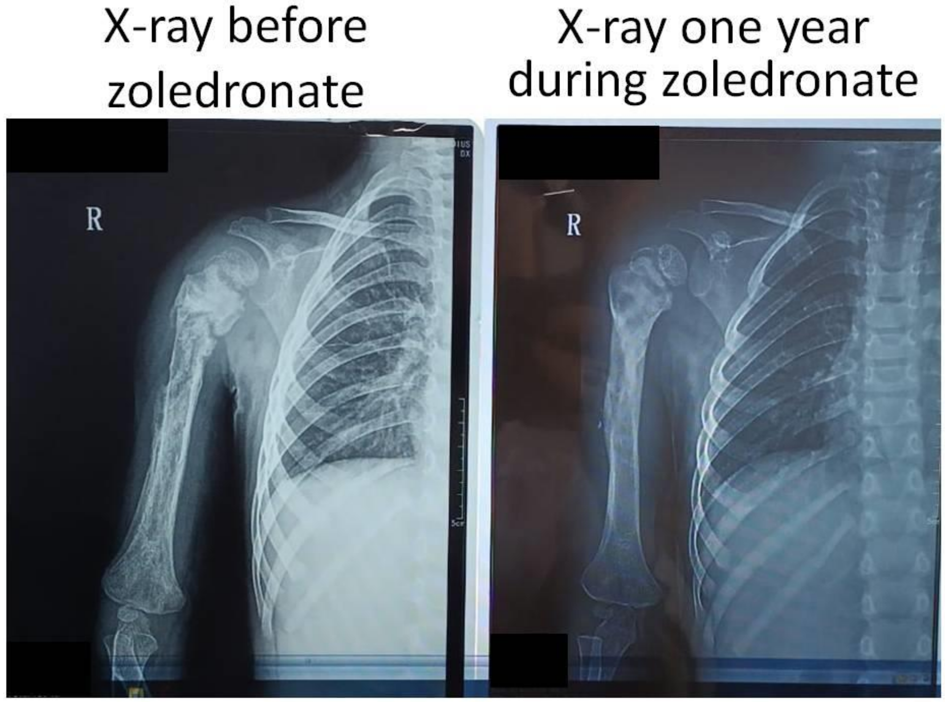

**Objectives**

Hematogenous osteomyelitis (HO) is usually acute disease, caused by different bacteria. The disease usually responds well to antibiotics, but in some cases it may have chronic course. In some localization (e.g. femoral head) of destructive foci the disease may have severe consequences required further orthopedic surgeries and arthroplasty.

Several previous studies showed the bone-conserving effects in bisphosphonates which may stop/decrease the following bone destruction in different bone diseases (chronic non-bacterial osteomyelitis, bone malignancy or bone metastatsis), has anti-iflammatory effect. In the experimental studies was shown the positive effect of bisphosphonates in rabbit experimental tuberculosis model. The role of bisphosphonates in HO has not yet studied before.

Our study aimed to evaluate the safety and efficacy of bisphosphonates in the HO

**Methods:** patients with chronic phase of HO were included. In all patients, routine clinical and radiological examination as well serial measurements of C-terminal telopeptide was done every six months.

**Results**

Five patients (3 boys and 2 girls) aged 3-13 years with chronic osteomyelitis as an outcome of acute HO became sadden acutely ill, in the summer and autumn period, against the background of complete well-being. All patients had a septic course with bilateral pneumonia with respiratory failure, and all were treated in the ICU, and had experience in massive broad-spectrum antibacterial therapy. Two patients developed femoral venous thrombosis. Four patients underwent surgical intervention for osteomyelitis. Three had proximal hip involvement with concomitant hip arthritis with flattering of the epiphysis and further femoral head subluxation development. One patient had the involvement of the distal femur metapiphysis and pelvic bones, and one had humerus osteomyeilits.

All patients received 2-4 zoledronic acid (0.05-1 mg/kg/infusion every 6 months) with significant pain, radiological (fig.) and functional improvement with discontinuation of the analgetics.  One patient underwent the hip arthroplasty and one is pending.

**Conclusion**

Bisphosphonates could be used in patients with HO. The studies about early bisphosphonates interventions in the acute stage are required.

### LB5

### Management of bisphosphonates in osteogenesis imperfecta at transition to adult care

Mark Heathfield[1], Martyn Dudley[2], Sophie Barlow[2], Judith Bubbear[2], Richard Keen[2], Belinda Crowe[1], Catherine DeVile[1]

[1]Osteogenesis Imperfecta Service, Great Ormond Street Hospital for Children NHS Foundation Trust, London, UK [2]Osteogenesis Imperfecta Transition Team, Royal National Orthopaedic Hospital, Stanmore, London, UK

### Abstract

**OBJECTIVES:** We aim to describe management of bisphosphonates at first transition appointment for young people aged 17 to 25 years who attended the Osteogenesis Imperfecta (OI) transition clinic, with mild, moderate, or severe OI within the last 5 years. This service is run collaboratively by the Royal National Orthopaedic Hospital (RNOH) Metabolic Bone service, and the OI service at Great Ormond Street Hospital for Children. The evaluation allows reflection on current practice for young people going through transition and decision-making around continuation or not of bisphosphonates as they enter adult services.

**METHODS:** Patients were identified who had a first appointment in the transition clinic. Data were collected on demographics, OI type and bisphosphonate regimen by two Clinical Nurse Specialists from paediatric and adult hospitals with administrative support.

**RESULTS:** Of 59 patients seen (M30, F29), 26 (44%) had type 1 OI, 8 (13.6%) type 3, 18 (30.5%) type 4, 4 (6.8%) type 5, 1 (1.7%) type 8, 1 (1.7%) type 13, and 1 (1.7%) was unclassified.

20 (34%) were not receiving bisphosphonate infusions at the time of transition although 10 had previously received pamidronate, of which 2 later switched to risedronate and 2 to zoledronate in childhood. 8 (14%) continued taking oral bisphosphonates, 20 (34%) continued intravenous (IV) bisphosphonates on the same dose as in paediatric care, 2 (3%) continued IV bisphosphonates at reduced doses from paediatric care, 4 (7%) had infusions stopped entirely, and 5 (8%) were switched from IV to oral bisphosphonates.

**CONCLUSIONS:** For young people with OI transition is an important process in moving from paediatric to adult healthcare services. These data reflect decisions made in the young person's first transition appointment. Most patients had type 1 OI and of those receiving bisphosphonates in any form, 89% continued receiving IV or oral forms. Our data suggest a tailored approach to bisphosphonate treatment across transition is required, involving discussion with the young person and their paediatric and adult teams. Further study including the young person's final transition appointment, bone mineral density and OI severity is planned, to fully evaluate the role of bisphosphonates for OI management in young adulthood.

### LB6

### Hypocalcemic rickets in a 19-month-old boy due to an activating variant on *CYP3A4*

Saila Laakso, Matti Hero, Helena Valta

Helsinki University Hospital and University of Helsinki, Helsinki, Finland

### Abstract

BACKGROUND: 19-month-old second child of the family was born full-term with birth size appropriate for gestational age. Growth and development had been normal until the age of 1 year. At the age of 11 months, he had started to stand and take steps against support. Normal nutrition with 0.5 L milk daily. He had received daily 10 microg vitamin D3 supplement from the age of 2 weeks. Family members were healthy.

PRESENTING PROBLEM: During the last 7 months, he did not want to stand or take steps and genu varum developed. His height Z-score declined from -1.0 at the age of 1 year to -2.0, mid-parental target height being +0.8 Z-scores.

His general state of health was good. Wide metaphyses were evident in wrists, ankles, knees, and ribs. Intermalleolar distance was 7 cm. Teeth were normal. Laboratory test showed hypocalcemia and low vitamin D levels (Table). In x-ray images, clear signs of rickets were seen in wrists, knees and ribs. Electrocardiogram and heart ultrasound were normal.

Exome sequencing revealed *CYP3A4* variant c.902T>C, (p.I301T) that has been reported previously in four patients with vitamin D-dependent rickets type 3.

CLINICAL MANAGEMENT: Treatment was started with alfacalcidol and calcium supplementation. Vitamin D3 supplementation was increased to 50 ug/day and 1250 ug was given i.m. without any response in D-25-OH levels. Dose of alfacalcidol was gradually increased to 1.6 ug/day and calciumcarbonat to 2000 mg/day. After ALP and PTH normalized, calcium supplementation was decreased to 750 mg/day. Signs of rickets healed gradually based on x-ray imaging. After 3 months of treatment, he started to walk without support. Height Z-score increased to -0.7.

DISCUSSION: Increased inactivation of vitamin D due to *CYP3A4* variant caused active hypocalcemic rickets in a 19-month-old boy. This case underscores the necessity for tailored therapeutic strategies in genetic disorders of vitamin D metabolism.

Table. Biochemical analyses

| Variable | Value | Normal range |
|---|---|---|
| Ionised calcium (mmol/L) | 0.75 | 1.17-1.35 |
| Phosphate (mmol/L) | 1.37 | 1.3-2.2 |
| ALP (U/L) | 1260 | 115-460 |

| | | |
|---|---|---|
| PTH (ng/L) | 643 | 18-80 |
| Creatinine (umol/L) | 23 | 13-56 |
| Venal blood pH | 7.37 | 7.32-7.42 |
| 25-(OH)D (nmol/L) | <10 | ›50 |
| 1,25-(OH)D (pmol/L) | 62 | 113-363 |
| U-Ca/Crea | 0.17 | ‹0.7 |

### LB7

### Rare causes of osteogenesis imperfecta: a cross-sectional study of skeletal features and treatment response in a Turkish cohort

Asli Derya Kardelen Al[1], Kubra Demir[2,3], Ayse Pinar Ozturk[1], Ayca Aslanger[4], Cagri Gulec[4], Elif Bahar Tuna Ince[5], Firdevs Bas[1], Guven Toksoy[4]

[1]Istanbul University, Department of Pediatric Endocrinology, Istanbul, Turkey. [2]Istanbul University, Institute of Graduate Studies in Health Sciences, Department of Genetics, Istanbul, Turkey. [3]Istanbul University, Institude of Graduate Studies in Health Sciences, Department of Restorative Dentistry, Istanbul, Turkey. [4]Istanbul University, Department of Genetics, Istanbul, Turkey. [5]Istanbul University, Department of Pediatric Dentistry, Istanbul, Turkey

### Abstract

**Objectives:** Osteogenesis imperfecta(OI) is a rare inherited disorder characterized by recurrent fragile fractures. Here, we present genetic alterations detected in *SERPINF1, FKBP10, P3H1,* and *IFITM5* genes in a cohort of OI patients. Our results provide additional information about the pattern of disease and response to treatment in rare forms of OI.

**Methods:** A total of 38 patients with OI who received outpatient care at Istanbul University from 2006 to 2024 were included in the present study. Pamidronate treatment was given to all patients with a total of 9 mg/kg per year. Clinical features and treatment responses were analyzed retrospectively.

**Results:** Using whole-exome sequencing, we identified mutations in *COL1A1*(n=17), *COL1A2*(n=13), *SERPINF1*(n=3), *FKBP10*(n=2), *IFITM5*(n=2) and *P3H1*(n=1) genes. When we evaluate the rare causes in detail (8 patients; F/M=4/4), median age of the patients at presentation was 0.8 (0.3and6.5) years, median height SDS was -1.6 (-10.3and-0.4) and median BMI SDS was -0.14 (-1.7and3.6). The characteristics of the patients are summarized in Table 1. Three patients had prenatal fractures. Patients' initial BMD z score was -4.8 (-7.1and-3.1) and pamidronate treatment was given for an average of 5.8 (3.5and10) years. Patient 1 received alternate treatment with denosumab and bisphosphonate for the last 1.5 years, and Patient 2 used alendronate for the last 1.5 years. The treatment of all patients was interrupted intermittently, especially Patient 6 (6 years) and Patient 8 (2 years) were lost to follow-up for a long time. All patients had scoliosis and vertebral compression fractures. At the final evaluation, the patients' height SDS was -3.3 (-13.1 and0.3), BMI SDS was -0.4(-1.4 and1.9) and BMD z score was -1.2 (-6.4and1.1). Seven patients were wheelchair-bound and had severe deformities. Especially patients with *SERPINF1* and *P3H1* had severe deformities and did not respond significantly to treatment. Two patients with *IFITM5* mutations were better progressing and had fewer deformities.

**Conclusion:** The osteoporosis and deformities in *SERPINF1* and *P3H1* patients were treatment-resistant. New treatment options should be explored in this area. Starting new treatment options from an early age can prevent the development of deformities and contribute to the quality of life of patients with OI.

Table 1. Clinical characteristics of the patients

| | Patient 1 | Patient 2 | Patient 3 | Patient 4 | Patient 5 | Patient 6 | Patient 7 | Patient 8 |
|---|---|---|---|---|---|---|---|---|
| **Presentation** | | | | | | | | |
| Age year | 0.83 | 6.5 | 1.5 | 0.7 | 0.4 | 6.1 | 0.3 | 0.7 |
| Gender | M | M | F | F | F | F | M | M |
| Height SDS | -1.4 | -2.1 | -0.4 | -1.05 | -1.7 | -10.3 | -1.1 | -2.0 |
| Consanguinity | + | + | + | + | + | + | + | - |
| BMİ SDS | -0.4 | -1.5 | 3.6 | -1.7 | 0.12 | 1.1 | 0.32 | -0.4 |
| Prenatal fracture | - | - | - | + | + | + | - | - |
| Age of first fracture | 0.6 | 1.5 | 0.7 | at birth | PN 2 days | at birth | 0.2 | at birth |
| Total number of fractures | 3 | 25 | 2 | 3 | 3 | multiple | 4 | 2 |
| Blue sclera | + | + | + | + | - | + | + | + |
| L1-L4 z score | -4.4 | -3.1 | -4.7 | -7.05 | -3.5 | -4.8 | -7.06 | -4.9 |
| First treatment age (year) | 1.1 | 7.2 | 2 | 1 | 1.1 | 7.1 | 1.3 | 1.8 |
| Gene mutation | SERPINF1 c.620T>G (p.Leu207Arg) | SERPINF1 c.188dup (p.Tyr63*) | SERPINF1 c.364_365insATA (p.Y122delinsYN) | FKBP10, c.897_898insTGATGGAC (p.Gly300*) | FKBP10, c.898_900delGGCinsTGA (p.Gly300*) | P3H1, c.2055+18G>A (p.Arg685_Asp686insVRAARAA Asp.686fs) | IFITM5, c.-14C>T | IFITM5, c.-14C>T |
| Zygosity | homozygous | homozygous | homozygous | homozygous | homozygous | homozygous | heterozygous | heterozygous |
| **Last evaluation** | | | | | | | | |
| Age years | 6.5 | 21.8 | 14.6 | 12.2 | 5.5 | 19 | 8 | 14.4 |
| Height SDS | -5.8 | -3.4 | # | -3.3 | -2.3 | -13.1 | 0.3 | -1.3 |
| BMI SDS | 0.5 | -0.4 | # | -1.0 | -1.2 | 1.9 | 0.02 | -1.4 |
| L1-L4 z score | -6.4 | 1.1 | -0.6 | -1.17 | -1.7 | -1.3 | -0.1 | -2.2 |

| | | | | | | | | |
|---|---|---|---|---|---|---|---|---|
| Total biphosphanate | Pamidronate 8/denosumab 3 times | Pamidronate 25 times/1.5 years alendronate | Pamidronate 23 times | Pamidronate 27 times | Pamidronate 9 times | Pamidronate 10 times | Pamidronate 17 times | Pamidronate 16 times |
| Total treatment duration (year) | 4 | 10 | 9 | 10 | 3.5 | 6 | 5.5 | 5.5 |
| Mobility | Wheelchair-bound | Wheelchair-bound | Wheelchair-bound | Wheelchair-bound | Wheelchair-bound | Wheelchair-bound | Mobile with mild deformities | Wheelchair-bound |
| Scoliosis | + | + | + | + | + | + | + | + |
| Hearing impairment | - | - | - | - | - | - | - | - |
| Vertebral compression | + | + | + | + | + | + | + | + |
| Intelligence | normal | normal | normal | normal | normal | normal | normal | normal |
| Total fracture | 10 | multiple | multiple | 15 | 25 | multiple | multiple | 8 |

### could not be measured because of deformities

### LB8

### Cessation of burosumab treatment due to end of skeletal growth (EoSG) in adolescents with XLH: A multi-centre case series

Charlotte Jarvis[1], Renuka Ramakrishnan[1], Poonam Dharmaraj[1], Talat Mushtaq[2], Sanjay Gupta[3], Annabel Nixon[4], Angela Rylands[5], Suma Uday[6]

[1]Alder Hey Children's NHS Foundation Trust, Liverpool, UK [2]Leeds Teaching Hospitals NHS Trust, Leeds, UK [3]Hull University Teaching Hospital, Hull, UK [4]Chilli Consultancy, Salisbury, UK [5]Kyowa Kirin Ltd, Marlow, UK [6]Birmingham Women's and Children's Hospital, Birmingham, UK

### Abstract

**OBJECTIVES:** X-linked hypophosphataemia (XLH), a genetic condition of renal phosphate wasting secondary to excess FGF23 leads to significant musculoskeletal impairments. The National Health Service in England and Wales recommend burosumab (anti-FGF23 antibody) for treating XLH in children and young people with growing bones, with radiographic evidence of bone disease. This multi-centre case series captures real-world clinician recorded experience of burosumab cessation in adolescents at the end of skeletal growth (EoSG).

**METHODS:** A retrospective descriptive case series of eight adolescents with XLH from four centres in England capturing medical history, biochemical data, radiological findings, and narratives of patient and caregiver reported experiences from clinical notes.

**RESULTS:** All patients switched to burosumab treatment from conventional therapy (oral phosphate and active Vitamin D) between 9-16 years. Median plasma phosphate levels at the end of conventional therapy were 0.59 mmol/L (n=7). Median burosumab treatment duration was 39 months (range 22-56 months) during which plasma phosphate levels increased to within or close to normal range for each patient (median 0.87 mmol/L, n=8). The reported benefits of burosumab treatment included leg straightening, improved bone biochemistry, pain, stiffness, mobility, energy, activity levels and mental health. Additionally, clinicians reported quicker recovery from orthopaedic surgery, improved school attendance and quality of life.

Following cessation of burosumab at EoSG, plasma phosphate levels decreased to below the lower limit of normal (median 0.60 mmol/L, n=6). Six of the eight adolescents reported that pain returned or worsened within a few months of stopping burosumab. Six reported reductions in mobility and physical function, with increased pain. Decreased mobility compromised patients' ability to participate in physical education, physical activity, work/college and social activities. Five reported that stopping burosumab treatment had a marked impact on their mental wellbeing. Five restarted treatment with conventional therapy.

**CONCLUSIONS:** Our case series provides insights into the positive experience of patients switching to burosumab treatment from conventional treatment which supports published evidence. Stopping burosumab treatment at EoSG had an adverse impact on pain, mobility and mental health in the majority of adolescents.

### Disclosure

AR is an employee of Kyowa Kirin Ltd, the study sponsor AN conducted the data analysis and writing support and is the CEO of Chilli consultancy that is funded by Kyowa Kirin Ltd

### LB9

### Camurati-Engelman disease - the long way to the diagnosis: Clinical case

Adelia Iskalieva[1], Eugenia Isupova[1], Ilia Avrusin[1], Evgeny Suspitsin[1,2], Mikhail Kostik[1]

[1]Saint Petersburg State Pediatric Medical University, Saint Petersburg, Russian Federation. [2]N. N. Petrov Institute of Oncology, Saint Petersburg, Russian Federation

### Abstract

**Background**. Camurati-Engelmann disease (CED) is a rare systemic skeletal disease based on a mutation in the TGFB1 gene, which is responsible for cell proliferation and differentiation. CED is inherited in an autosomal dominant manner and it is characterized by systemic skeletal damage, myopathy, and pain syndrome. According to GeneReviews, as of 2023, 306 cases of the disease have been registered in the world.

**Presenting problem**

The 5-year-old girl was admitted to the clinic with the diagnosis "fibrous dysplasia (polyostotic form)". Upon admission, there were complaints of bone pain, sleep disturbances and a constant requirement in NSAIDs. During an objective examination, 2 café-au-lait lesions were noted on the skin of torso, hypermobility of joints; pain on palpation in the thoracic spine, sacroiliac joint; limitation of internal rotation in the hip joints, feet valgus position, rickets-like chest deformity, and scoliotic posture disorder were mentioned. For the first time, parents noticed the asymmetry of the child's face, pain in the limbs, neck and chest at the age of 3 months. On the X-ray in April 2021 there was revealed multifocal damage to the tubular bones with areas of osteosclerosis and hyperostosis of the cortical layer (fig.1). In May 2022 bone biopsy and punch skin biopsy were performed. The diagnosis was interpreted as "Chronic multifocal osteomyelitis, polyostotic form."

**Clinical management**

A genome sequencing has revealed heterozygous c.512A>G variant in TGFB1 gene and CED was identified. The patient was recommended to continue treatment with bisphosphonates and discuss the possibility of adding gabapentin and denosumab if their effectiveness was insufficient.

**Discussion**

In patients with metabolic and rheumatic diseases of the skeleton, a wide range of differential diagnostics is required, including rare and ultra-rare genetic syndromes. When examining children with metabolic diseases of the skeleton, it is reasonable to conduct a molecular genetic study, which makes it possible to establish a final diagnosis.

**Funding information:** the work was supported by Russian Science Foundation grant RSF 24-45-00067

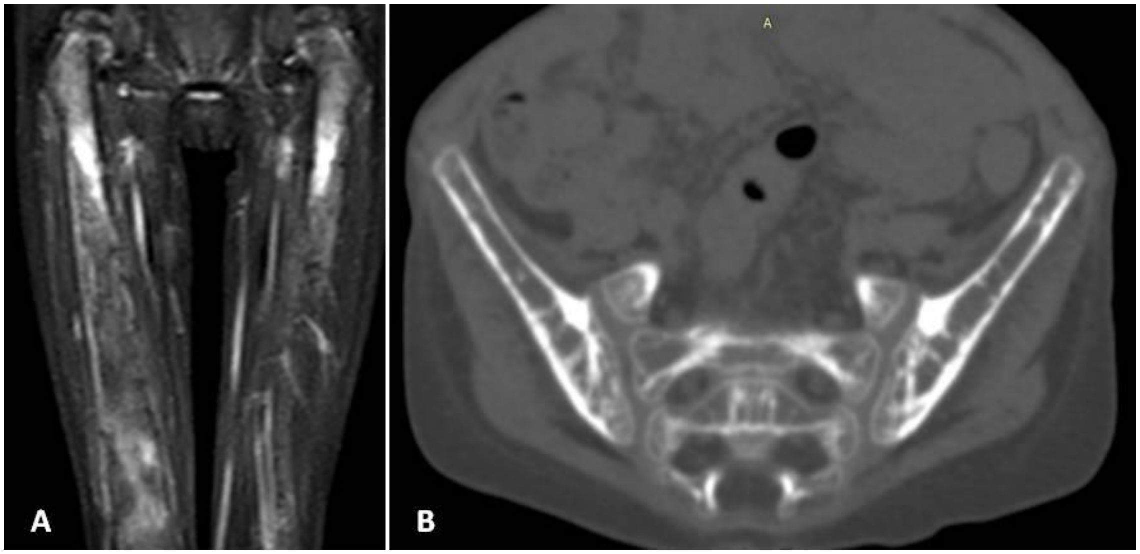

**Figure 1:** A) Bone marrow edema of both hips (MRI); B) CT of pelvis shows bone hyperostosis and multiple lesions of pelvic bones.

**LB10**

Withdrawn

### LB11

### Specific primary osteoporosis and enamel dysplasia: Expanding the spectrum of metaphyseal dysplasia and maxillary hypoplasia with brachydactyly associated with duplication in RUNX2

Thomas Zoller[1,2], Maelle Charpie[1], Graziella Pinto[3], Zagorska Pejin[4], Martin Biosse Duplan[5], Eugénie Koumakis[6], Valerie Malan[1], Sophie Rondeau[1], Serge Romana[1], Valerie Cormier-Daire[1], Genevieve Baujat[1]

[1]Université Paris Cité, Reference Center for Skeletal Dysplasia, Service de Médecine Génomique des Maladies Rares, Hôpital Universitaire Necker-Enfants malades, AP-HP, Paris, France. [2]Department of Surgical Sciences, Dentistry, Gynecology and Pediatrics, Pediatric Clinic, School of Medicine, University of Verona, Veronq, Italy. [3]Service d'Endocrinologie Pédiatrique, Hôpital Universitaire Necker-Enfants malades, AP-HP, Paris, France. [4]Département d'Orthopédie Pédiatrique, Hôpital Universitaire Necker-Enfants Malades, AP-HP, Paris, France. [5]Département de Médecine Dentaire, Hôpital Bretonneau, AP-HP, Paris, France. [6]Reference Center for Skeletal Dysplasia, Service de Rhumatologie, Hôpital Cochin, AP-HP, Paris, France

### Abstract

BACKGROUND

Skeletal primary disorders with bone fragility are characterized by variable degree of fractures, pain, skeletal deformities and other specific symptoms according to the various molecular causes. Within this group, can be included Metaphyseal Dysplasia and Maxillary Hypoplasia with or without Brachydactyly (MDMHB, OMIM 156510).

This condition has been described in the literature in only 16 patients, having in common an intragenic duplication of the RUNX2 gene. *RUNX2* encodes CBFA1, a master transcription factor, regulating both osteoblasts and terminal chondrocytes differentiation, essential for bone development and mineralization.

Clinically, MDMHB is characterized by variable bone and dental features, including metaphyseal flaring, thin diaphyseal cortices, wormian bones, osteoporosis, brachydactyly, enlargement of the proximal portion of the clavicles, facial features, and amelogenesis imperfecta.

PRESENTING PROBLEM

Considering this ultra-rare specific condition with low bone mass, dysmorphism and yellowish teeth, we have identified, within our patients with atypical osteoporosis followed in the Centre of Reference for Skeletal Dysplasia, 5 patients from 3 unrelated families, presenting with clinical and radiographic features resembling MDMHB. Patients A and B are siblings from unrelated parents, and were referred for painful gait and nasal obstruction at 9 years. Patients C and D, are also siblings, and C was referred for painful vertebral compression fractures, leading to the suspicion of OI at 3 years. The last one, E, was diagnosed at only 33 years, because of multiple recent fractures, amelogenesis imperfecta and lachrymal obstruction.

MANAGEMENT

CGH array and Q-PCR detected a *RUNX2* duplication spanning exon 2 to exon 5 for patients C, D and E, and a duplication spanning exon 2 to exon 6 for patients A and B, resulting in all cases in the duplication of the functional CBFA1 QA and RUN domain. In the two families (A-B and C-D), RT-PCR detected that the father was mosaic for this *RUNX2* duplication.

DISCUSSION

We describe deeply the clinical and radiographic phenotypes between our 5 patients and compared their features with the previously published patients. We highlight the main features of this recognizable phenotype including both osteopenia and osteosclerosis with abnormal clavicles, and amelogenesis imperfecta.

### LB12

### The impact of palovarotene on patients with fibrodysplasia ossificans progressiva: Results from participant interviews in the phase III PIVOINE trial

Edna E. Mancilla[1], Geneviève Baujat[2], Mona Al Mukaddam[3], Javier Bachiller-Corral[4], Staffan K Berglund[5], Carmen De Cunto[6], Patricia Delai[7], Richard Keen[8], Riccardo Papa[9], Robert J Pignolo[10], Sally Lanar[11], Alexia Marrel[11], Alexander Artyomenko[12], Kim Croskery[12], Christelle Pommie[13], Edward C Hsiao[14]

[1]Children's Hospital of Philadelphia, Perelman School of Medicine, University of Pennsylvania, Philadelphia, Pennsylvania, USA [2]Service de Médecine Génomique des Maladies Rares, Hôpital Necker-Enfants Malades, Paris, France. [3]Departments of Orthopaedic Surgery and Medicine, Centre for Research in FOP and Related Disorders, Perelman School of Medicine, University of Pennsylvania, Philadelphia, USA [4]Department of Rheumatology, Hospital Universitario Ramón y Cajal, Madrid, Spain. [5]Department of Clinical Sciences, Paediatrics, Umeå University, Umeå, Sweden. [6]Paediatric Rheumatology Section, Department of Paediatrics, Hospital Italiano de Buenos Aires, Buenos Aires, Argentina. [7]Centro de Pesquisa Clinica, Hospital Israelita Albert Einstein, São Paolo, Brazil. [8]Centre for Metabolic Bone Disease, Royal National Orthopaedic Hospital, Stanmore, UK [9]UOC Reumatologia e Malattie Autoinfiammatorie, IRCCS Istituto Giannina Gaslini, Genova, Italy. [10]Department of Medicine, Mayo Clinic, Rochester, Rochester, Minnesota, USA [11]ICON plc, Insights, Evidence and Value, Patient-Centred Outcomes, Lyon, France. [12]Ipsen, London, UK [13]Ipsen, Boulogne-Billancourt, France. [14]Division of Endocrinology and Metabolism, the UCSF Metabolic Bone Clinic, the Eli and Edyth Broad Institute for Regeneration Medicine, and the Institute of Human Genetics, Department of Medicine, and the UCSF Program in Craniofacial Biology, University of California San Francisco,, San Francisco, California, USA

### Abstract

OBJECTIVES

Fibrodysplasia ossificans progressiva (FOP) is an ultra-rare genetic disorder characterised by progressive heterotopic ossification (HO). Palovarotene is the first approved treatment for the reduction of new HO in patients with FOP. Here, patient interviews in the open-label, phase III PIVOINE trial (NCT05027802) are presented to better understand patients' experiences with FOP and palovarotene.

METHODS

Cross-sectional, qualitative, semi-structured interviews (~45 minutes) were conducted by the Principal Investigator or trained staff ideally within four weeks of trial inclusion, or up to 1-year following entry. Transcripts were analysed qualitatively to extract key concepts and themes. Changes over time were explored, for patients' feelings related to FOP, daily activities, need for assistive devices and their need for help from others, by asking patients to recall a time before they received palovarotene and comparing this to the time of the interview when patients were receiving palovarotene.

RESULTS

Of 61 patients enrolled in PIVOINE, 51 completed interviews, including 14 adolescent patients (aged 14–17 years). Of interviewed patients, 24/51 (47.1%) recalled feelings of fear and worry for the future before they received palovarotene. When patients were receiving palovarotene, 27/51 (52.9%) expressed feelings of hope and confidence in the future; 17/51 (33.3%) were hopeful because of the new treatment option palovarotene represented. Compared with before patients received palovarotene, around half of interviewed patients were classed as having a worsened impact on daily activities (29/51; 56.9%), need for assistive devices (23/51; 45.1%) and help from others (22/51; 43.1%) when they received palovarotene; however, approximately a quarter of patients were categorized as remaining stable (11/51 [21.6%], 13/51 [25.5%] and 14/51 [27.5%], respectively) and some as improving (5/51 [9.8%], 2/51 [3.9%] and 4/51 [7.8%], respectively). Around two-thirds of patients (31/51; 60.8%) indicated that the benefits of palovarotene greatly outweighed the negatives, most commonly due to palovarotene stopping or reducing flare-up frequency and/or new bone growth (24/51; 47.1%).

CONCLUSIONS

These interviews showed that even in this progressive disease, one-third of patients with FOP remained hopeful for the future because of the treatment option palovarotene represented. Further studies are needed to fully determine palovarotene's impact on the rate of disease progression.

### Disclosure

EEM: Research Investigator: Clementia/Ipsen, Incyte; Consultant: Ipsen; GB: Research Investigator: Clementia/Ipsen, Incyte, BioMarin, Alexion; Advisory Board: Clementia/Ipsen, Inozyme, BioMarin; Speaker: Clementia/Ipsen, FOP European Consortium, International Clinical Council on FOP, IFOPA Registry Medical Advisory Board (all voluntary); MAM: Research Investigator: Clementia/Ipsen, Incyte, Regeneron; Non-paid consultant: Biocryst, Blueprint, Daiichi Sankyo, Incyte, Keros; Advisory Board: IFOPA Registry Medical Advisory Board, International Clinical Council on FOP (all voluntary); JBC: Research Investigator: Clementia/Ipsen, Incyte, Regeneron; Advisory Board: Clementia/Ipsen; SKB: Research Investigator: Clementia/Ipsen; Speaker: Nestlé Nutrition, Nutricia, Semper; Funding: Mead Johnson Nutrition; CDC: Research Investigator: Clementia/Ipsen; Member of the International Clinical Council on FOP; PD: Research Investigator: Clementia/Ipsen; Member of the International Clinical Council on FOP; RK: Research Investigator: Clementia/Ipsen, Kyowa Kirin, Regeneron; Advisory Board: IFOPA Registry Medical Advisory Board, International Clinical Council on FOP; RP: No conflicts of interest to disclose; RJP: Research Investigator: Clementia/Ipsen, Incyte, Regeneron; Advisory Board: Past President of the International Clinical Council on FOP; SL, AM: Employees of ICON; AA, KC: Employees and shareholders of Ipsen; CP: Employee of Ipsen; ECH: Research support: Ascendis, Clementia/Ipsen, Ultragenyx, Ashibio; Research Investigator: Ascendis, Clementia/Ipsen, Ashibio; Member of the International Clinical Council on FOP, Fibrous Dysplasia Foundation, and IFOPA Registry Advisory Board (all voluntary).

### LB13

### Incidence and management of skin adverse events in patients receiving palovarotene for the treatment of fibrodysplasia ossificans progressiva

Patricia Delai[1], Mona Al Mukaddam[2], Geneviève Baujat[3], Carmen De Cunto[4], Edward C Hsiao[5], Richard Keen[6], Kim Croskery[7], Jean-Luc Delhay[8], Christelle Pommie[8], Robert J Pignolo[9]

[1]Centro de Pesquisa Clinica, Hospital Israelita Albert Einstein, São Paulo, Brazil. [2]Departments of Orthopaedic Surgery and Medicine, Centre for Research in FOP and Related Disorders, Perelman School of Medicine, University of Pennsylvania, Philadelphia, Pennsylvania, USA [3]Service de Médecine Génomique des Maladies Rares, Hôpital Necker-Enfants Malades, Paris, France. [4]Paediatric Rheumatology Section, Department of Paediatrics, Hospital Italiano de Buenos Aires, Buenos Aires, Argentina. [5]Division of Endocrinology and Metabolism, the UCSF Metabolic Bone Clinic, the Eli and Edyth Broad Institute for Regeneration Medicine, and the Institute of Human Genetics, Department of Medicine, and the UCSF Program in Craniofacial Biology, University of California San Francisco, San Francisco, USA [6]Centre for Metabolic Bone Disease, Royal National Orthopaedic Hospital, Stanmore, UK [7]Ipsen, London, UK [8]Ipsen, Boulogne-Billancourt, France. [9]Department of Medicine, Mayo Clinic, Rochester, Minnesota, USA

### Abstract

OBJECTIVES

Fibrodysplasia ossificans progressiva (FOP) is an ultra-rare genetic disorder characterised by heterotopic ossification (HO). Palovarotene, a selective retinoic acid receptor-γ agonist, is approved for reduction of new HO in patients with FOP, supported by the phase III MOVE trial (NCT03312634). In MOVE, palovarotene was generally well tolerated; the most common treatment-emergent adverse events (TEAEs) were skin-related, consistent with known effects of systemic retinoids. Here, post hoc analyses of the incidence, duration and intensity of skin TEAEs in patients receiving palovarotene in MOVE, with and without skin prophylaxis, are reported.

METHODS

Patients in MOVE received palovarotene 5 mg once-daily (chronic), increasing to 20 mg for 4 weeks then 10 mg for ≥8 weeks during flare-ups (weight-adjusted if skeletally immature). Prophylactic therapies could be used to manage skin TEAEs. Skin TEAEs (System Organ Class: “skin and subcutaneous disorders” or Preferred Term: “lip dry”) were recorded based on MedDRA Preferred Term (version 22.0), characterised using the Standardised MedDRA Query of Severe Cutaneous Adverse Reactions (broad and narrow) and graded using the Common Terminology Criteria for Adverse Events (version 4.03). Patients who reported ≥1 skin TEAE during MOVE were included in the post hoc analyses, encompassing data from screening to final data cut-off (19 December 2022).

RESULTS

Of 97 patients, 20 (20.6%) had a medical history of skin and subcutaneous tissue disorders. The most commonly occurring skin TEAEs during palovarotene treatment included dry skin, dry lips, alopecia, drug eruption and rash (Table). Prophylaxis generally shortened the median duration of these events in patients receiving chronic and flare-up treatment versus those not receiving prophylaxis; this was not the case for drug eruption and rash with chronic treatment and dry skin with flare-up treatment (Table). Skin TEAEs were mostly mild or moderate for both dosing regimens; in patients receiving flare-up treatment, prophylaxis generally reduced TEAE intensity (Table). Skin TEAEs led to palovarotene dose modification in 29 (29.9%) patients and drug withdrawal in 2 (2.1%) patients.

CONCLUSIONS

Management of skin TEAEs with prophylaxis may reduce the intensity and duration of these events, especially in patients receiving flare-up palovarotene treatment, which may help to improve treatment adherence.

**Table:** Most common skin TEAEs by Preferred Term in patients receiving chronic and flare-up palovarotene treatment, with and without skin prophylaxis.

| | Chronic treatment (N=97)[a] | | | | | | Flare-up treatment (N=76)[b] | | | | | |
|---|---|---|---|---|---|---|---|---|---|---|---|---|
| | With skin prophylaxis[c,d] | | | Without skin prophylaxis | | | With skin prophylaxis[c,d] | | | Without skin prophylaxis | | |
| **Any skin TEAE**, n (%) [number of events] | 48 (49.5) [217] | | | 54 (55.7) [160] | | | 39 (51.3) [215] | | | 30 (39.5) [136] | | |
| **Most common skin TEAEs,** n (%) [number of events][e] | | | | | | | | | | | | |
| Dry skin | 31 (32.0) [54] | | | 30 (30.9) [37] | | | 19 (25.0) [37] | | | 17 (22.4) [39] | | |
| Dry lips | 15 (15.5) [15] | | | 20 (20.6) [20] | | | 12 (15.8) [12] | | | 5 (6.6) [5] | | |
| Alopecia | 7 (7.2) [8] | | | 11 (11.3) [13] | | | 9 (11.8) [13] | | | 10 (13.2) [12] | | |
| Drug eruption[f] | 9 (9.3) [13] | | | 6 (6.2) [10] | | | 16 (21.1) [26] | | | 10 (13.2) [14] | | |
| Rash | 13 (13.4) [18] | | | 11 (11.3) [15] | | | 7 (9.2) [13] | | | 3 (3.9) [6] | | |
| **AE duration**, days, median (range) | | | | | | | | | | | | |
| Dry skin | 85.0 (1–1360) | | | 107.0 (2–1249) | | | 43.0 (5–770) | | | 35.0 (5–515) | | |
| Dry lips | 479.0 (14–645) | | | 485.5 (7–1130) | | | 45.0 (22–806) | | | 82.5 (71–94) | | |
| Alopecia | 304.0 (45–1214) | | | 331.0 (8–729) | | | 91.5 (17–318) | | | 142.0 (8–274) | | |
| Drug eruption | 23.5 (3–77) | | | 19.0 (6–171) | | | 22.5 (7–132) | | | 71.0 (19–564) | | |
| Rash | 25.5 (3–440) | | | 16.0 (3–437) | | | 15.0 (1–104) | | | 18.0 (8–328) | | |
| **AE intensity**, number of events (%) | Mild | Moderate | Severe | Mild | Moderate | Severe | Mild | Moderate | Severe | Mild | Moderate | Severe |
| Dry skin | 53 (98.1) | 1 (1.9) | 0 | 34 (91.9) | 3 (8.1) | 0 | 33 (89.2) | 3 (8.1) | 1 (2.7) | 24 (61.5) | 15 (38.5) | 0 |
| Dry lips | 15 (100.0) | 0 | 0 | 15 (75.0) | 5 (25.0) | 0 | 12 (100.0) | 0 | 0 | 4 (80.0) | 1 (20.0) | 0 |
| Alopecia | 8 (100.0) | 0 | 0 | 13 (100.0) | 0 | 0 | 12 (92.3) | 1 (7.7) | 0 | 10 (83.3) | 2 (16.7) | 0 |
| Drug eruption | 9 (69.2) | 4 (30.8) | 0 | 10 (100.0) | 0 | 0 | 15 (57.7) | 10 (38.5) | 1 (3.8) | 8 (57.1) | 6 (42.9) | 0 |
| Rash | 16 (88.9) | 2 (11.1) | 0 | 12 (80.0) | 3 (20.0) | 0 | 13 (100.0) | 0 | 0 | 6 (100.0) | 0 | 0 |

Skin AEs were defined by a "Skin and subcutaneous tissue disorders" System Organ Class or a "Lip dry" Preferred Term. One TEAE could only occur during chronic or flare-up treatment. [a]Treatment emergence for chronic treatment was defined as any AE with an onset date on or after first dose date of chronic treatment and up to seven days past the date of last dose of chronic treatment. [b]Treatment emergence for flare-up treatment was defined as any AE with an onset date on or after first dose date of flare-up treatment and up to seven days past the date of last dose of flare-up treatment. [c]Only TEAEs occurring while on skin concomitant medications taken as prophylaxis that started or were ongoing seven days prior to start date of TEAEs were included. [d]Most common types of prophylaxis used were soft paraffin and fat products (23 [23.7%]), other emollients and protectives (17 [17.5%]), protectives against UV radiation for topical (14 [14.4%]), Avena sativa fluid extract (10 [10.3%]), Vanicream (6 [6.2%]) and dimeticone (6 [6.2%]). [e]If a patient experienced more than one event in a category, the patient was counted only once in that category. Percentages were calculated with the number of patients in each treatment group as the denominator. [f]Drug eruption was the MedDRA Preferred Term for "retinoid dermatitis", defined as a triad of generalised rash, dry skin and erythema with onset of all three findings within 2–3 days. AE: adverse event; MedDRA: Medical Dictionary for Regulatory Activities; TEAE: treatment-emergent AE.

### Disclosure

PD: Research Investigator: Clementia/Ipsen; Member of the International Clinical Council on FOP; MAM: Research Investigator: Clementia/Ipsen, Incyte, Regeneron; Non-paid consultant: Biocryst, Blueprint, Daiichi Sankyo, Incyte, Keros; Advisory Board: IFOPA Registry Medical Advisory Board, International Clinical Council on FOP (all voluntary); GB: Research Investigator: Clementia/Ipsen, Incyte, BioMarin, Alexion; Advisory Board: Clementia/Ipsen, BioMarin, Inozyme; Speaker: Clementia/ Ipsen; FOP European Consortium, International Clinical Council on FOP, IFOPA Registry Medical Advisory Board (all voluntary); CDC: Research Investigator: Clementia/Ipsen; Member of the International Clinical Council on FOP; ECH: Research support: Ascendis, Clementia/Ipsen, Ultragenyx, Ashibio; Research Investigator: Ascendis, Clementia/Ipsen, Ashibio; Member of the International Clinical Council on FOP, Fibrous Dysplasia Foundation, and IFOPA Registry Advisory Board (all voluntary); RK: Research Investigator: Clementia/Ipsen, Kyowa Kirin, Regeneron; Advisory Board: IFOPA Registry Medical Advisory Board, International Clinical Council on FOP; KC: Employee and shareholder of Ipsen; JLD, CP: Employees of Ipsen; RJP: Research Investigator: Clementia/Ipsen, Incyte, Regeneron; Advisory Board: Past President of the International Clinical Council on FOP.

### LB14

### A pediatric patient with osteoma cutis due to a variant in *GNAS*

Esra Bal Yüksel[1], Durdugül Ayyıldız Emecen[2], İbrahim Mert Erbaş[1], Özlem Nalbantoğlu[1], Behzat Özkan[1]

[1]University of Health Sciences Dr Behçet Uz Children's Hospital, Department of Pediatric Endocrinology, İzmir, Turkey. [2]University of Health Sciences Dr Behçet Uz Children's Hospital, Department of Pediatric Genetics, İzmir, Turkey

### Abstract

**BACKGROUND:** Osteoma cutis is characterized by bone formation in the dermis or subcutaneous tissue and rarely seen in childhood. This condition may occur due to primary or secondary causes. Inactivating variants in *GNAS* gene can cause diverse phenotypes, including primary osteoma cutis in a small number of patients. Herein, we presented a pediatric patient of osteoma cutis associated with a variant in *GNAS*.

**PRESENTING PROBLEM:** An 8-year-old female patient applied to our outpatient clinic due to short fingers and mobile nodules under the skin. Her past medical and family history revealed nothing and her neuromotor development was consistent with her peers. Her body weight, height, and body mass index were measured as 20.5 kg (-1.1 SDS), 116.4 cm (-1.5 SDS), and 15.1 kg/m2 (-0.4 SDS), respectively. She was prepubertal and her physical examination revealed hypertelorism, proptosis of the right eye, low-set ears, micrognathia, mobile painful nodules with medium-hard consistency on the medial of the hands and wrists, and shortness of the 4th and 5th metacarpals of both hands and feet.

**CLINICAL MANAGEMENT:** Laboratory tests showed normal serum calcium, phosphorus, parathormone, and alkaline phosphatase levels. Other endocrinological investigations were normal for her age and no hormone resistance was found. X-ray of her hands demonstrated heterotopic ossifications and metacarpal shortness (brachydactyly type E). A pathogenic heterozygous variant (c.432+1G>A) in *GNAS* gene was found in the genetic analysis. The segregation analysis of her parents revealed no variants. Since our patient did not have two or more of the phenotypic features of Albright's hereditary osteodystrophy, she was followed up with a diagnosis of primary osteoma cutis.

**DISCUSSION:** Osteoma cutis, a member of *GNAS*-related disease spectrum, is very rare in childhood. In these patients, close monitoring and differential diagnosis are important in terms of the development of progressive osseous heteroplasia. With this report, we demonstrated that variants in *GNAS* may present with only skeletal findings without any endocrinological disorders such as pseudohypoparathyroidism or any other hormone resistance. Children with brachydactyly and subcutaneous nodules due to heterotopic ossifications should be evaluated with *GNAS* gene analysis in this respect.

### LB15

### ANDECAL - a phase 2/3 study to determine the efficacy of the MMP9-inhibitor Andecaliximab to block new heterotopic ossification in FOP: Methodology for part 1 of the study

Deborah Wenkert[1], Frederick Kaplan[2], Robert Pignolo[3], Edward Hsiao[4], Mona Al Mukaddam[2], Eric Soliman[1], Victoria Smith[1], Pankaj Bhargava[1]

[1]ashibio, Brisbane, USA [2]Perelman School of Medicine of The University of Pennsylvania, Philadelphia, USA [3]Mayo Clinic College Of Medicine, Rochester, USA [4]University of California - San Francisco, San Francisco, USA

### Abstract

**Background:** Fibrodysplasia ossificans progressiva (FOP), is characterized by progressive transformation of connective tissues and muscle into bone. Matrix metalloproteinase-9 (MMP9) was identified as a novel target for FOP through the study of a "resilient patient" (Patient-R) who at age 35 years (yrs) has had minimal flare-ups or heterotopic ossification (HO). Patient-R carries both the classic ACVR1 FOP mutation (R206H) and 2 matrix metalloproteinase-9 (MMP9) gene variants that result in decreased expression and/or activity of MMP9. In mouse studies MMP9 knock-out abrogates injury-induced HO in FOP mice, and pharmacologic inhibition with MMP9 murine antibody inhibited the development of HO (Lounev 2024). MMP9 inhibition represents a novel strategy to block HO formation in FOP. MMP9 has limited expression in healthy tissues and is associated with chronic inflammation and dysfunctional tissue repair in disease. MMP9 releases factors tethered to the extracellular matrix, which modulate inflammation, neovascularization, and matrix remodeling and appears to be an important factor in the pathogenesis of non-genetic, trauma-induced HO (Evans 2014, Davis 2016). Andecaliximab, a humanized monoclonal antibody to MMP9, has demonstrated a favorable safety profile in prior clinical trials involving approximately 1000 adults. Toxicology studies support the use of andecaliximab in ages ≥ 12 yrs.

**Methods:** ANDECAL is a Phase 2/3, two-Part Study of Andecaliximab in FOP. Part 1 is a Lead-in Study to assess safety, PK/PD and preliminary efficacy, and Part 2 is the Main Study, a Phase 2/3 randomized, double-blind, placebo-controlled trial.

Part 1 (Part 1a & Part 1b) is a double-blind study that randomly assigns up to 12 participants (1:1) to 150 mg or 50 mg andecaliximab administered subcutaneously (SC) once a week (wk). 91 wks extensions of 150 mg SC QW andecaliximab include Patient and Investigator Reported Outcomes (including flare-ups) and radiographic assessments (whole body computed tomography (CT), less head) every 6 months.

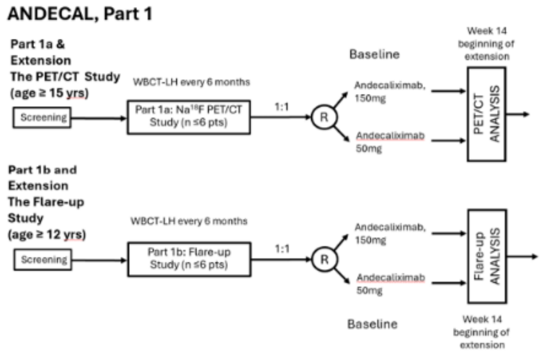

-

**Results and Conclusions:** Enrollment in Part 1 of ANDECAL study is expected to initiate in the second half of 2024. Part 1 of ANDECAL study will evaluate the safety, PK/PD and preliminary efficacy of andecaliximab in participants with FOP and will serve as a lead-in for Part 2 of the study.

### Disclosure

Pankaj Bhargava is employed by ashibio, Deborah Wenkert, Victoria Smith, and Eric Soliman are consultants for ashibio. They have received Salary (PB), stock options (PB, DW, VS), and consulting fees (DW, VS, ES). Frederick S Kaplan is a research investigator for Clementia/Ipsen and Incyte Pharmaceuticals, is a co-inventor on the patent for Methods of Treating HO, which includes MMP-9 as a target, serves on the International FOP Association Medical Registry Advisory Board and is the Founder and Past President of The International Clinical Council (ICC) on FOP. Robert J Pignolo is a co-inventor on the patent for Methods of Treating HO, which includes MMP-9 as a target. Mona M Al Mukaddam is a Research Investigator for Clementia/Ipsen, Incyte, āshibio and a Member of the International Clinical Council on FOP and IFOPA Registry Advisory Board (all voluntary). ECH: Research support: Ascendis, Clementia/Ipsen, Ultragenyx, āshibio; Research Investigator: Ascendis, Clementia/Ipsen, āshibio; Member of the International Clinical Council on FOP, Fibrous Dysplasia Foundation, and IFOPA Registry Advisory Board (all voluntary). Edward C Hsiao: Research support: Ascendis, Clementia/Ipsen, Ultragenyx, āshibio; Research Investigator: Ascendis, Clementia/Ipsen, āshibio; Member of the International Clinical Council on FOP, Fibrous Dysplasia Foundation, and IFOPA Registry Advisory Board (all voluntary).

### LB16

### Hyperphosphatemia in a child with bilateral shin pain

Rachel Bello[1,2], Sapir Bar[3,2], Oded Scheuerman[4,2], Vered Hoffer[4,2], Tal Cohen[4,2], Noa Shefer-Averbuch[1,2,3]

[1]The Jesse Z and Sara Lea Shafer Institute for Endocrinology and Diabetes, National Center for Childhood Diabetes, Schneider Children's Medical Center of Israel, Petach Tikva, Israel. [2]The Faculty of Medicine, Tel Aviv University, Tel Aviv, Israel. [3]Recanati Genetic Institute, Rabin Medical Center–Beilinson Hospital, Petach Tikva, Israel. [4]Department of Pediatrics B, Schneider Children's Medical Center of Israel, Petach Tikva, Israel

### Abstract

BACKGROUND:

Hyperphosphatemic Familial Tumoral Calcinosis (HFTC) is an autosomal-recessive disorder arising from functional deficiency or resistance to intact Fibroblast growth factor 23 (iFGF23). Clinical manifestations include painful subcutaneous masses, peri-articular calcifications, hyperostosis of long bone diaphyses, systemic inflammation, ocular and dental findings.

Decreased iFGF23 leads to stimulation of sodium phosphate co-transporters and 25-OH-vitamin D3-1-α–hydroxylase in the proximal tubule. Hyperphosphatemia ensues from increased renal tubular reabsorption of phosphate and increased gastrointestinal absorption of phosphate and calcium. The increased calcium x phosphate product contributes to ectopic calcifications.

PRESENTING PROBLEM:

A 7-year-old girl with consanguineous parents of Bedouin descent, presented with bilateral shin pain and swelling.

During the first episode left tibia was involved. Extensive investigation for osteomyelitis revealed no infectious cause. Due to suspected bone or mesenchymal tumor, she underwent bone biopsy which showed reactive nonspecific changes, bone marrow biopsy was normal and total body imaging did not demonstrate a mesenchymal tumor.

Six months later she was presented to our hospital with right tibial pain and swelling. Laboratory investigations showed hyperphosphatemia (8.5mg/dL) with normal serum calcium, alkaline phosphatase and renal function tests. Low parathyroid hormone with elevated 1,25OH2D and increased renal tubular reabsorption of phosphate (100%) were noted. Skeletal X-rays showed hyperostosis and periosteal reaction in the tibial diaphysis without any peri-articular soft tissue calcifications. Ophthalmologic examination revealed conjunctival calcifications. Nevertheless, no vascular or renal calcifications and no classical dental features were noticed. Clinical diagnosis of HFTC was done.

CLINICAL MANAGEMENT:

The child was treated with a low-phosphate diet and Sevelamer to lower serum phosphate level, and Acetaminophen, NSAID's and Tramadol to control her pain. Genetic analysis revealed a novel homozygous variant in FGF23 gene.

DISCUSSION:

HFTC is a rare disorder of phosphate homeostasis. Clinical manifestations of long bone pain with hyperostosis and periosteal reaction should raise the possibility of this diagnosis, emphasizing the necessity of measuring serum phosphate levels.

HFTC affects dental development of permanent tooth roots most severely at ages 9–11 years, which might explain the normal dental panoramic radiograph at the age of 7 years.

### LB17

### Case report of McCune–Albright syndrome

Larysa Iatsun[1], Nataliya Balatska[2]

[1]National Specialized Children's Hospital "OHMATDYT", Kyiv, Ukraine. [2]Bogomolets National Medical University, Kyiv, Ukraine

### Abstract

McCune-Albright syndrome (MAS) is typically characterized by a trio of symptoms: fibrous dysplasia of bone (FD), café-au-lait skin spots, and precocious puberty (PP). It is a rare disorder with an estimated prevalence of between 1/100,000 and 1/1,000,000. FD can affect one or multiple skeletal sites and often presents with limping and/or pain, and occasionally, a pathological fracture.

**Case Report:**

This case report describes a girl from Kherson, Ukraine, who was receiving medical care in Kharkiv. At two weeks old, she developed café-au-lait skin macules with jagged borders that fully formed by four months. Vaginal bleeding began at two months. At seven years old, she suffered a fracture in her left femur and was subsequently treated for osteomyelitis. The onset of the full-scale invasion of Ukraine by russia severely disrupted her access to consistent and specialized medical care, impacting her ability to receive timely treatments and follow-up care, thus exacerbating the management challenges of her condition.

At the age of 9 years and 7 months, the patient was admitted to our clinic with complaints of pain in the lower extremities and periodic menstrual bleeding. Clinical examination revealed a height of 149 cm (+2.0 SD), a weight of 53 kg, and a BMI of 23.8 kg/m² (above the 97th percentile). Sexual development, according to Tanner staging, was Ax4P4Ma2Me2. Also noted was a valgus deformity of the knees. Treatment included cyproterone 50 mg per day, alphacalcidol 1 mcg per day, calcium 1000 mg per day, and vitamin D3 2000 IU per day, although her intake was irregular due to the ongoing conflict. She did not receive phosphate supplements.

Laboratory investigations showed normal ionized calcium levels (1.31 mmol/L; normal range: 1.1-1.35 mmol/l), decreased phosphorus levels (0.90 mmol/l; normal range: 1.32–2.0 mmol/l), and elevated alkaline phosphatase levels (1725 U/l; normal range: 69-325 U/l). Hormonal tests indicated increased estradiol (58.80 pg/ml; normal range: 6-27 pg/ml), prolactin (38.18 ng/ml; normal range: 0.3–12.9 ng/ml), and slightly elevated IGF-1 (399.9 ng/ml; normal range: 99.9–363 ng/mL). Hand X-rays indicated a biological age of 14 years with a heterogeneous bone structure due to alternating areas of sclerosis and dilution. A DEXA scan showed no decrease in bone mineral density.

Dynamics revealed a sustained increase in alkaline phosphatase (1584 U/L; normal range: 69-325 U/l) and a decrease in the calcium-creatinine ratio (0.004; normal range: 0.04-0.7). The phosphorus reabsorption rate in the renal tubules was 94.6% (normal range: 85-95%), and the maximum phosphorus reabsorption to glomerular filtration rate (TmP/GFR) was 0.82 (normal range: 0.97-1.64). During this period, the girl suffered a fracture of the tibia.

**Discussion:**

The patient exhibits classic signs of MAS, including fibrous dysplasia of bones, café-au-lait skin macules, and precocious puberty. Elevated levels of alkaline phosphatase, disruptions in bone mineral metabolism, and hormonal imbalances are also observed. Treatment complexities are further exacerbated by the ongoing military conflict, affecting the regularity of medication intake and access to specialized medical care. The lack of phosphate supplementation, despite the presence of MAS-related hypophosphatemic rickets, underscores the challenges in managing her condition effectively. It is crucial to ensure regular monitoring of the patient's condition, strict adherence to treatment protocols, psychological support, and coordination with humanitarian organizations to ensure continuous access to necessary medications and medical assistance. This case highlights the complexities of managing a rare genetic disorder like MAS under normal circumstances, further complicated by war-induced disruptions in healthcare services. The patient's situation underscores the critical need for stable and accessible healthcare infrastructure, especially for individuals with chronic and complex conditions.

### LB18

### Comparative analysis of anthropometric and metabolic profiles in achondroplasia and hypochondroplasia: Insights from a Brazilian pediatric cohort

João Victor Riposati Canedo[1], Fabiana Benedini Galli Zambardino[1], Milena Franco de Pontes[1], Patricia Salmona[2], Carolina Costa Figueiredo[2], Nara Michele Evangelista de Araújo[2], Vânia de Fátima Tonetto Fernandes[2], Sabrina de Miguel Augusto[1], Milena Santos Aguiar[3], Guido de Paula Colares Neto[1]

[1]Centro Universitário São Camilo, São Paulo, Brazil. [2]Hospital Infantil Darcy Vargas, São Paulo, Brazil. [3]Universidade de São Paulo, São Paulo, Brazil

### Abstract

**Objective:** To evaluate anthropometric and metabolic data in patients with achondroplasia and hypochondroplasia in a Brazilian pediatric cohort.

**Methods:** A descriptive cross-sectional study conducted at a tertiary pediatric hospital. Eighteen patient records were evaluated, and the patients were divided into two groups based on confirmatory molecular diagnosis. Clinical, anthropometric, and laboratory data were analyzed through medical records.

**Results:** Fifteen patients (six females and nine males), aged 7.2 [0.1; 16] years, were diagnosed with achondroplasia. All exhibited pathogenic missense variants in heterozygosity in the *FGFR3* gene (c.1138G>A; p.Gly380Arg), with seven familial and eight sporadic cases. Three patients (2 females and one male), aged 8.6 [8.4; 11.9], were diagnosed with hypochondroplasia, and the missense variants in heterozygosity in the *FGFR3* gene differed among them: c.1620C>A (p.Asn540Lys), c.1620C>G (p.Asn540Lys), and c.1950G>T (p.Lys650Asn), with all cases being sporadic. There was no difference between groups in gestational age, length, weight, and head circumference at birth. According to the growth curves for achondroplasia, patients with achondroplasia showed a lower height Z-score than patients with hypochondroplasia (-0.73±1.33 vs. 2.16±1.3; p<0.01), while the weight Z-score (-0.00±2.05 vs. 1.42±1.48; p=0.28) and body mass index Z-score (2.57±2.53 vs. 0.92±2.57; p=0.32) showed no significant differences. Patients with achondroplasia tended to have a greater difference between arm span and height (11.2±3.7cm vs. 7.3±2.3cm; p=0.07) and a higher upper segment/lower segment ratio than patients with hypochondroplasia (1.7±0.34cm vs. 1.2±0.05cm; p<0.01). No differences were seen between groups in the prevalence of genu varum and thoracolumbar kyphosis. Although there was no difference in the prevalence of obesity between groups, 60% of patients with achondroplasia and 66.7% of patients with hypochondroplasia were overweight. One (6.6%) patient with achondroplasia had altered glycated hemoglobin levels. Two (13.3%) patients with achondroplasia had hypercholesterolemia and low HDL. One (6.6%) patient with achondroplasia had hypertriglyceridemia.

**Conclusion:** Compared to neonatal anthropometry, postnatal height, the difference between arm span and height, and the upper segment/lower segment ratio were essential for the differential diagnosis between achondroplasia and hypochondroplasia. Although obesity was prevalent in both groups, no evidence of significant metabolic repercussions was observed, necessitating a more detailed analysis considering their body composition.

### LB19

### Utilizing pamidronate presents a novel approach to restoring bone tissue in the surgical management of tuberculous osteomyelitis

Veronika Petukhova[1], Alexander Mushkin[1], Tatiana Vinogradova[1], Mikhail Kostik[2]

[1]Saint-Petersburg Research Institute of Phthisiopulmonology, Saint-Petersburg, Russian Federation.
[2]Saint-Petersburg State Pediatric Medical University, Saint-Petersburg, Russian Federation

### Abstract

**Objective:** To validate the efficacy of pamidronic acid in bone restoration during the surgical treatment of experimental tuberculous osteomyelitis (TB-OM).

**Methods:** Clinical, radiological, morphological, and morphometric characteristics were studied using an experimental TB-OM model of the femur condyle (M. tub. H37Rv) in 21 rabbits. The rabbits were divided into four groups: control group (no treatment), group 2-4 underwent radical surgical removal of the focus and implant plasty with 2DBM, supplemented as follows: group 2-anti-TB drugs, group 3-anti-TB drugs and a single injection of PAM 1 mg/kg, group 4-only PAM. Autopsy was conducted at 3 and 6 months post-surgery to assess implantation zone features, bone tissue area, bone beam thickness, osteoblasts, osteocytes, osteoclasts, and their active forms.

**Results:** The use of PAM: a) aided implant adaptation by preventing premature lysis (CT detection of the implant in 85.7 - 100% of cases in groups 3 and 4 after 3 and 6 months (fig. 1), 50% in group 2 after 3 months; 0% after 6 months); and maintaining its osteoconductive and osteoinductive properties (implant ingrowth by bone beams in 100% of cases in groups 3 and 4 according to CT and morphology (fig. 1), formation of stromal cords and numerous growing bone beams around the implant); b) demonstrated safety in the postoperative period, did not increase TB-OM progression risk, and was compatible with anti-TB drugs (no adverse reactions, deaths, reduced specific inflammation in 50-75% of cases in groups 2-4 during the experiment, $p<0.05$); c) prolonged induction of osteogenesis (increase in active osteoblast proportion in perifocal zones by 6 months in groups 3 and 4, $p<0.05$); e) led to increased bone tissue area and bone beam thickness (group 4 showed maximum values of % of bone tissue area in the epiphysis and beam thickness after 3 and 6 months, groups 3 and 4 showed higher beam thickness values compared to group 2 after 3 months, $p<0.05$).

**Conclusion:** Experimental evidence supports the effectiveness of pamidronic acid in the surgical management of tuberculous osteomyelitis.

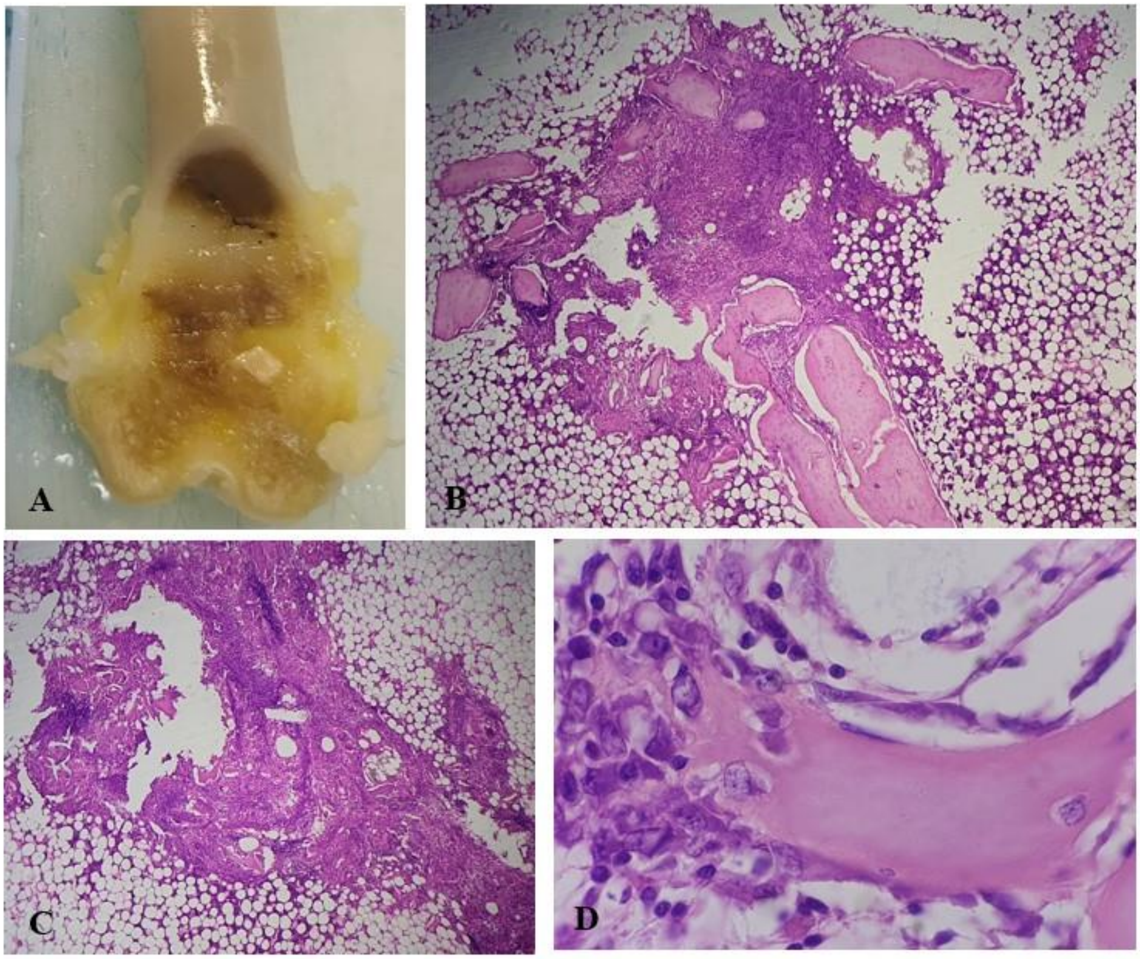

Fig. 1 Macro- (A) and microphotographs of group 4, stained with hematoxylin and eosin (magnification 100x) (B-D). In the area of the epiphysis closer to the articular surface, implant granules are visualized, around which zones of nonspecific inflammation, represented mainly by lymphoplasmacytic infiltration, are identified. In the same zone, growing beams with active osteoblasts on the surface and full-blooded vessels are found. Around the foci of inflammation, bone marrow remodeling is active mesenchyme, granulation tissue of varying maturity with an increased number of full-blooded vessels.

### LB20

### The diversity of bone microbiom in children with different forms of osteomyelitis

Mikhail Kostik[1], Veronika Petukhova[2], Alexei Maletin[2], Lavrentii Danilov[3], Vasilisa Dudurich[4], Oleg Glotov[5], Lubov Sorokina[1], Eugenia Isupova[1], Alexander Mushkin[2,6]

[1]Saint-Petersburg State Pediatric Medical University, Saint-Petersburg, Russian Federation. [2]Saint-Petersburg Research Institute of Phthisiopulmonology, Saint-Petersburg, Russian Federation. [3]Saint-Petersburg State University, Saint-Petersburg, Russian Federation. [4]Cerbalab Laboratory, Saint-Petersburg, Russian Federation. [5]Pediatric Research and Clinical Center for Infectious Diseases, Saint-Petersburg, Russian Federation. [6]First Saint-Petersburg State Medical University n.a. academician Pavlov, Saint-Petersburg, Russian Federation

### Abstract

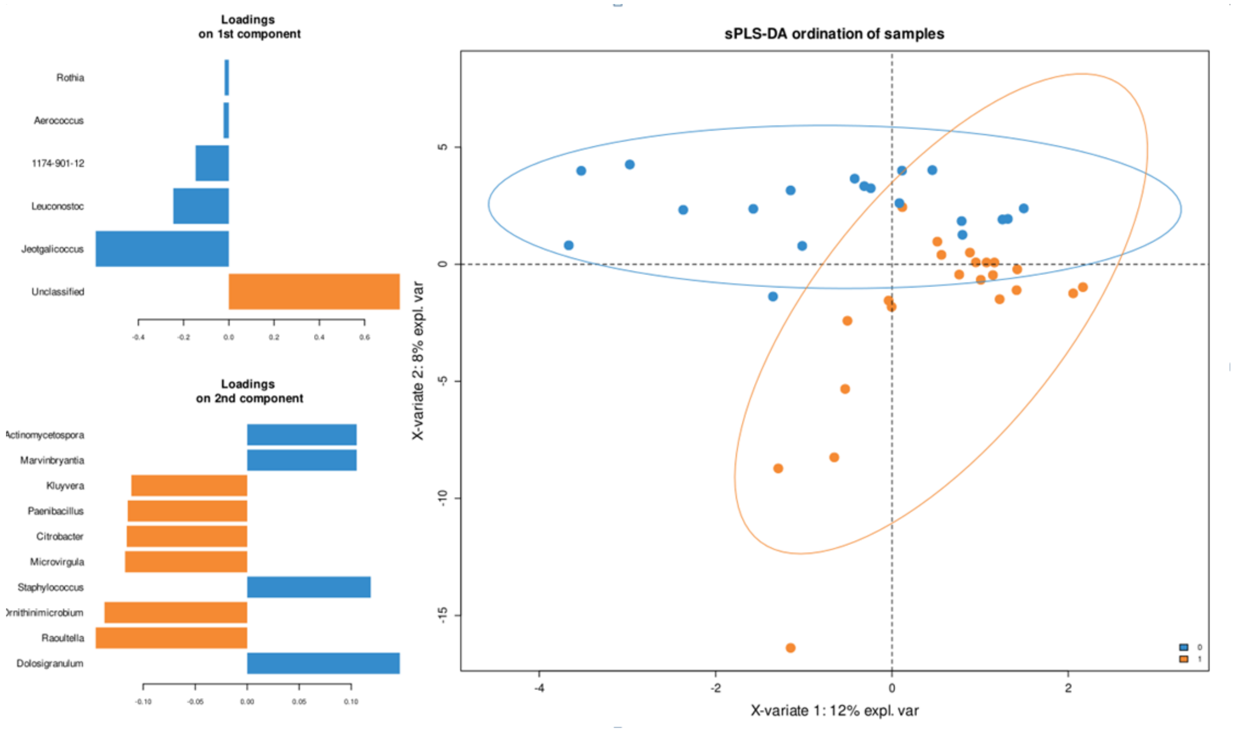

### Introduction

Bone inflammatory diseases are the heterogenous group with infection and non-infection etiology. The infection osteomyelitis are hematogenous osteomyelitis (HO) and tuberculosis osteomyelitis (TBO) and chronic nonbacterial osteomyelitis (CNO) is an immune-mediated disease with unknown etiology with possible combination of environmental, including low-virulent infection and genetic factors interaction, despite the name “nonbacterial”. Some previous studies suspected some bacteria, e.g. Propionobacteria as a possible ethiological factor for the CNO.

### Aim

The study aimed to evaluate the differences of bone mircobiom in children with bone inflammatory diseases.

**Methods**

In the prospective study we included children with CNO (n=13), TBO (n=21) and HO (n=5). The diagnosis of CNO was made with 2007 and 2016 criteria with negative culture from the bone; TBO was confirmed by culture and/or tissue PCR and HO according acute clinical course, high CRP, positive culture (St.aureus in all cases) and positive effect of antibiotics. In all cases the total DNA was isolated during diagnostic bone biopsy or bone surgery and was subjected to homogenization in a lysing solution. Homogenization was carried out together with the beads, followed by DNA extraction by the sorbent column method (Qiagen, Germantown, MD, USA) in accordance with the manufacturer's recommendations.

Libraries for 16S DNA sequencing were prepared in accordance with the Illumina protocol for the preparation of 16S metagenomic libraries for sequencing (Part #15044223 Rev. B). Identification of bacterial species was performed using the exact matching algorithm in DADA2 using SILVA v138 sequences pre-processed in an appropriate way using the custom scripts. To identify special taxa for each group, sparse partial least squares discriminant analysis (sPLS-DA) was carried out with the “multiomix” software (mixOmics software package) in the R programming language.

**Results**

The CNO group is characterized by an increase in such genera of bacteria as Halomonas, Anaerobacillus, Bacillus, especially Acinetobacter ursingii, Pseudomonas aeruginosa, Acinetobacter haemolyticus. The TBO group is characterized by a decrease in such bacterial species as *Acinetobacter baumannii, Sphingomonas insulae, Stenotrophomonas maltophilia.* The HO group is characterized by a decrease in such genera of bacteria Serratia, Bosea, Nissabacter and decrease of *Dermacoccus nishinomiyaensis, Sphingobacterium cladoniae, Pseudomonas mendocina*.

**Conclusion:**

Children with bone inflammatory disease have unique bone bacterial microbiom profile. Further investigations are needed.

### LB21

### Protocol for enhanced glucocorticoid induced osteoporosis screening in DMD patients receiving gene therapy

Virginia Kaperick

Medical College of Wisconsin, Milwaukee, USA

### Abstract

**Background:**

Elevidys (delandistrogene moxeparvovec) was FDA approved in June 2023 as the first gene therapy for Duchenne Muscular Dystrophy (DMD). Treatment with supraphysiologic steroids is required for tolerance of the viral vector. It is essential that these patients receive enhanced screening for Glucocorticoid Induced Osteoporosis (GIO) before, during, and after this therapy.

**Emerging Issue:**

Elevidys is FDA approved for ages 4-5 years and on track to expand to other ages. Younger children with DMD remain ambulatory, yet with progressive weakness and poor balance they develop increased vulnerability to falls. Early adverse bone health effects from DMD are common due to myopathy and chronic inflammation from underlying muscle breakdown, compounded by the start of standard glucocorticoid therapy. Additional high-dose steroids of 1 mg/kg/day for at least 8-12 weeks as part of gene therapy protocols increases the risk for fractures.

**Discussion:**

GIO results from multiple direct osteotoxic effects of steroids, decreased vitamin D and calcium absorption, increased vitamin D catabolism, poor growth and/or cushingoid features. Glucocorticoids have a strong predilection for the microarchitecture of trabecular bone leading to increased risk of vertebral fractures, that are otherwise quite rare in children. Falls (particularly onto the buttocks) can result in painful or asymptomatic vertebral fractures.

Proactive vitamin D supplementation can help limit the drop in vitamin D levels, which frequently occurs secondary to steroids. This approach is preferred over a reactive approach as supplementation takes time to replete. Calcium intake should also be optimized given the body’s priority to maintain strict homeostasis to the detriment of bone mineral content. Dual X-ray Absorptiometry does not reliably reflect GIO changes, and often Bone Mineral Density (BMD) falls within low normal range. Evidence of vertebral fracture on imaging prompts the start of bisphosphonate therapy regardless of BMD.

**Clinical Management:**

Vitamin D and calcium optimization during this high-risk time are easily modifiable factors. Close screening for vertebral fractures should be utilized, as early recognition and subsequent bisphosphonate therapy can prevent onset of chronic pain and vertebral fracture cascade.

I developed the following screening and management protocol for Children's Wisconsin, which is readily adaptable to other institutions:

**Enhanced Glucocorticoid Induced Osteoporosis Screening**

**in DMD Patients Receiving Gene Therapy**

| | Gene Therapy<br><br>Pre-Screen | Pre-infusion<br><br>Day -2, -1, 0 | Post infusion<br><br>Day 28 | Post infusion<br><br>Day 90 | Post infusion<br><br>Day 180 | Post infusion<br><br>Day 270 | Post infusion<br><br>Day 365 | PRN |
|---|---|---|---|---|---|---|---|---|
| **DXA:**<br><br>**whole body and lumbar spine** | X<br><br>If not done in last 6 months | or X<br><br>If not at screen | | | X | | Then per routine protocol | Back pain if > 6 mo since last DXA |
| **Lateral Spine Xray** | X<br><br>If not done in last 6 months | or X<br><br>If not at screen | PRN<br><br>back pain | PRN<br><br>back pain | If back pain; or VFA not avail or adequate. | | Then per routine protocol | Back pain |
| **VFA**<br><br>**if available** | X<br><br>If not done in last 6 months | or X<br><br>If not at screen | | | X | | Then per routine protocol | Back pain<br><br>if > 6 mo since last VFA |
| **25 OH-Vit D level** | X | X | X | X | X | If decreased supplementDay 180 | X | very low/ high level, or<br><br>compliance issues |

**Goal Vitamin D = 40-70 ng/dl with appropriate calcium intake x 6 months**

**given increased risk of Glucocorticoid Induced Osteoporosis.**

| **25 OH-Vit D**<br><br>**ng/dl** | 0-20 | 21-30 | 31-40 | >70 | 41-70 | After Day 180 |
|---|---|---|---|---|---|---|
| **mmol/L** | 0-50 | 52.5-75 | 77.5-100 | 175 | 102.5-175 | |

| | | | | | | |
|---|---|---|---|---|---|---|
| **Vit D supplement**<br><br>**Dose** | 50,000 IU tablet weekly x 12 weeks<br><br>(or ~ 6000 IU daily for drops/ liquid) | Add<br><br>2000 IU | Add<br><br>1000 IU | Decrease Vit D supplement<br><br>by 1000 IU for every 10 ng/dl (25 mmol/L) decrease<br><br>planned | IF 40-70<br><br>Do NOT decrease supplement until after Day 180 | Treat per routine protocol |
| **Vit D If significant obesity** | May need longer treatment | Add<br><br>4000 IU | Add<br><br>2000 IU | | | |
| **Calcium intake recs** | Age 0-3 yrs | Age 4-8 yrs | Age 9 yrs & up | | | |
| **Diet + suppl** | 700 mg | 1000 mg | 1300 mg | | | |
